# Supplementary figures and images for: Research on the correlation between retinal vascular parameters and axial length in children using an AI-based fundus image analysis system (part 2 of 3)
Source: PLoS One. 2025 Jun 17;20(6):e0324352. doi: 10.1371/journal.pone.0324352 (PMC12173413; doi:10.1371/journal.pone.0324352)

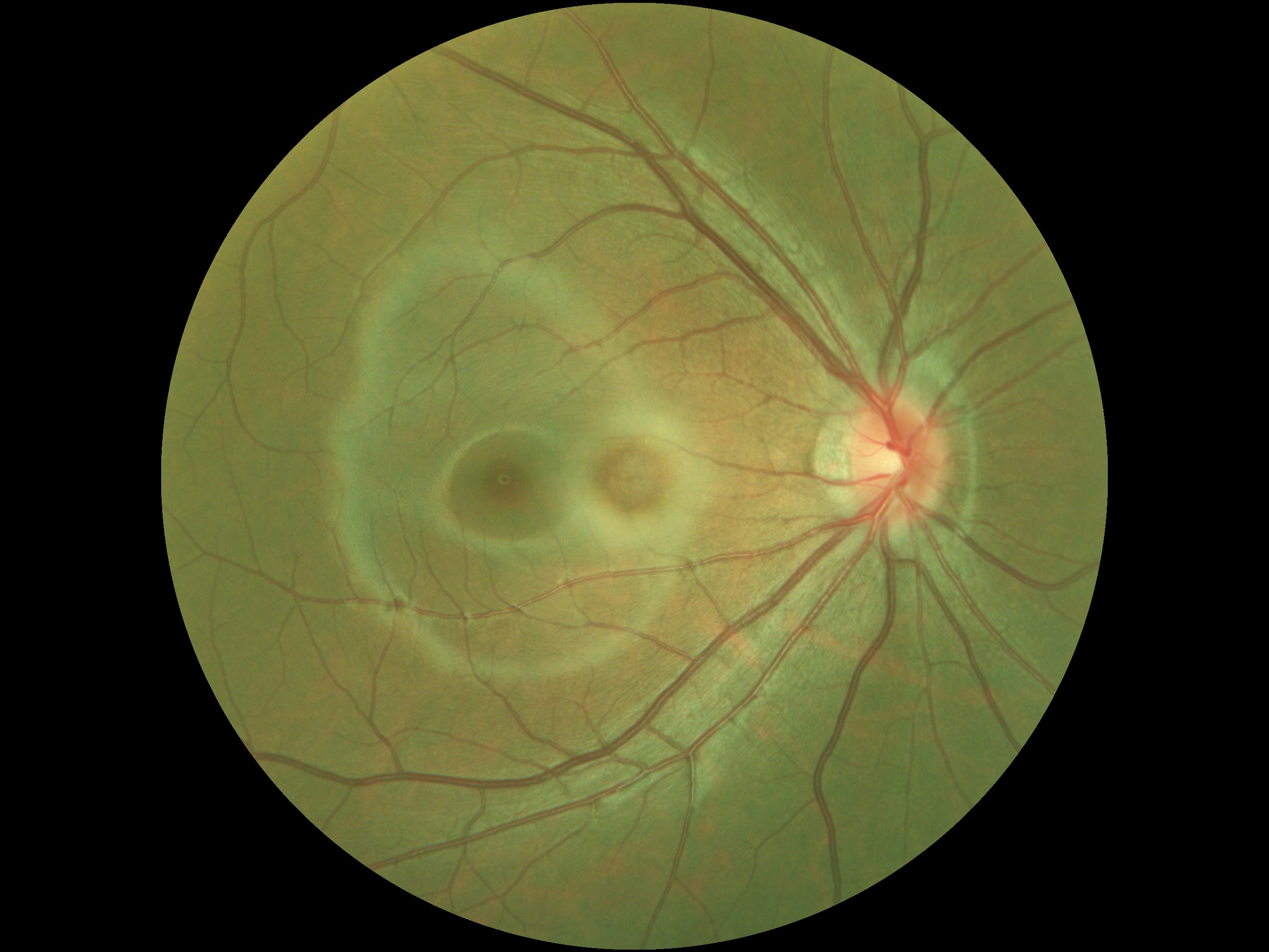

Supplement: S3 File — (ZIP) [file pone.0324352.s003.zip › Original fundus photographs (1)/Subject 55/OD_20230615564082_20230615160249_1.jpg]

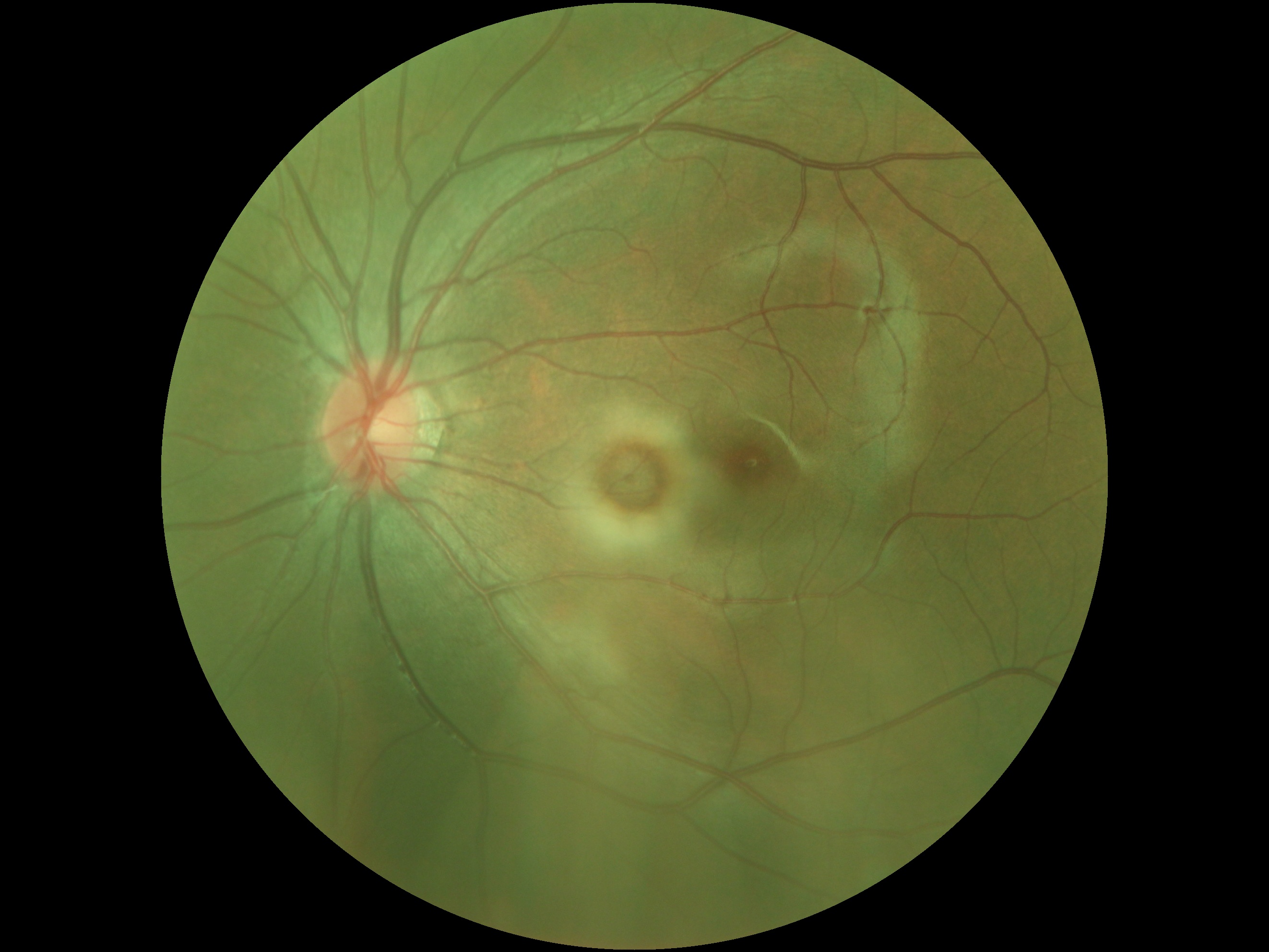

Supplement: S3 File — (ZIP) [file pone.0324352.s003.zip › Original fundus photographs (1)/Subject 55/OS_20230615564082_20230615160320_3.jpg]

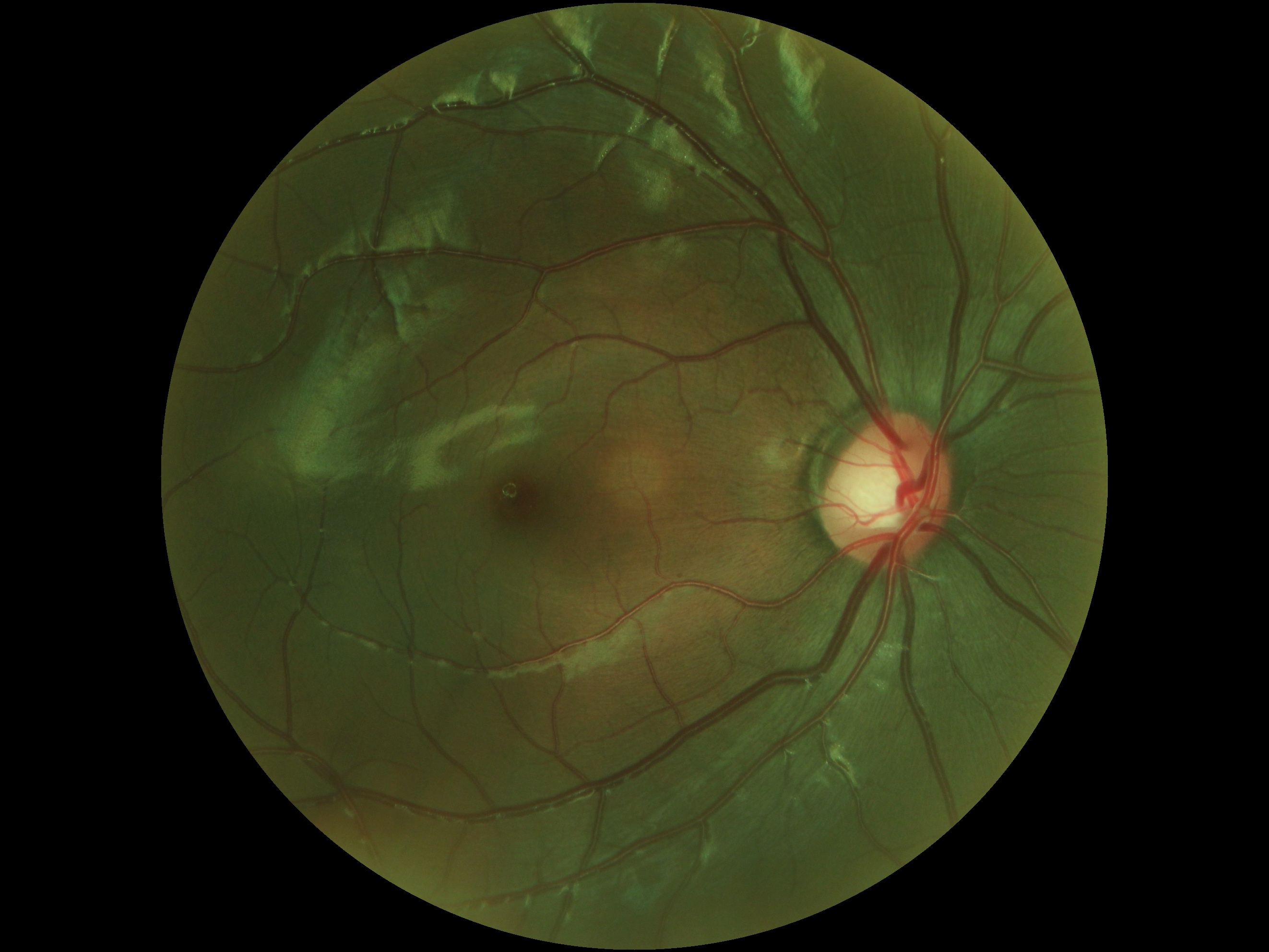

Supplement: S3 File — (ZIP) [file pone.0324352.s003.zip › Original fundus photographs (1)/Subject 56/OD_20230611610021_20230612113200_2.jpg]

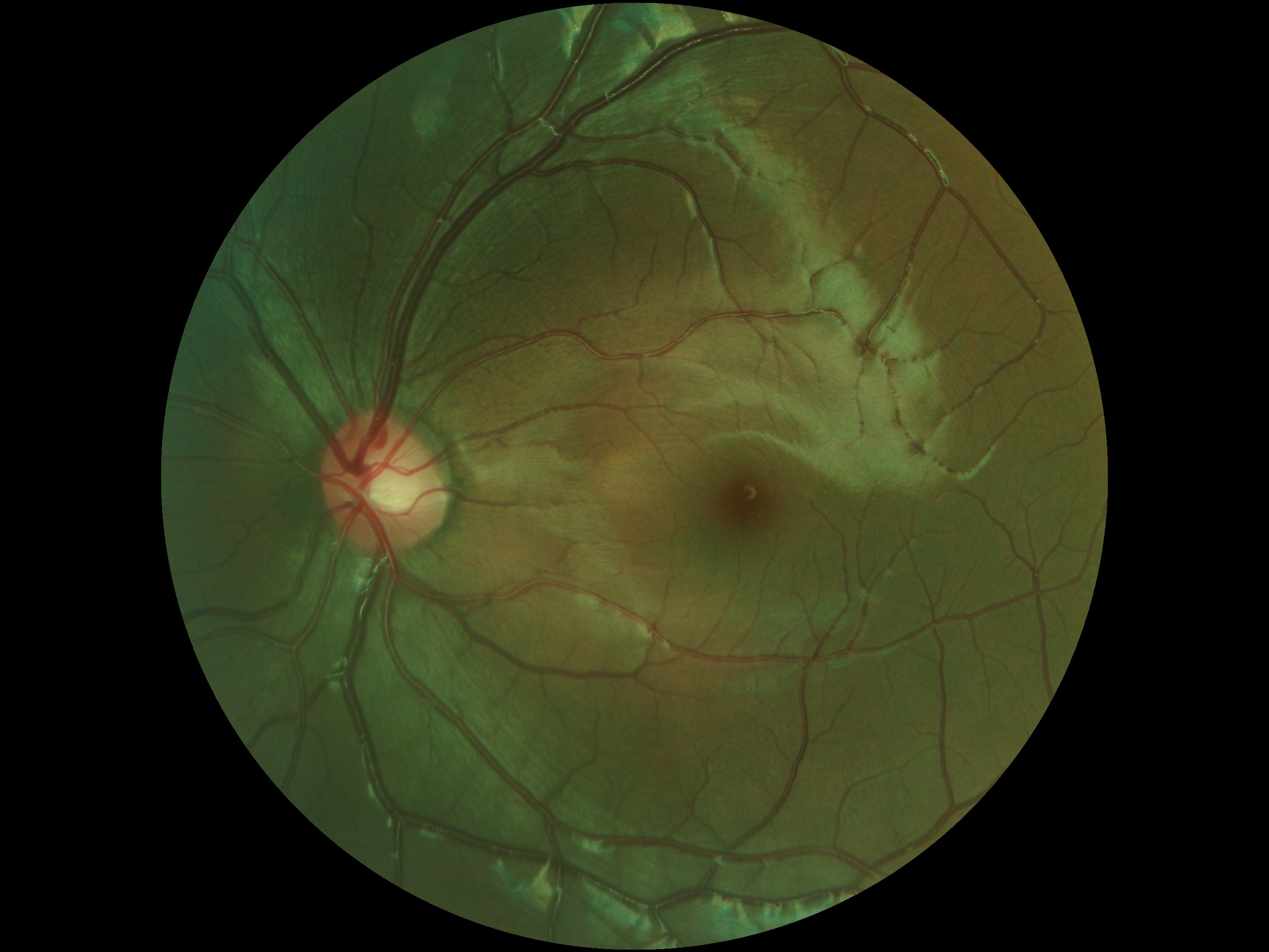

Supplement: S3 File — (ZIP) [file pone.0324352.s003.zip › Original fundus photographs (1)/Subject 56/OS_20230611610021_20230612113316_3.jpg]

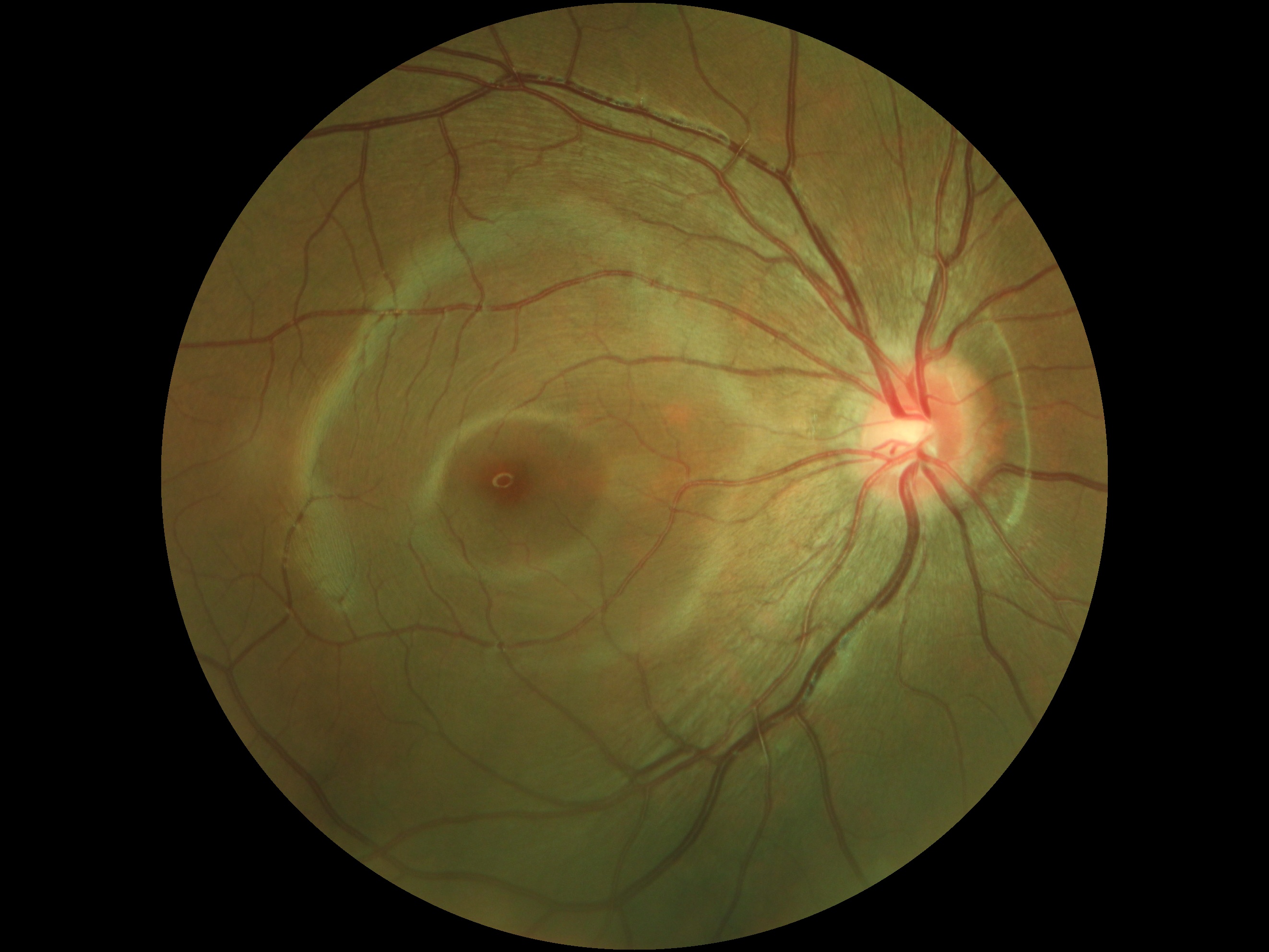

Supplement: S3 File — (ZIP) [file pone.0324352.s003.zip › Original fundus photographs (1)/Subject 57/OD_20230611543189_20230613105514_3.jpg]

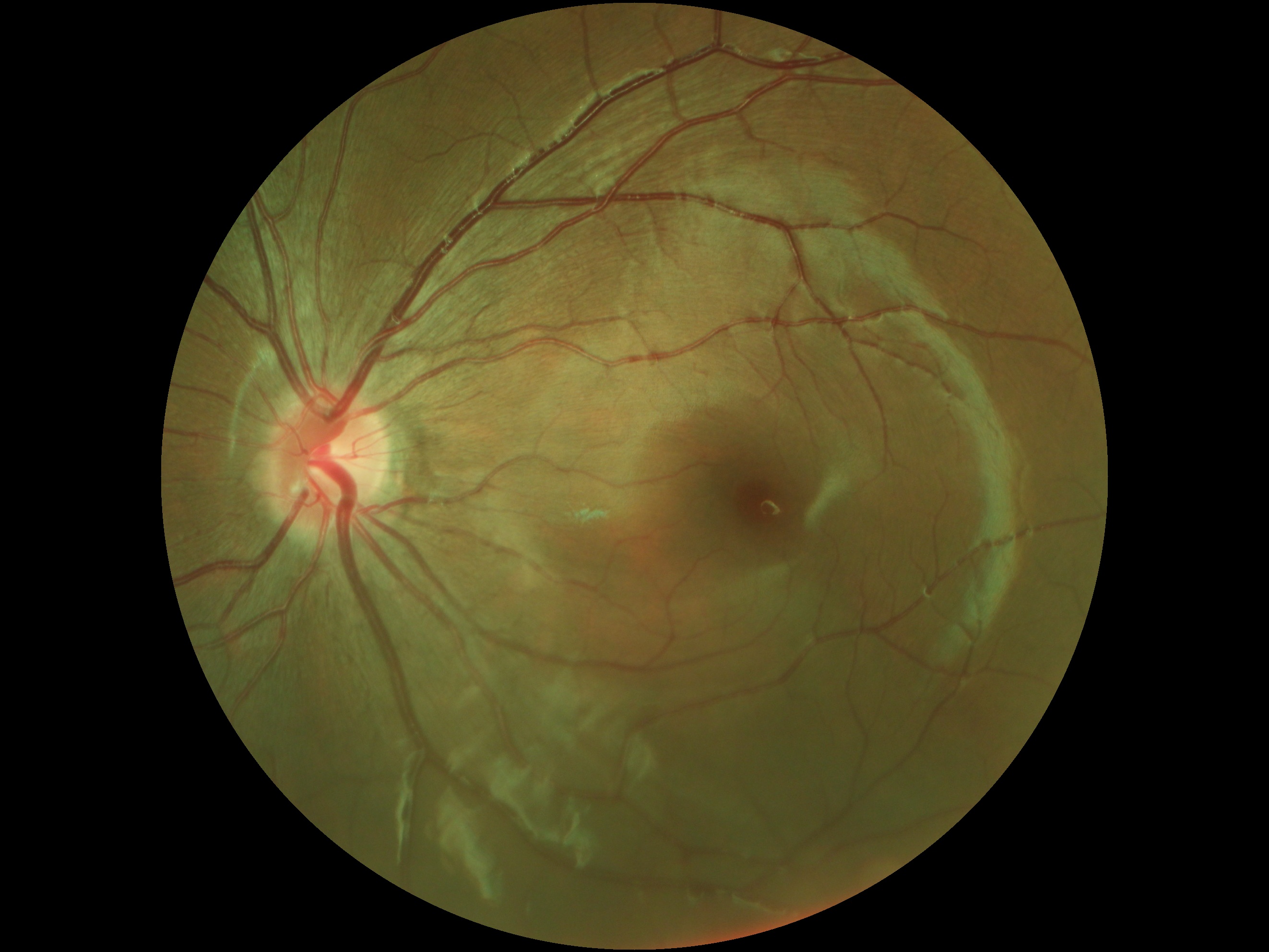

Supplement: S3 File — (ZIP) [file pone.0324352.s003.zip › Original fundus photographs (1)/Subject 57/OS_20230611543189_20230613105614_4.jpg]

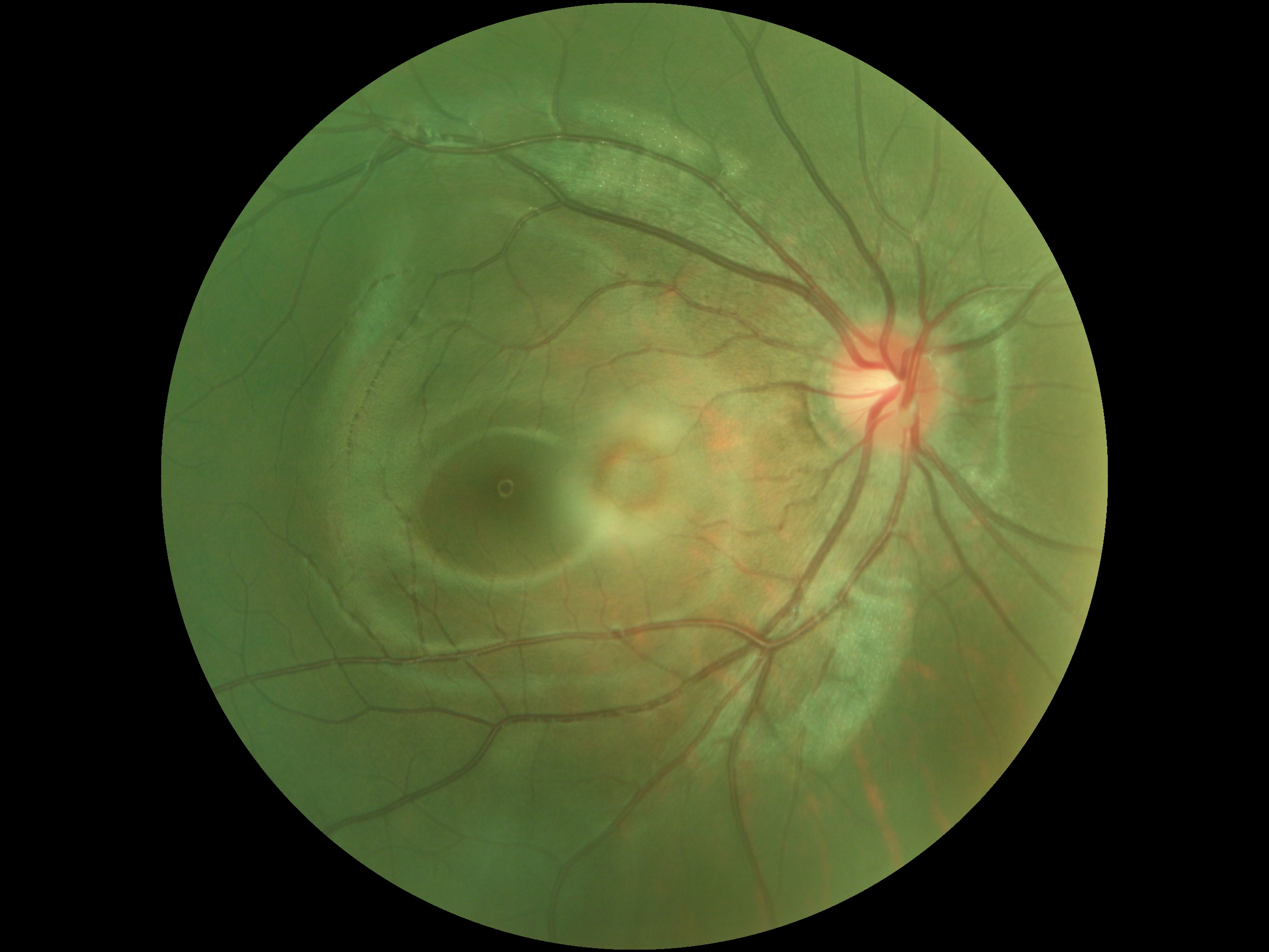

Supplement: S3 File — (ZIP) [file pone.0324352.s003.zip › Original fundus photographs (1)/Subject 58/OD_20230615627083_20230615160130_2.jpg]

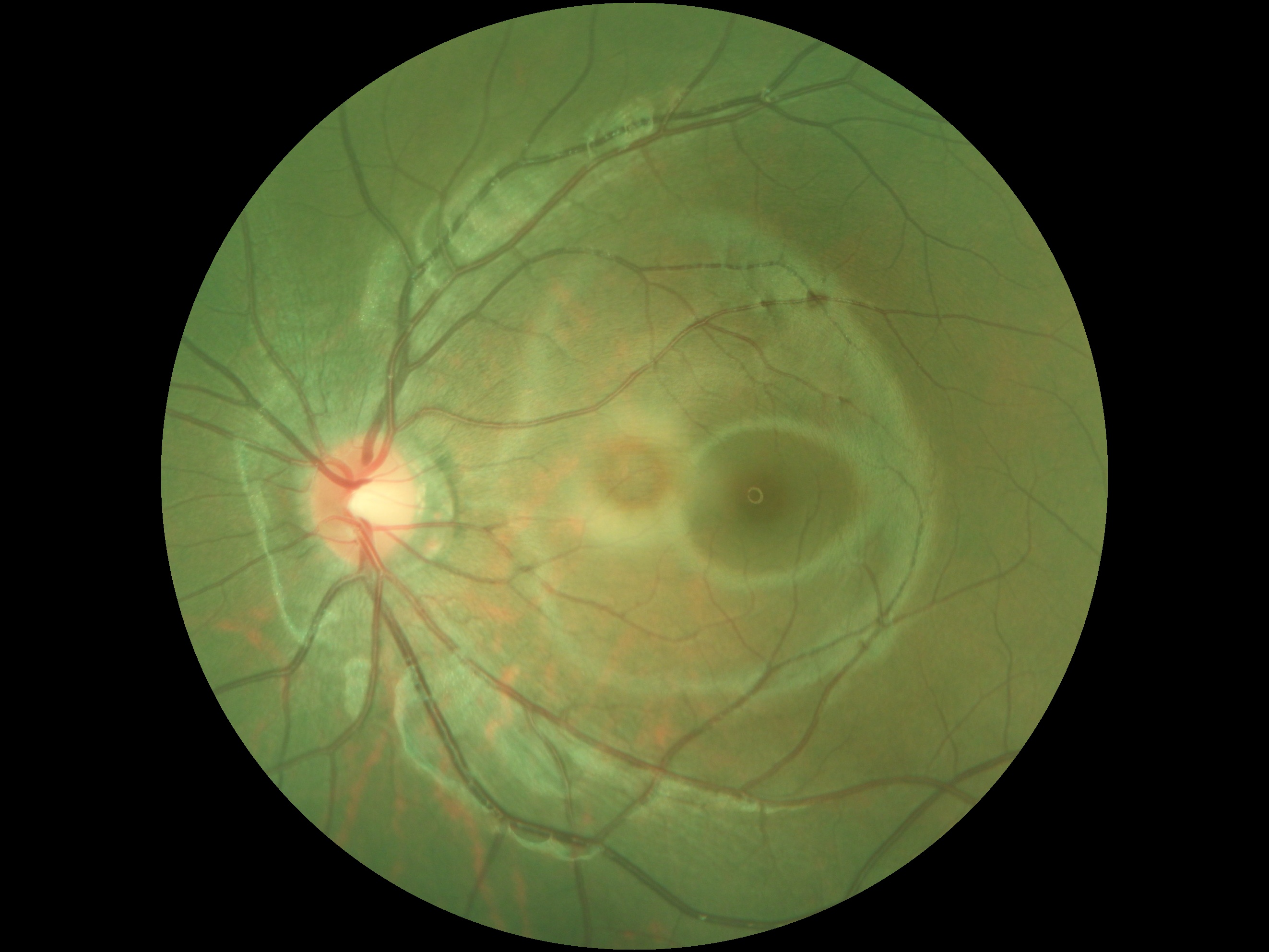

Supplement: S3 File — (ZIP) [file pone.0324352.s003.zip › Original fundus photographs (1)/Subject 58/OS_20230615627083_20230615160212_3.jpg]

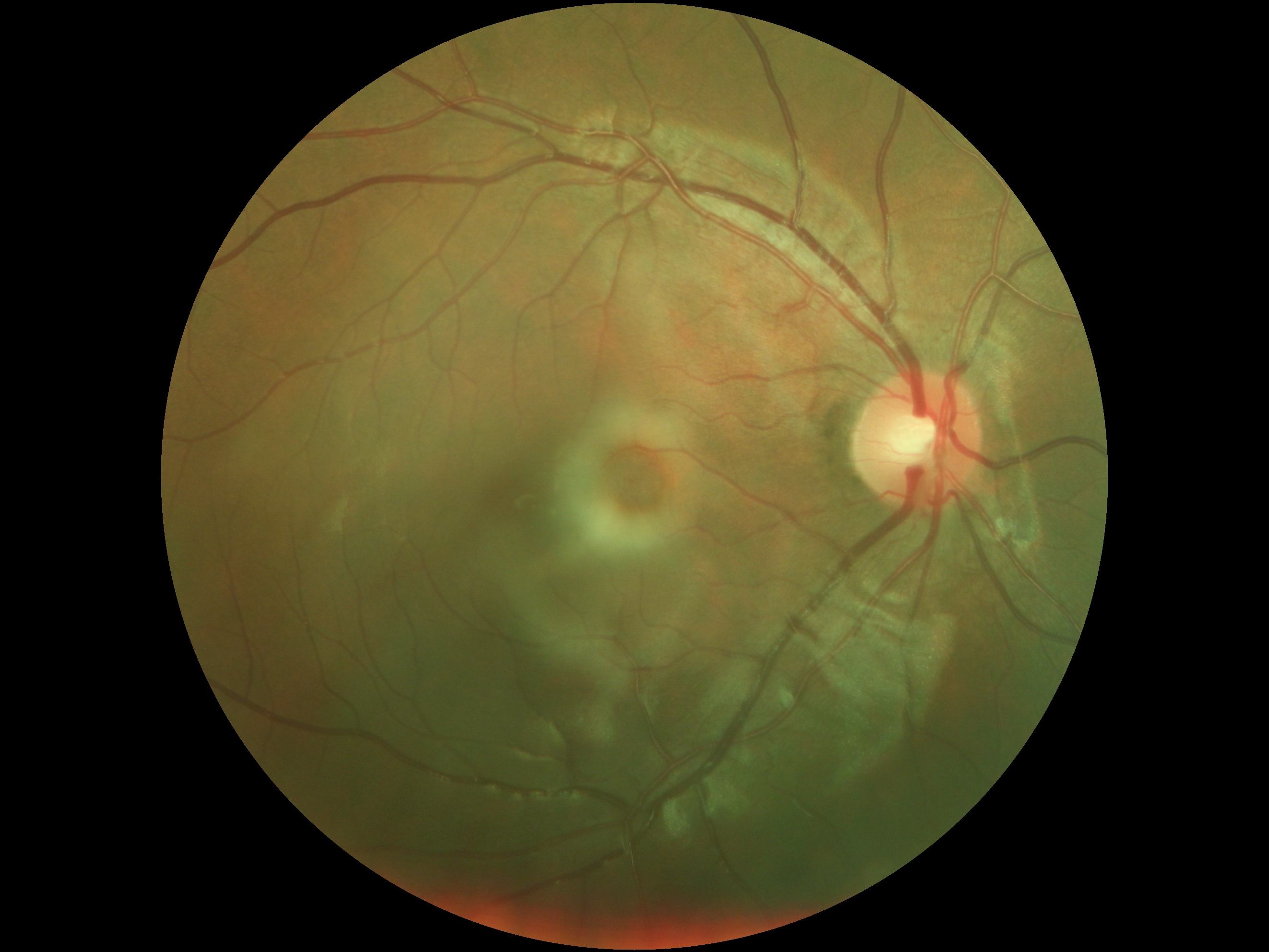

Supplement: S3 File — (ZIP) [file pone.0324352.s003.zip › Original fundus photographs (1)/Subject 59/OD_20230611786192_20230615114027_1.jpg]

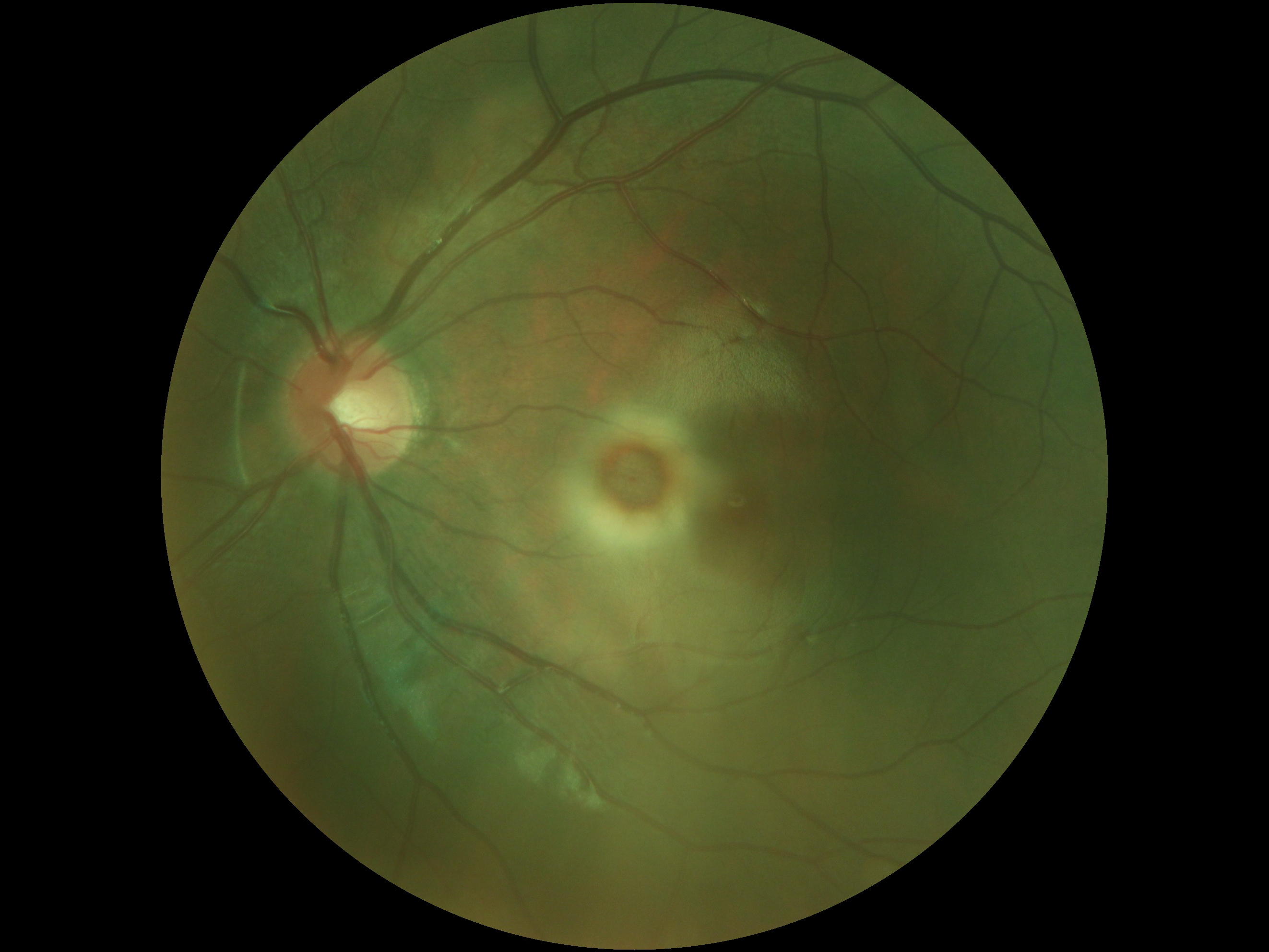

Supplement: S3 File — (ZIP) [file pone.0324352.s003.zip › Original fundus photographs (1)/Subject 59/OS_20230611786192_20230615114152_4.jpg]

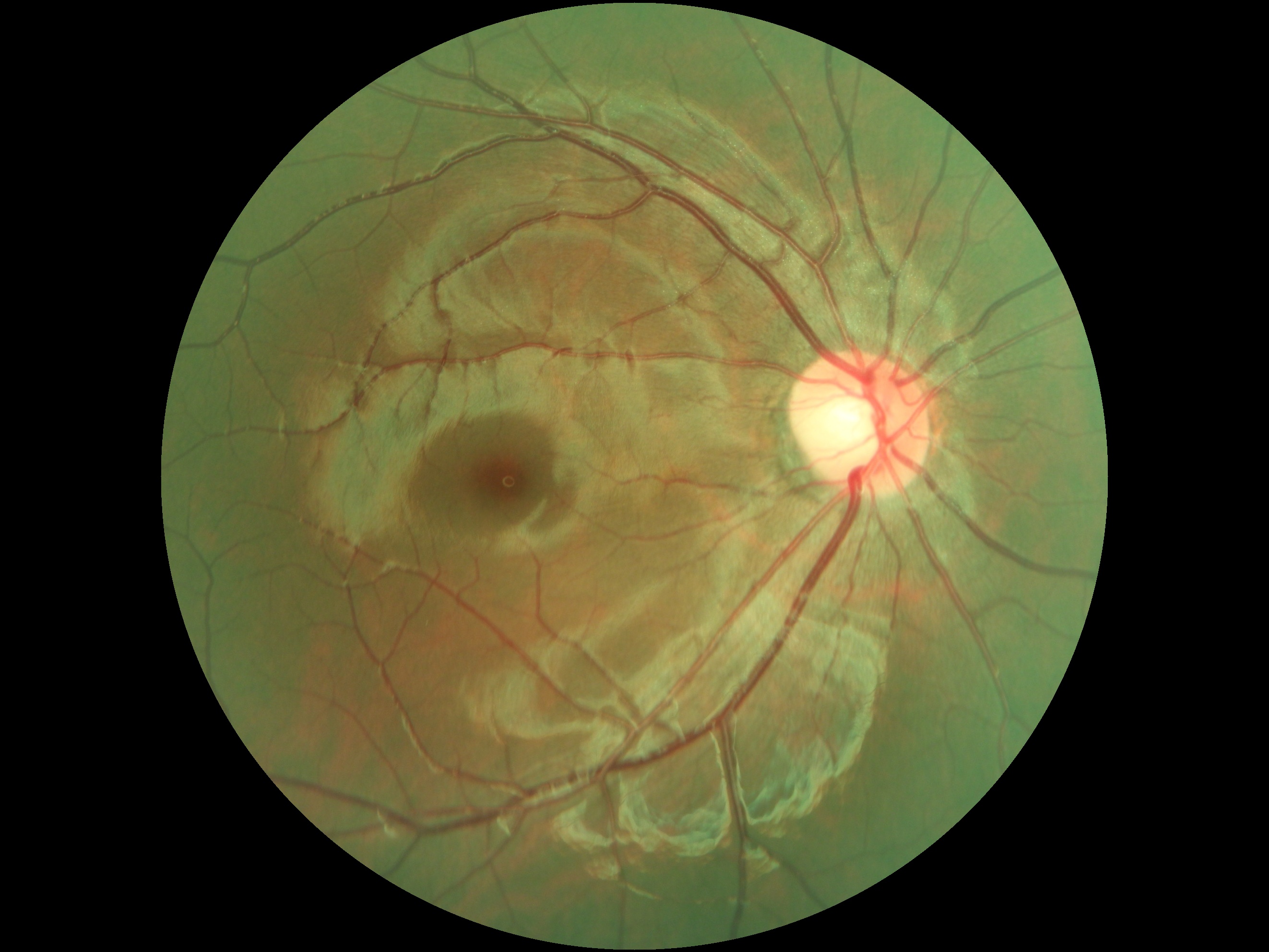

Supplement: S3 File — (ZIP) [file pone.0324352.s003.zip › Original fundus photographs (1)/Subject 6/OD_20230611698061_20230612152506_1.jpg]

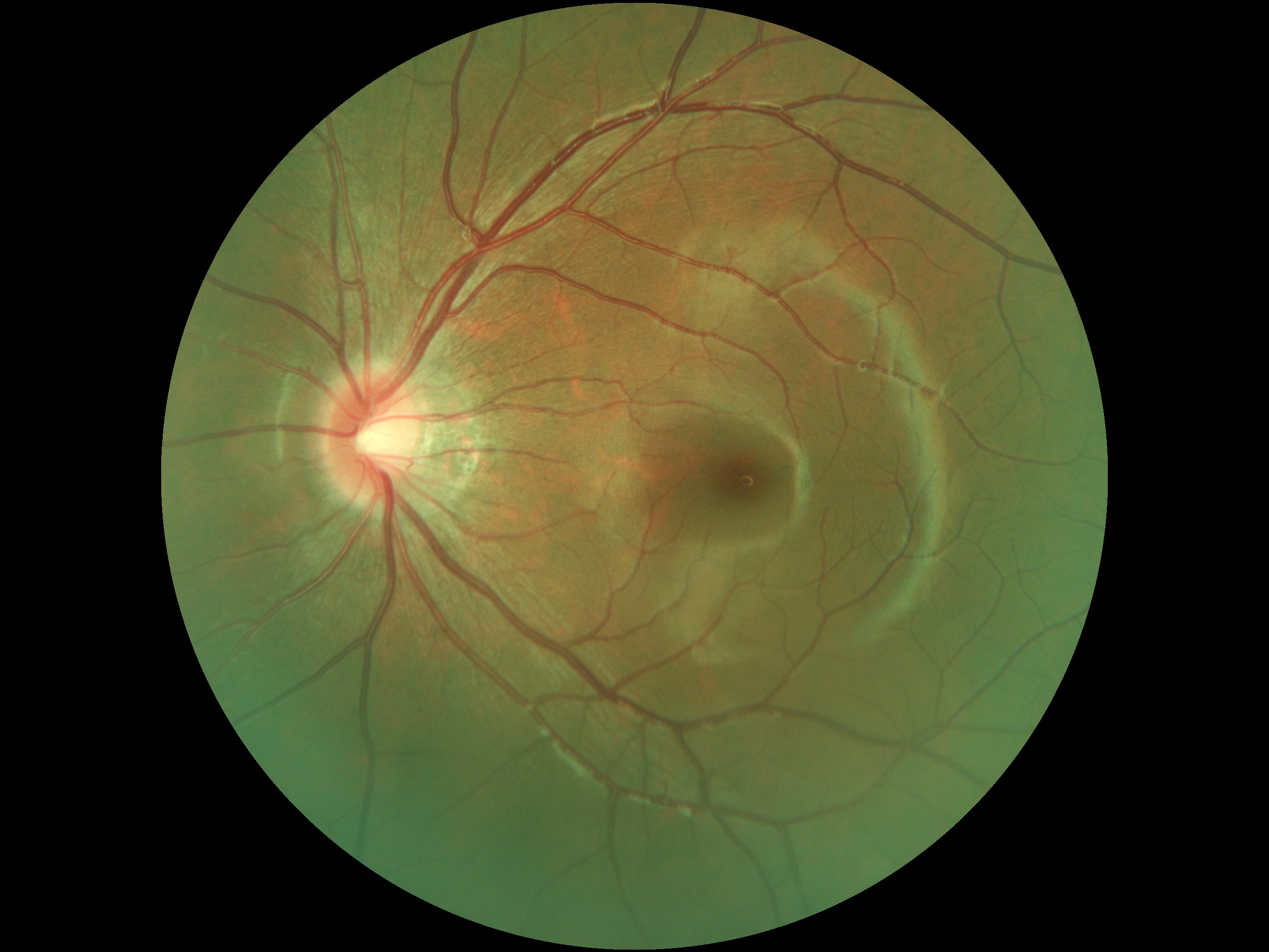

Supplement: S3 File — (ZIP) [file pone.0324352.s003.zip › Original fundus photographs (1)/Subject 6/OS_20230611698061_20230612152555_2.jpg]

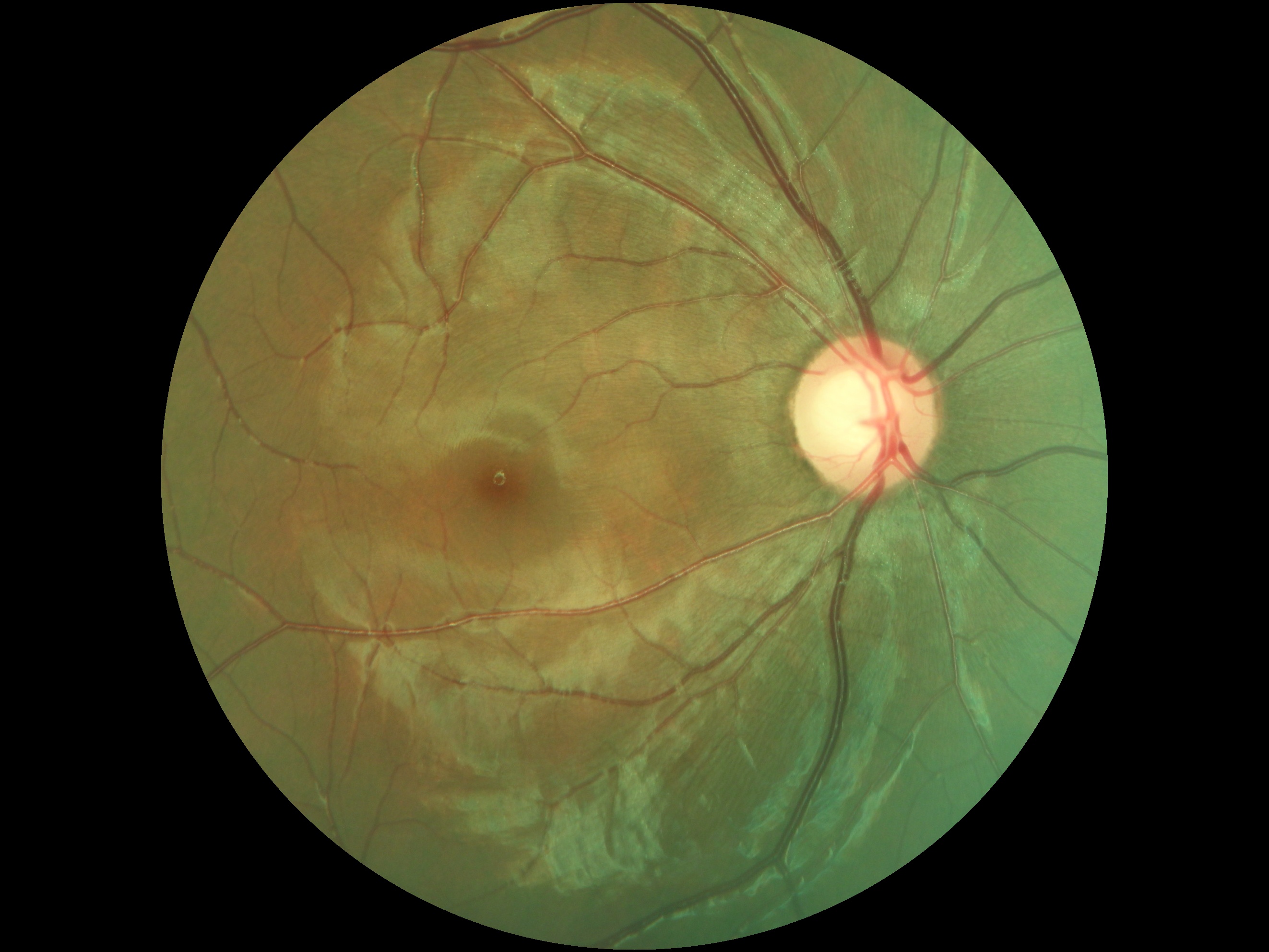

Supplement: S3 File — (ZIP) [file pone.0324352.s003.zip › Original fundus photographs (1)/Subject 60/OD_20230611232091_20230612160345_1.jpg]

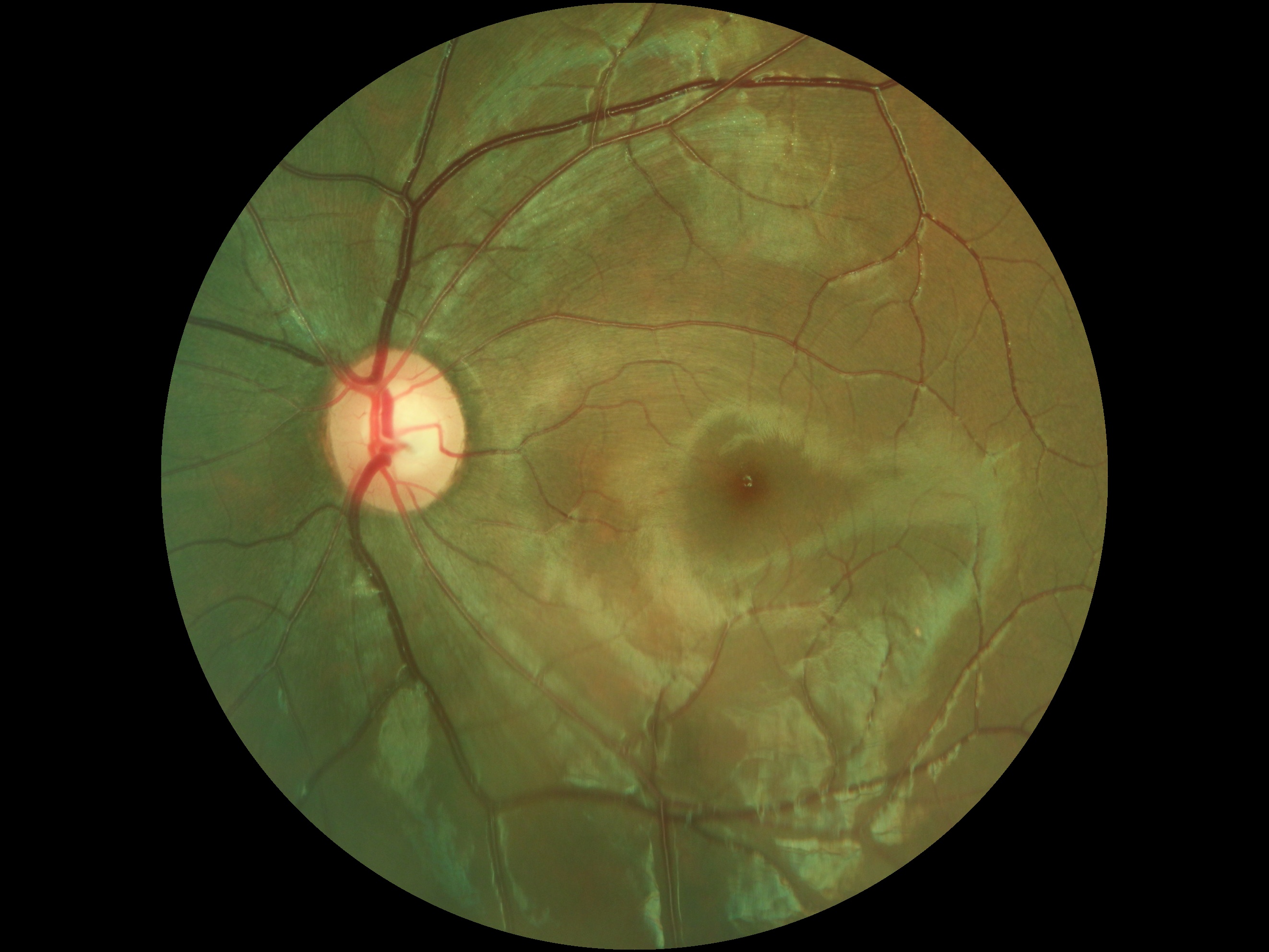

Supplement: S3 File — (ZIP) [file pone.0324352.s003.zip › Original fundus photographs (1)/Subject 60/OS_20230611232091_20230612160408_2.jpg]

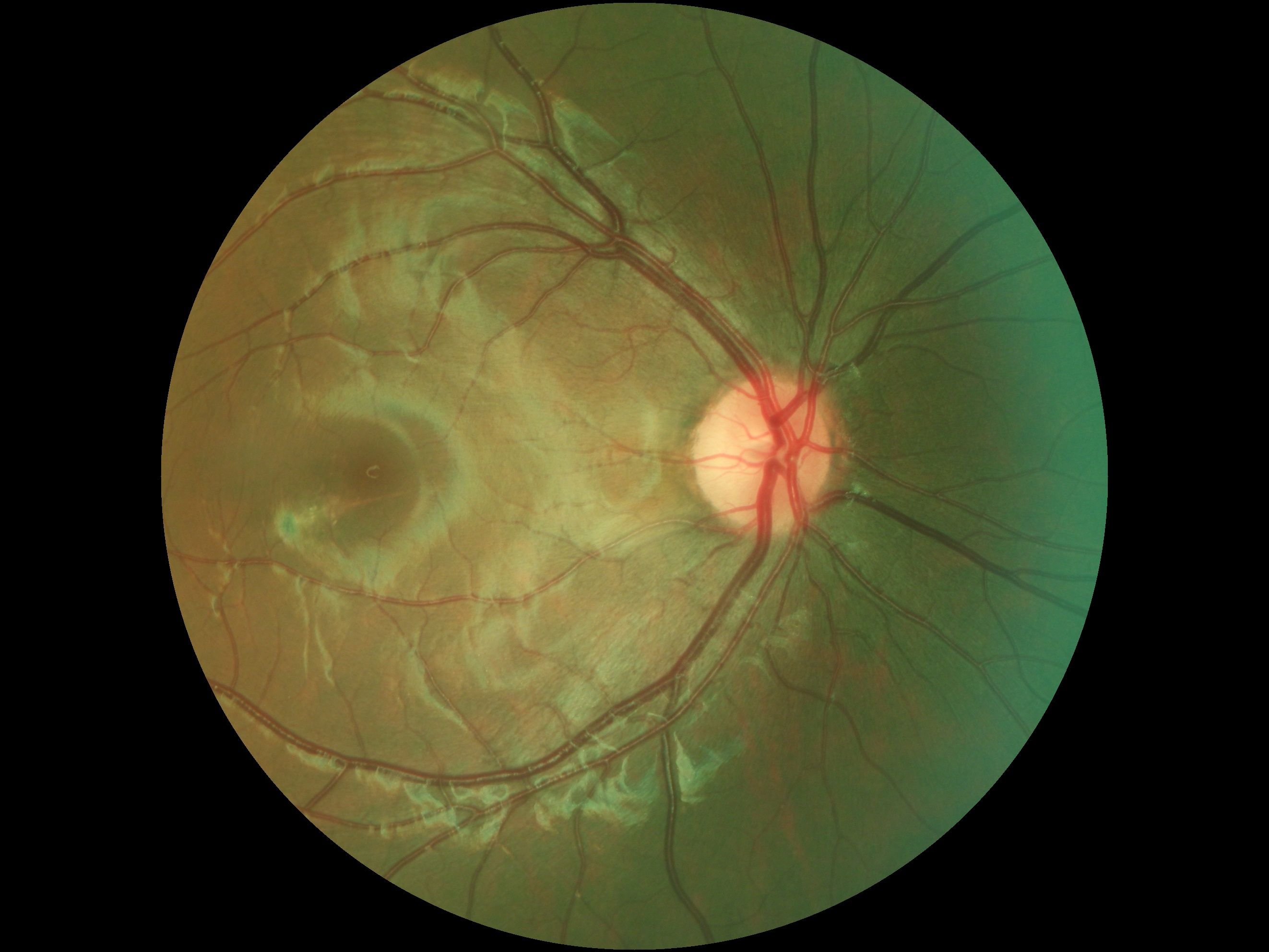

Supplement: S3 File — (ZIP) [file pone.0324352.s003.zip › Original fundus photographs (1)/Subject 61/OD_20230612201007_20230614160921_1.jpg]

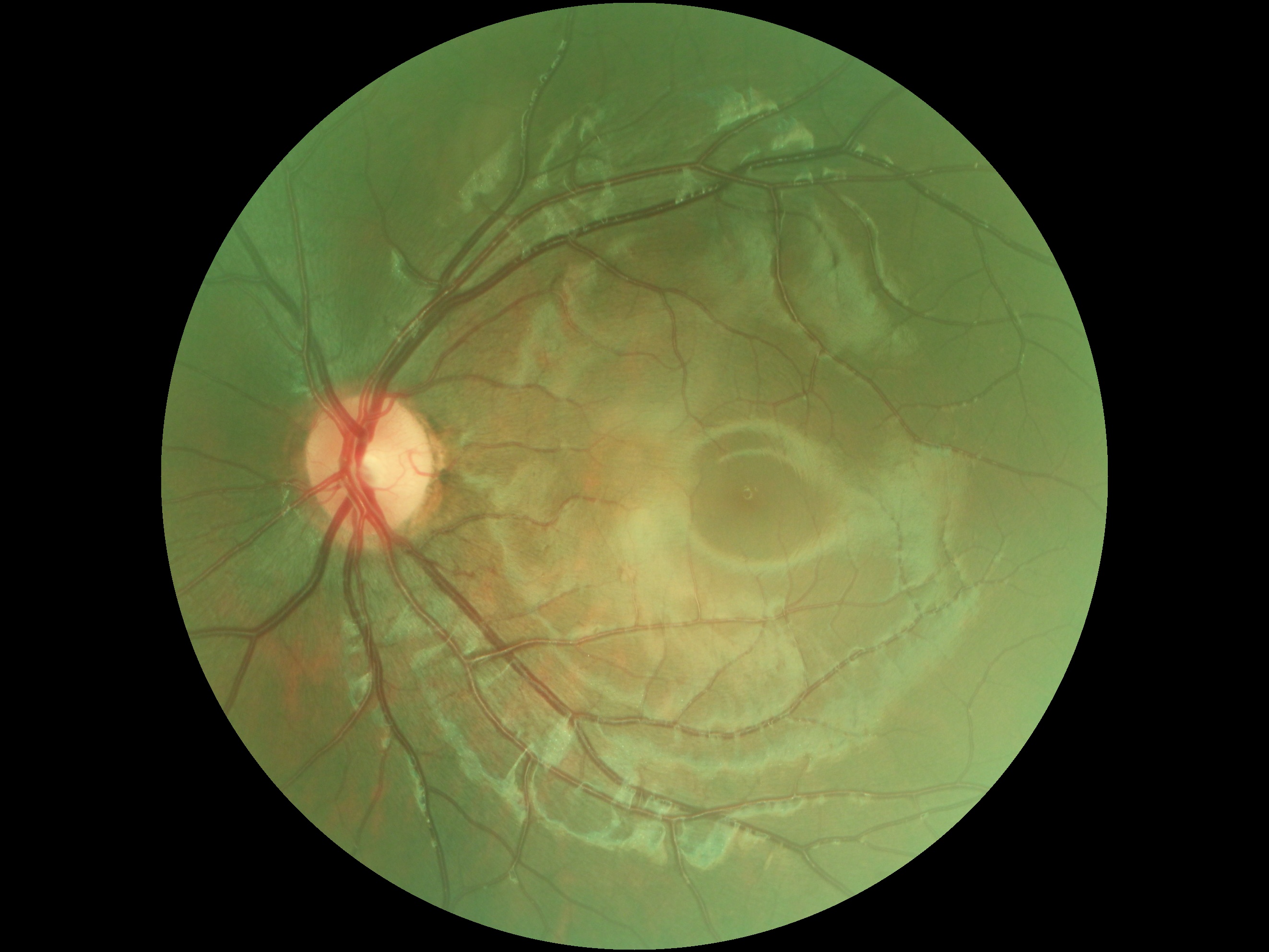

Supplement: S3 File — (ZIP) [file pone.0324352.s003.zip › Original fundus photographs (1)/Subject 61/OS_20230612201007_20230614160939_2.jpg]

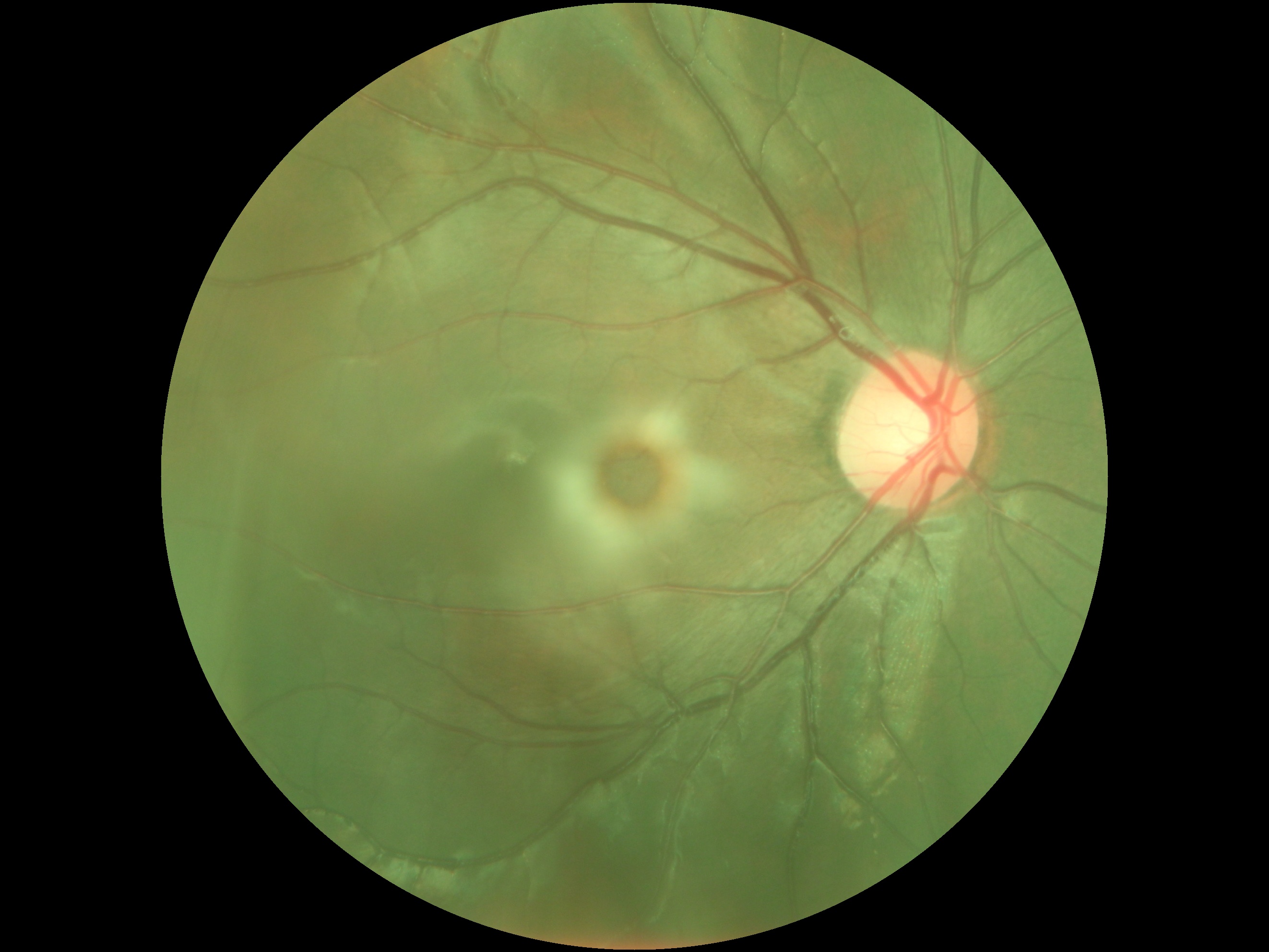

Supplement: S3 File — (ZIP) [file pone.0324352.s003.zip › Original fundus photographs (1)/Subject 62/OD_20230611754177_20230613170219_1.jpg]

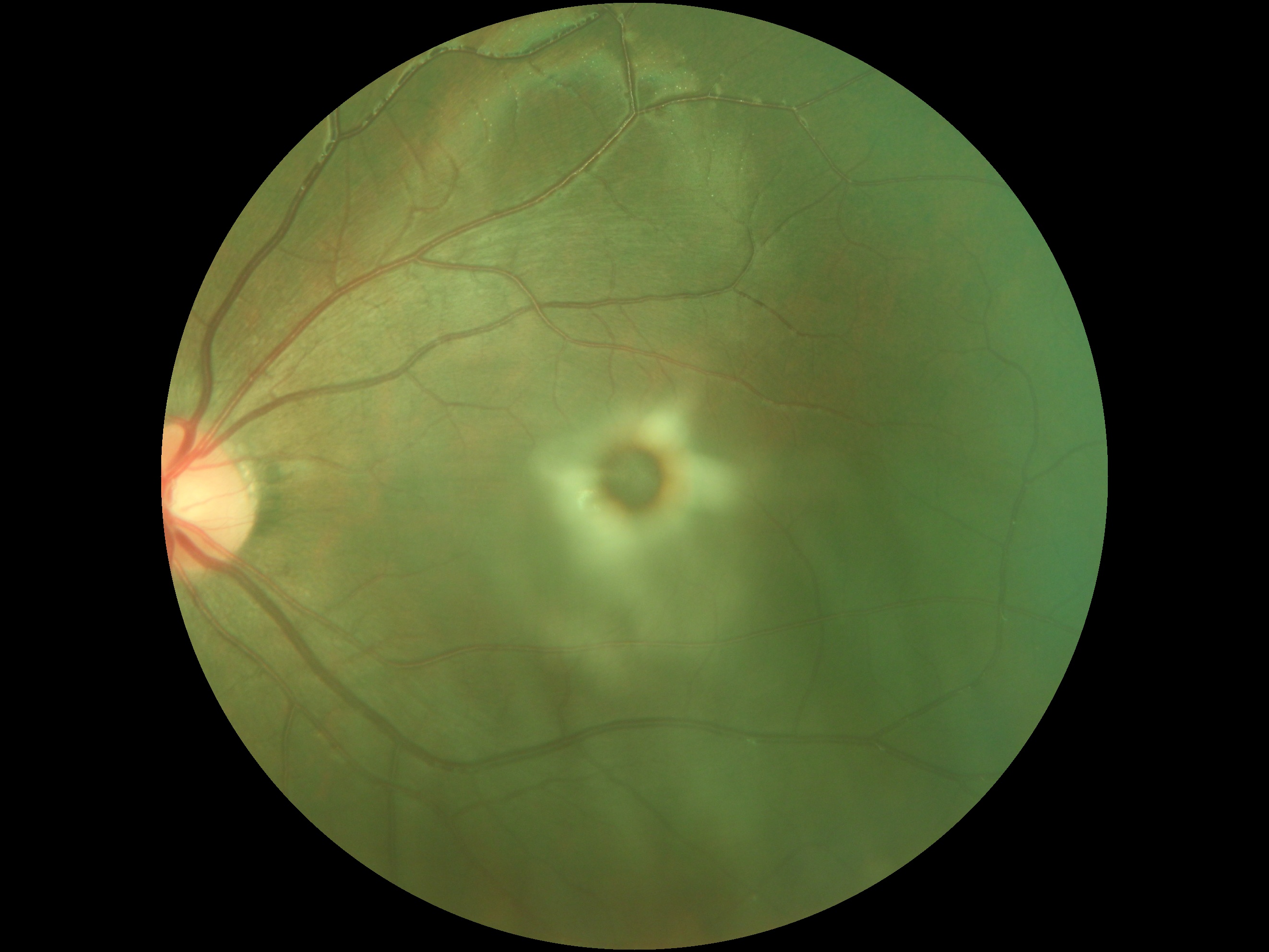

Supplement: S3 File — (ZIP) [file pone.0324352.s003.zip › Original fundus photographs (1)/Subject 62/OS_20230611754177_20230613170333_4.jpg]

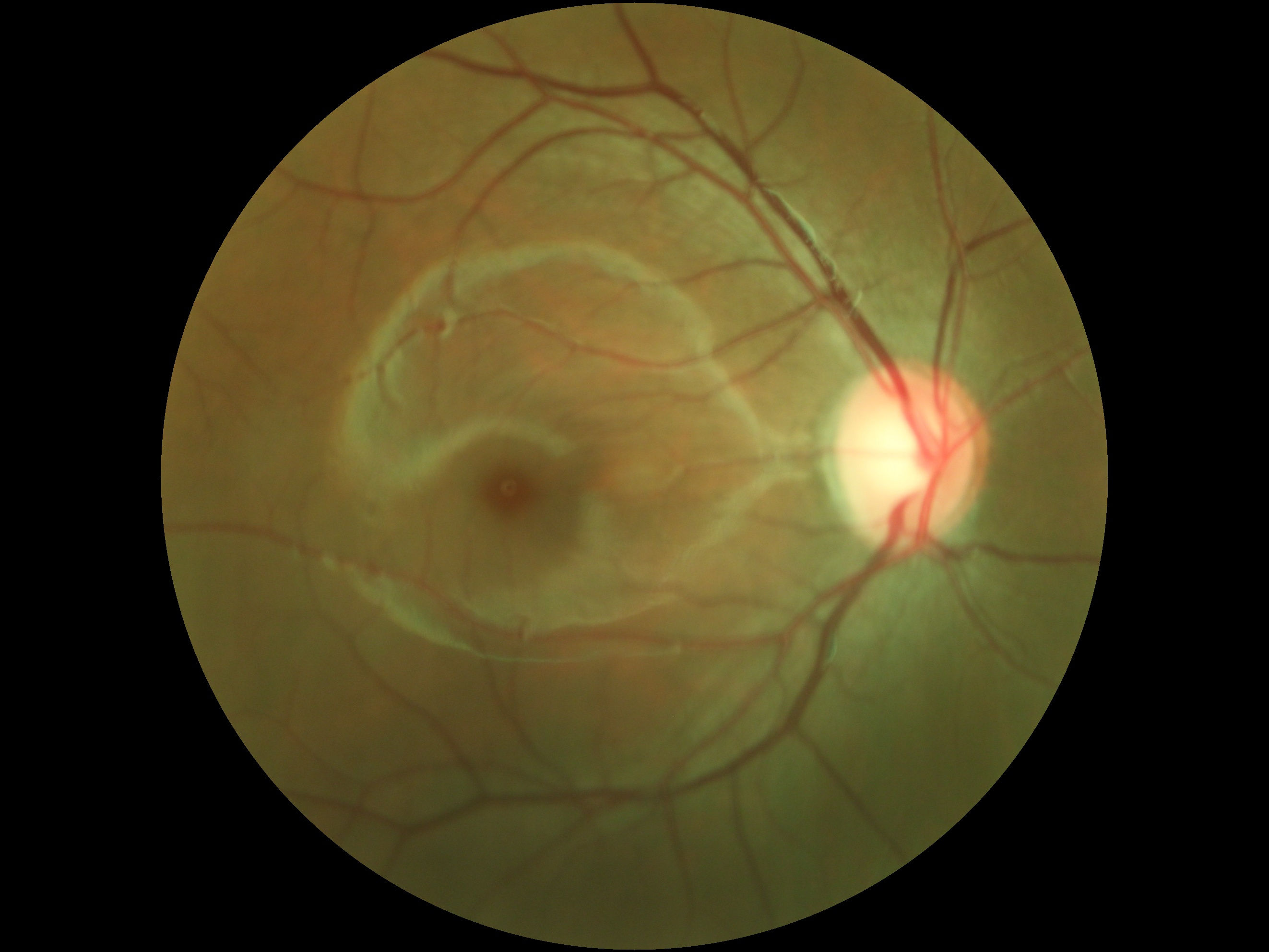

Supplement: S3 File — (ZIP) [file pone.0324352.s003.zip › Original fundus photographs (1)/Subject 7/OD_20230611817007_20230612111215_1.jpg]

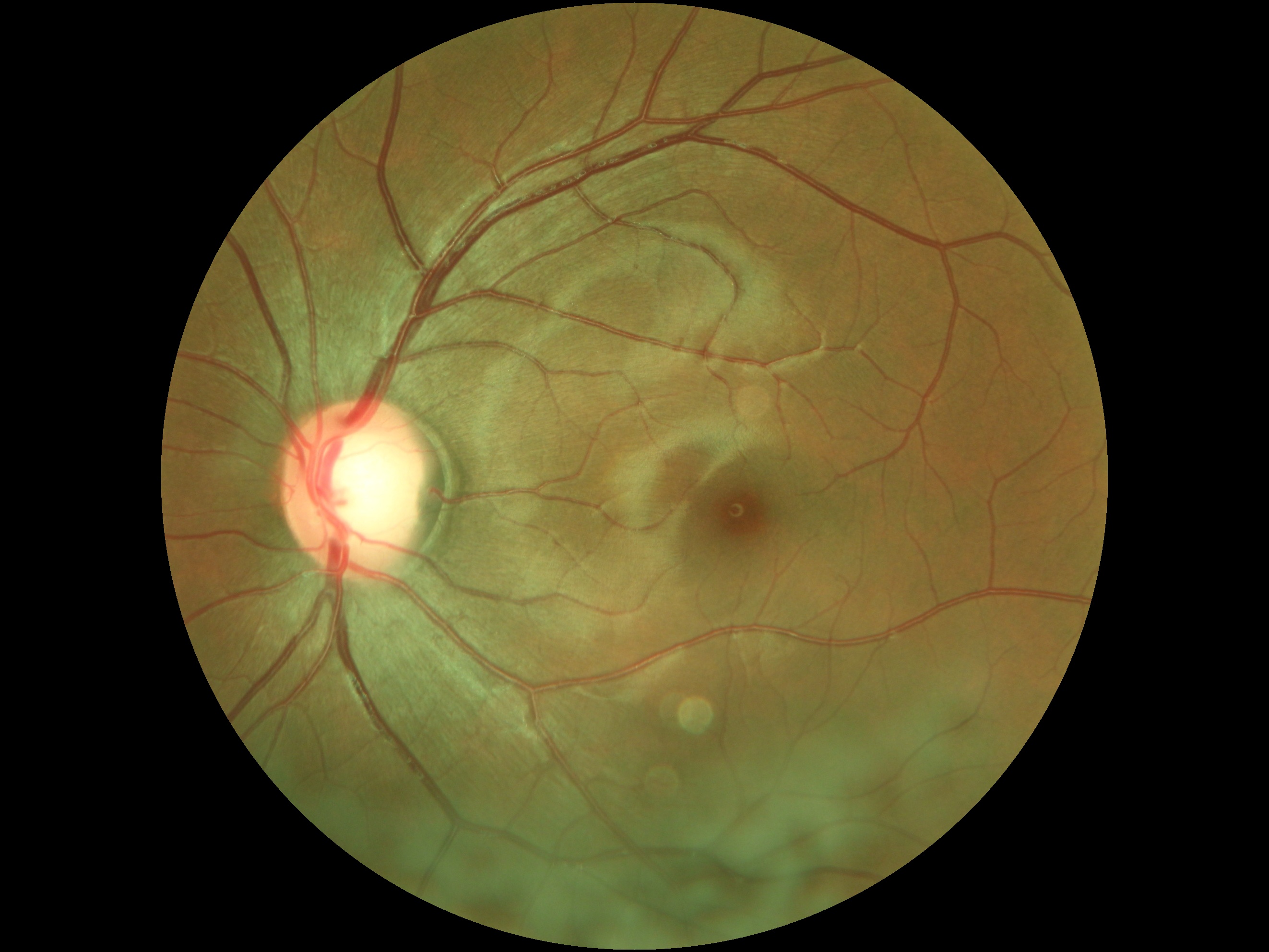

Supplement: S3 File — (ZIP) [file pone.0324352.s003.zip › Original fundus photographs (1)/Subject 7/OS_20230611817007_20230612111245_2.jpg]

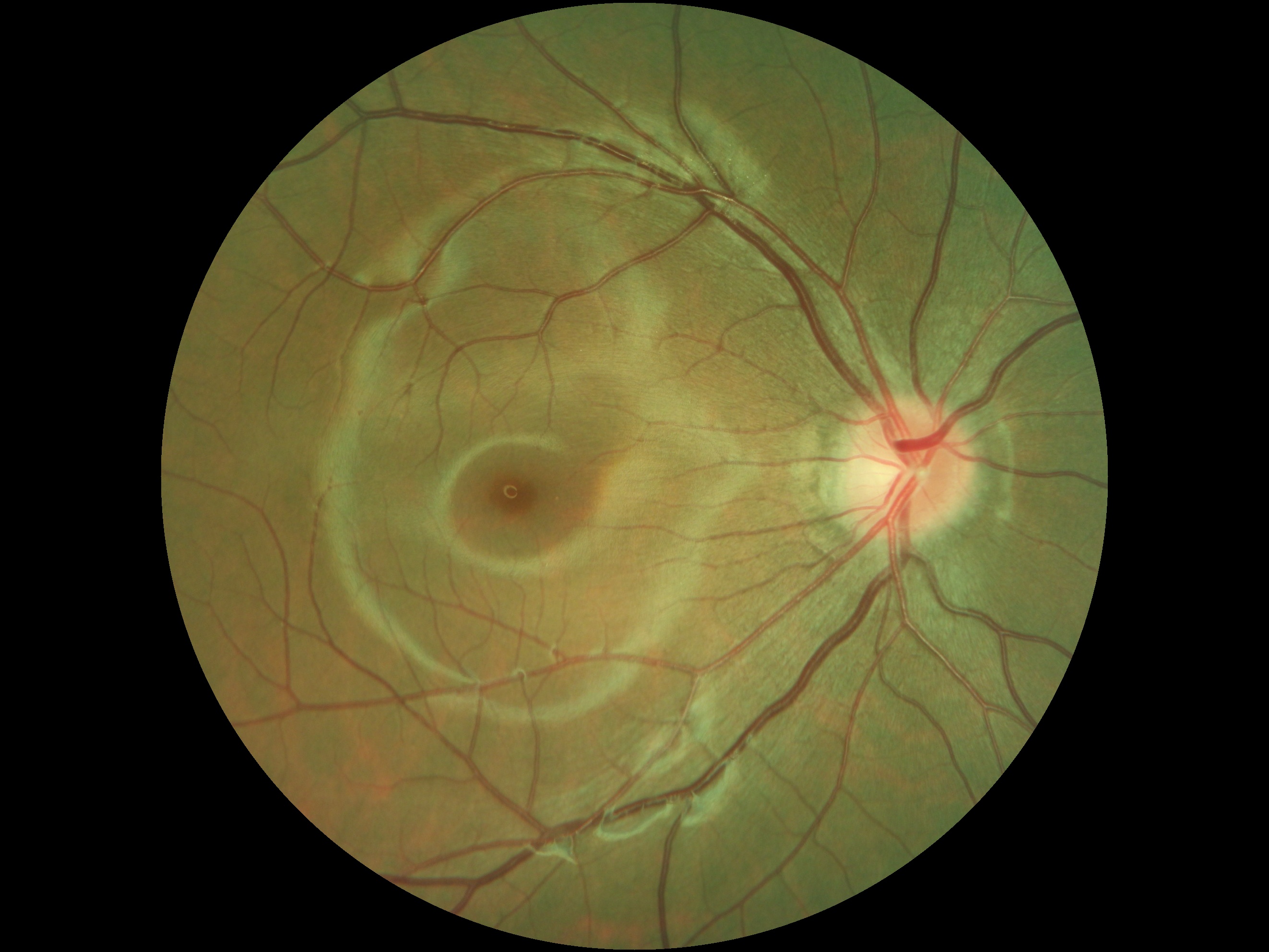

Supplement: S3 File — (ZIP) [file pone.0324352.s003.zip › Original fundus photographs (1)/Subject 8/OD_20230611972029_20230612161147_1.jpg]

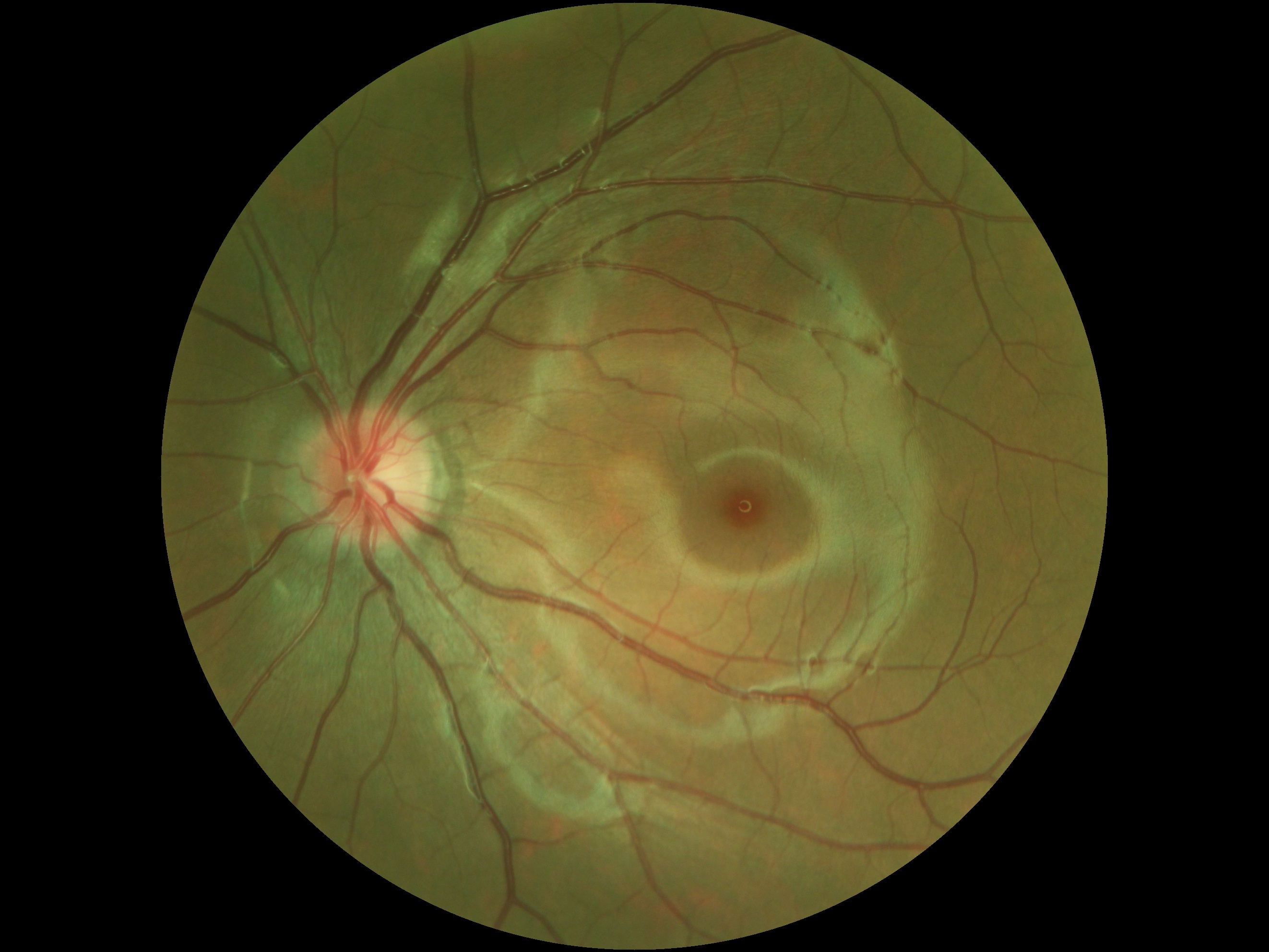

Supplement: S3 File — (ZIP) [file pone.0324352.s003.zip › Original fundus photographs (1)/Subject 8/OS_20230611972029_20230612161219_2.jpg]

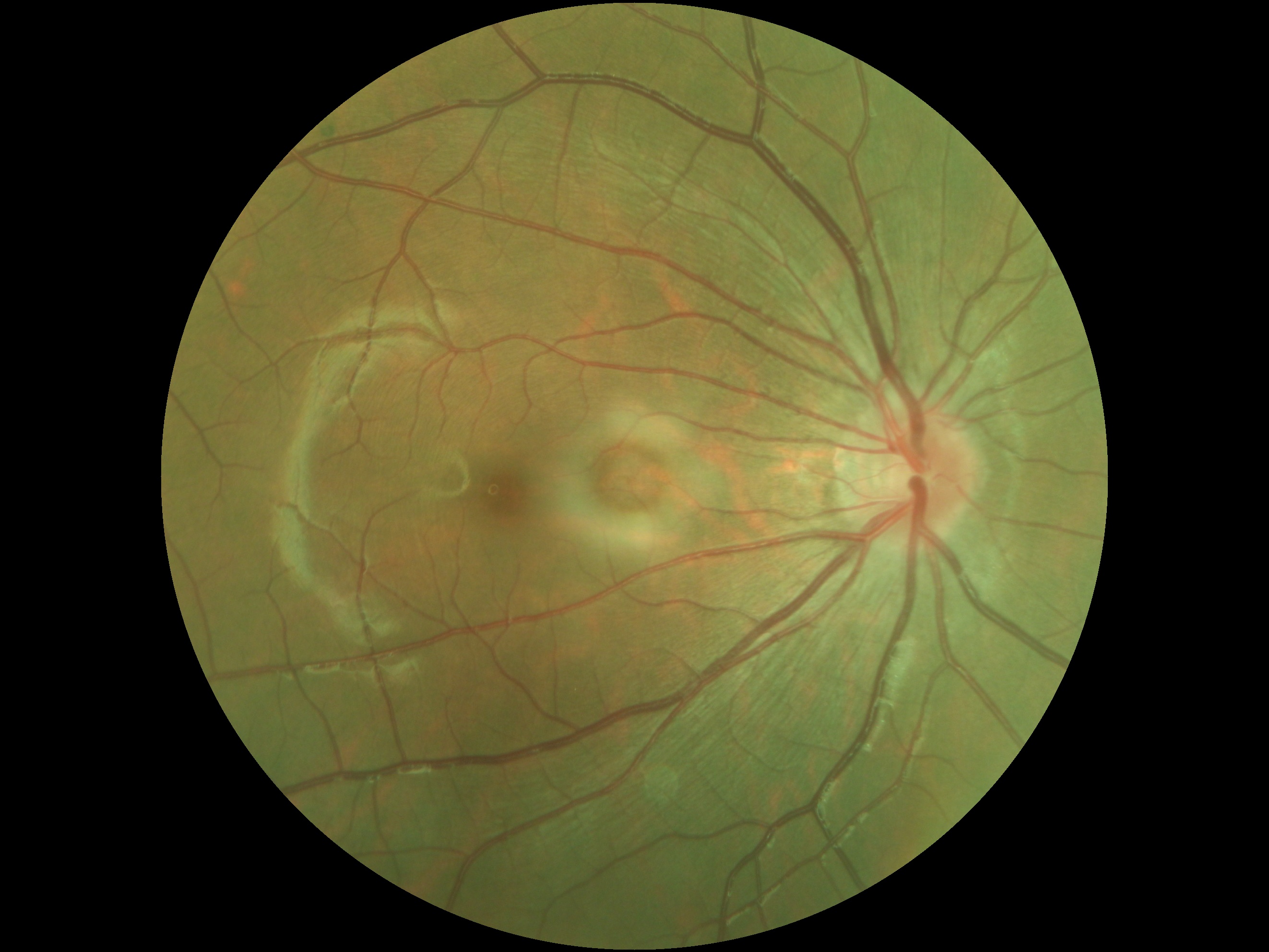

Supplement: S3 File — (ZIP) [file pone.0324352.s003.zip › Original fundus photographs (1)/Subject 9/OD_20230611444139_20230614100220_1.jpg]

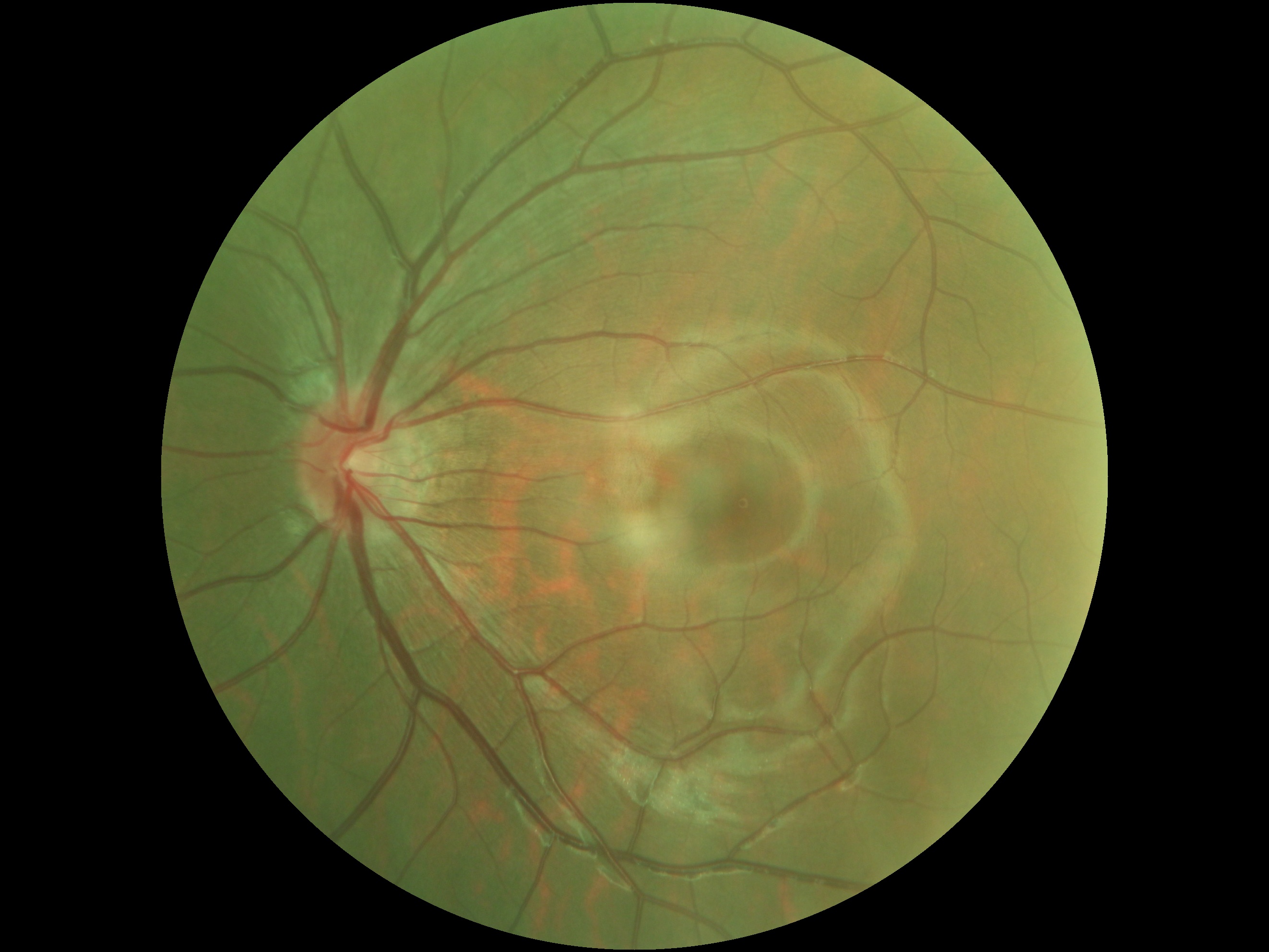

Supplement: S3 File — (ZIP) [file pone.0324352.s003.zip › Original fundus photographs (1)/Subject 9/OS_20230611444139_20230614100340_2.jpg]

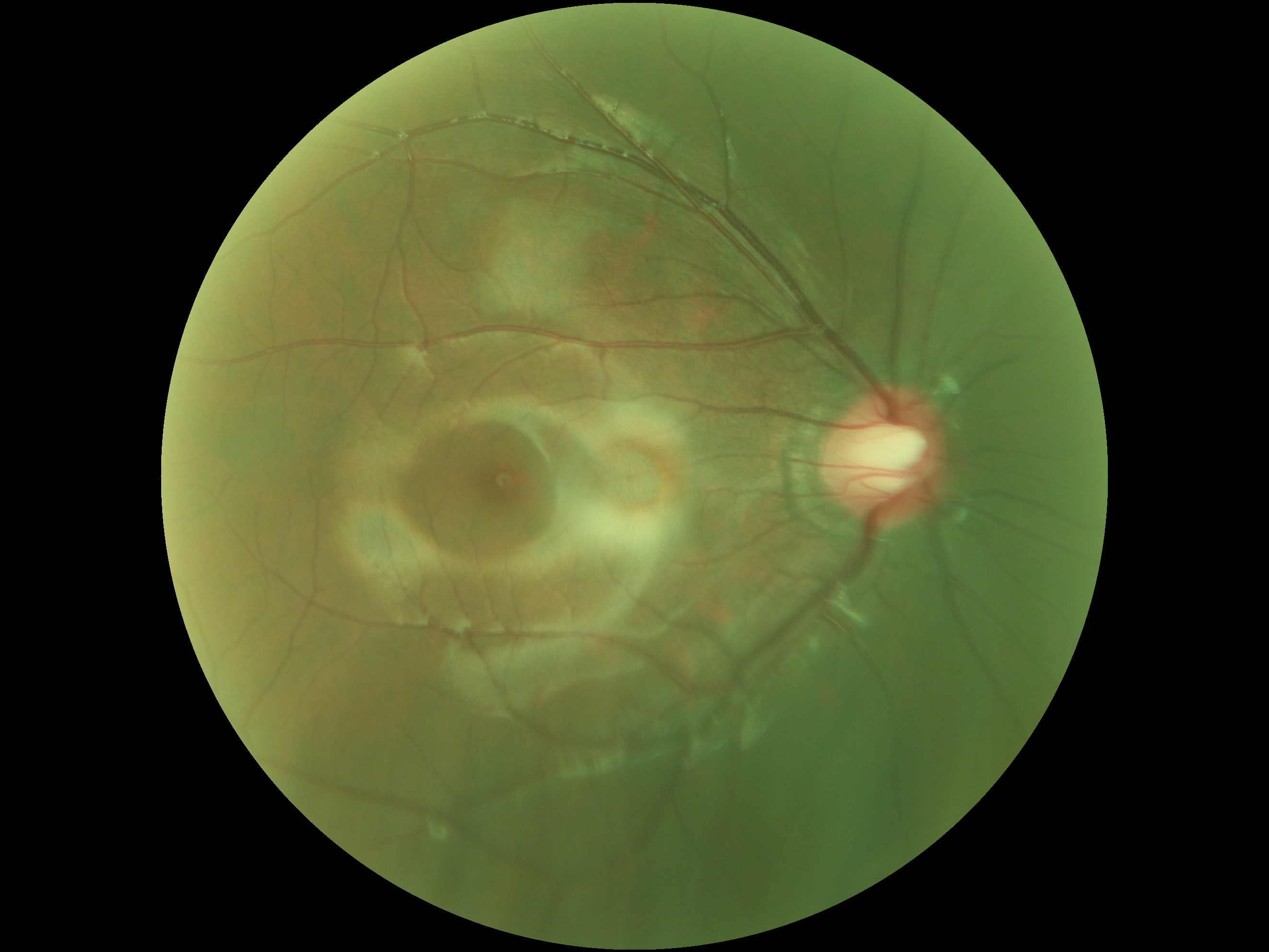

Supplement: S4 File — (ZIP) [file pone.0324352.s004.zip › Original fundus photographs (2)/Subject 100/OD_20230611896268_20230615104208_2.jpg]

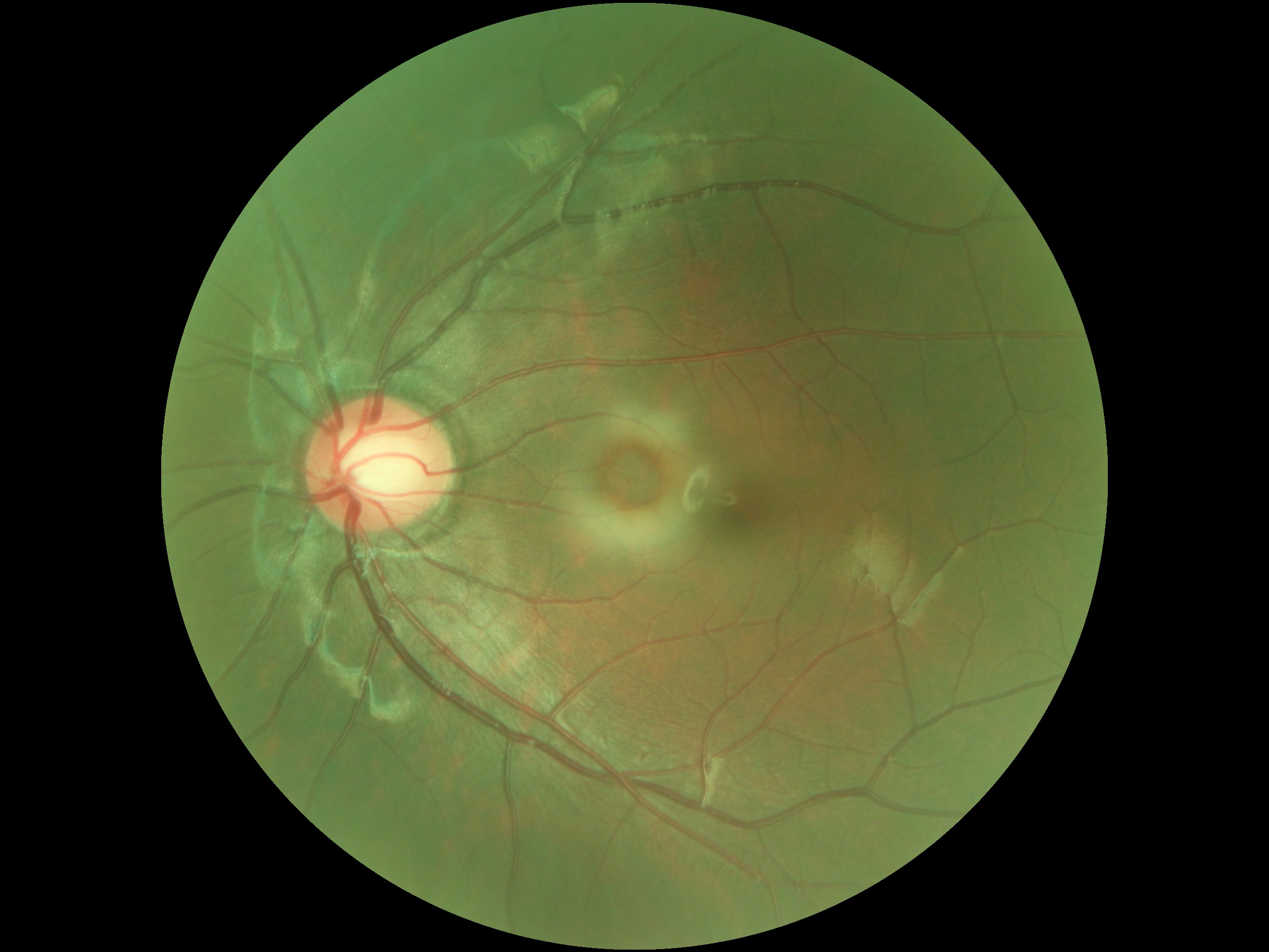

Supplement: S4 File — (ZIP) [file pone.0324352.s004.zip › Original fundus photographs (2)/Subject 100/OS_20230611896268_20230615104147_1.jpg]

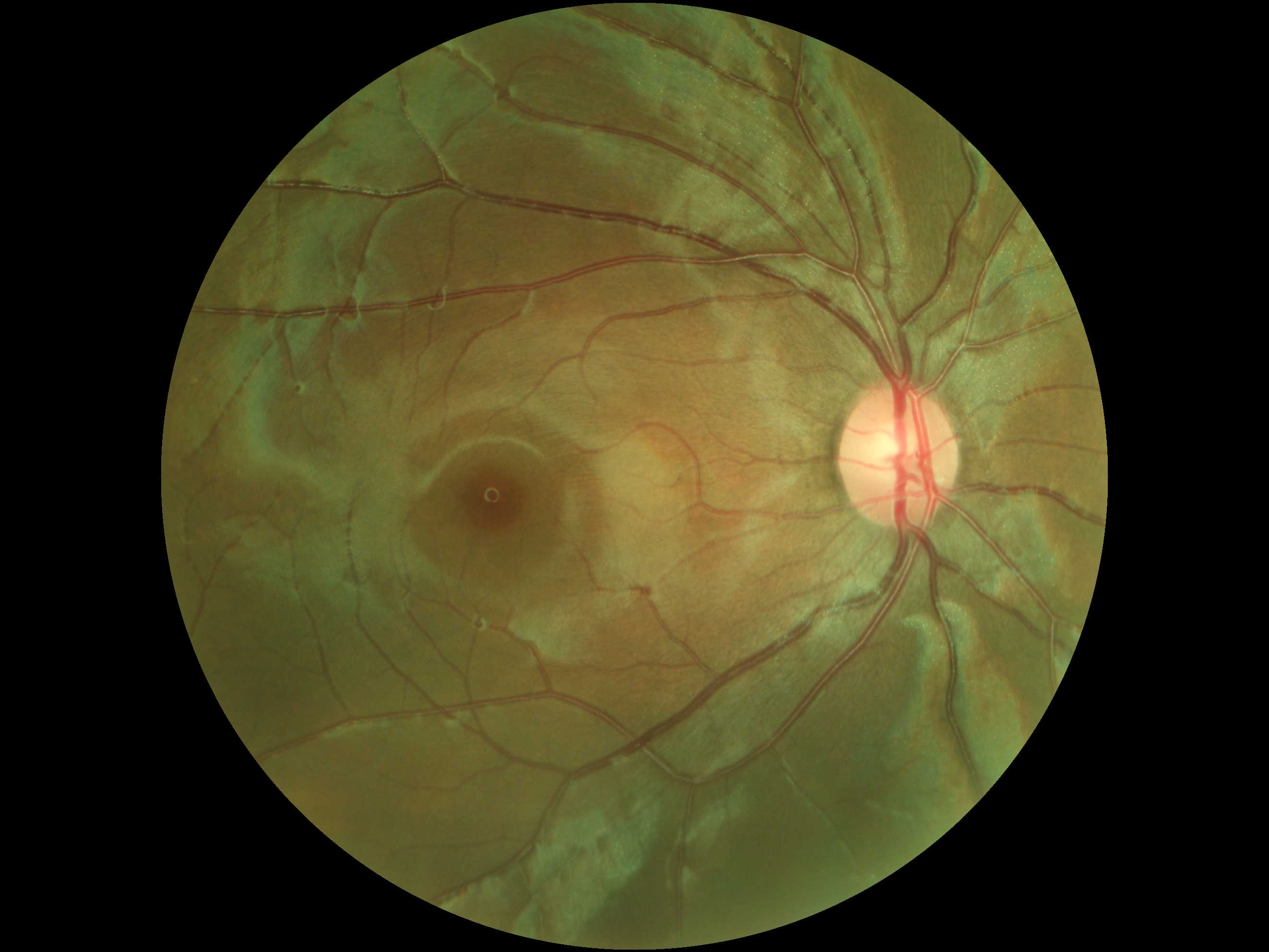

Supplement: S4 File — (ZIP) [file pone.0324352.s004.zip › Original fundus photographs (2)/Subject 101/OD_20230611435048_20230613105746_2.jpg]

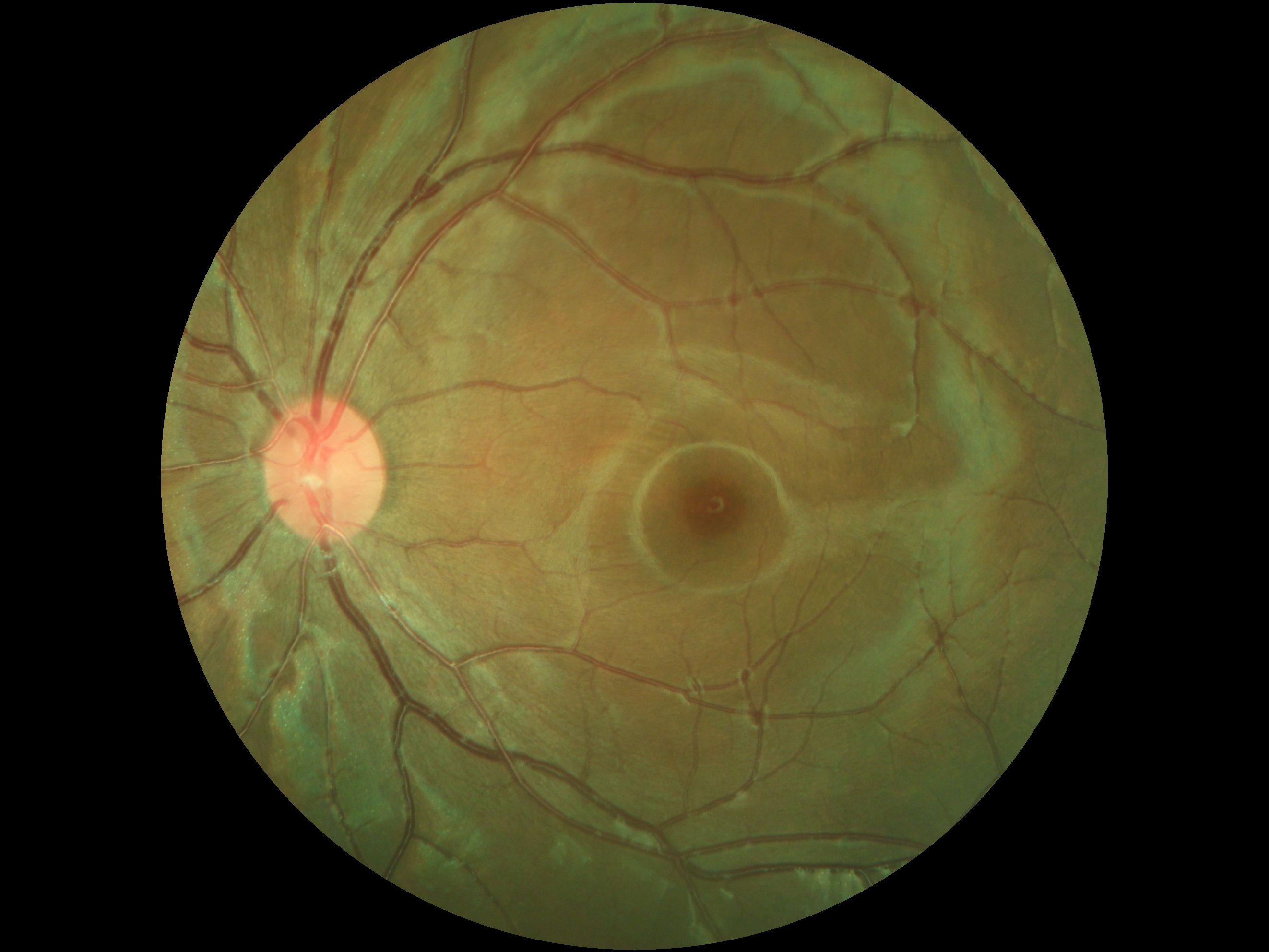

Supplement: S4 File — (ZIP) [file pone.0324352.s004.zip › Original fundus photographs (2)/Subject 101/OS_20230611435048_20230613105830_3.jpg]

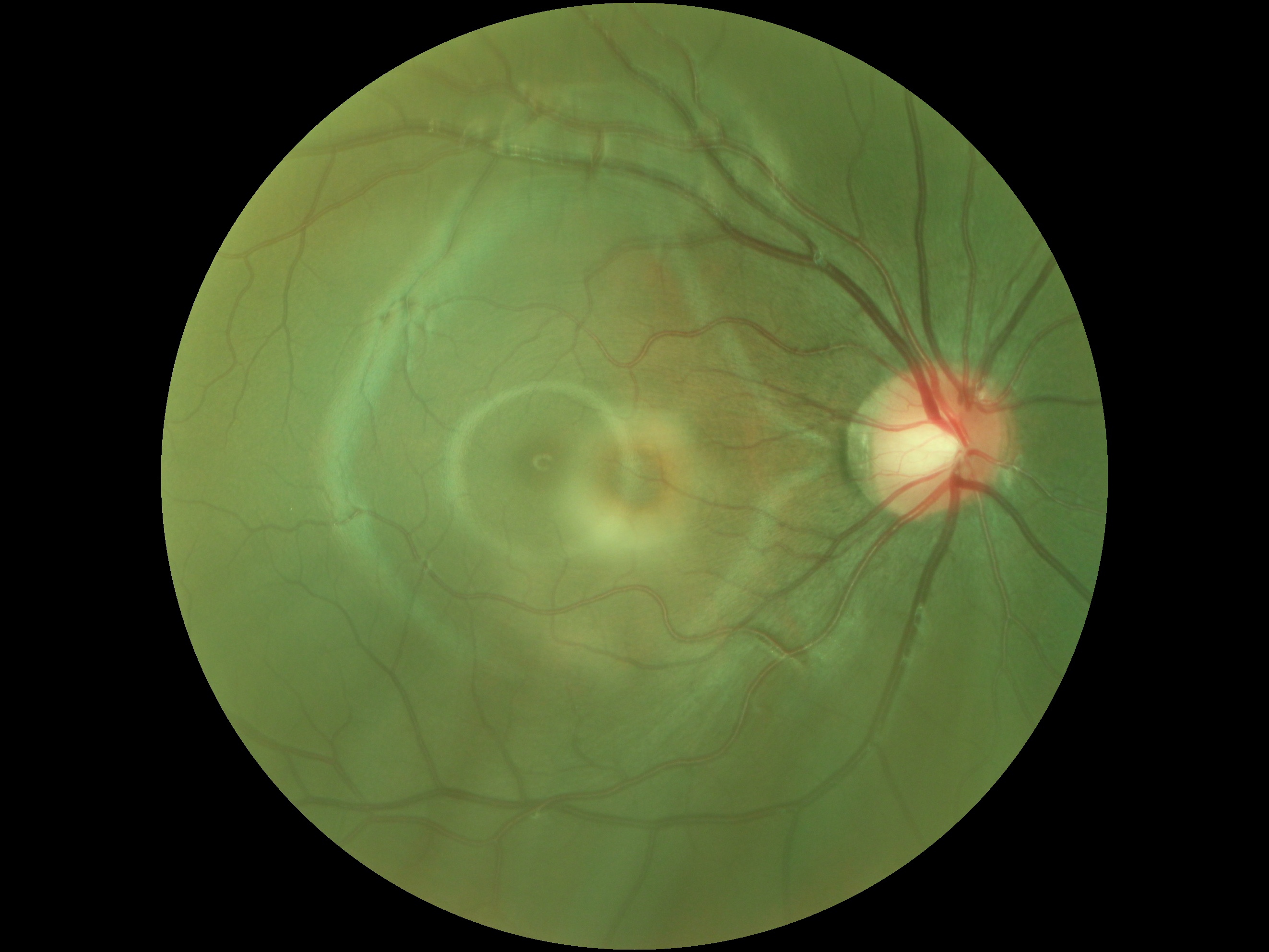

Supplement: S4 File — (ZIP) [file pone.0324352.s004.zip › Original fundus photographs (2)/Subject 102/OD_20230615639065_20230615154145_4.jpg]

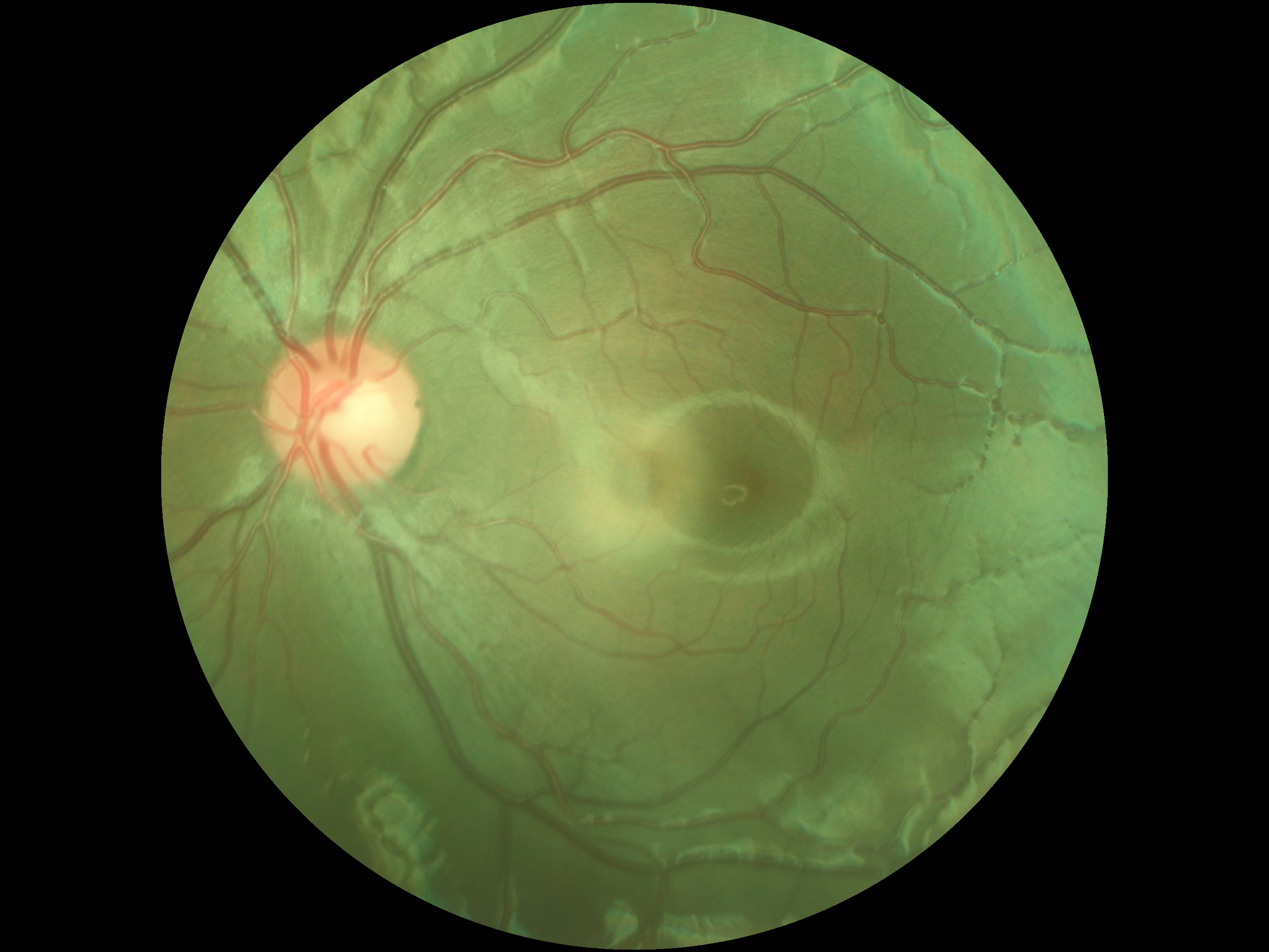

Supplement: S4 File — (ZIP) [file pone.0324352.s004.zip › Original fundus photographs (2)/Subject 102/OS_20230615639065_20230615154042_2.jpg]

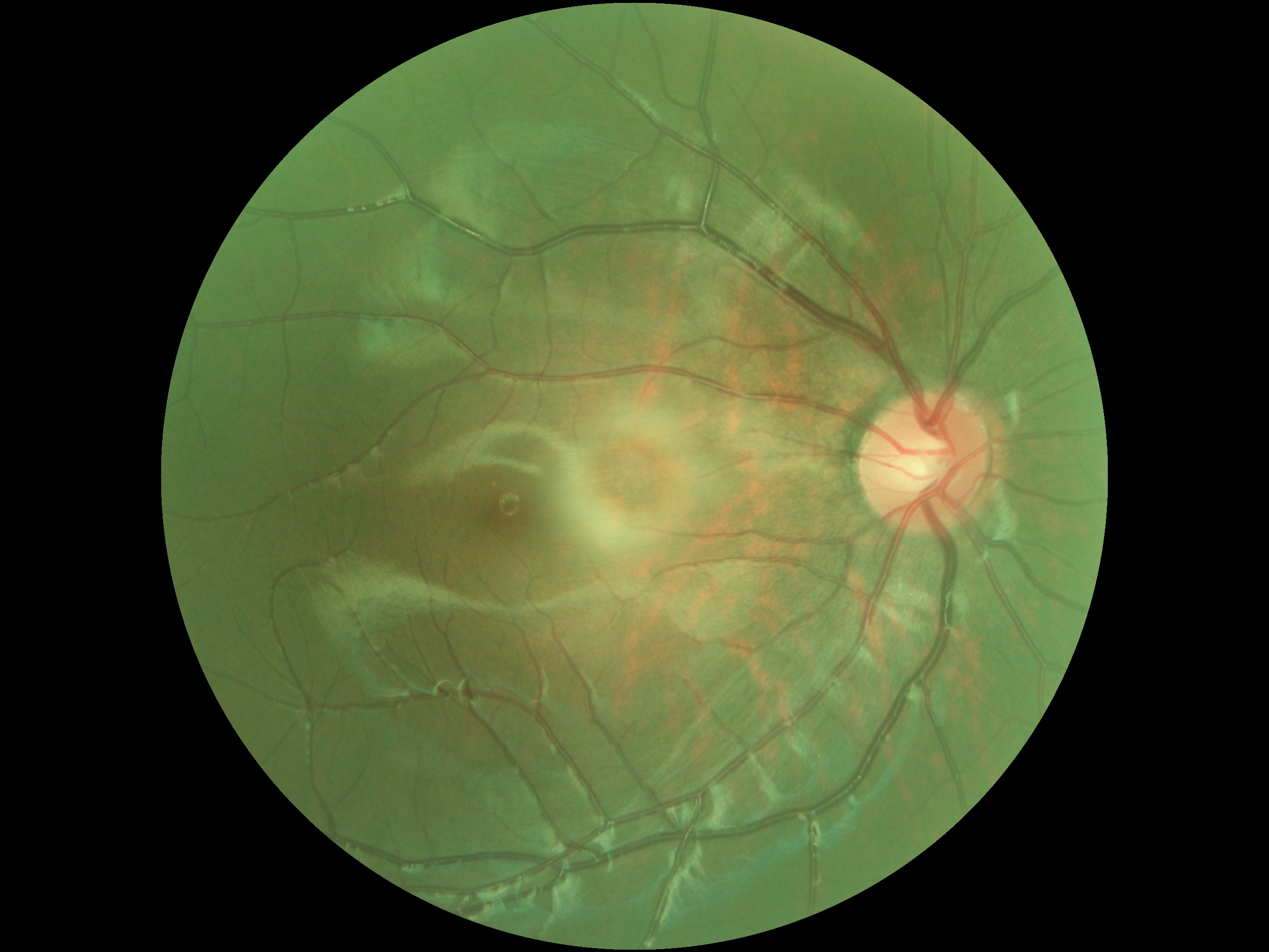

Supplement: S4 File — (ZIP) [file pone.0324352.s004.zip › Original fundus photographs (2)/Subject 103/OD_20230611524242_20230614113916_3.jpg]

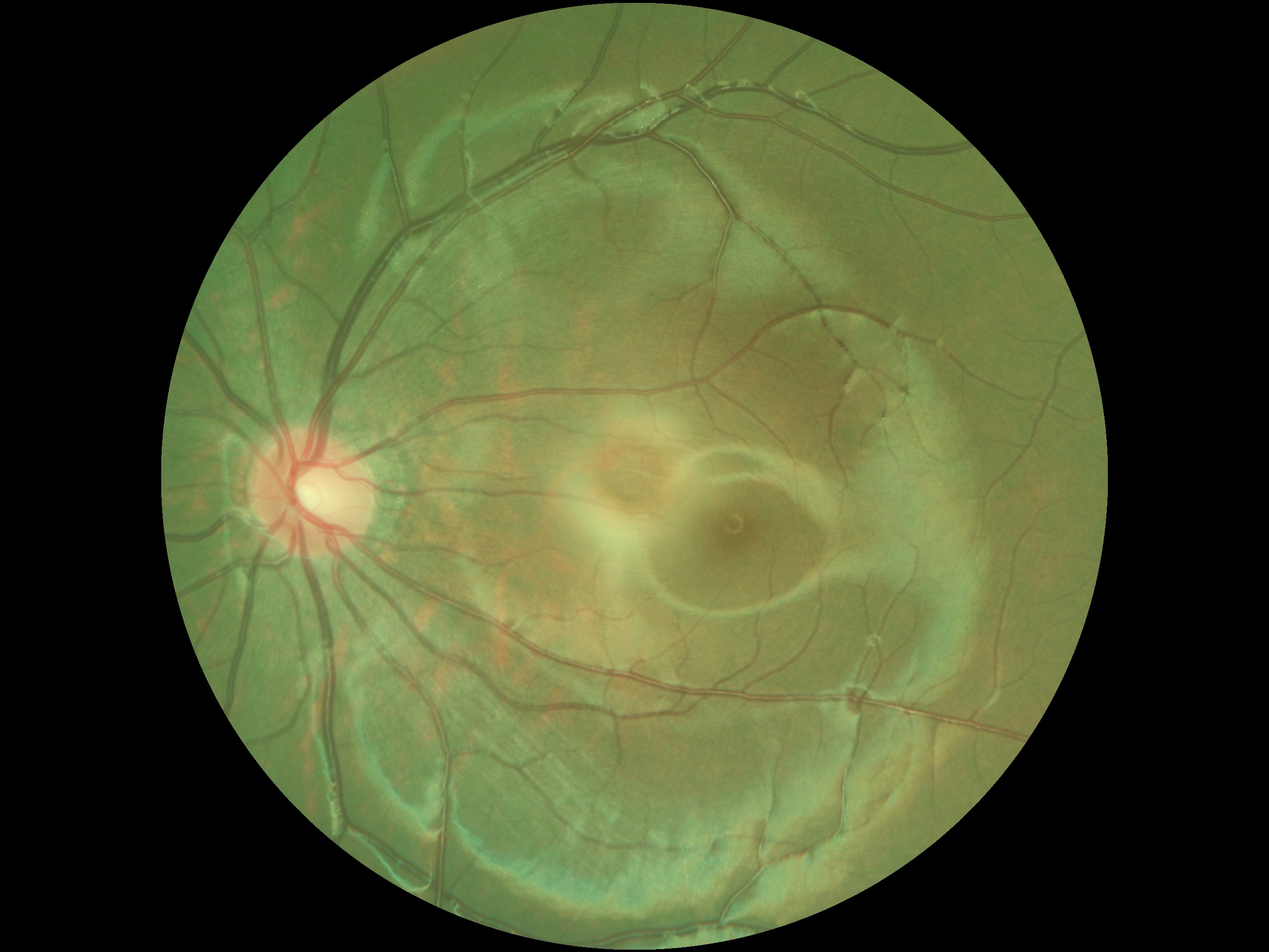

Supplement: S4 File — (ZIP) [file pone.0324352.s004.zip › Original fundus photographs (2)/Subject 103/OS_20230611524242_20230614113857_2.jpg]

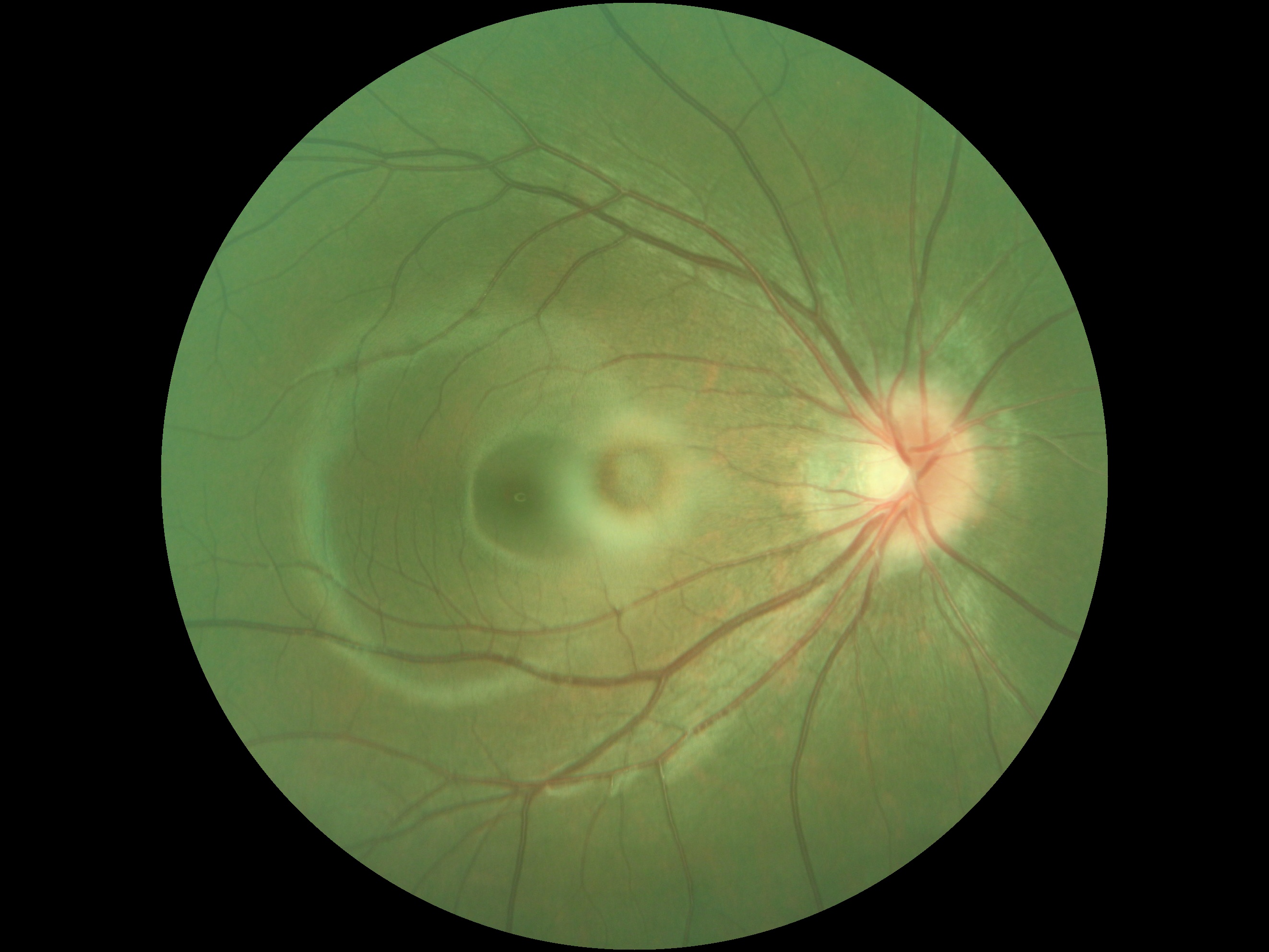

Supplement: S4 File — (ZIP) [file pone.0324352.s004.zip › Original fundus photographs (2)/Subject 104/OD_20230611440187_20230614102542_2.jpg]

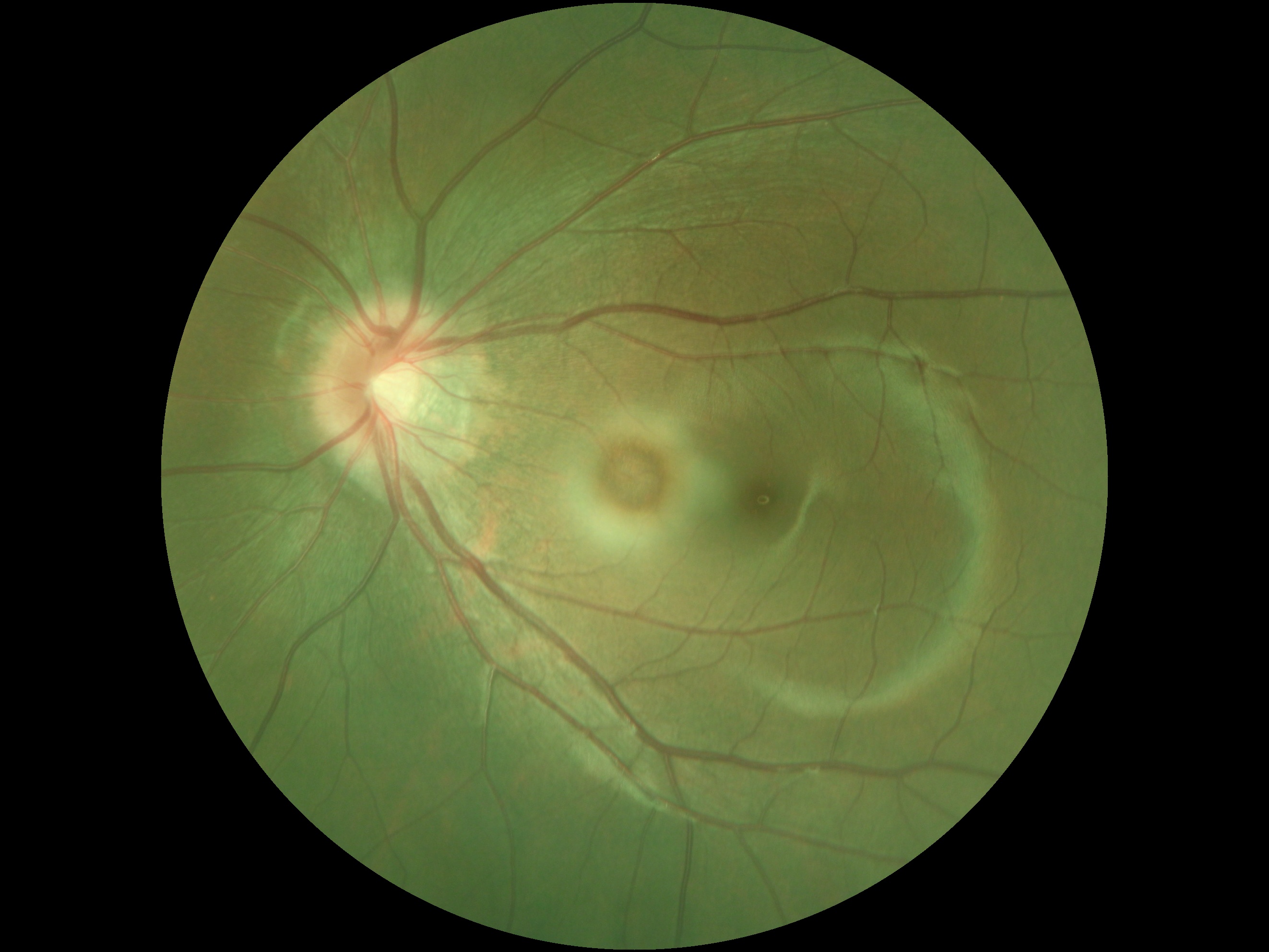

Supplement: S4 File — (ZIP) [file pone.0324352.s004.zip › Original fundus photographs (2)/Subject 104/OS_20230611440187_20230614102514_1.jpg]

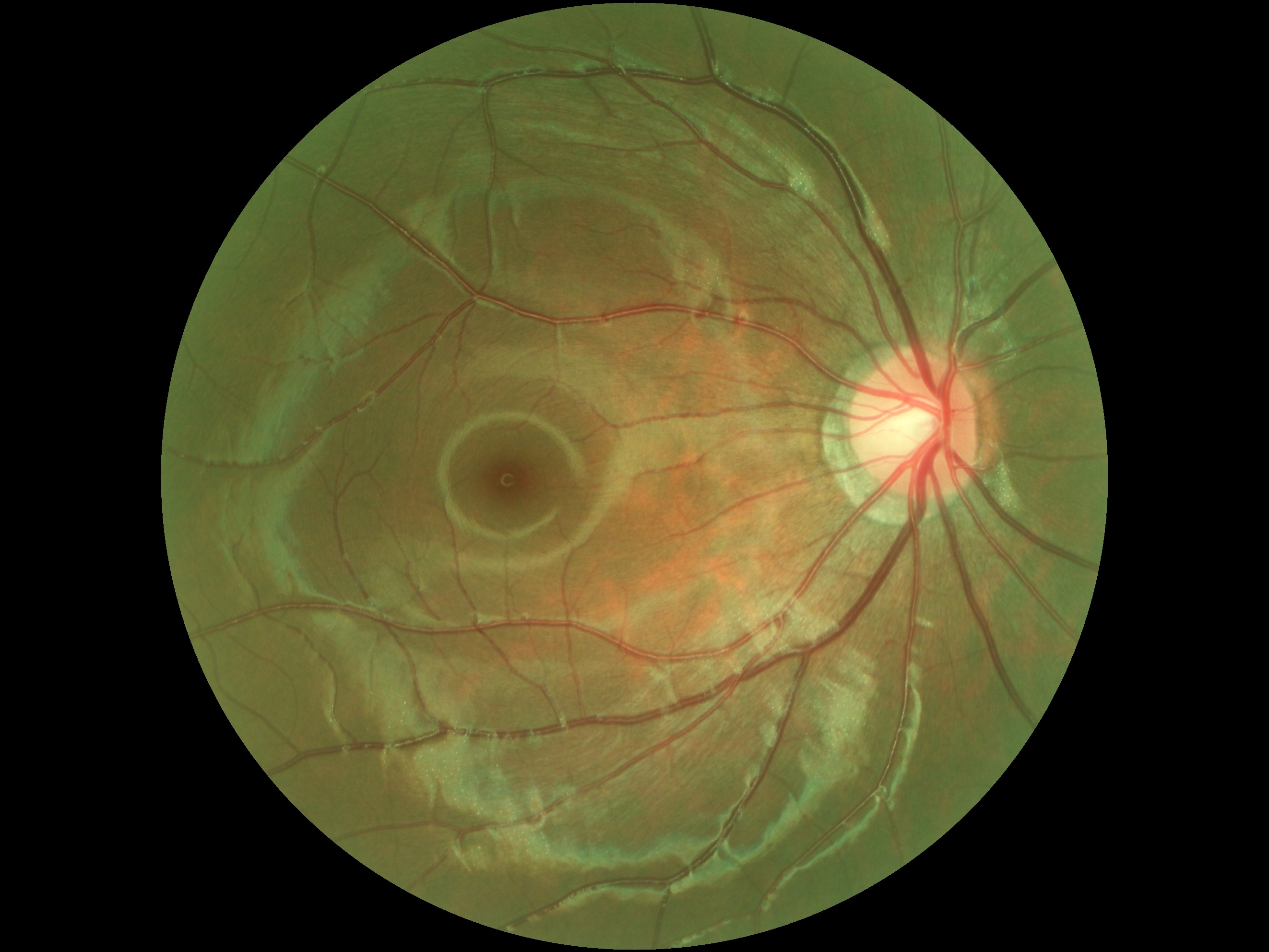

Supplement: S4 File — (ZIP) [file pone.0324352.s004.zip › Original fundus photographs (2)/Subject 105/OD_20230611213038_20230613103650_1.jpg]

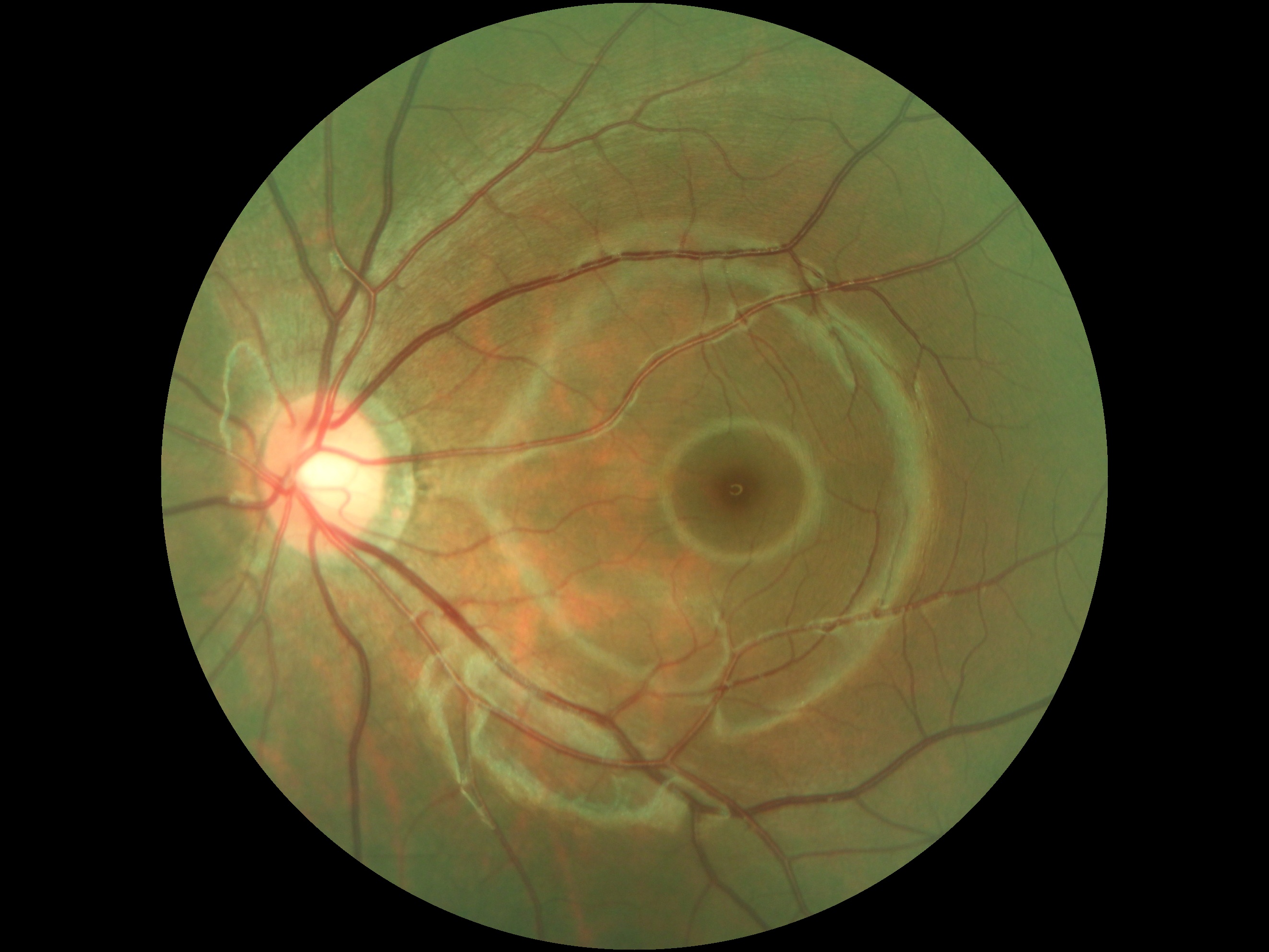

Supplement: S4 File — (ZIP) [file pone.0324352.s004.zip › Original fundus photographs (2)/Subject 105/OS_20230611213038_20230613103742_2.jpg]

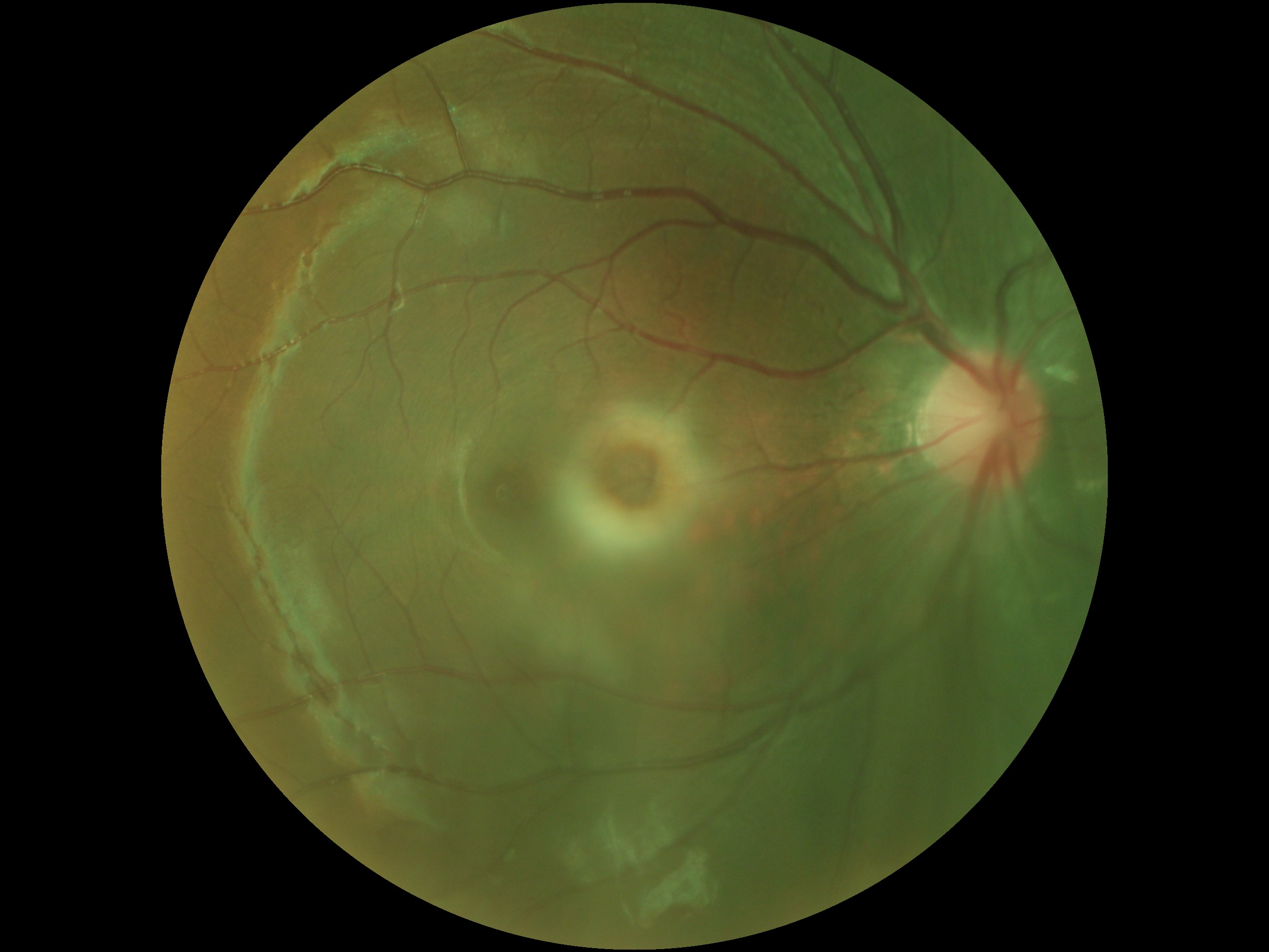

Supplement: S4 File — (ZIP) [file pone.0324352.s004.zip › Original fundus photographs (2)/Subject 106/OD_20230613104059_20230614161335_2.jpg]

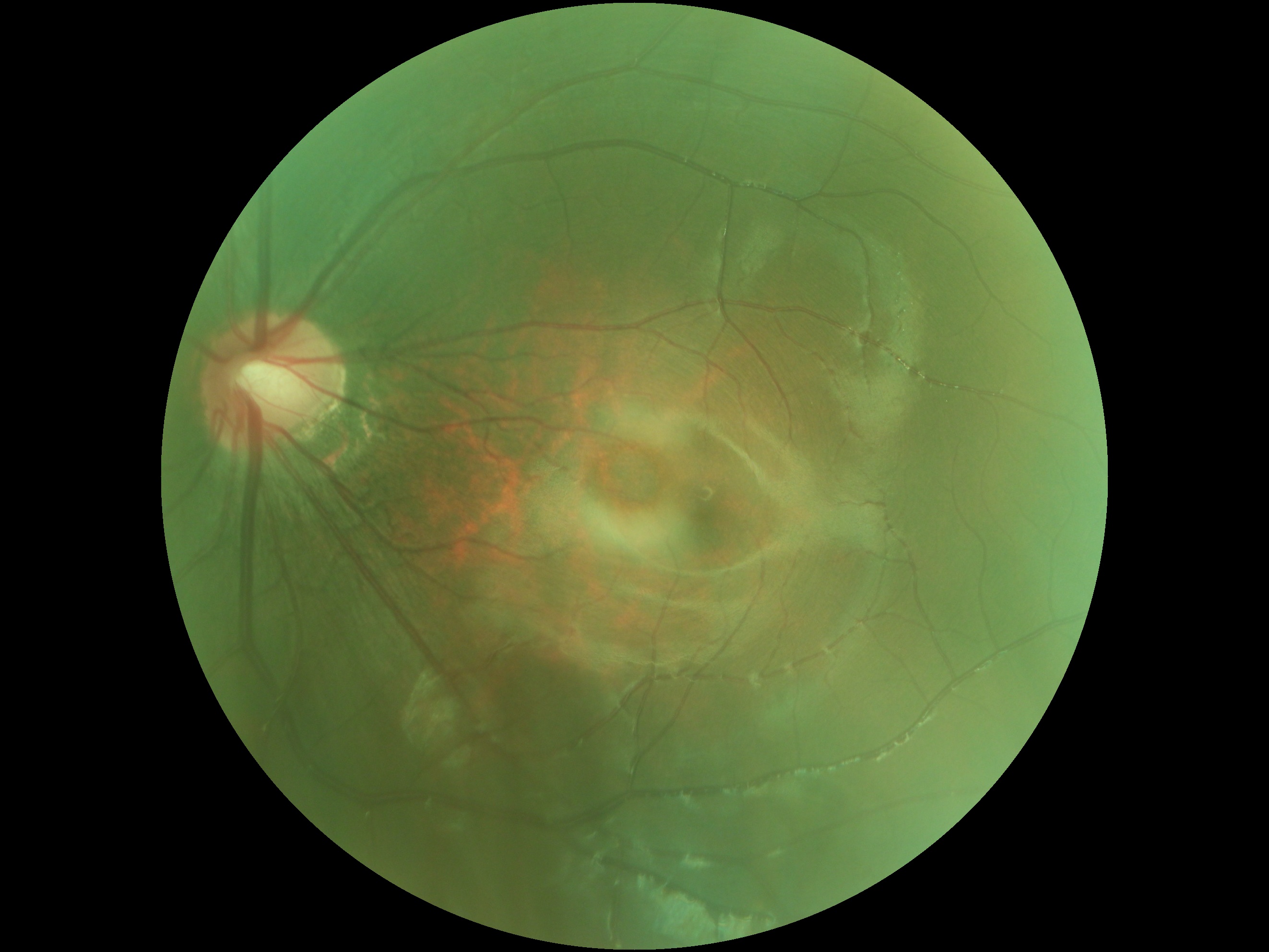

Supplement: S4 File — (ZIP) [file pone.0324352.s004.zip › Original fundus photographs (2)/Subject 106/OS_20230613104059_20230614161456_3.jpg]

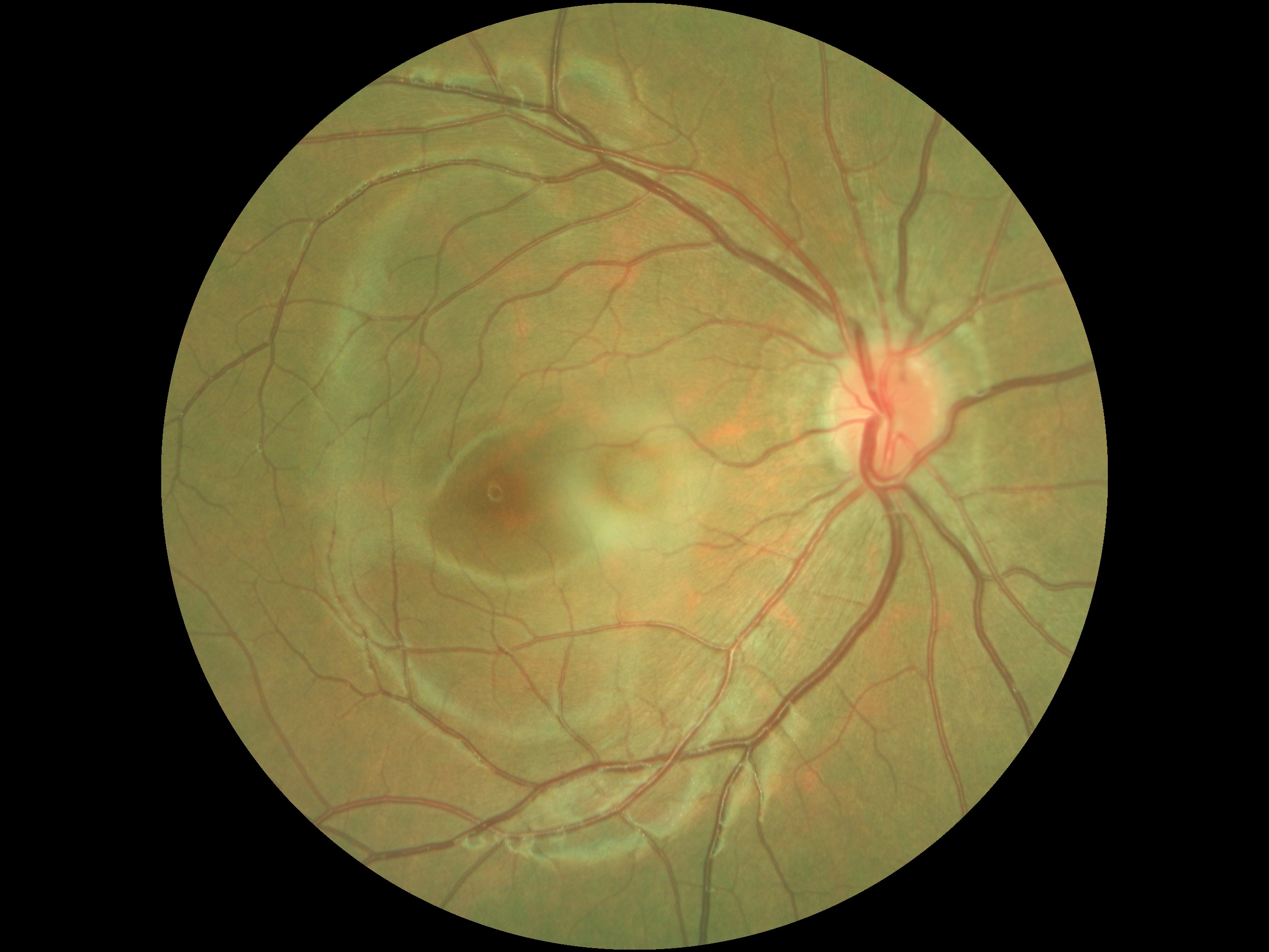

Supplement: S4 File — (ZIP) [file pone.0324352.s004.zip › Original fundus photographs (2)/Subject 107/OD_20230611727163_20230614161734_1.jpg]

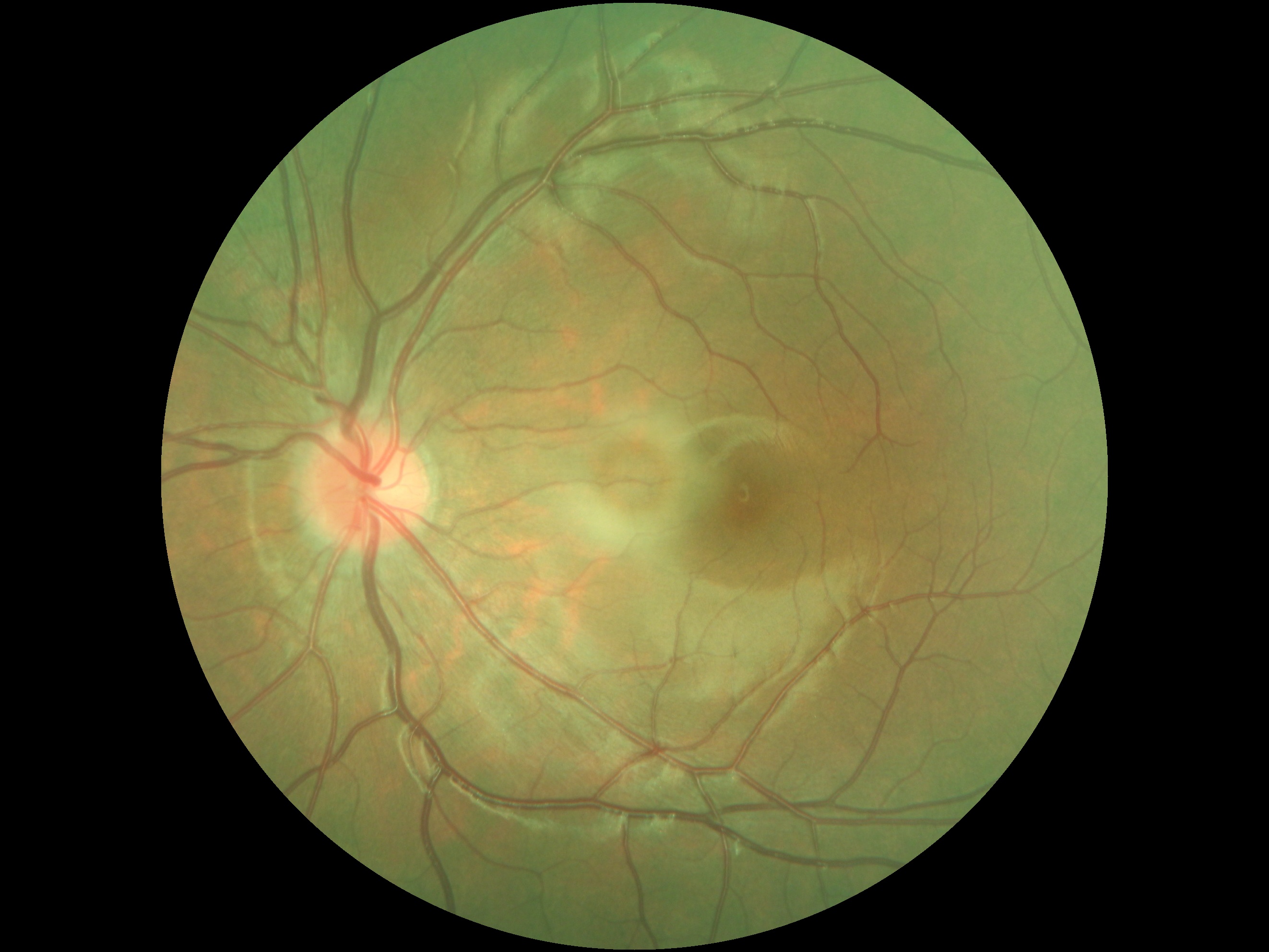

Supplement: S4 File — (ZIP) [file pone.0324352.s004.zip › Original fundus photographs (2)/Subject 107/OS_20230611727163_20230614161804_2.jpg]

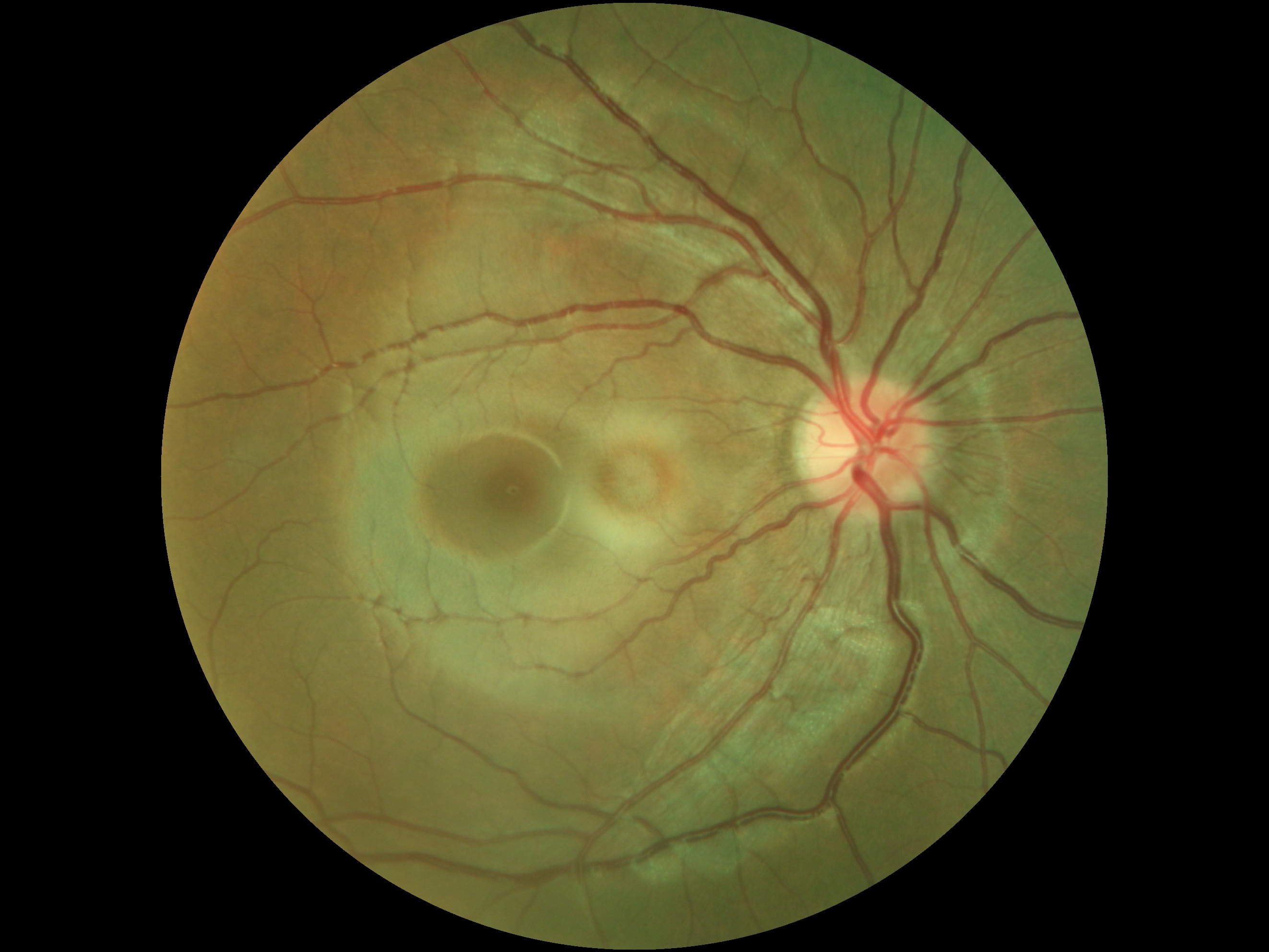

Supplement: S4 File — (ZIP) [file pone.0324352.s004.zip › Original fundus photographs (2)/Subject 108/OD_20230611171199_20230615112844_1.jpg]

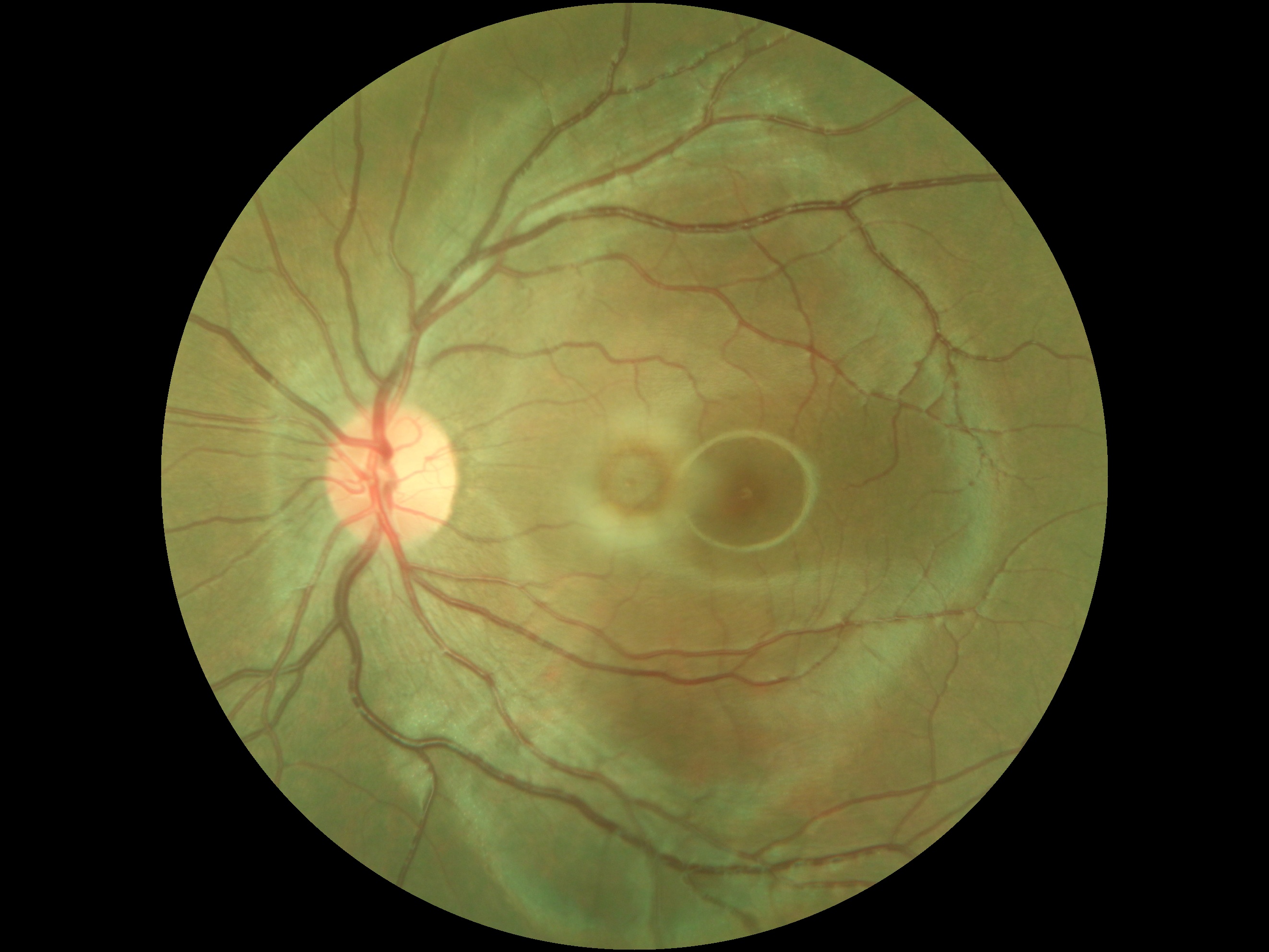

Supplement: S4 File — (ZIP) [file pone.0324352.s004.zip › Original fundus photographs (2)/Subject 108/OS_20230611171199_20230615112907_2.jpg]

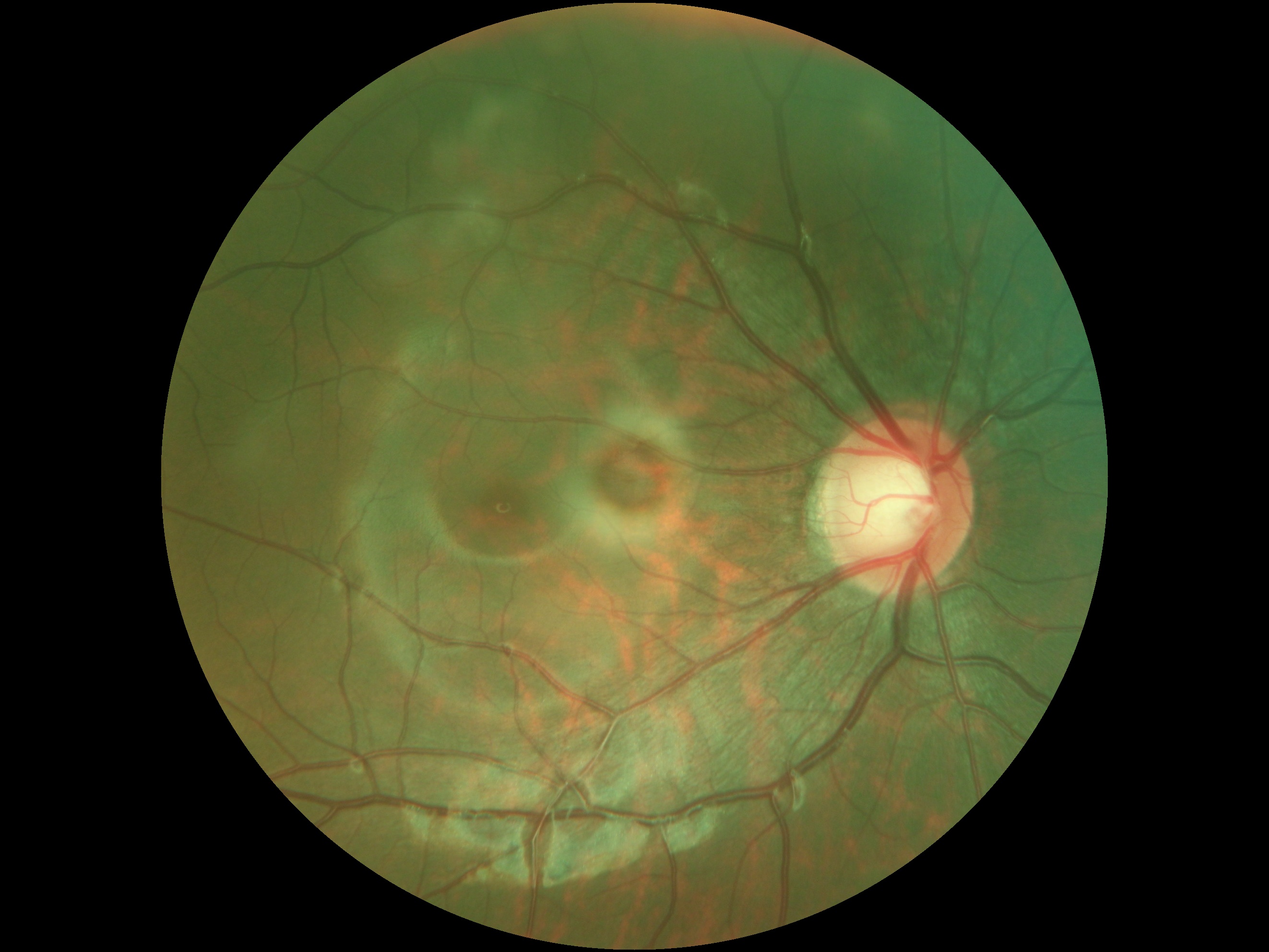

Supplement: S4 File — (ZIP) [file pone.0324352.s004.zip › Original fundus photographs (2)/Subject 109/OD_20230611412264_20230615103124_2.jpg]

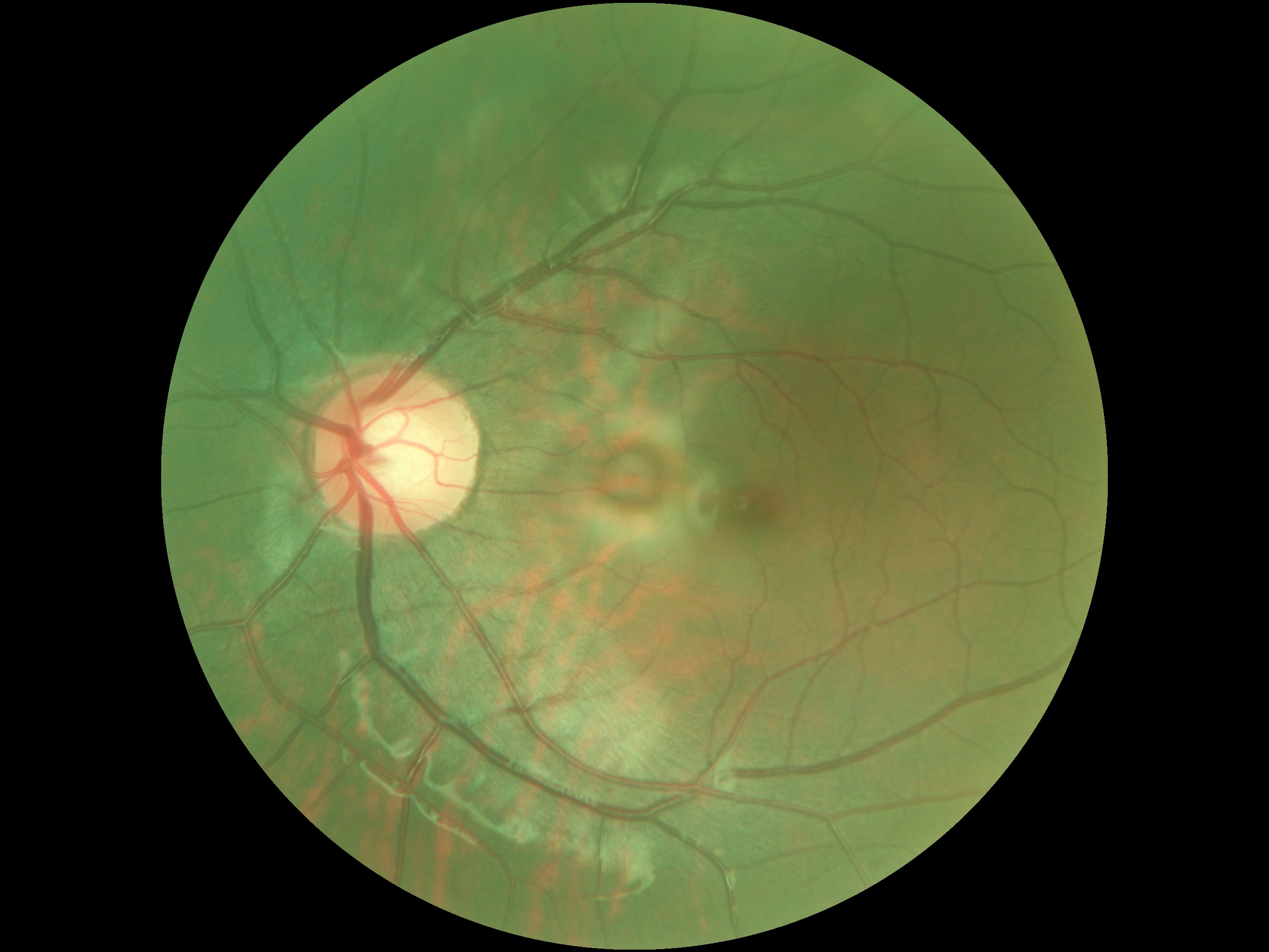

Supplement: S4 File — (ZIP) [file pone.0324352.s004.zip › Original fundus photographs (2)/Subject 109/OS_20230611412264_20230615103231_4.jpg]

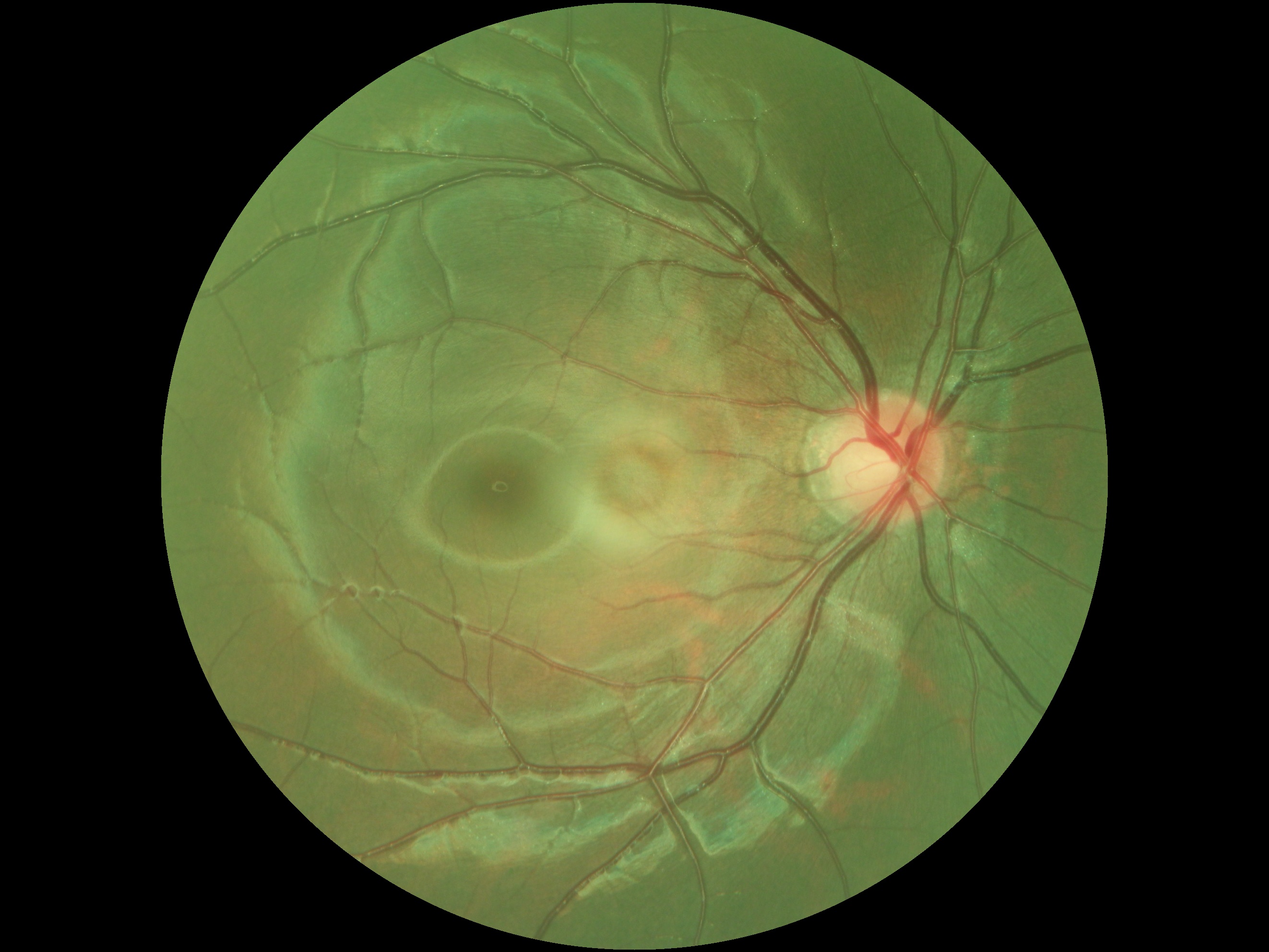

Supplement: S4 File — (ZIP) [file pone.0324352.s004.zip › Original fundus photographs (2)/Subject 110/OD_20230611392198_20230615112956_1.jpg]

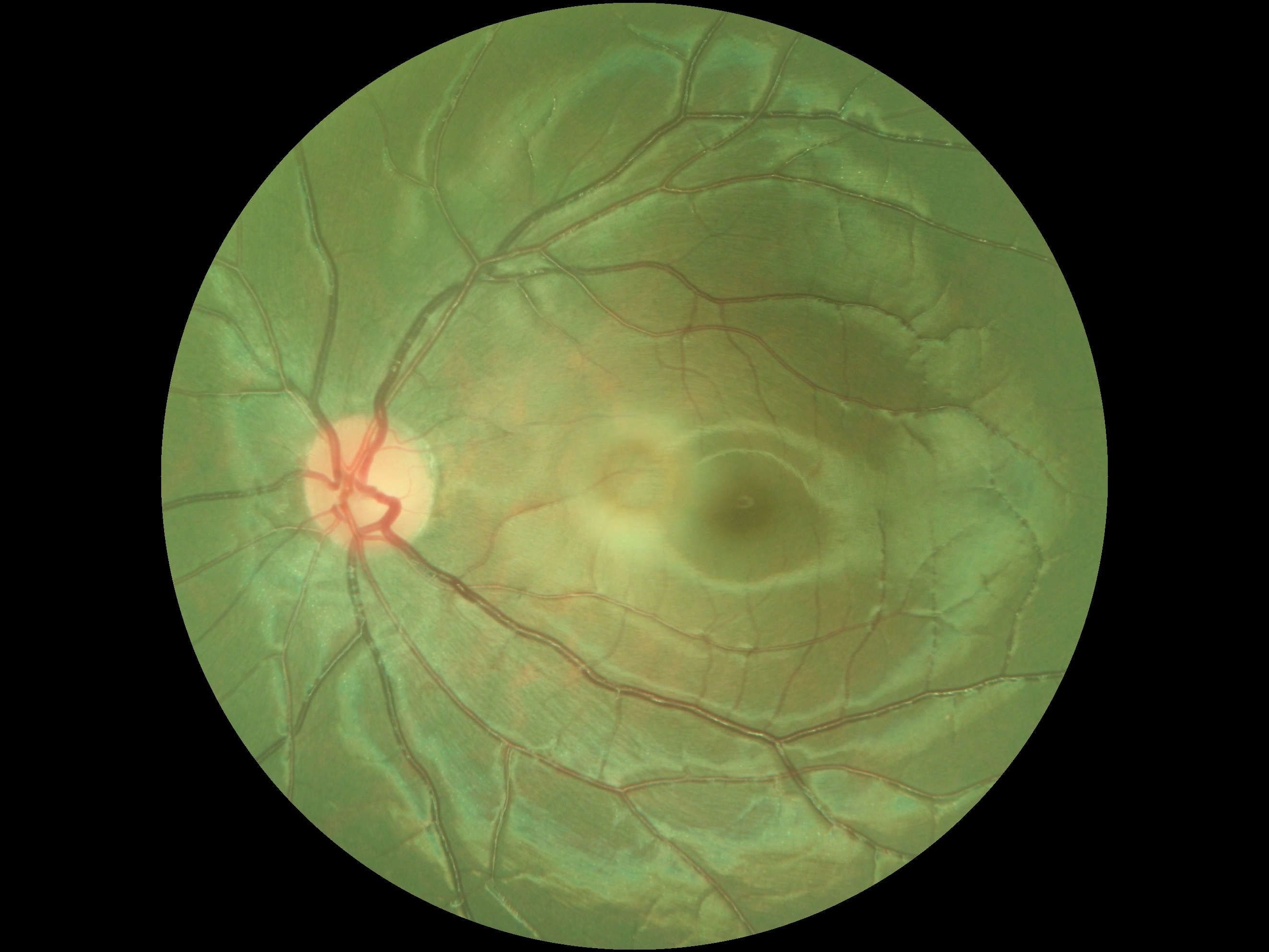

Supplement: S4 File — (ZIP) [file pone.0324352.s004.zip › Original fundus photographs (2)/Subject 110/OS_20230611392198_20230615113008_2.jpg]

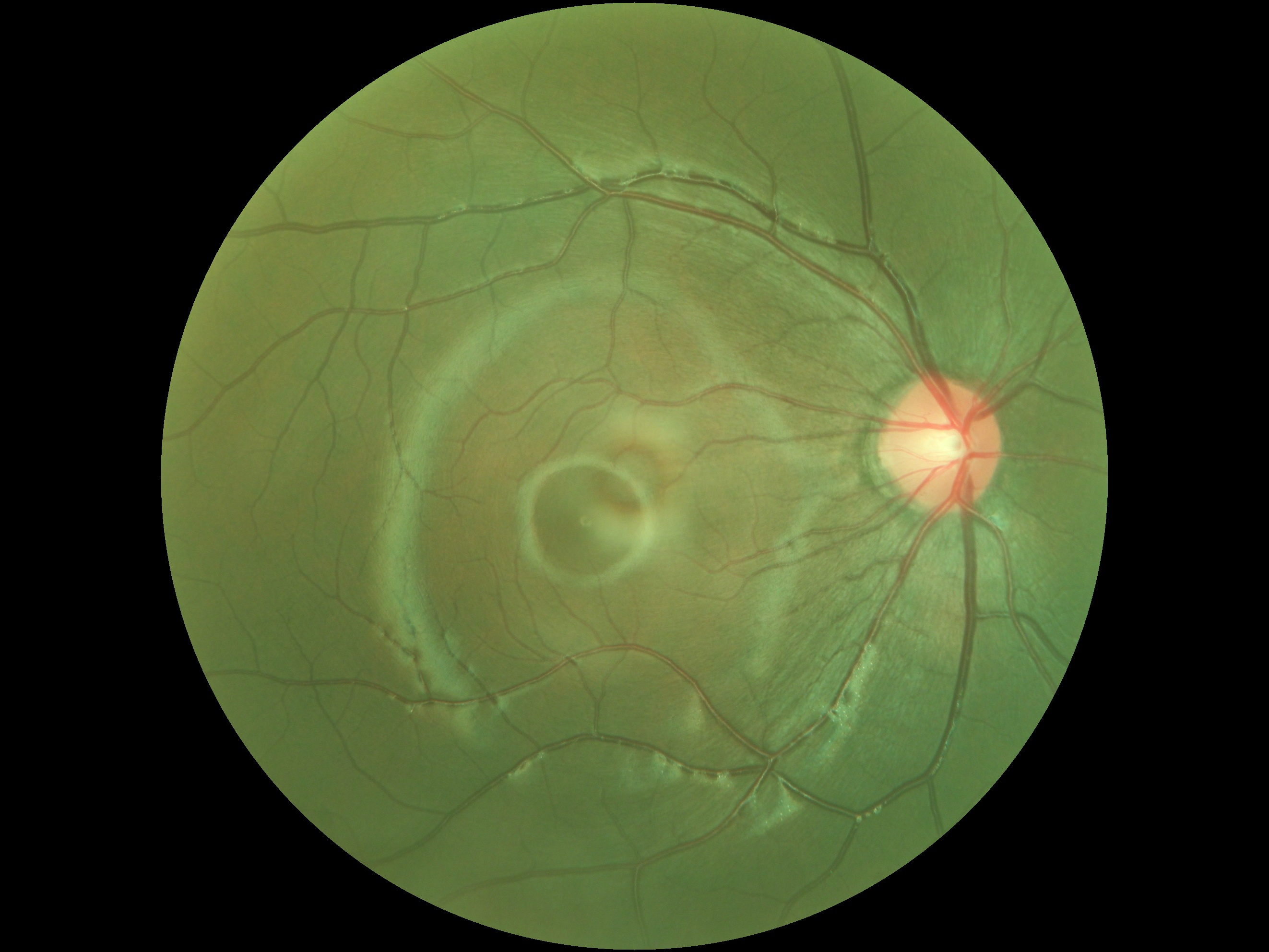

Supplement: S4 File — (ZIP) [file pone.0324352.s004.zip › Original fundus photographs (2)/Subject 111/OD_20230611554263_20230615102809_3.jpg]

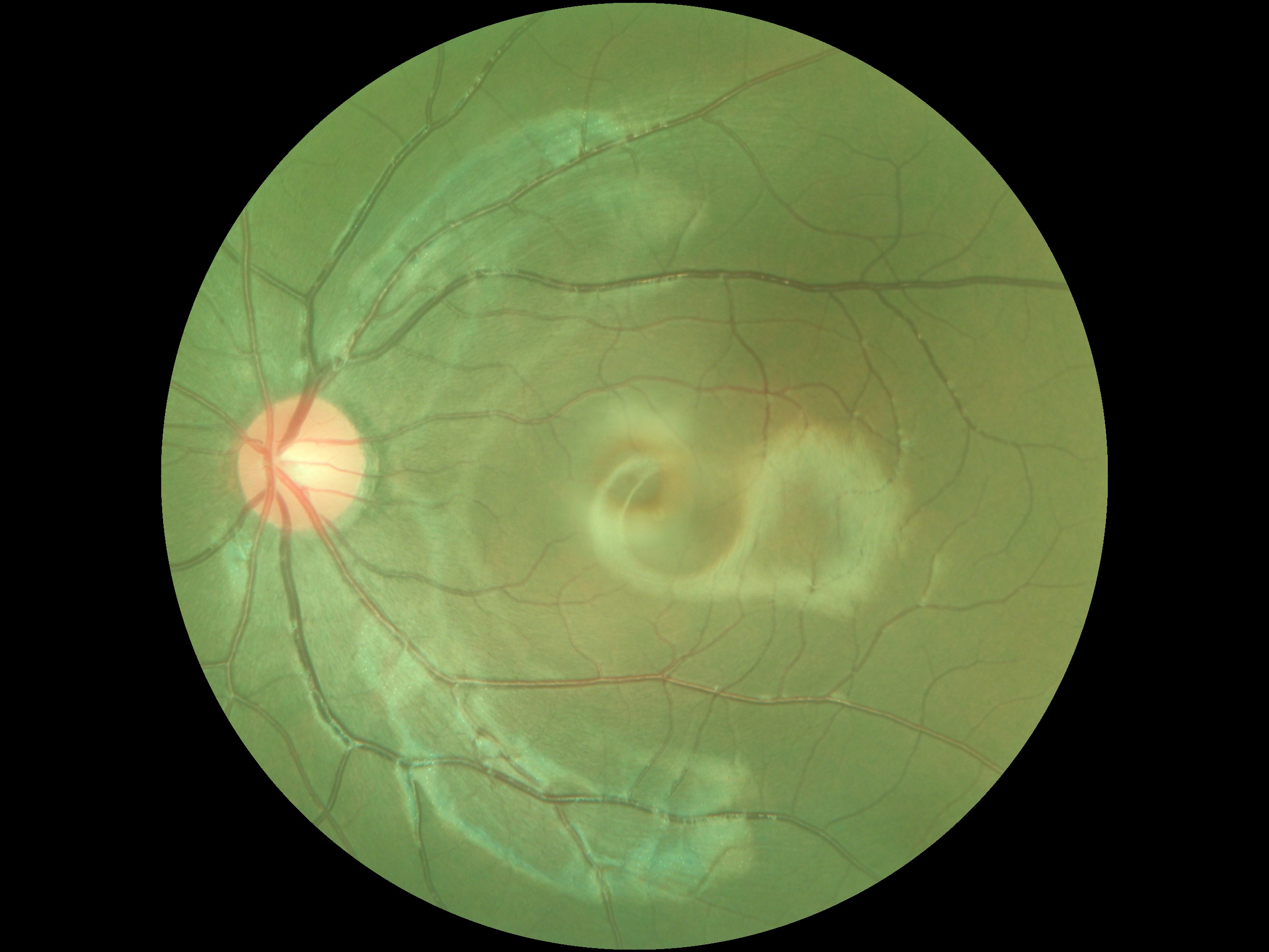

Supplement: S4 File — (ZIP) [file pone.0324352.s004.zip › Original fundus photographs (2)/Subject 111/OS_20230611554263_20230615102904_5.jpg]

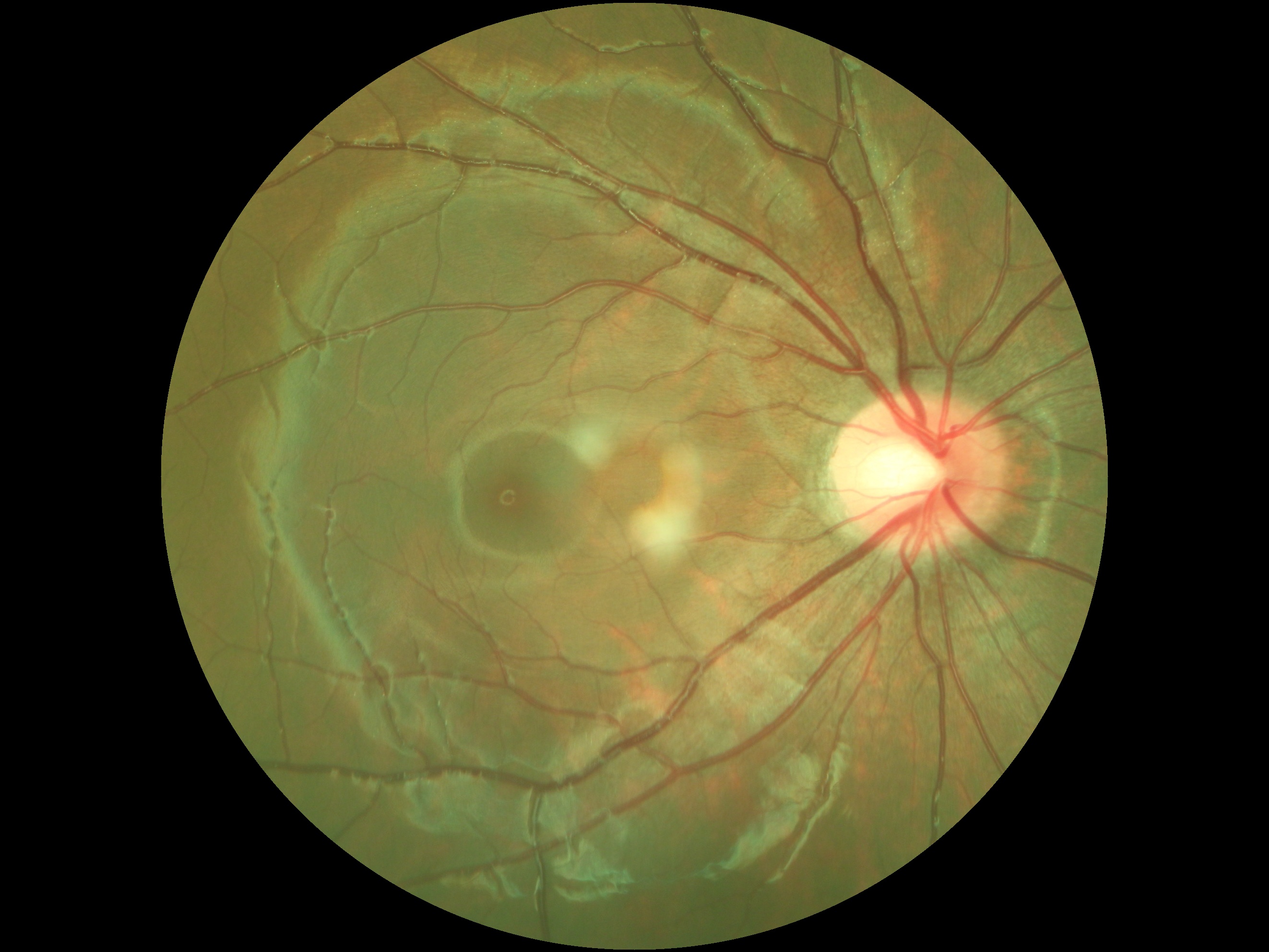

Supplement: S4 File — (ZIP) [file pone.0324352.s004.zip › Original fundus photographs (2)/Subject 112/OD_20230611119053_20230615165741_2.jpg]

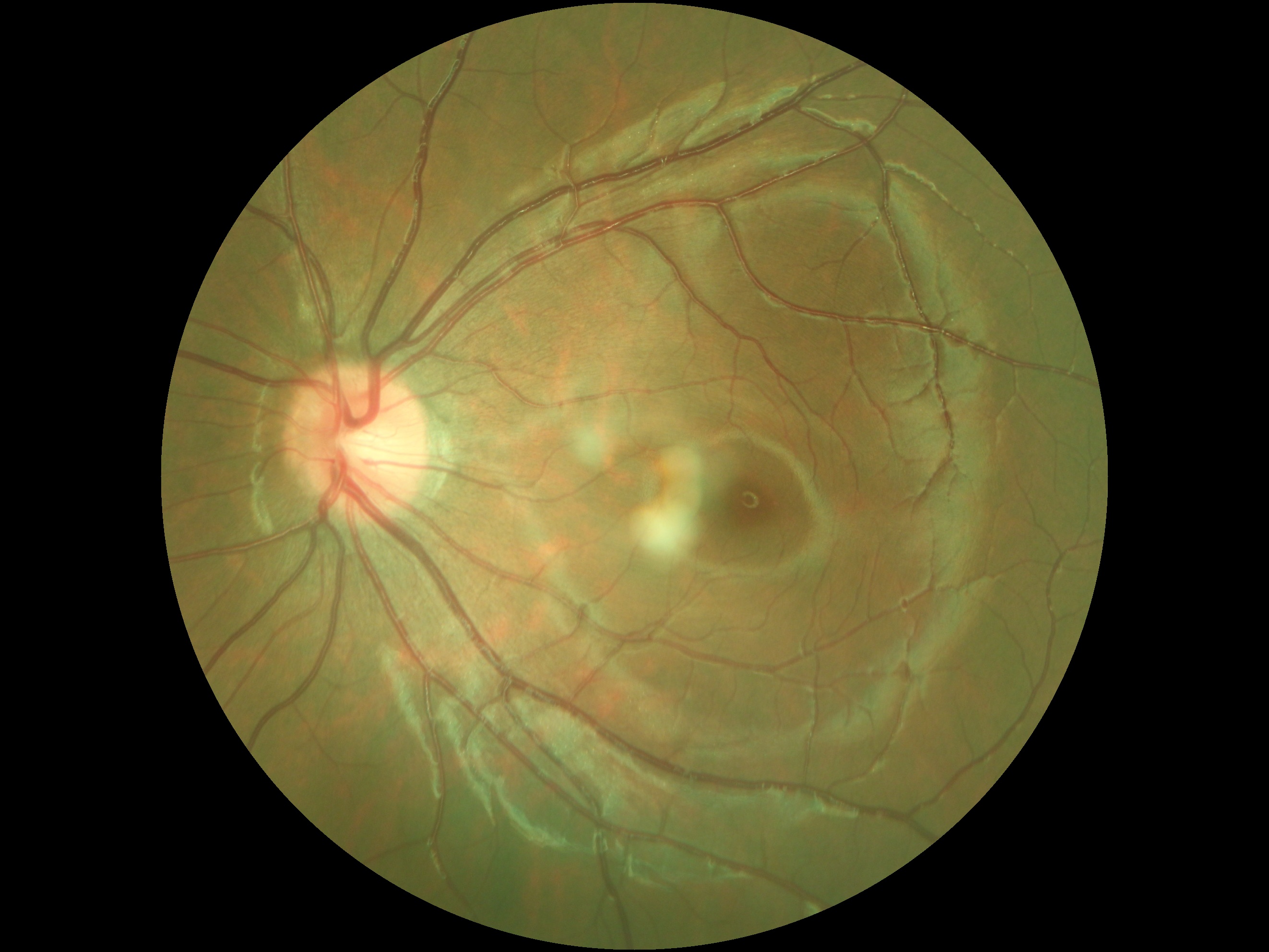

Supplement: S4 File — (ZIP) [file pone.0324352.s004.zip › Original fundus photographs (2)/Subject 112/OS_20230611119053_20230615165719_1.jpg]

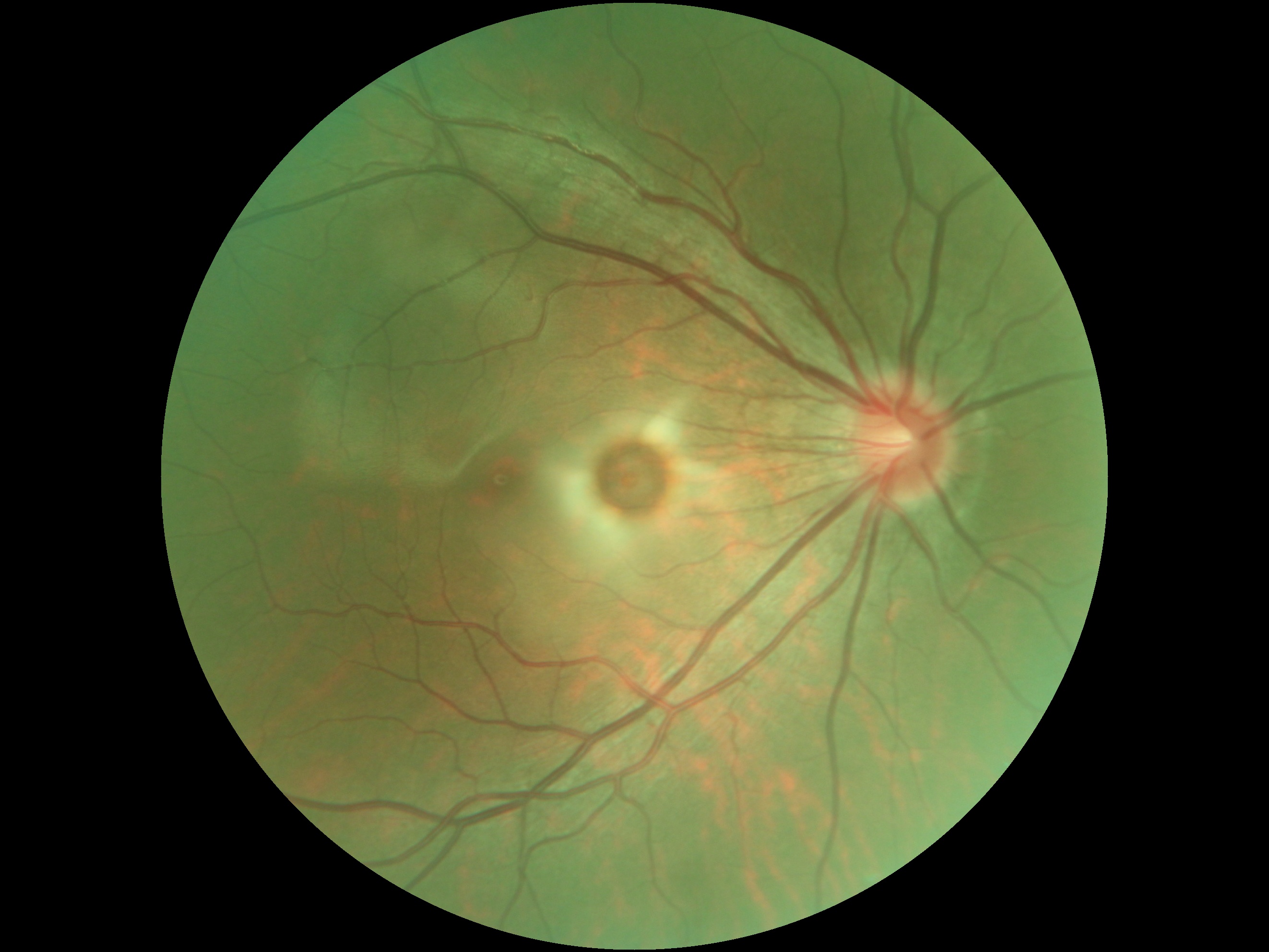

Supplement: S4 File — (ZIP) [file pone.0324352.s004.zip › Original fundus photographs (2)/Subject 113/OD_20230611843120_20230613171533_2.jpg]

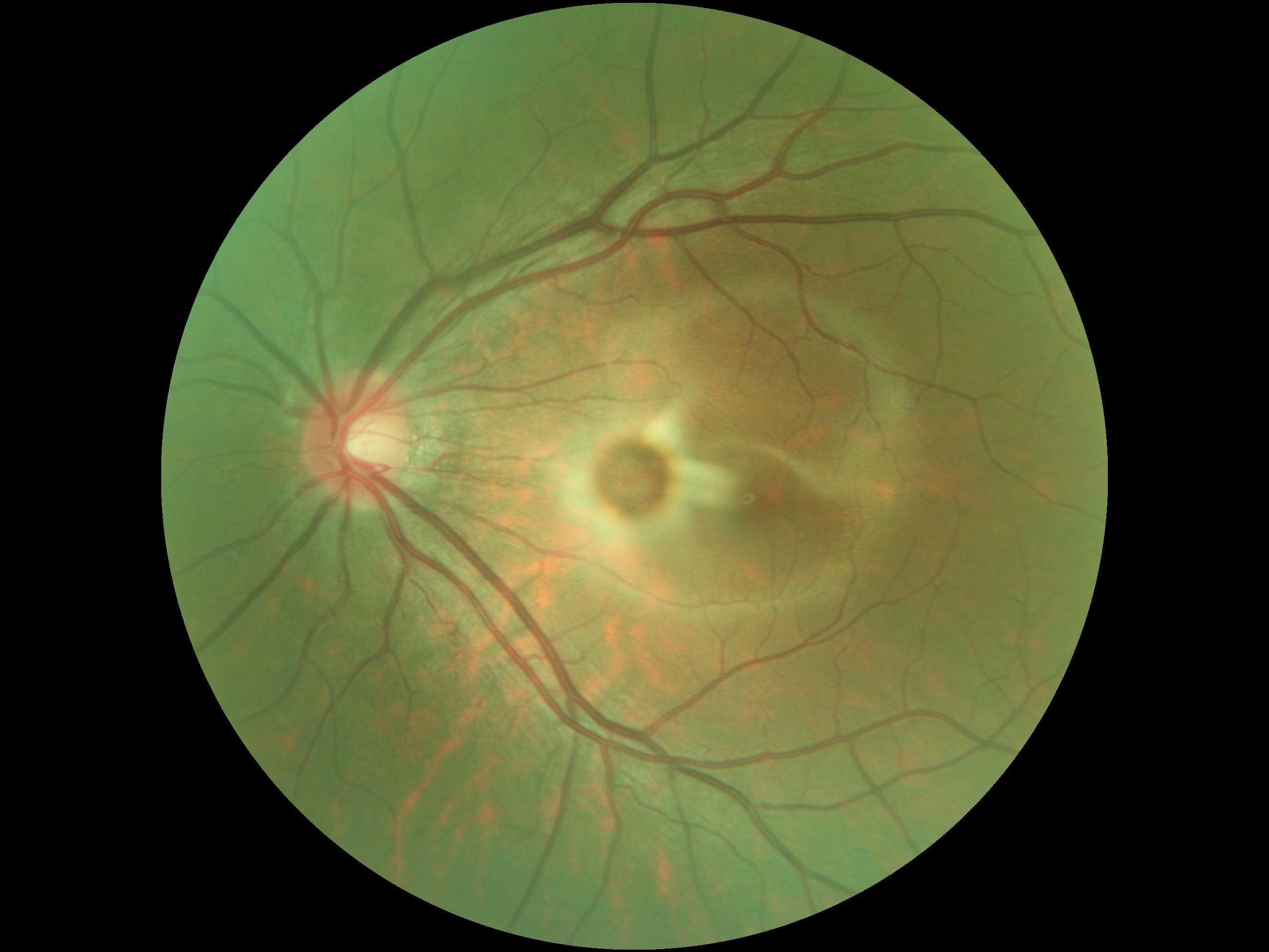

Supplement: S4 File — (ZIP) [file pone.0324352.s004.zip › Original fundus photographs (2)/Subject 113/OS_20230611843120_20230613171450_1.jpg]

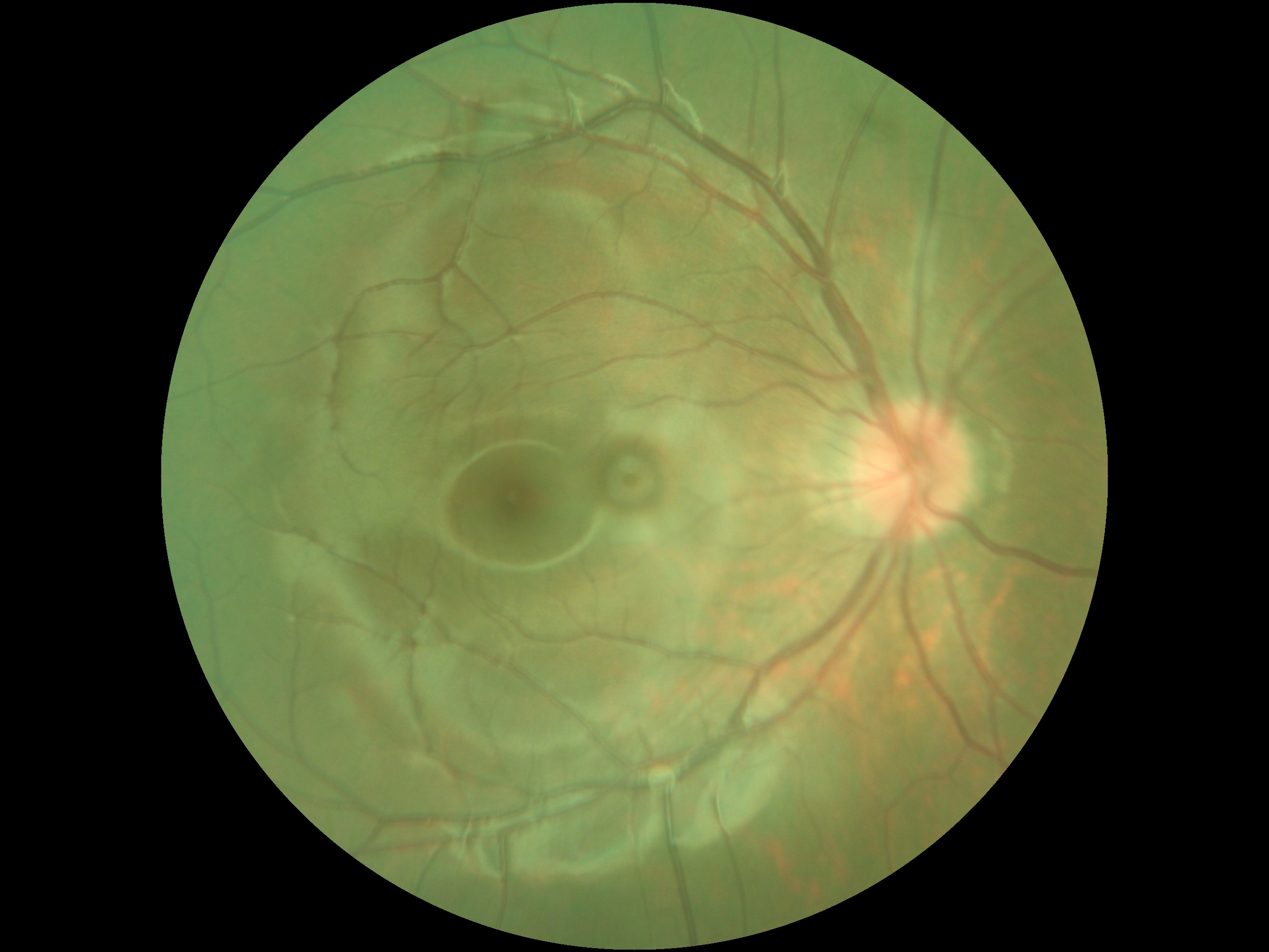

Supplement: S4 File — (ZIP) [file pone.0324352.s004.zip › Original fundus photographs (2)/Subject 114/OD_20230611899183_20230614095749_1.jpg]

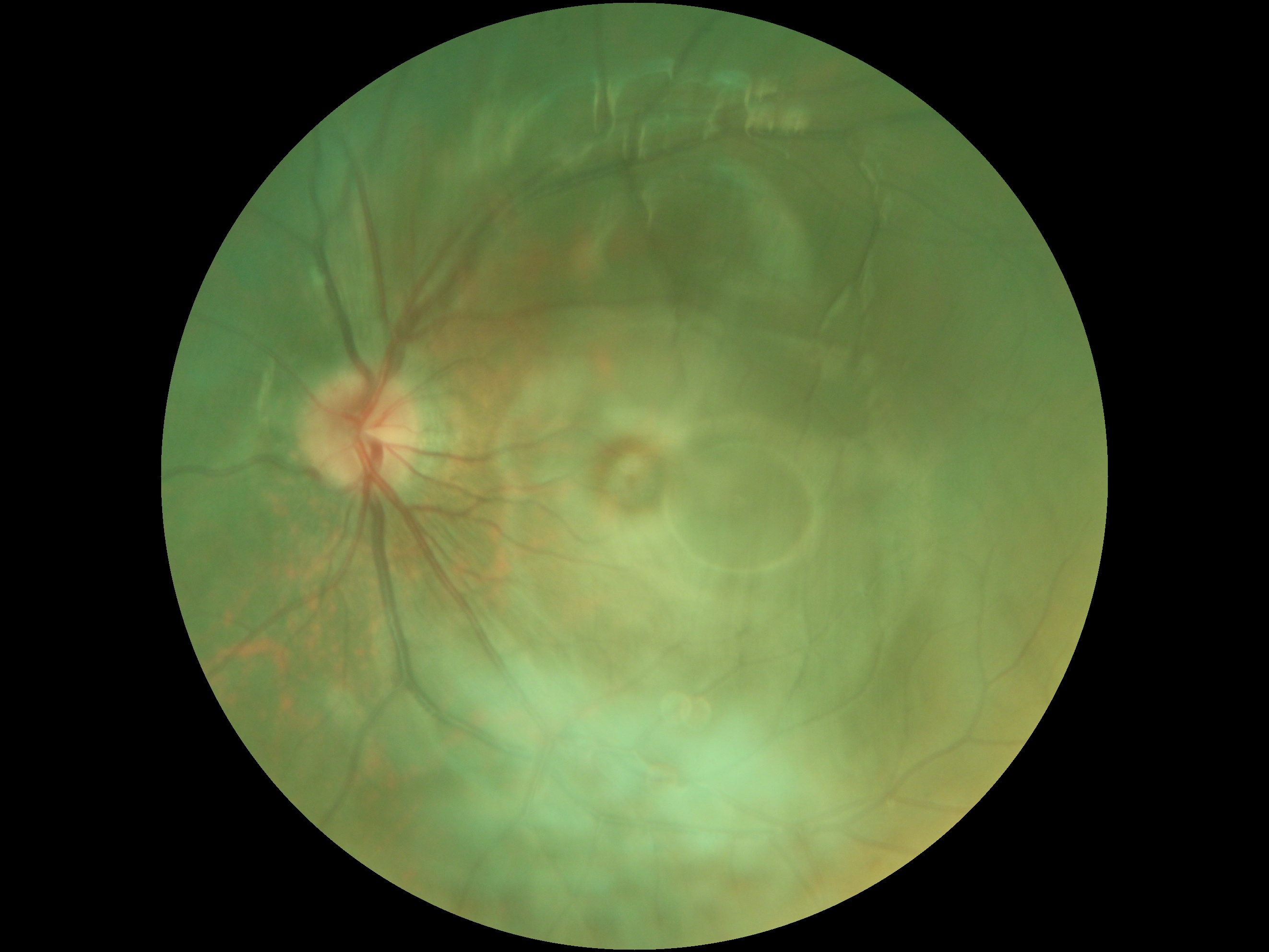

Supplement: S4 File — (ZIP) [file pone.0324352.s004.zip › Original fundus photographs (2)/Subject 114/OS_20230611899183_20230614095815_2.jpg]

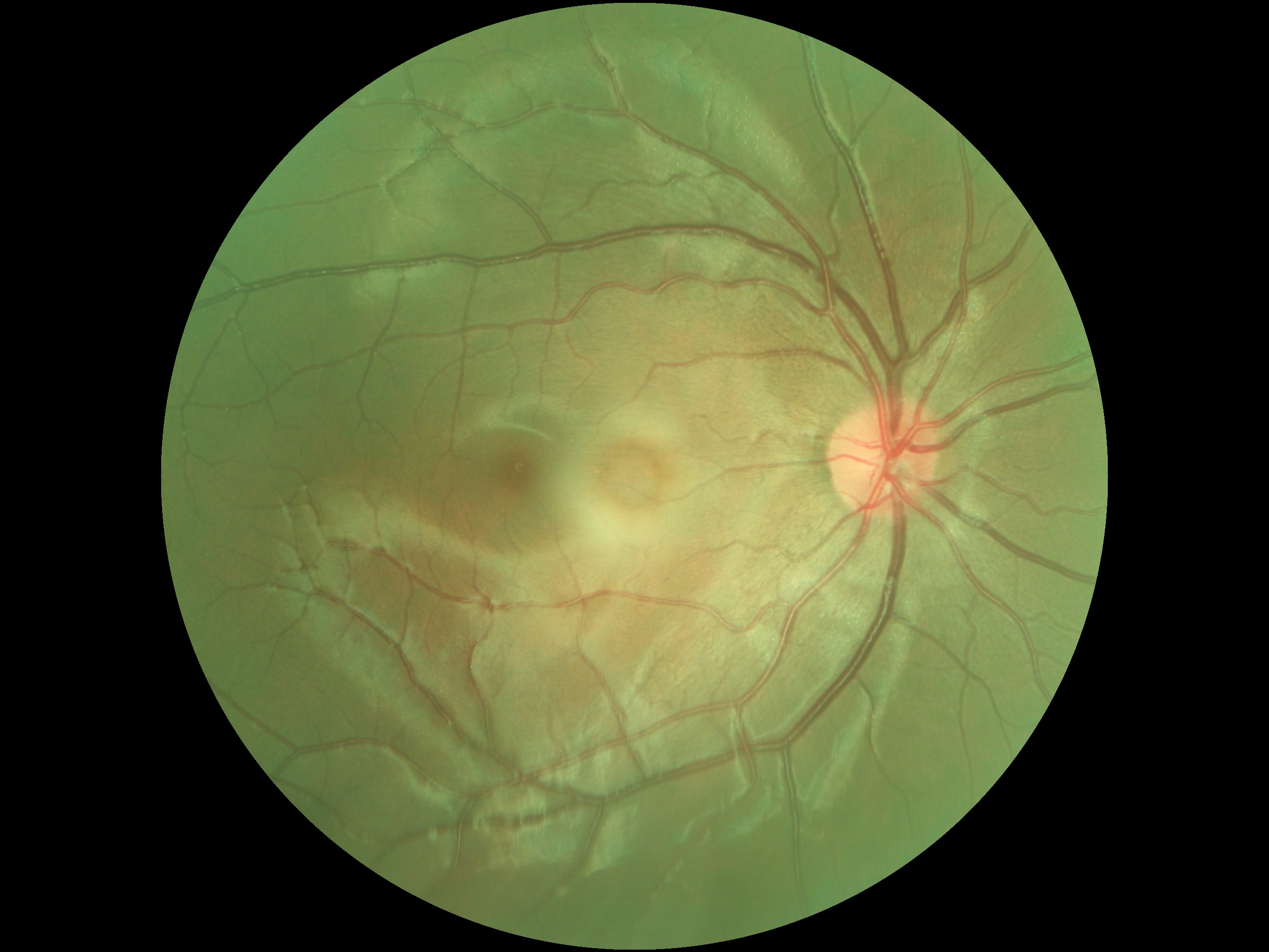

Supplement: S4 File — (ZIP) [file pone.0324352.s004.zip › Original fundus photographs (2)/Subject 115/OD_20230611583294_20230614155757_1.jpg]

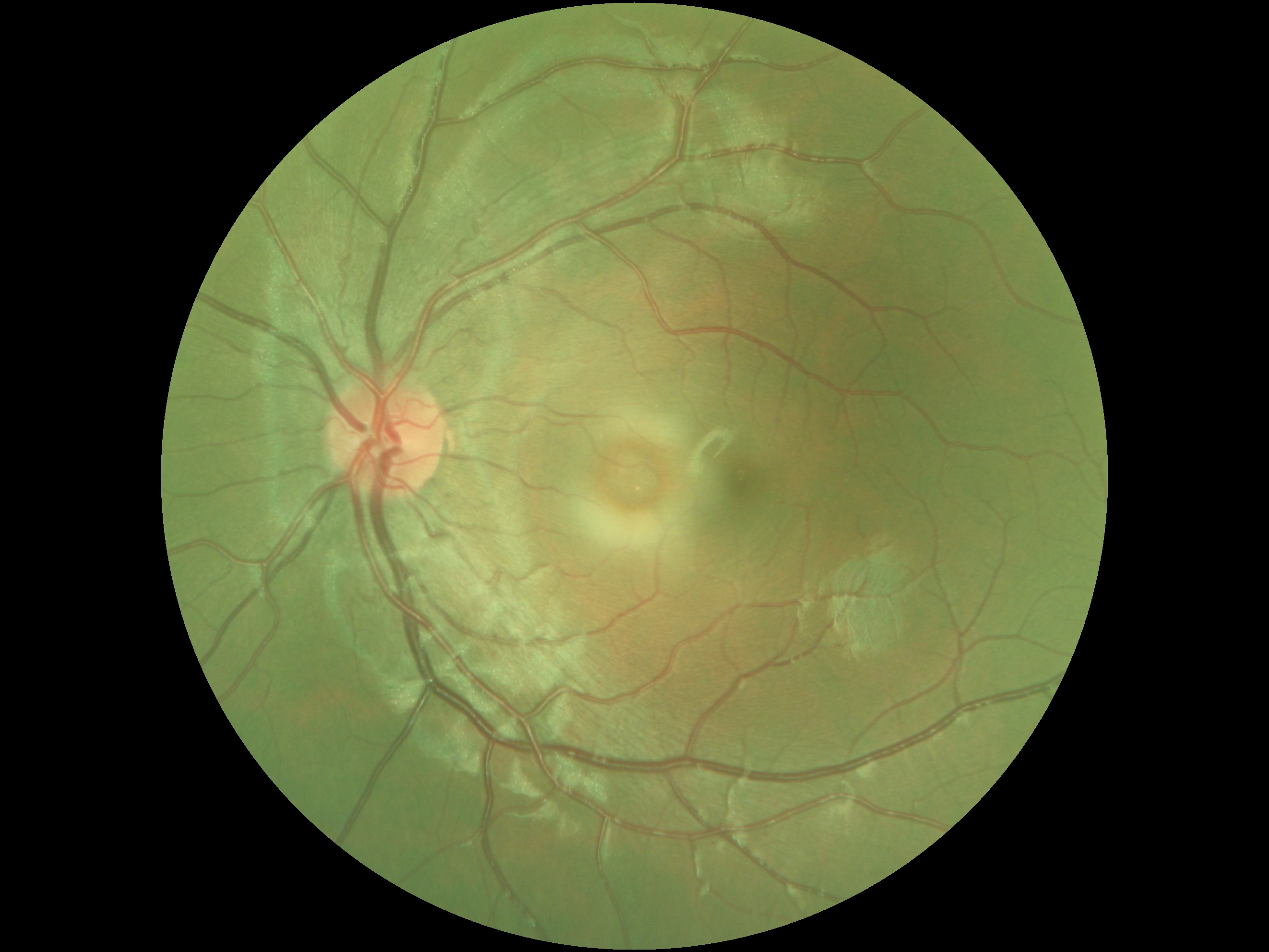

Supplement: S4 File — (ZIP) [file pone.0324352.s004.zip › Original fundus photographs (2)/Subject 115/OS_20230611583294_20230614155818_2.jpg]

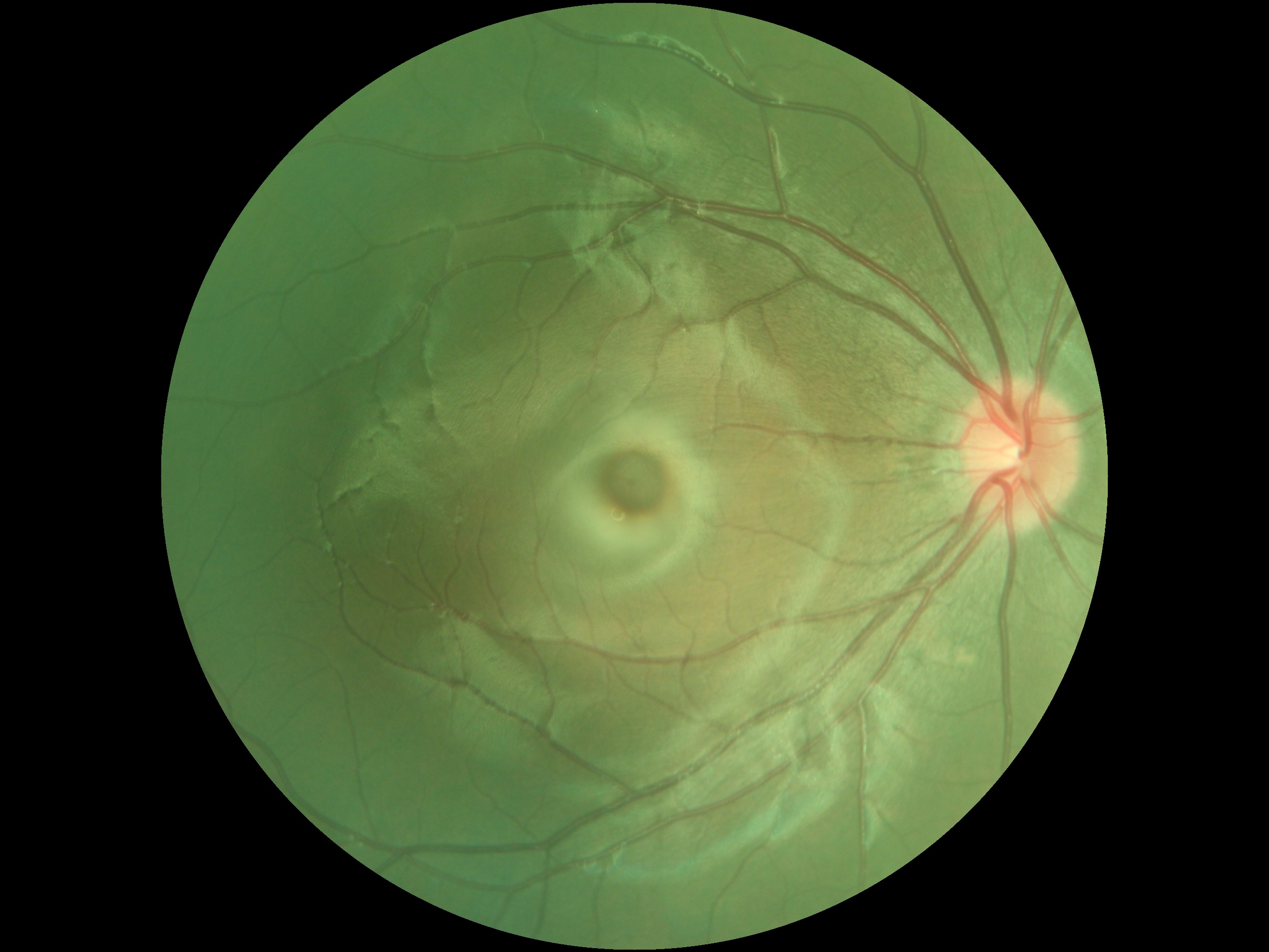

Supplement: S4 File — (ZIP) [file pone.0324352.s004.zip › Original fundus photographs (2)/Subject 116/OD_20230611738081_20230615152654_1.jpg]

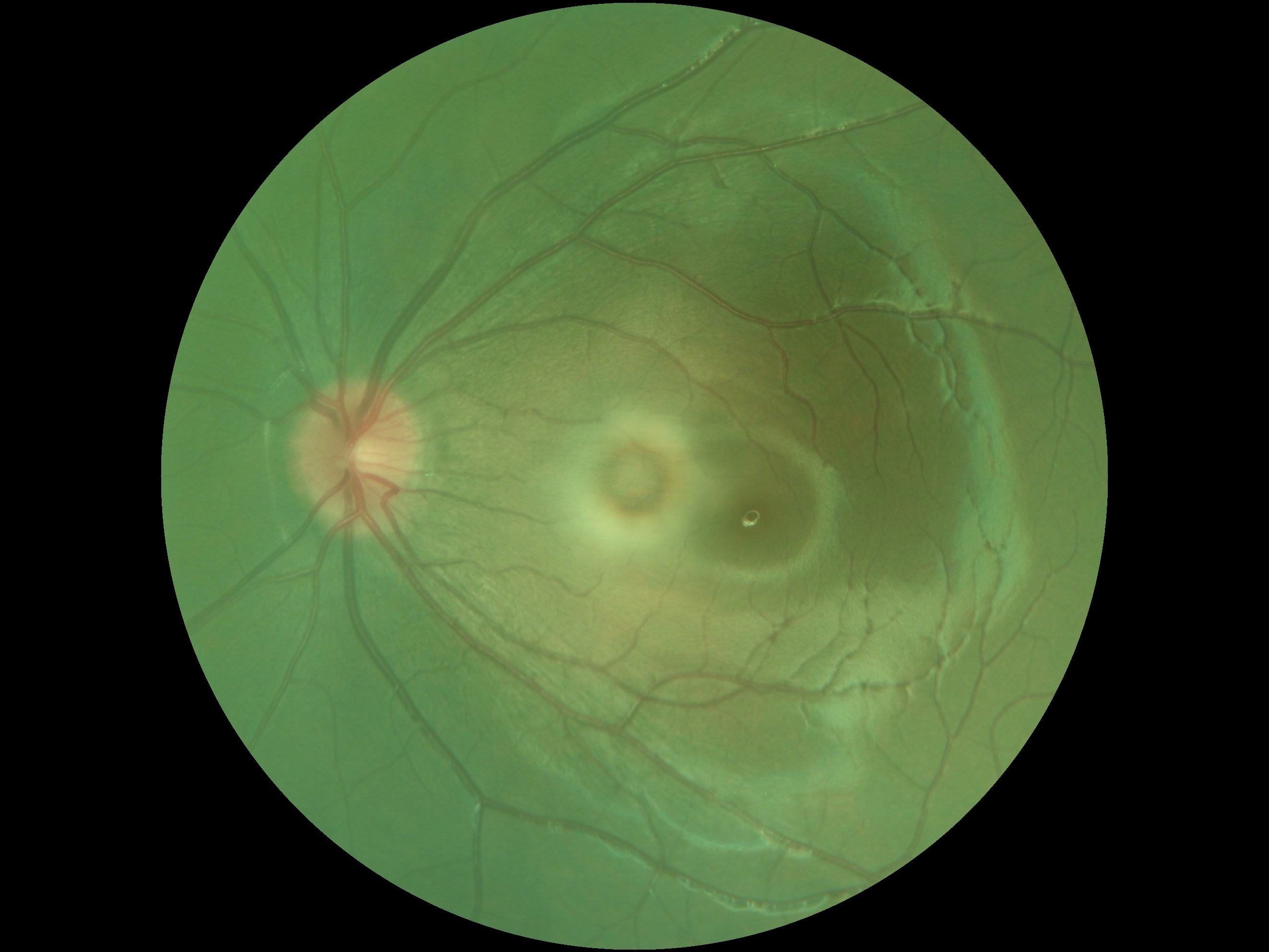

Supplement: S4 File — (ZIP) [file pone.0324352.s004.zip › Original fundus photographs (2)/Subject 116/OS_20230611738081_20230615152704_2.jpg]

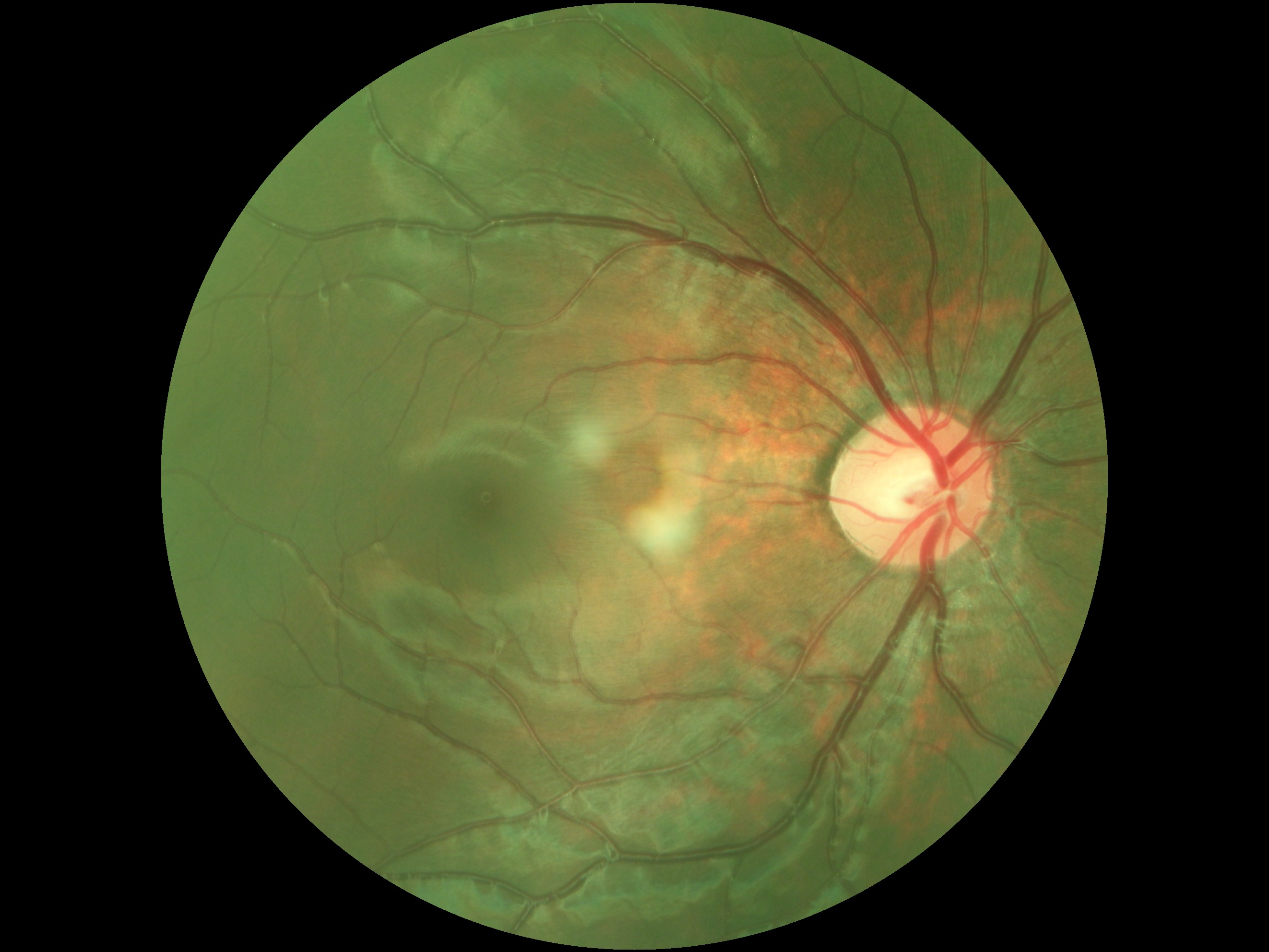

Supplement: S4 File — (ZIP) [file pone.0324352.s004.zip › Original fundus photographs (2)/Subject 117/OD_20230611555055_20230615165541_1.jpg]

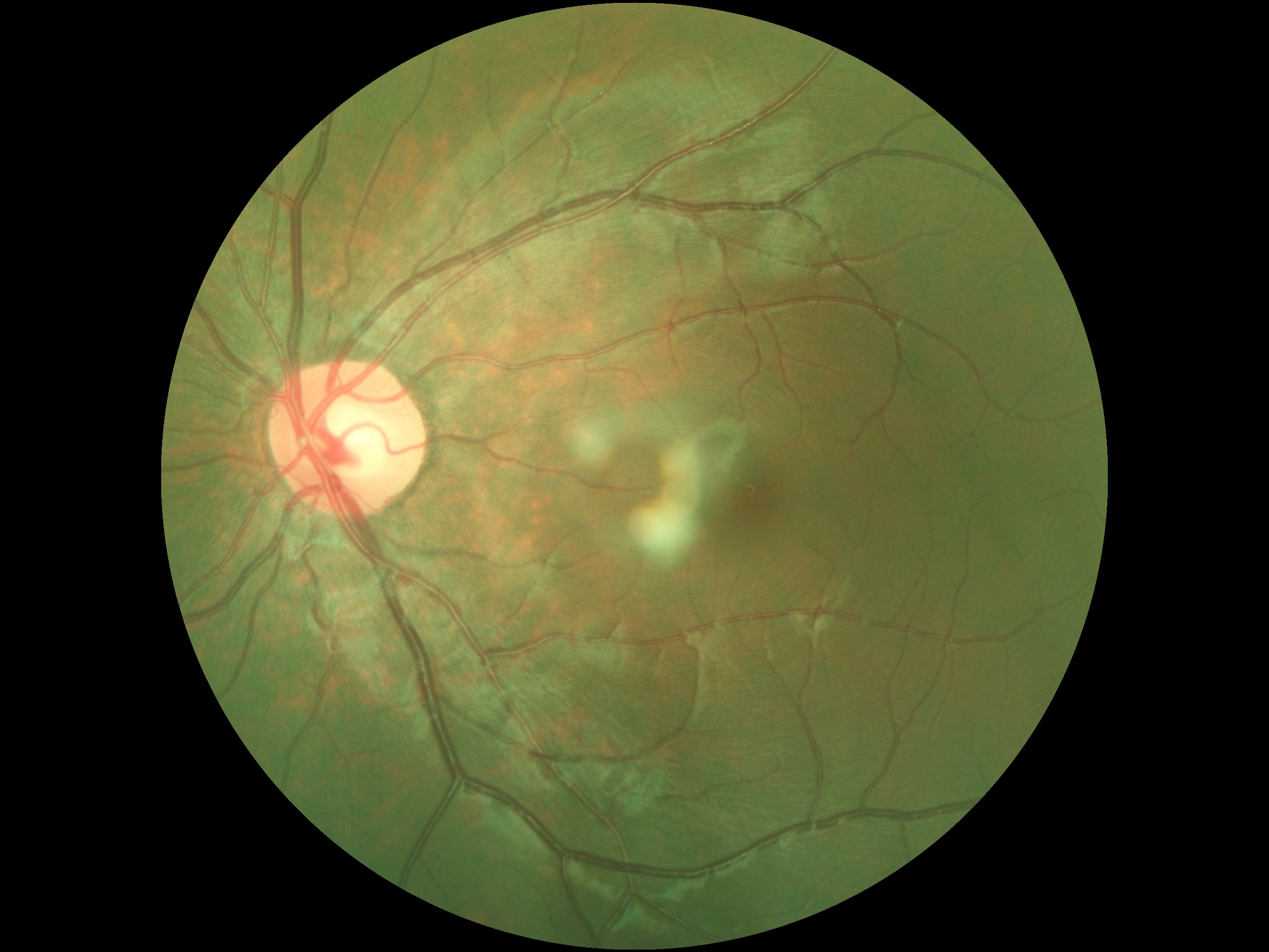

Supplement: S4 File — (ZIP) [file pone.0324352.s004.zip › Original fundus photographs (2)/Subject 117/OS_20230611555055_20230615165623_2.jpg]

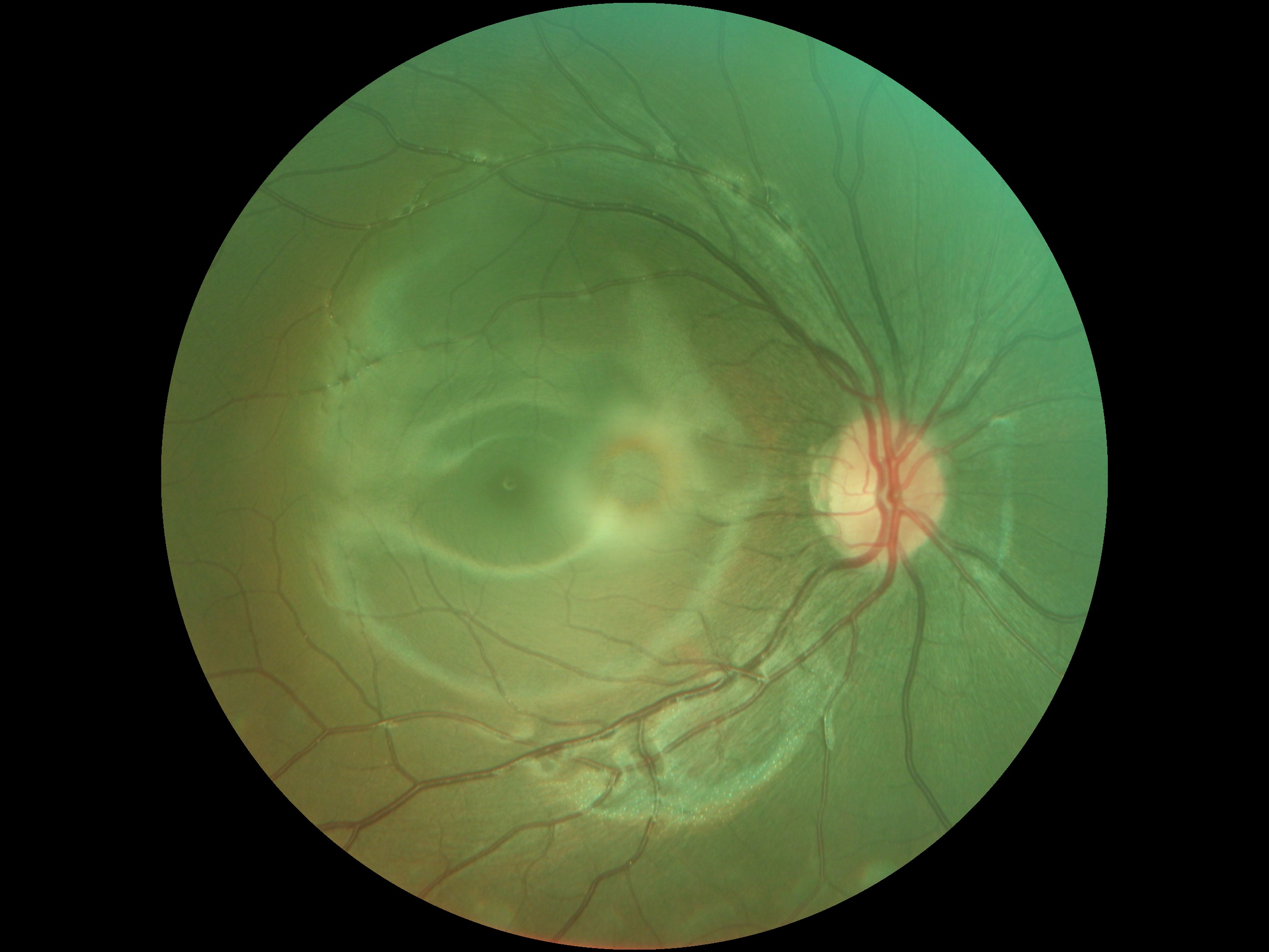

Supplement: S4 File — (ZIP) [file pone.0324352.s004.zip › Original fundus photographs (2)/Subject 118/OD_20230611582112_20230614160413_1.jpg]

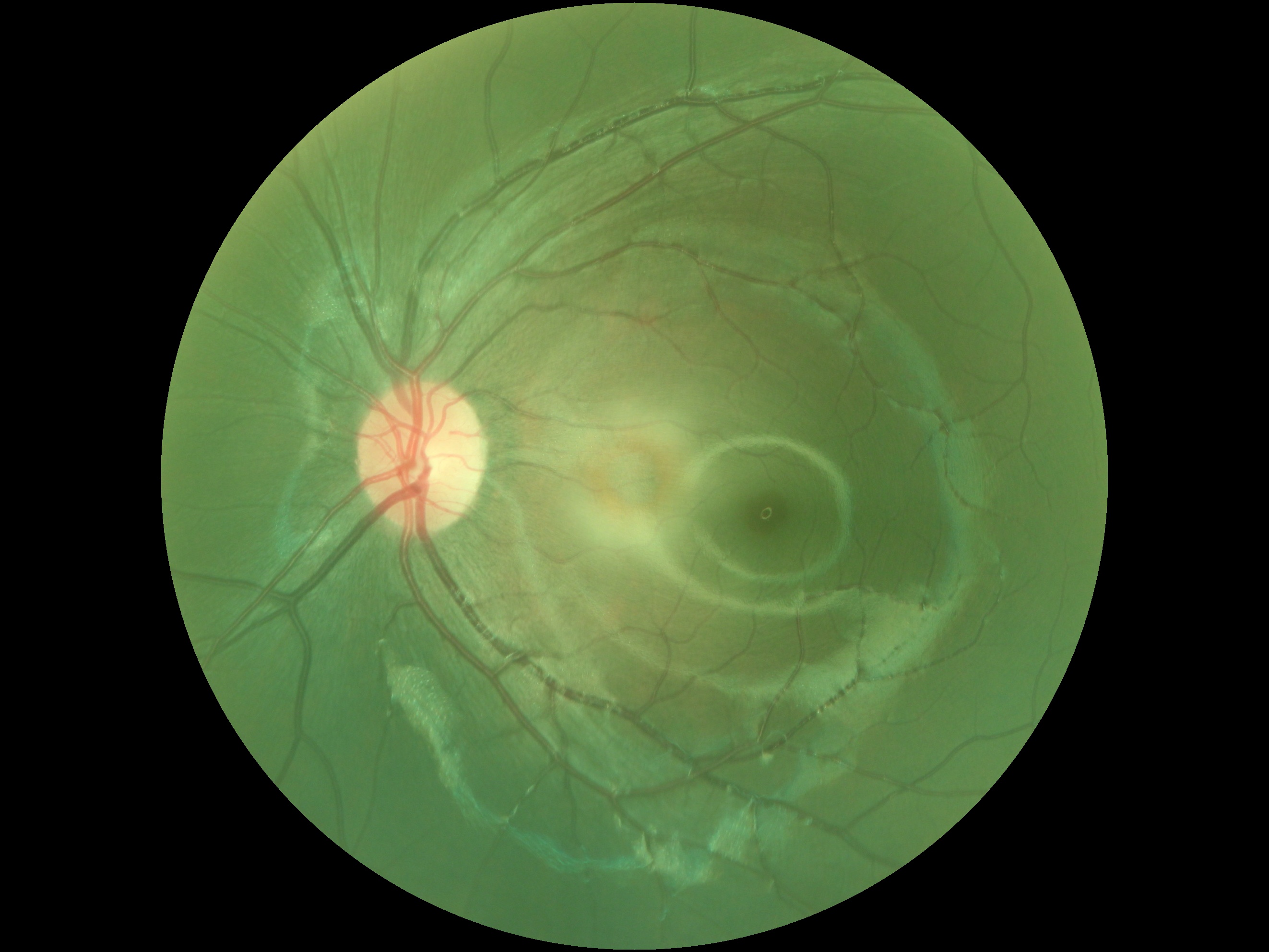

Supplement: S4 File — (ZIP) [file pone.0324352.s004.zip › Original fundus photographs (2)/Subject 118/OS_20230611582112_20230614160551_4.jpg]

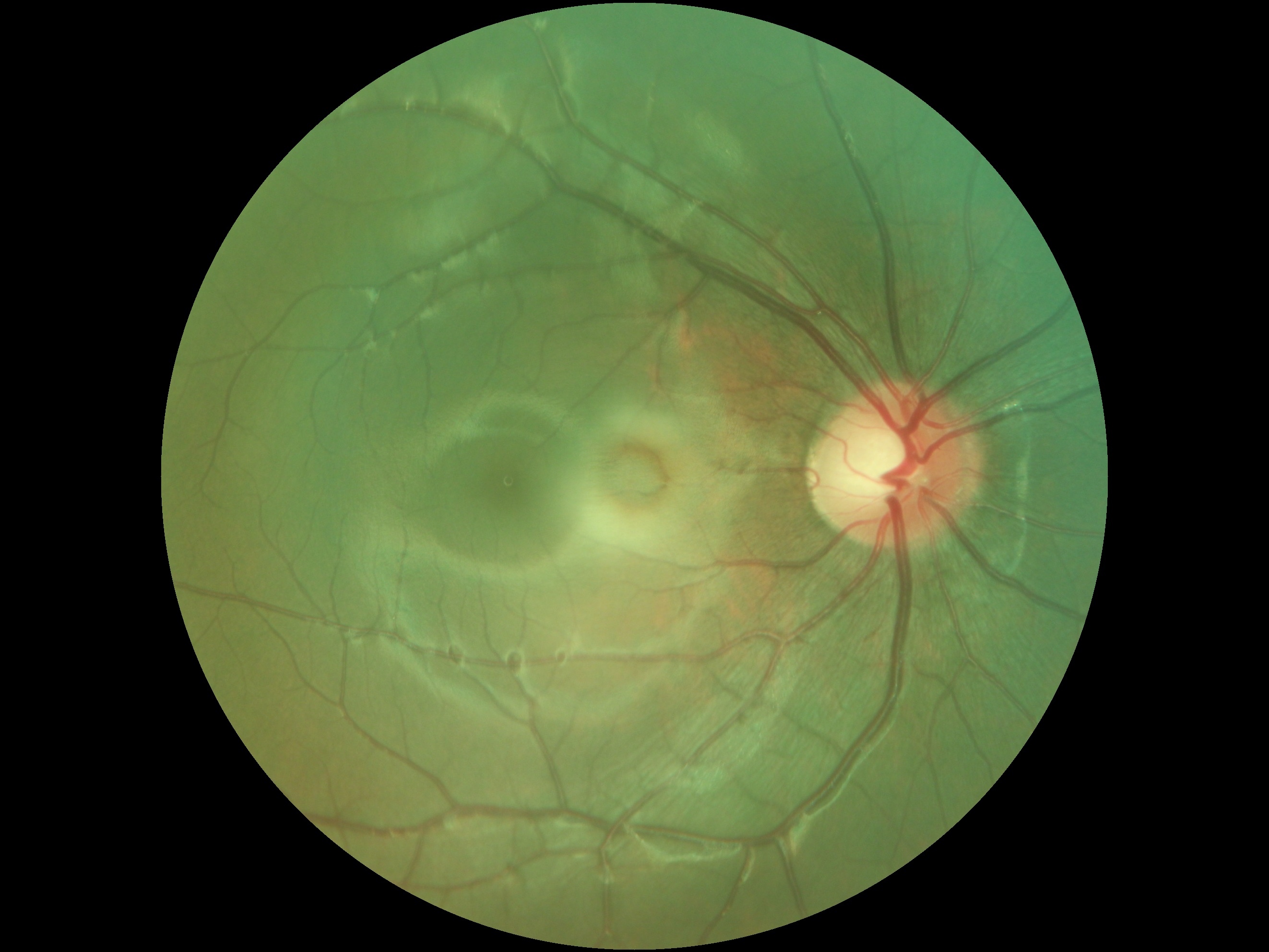

Supplement: S4 File — (ZIP) [file pone.0324352.s004.zip › Original fundus photographs (2)/Subject 119/OD_20230611704092_20230615153156_3.jpg]

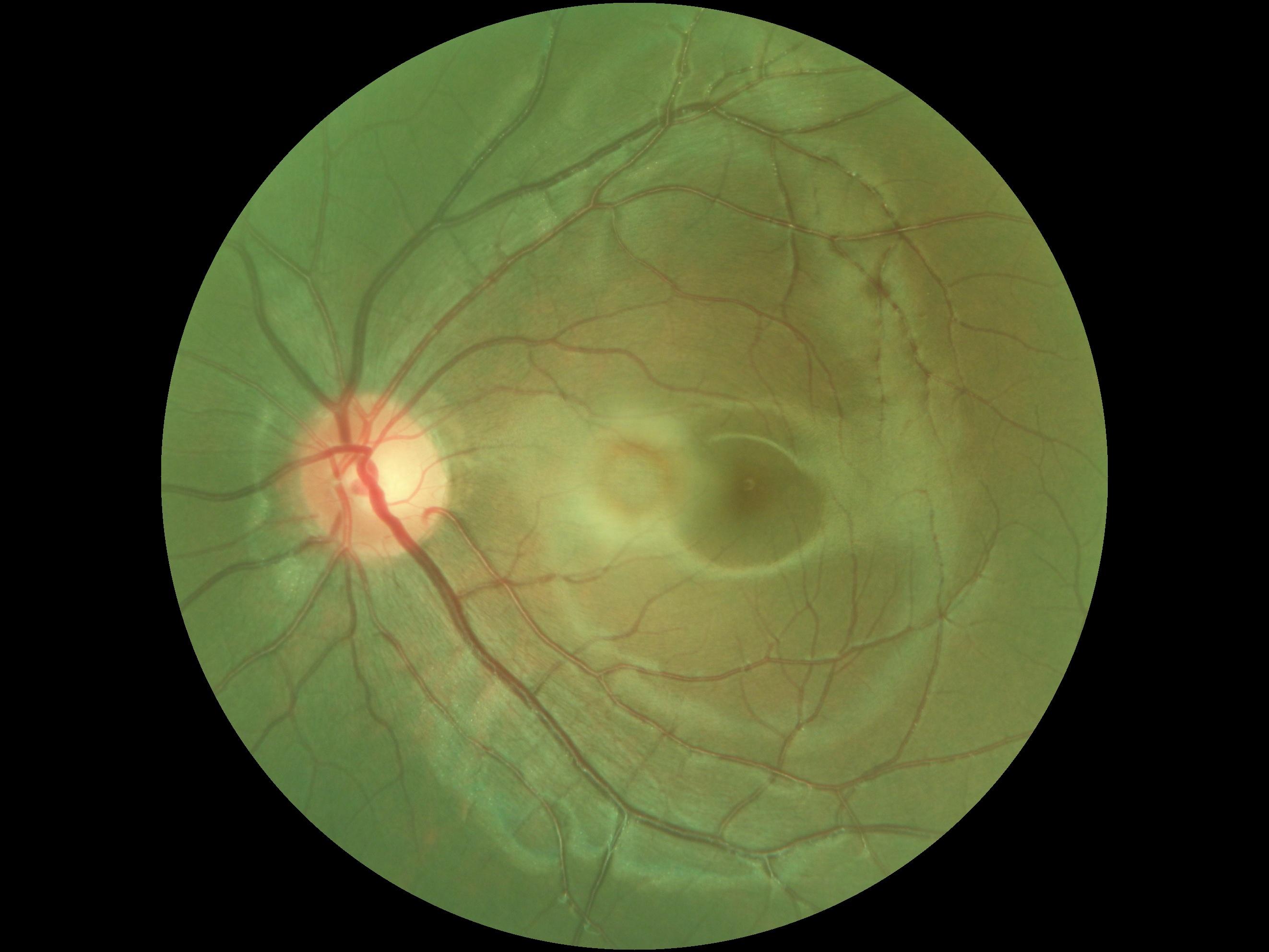

Supplement: S4 File — (ZIP) [file pone.0324352.s004.zip › Original fundus photographs (2)/Subject 119/OS_20230611704092_20230615152737_1.jpg]

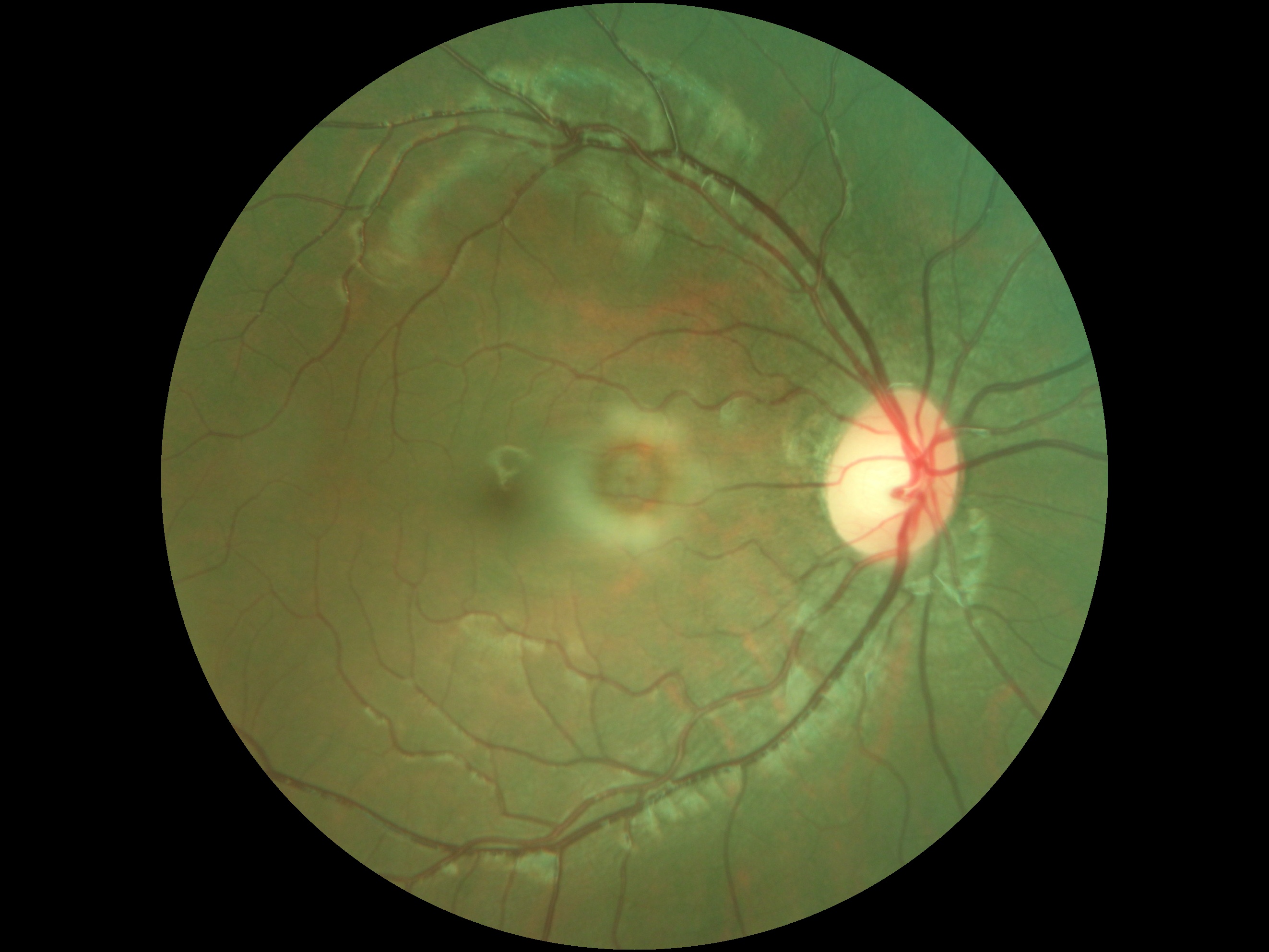

Supplement: S4 File — (ZIP) [file pone.0324352.s004.zip › Original fundus photographs (2)/Subject 120/OD_20230611578117_20230614100508_3.jpg]

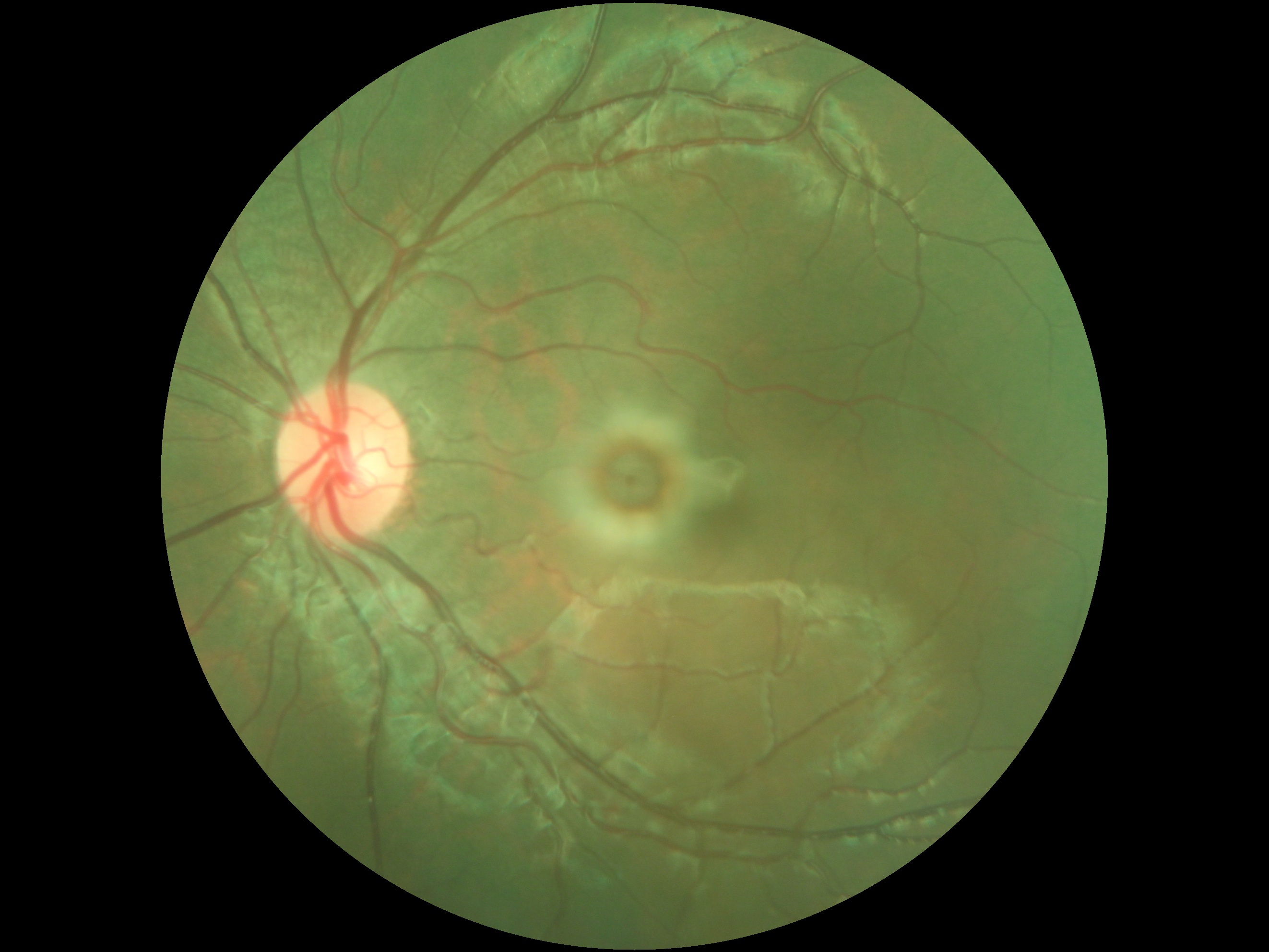

Supplement: S4 File — (ZIP) [file pone.0324352.s004.zip › Original fundus photographs (2)/Subject 120/OS_20230611578117_20230614100450_2.jpg]

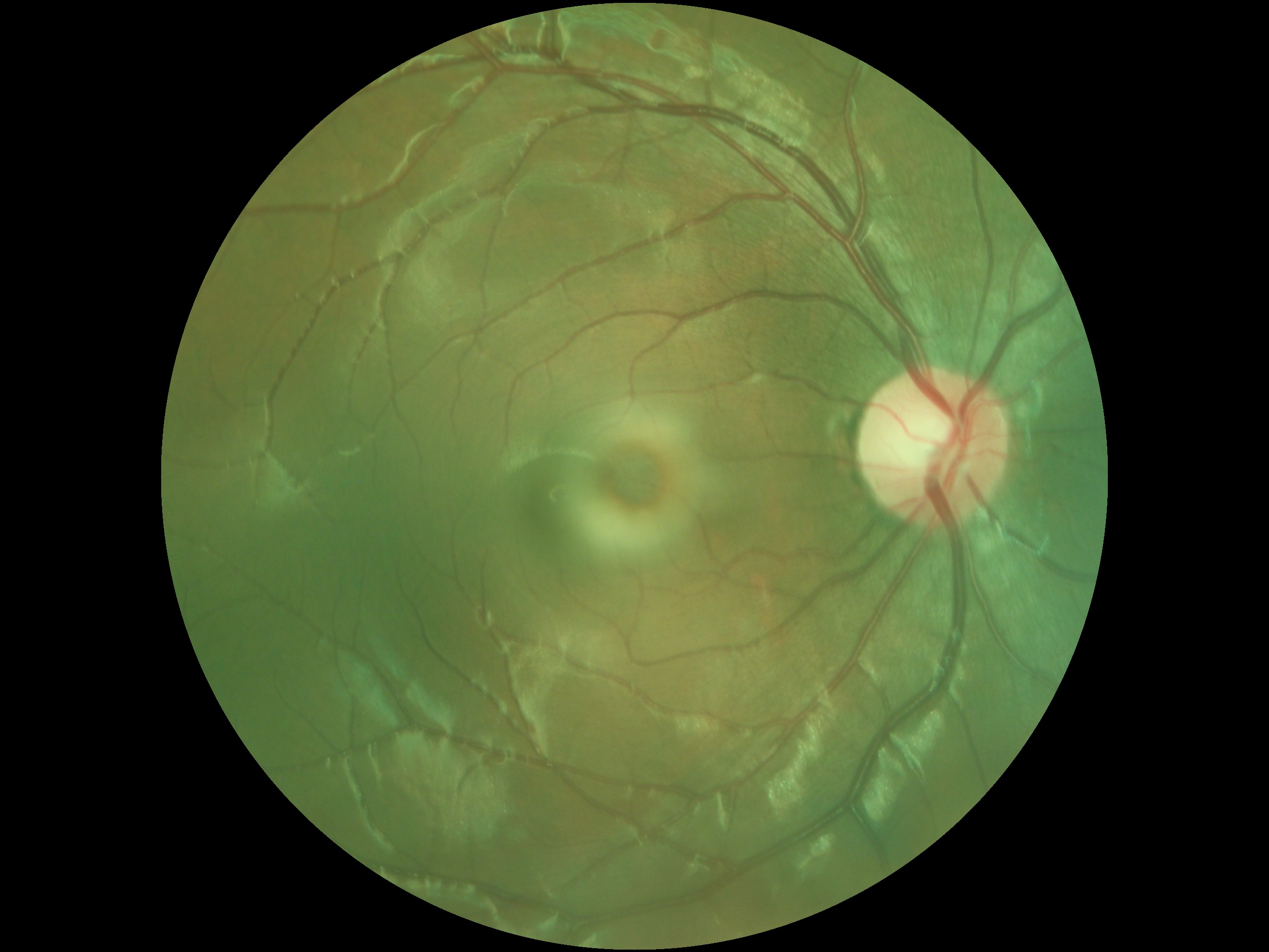

Supplement: S4 File — (ZIP) [file pone.0324352.s004.zip › Original fundus photographs (2)/Subject 121/OD_20230611825197_20230615113225_1.jpg]

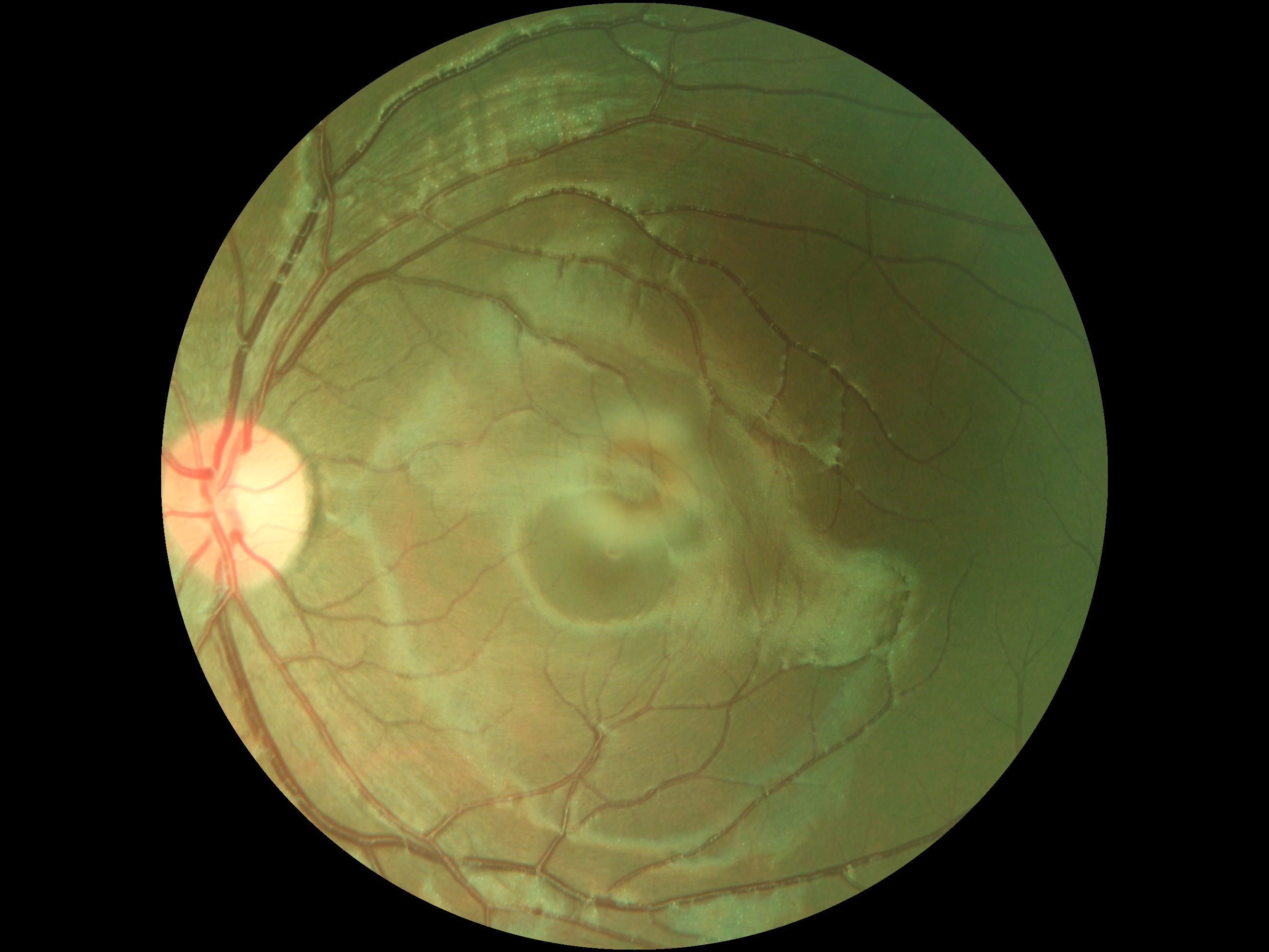

Supplement: S4 File — (ZIP) [file pone.0324352.s004.zip › Original fundus photographs (2)/Subject 121/OS_20230611825197_20230615114858_1.jpg]

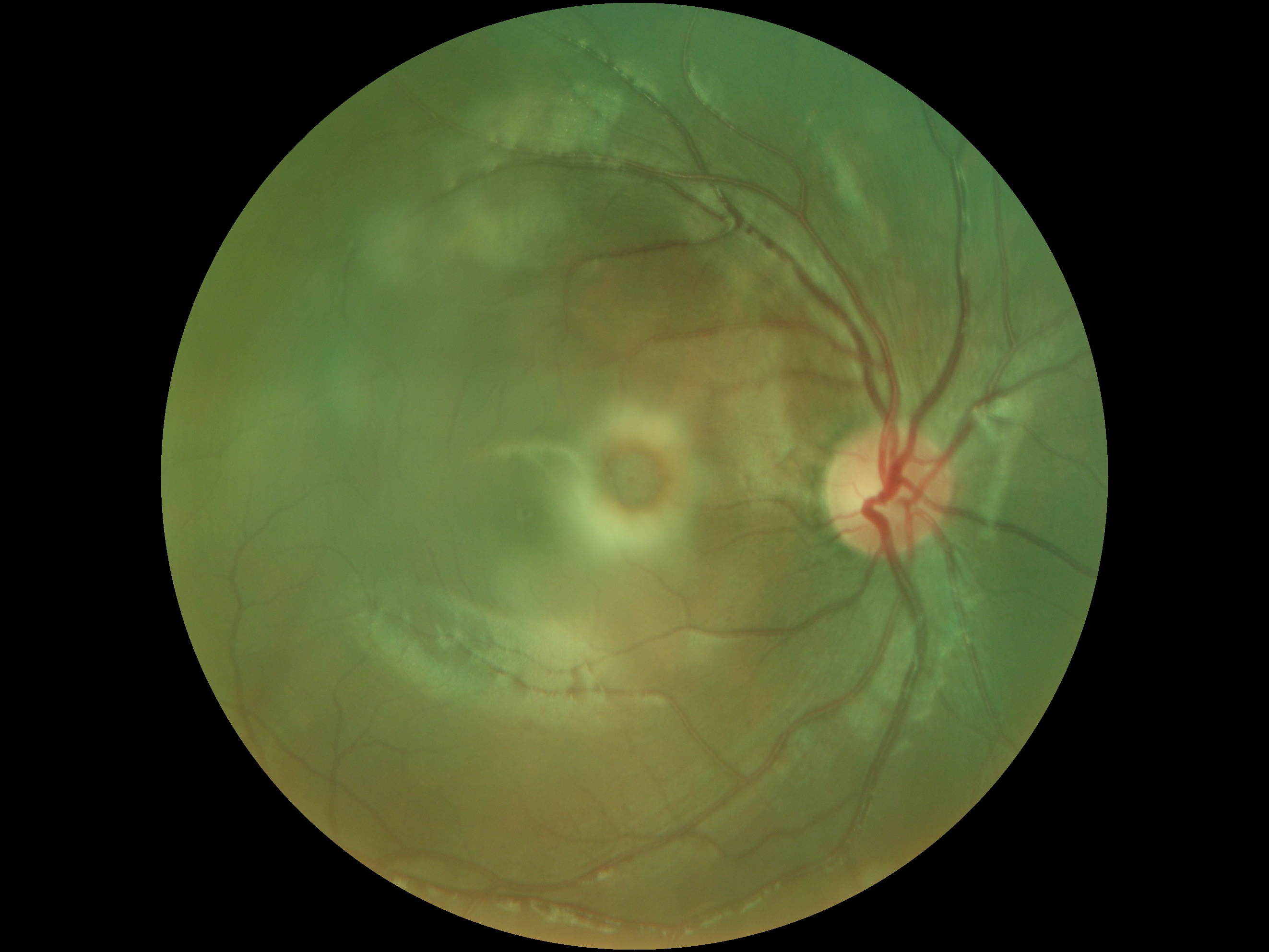

Supplement: S4 File — (ZIP) [file pone.0324352.s004.zip › Original fundus photographs (2)/Subject 122/OD_20230615299063_20230615153702_3.jpg]

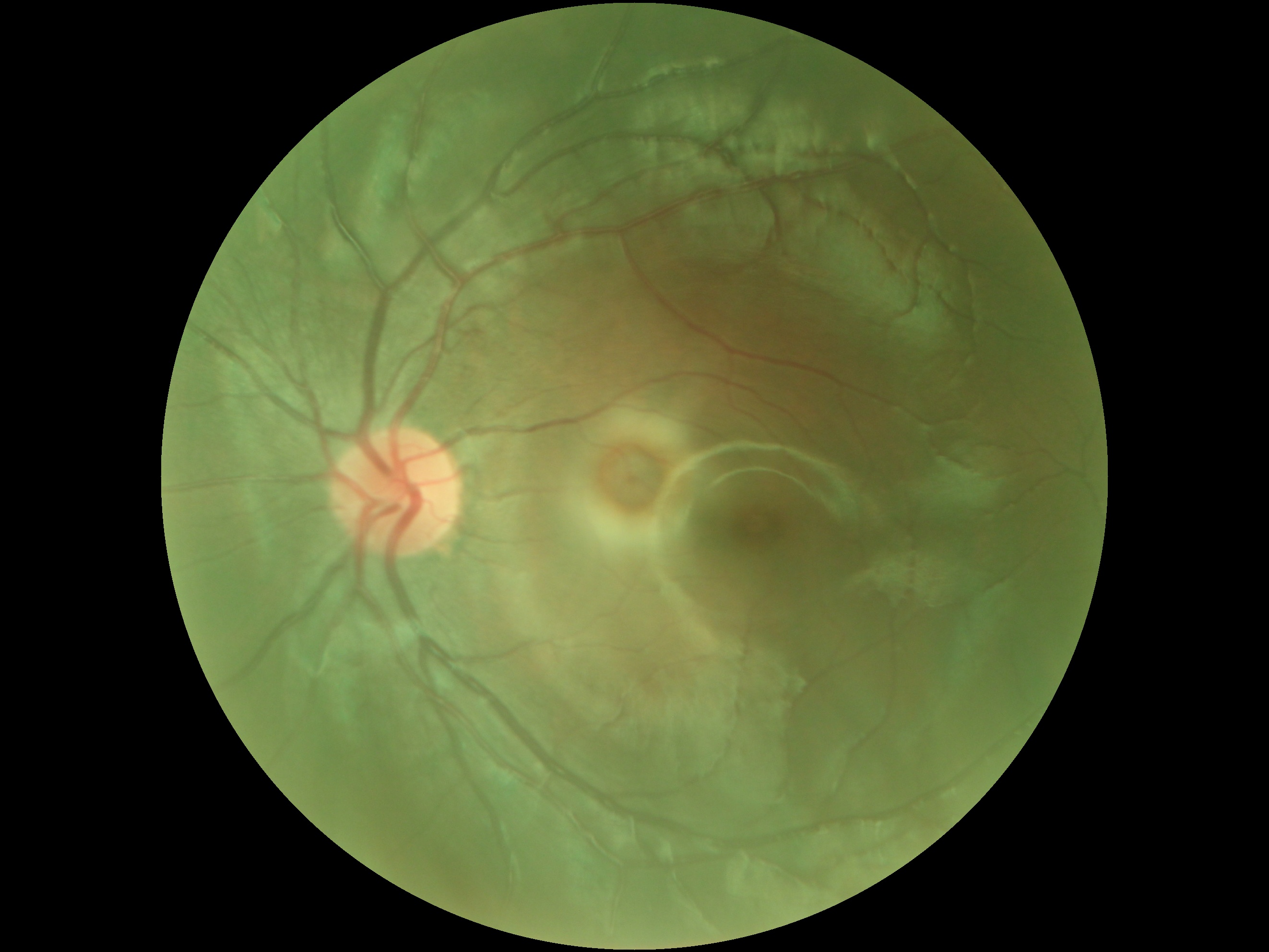

Supplement: S4 File — (ZIP) [file pone.0324352.s004.zip › Original fundus photographs (2)/Subject 122/OS_20230615299063_20230615153639_2.jpg]

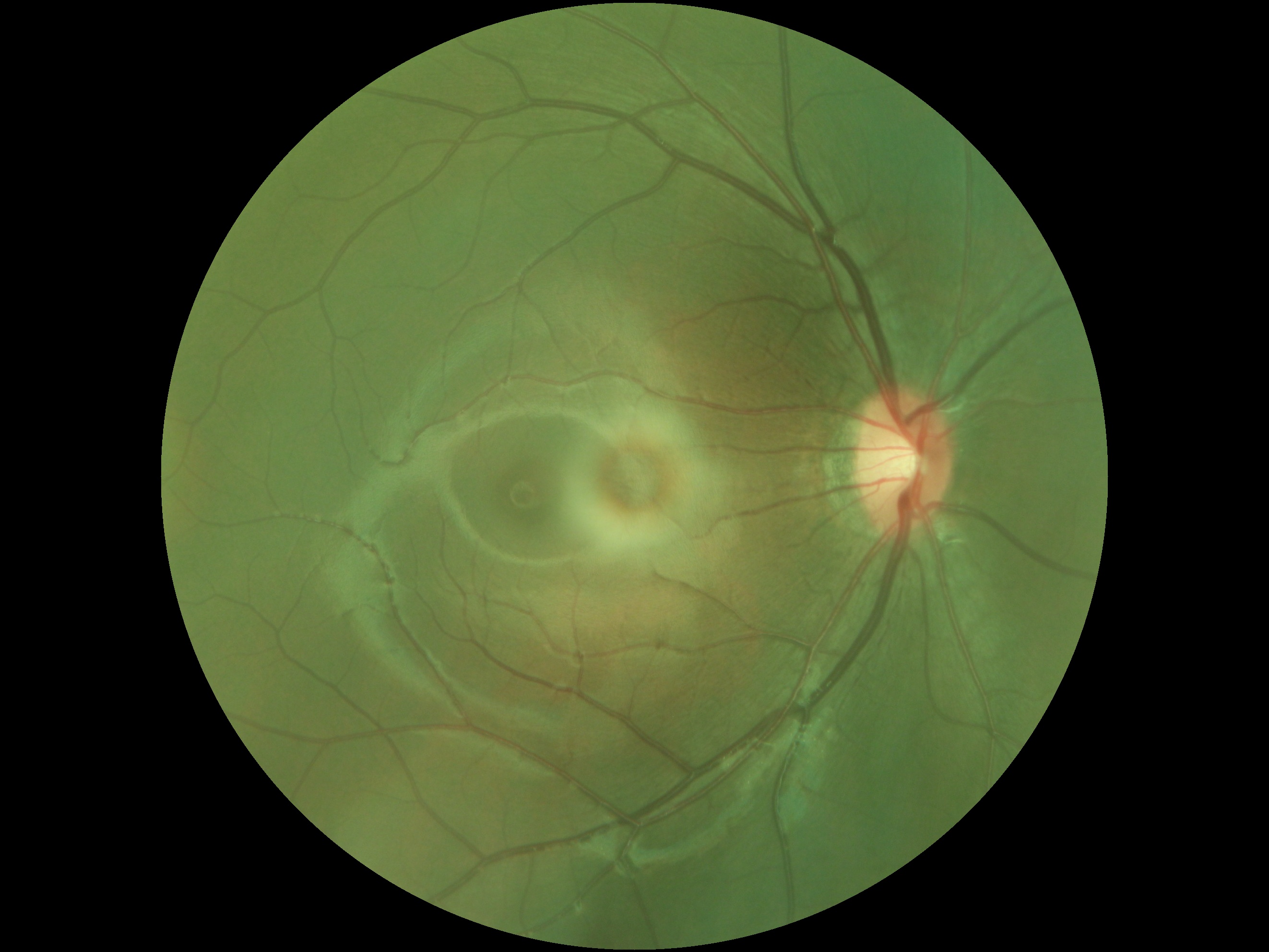

Supplement: S4 File — (ZIP) [file pone.0324352.s004.zip › Original fundus photographs (2)/Subject 123/OD_20230611289194_20230615113849_2.jpg]

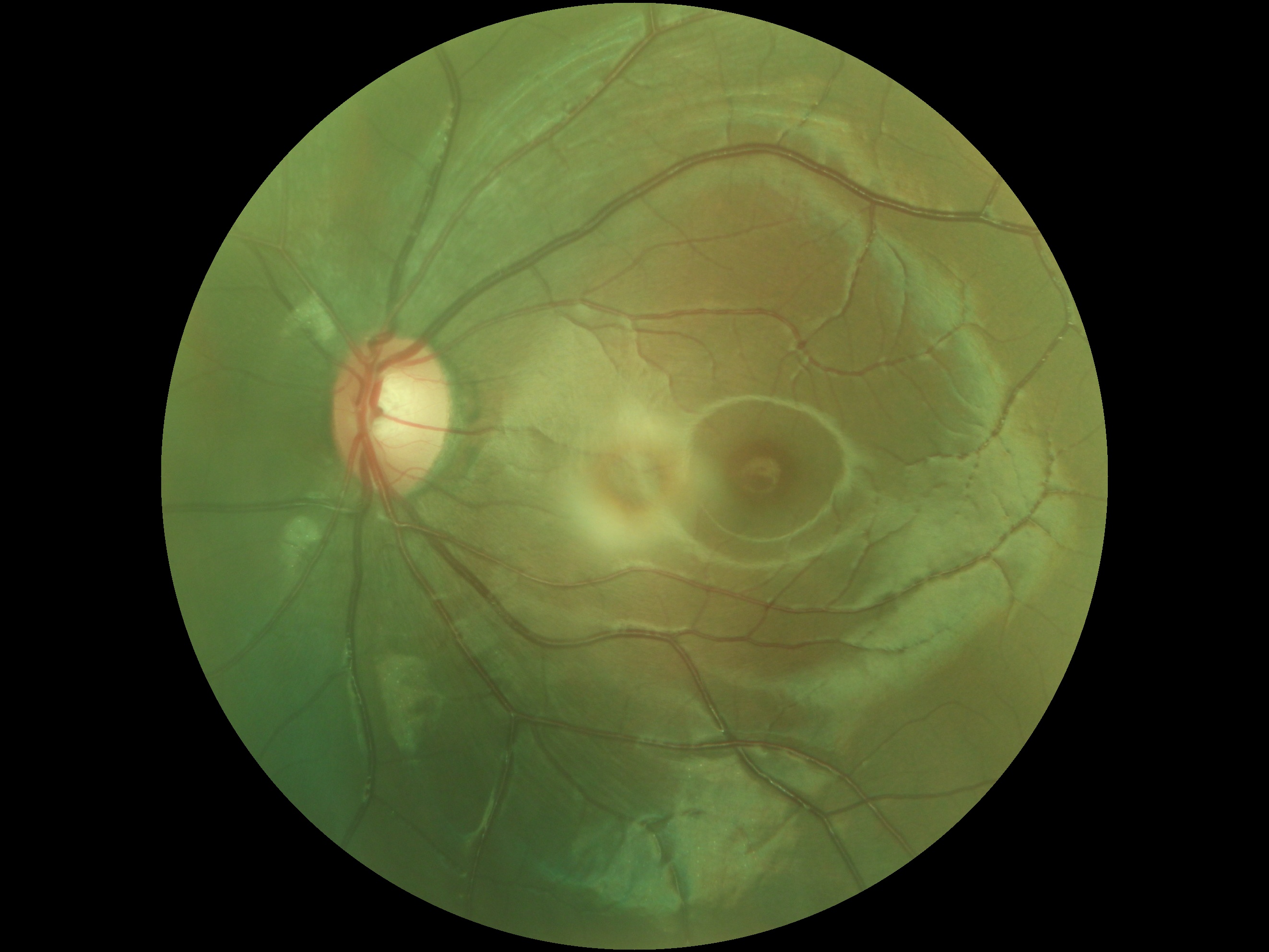

Supplement: S4 File — (ZIP) [file pone.0324352.s004.zip › Original fundus photographs (2)/Subject 123/OS_20230611289194_20230615113820_1.jpg]

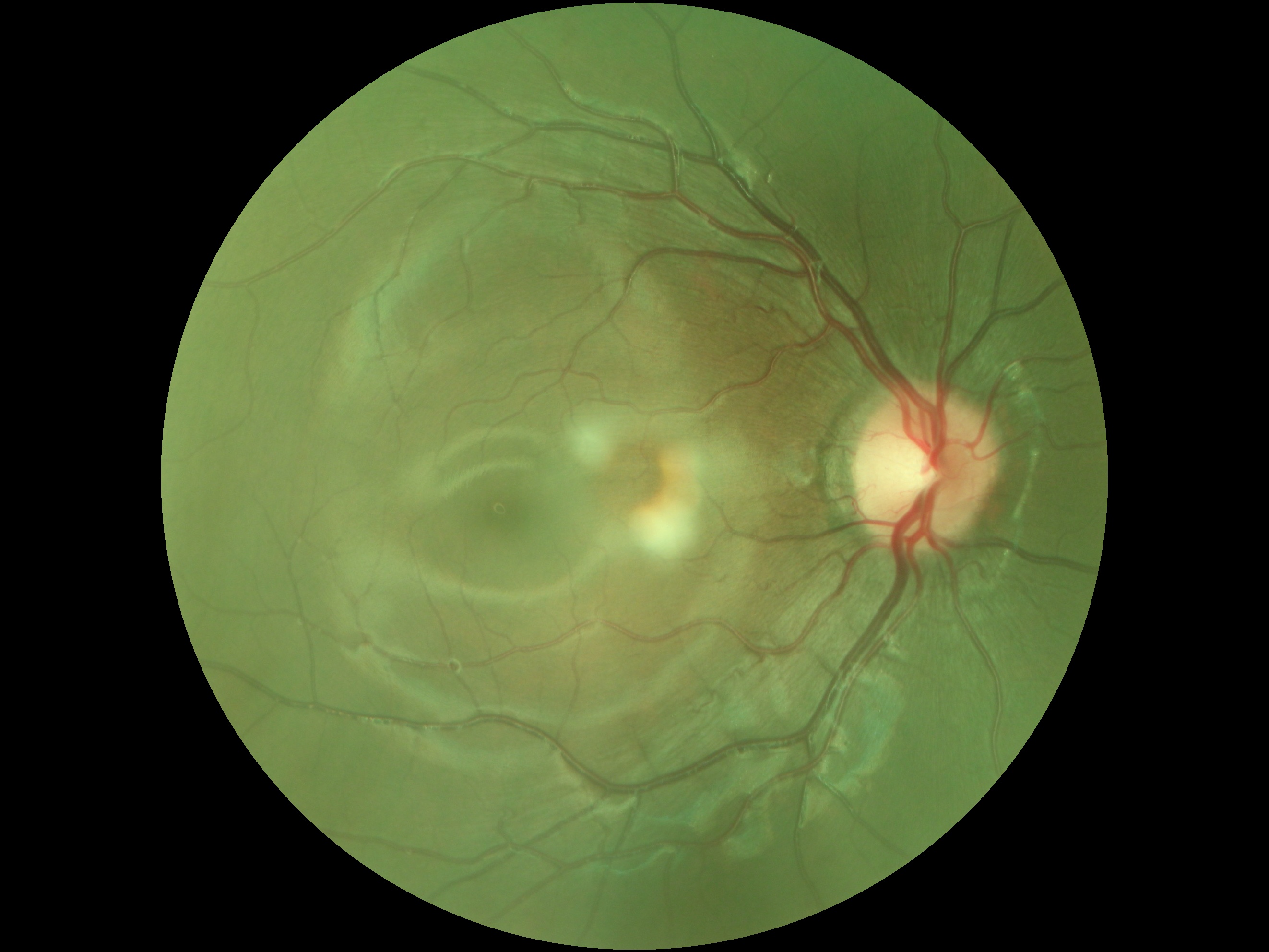

Supplement: S4 File — (ZIP) [file pone.0324352.s004.zip › Original fundus photographs (2)/Subject 124/OD_20230615692097_20230615170225_1.jpg]

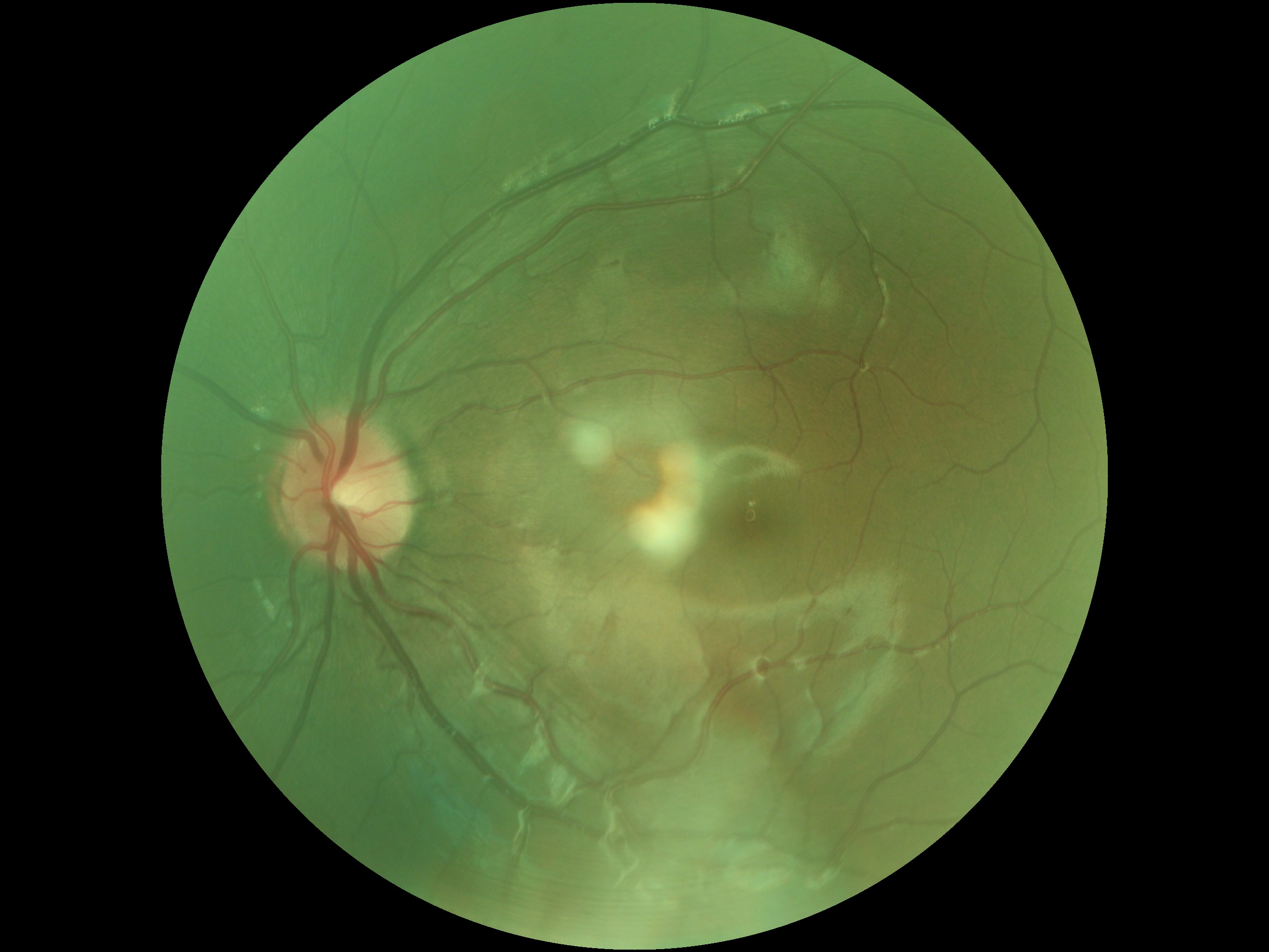

Supplement: S4 File — (ZIP) [file pone.0324352.s004.zip › Original fundus photographs (2)/Subject 124/OS_20230615692097_20230615170239_2.jpg]

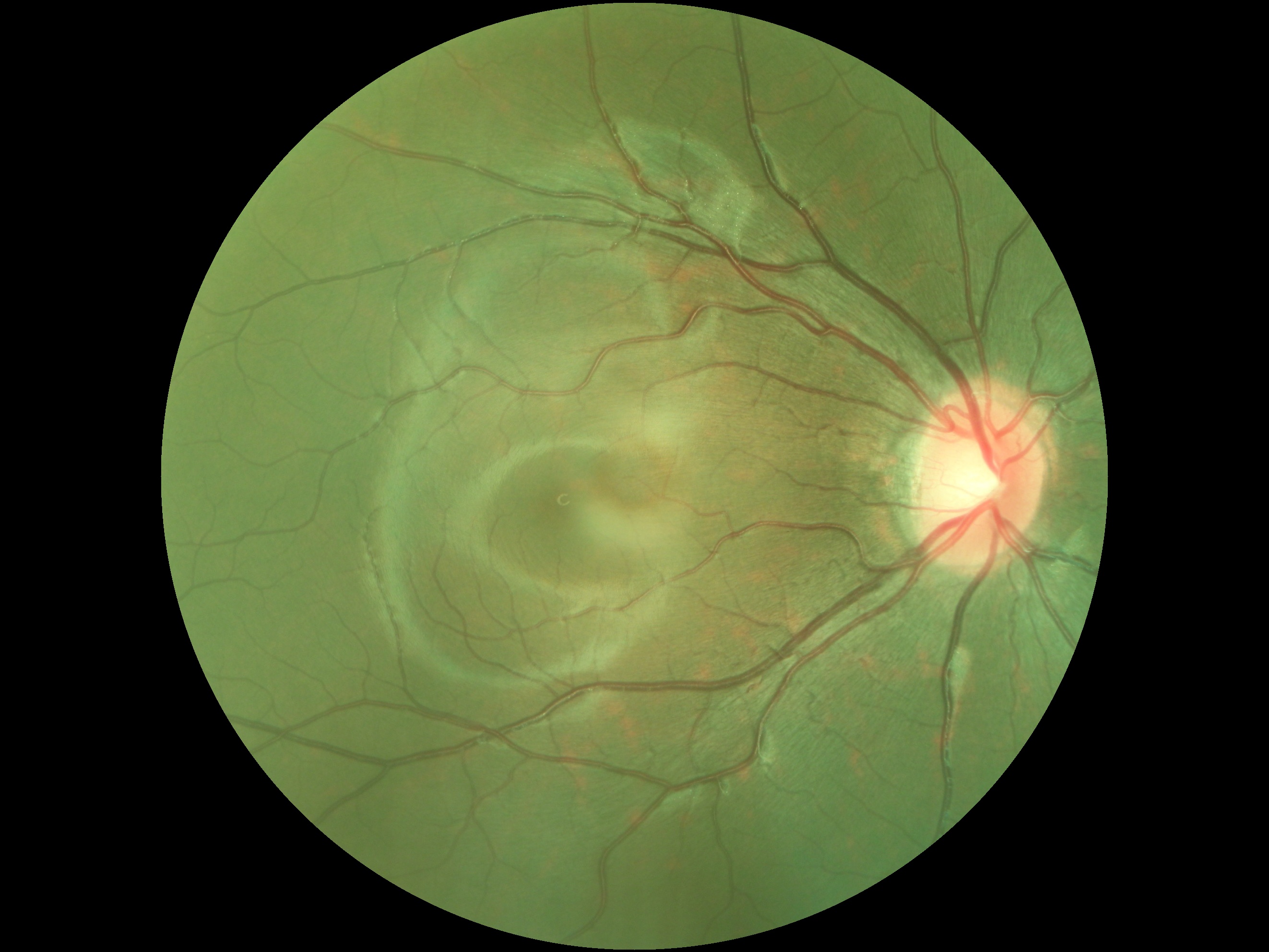

Supplement: S4 File — (ZIP) [file pone.0324352.s004.zip › Original fundus photographs (2)/Subject 63/OD_20230615564084_20230615160038_2.jpg]

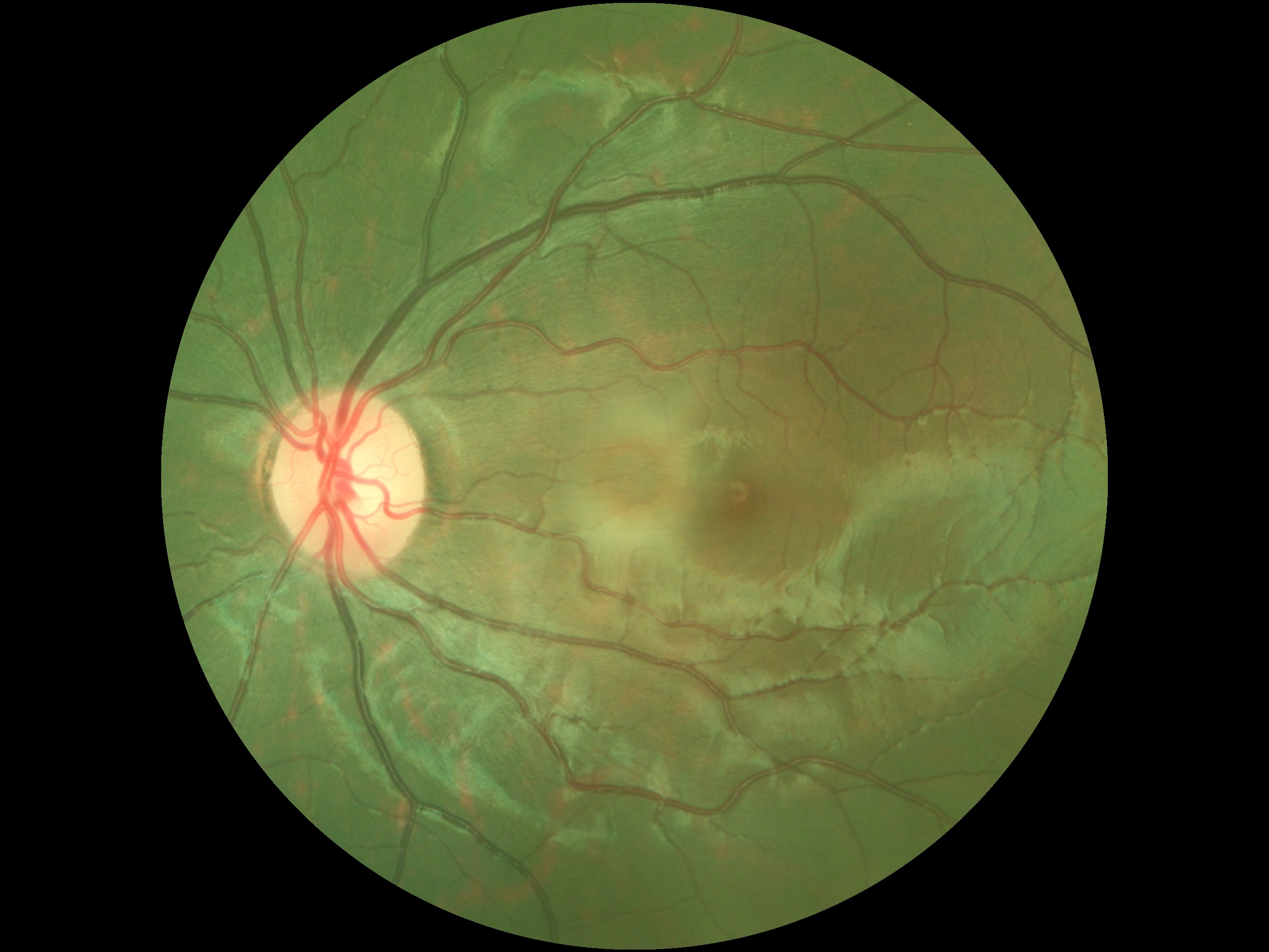

Supplement: S4 File — (ZIP) [file pone.0324352.s004.zip › Original fundus photographs (2)/Subject 63/OS_20230615564084_20230615160002_1.jpg]

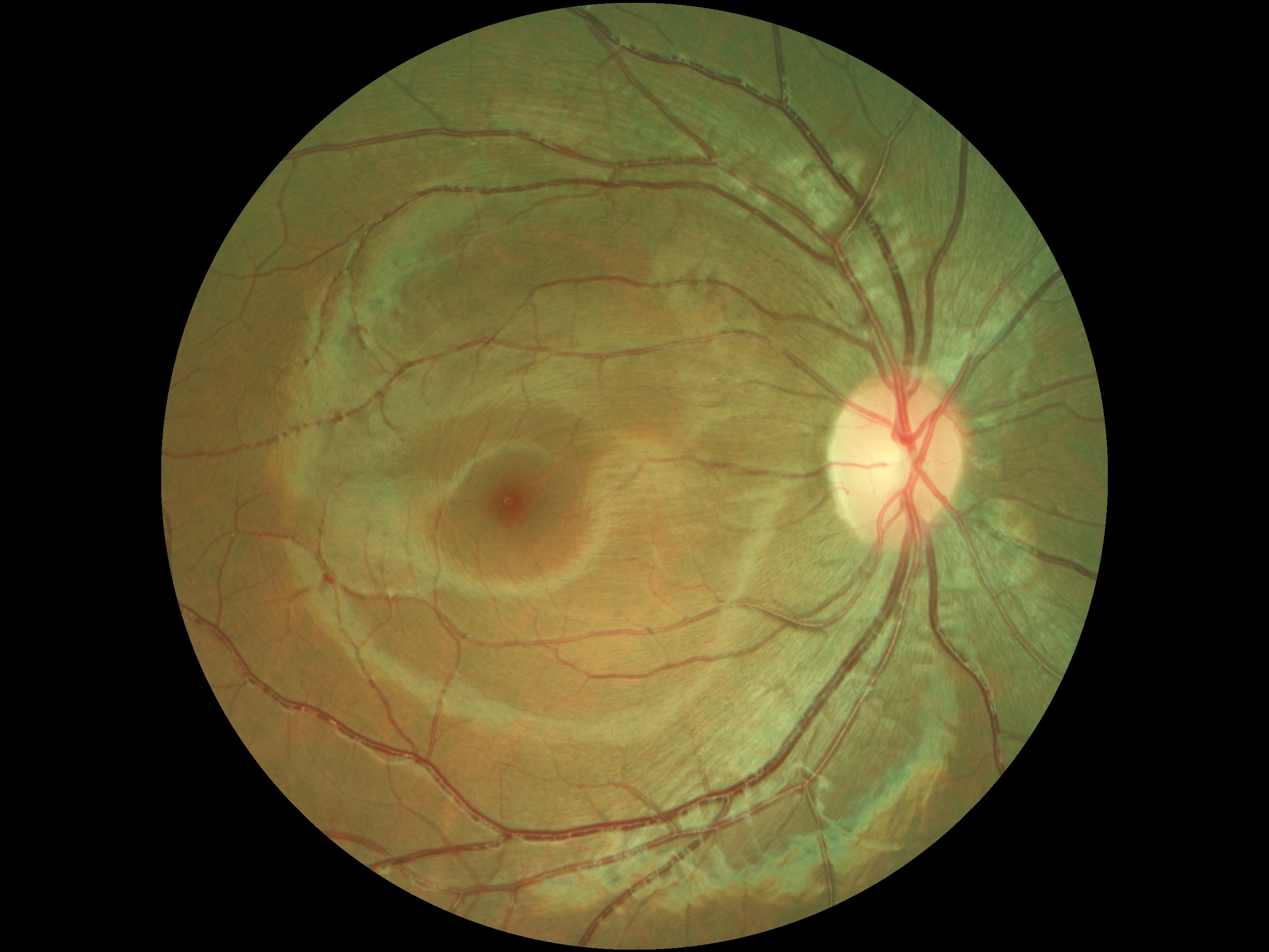

Supplement: S4 File — (ZIP) [file pone.0324352.s004.zip › Original fundus photographs (2)/Subject 64/OD_20230611045075_20230612163526_1.jpg]

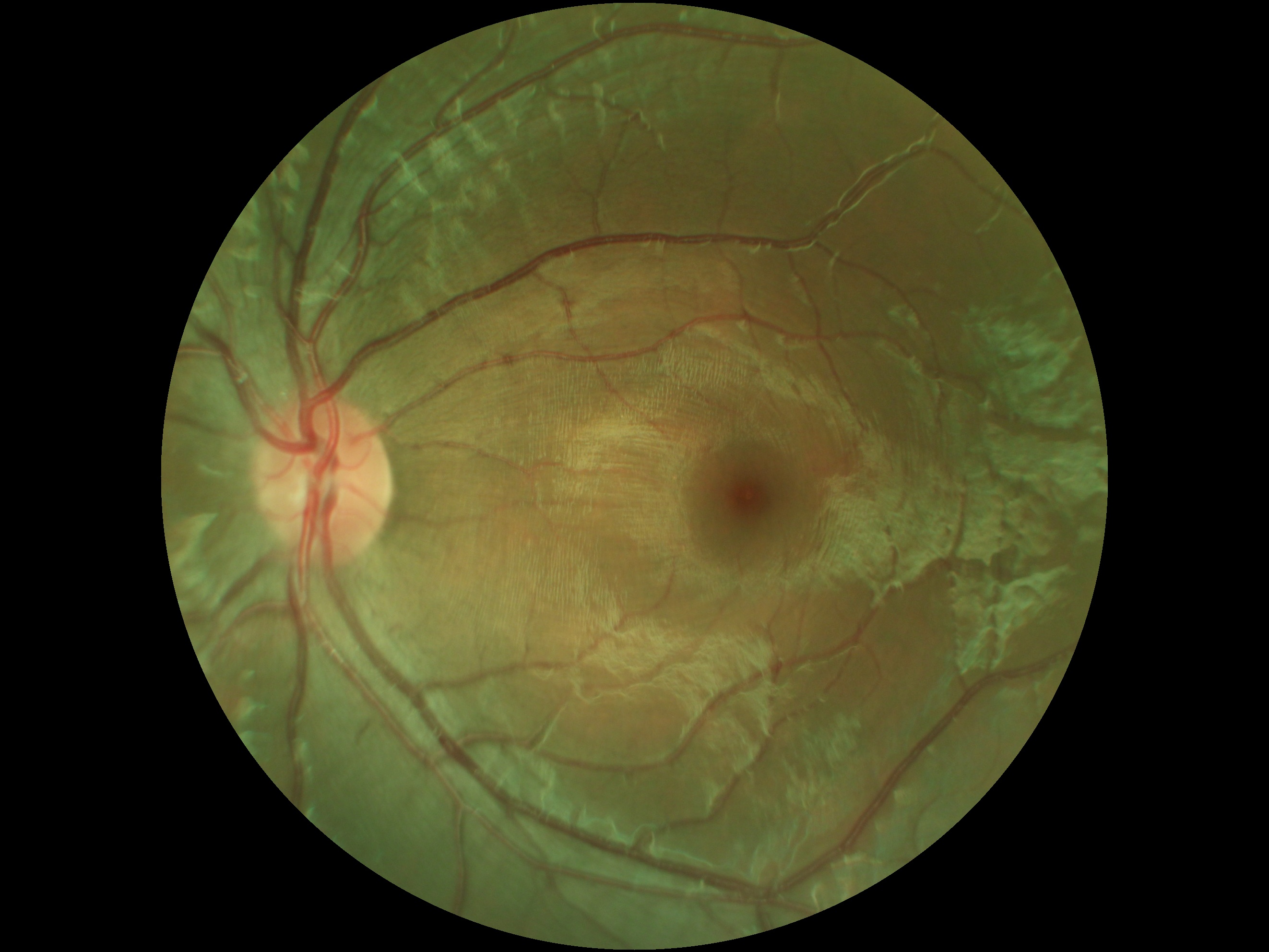

Supplement: S4 File — (ZIP) [file pone.0324352.s004.zip › Original fundus photographs (2)/Subject 64/OS_20230611045075_20230612163543_2.jpg]

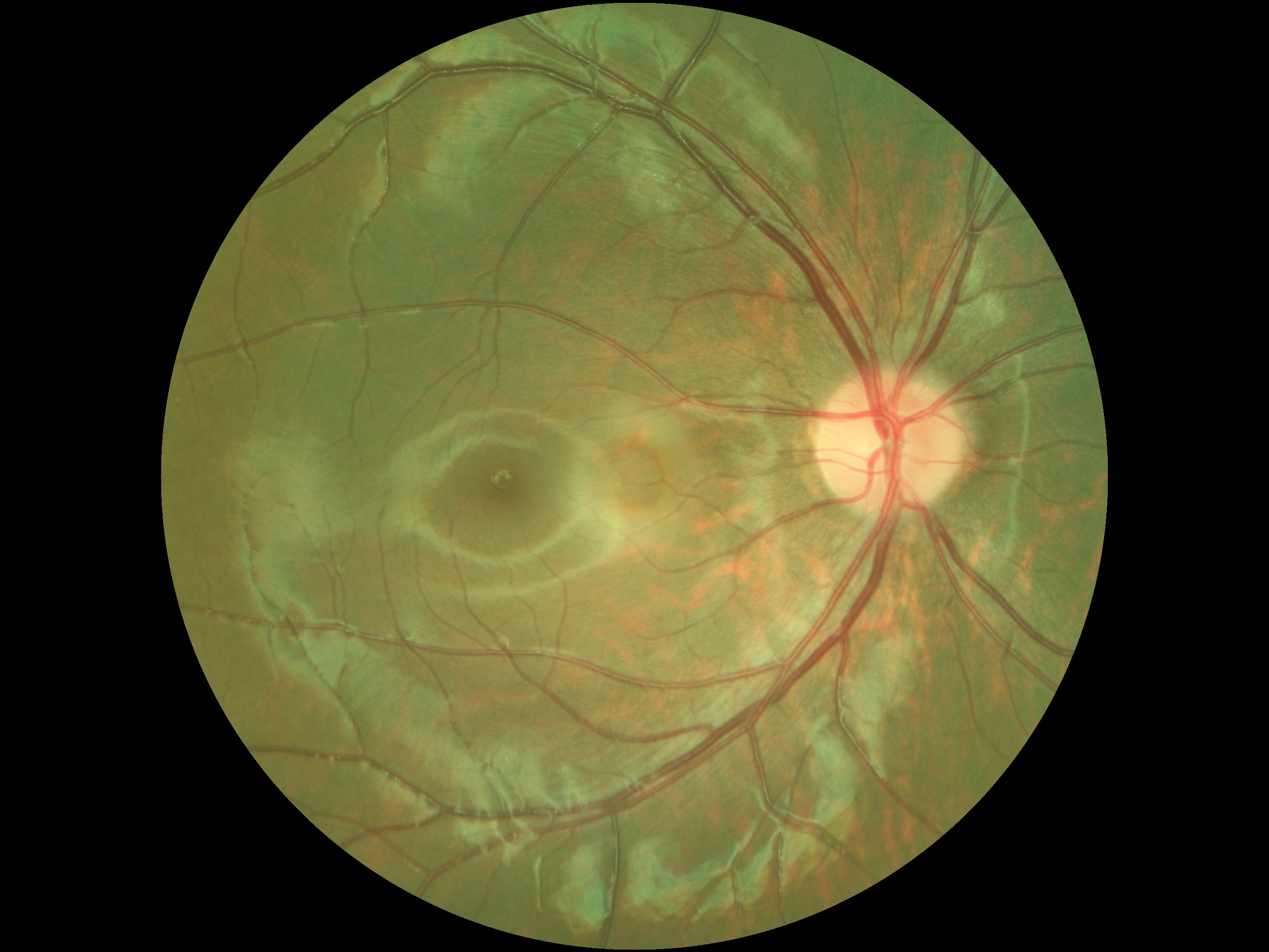

Supplement: S4 File — (ZIP) [file pone.0324352.s004.zip › Original fundus photographs (2)/Subject 65/OD_20230615294085_20230615155845_1.jpg]

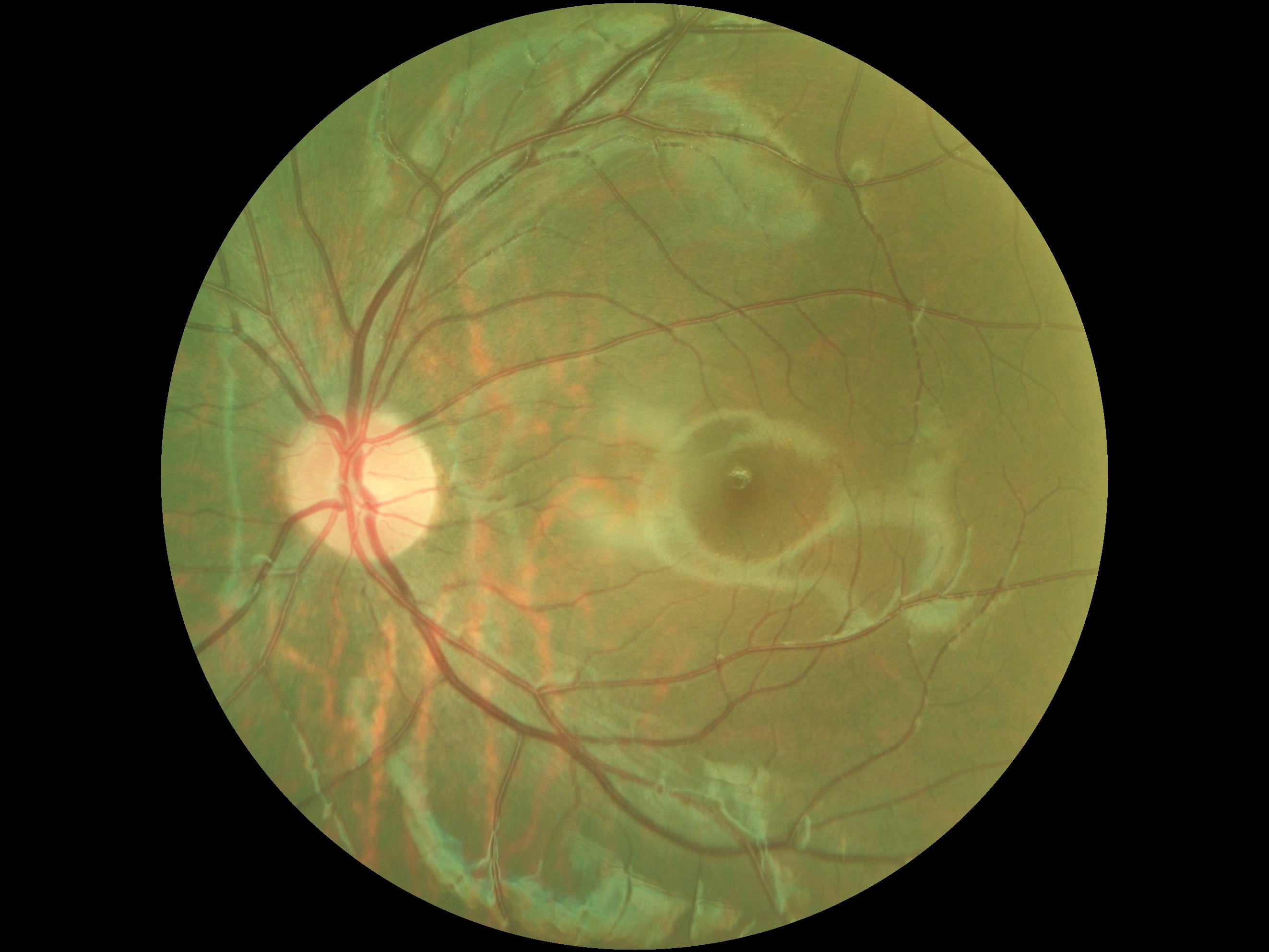

Supplement: S4 File — (ZIP) [file pone.0324352.s004.zip › Original fundus photographs (2)/Subject 65/OS_20230615294085_20230615155902_2.jpg]

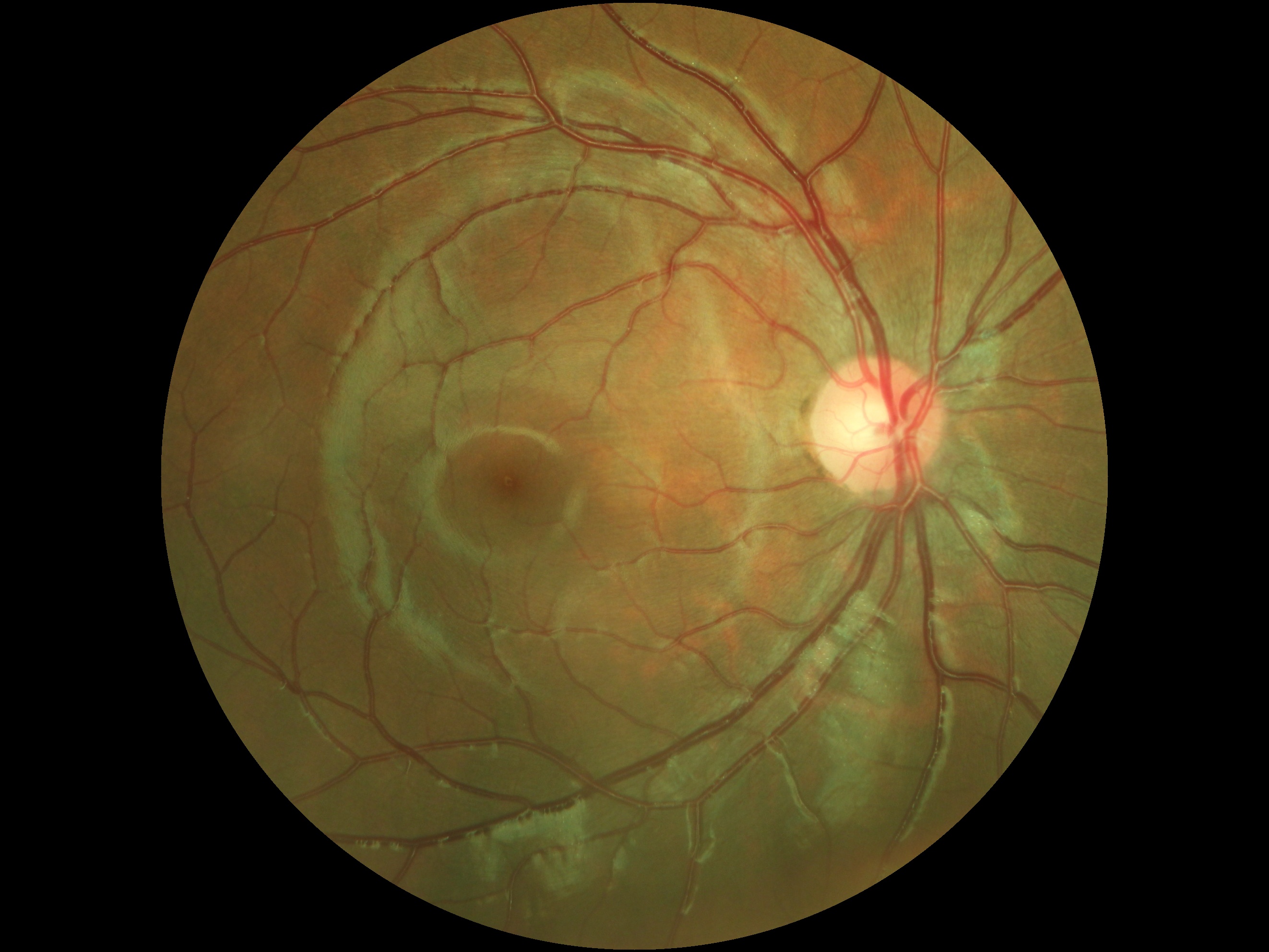

Supplement: S4 File — (ZIP) [file pone.0324352.s004.zip › Original fundus photographs (2)/Subject 66/OD_20230611913022_20230612155522_1.jpg]

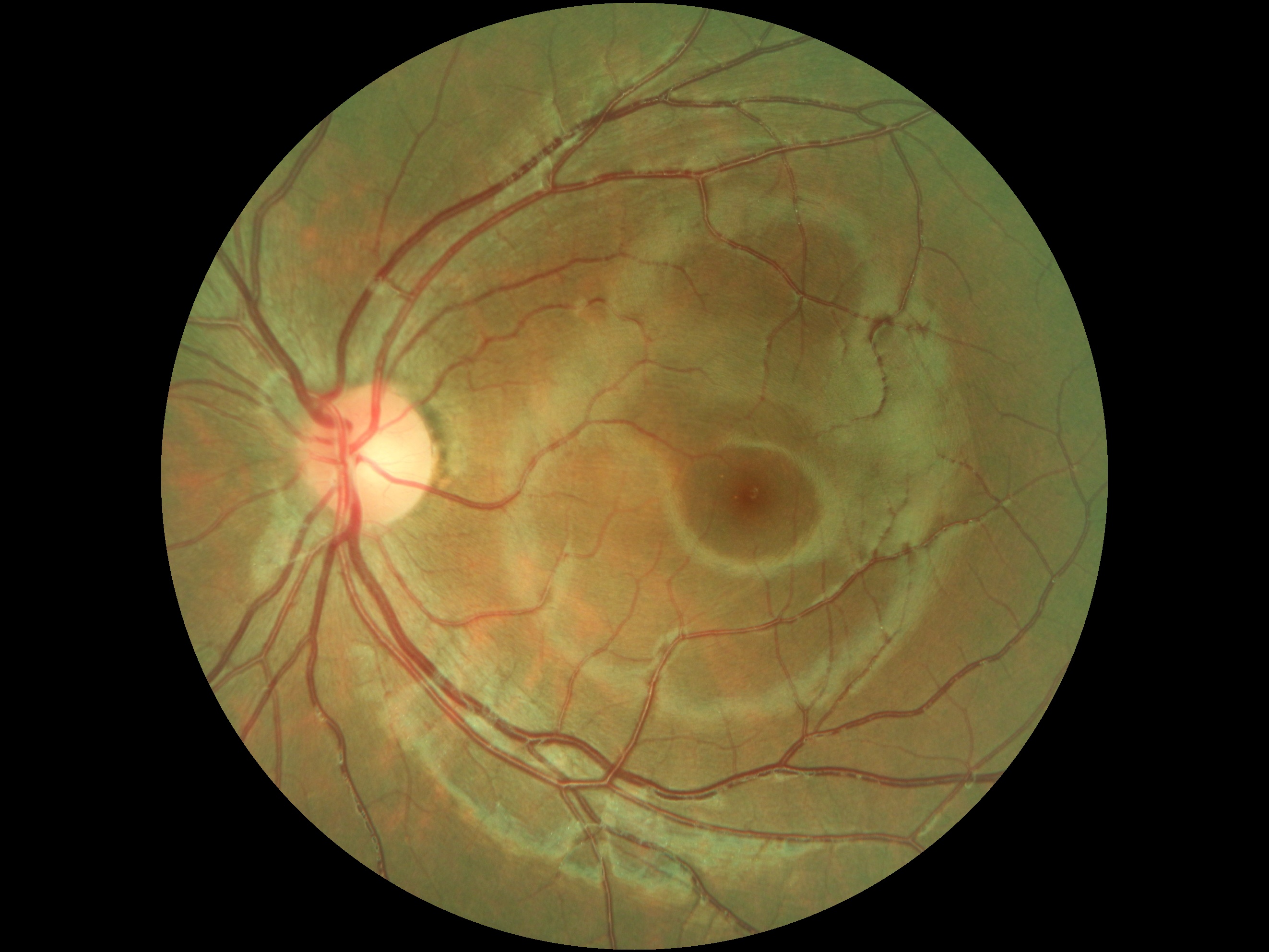

Supplement: S4 File — (ZIP) [file pone.0324352.s004.zip › Original fundus photographs (2)/Subject 66/OS_20230611913022_20230612155554_2.jpg]

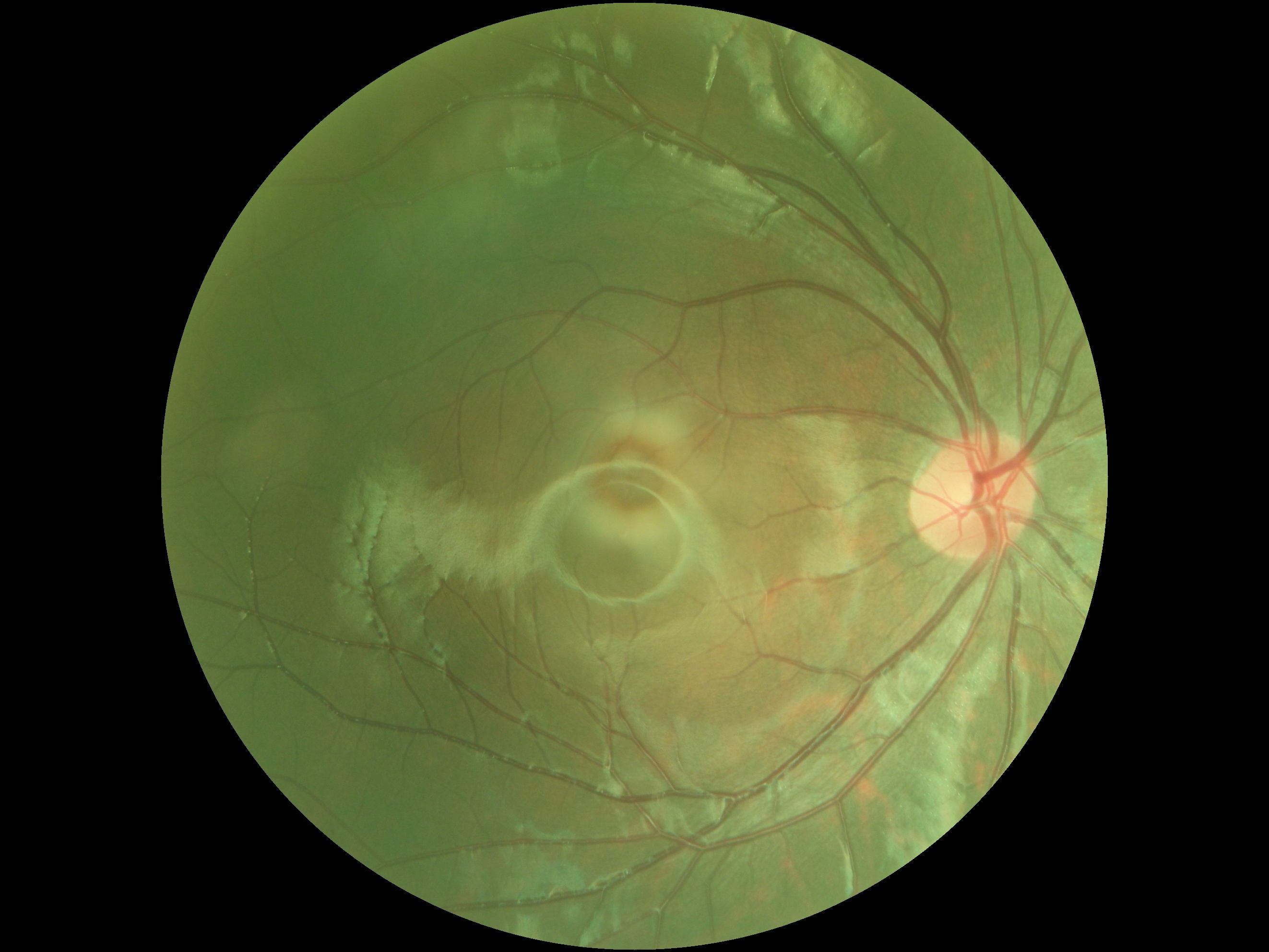

Supplement: S4 File — (ZIP) [file pone.0324352.s004.zip › Original fundus photographs (2)/Subject 67/OD_20230611963271_20230615103831_2.jpg]

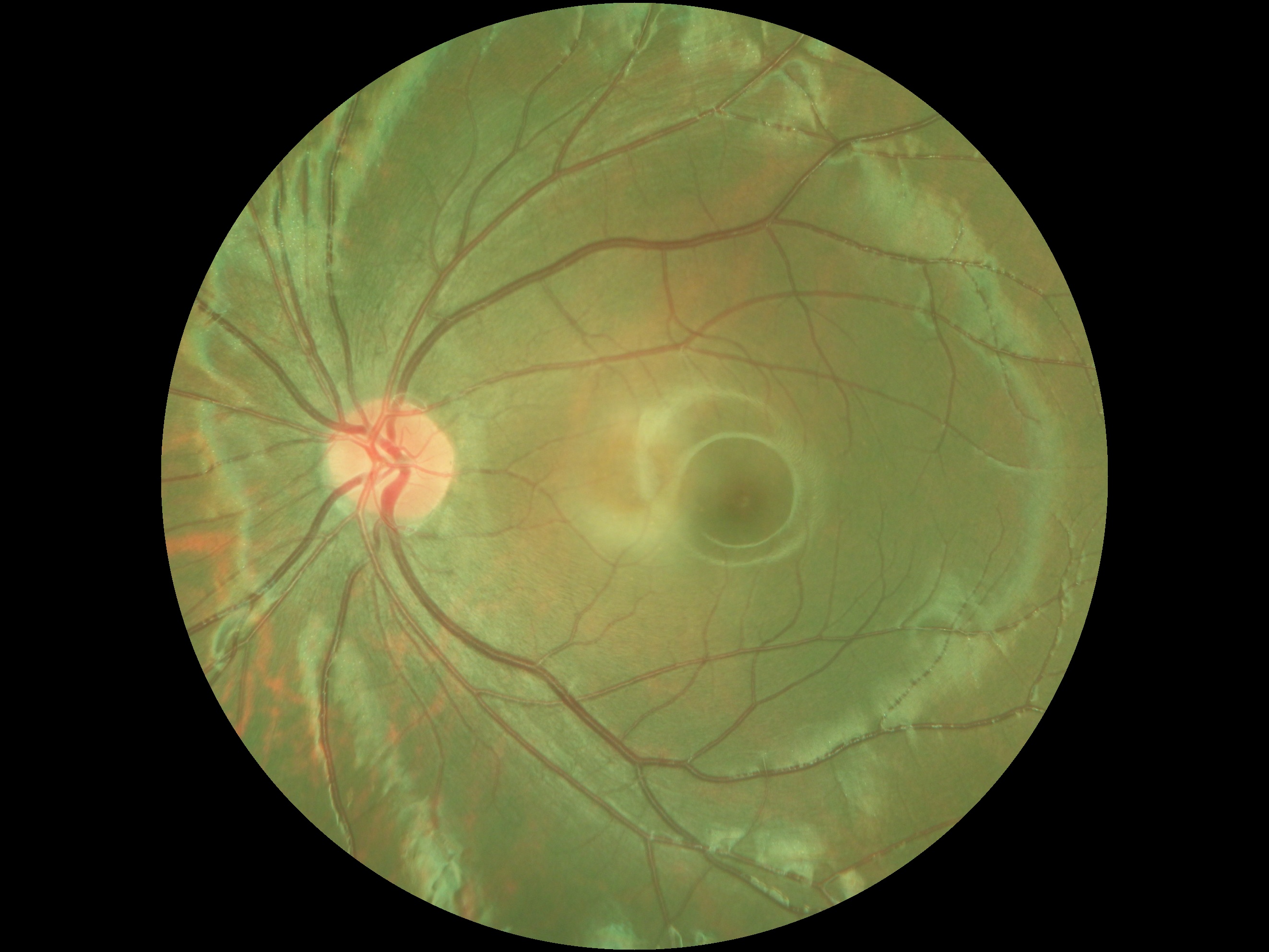

Supplement: S4 File — (ZIP) [file pone.0324352.s004.zip › Original fundus photographs (2)/Subject 67/OS_20230611963271_20230615103454_1.jpg]

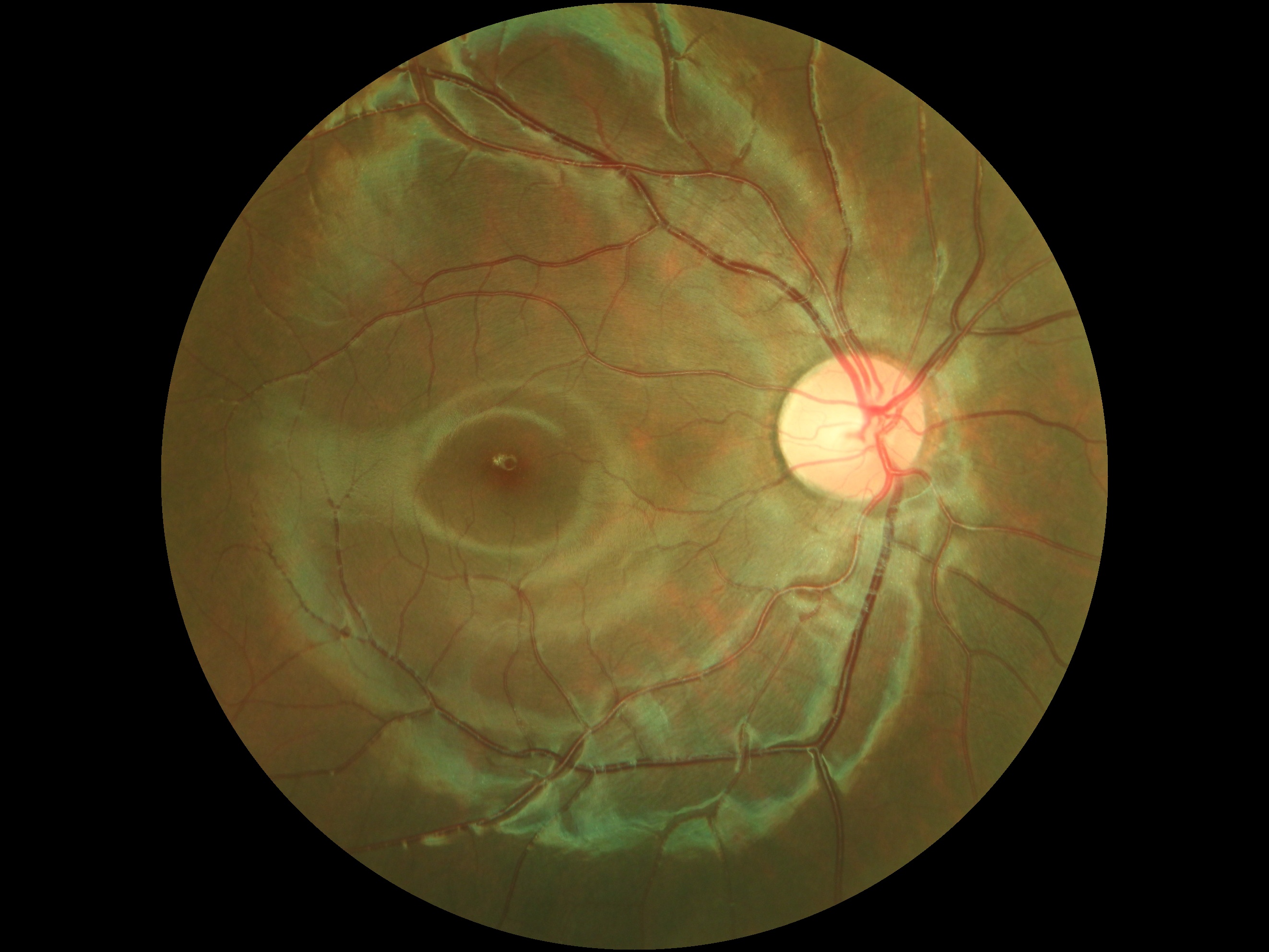

Supplement: S4 File — (ZIP) [file pone.0324352.s004.zip › Original fundus photographs (2)/Subject 68/OD_20230611902090_20230612155808_1.jpg]

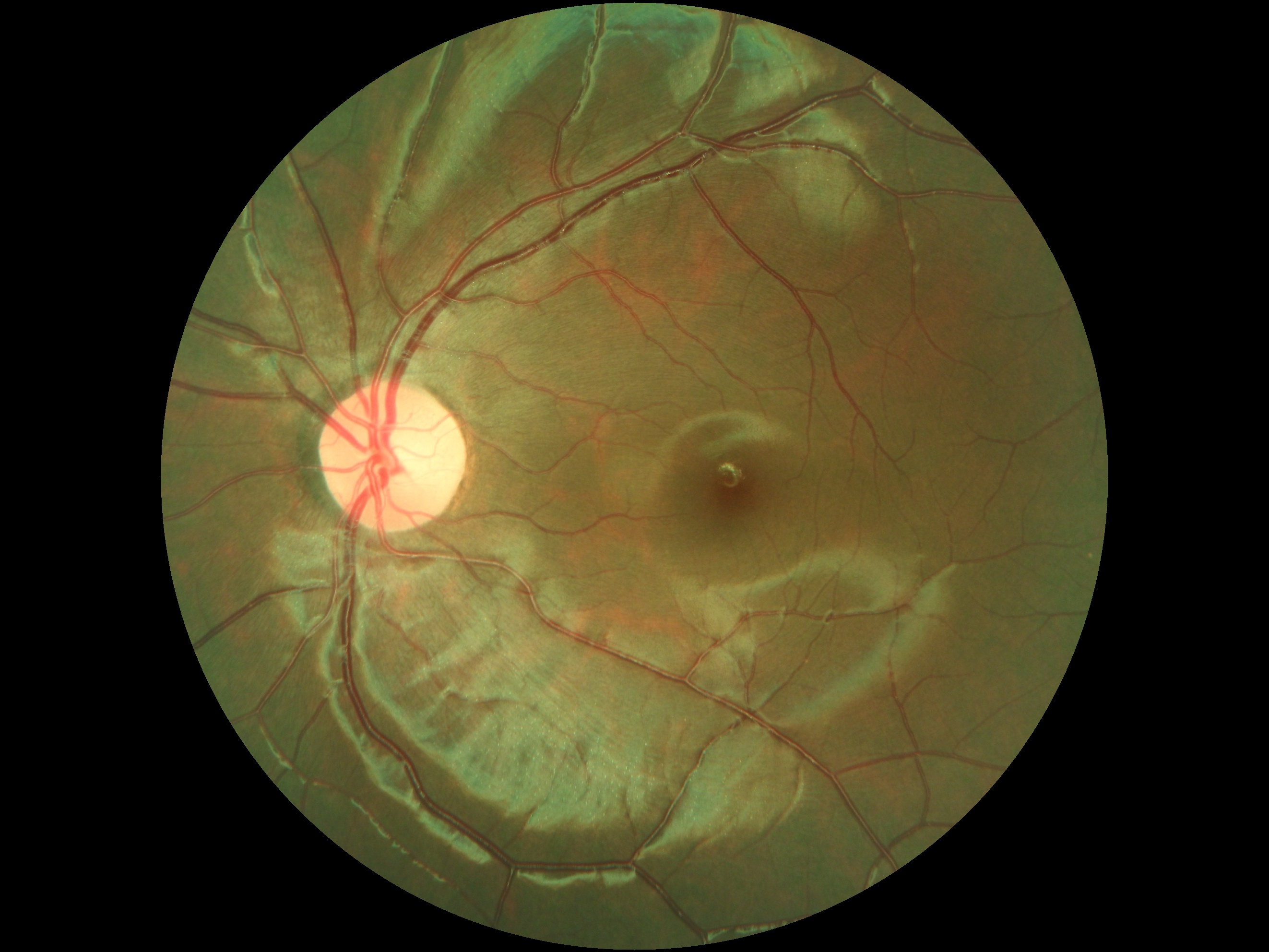

Supplement: S4 File — (ZIP) [file pone.0324352.s004.zip › Original fundus photographs (2)/Subject 68/OS_20230611902090_20230612155832_2.jpg]

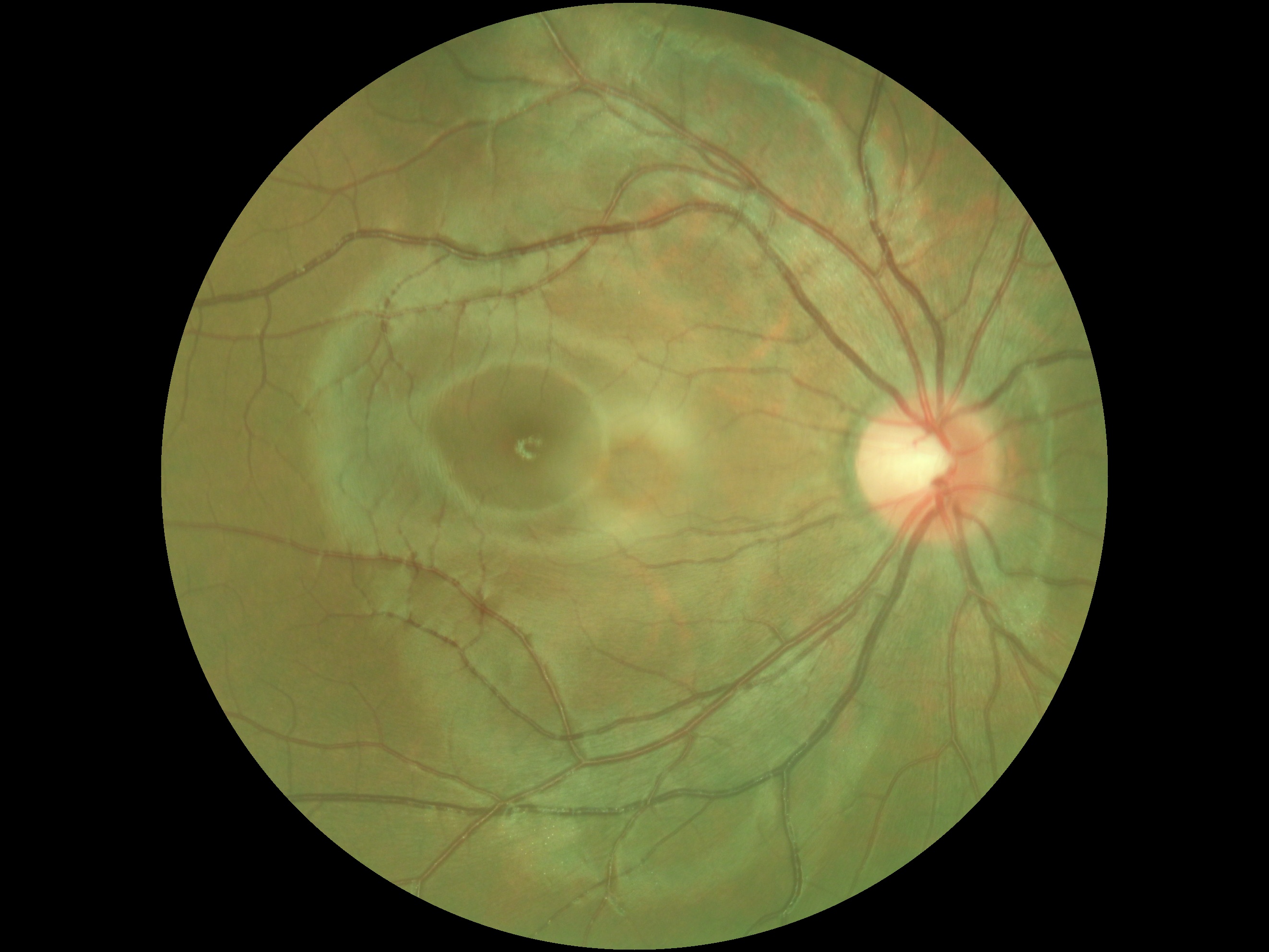

Supplement: S4 File — (ZIP) [file pone.0324352.s004.zip › Original fundus photographs (2)/Subject 69/OD_20230615342012_20230615105147_1.jpg]

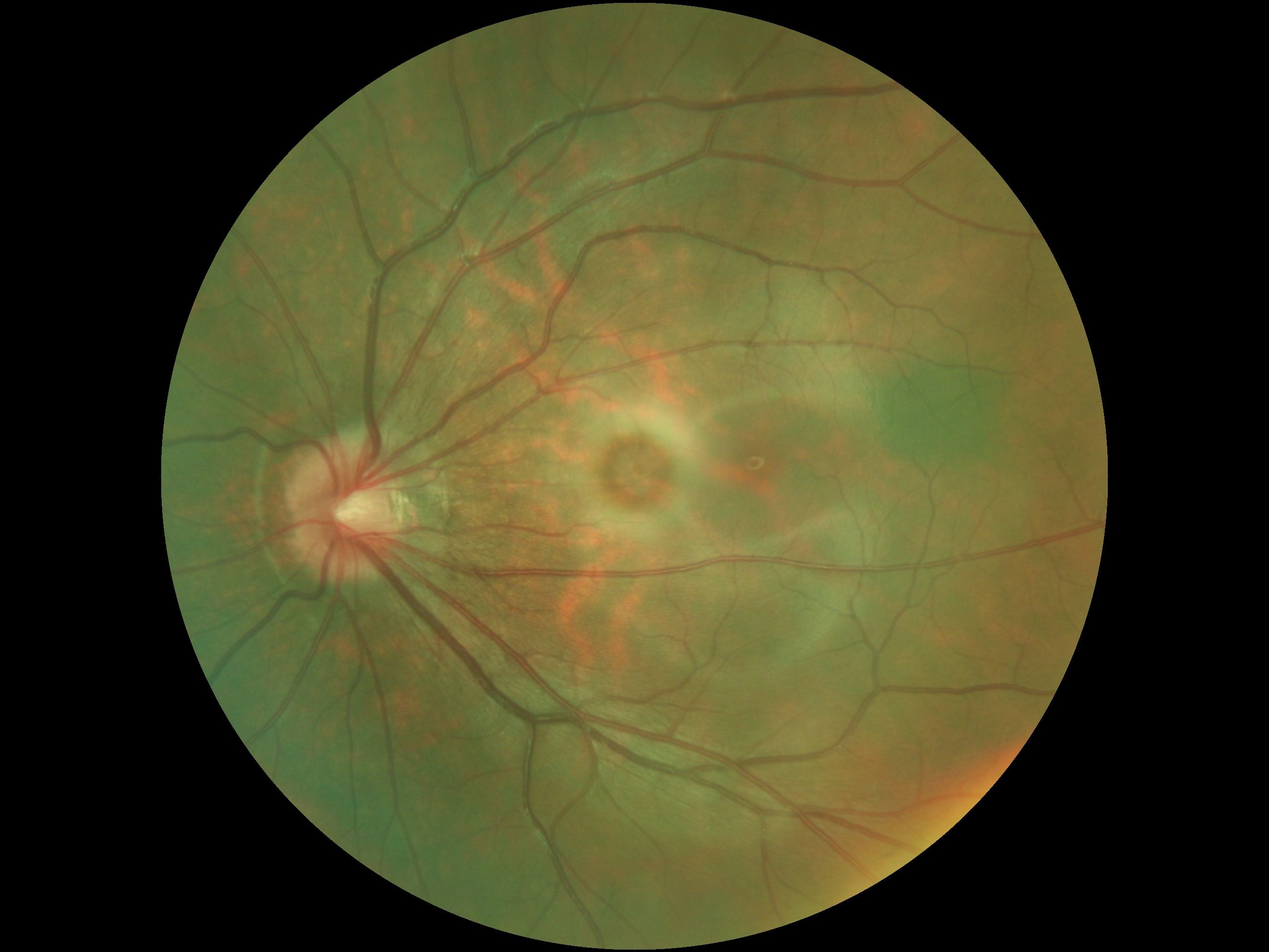

Supplement: S4 File — (ZIP) [file pone.0324352.s004.zip › Original fundus photographs (2)/Subject 69/OS_20230615342012_20230615105205_2.jpg]

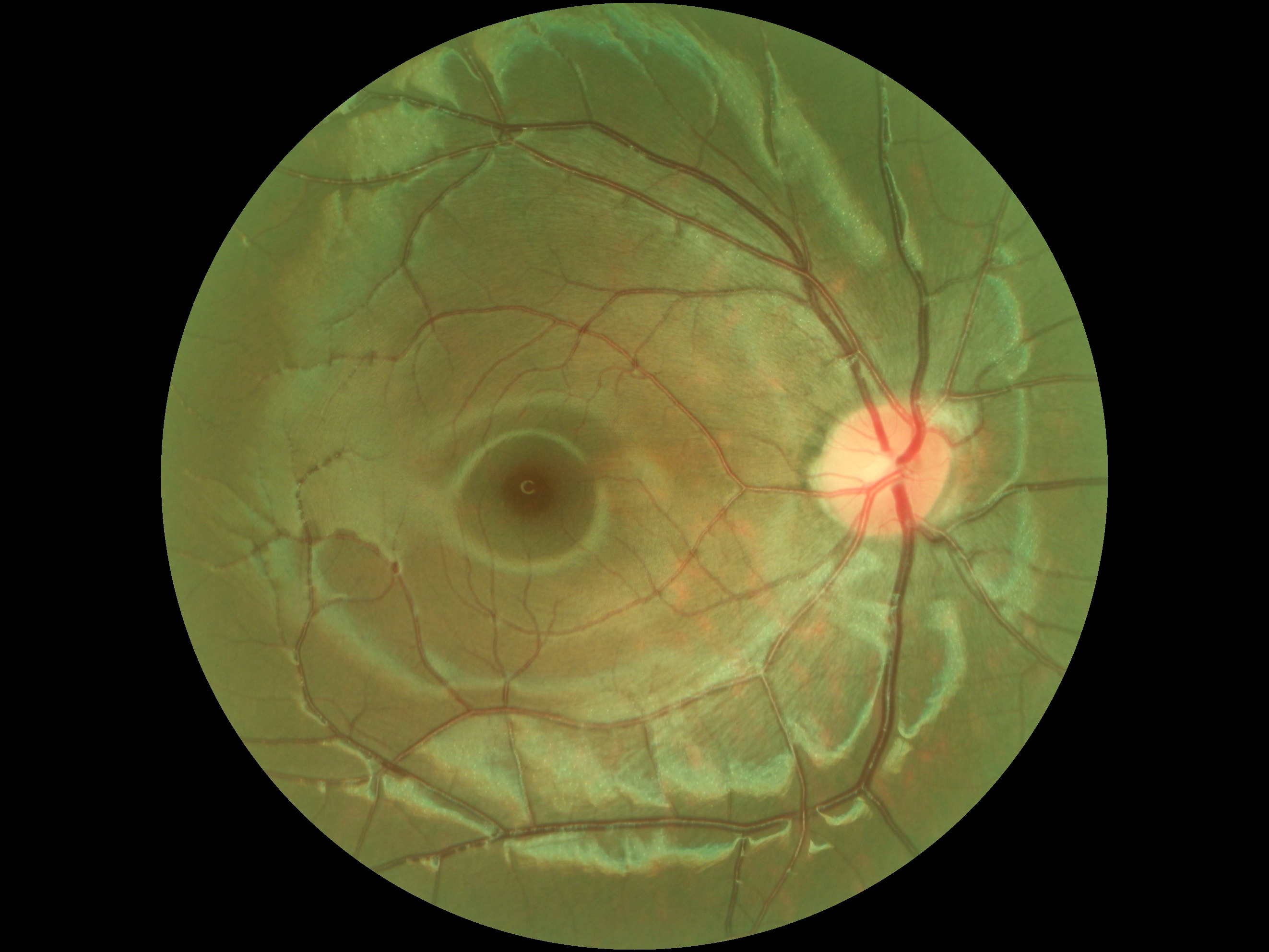

Supplement: S4 File — (ZIP) [file pone.0324352.s004.zip › Original fundus photographs (2)/Subject 70/OD_20230611847069_20230612163748_1.jpg]

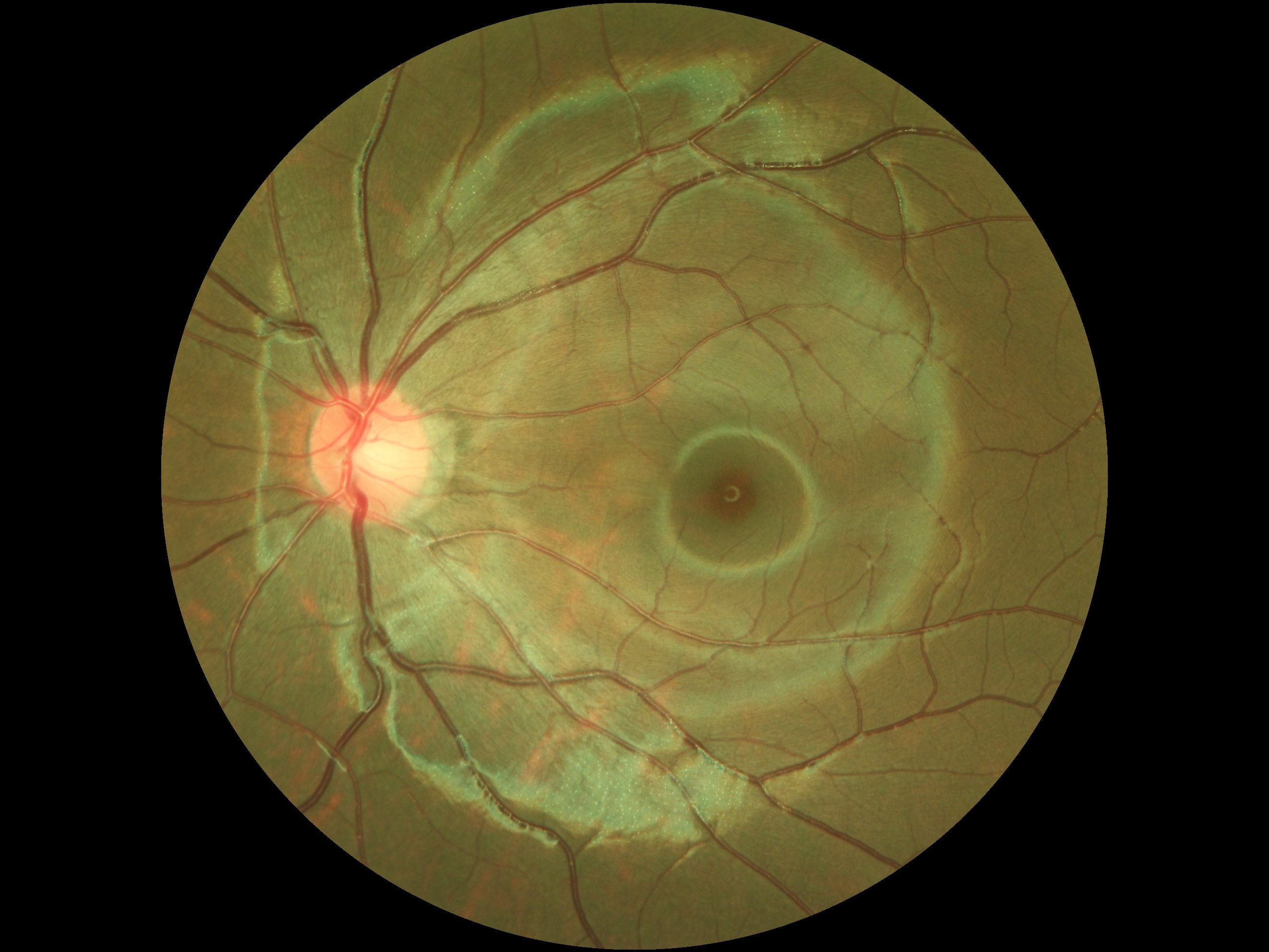

Supplement: S4 File — (ZIP) [file pone.0324352.s004.zip › Original fundus photographs (2)/Subject 70/OS_20230611847069_20230612163849_2.jpg]

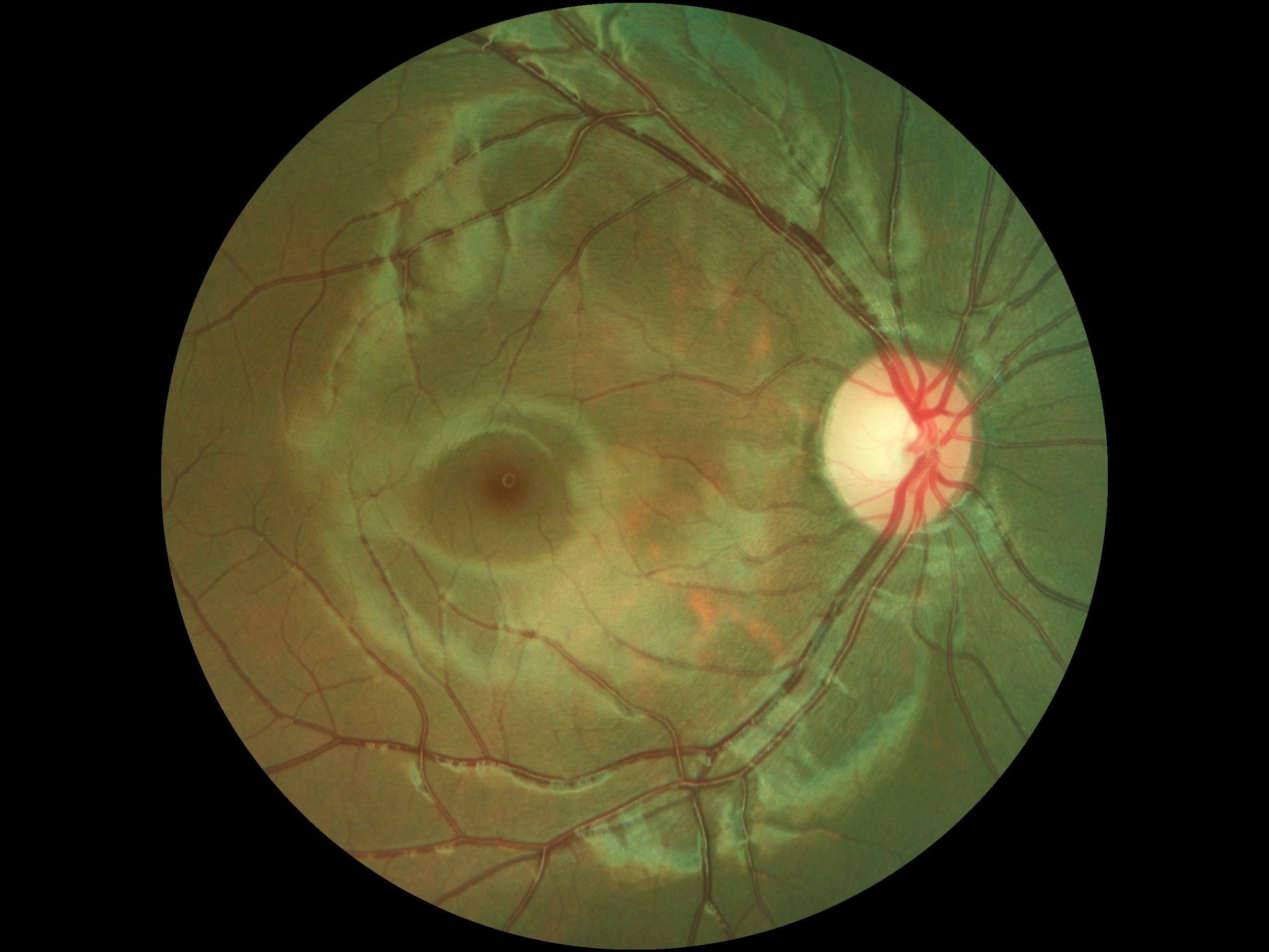

Supplement: S4 File — (ZIP) [file pone.0324352.s004.zip › Original fundus photographs (2)/Subject 71/OD_20230611198094_20230612160032_1.jpg]

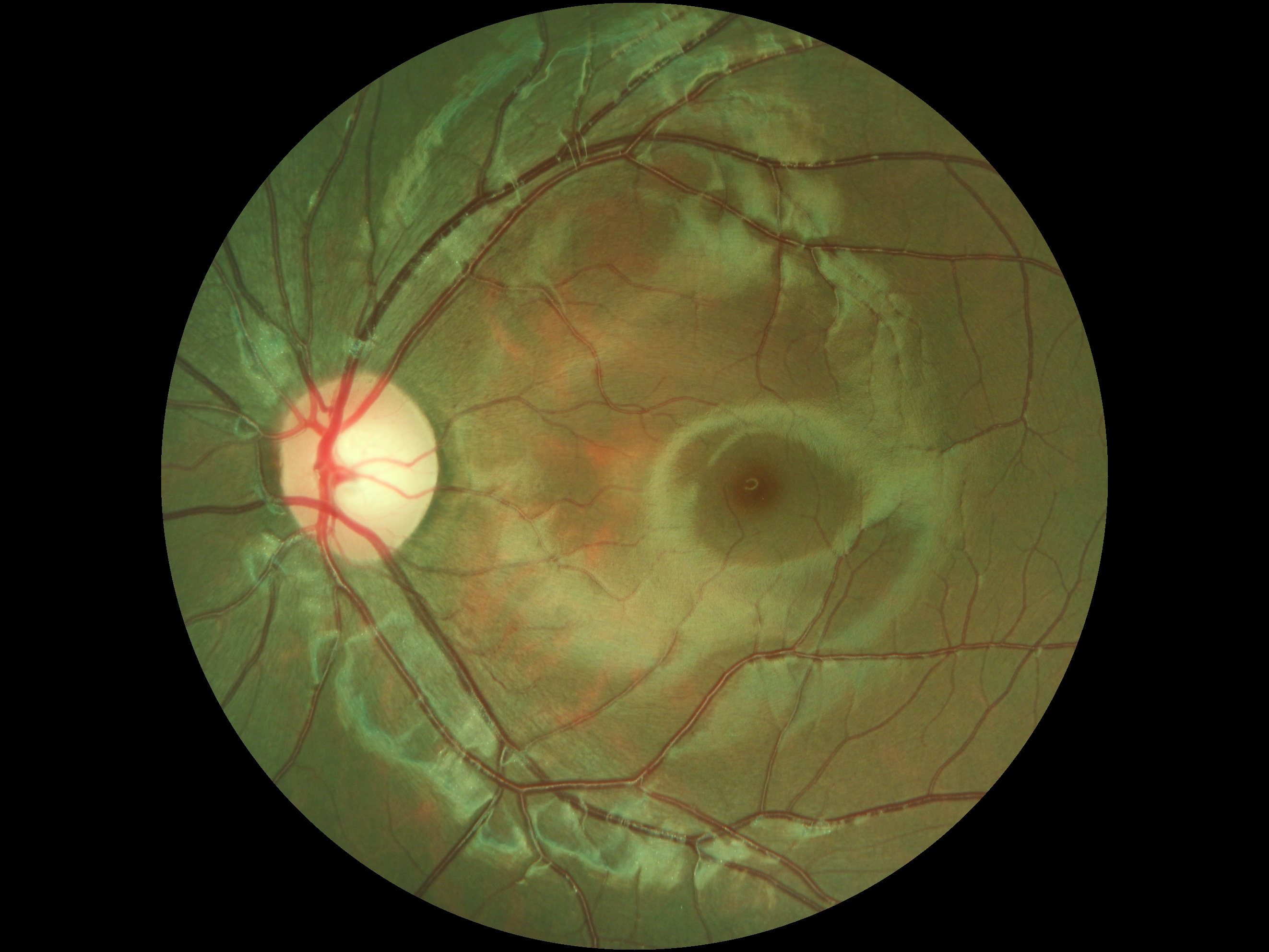

Supplement: S4 File — (ZIP) [file pone.0324352.s004.zip › Original fundus photographs (2)/Subject 71/OS_20230611198094_20230612160052_2.jpg]

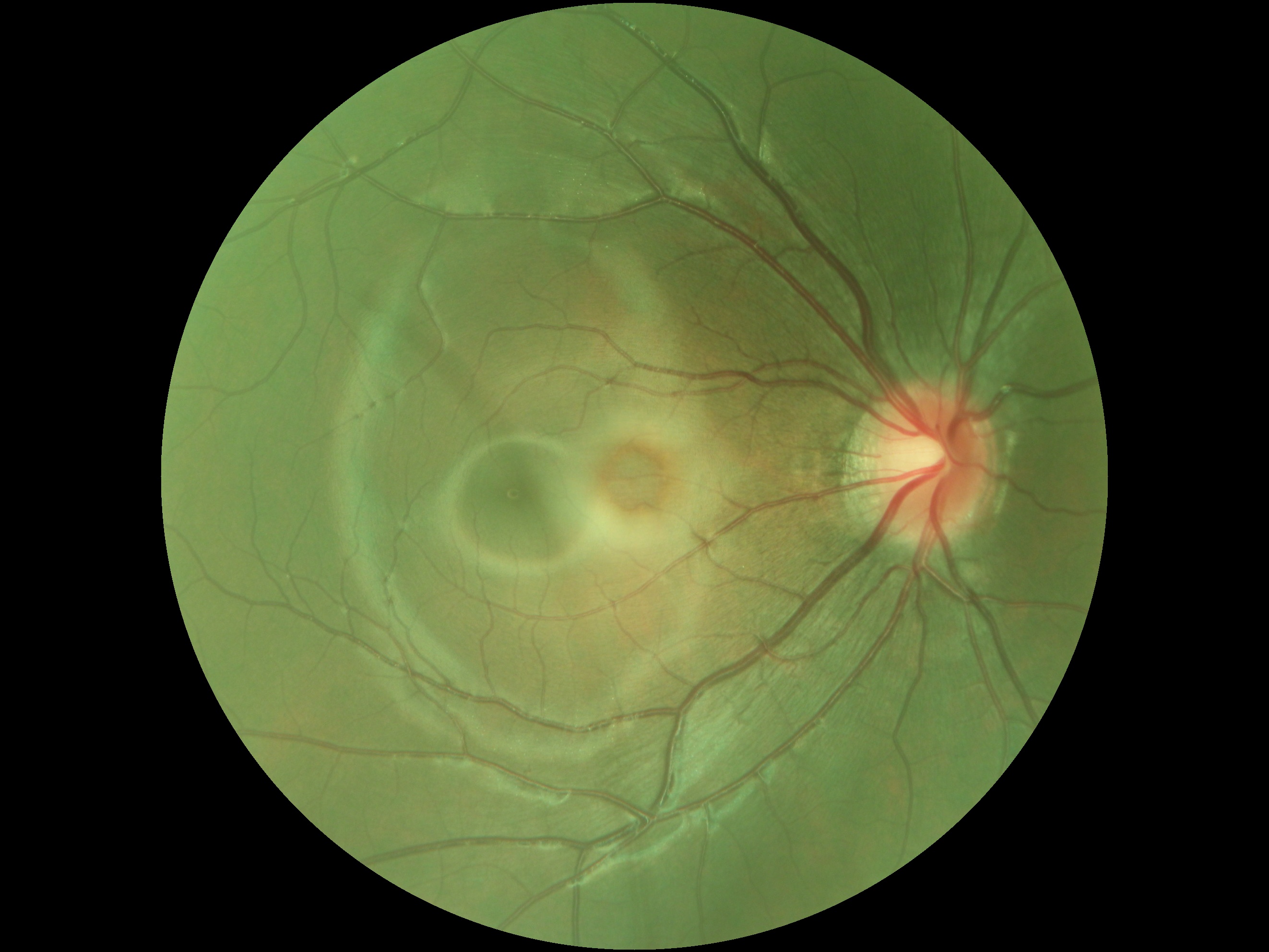

Supplement: S4 File — (ZIP) [file pone.0324352.s004.zip › Original fundus photographs (2)/Subject 72/OD_20230615876067_20230615154543_2.jpg]

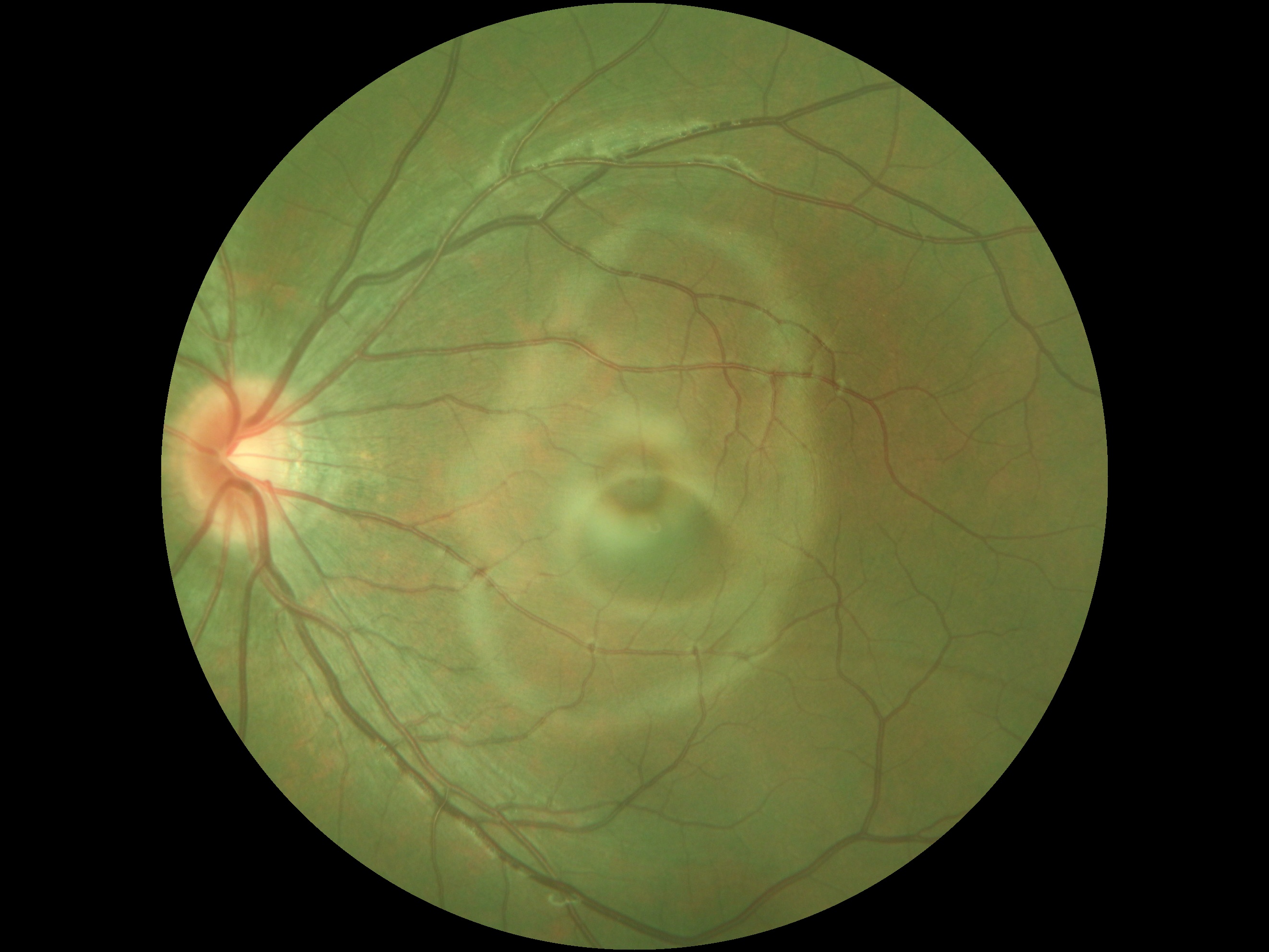

Supplement: S4 File — (ZIP) [file pone.0324352.s004.zip › Original fundus photographs (2)/Subject 72/OS_20230615876067_20230615154527_1.jpg]

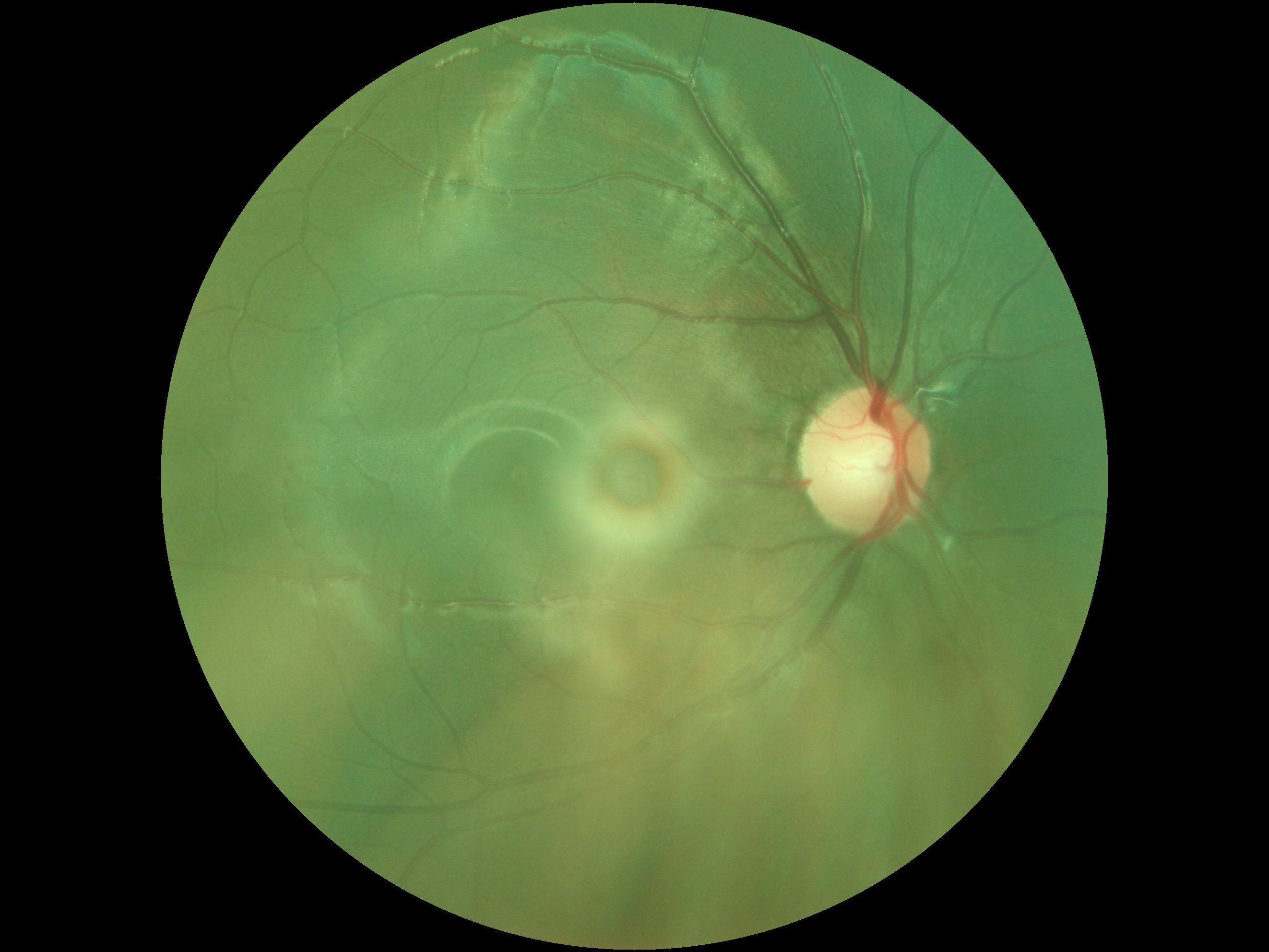

Supplement: S4 File — (ZIP) [file pone.0324352.s004.zip › Original fundus photographs (2)/Subject 73/OD_20230611212030_20230614160230_1.jpg]

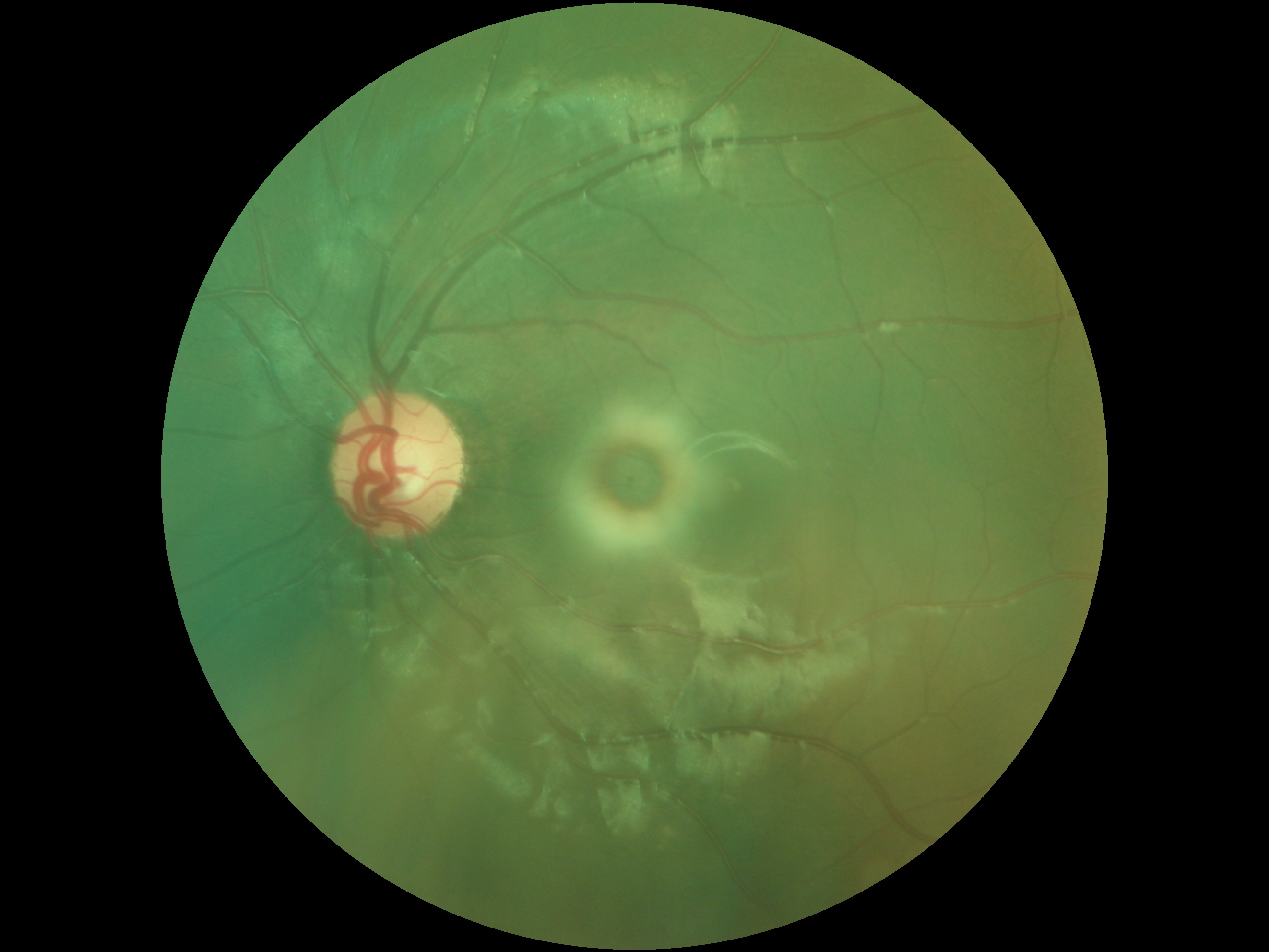

Supplement: S4 File — (ZIP) [file pone.0324352.s004.zip › Original fundus photographs (2)/Subject 73/OS_20230611212030_20230614160249_2.jpg]

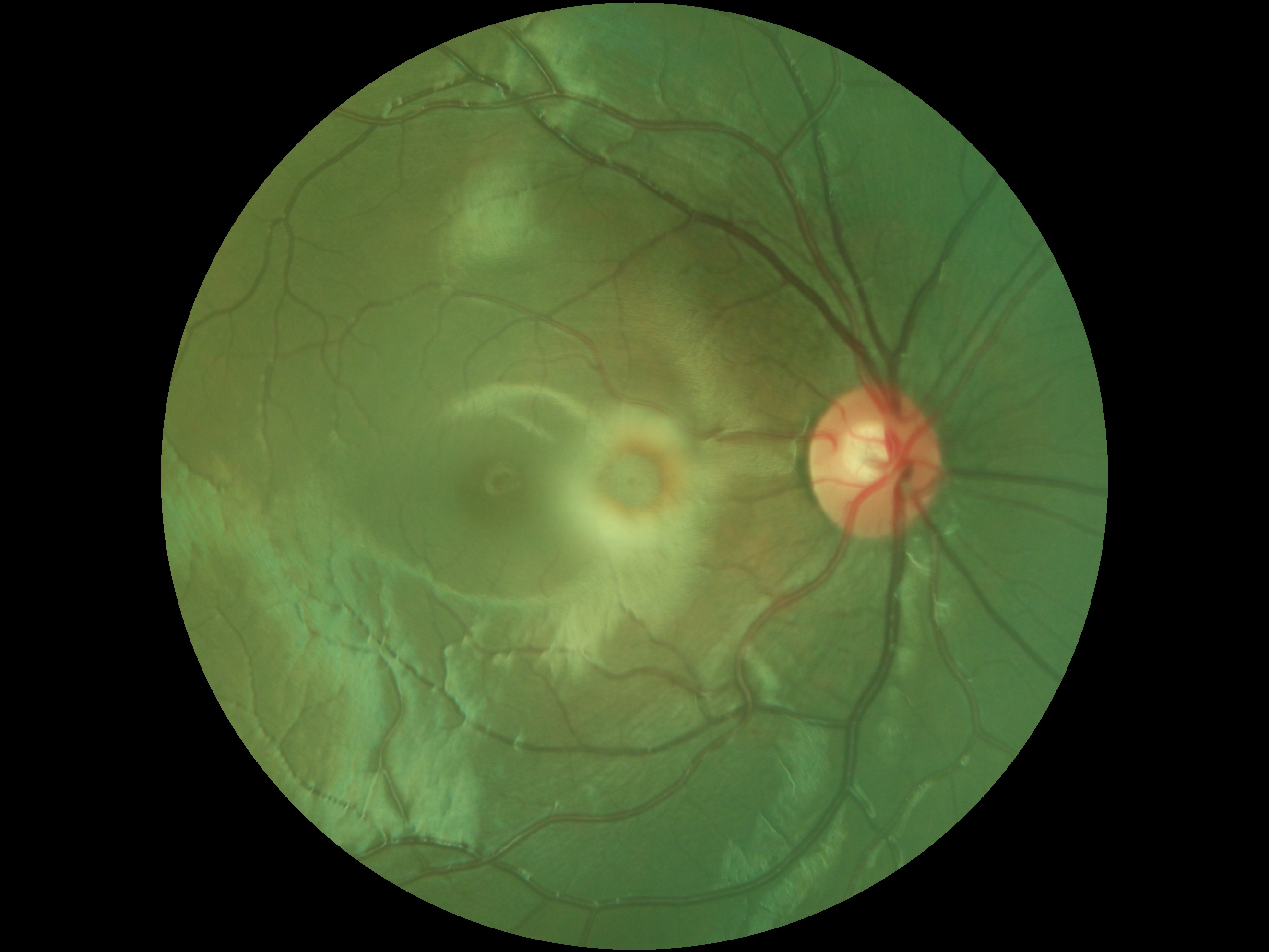

Supplement: S4 File — (ZIP) [file pone.0324352.s004.zip › Original fundus photographs (2)/Subject 74/OD_20230615931049_20230615114749_2.jpg]

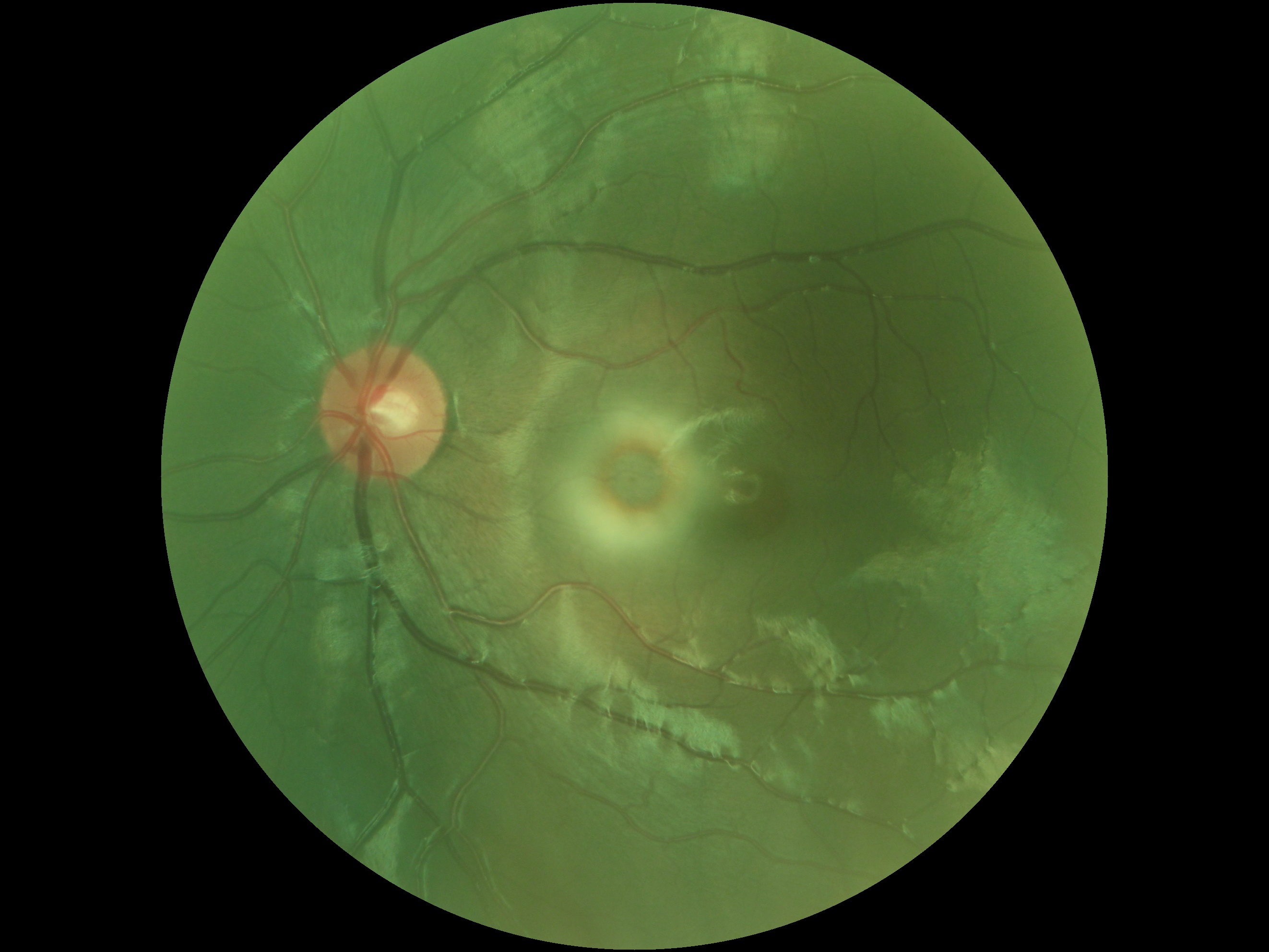

Supplement: S4 File — (ZIP) [file pone.0324352.s004.zip › Original fundus photographs (2)/Subject 74/OS_20230615931049_20230615114632_1.jpg]

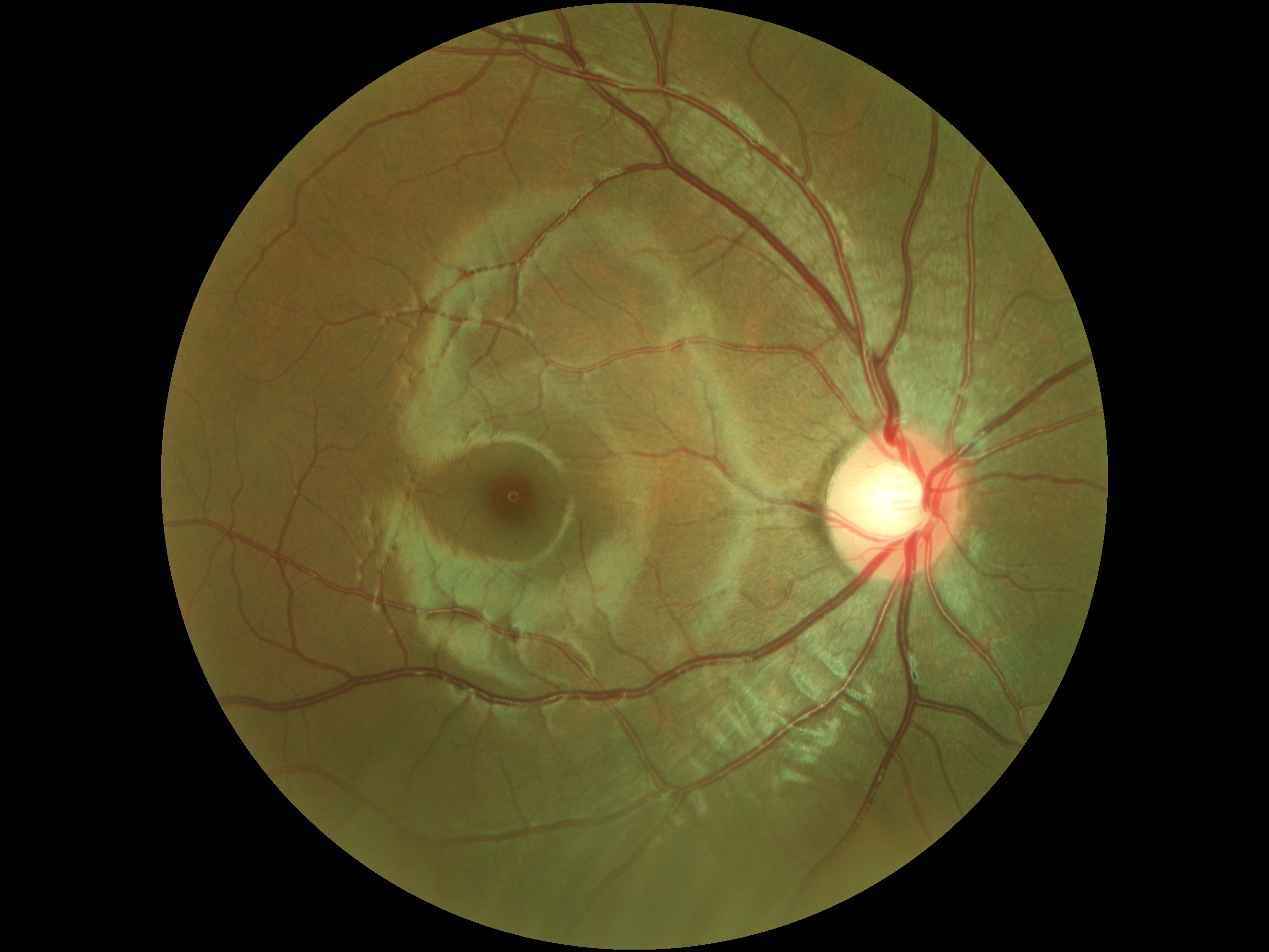

Supplement: S4 File — (ZIP) [file pone.0324352.s004.zip › Original fundus photographs (2)/Subject 75/OD_20230611931093_20230612154603_1.jpg]

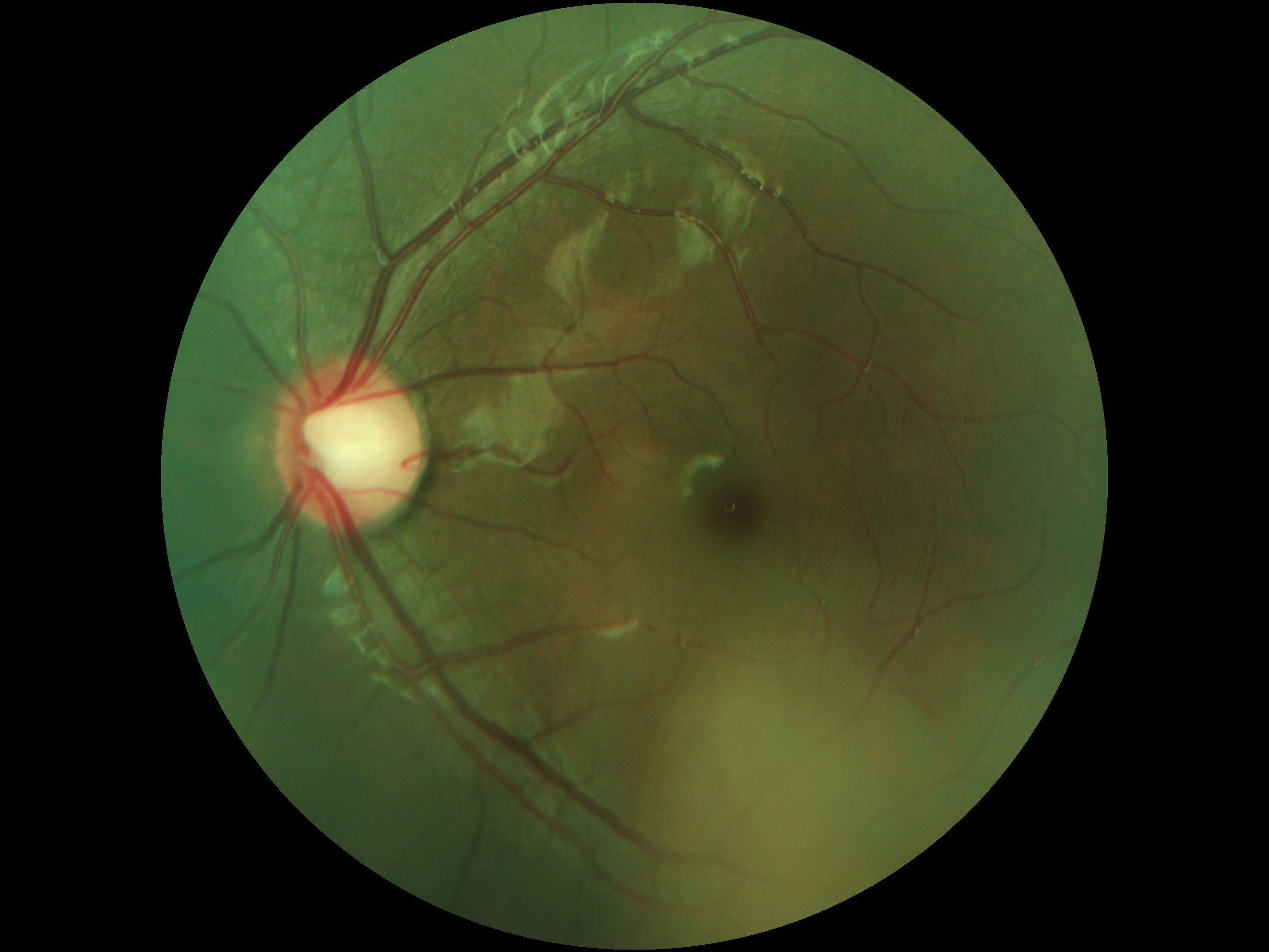

Supplement: S4 File — (ZIP) [file pone.0324352.s004.zip › Original fundus photographs (2)/Subject 75/OS_20230611931093_20230612154924_4.jpg]
