# Supplementary figures and images for: Research on the correlation between retinal vascular parameters and axial length in children using an AI-based fundus image analysis system (part 1 of 3)
Source: PLoS One. 2025 Jun 17;20(6):e0324352. doi: 10.1371/journal.pone.0324352 (PMC12173413; doi:10.1371/journal.pone.0324352)

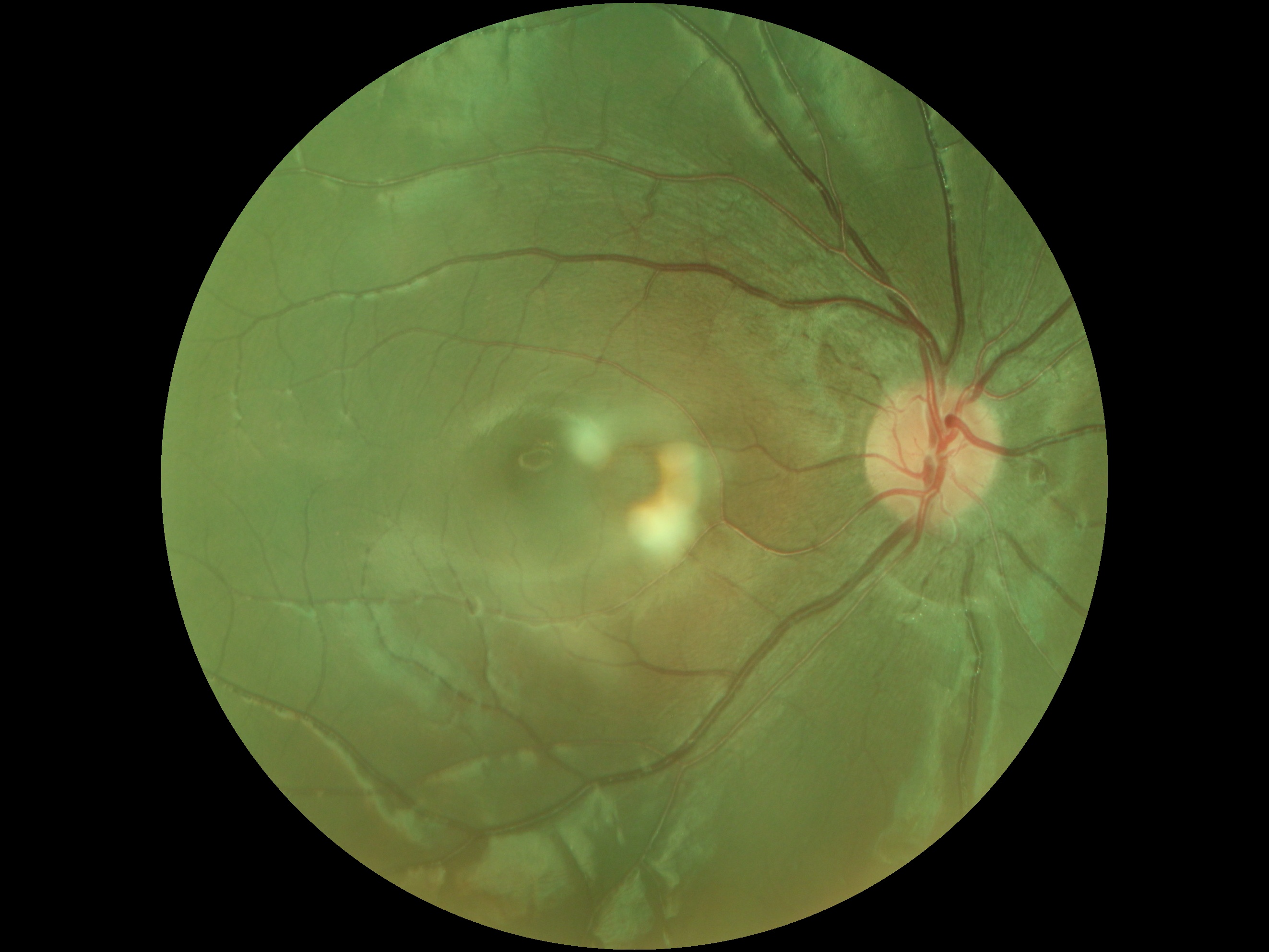

Supplement: S3 File — (ZIP) [file pone.0324352.s003.zip › Original fundus photographs (1)/Subject 1/OD_20230615223070_20230615162951_3.jpg]

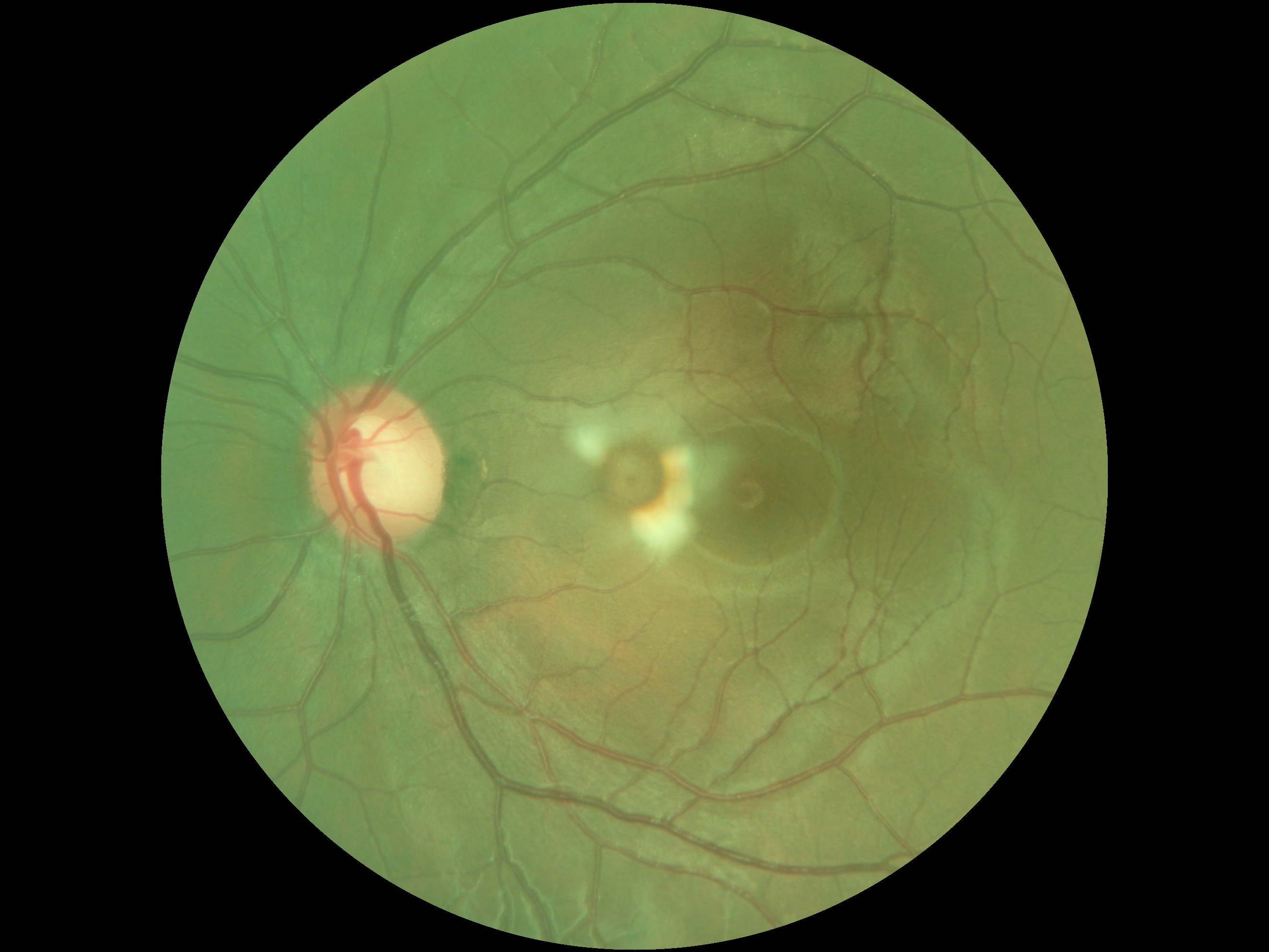

Supplement: S3 File — (ZIP) [file pone.0324352.s003.zip › Original fundus photographs (1)/Subject 1/OS_20230615223070_20230615163244_1.jpg]

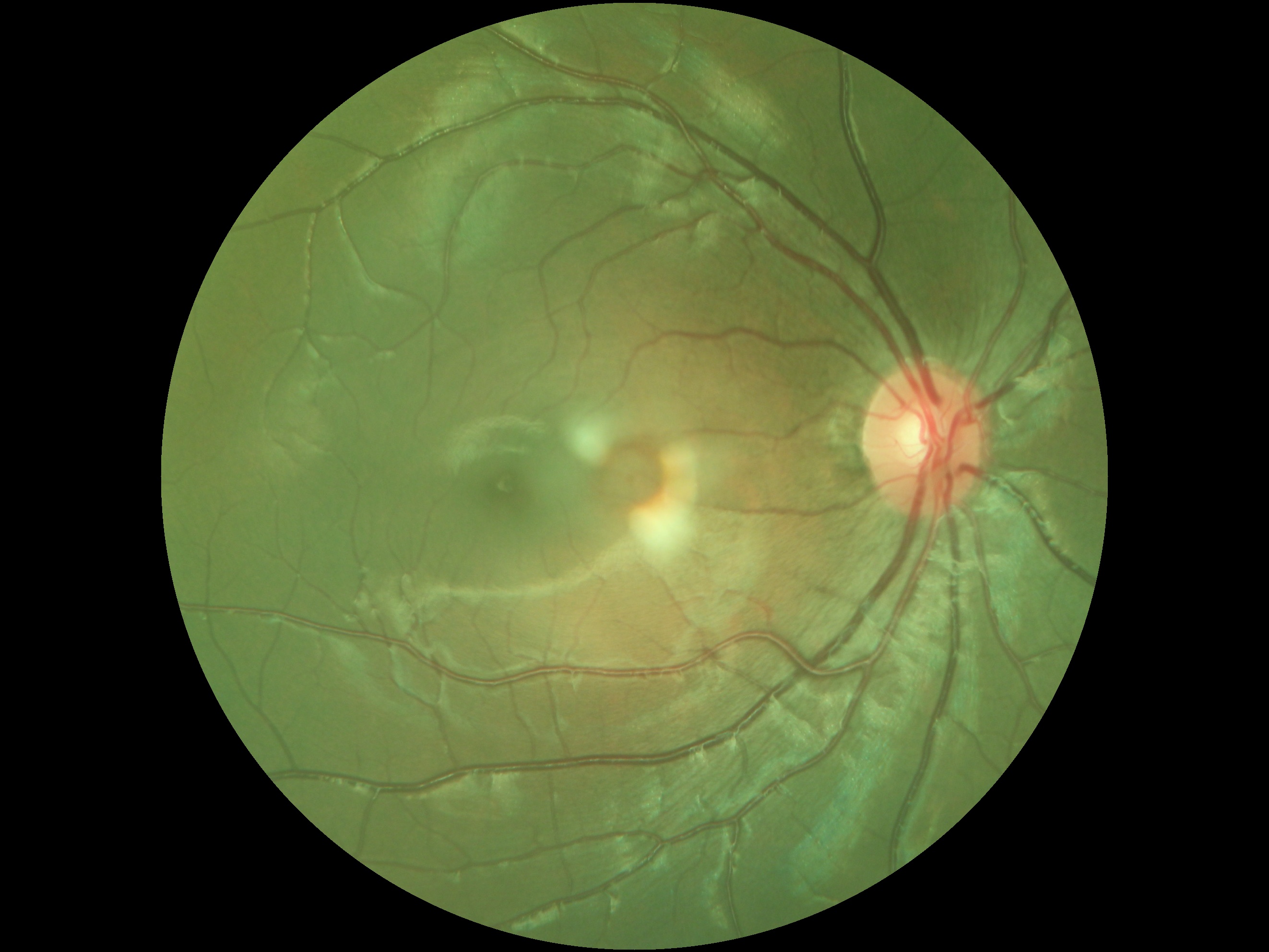

Supplement: S3 File — (ZIP) [file pone.0324352.s003.zip › Original fundus photographs (1)/Subject 10/OD_20230615054071_20230615162059_3.jpg]

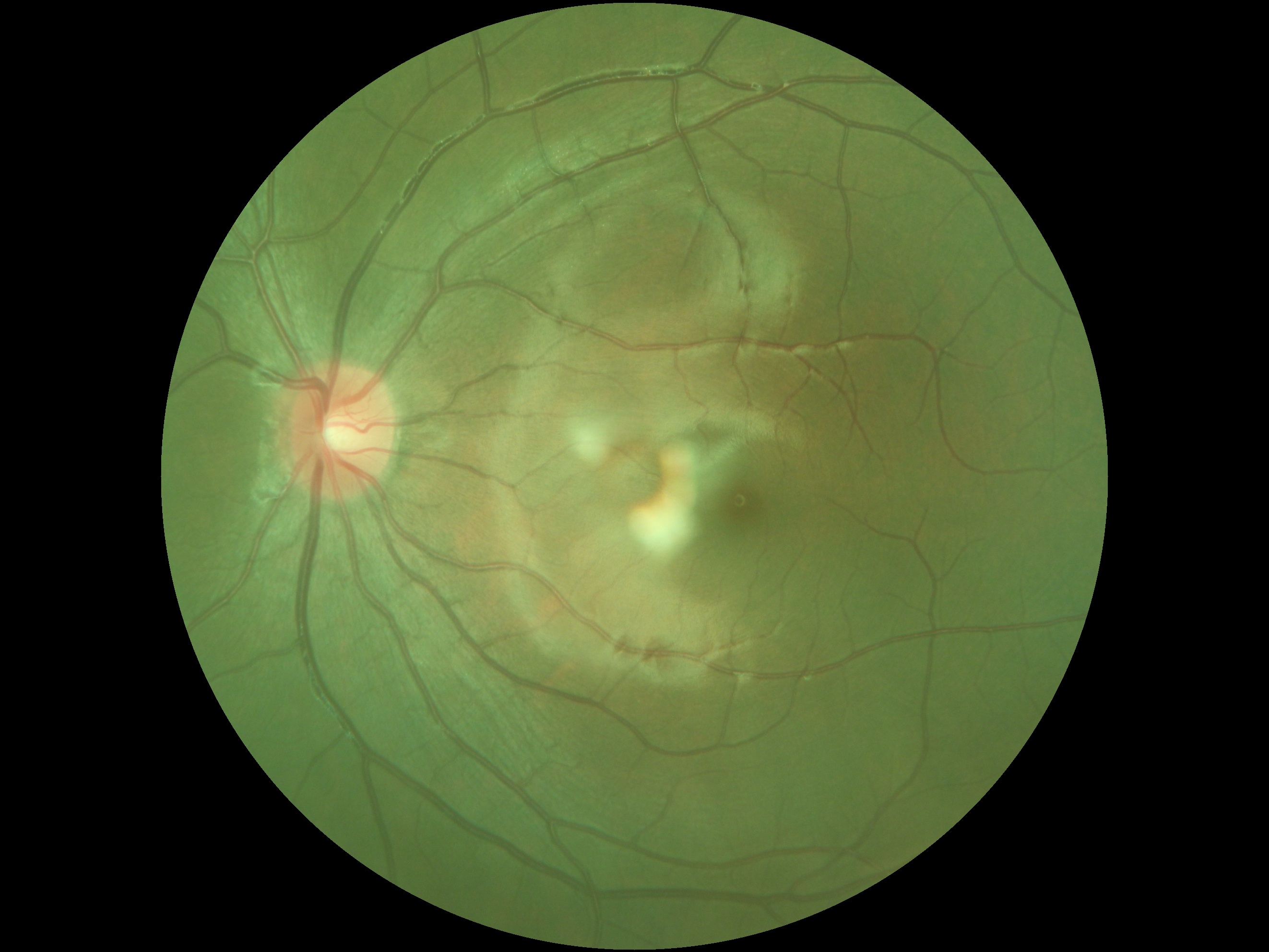

Supplement: S3 File — (ZIP) [file pone.0324352.s003.zip › Original fundus photographs (1)/Subject 10/OS_20230615054071_20230615162122_4.jpg]

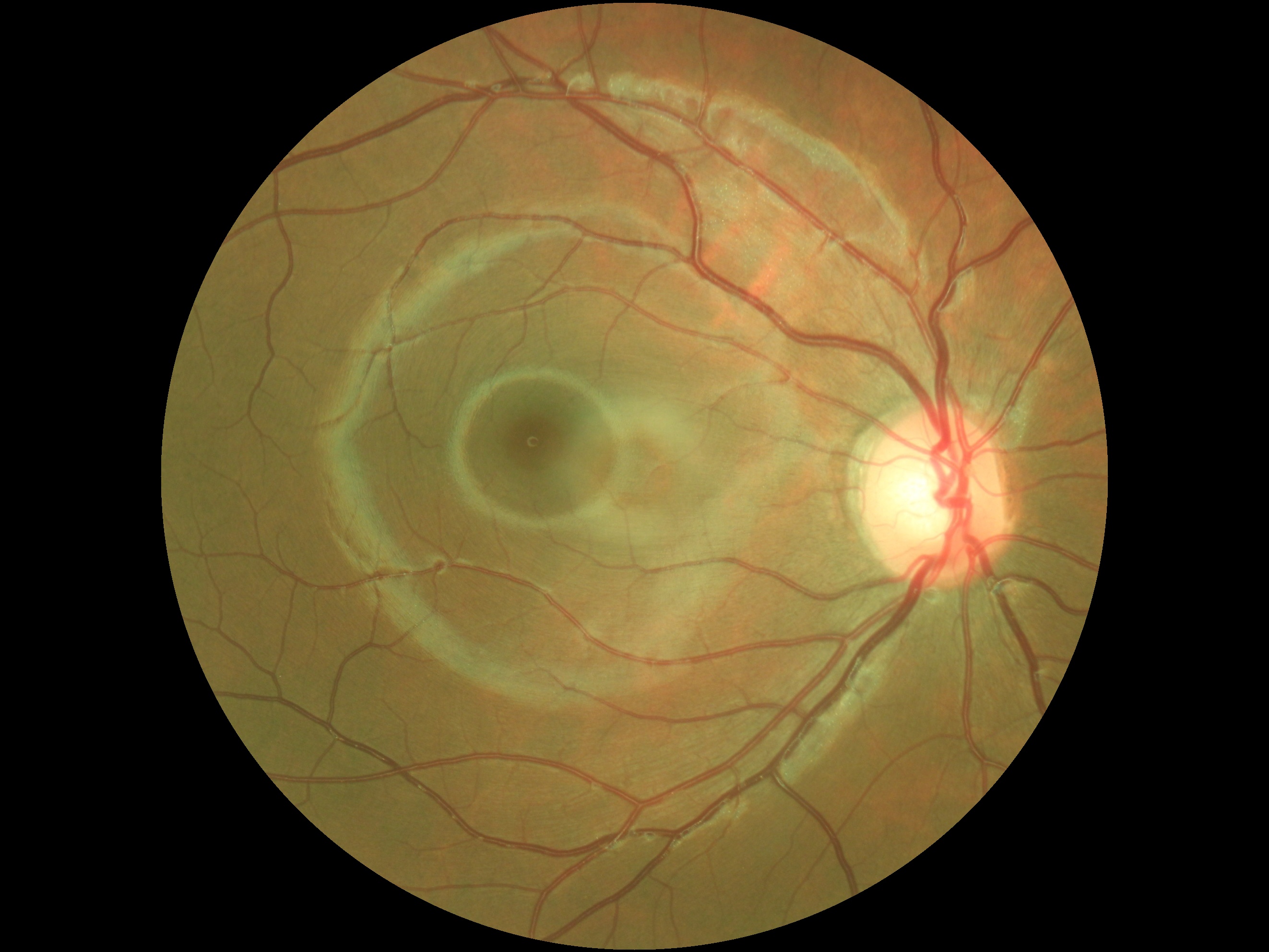

Supplement: S3 File — (ZIP) [file pone.0324352.s003.zip › Original fundus photographs (1)/Subject 11/OD_20230611170277_20230615104923_2.jpg]

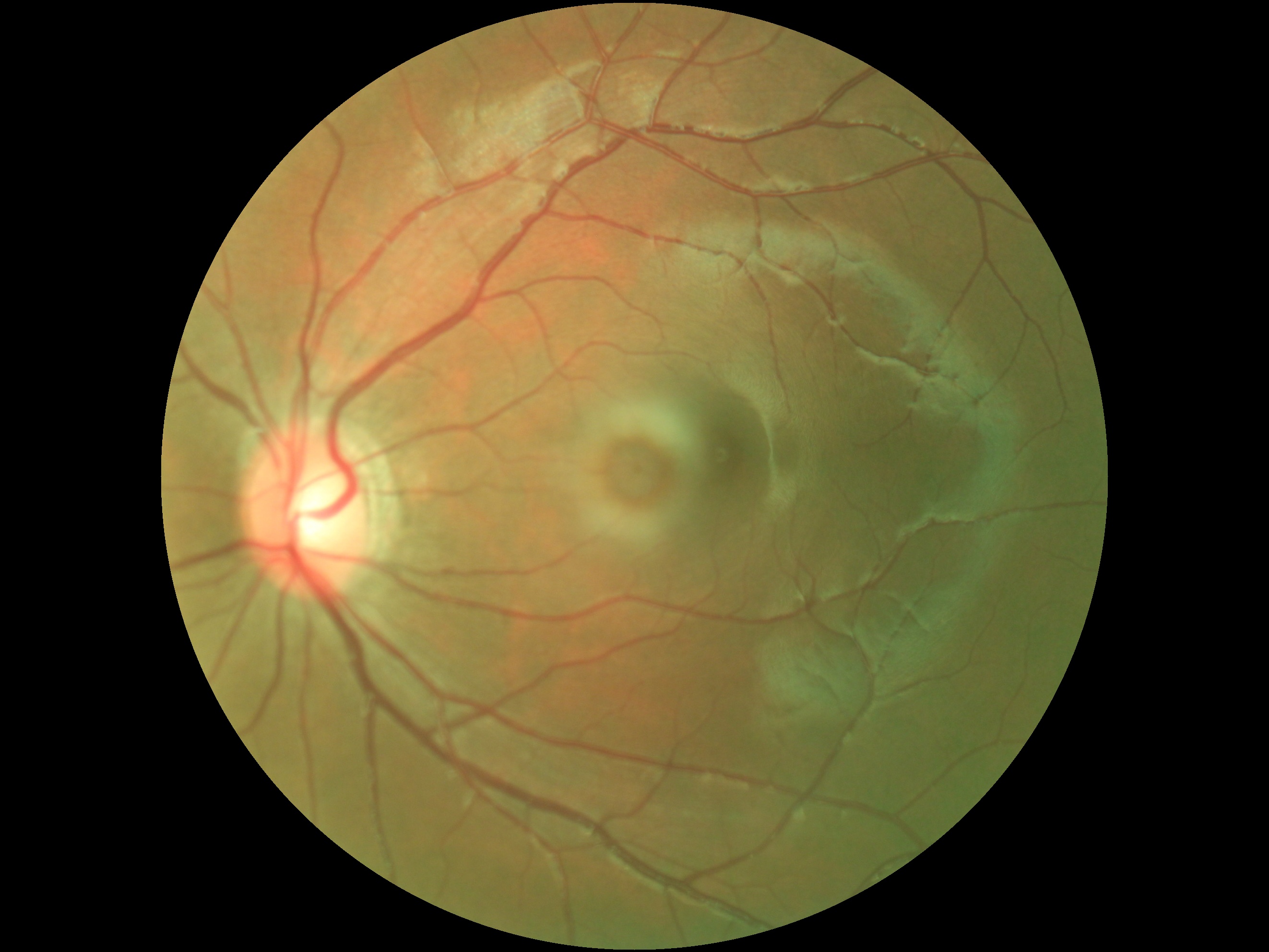

Supplement: S3 File — (ZIP) [file pone.0324352.s003.zip › Original fundus photographs (1)/Subject 11/OS_20230611170277_20230615104904_1.jpg]

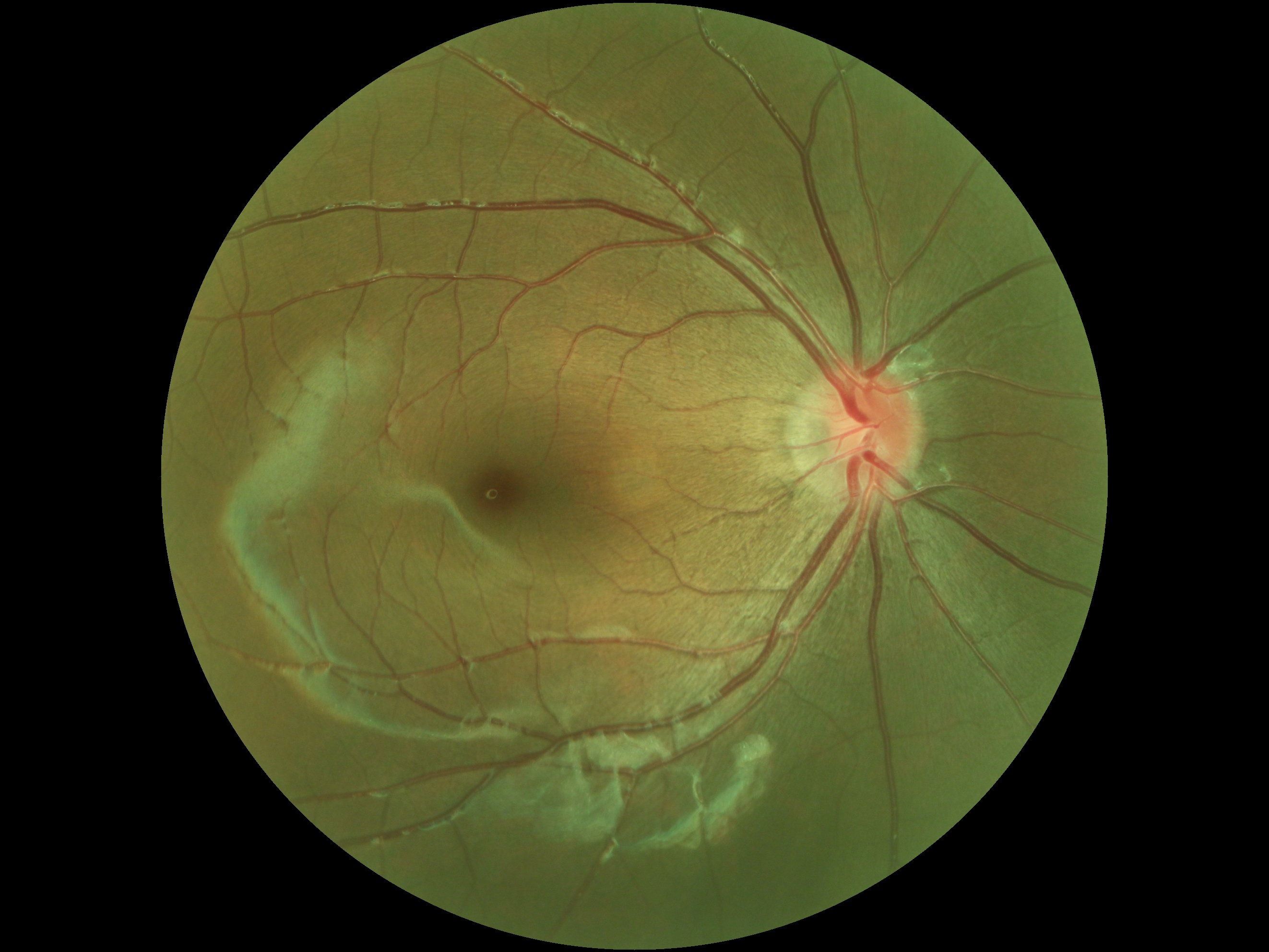

Supplement: S3 File — (ZIP) [file pone.0324352.s003.zip › Original fundus photographs (1)/Subject 12/OD_20230611791027_20230612152845_1.jpg]

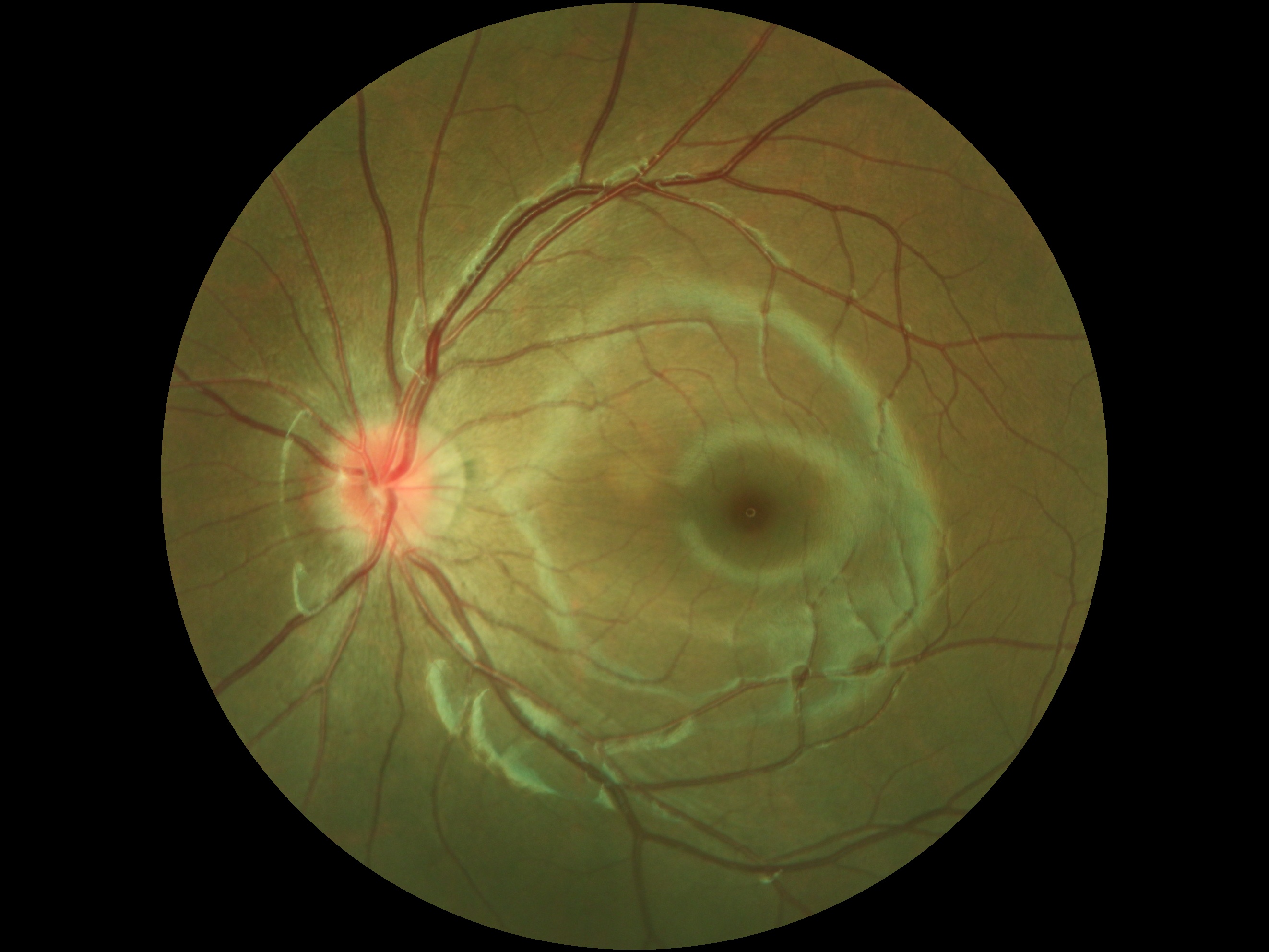

Supplement: S3 File — (ZIP) [file pone.0324352.s003.zip › Original fundus photographs (1)/Subject 12/OS_20230611791027_20230612152930_2.jpg]

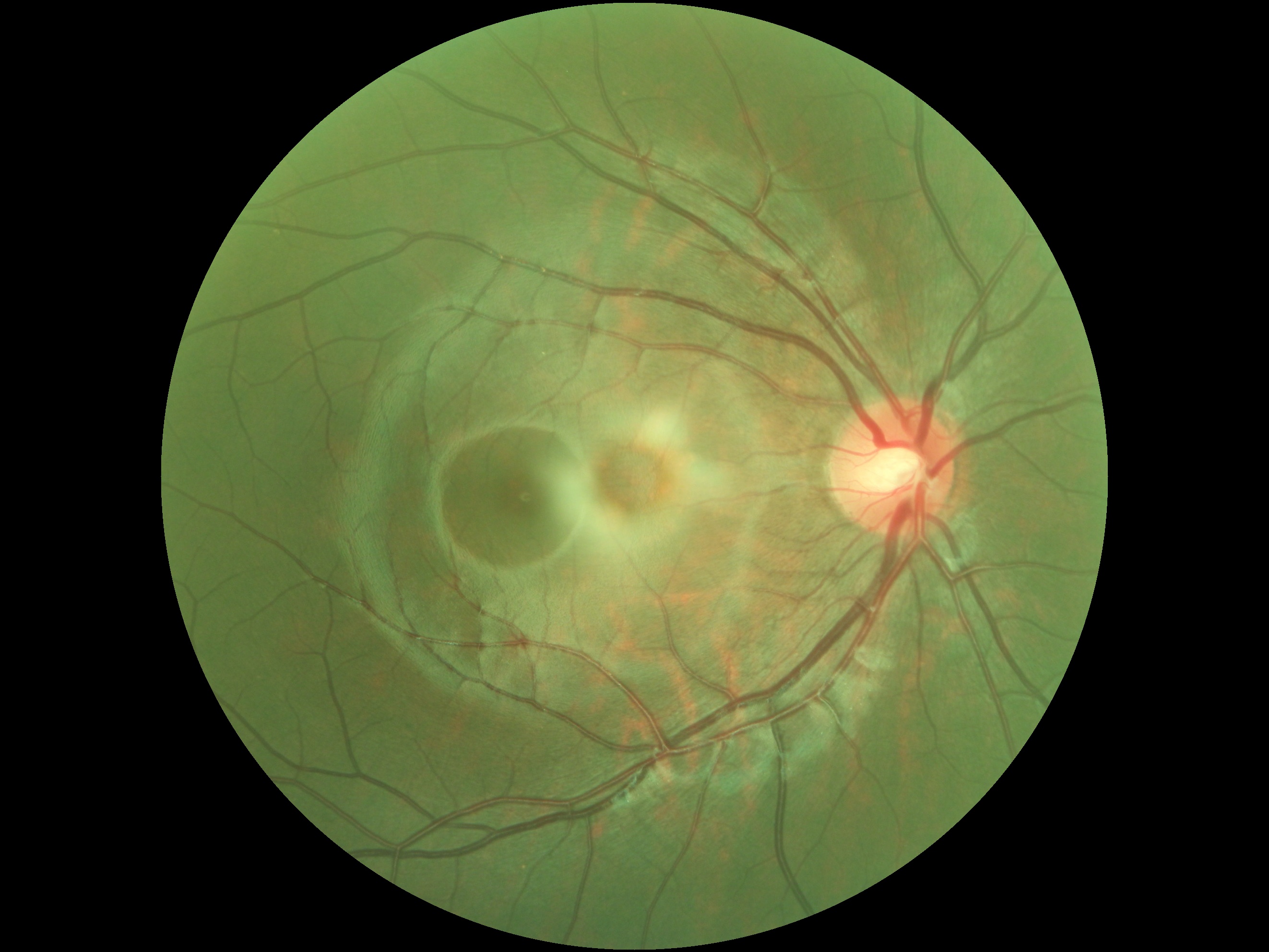

Supplement: S3 File — (ZIP) [file pone.0324352.s003.zip › Original fundus photographs (1)/Subject 13/OD_20230611844216_20230613165624_1.jpg]

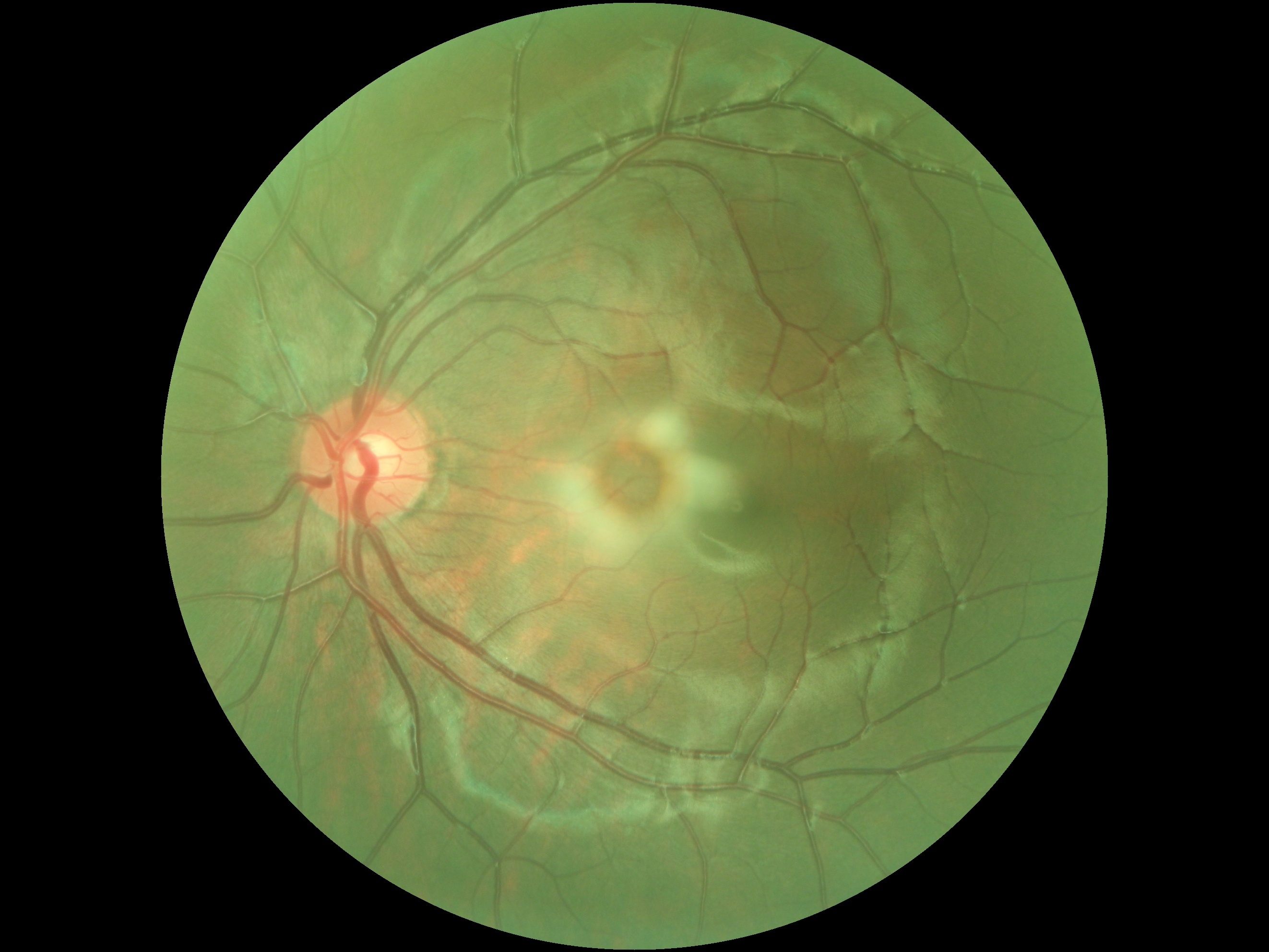

Supplement: S3 File — (ZIP) [file pone.0324352.s003.zip › Original fundus photographs (1)/Subject 13/OS_20230611844216_20230613165634_2.jpg]

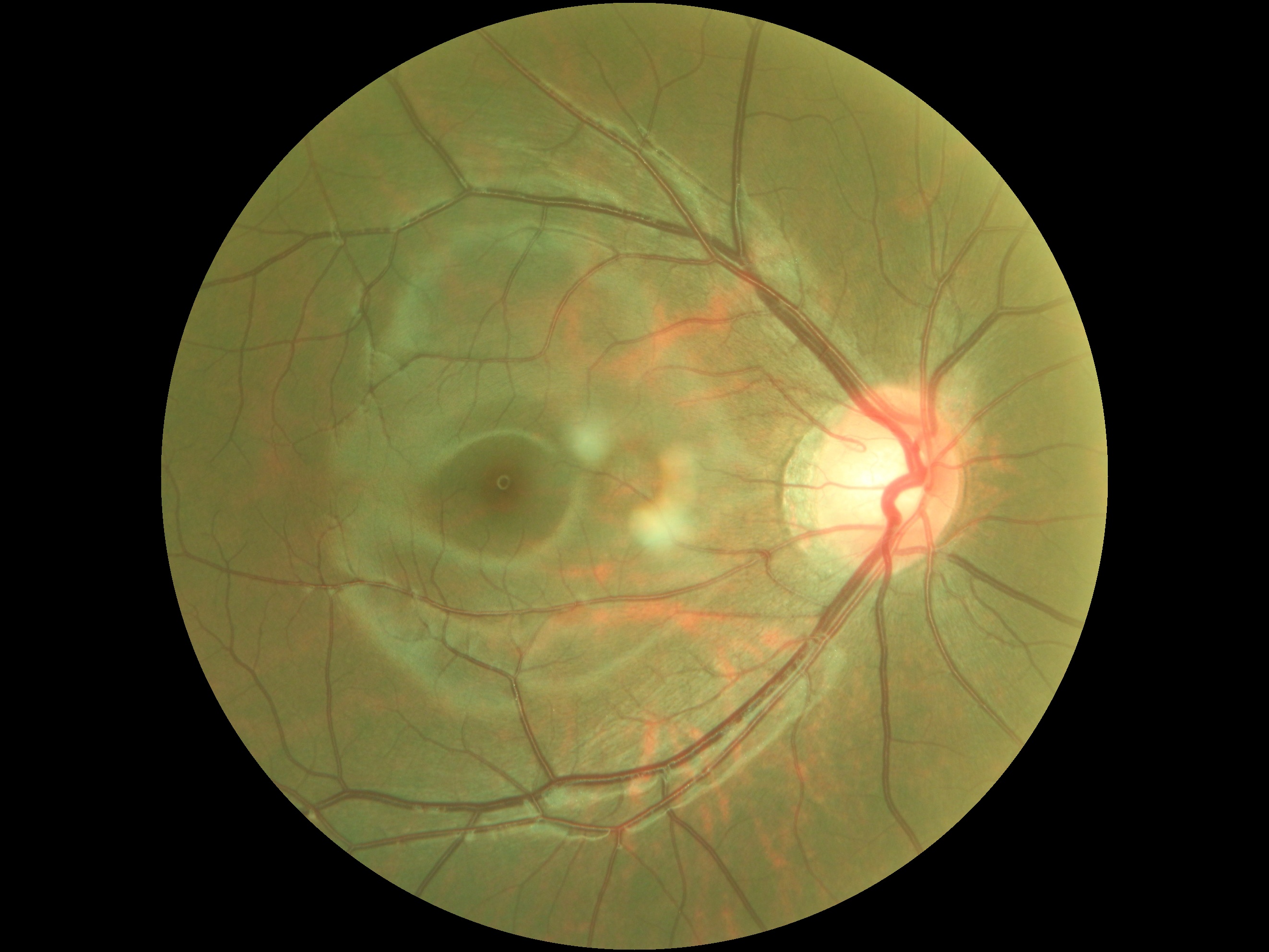

Supplement: S3 File — (ZIP) [file pone.0324352.s003.zip › Original fundus photographs (1)/Subject 14/OD_20230615794072_20230615161726_1.jpg]

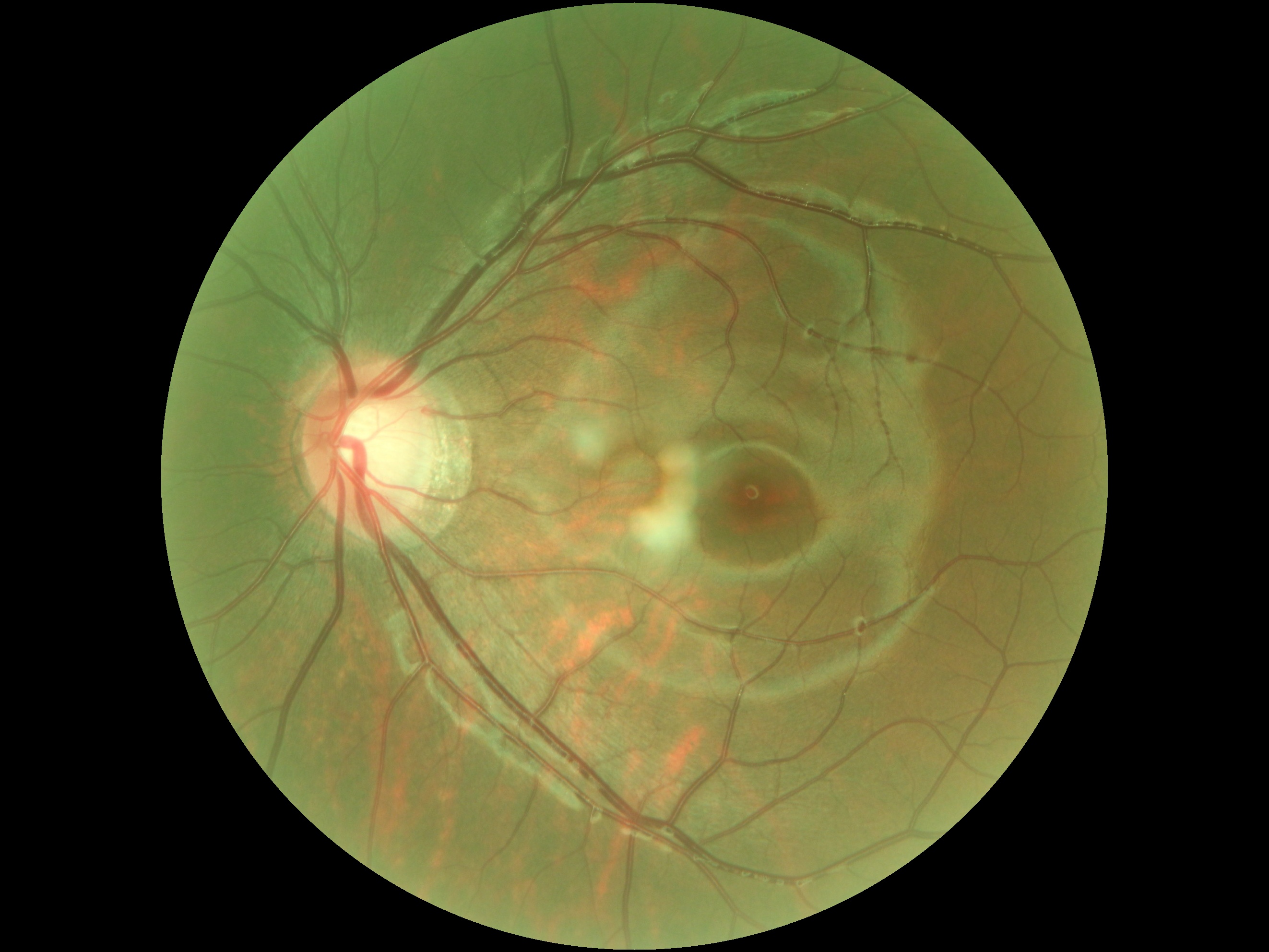

Supplement: S3 File — (ZIP) [file pone.0324352.s003.zip › Original fundus photographs (1)/Subject 14/OS_20230615794072_20230615161753_2.jpg]

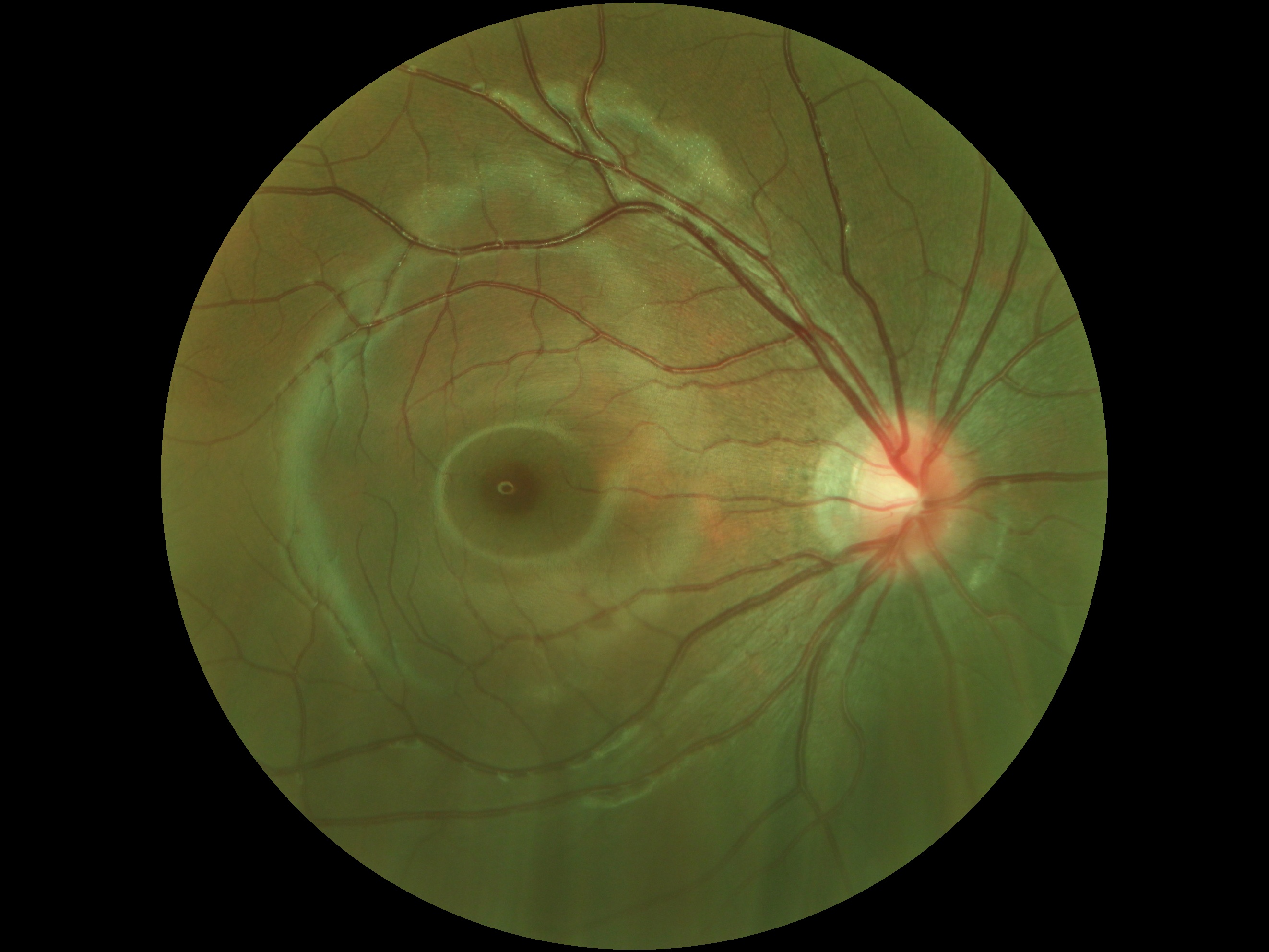

Supplement: S3 File — (ZIP) [file pone.0324352.s003.zip › Original fundus photographs (1)/Subject 15/OD_20230611572006_20230612111521_1.jpg]

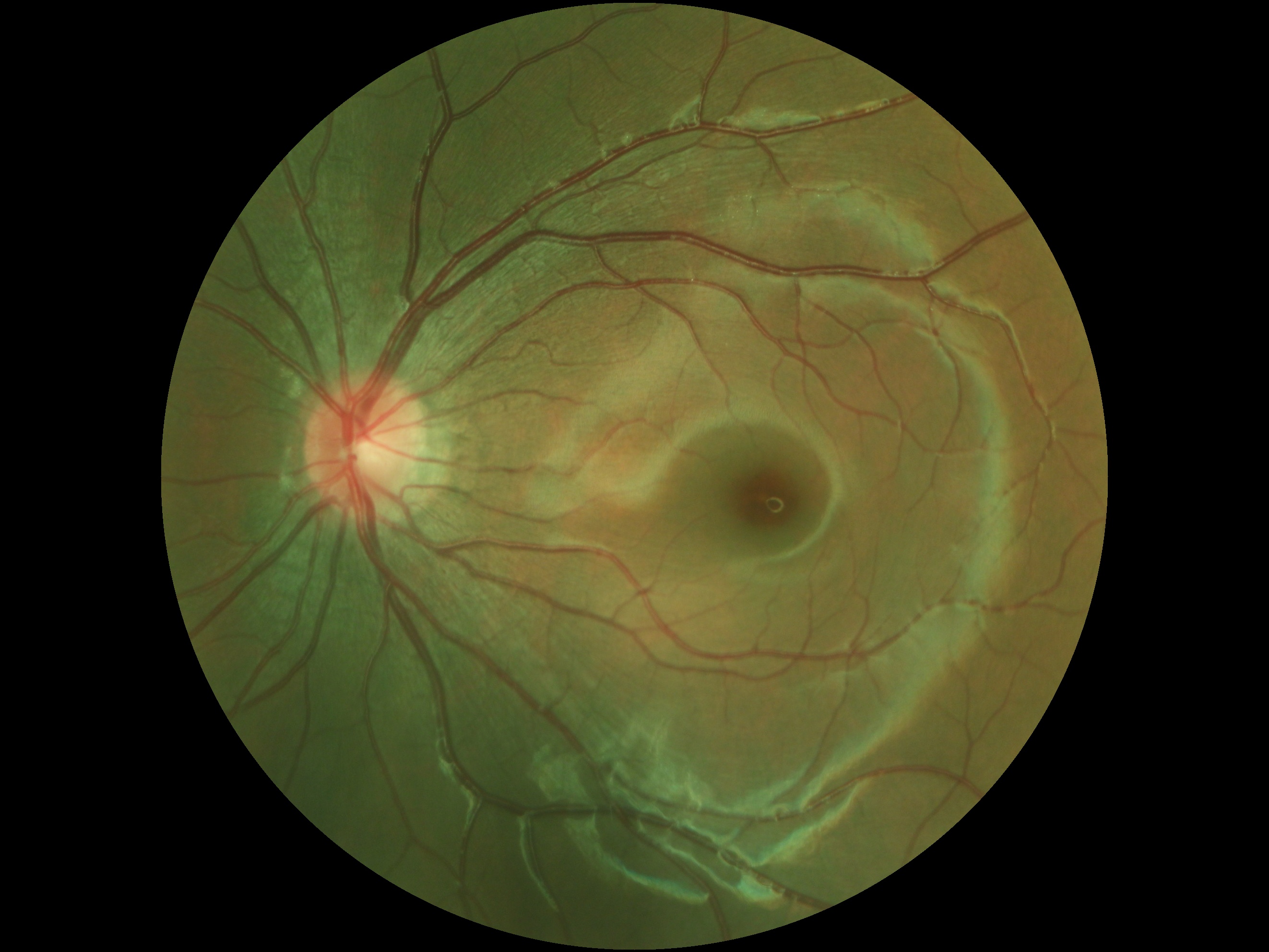

Supplement: S3 File — (ZIP) [file pone.0324352.s003.zip › Original fundus photographs (1)/Subject 15/OS_20230611572006_20230612111559_2.jpg]

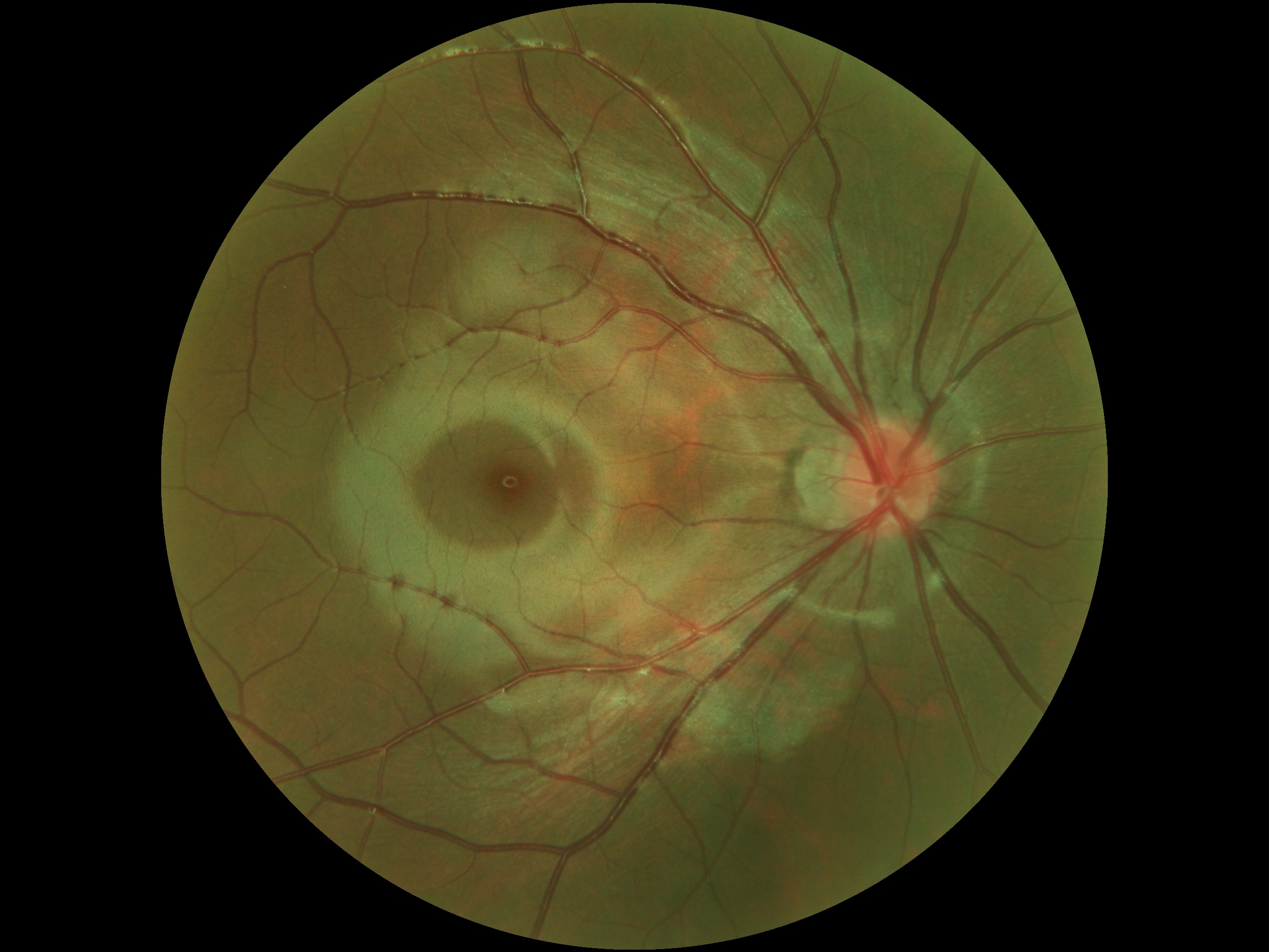

Supplement: S3 File — (ZIP) [file pone.0324352.s003.zip › Original fundus photographs (1)/Subject 16/OD_20230611286020_20230612112900_1.jpg]

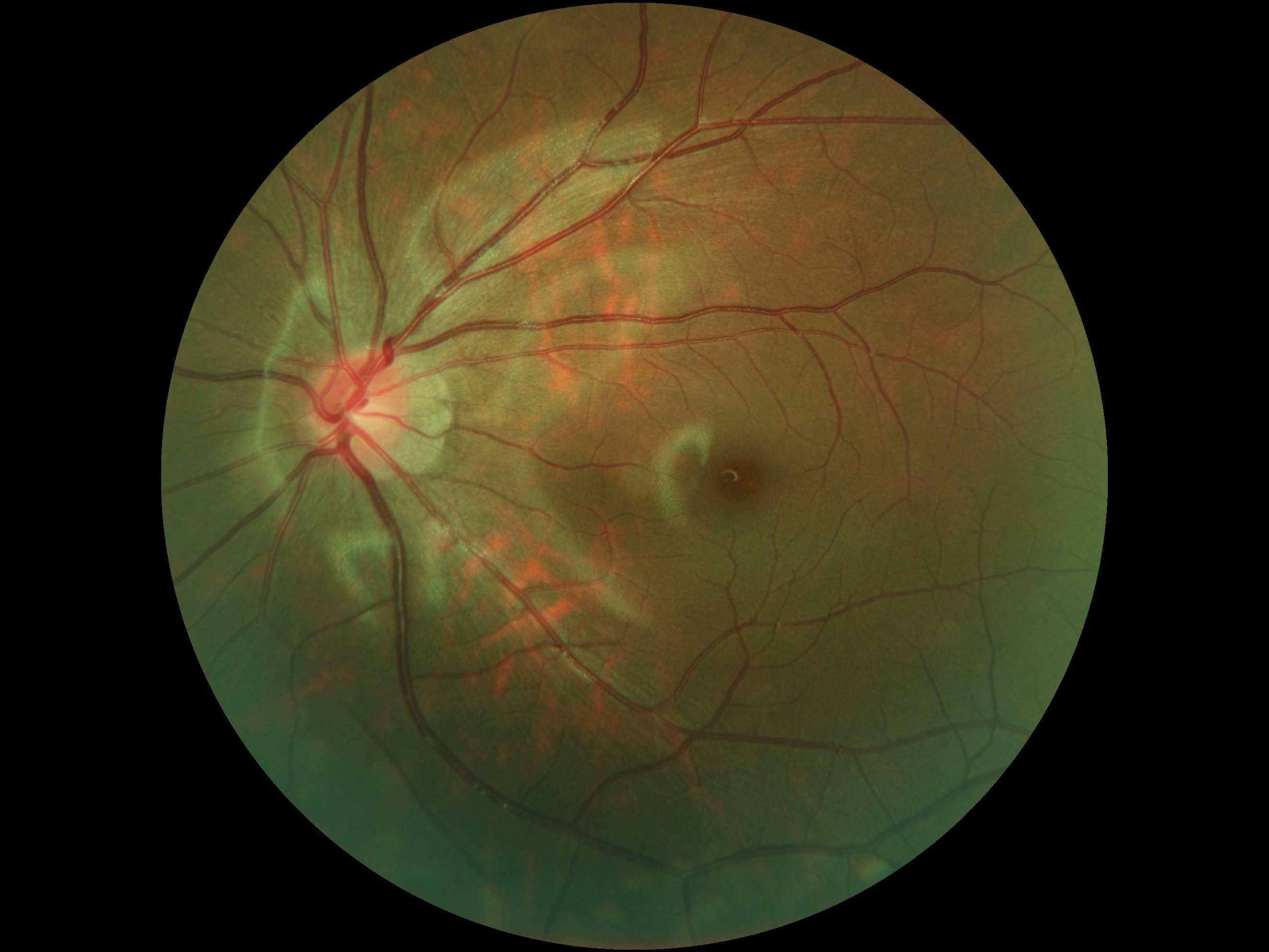

Supplement: S3 File — (ZIP) [file pone.0324352.s003.zip › Original fundus photographs (1)/Subject 16/OS_20230611286020_20230612112934_2.jpg]

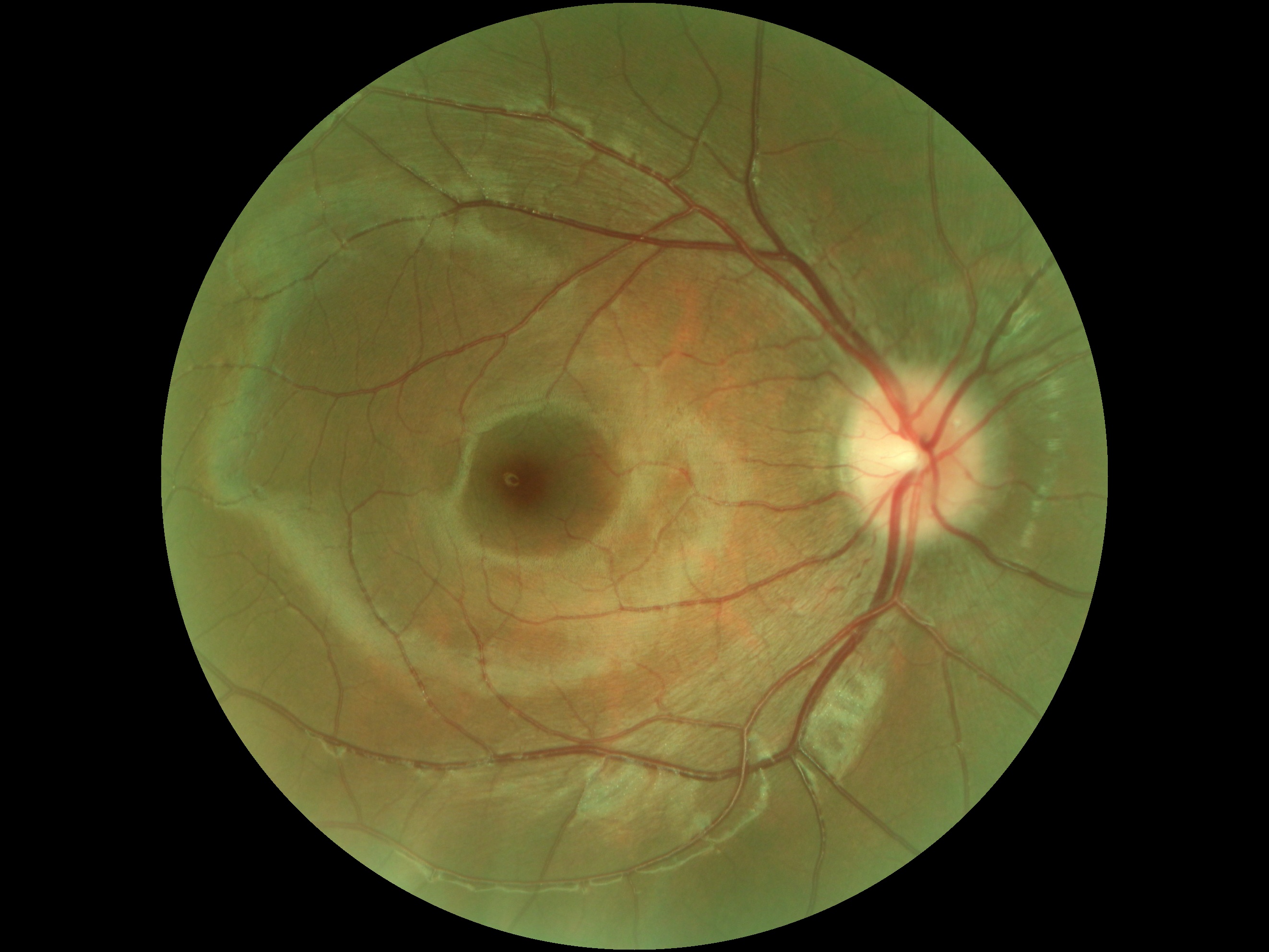

Supplement: S3 File — (ZIP) [file pone.0324352.s003.zip › Original fundus photographs (1)/Subject 17/OD_20230611420064_20230612161421_1.jpg]

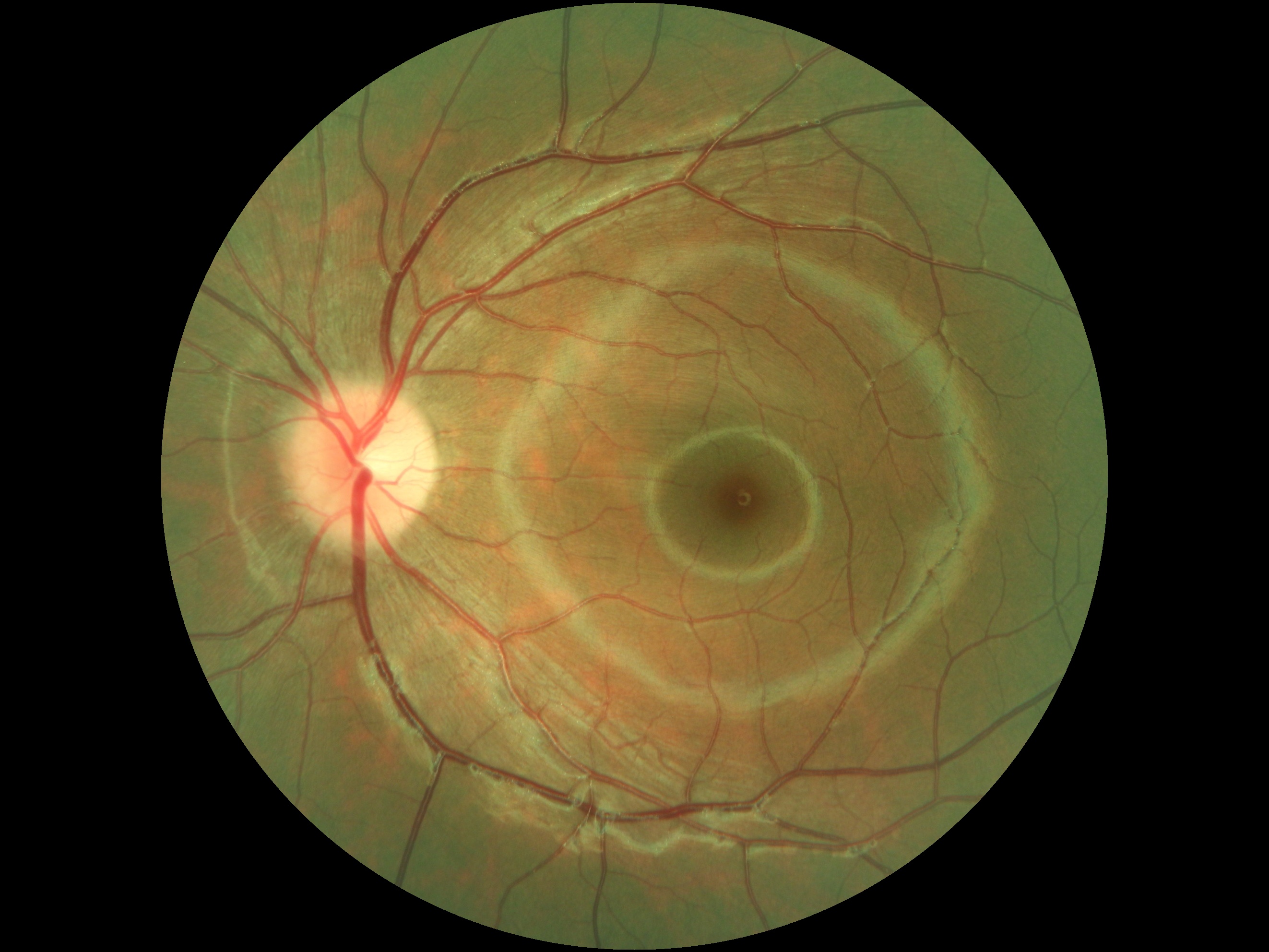

Supplement: S3 File — (ZIP) [file pone.0324352.s003.zip › Original fundus photographs (1)/Subject 17/OS_20230611420064_20230612161503_2.jpg]

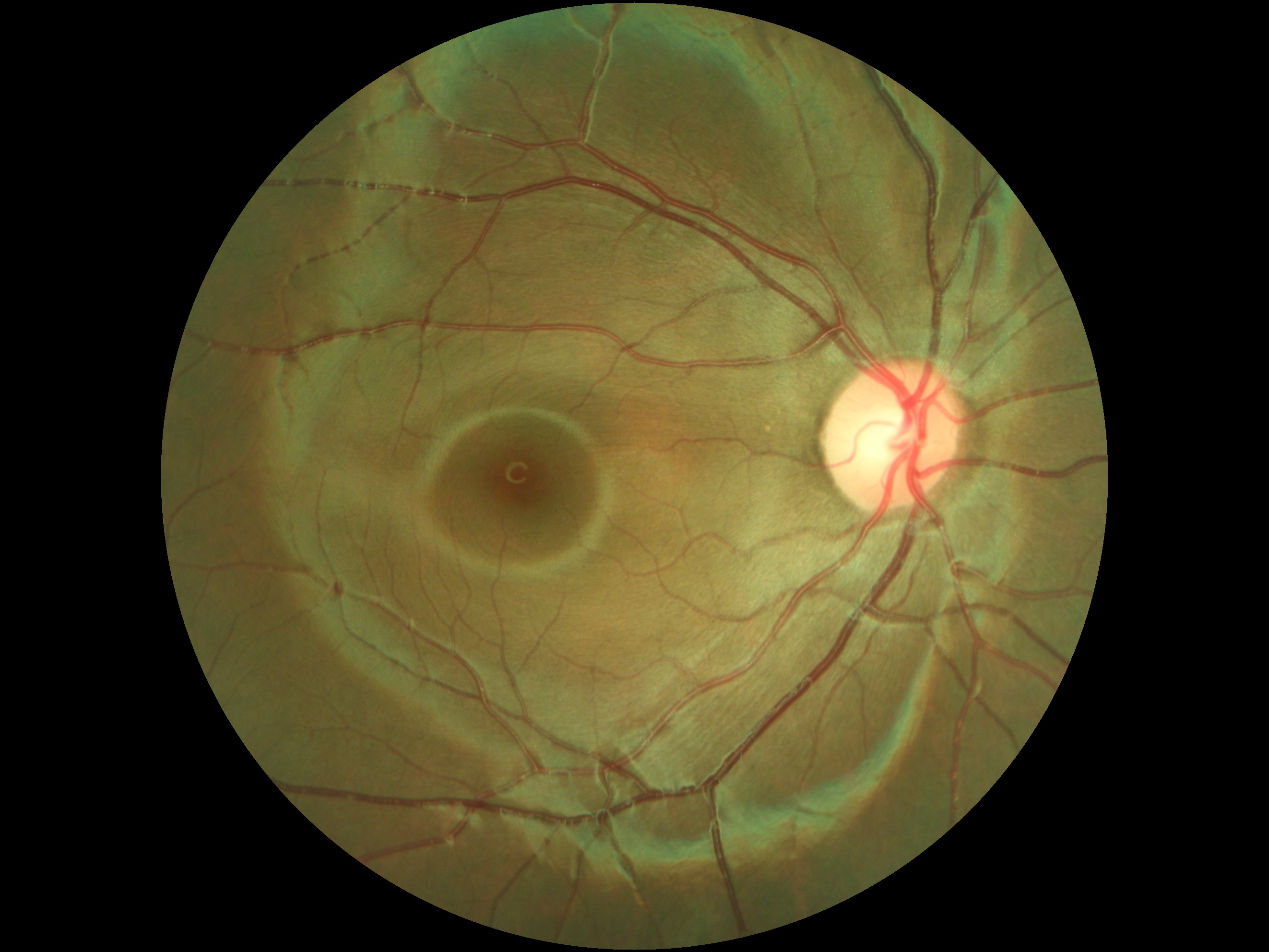

Supplement: S3 File — (ZIP) [file pone.0324352.s003.zip › Original fundus photographs (1)/Subject 18/OD_20230611263026_20230612161751_1.jpg]

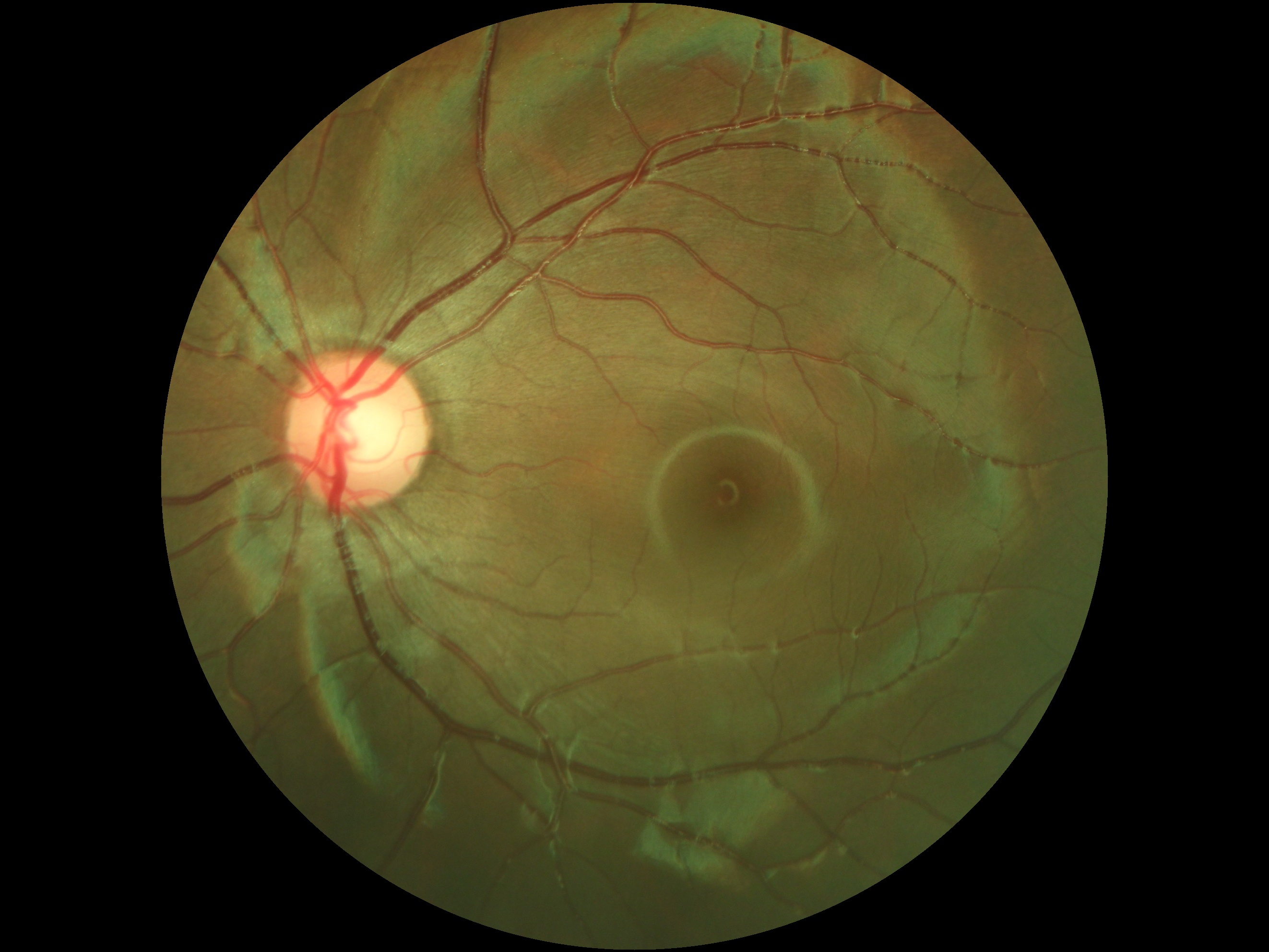

Supplement: S3 File — (ZIP) [file pone.0324352.s003.zip › Original fundus photographs (1)/Subject 18/OS_20230611263026_20230612161820_2.jpg]

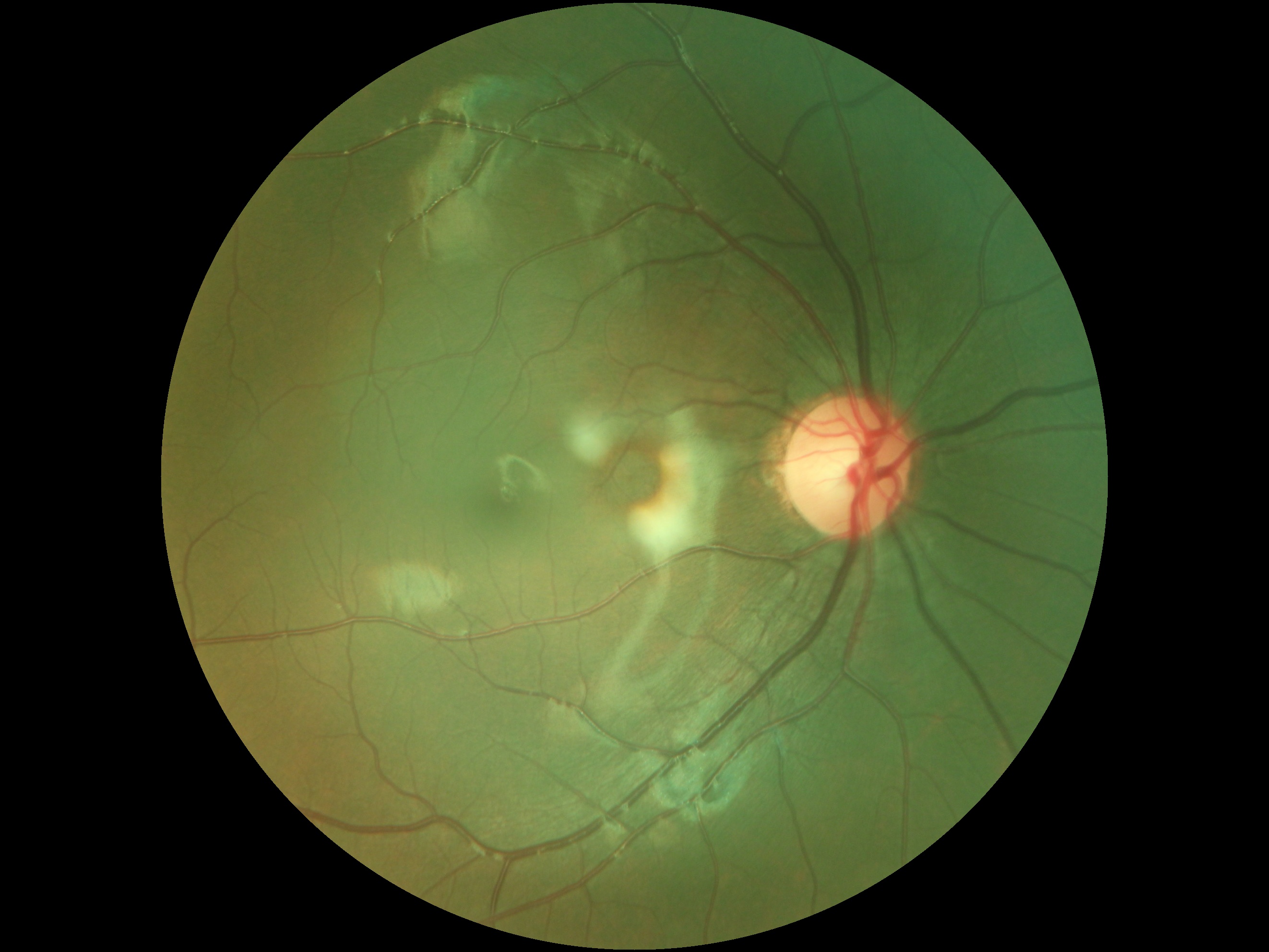

Supplement: S3 File — (ZIP) [file pone.0324352.s003.zip › Original fundus photographs (1)/Subject 19/OD_20230615893073_20230615162242_2.jpg]

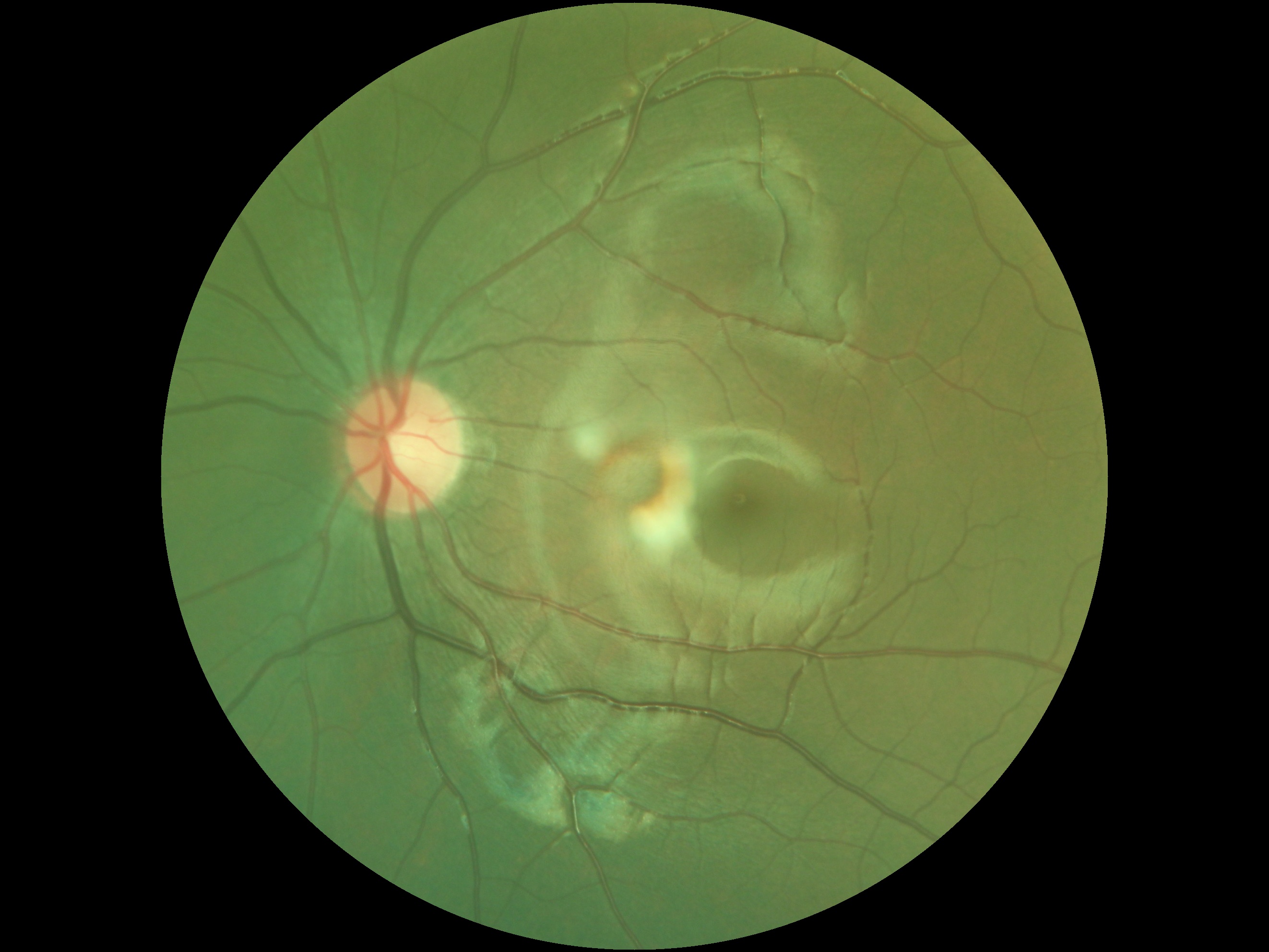

Supplement: S3 File — (ZIP) [file pone.0324352.s003.zip › Original fundus photographs (1)/Subject 19/OS_20230615893073_20230615162225_1.jpg]

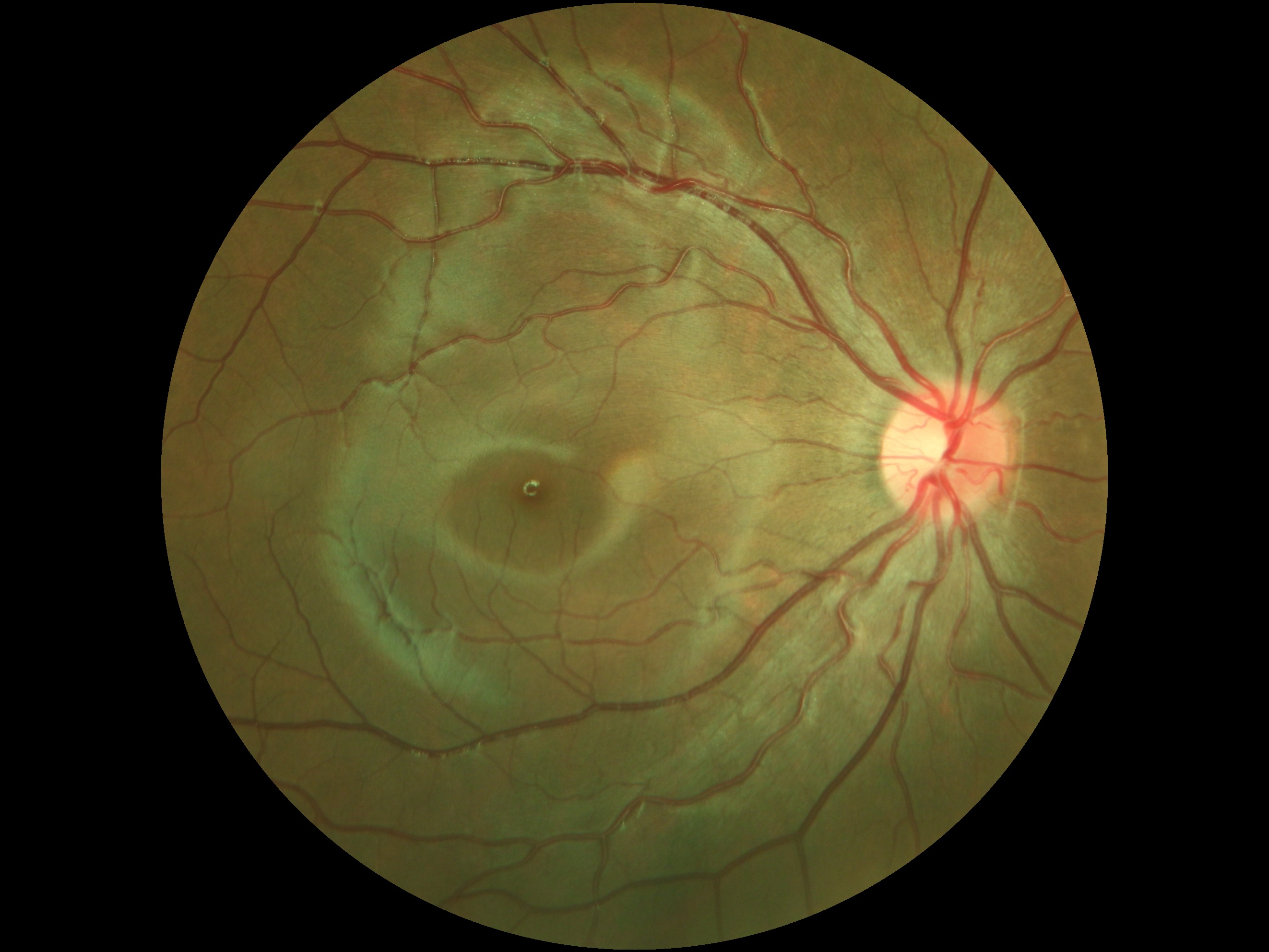

Supplement: S3 File — (ZIP) [file pone.0324352.s003.zip › Original fundus photographs (1)/Subject 2/OD_20230611512065_20230612110519_1.jpg]

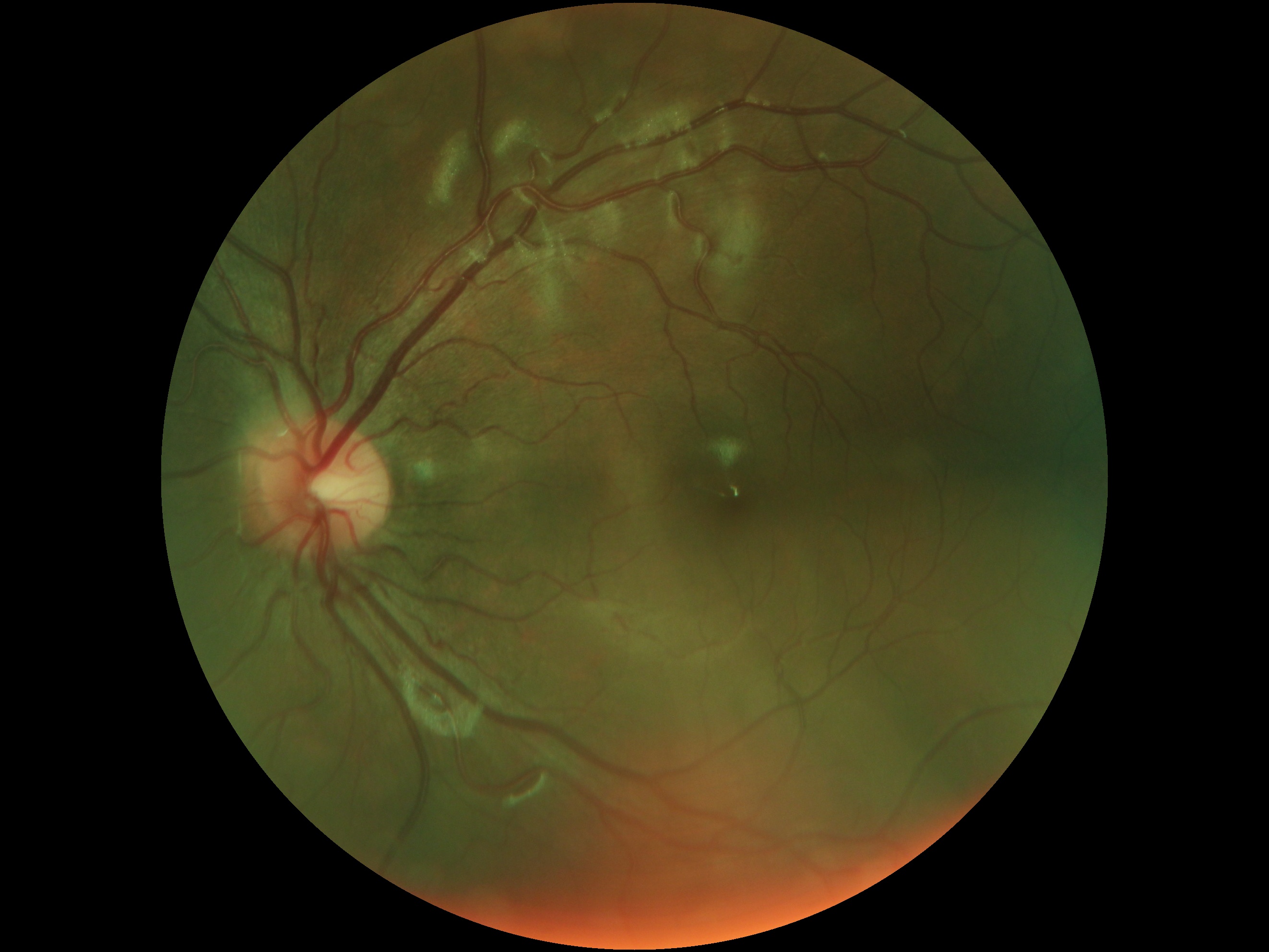

Supplement: S3 File — (ZIP) [file pone.0324352.s003.zip › Original fundus photographs (1)/Subject 2/OS_20230611512065_20230612110732_4.jpg]

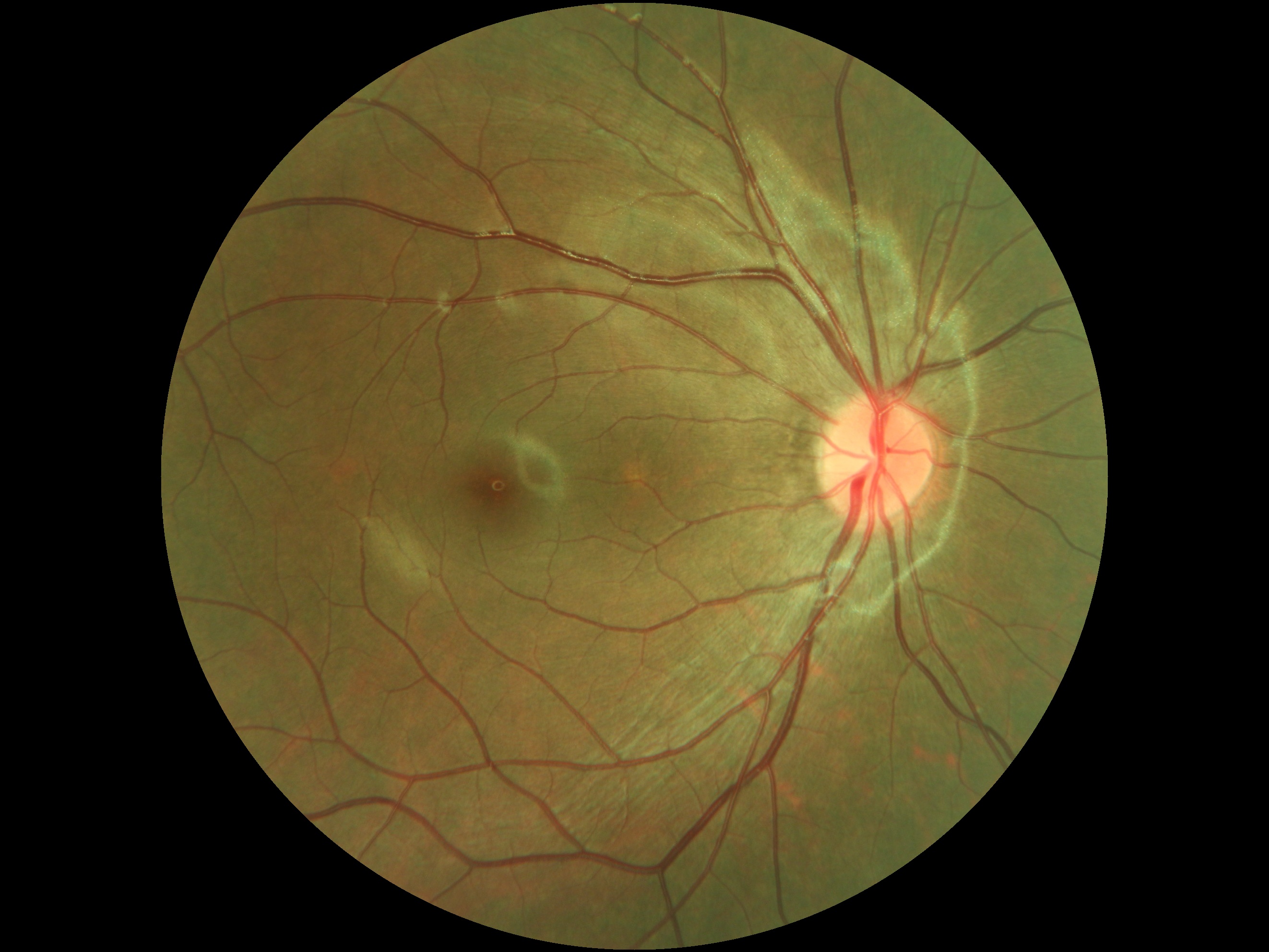

Supplement: S3 File — (ZIP) [file pone.0324352.s003.zip › Original fundus photographs (1)/Subject 20/OD_20230611384066_20230612162031_1.jpg]

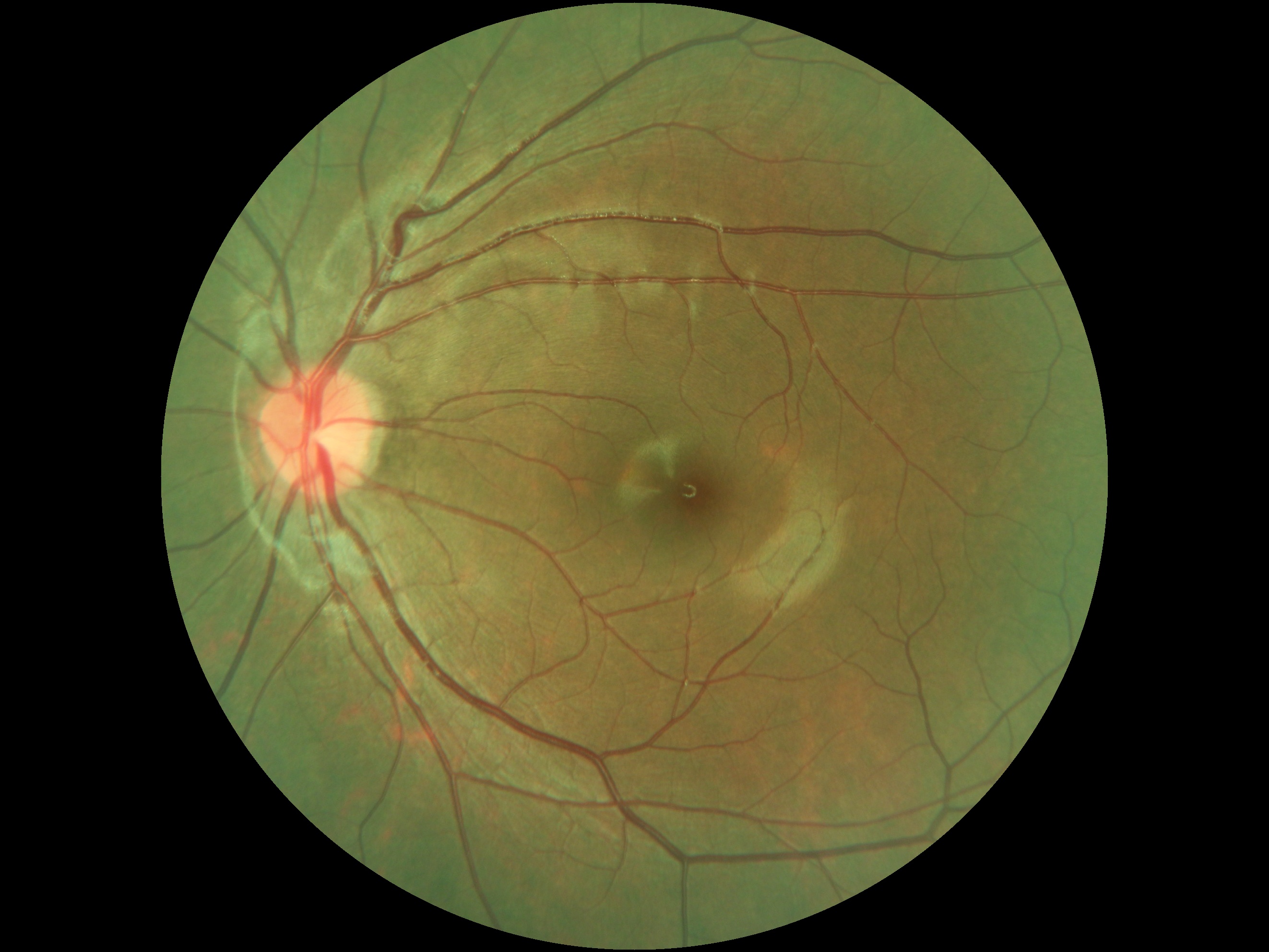

Supplement: S3 File — (ZIP) [file pone.0324352.s003.zip › Original fundus photographs (1)/Subject 20/OS_20230611384066_20230612162100_2.jpg]

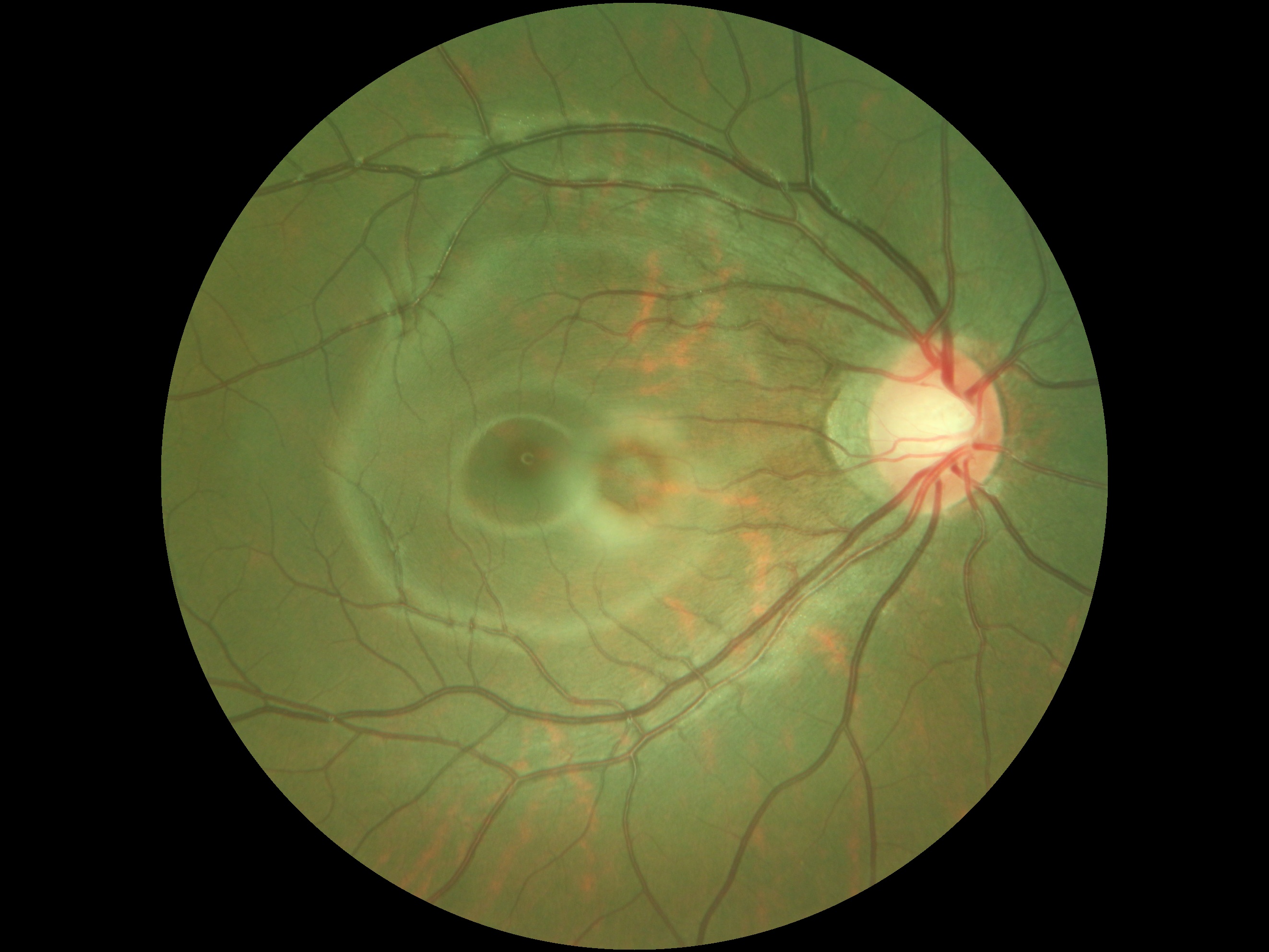

Supplement: S3 File — (ZIP) [file pone.0324352.s003.zip › Original fundus photographs (1)/Subject 21/OD_20230611904176_20230614103015_1.jpg]

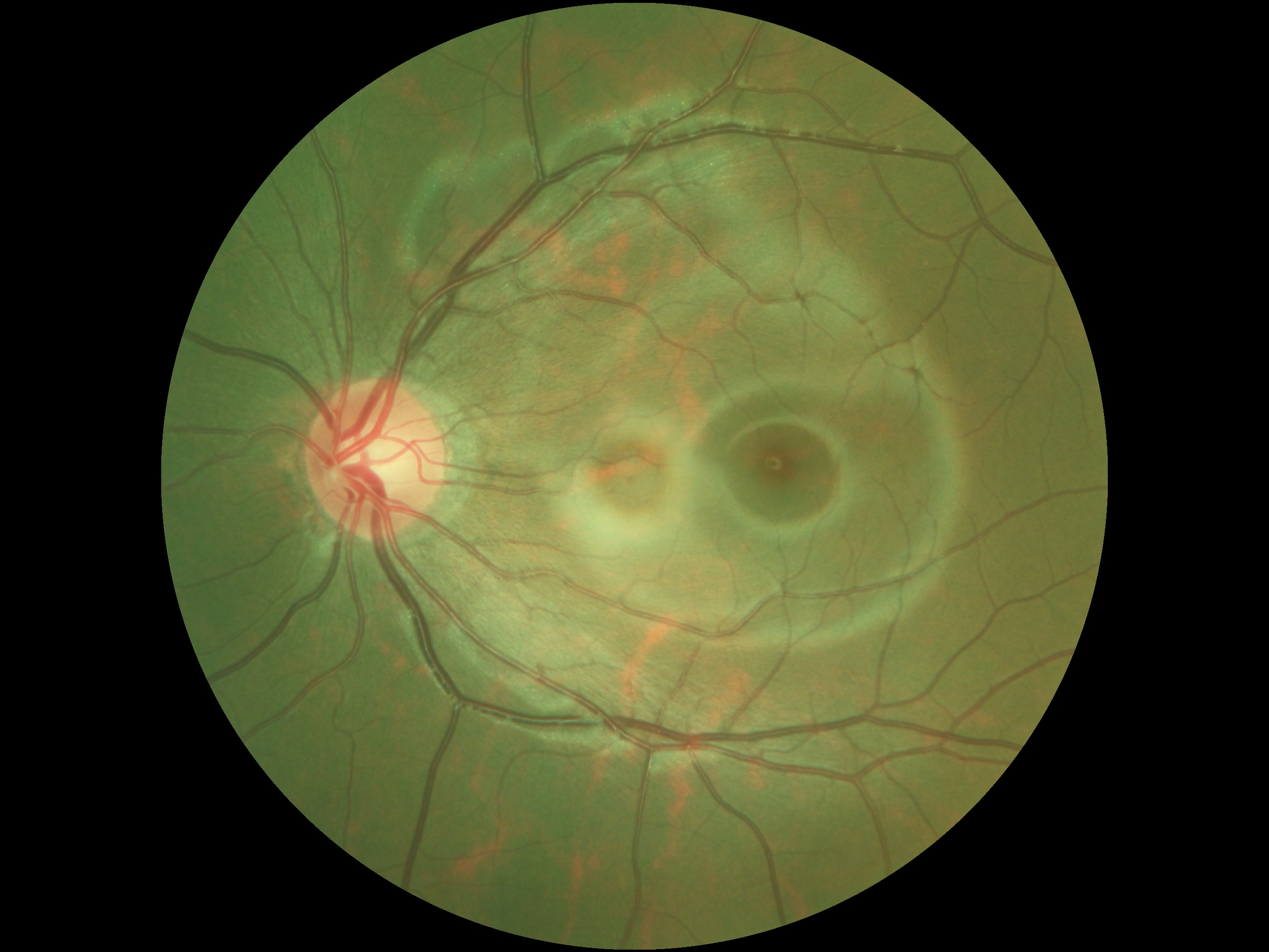

Supplement: S3 File — (ZIP) [file pone.0324352.s003.zip › Original fundus photographs (1)/Subject 21/OS_20230611904176_20230614103132_2.jpg]

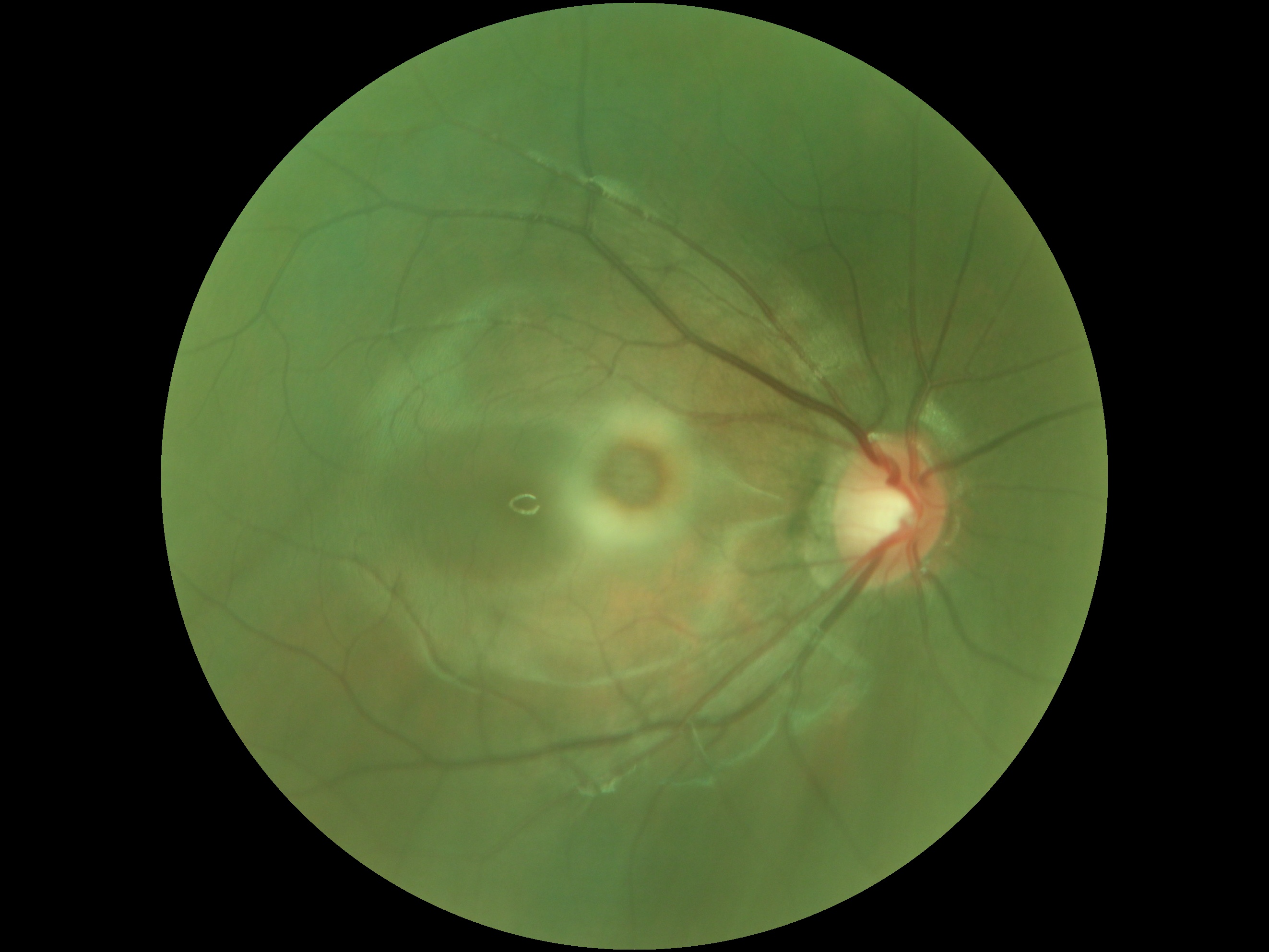

Supplement: S3 File — (ZIP) [file pone.0324352.s003.zip › Original fundus photographs (1)/Subject 22/OD_20230611009274_20230615104702_1.jpg]

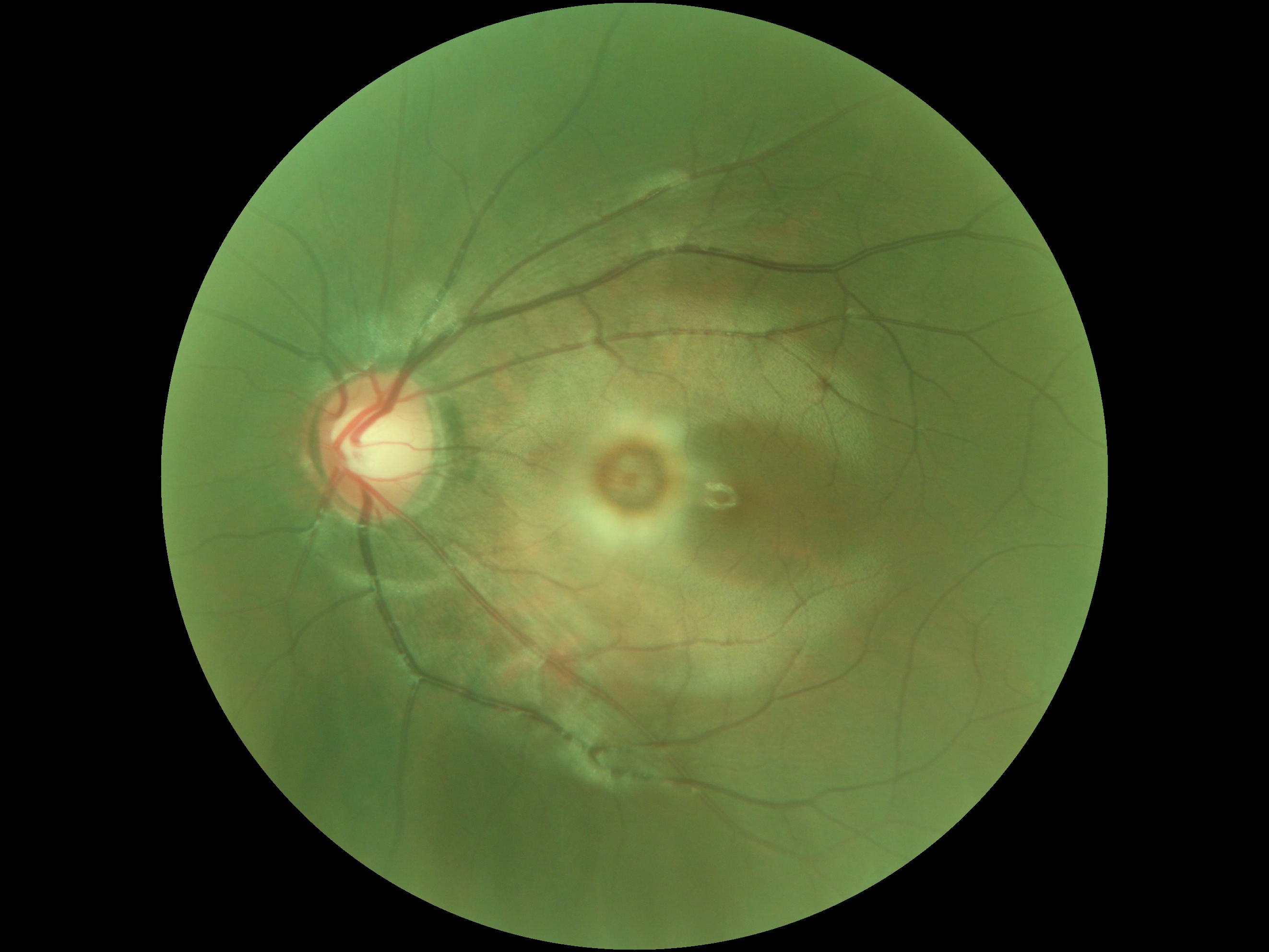

Supplement: S3 File — (ZIP) [file pone.0324352.s003.zip › Original fundus photographs (1)/Subject 22/OS_20230611009274_20230615104757_3.jpg]

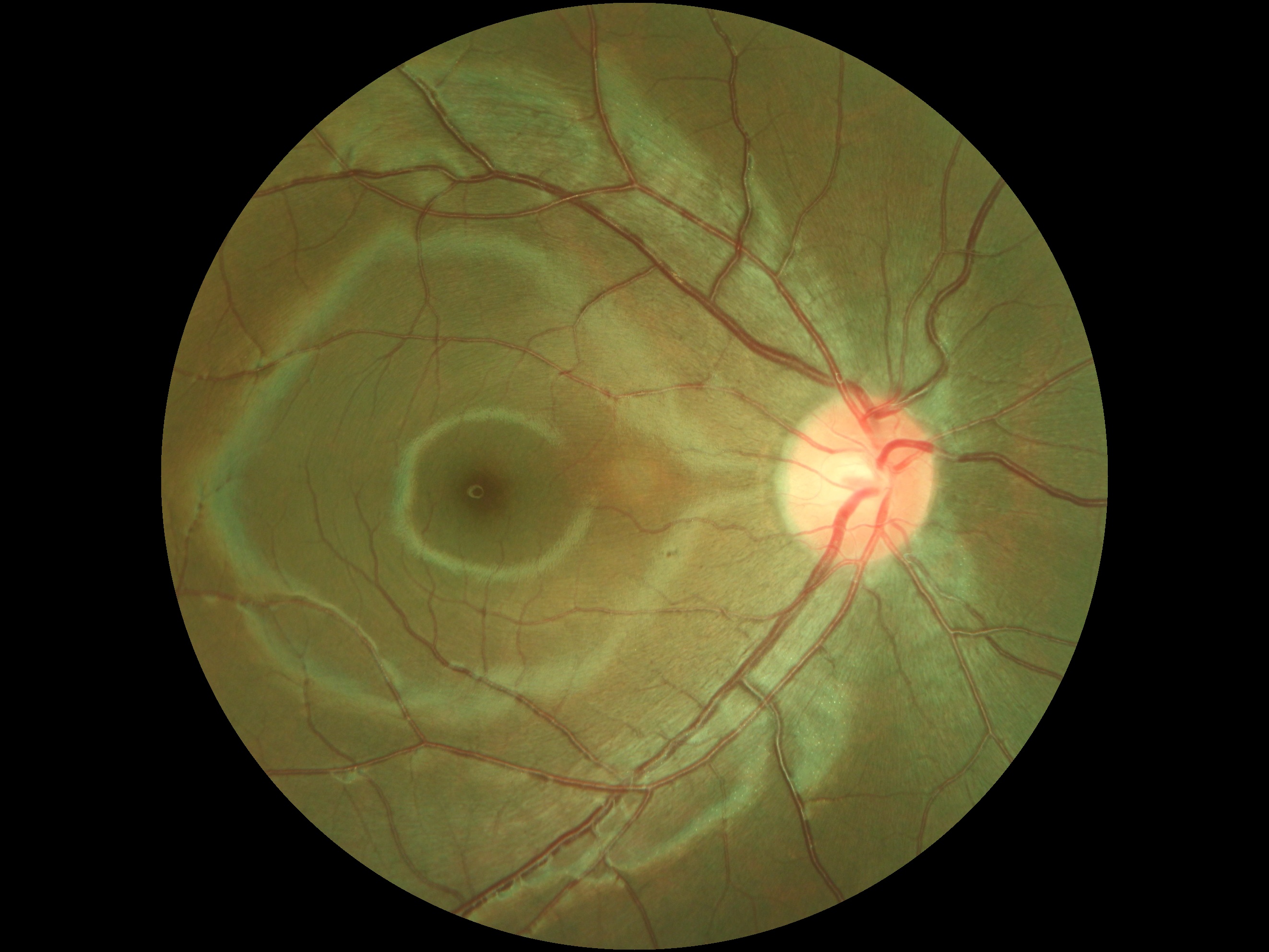

Supplement: S3 File — (ZIP) [file pone.0324352.s003.zip › Original fundus photographs (1)/Subject 23/OD_20230611484067_20230612162316_1.jpg]

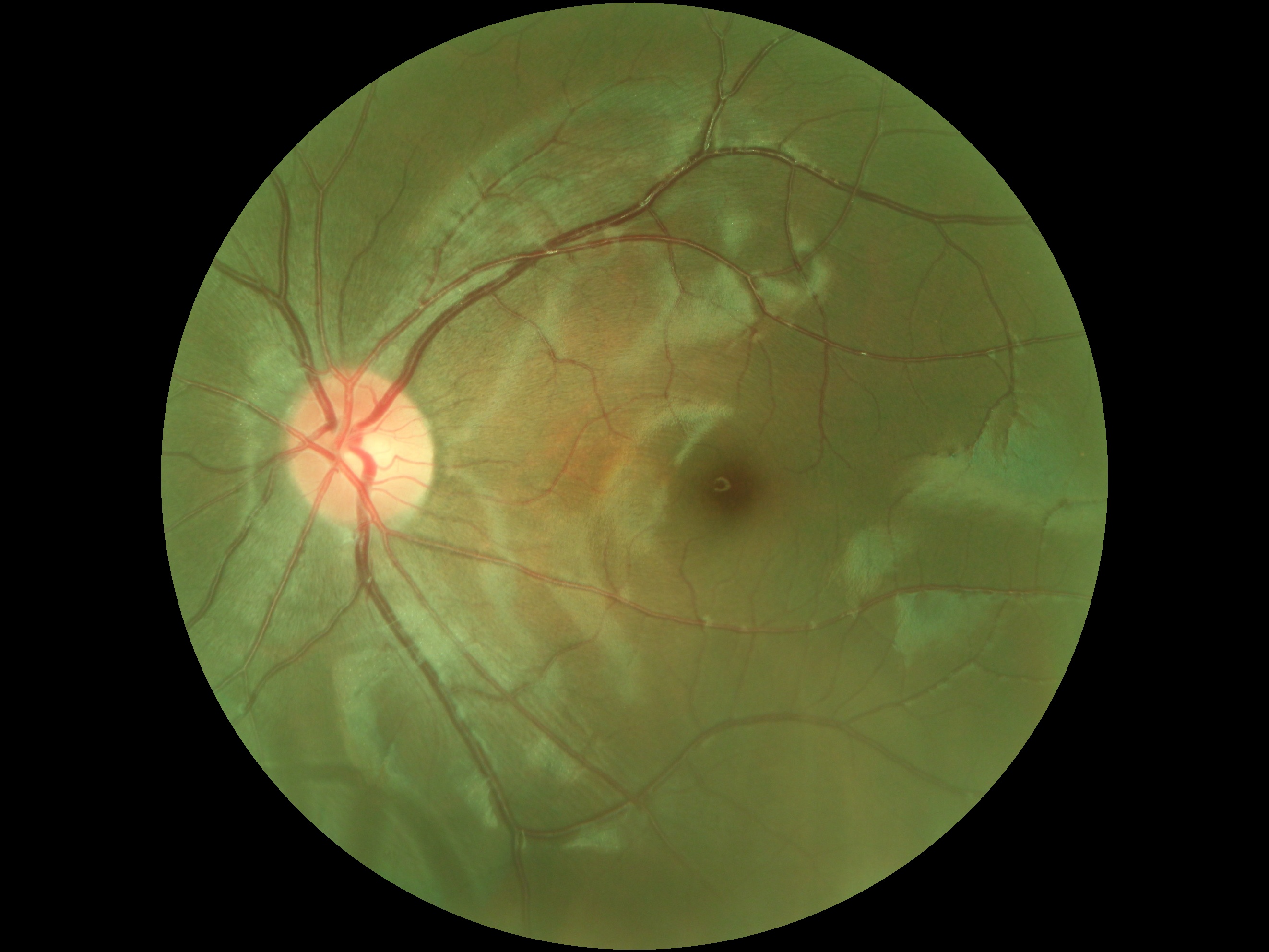

Supplement: S3 File — (ZIP) [file pone.0324352.s003.zip › Original fundus photographs (1)/Subject 23/OS_20230611484067_20230612162341_2.jpg]

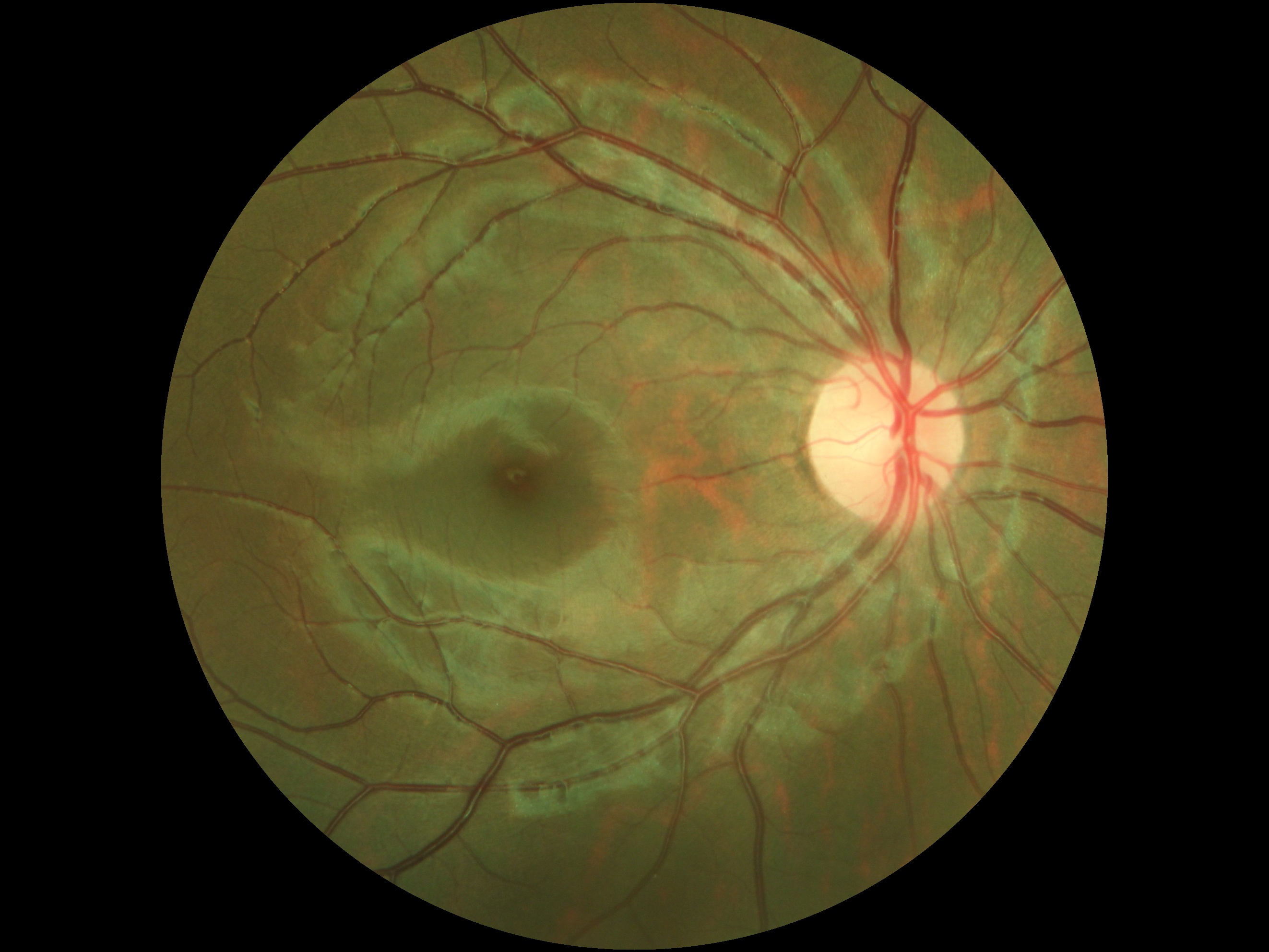

Supplement: S3 File — (ZIP) [file pone.0324352.s003.zip › Original fundus photographs (1)/Subject 24/OD_20230611876068_20230612110237_1.jpg]

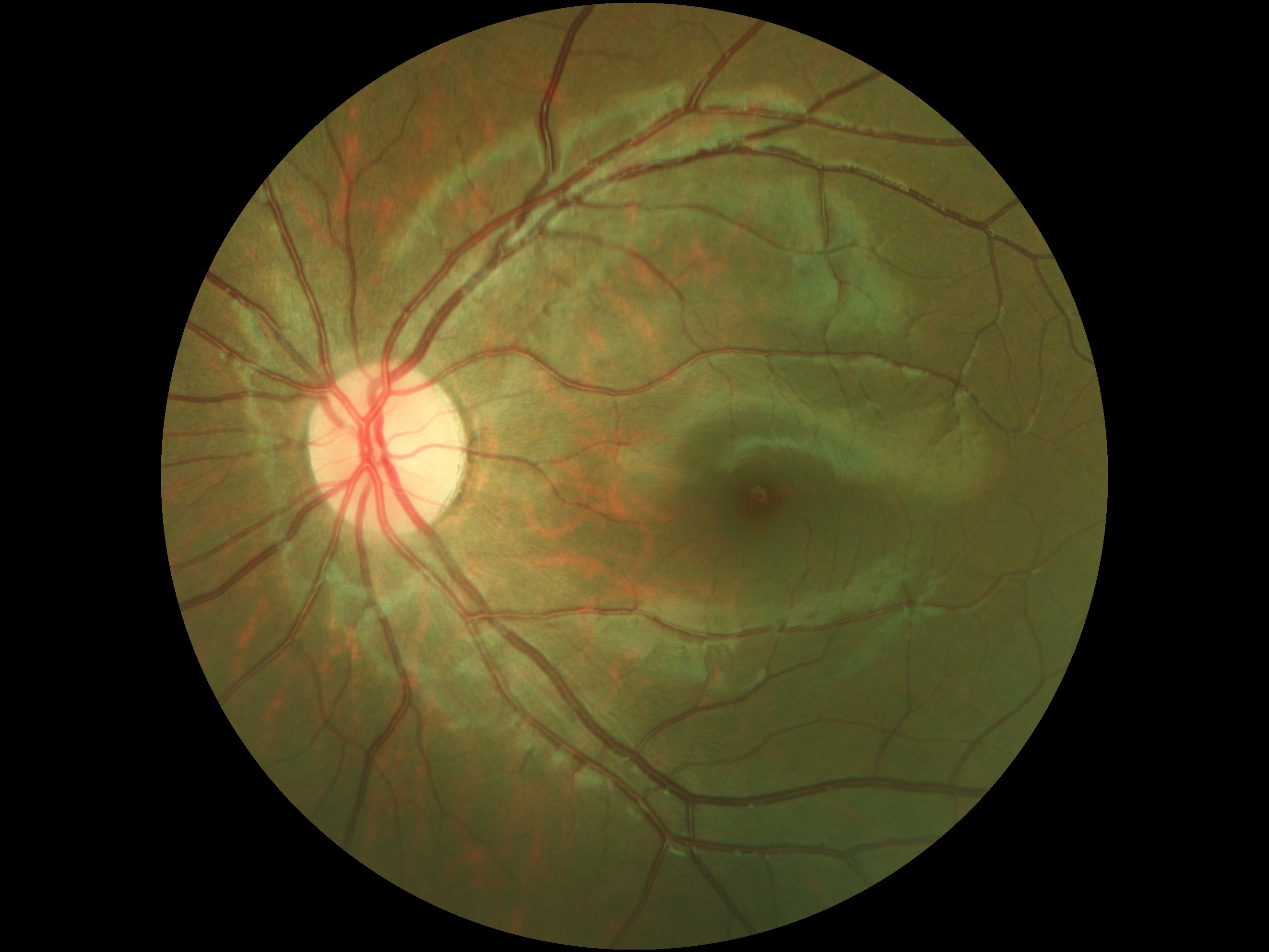

Supplement: S3 File — (ZIP) [file pone.0324352.s003.zip › Original fundus photographs (1)/Subject 24/OS_20230611876068_20230612110323_2.jpg]

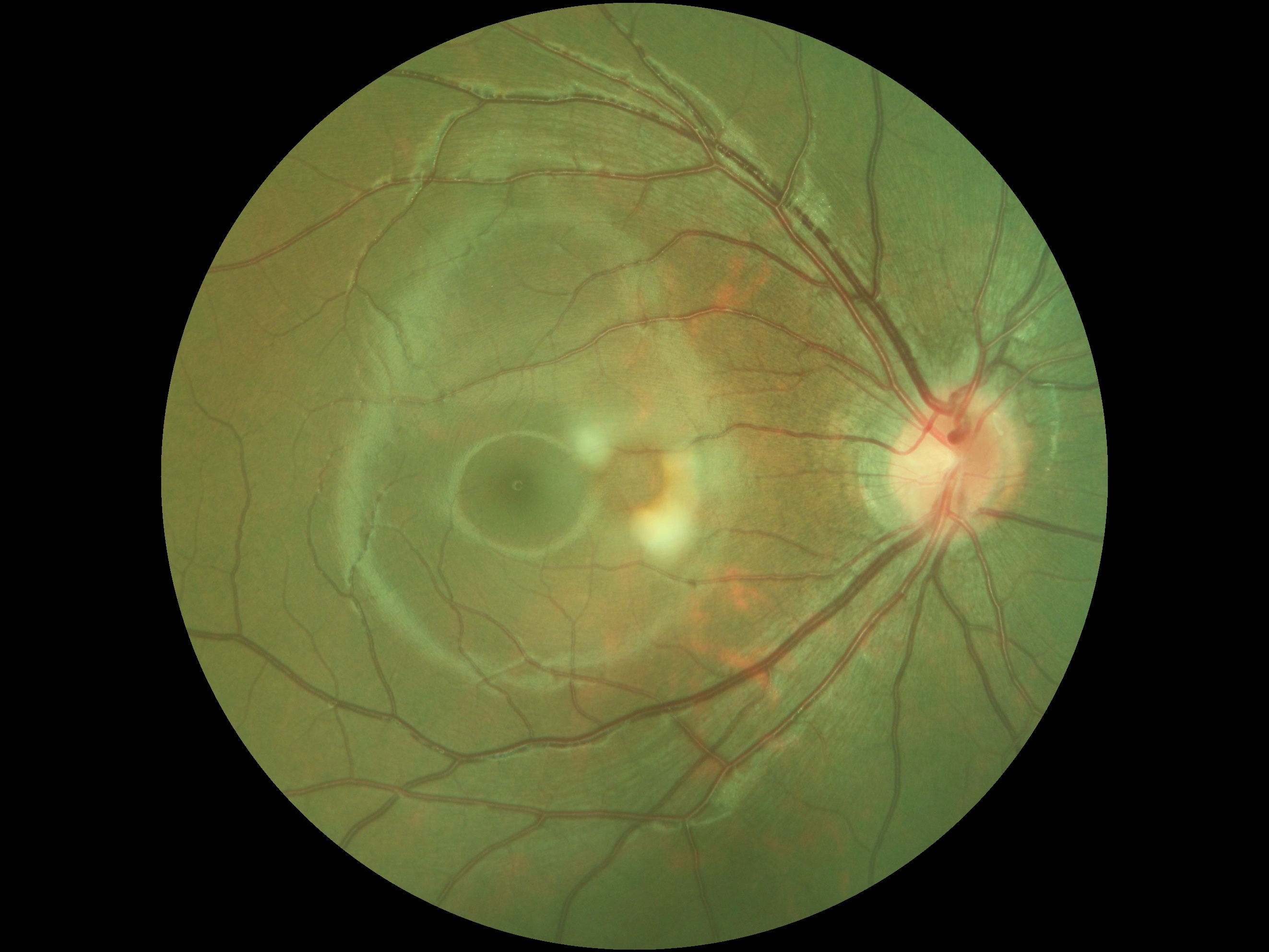

Supplement: S3 File — (ZIP) [file pone.0324352.s003.zip › Original fundus photographs (1)/Subject 25/OD_20230611829054_20230615165832_1.jpg]

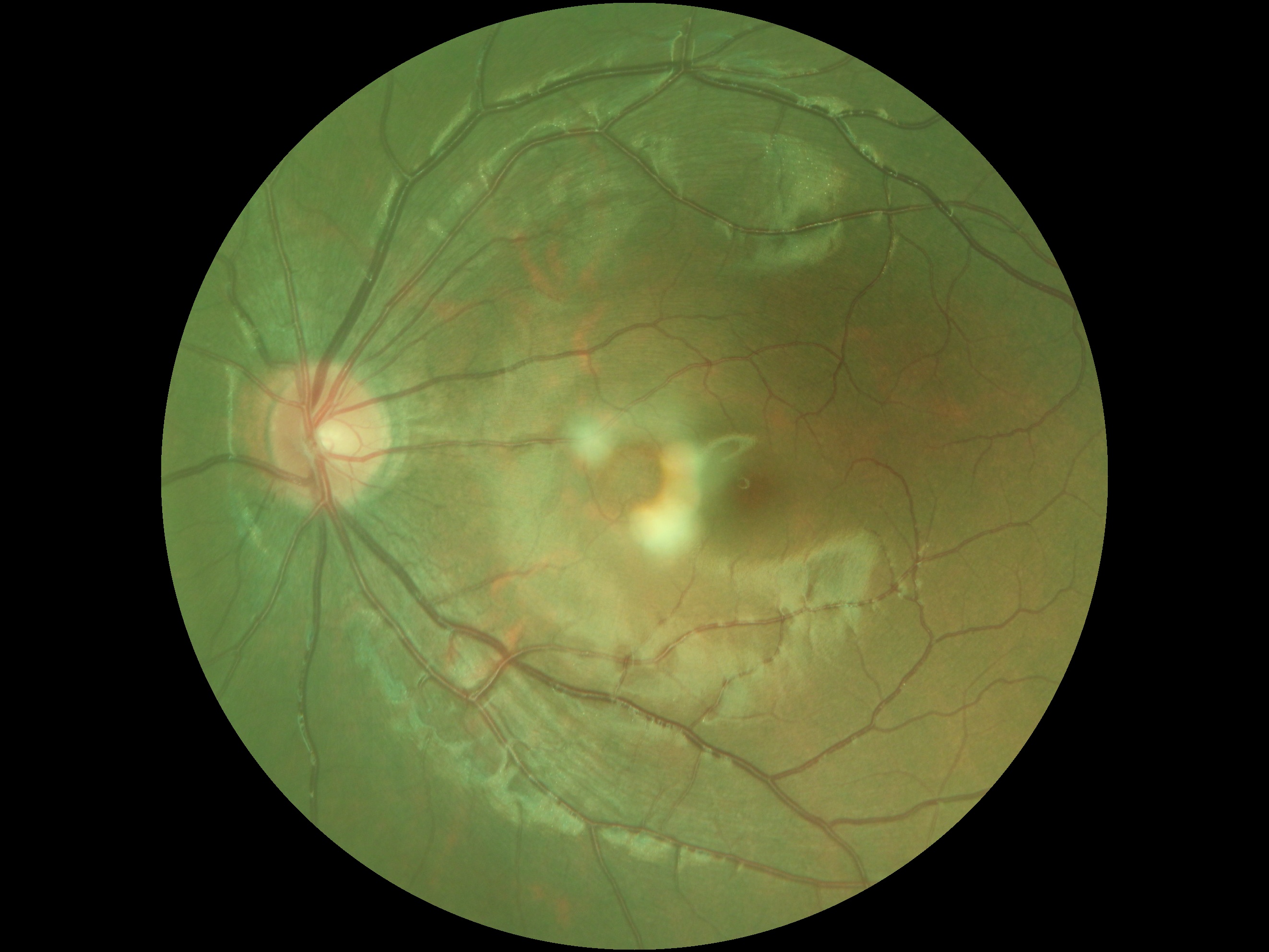

Supplement: S3 File — (ZIP) [file pone.0324352.s003.zip › Original fundus photographs (1)/Subject 25/OS_20230611829054_20230615165857_2.jpg]

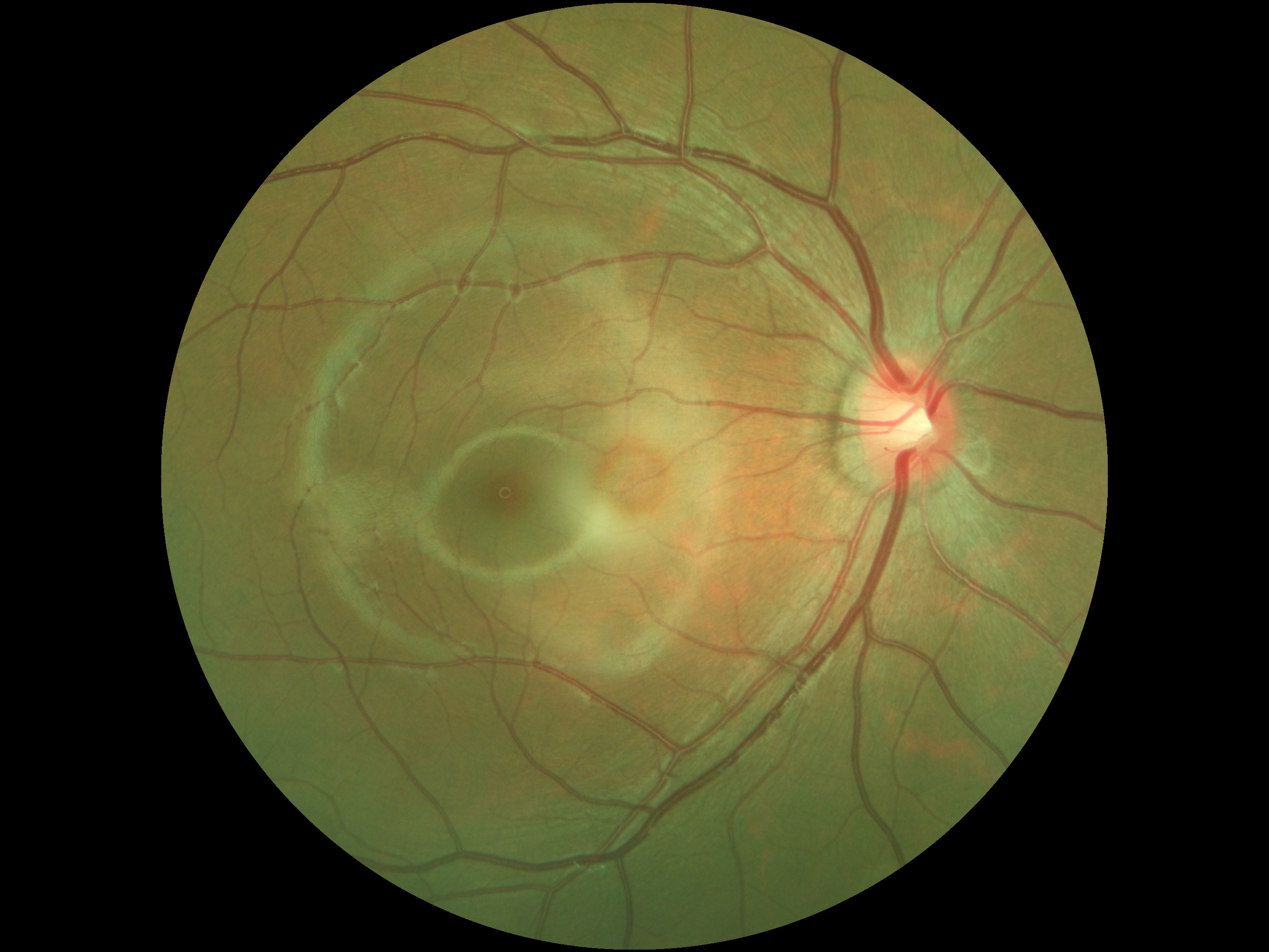

Supplement: S3 File — (ZIP) [file pone.0324352.s003.zip › Original fundus photographs (1)/Subject 26/OD_20230613156061_20230614161632_2.jpg]

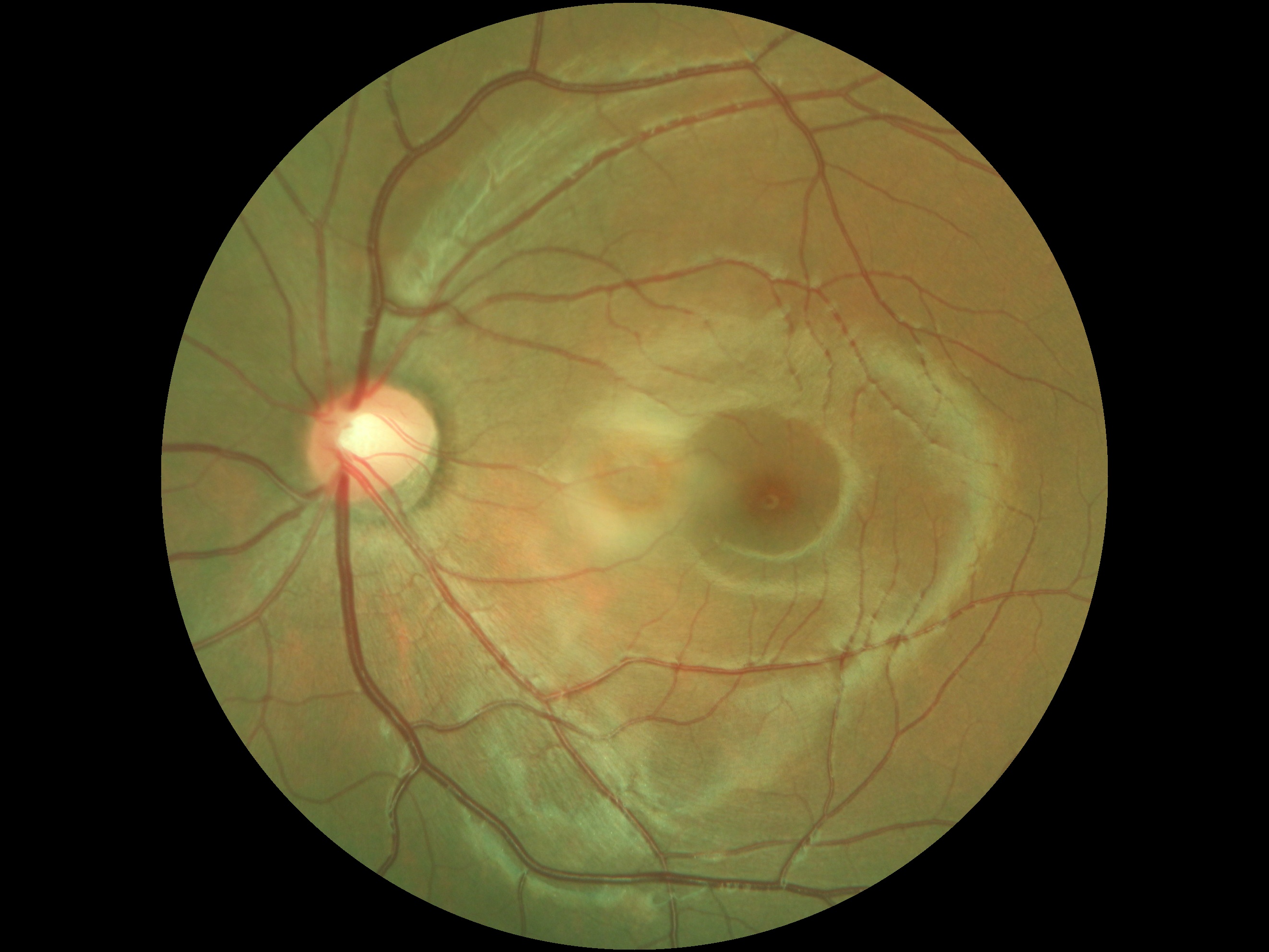

Supplement: S3 File — (ZIP) [file pone.0324352.s003.zip › Original fundus photographs (1)/Subject 26/OS_20230613156061_20230614161615_1.jpg]

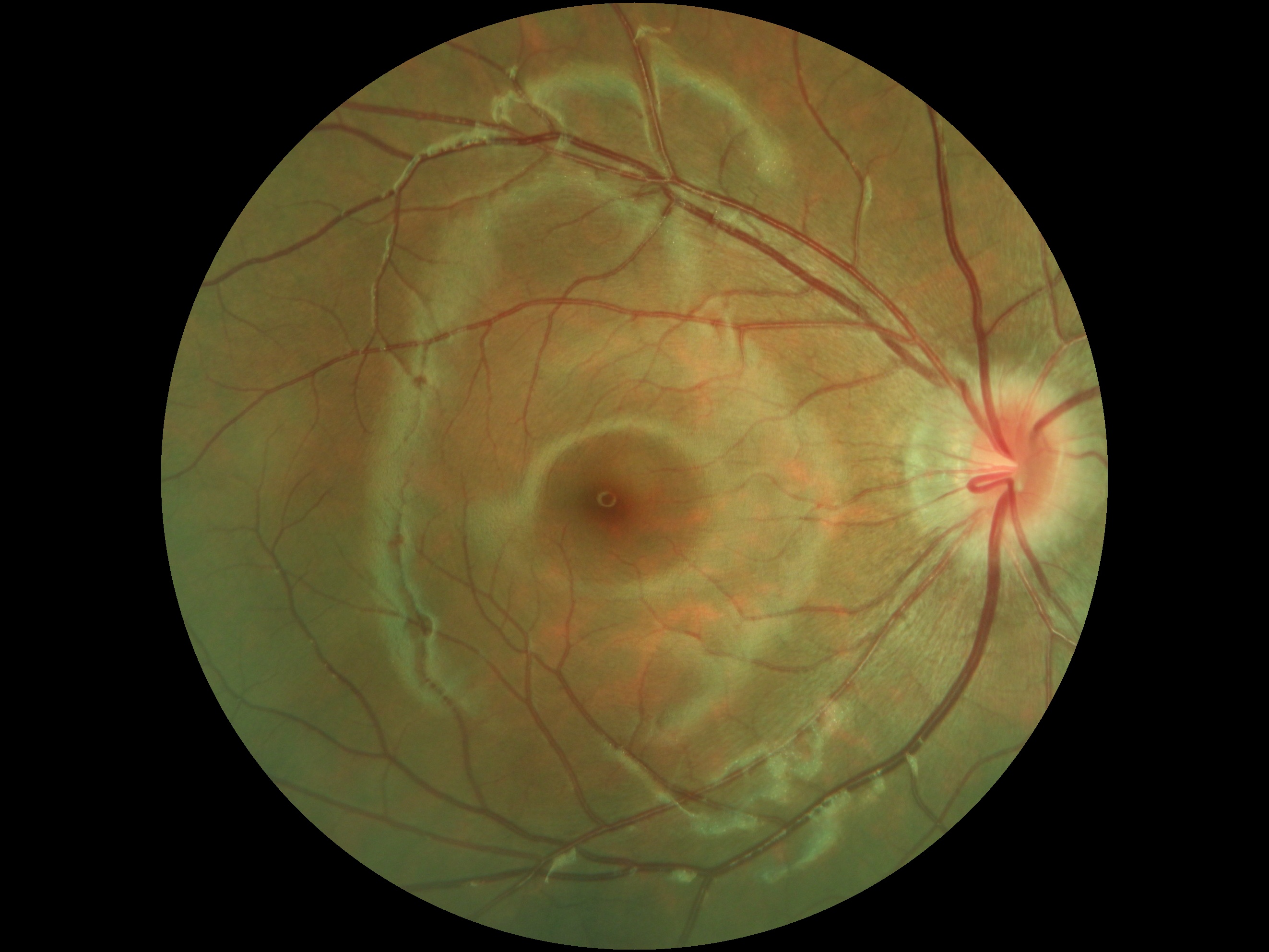

Supplement: S3 File — (ZIP) [file pone.0324352.s003.zip › Original fundus photographs (1)/Subject 27/OD_20230611503070_20230612103041_1.jpg]

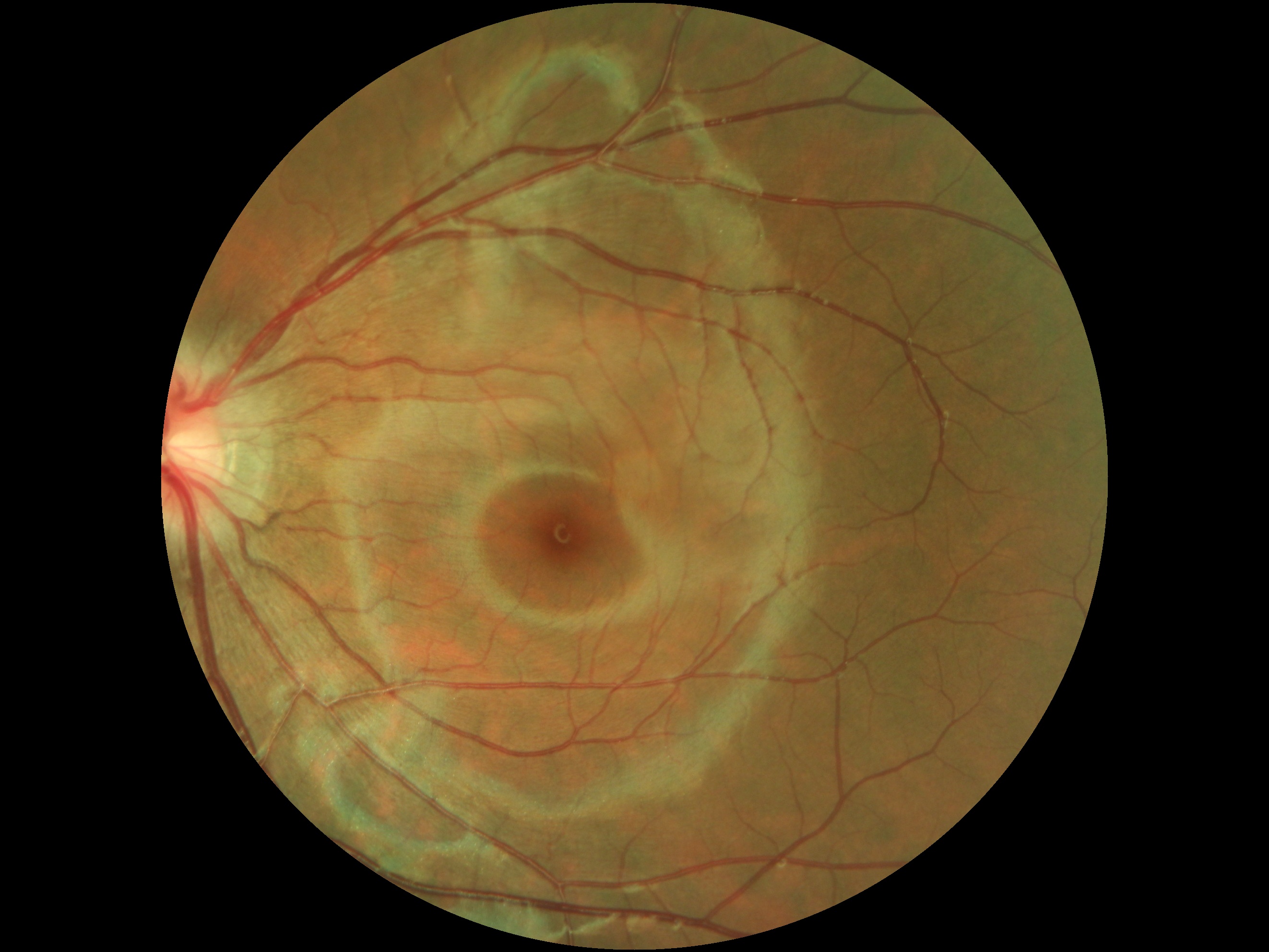

Supplement: S3 File — (ZIP) [file pone.0324352.s003.zip › Original fundus photographs (1)/Subject 27/OS_20230611503070_20230612103310_2.jpg]

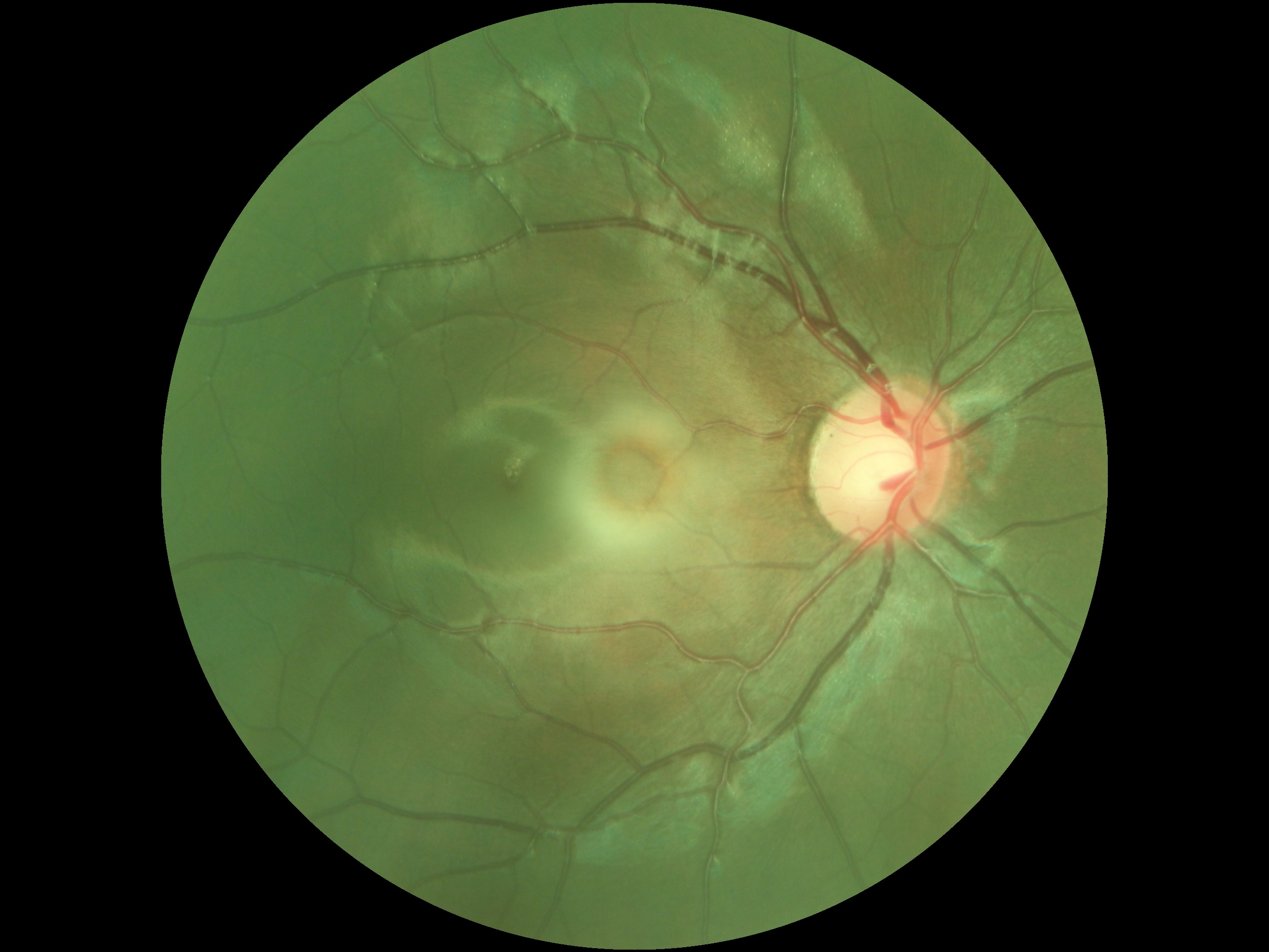

Supplement: S3 File — (ZIP) [file pone.0324352.s003.zip › Original fundus photographs (1)/Subject 28/OD_20230611715114_20230614104841_3.jpg]

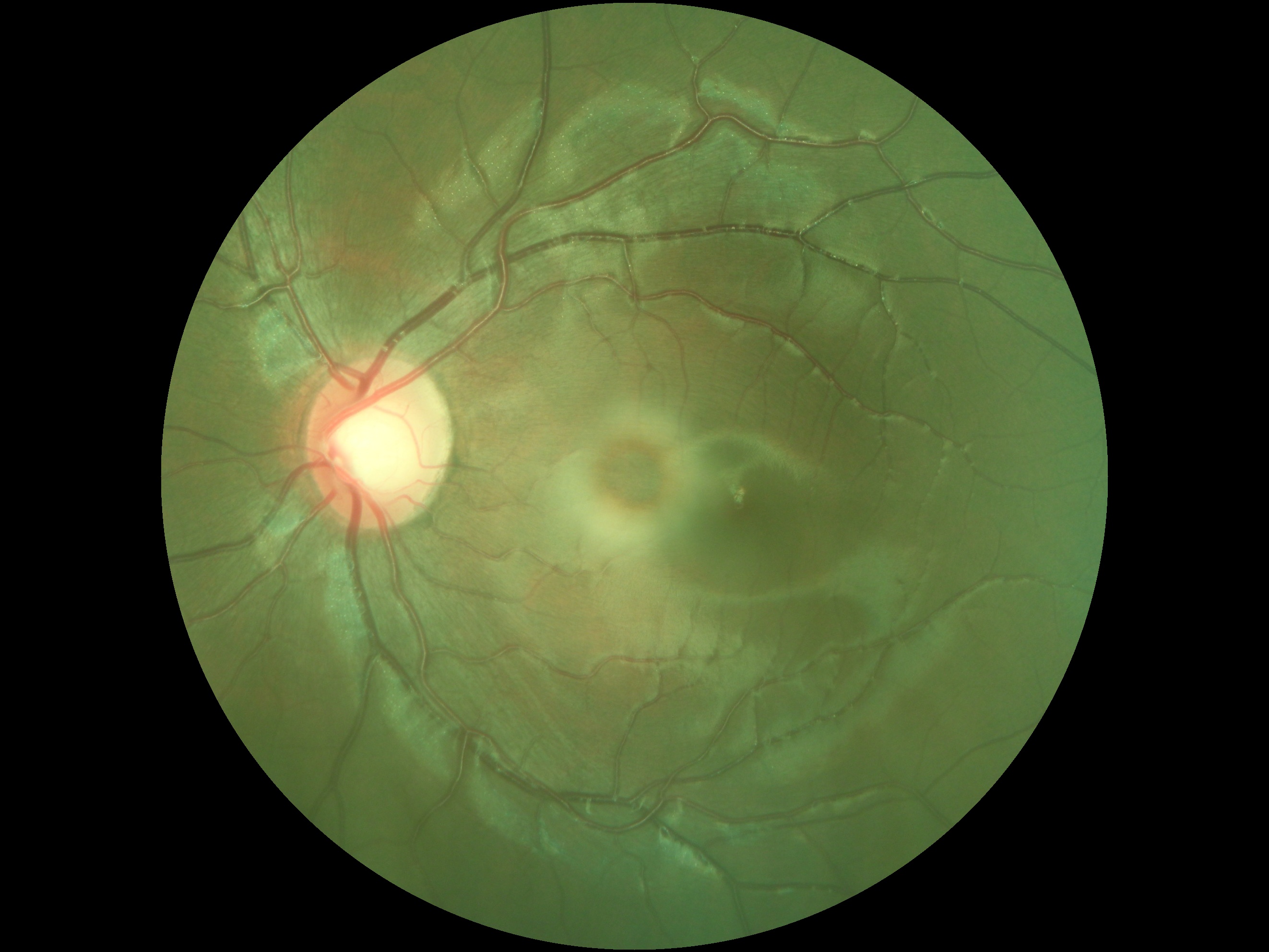

Supplement: S3 File — (ZIP) [file pone.0324352.s003.zip › Original fundus photographs (1)/Subject 28/OS_20230611715114_20230614104637_1.jpg]

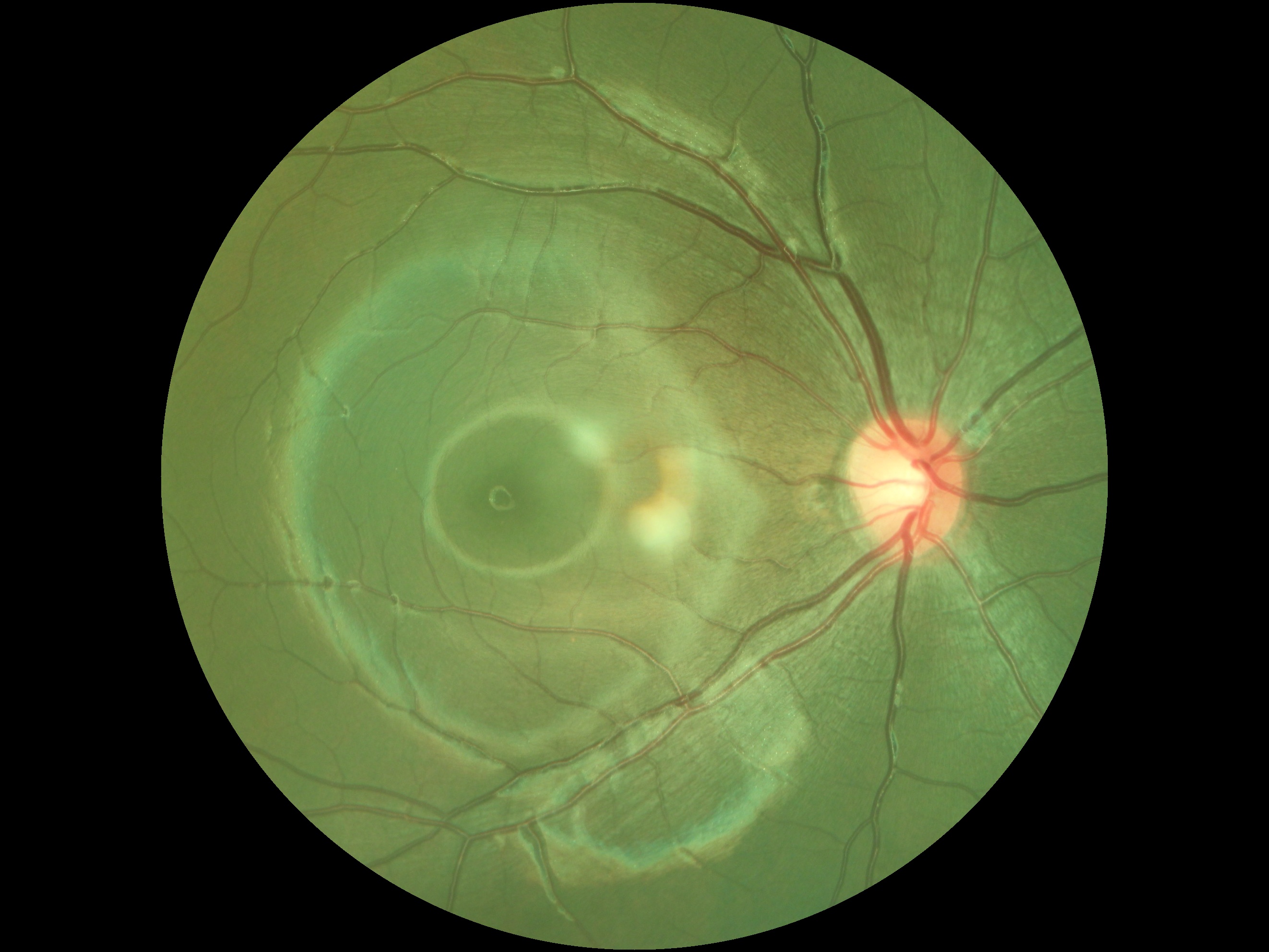

Supplement: S3 File — (ZIP) [file pone.0324352.s003.zip › Original fundus photographs (1)/Subject 29/OD_20230615417074_20230615162358_1.jpg]

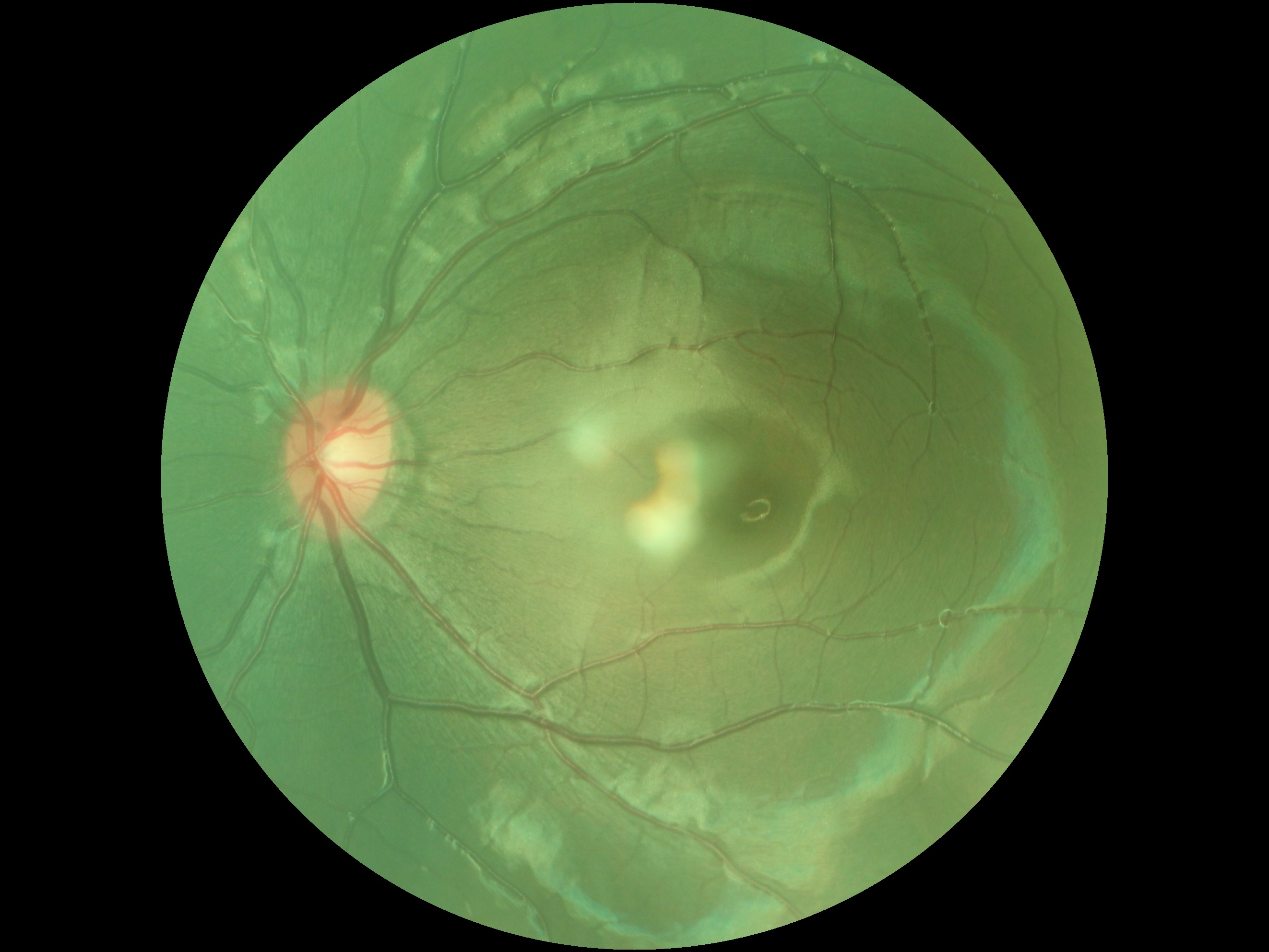

Supplement: S3 File — (ZIP) [file pone.0324352.s003.zip › Original fundus photographs (1)/Subject 29/OS_20230615417074_20230615162432_2.jpg]

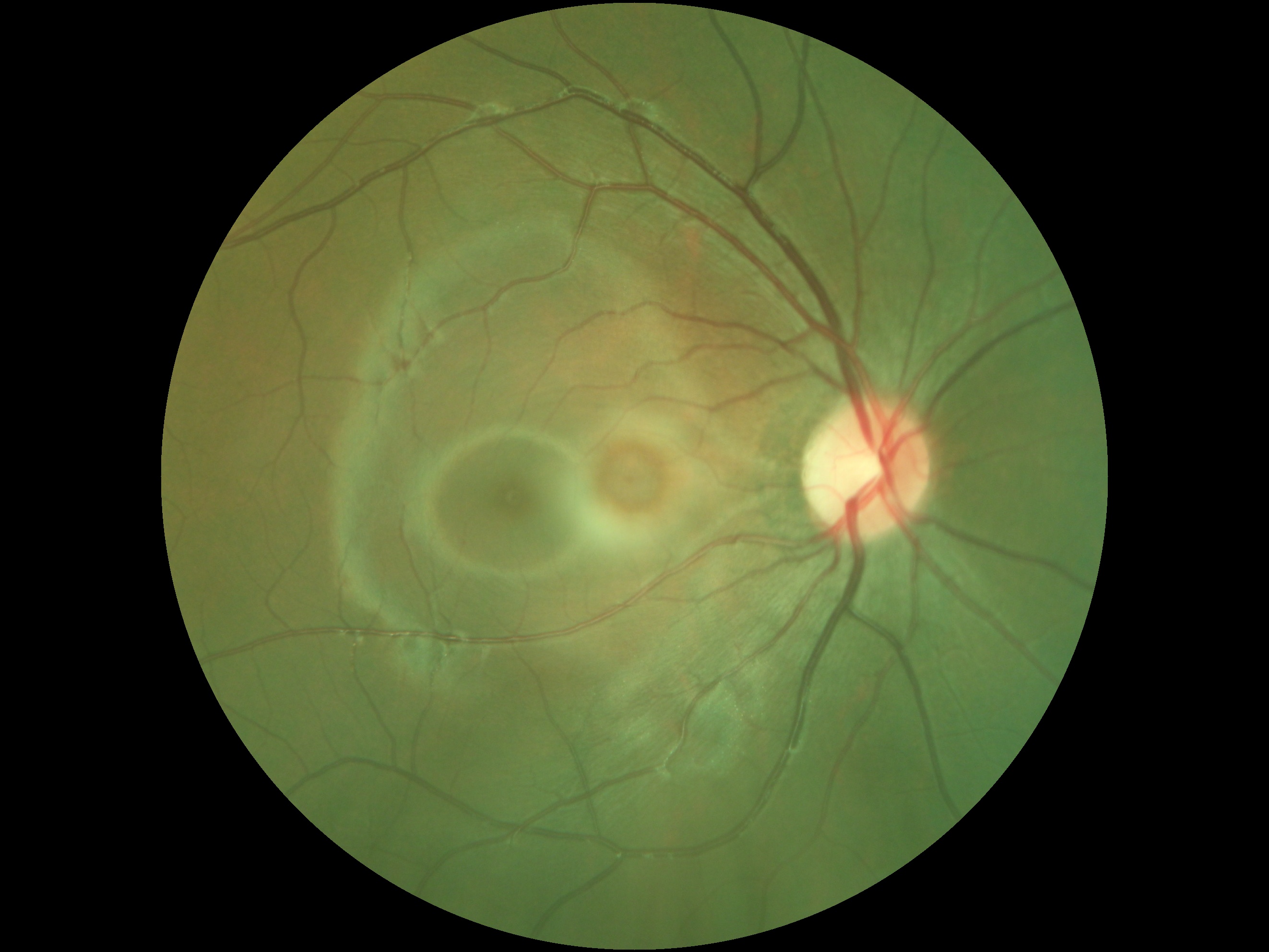

Supplement: S3 File — (ZIP) [file pone.0324352.s003.zip › Original fundus photographs (1)/Subject 3/OD_20230611566174_20230614103503_3.jpg]

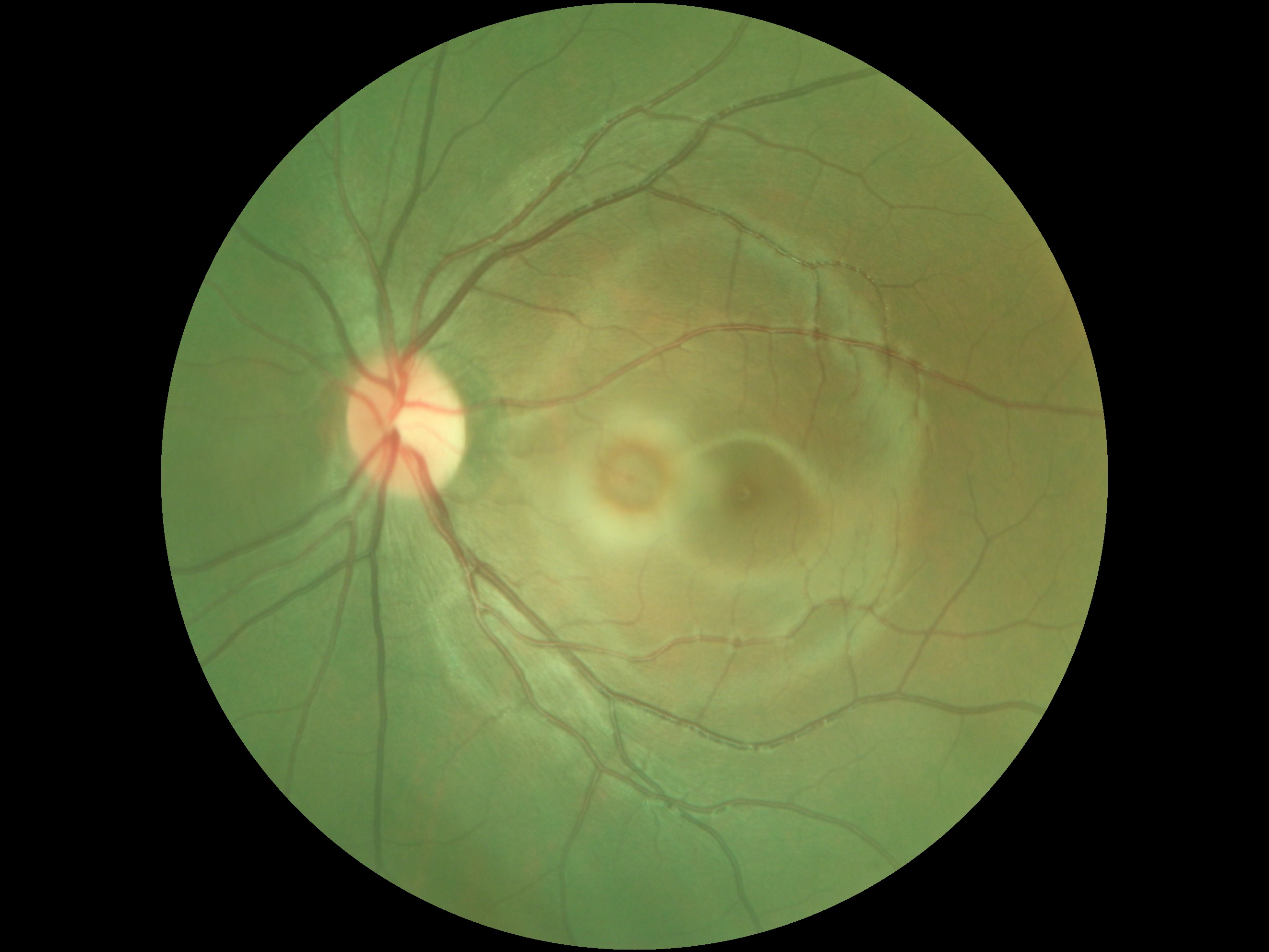

Supplement: S3 File — (ZIP) [file pone.0324352.s003.zip › Original fundus photographs (1)/Subject 3/OS_20230611566174_20230614103435_2.jpg]

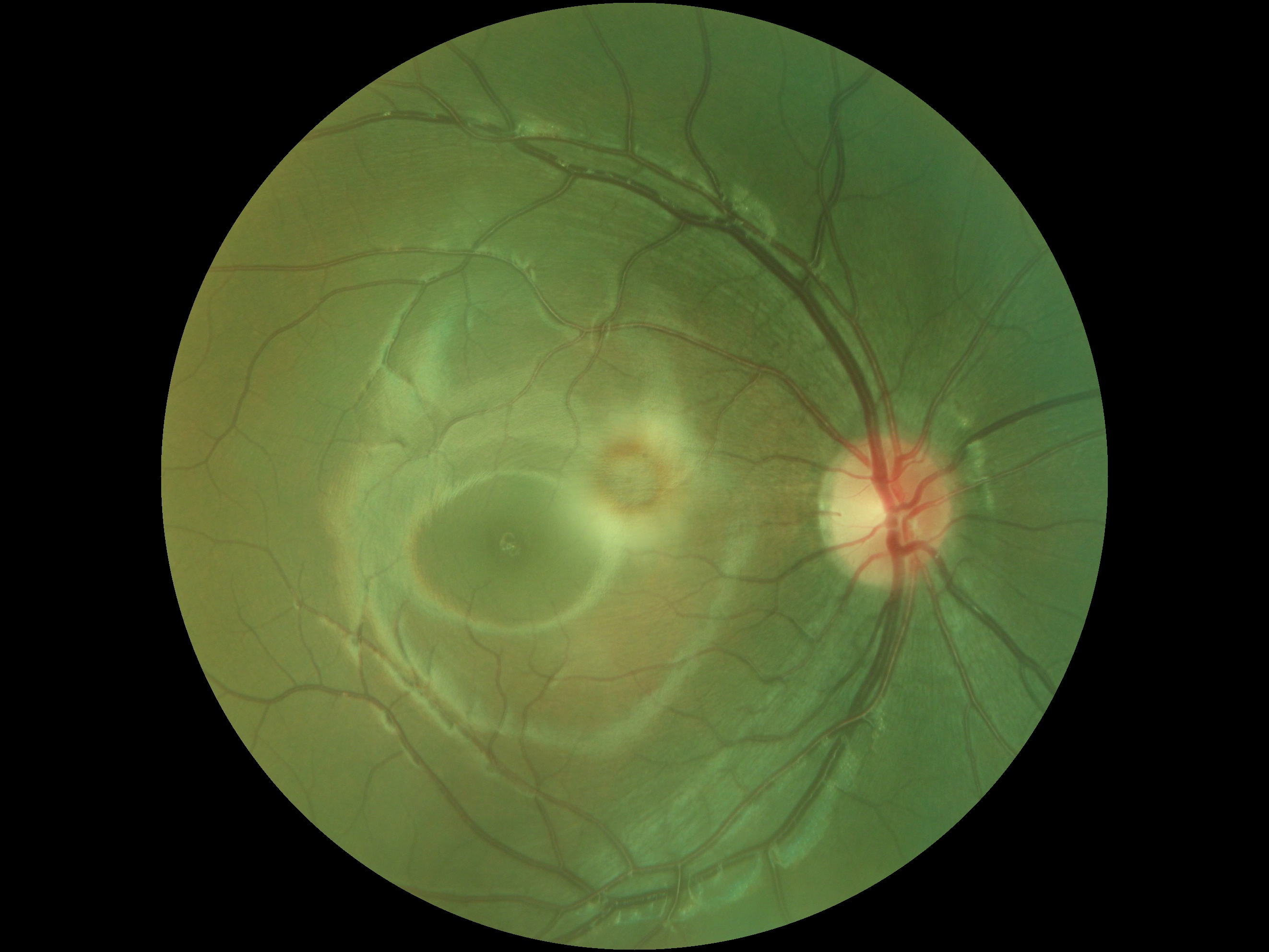

Supplement: S3 File — (ZIP) [file pone.0324352.s003.zip › Original fundus photographs (1)/Subject 30/OD_20230615669069_20230615154648_1.jpg]

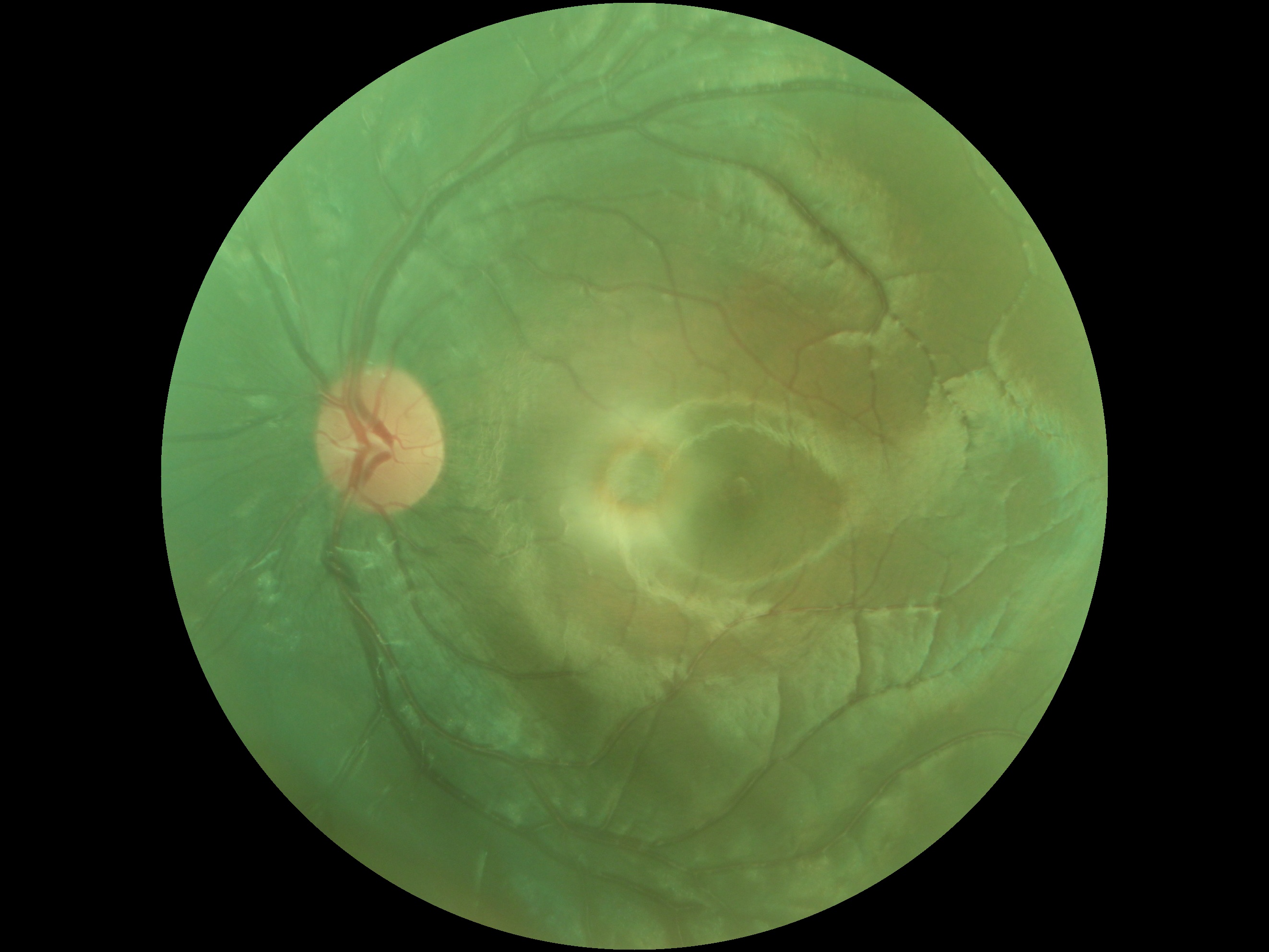

Supplement: S3 File — (ZIP) [file pone.0324352.s003.zip › Original fundus photographs (1)/Subject 30/OS_20230615669069_20230615154751_2.jpg]

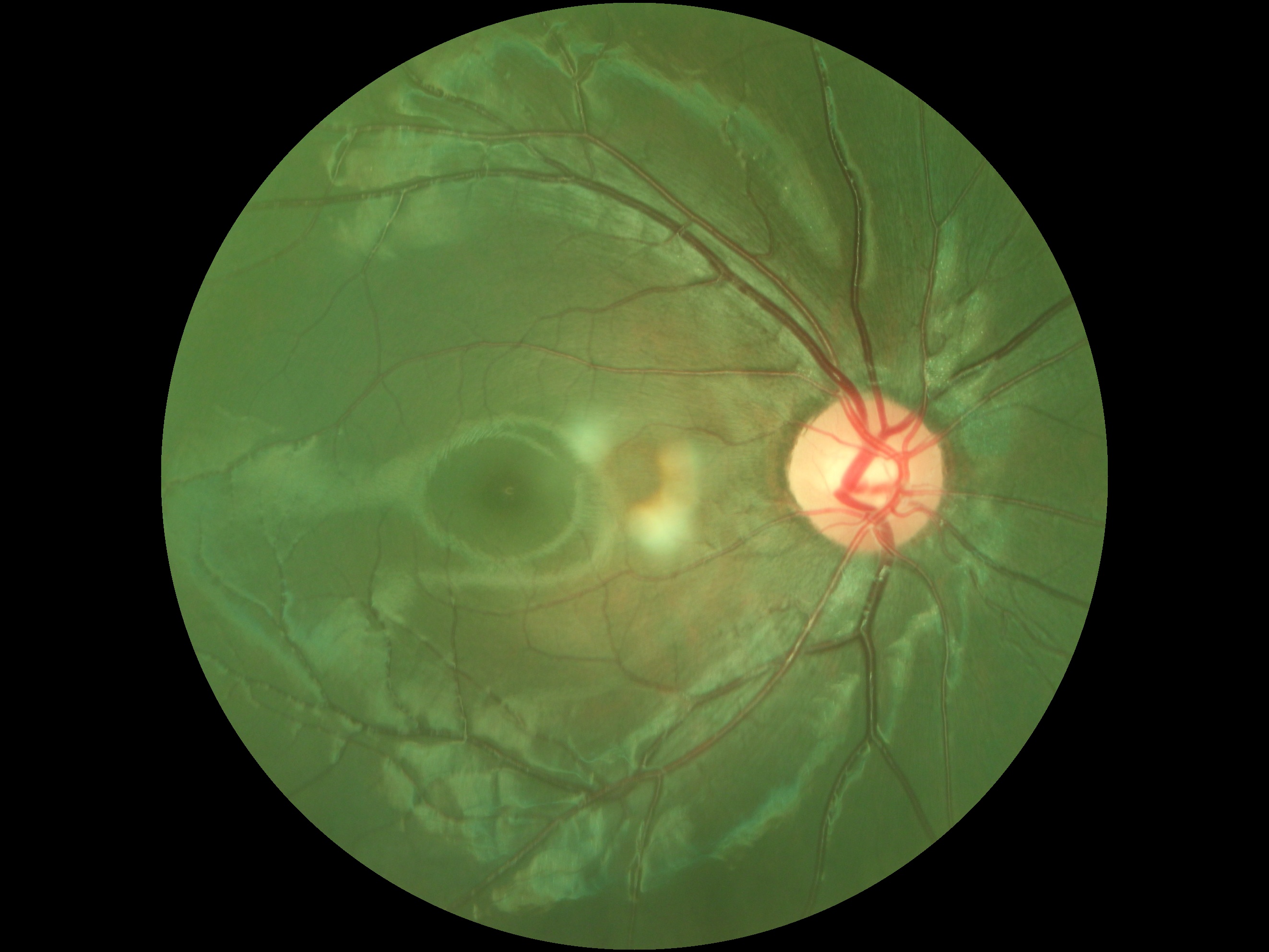

Supplement: S3 File — (ZIP) [file pone.0324352.s003.zip › Original fundus photographs (1)/Subject 31/OD_20230615721075_20230615161856_2.jpg]

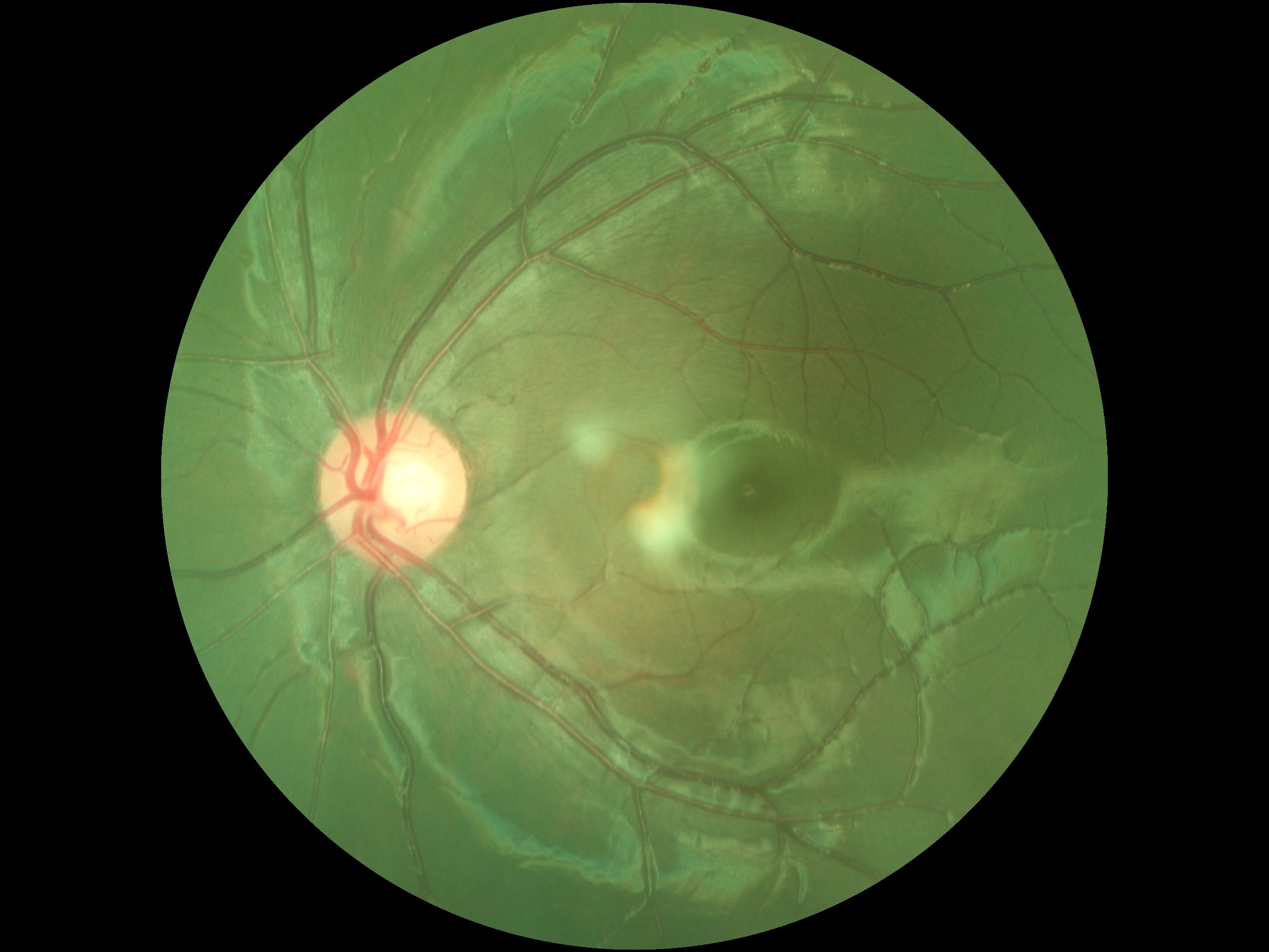

Supplement: S3 File — (ZIP) [file pone.0324352.s003.zip › Original fundus photographs (1)/Subject 31/OS_20230615721075_20230615161841_1.jpg]

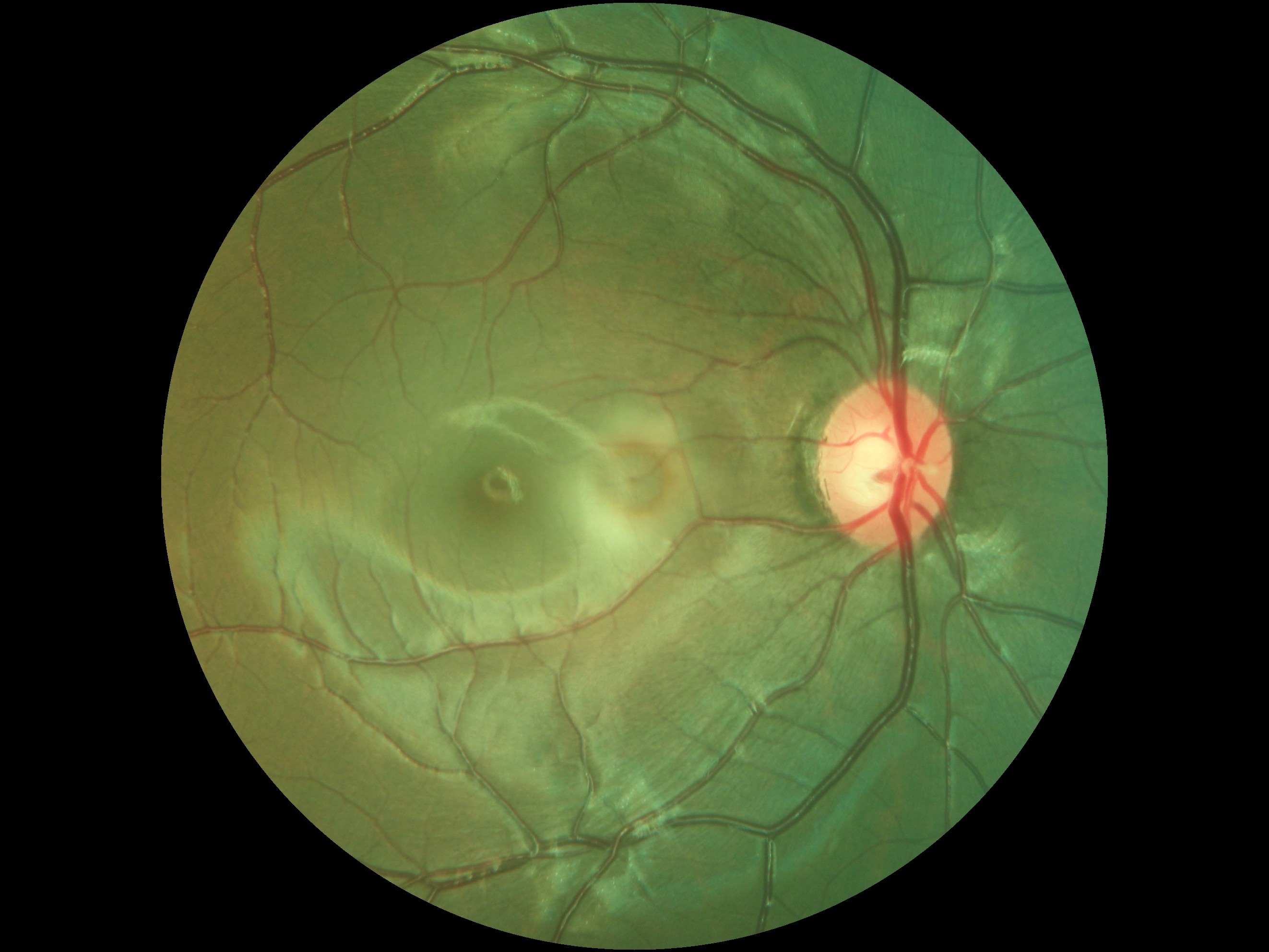

Supplement: S3 File — (ZIP) [file pone.0324352.s003.zip › Original fundus photographs (1)/Subject 32/OD_20230615871047_20230615114311_2.jpg]

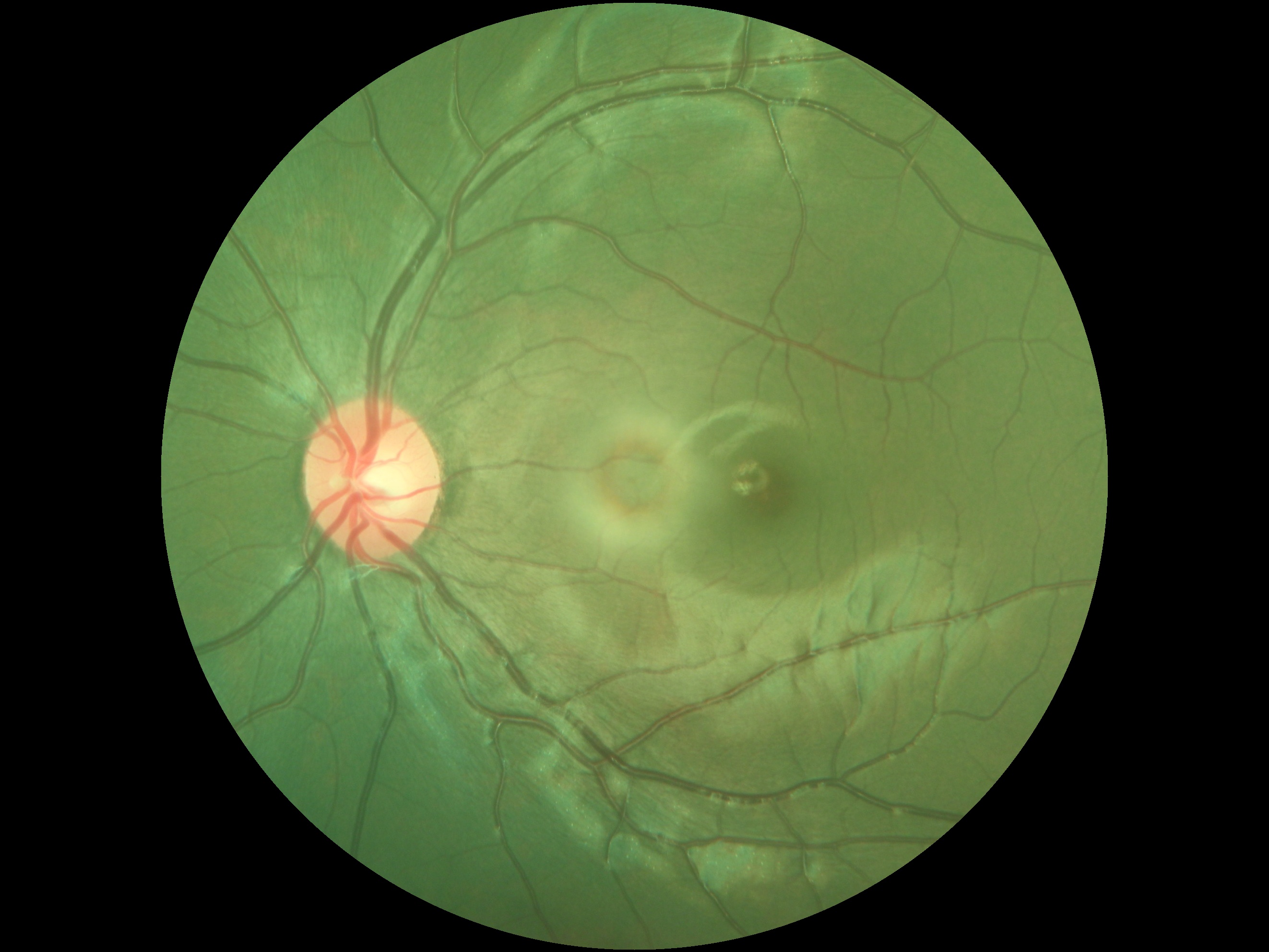

Supplement: S3 File — (ZIP) [file pone.0324352.s003.zip › Original fundus photographs (1)/Subject 32/OS_20230615871047_20230615114259_1.jpg]

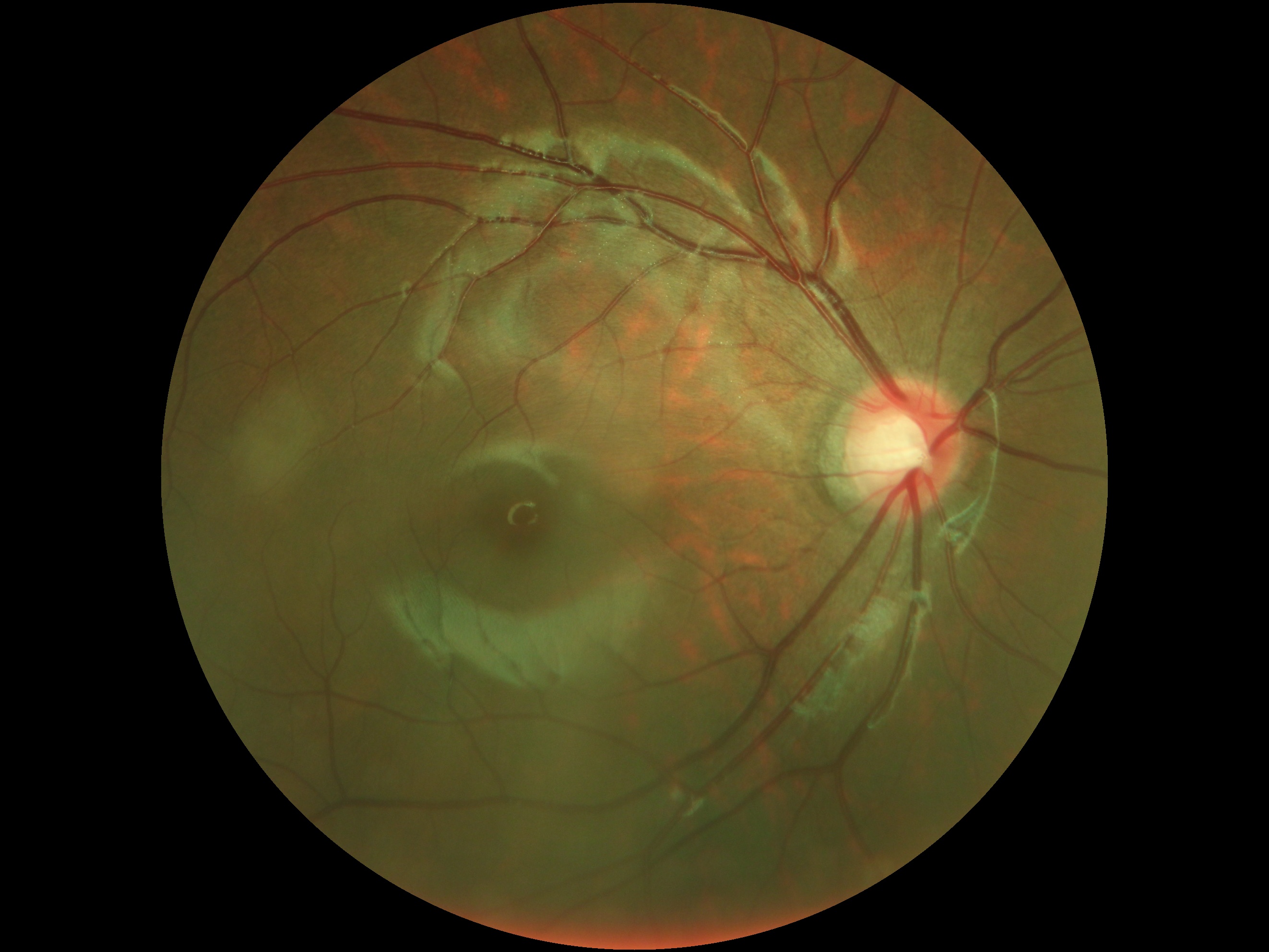

Supplement: S3 File — (ZIP) [file pone.0324352.s003.zip › Original fundus photographs (1)/Subject 33/OD_20230611025071_20230612105658_1.jpg]

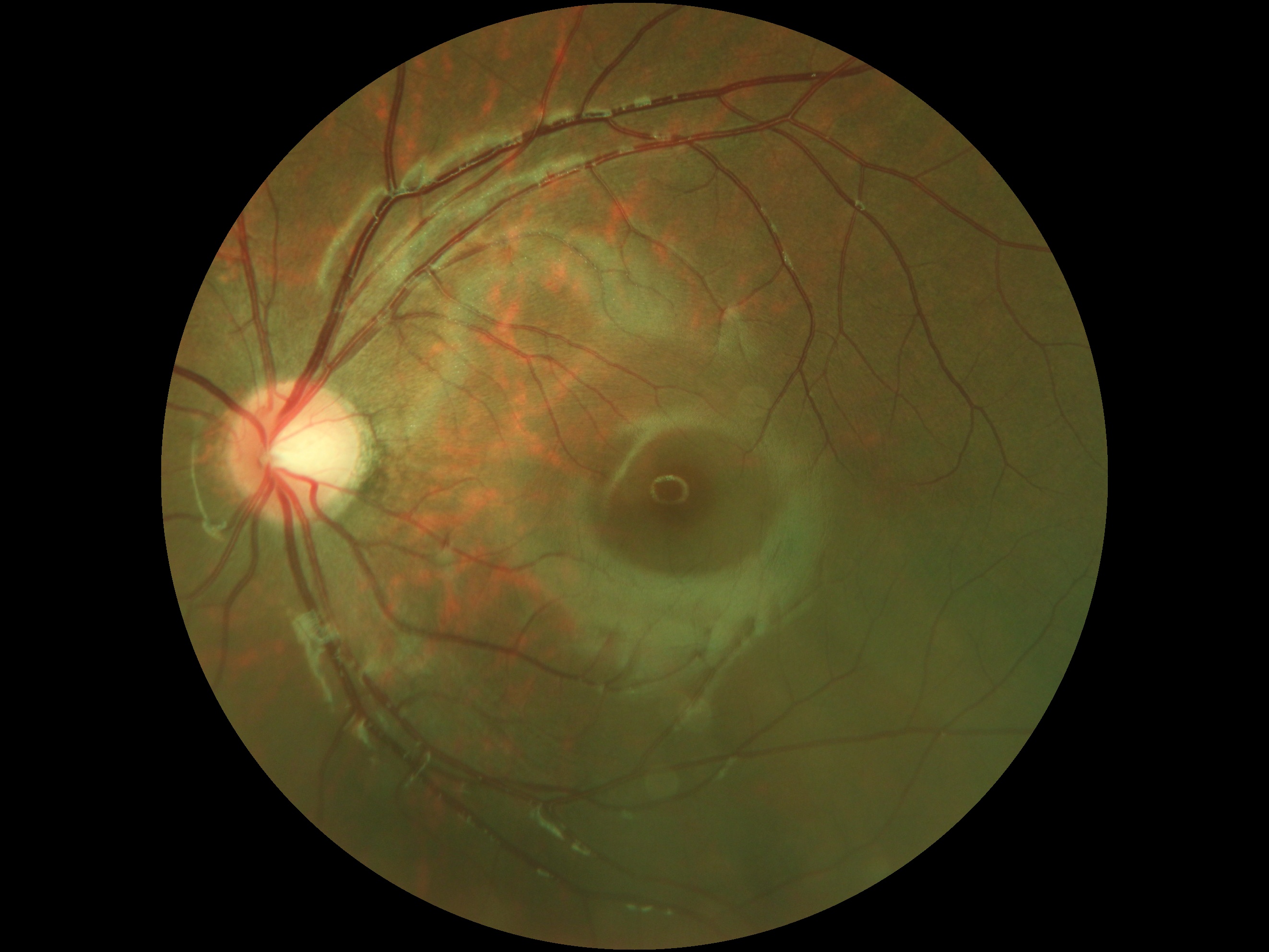

Supplement: S3 File — (ZIP) [file pone.0324352.s003.zip › Original fundus photographs (1)/Subject 33/OS_20230611025071_20230612105927_3.jpg]

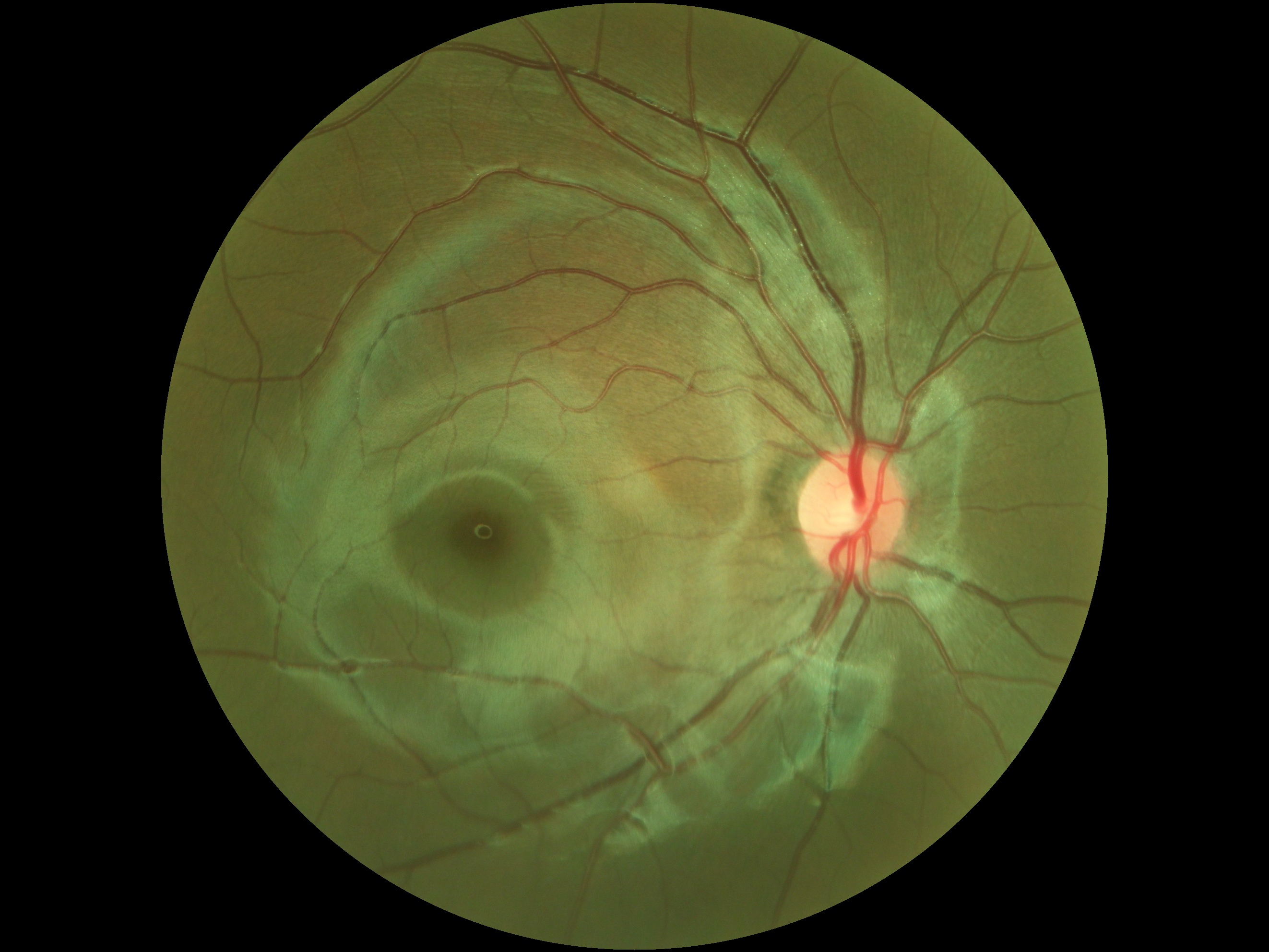

Supplement: S3 File — (ZIP) [file pone.0324352.s003.zip › Original fundus photographs (1)/Subject 34/OD_20230611138017_20230612112709_1.jpg]

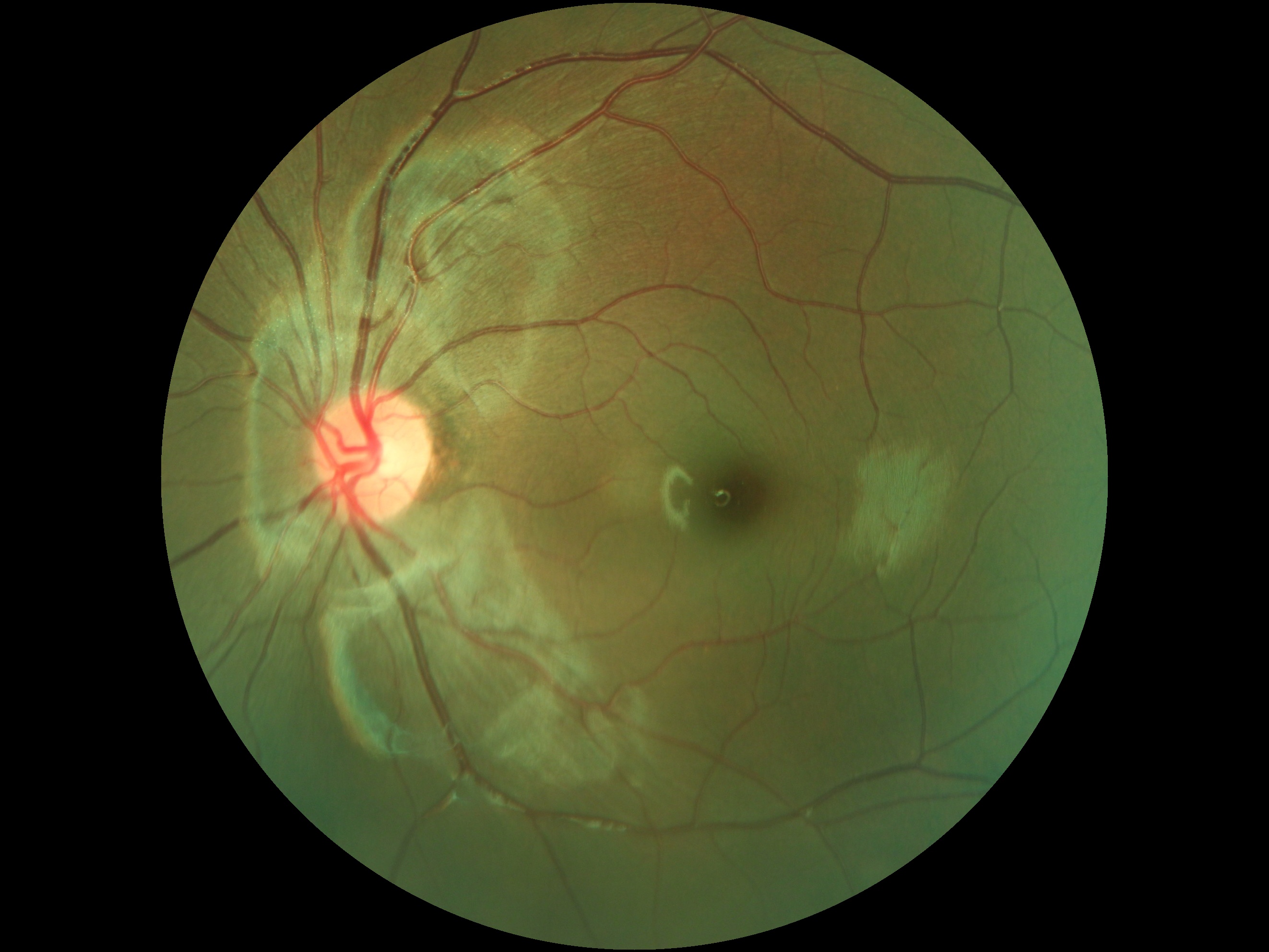

Supplement: S3 File — (ZIP) [file pone.0324352.s003.zip › Original fundus photographs (1)/Subject 34/OS_20230611138017_20230612112737_2.jpg]

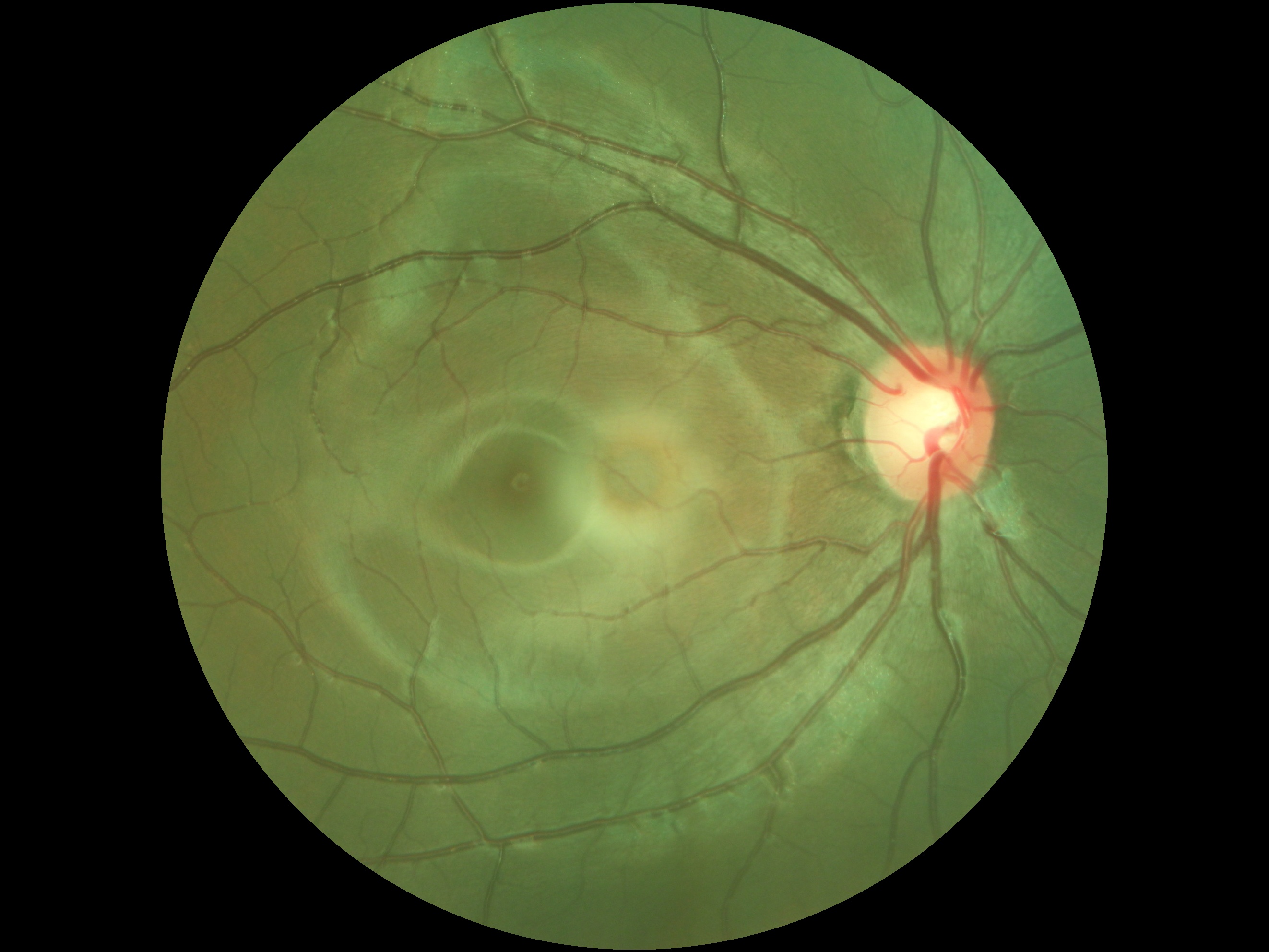

Supplement: S3 File — (ZIP) [file pone.0324352.s003.zip › Original fundus photographs (1)/Subject 35/OD_20230611269146_20230614102937_4.jpg]

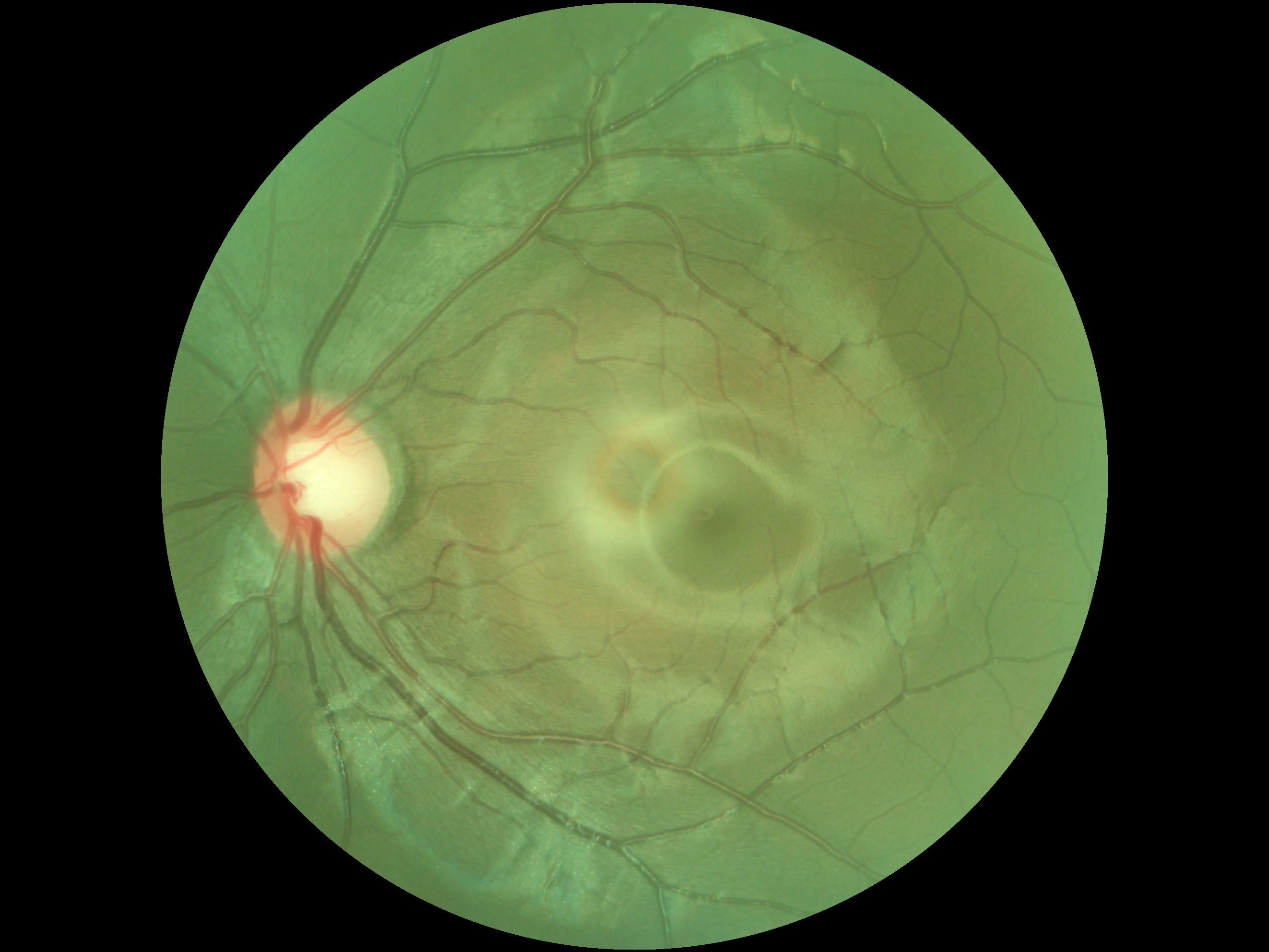

Supplement: S3 File — (ZIP) [file pone.0324352.s003.zip › Original fundus photographs (1)/Subject 35/OS_20230611269146_20230614102826_2.jpg]

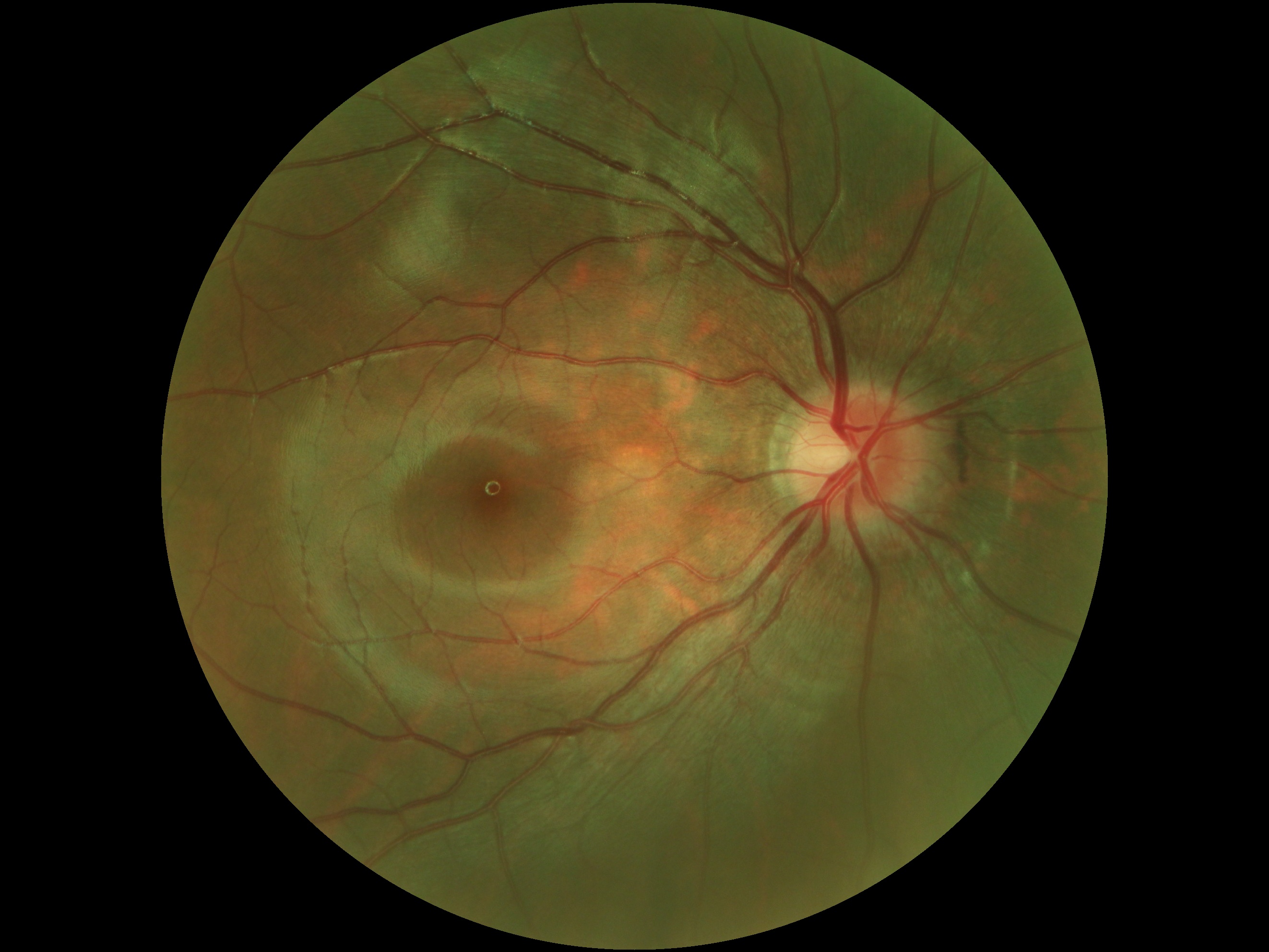

Supplement: S3 File — (ZIP) [file pone.0324352.s003.zip › Original fundus photographs (1)/Subject 36/OD_20230611923083_20230612153152_1.jpg]

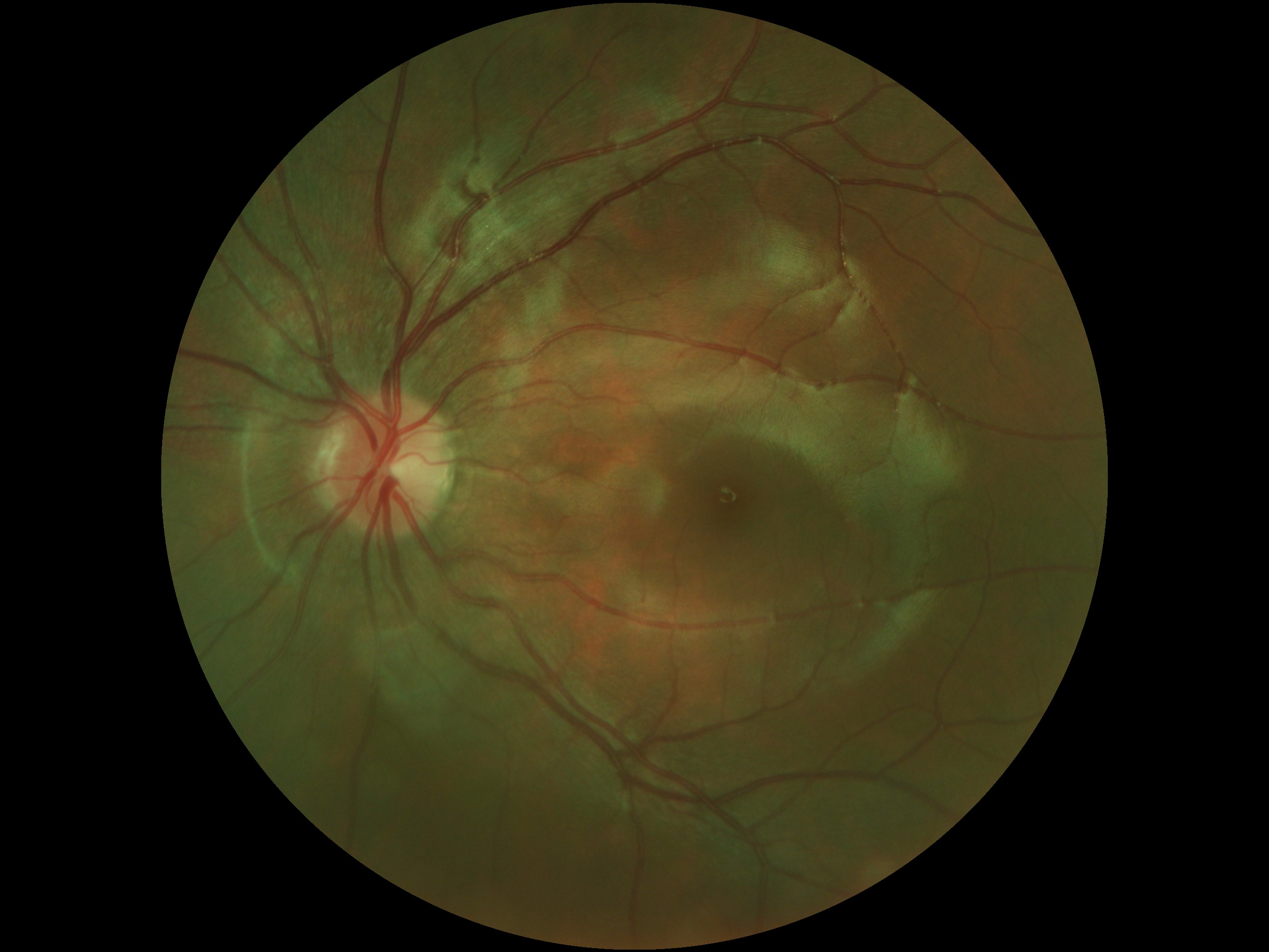

Supplement: S3 File — (ZIP) [file pone.0324352.s003.zip › Original fundus photographs (1)/Subject 36/OS_20230611923083_20230612153232_2.jpg]

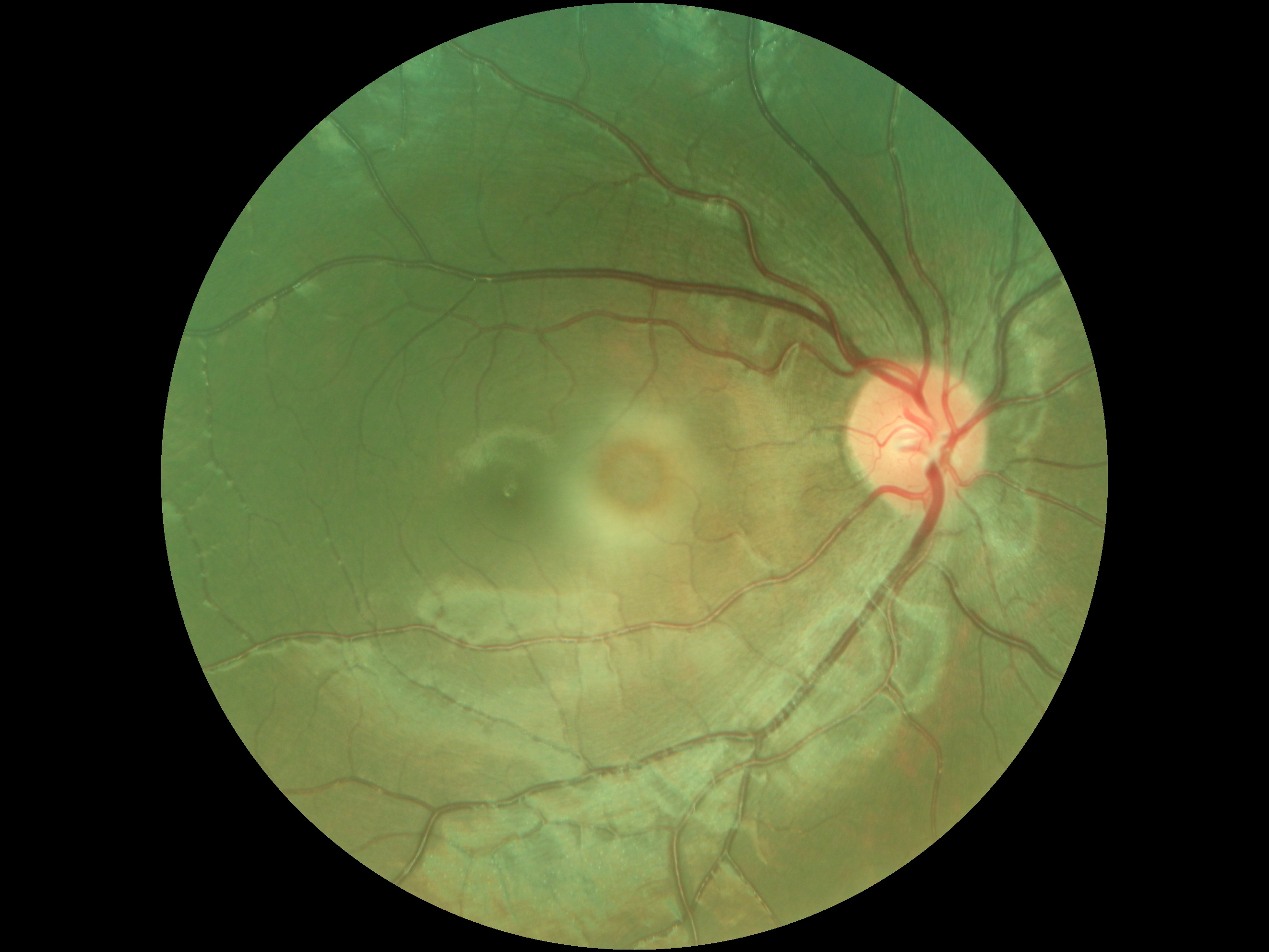

Supplement: S3 File — (ZIP) [file pone.0324352.s003.zip › Original fundus photographs (1)/Subject 37/OD_20230615859064_20230615153813_1.jpg]

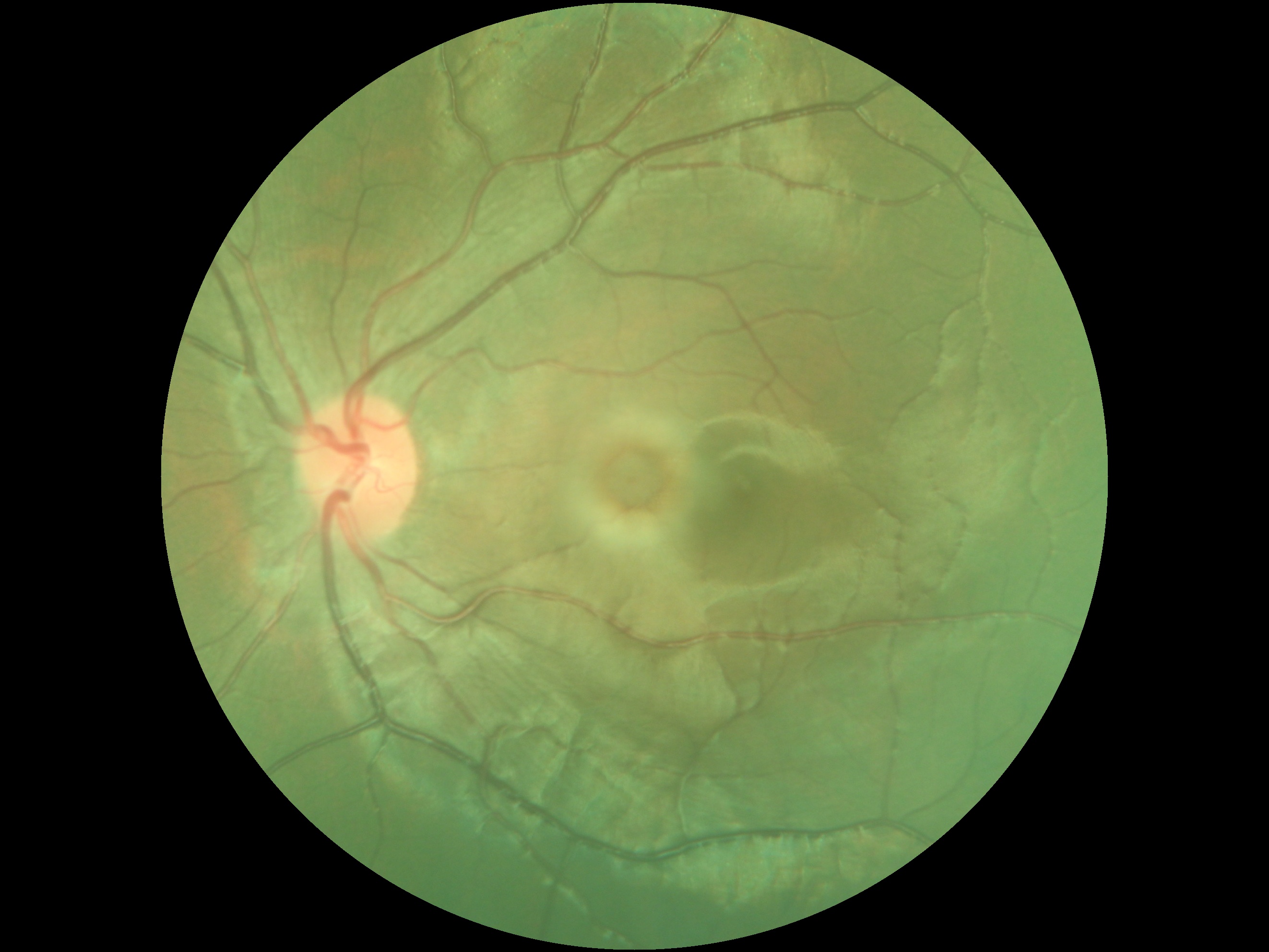

Supplement: S3 File — (ZIP) [file pone.0324352.s003.zip › Original fundus photographs (1)/Subject 37/OS_20230615859064_20230615153850_2.jpg]

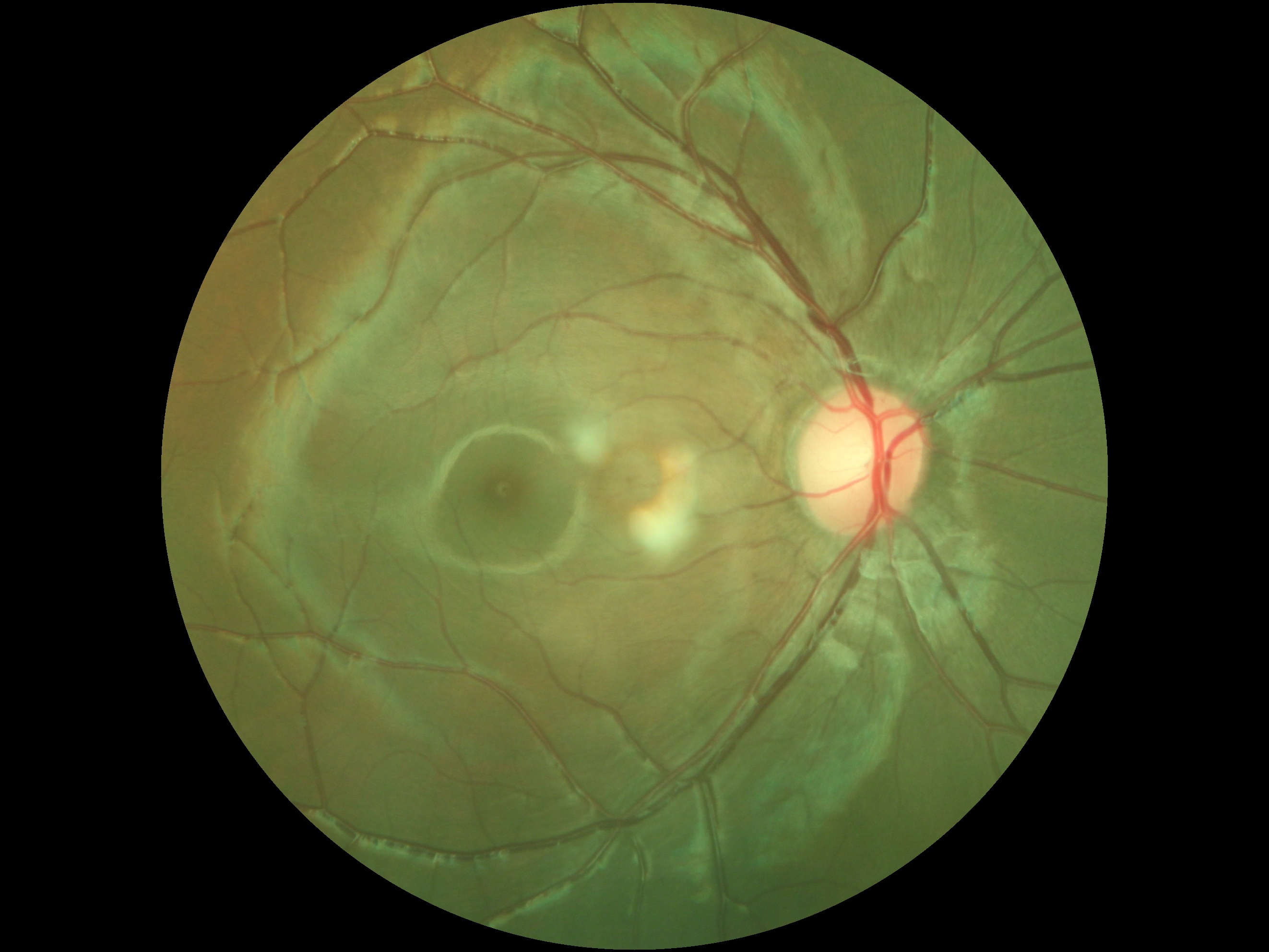

Supplement: S3 File — (ZIP) [file pone.0324352.s003.zip › Original fundus photographs (1)/Subject 38/OD_20230615290076_20230615161633_3.jpg]

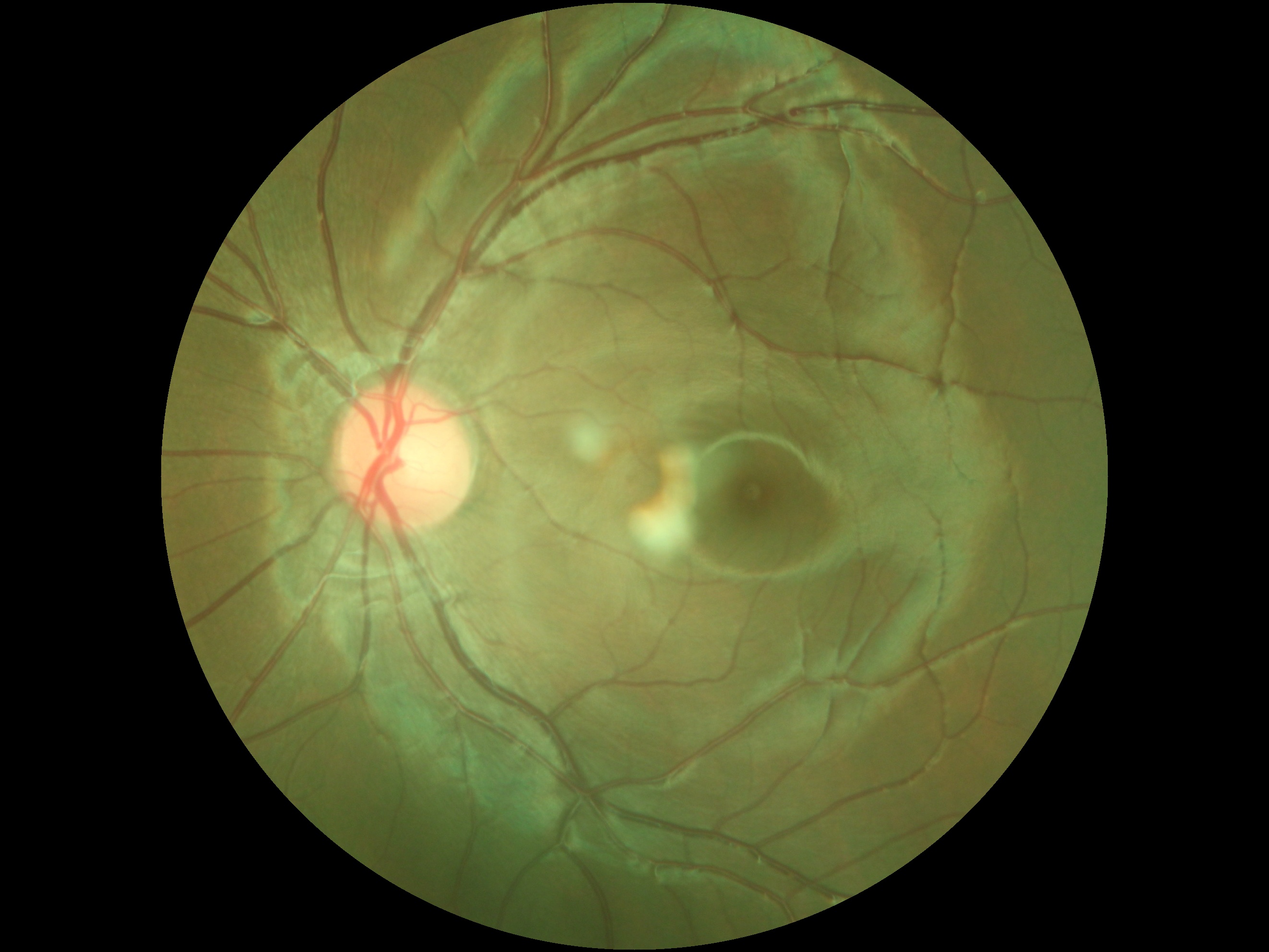

Supplement: S3 File — (ZIP) [file pone.0324352.s003.zip › Original fundus photographs (1)/Subject 38/OS_20230615290076_20230615161616_2.jpg]

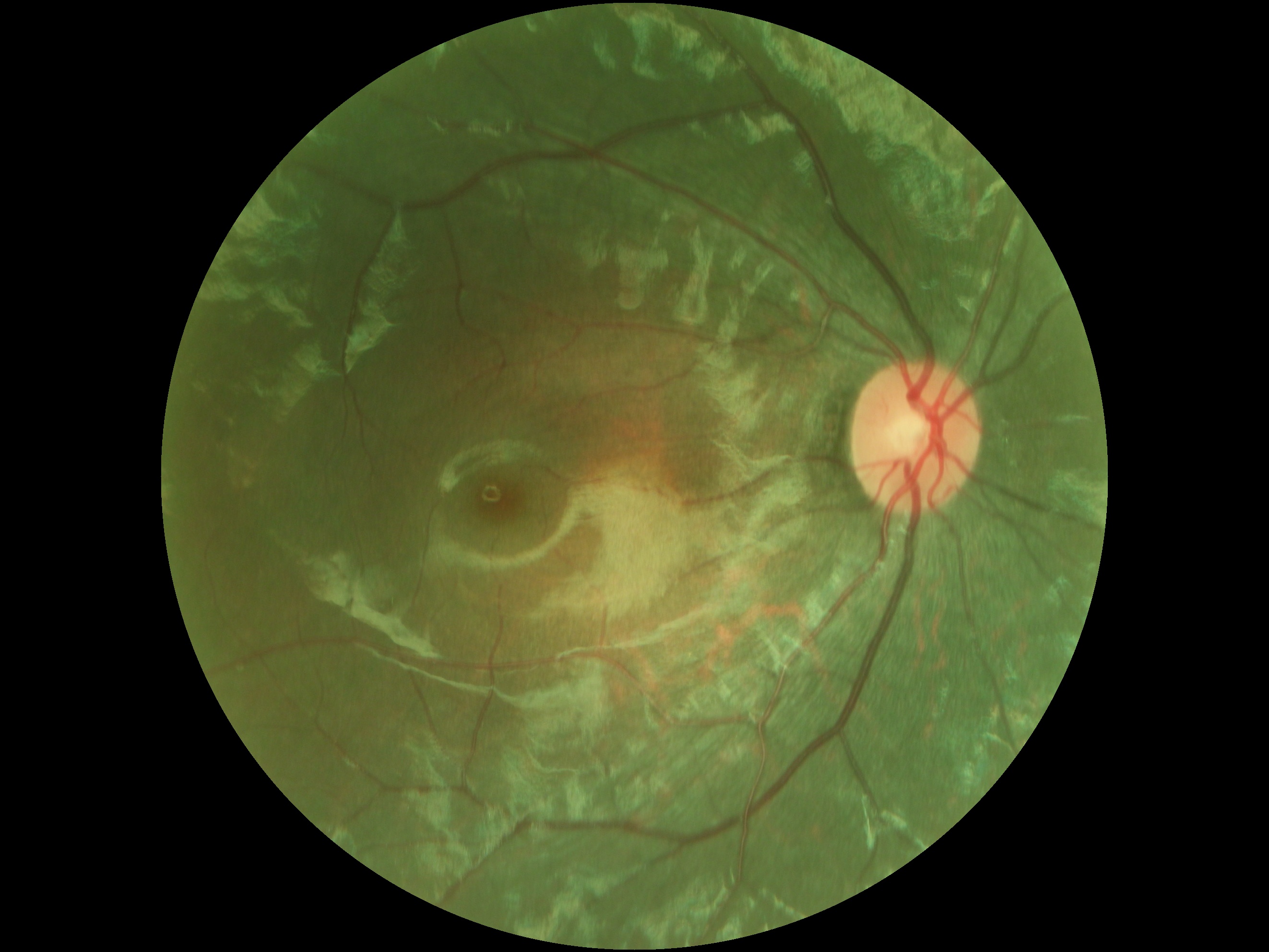

Supplement: S3 File — (ZIP) [file pone.0324352.s003.zip › Original fundus photographs (1)/Subject 39/OD_20230611949063_20230612162640_1.jpg]

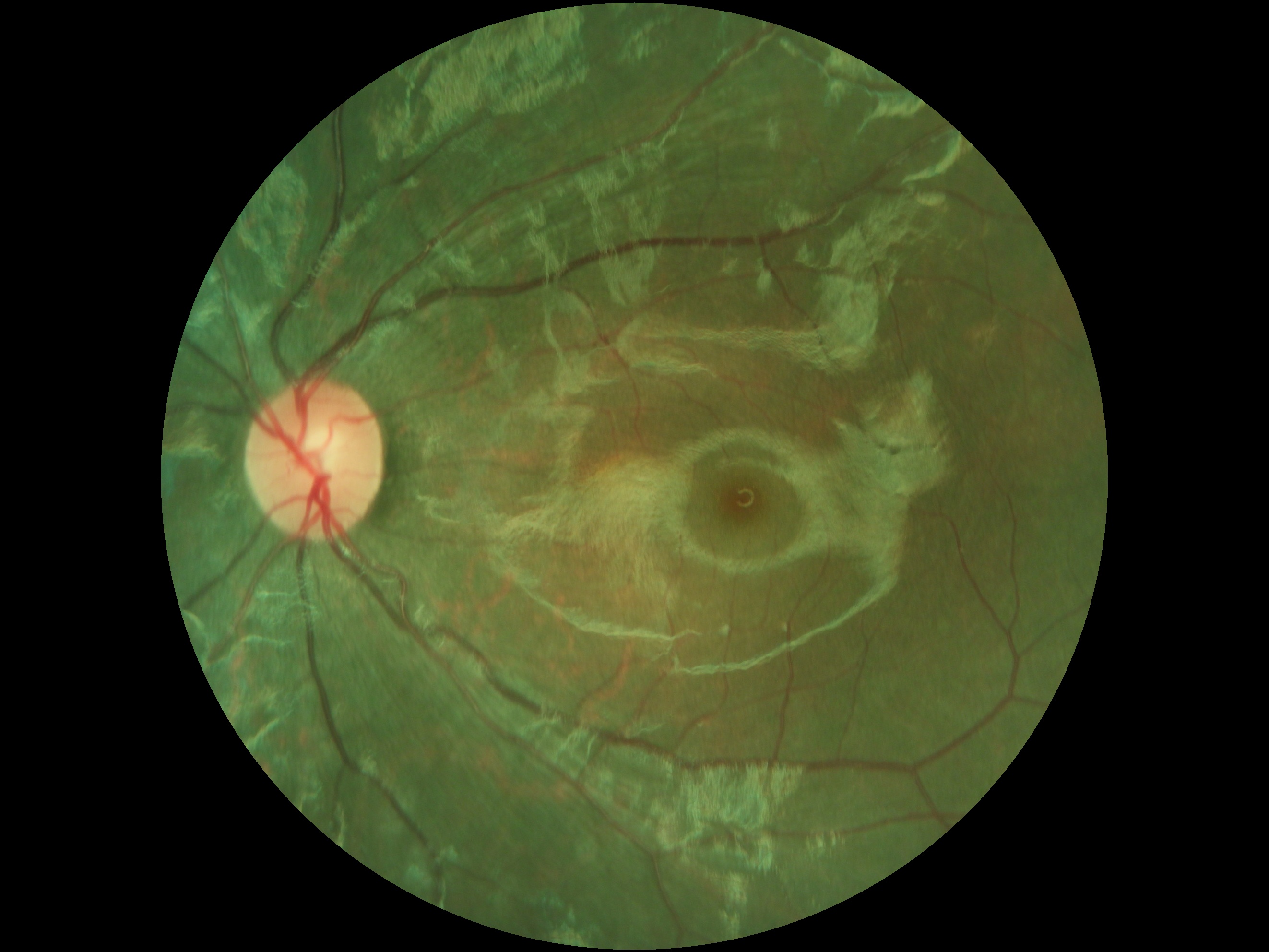

Supplement: S3 File — (ZIP) [file pone.0324352.s003.zip › Original fundus photographs (1)/Subject 39/OS_20230611949063_20230612162704_2.jpg]

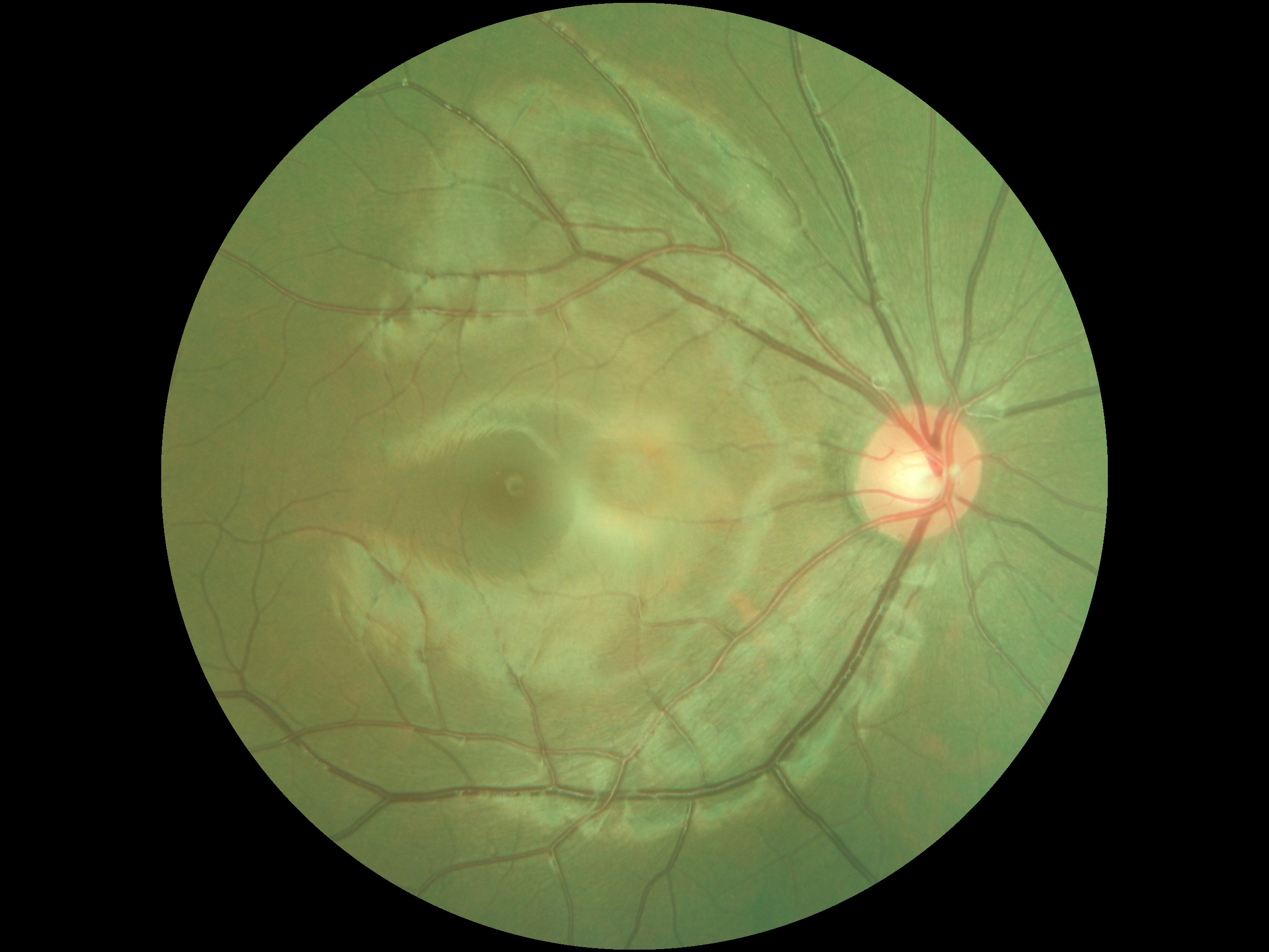

Supplement: S3 File — (ZIP) [file pone.0324352.s003.zip › Original fundus photographs (1)/Subject 4/OD_20230613268015_20230614161101_2.jpg]

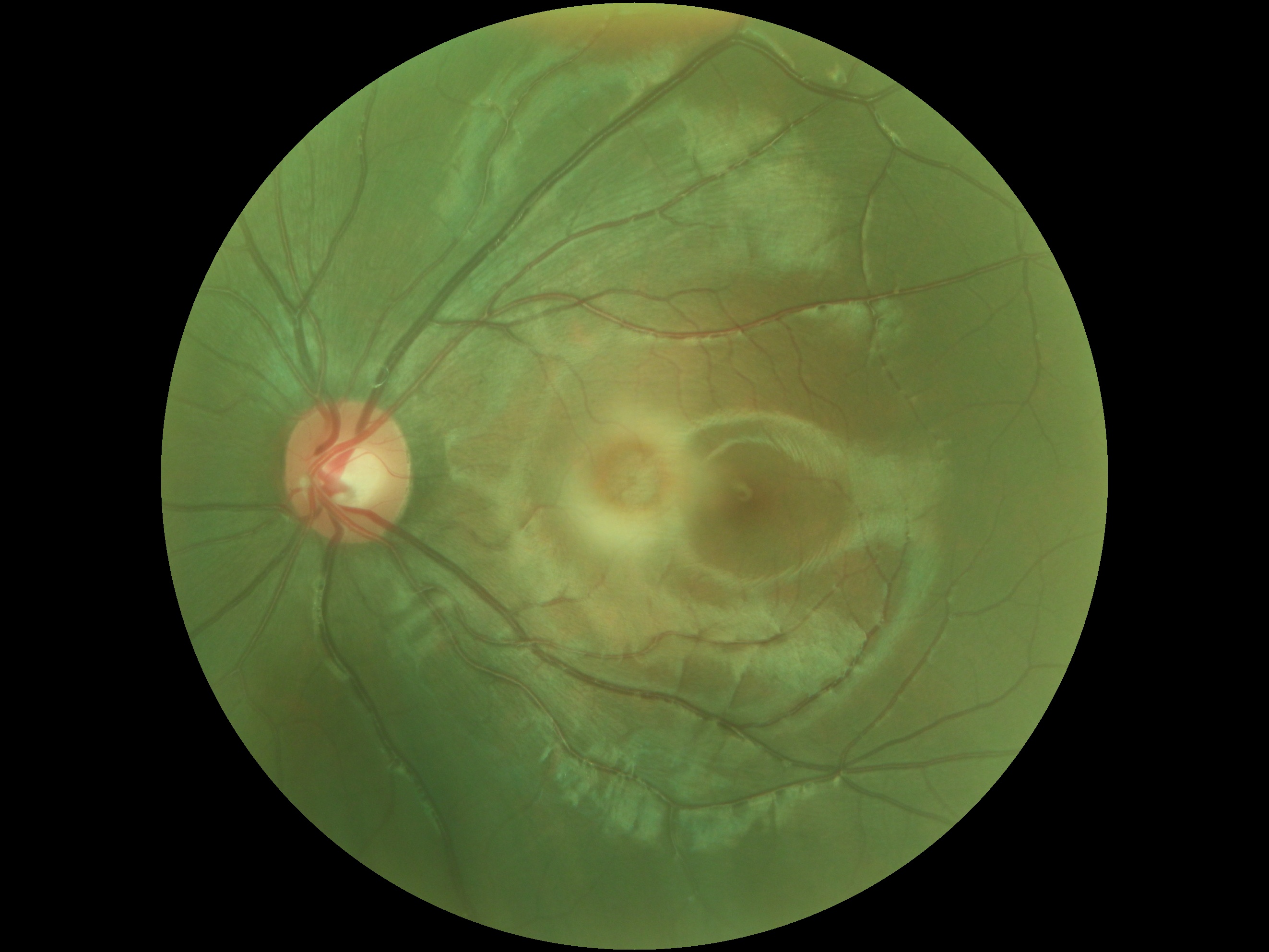

Supplement: S3 File — (ZIP) [file pone.0324352.s003.zip › Original fundus photographs (1)/Subject 4/OS_20230613268015_20230614161033_1.jpg]

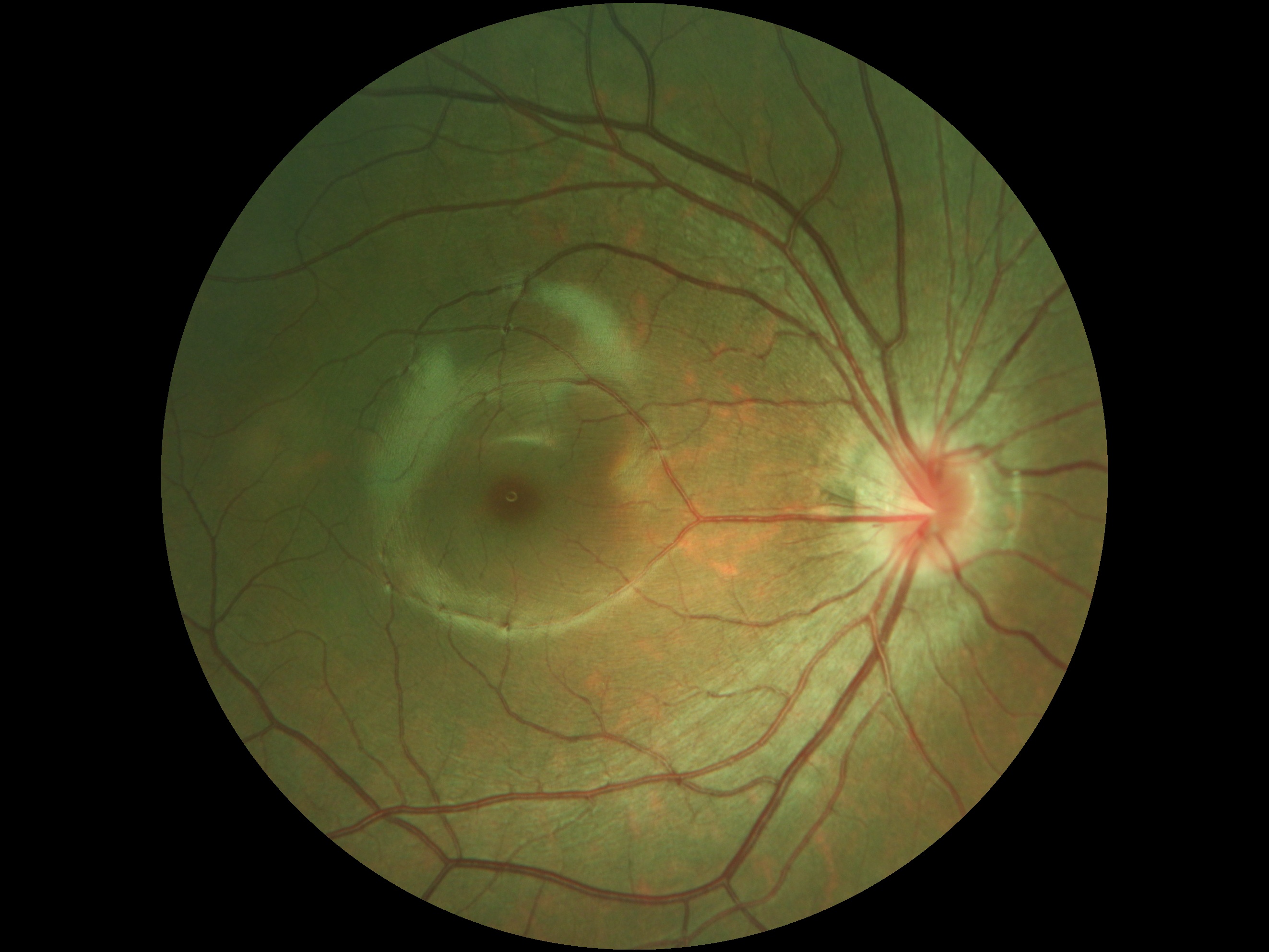

Supplement: S3 File — (ZIP) [file pone.0324352.s003.zip › Original fundus photographs (1)/Subject 40/OD_20230611842014_20230612112522_1.jpg]

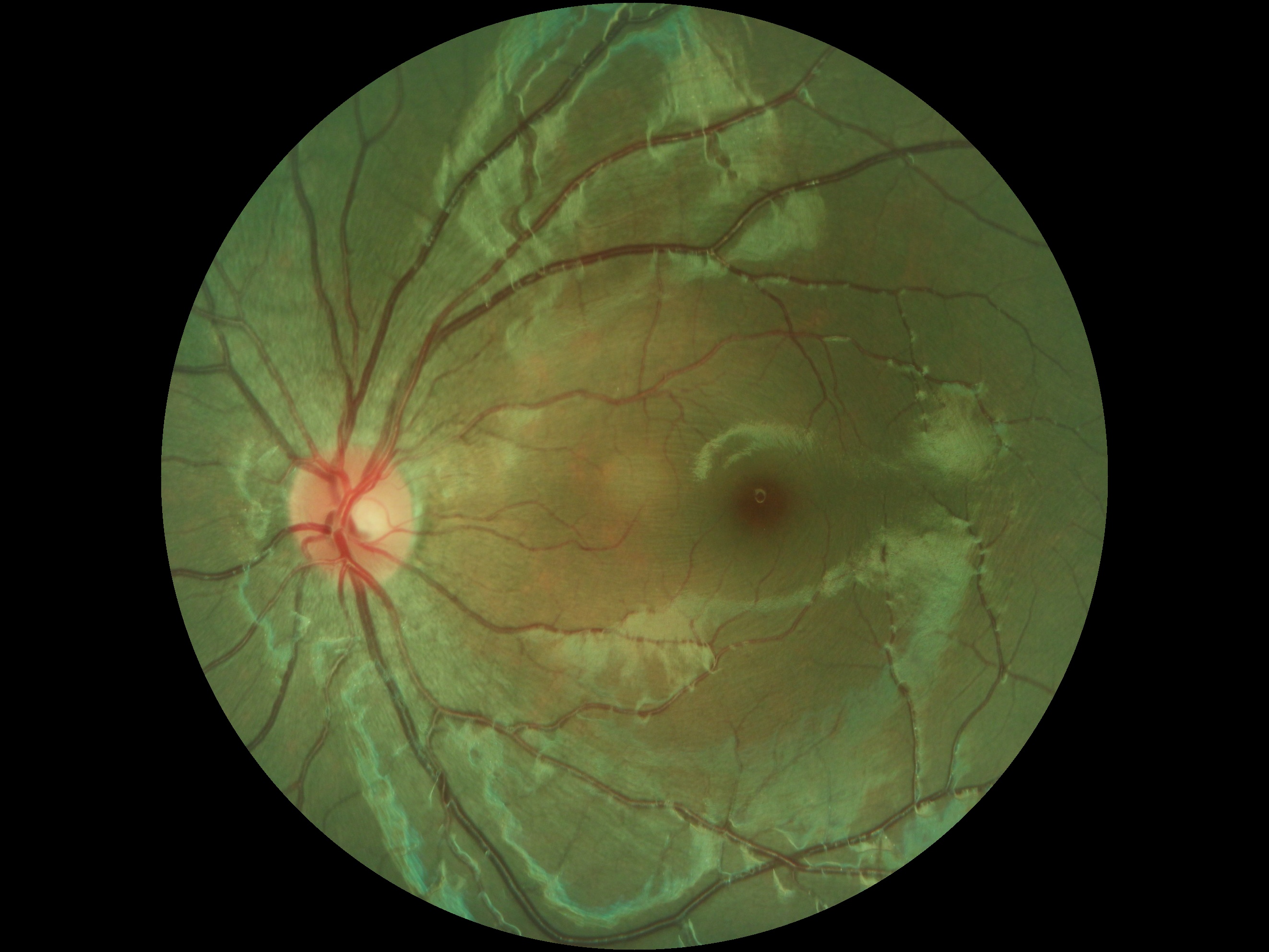

Supplement: S3 File — (ZIP) [file pone.0324352.s003.zip › Original fundus photographs (1)/Subject 40/OS_20230611842014_20230612112549_2.jpg]

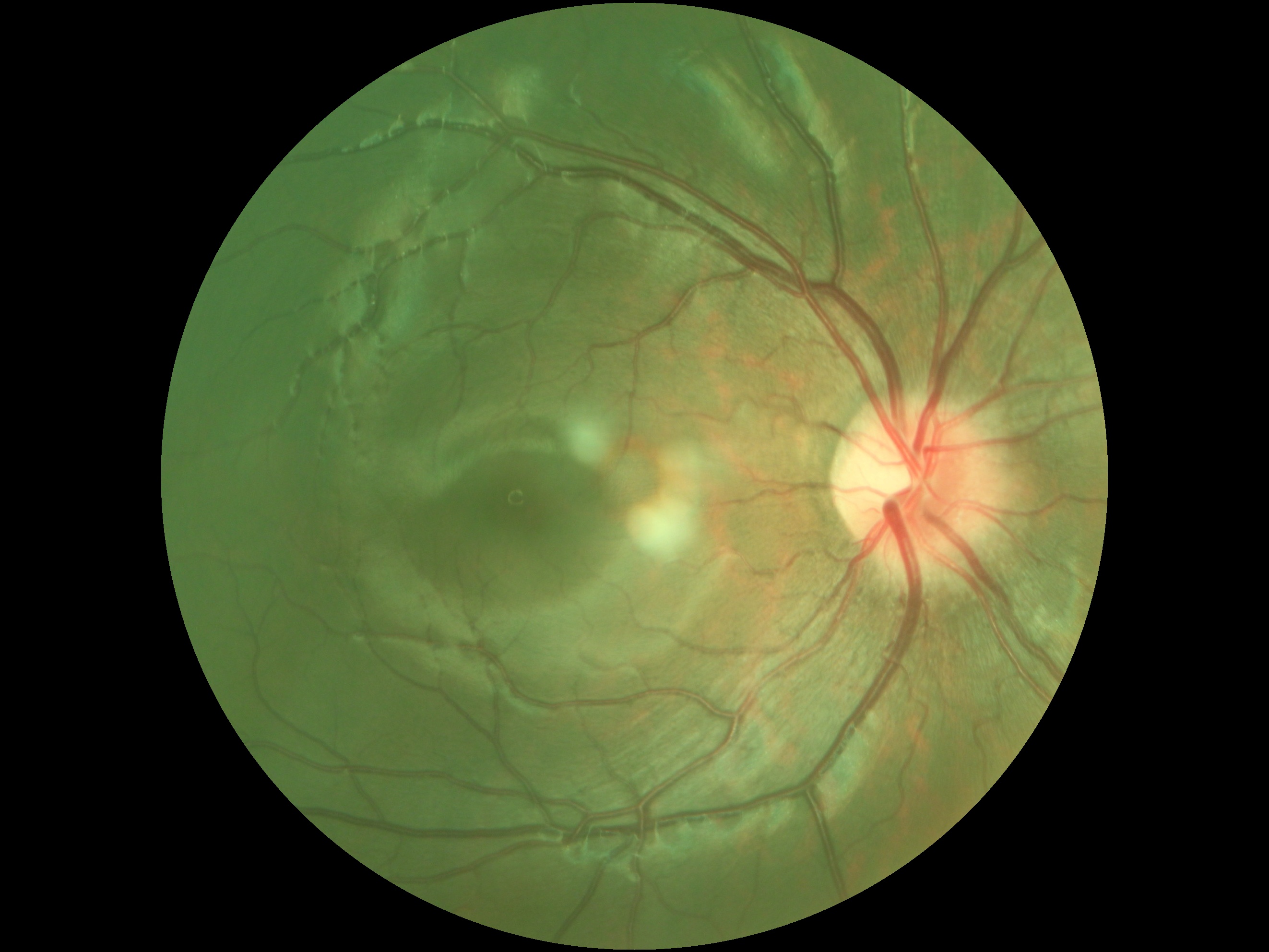

Supplement: S3 File — (ZIP) [file pone.0324352.s003.zip › Original fundus photographs (1)/Subject 41/OD_20230615593077_20230615161243_1.jpg]

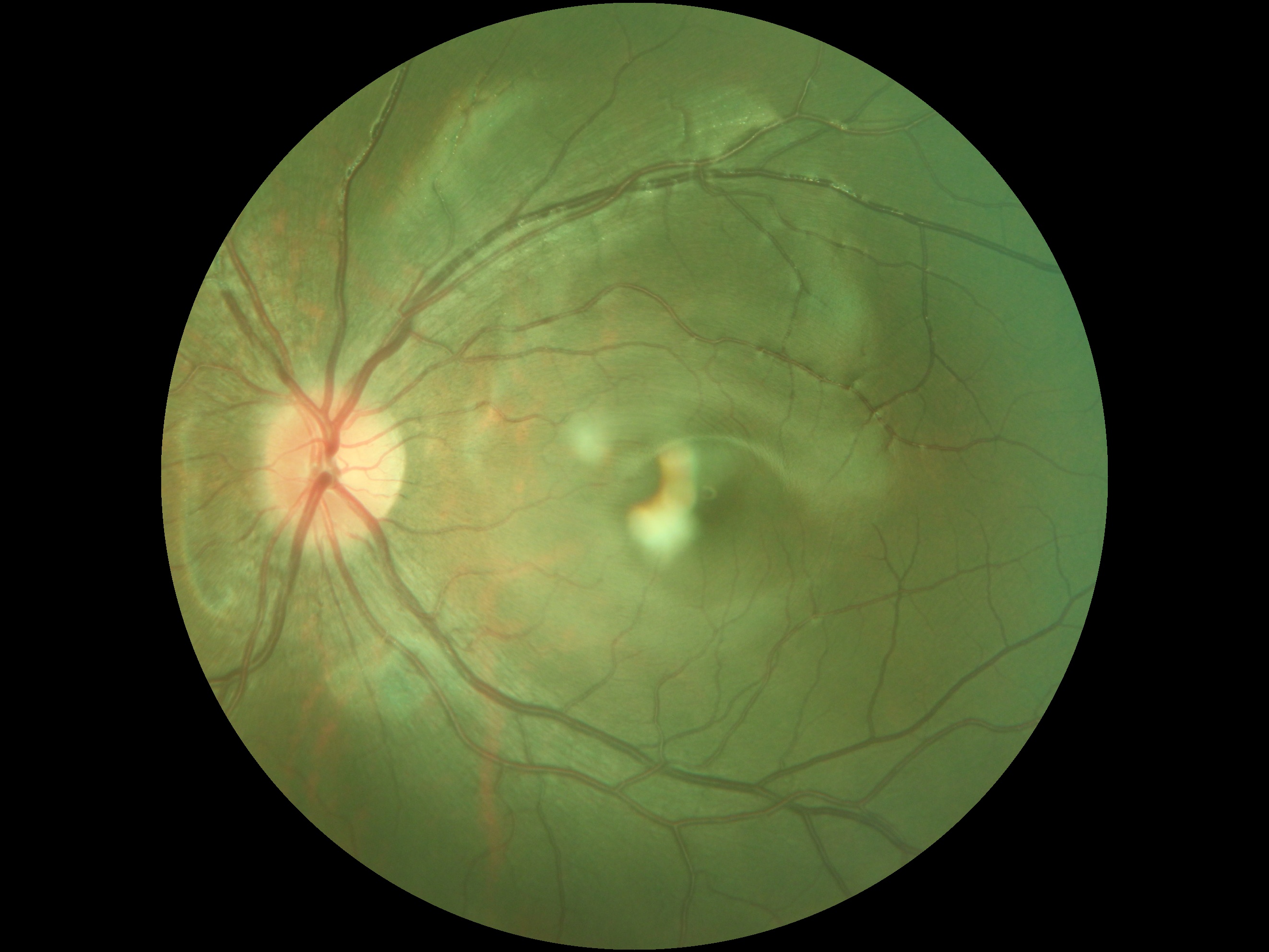

Supplement: S3 File — (ZIP) [file pone.0324352.s003.zip › Original fundus photographs (1)/Subject 41/OS_20230615593077_20230615161515_5.jpg]

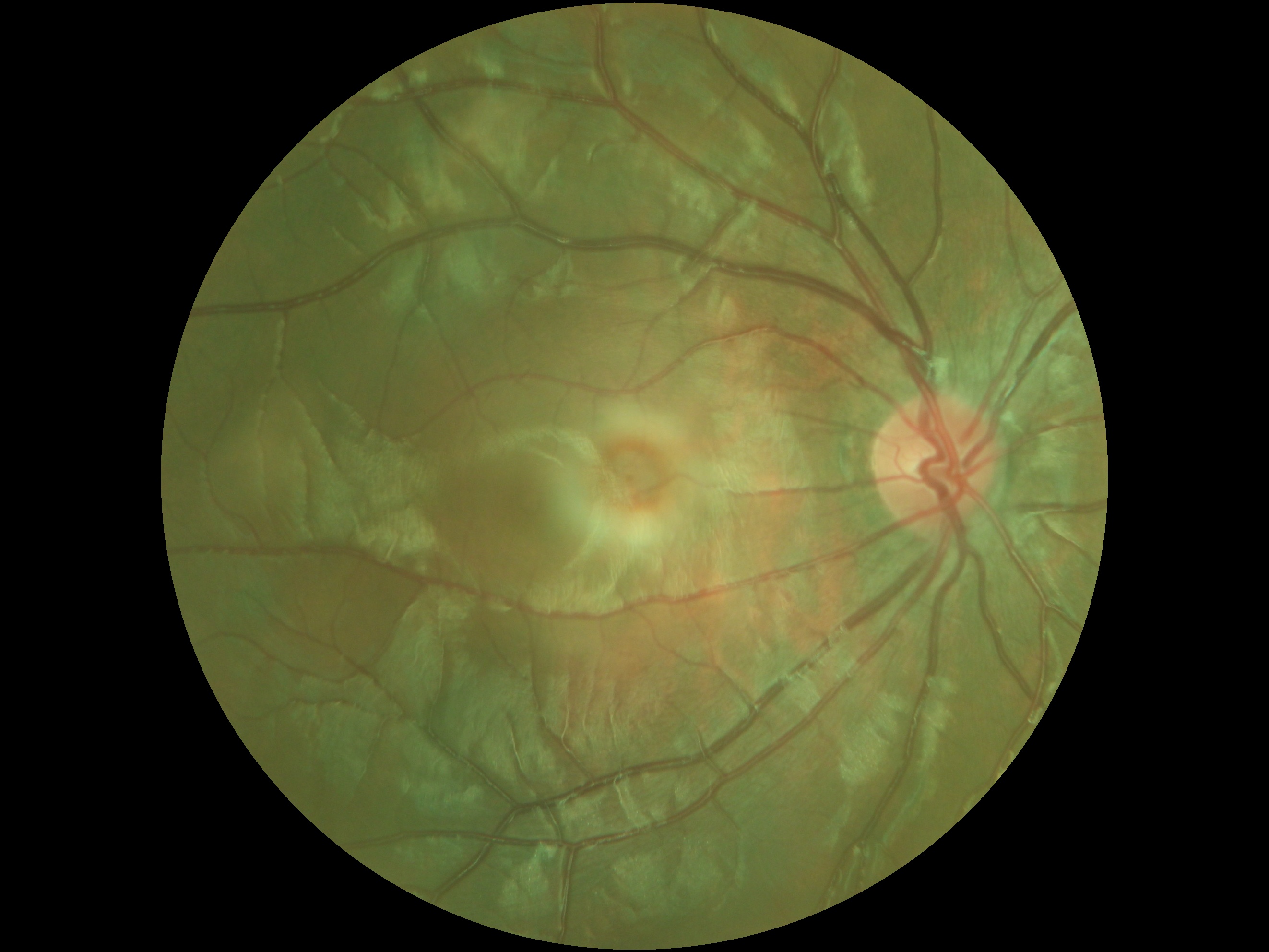

Supplement: S3 File — (ZIP) [file pone.0324352.s003.zip › Original fundus photographs (1)/Subject 42/OD_20230611728185_20230614101227_2.jpg]

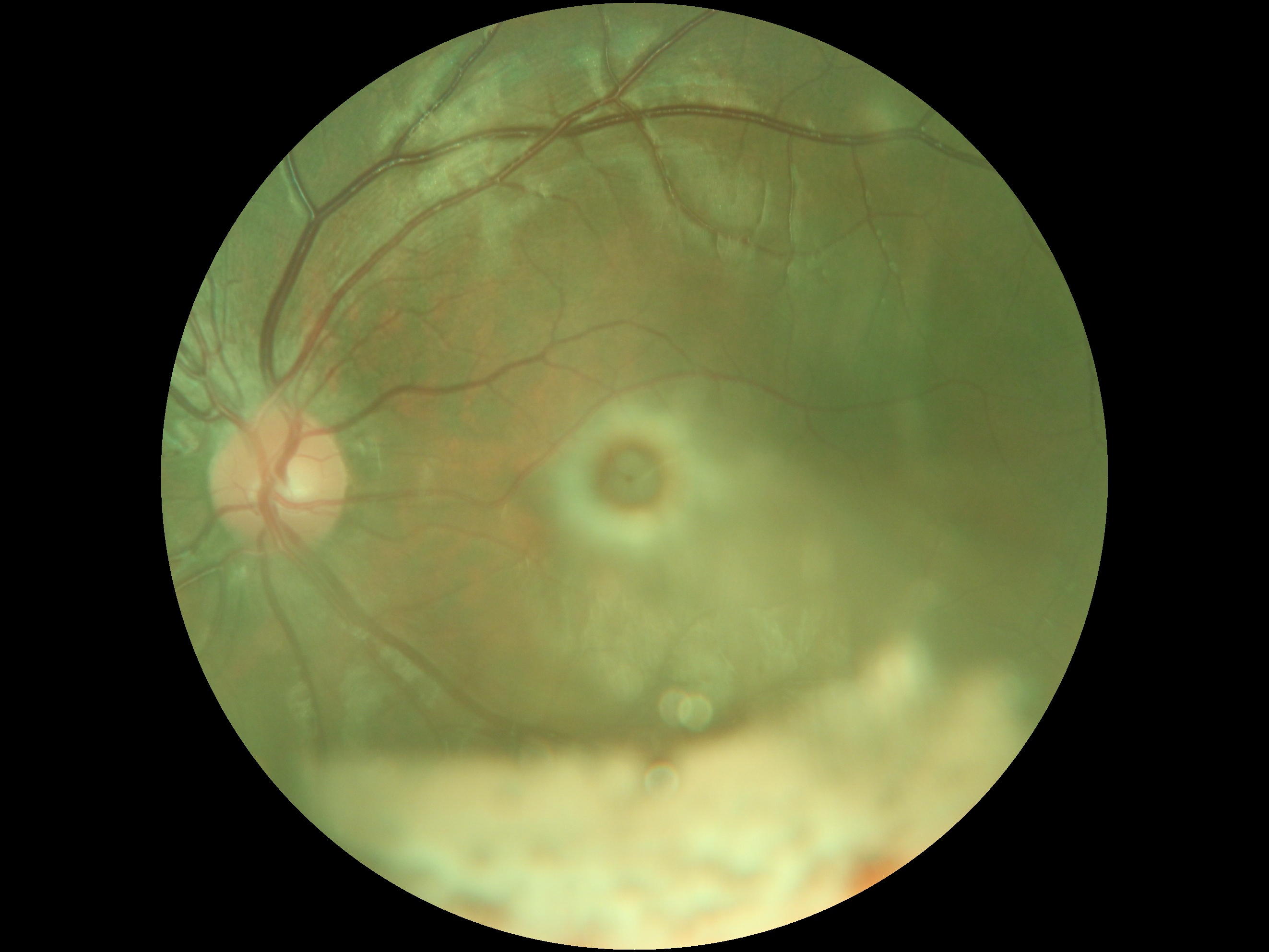

Supplement: S3 File — (ZIP) [file pone.0324352.s003.zip › Original fundus photographs (1)/Subject 42/OS_20230611728185_20230614101451_4.jpg]

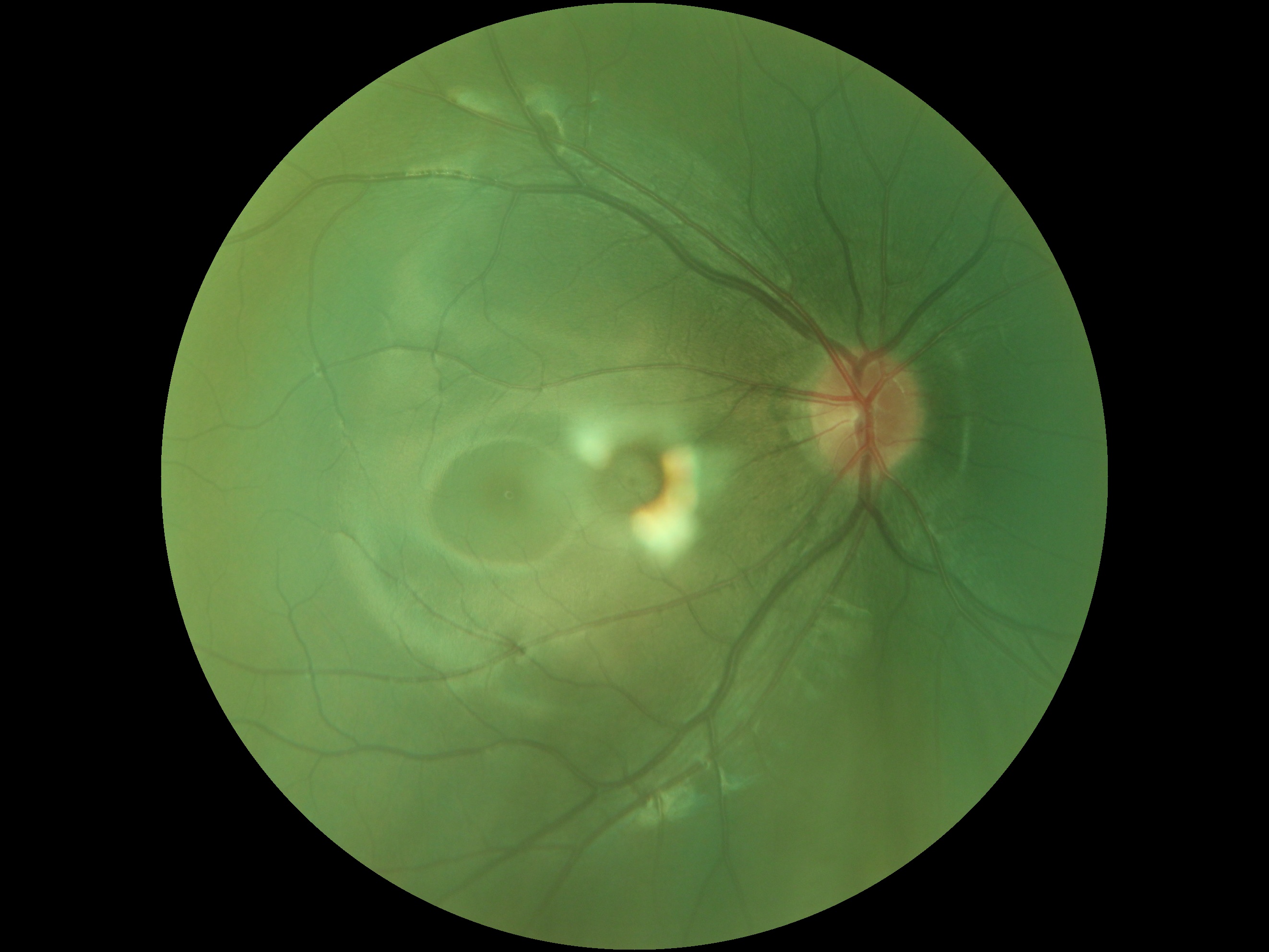

Supplement: S3 File — (ZIP) [file pone.0324352.s003.zip › Original fundus photographs (1)/Subject 43/OD_20230615125096_20230615170114_2.jpg]

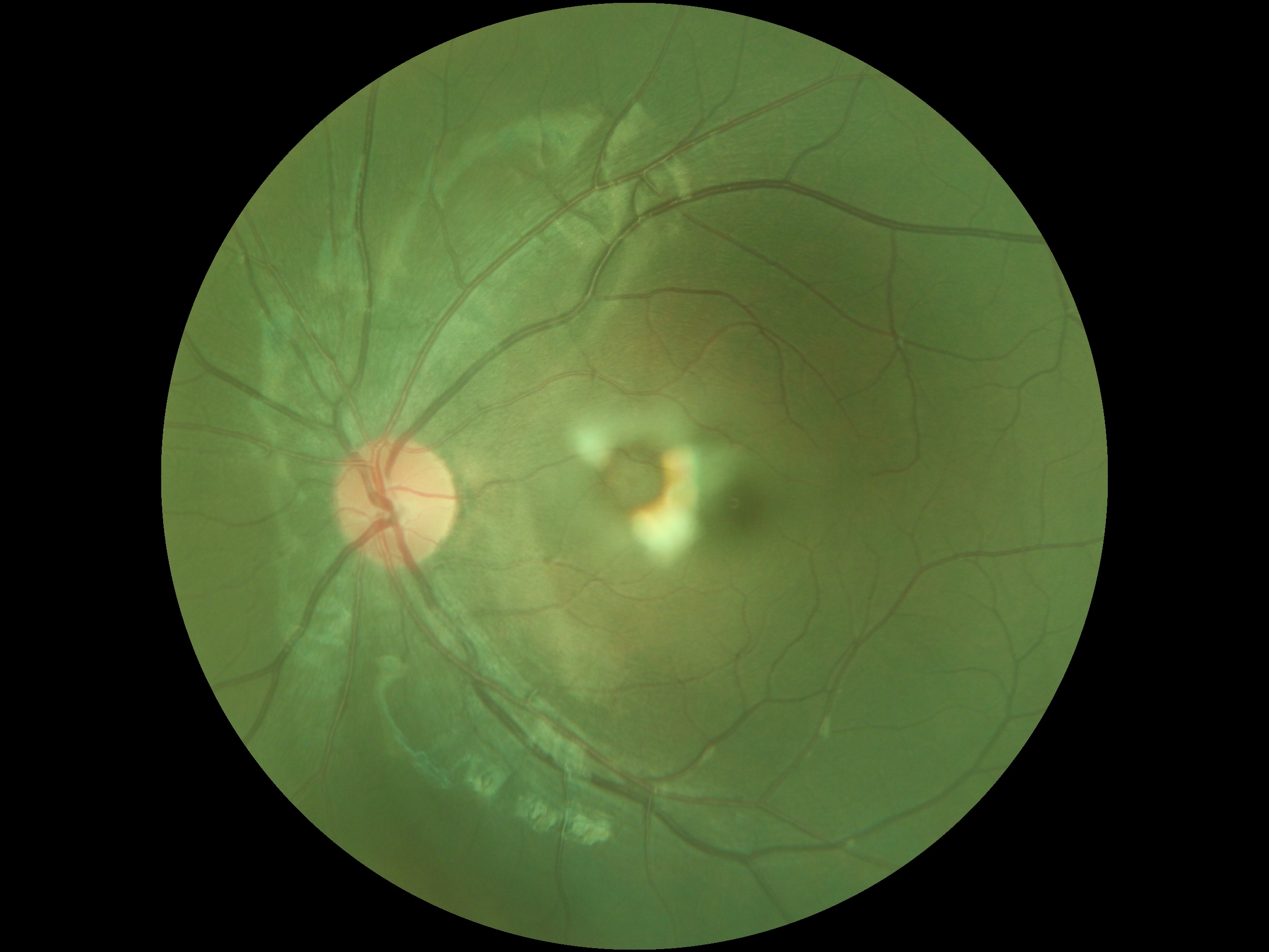

Supplement: S3 File — (ZIP) [file pone.0324352.s003.zip › Original fundus photographs (1)/Subject 43/OS_20230615125096_20230615170054_1.jpg]

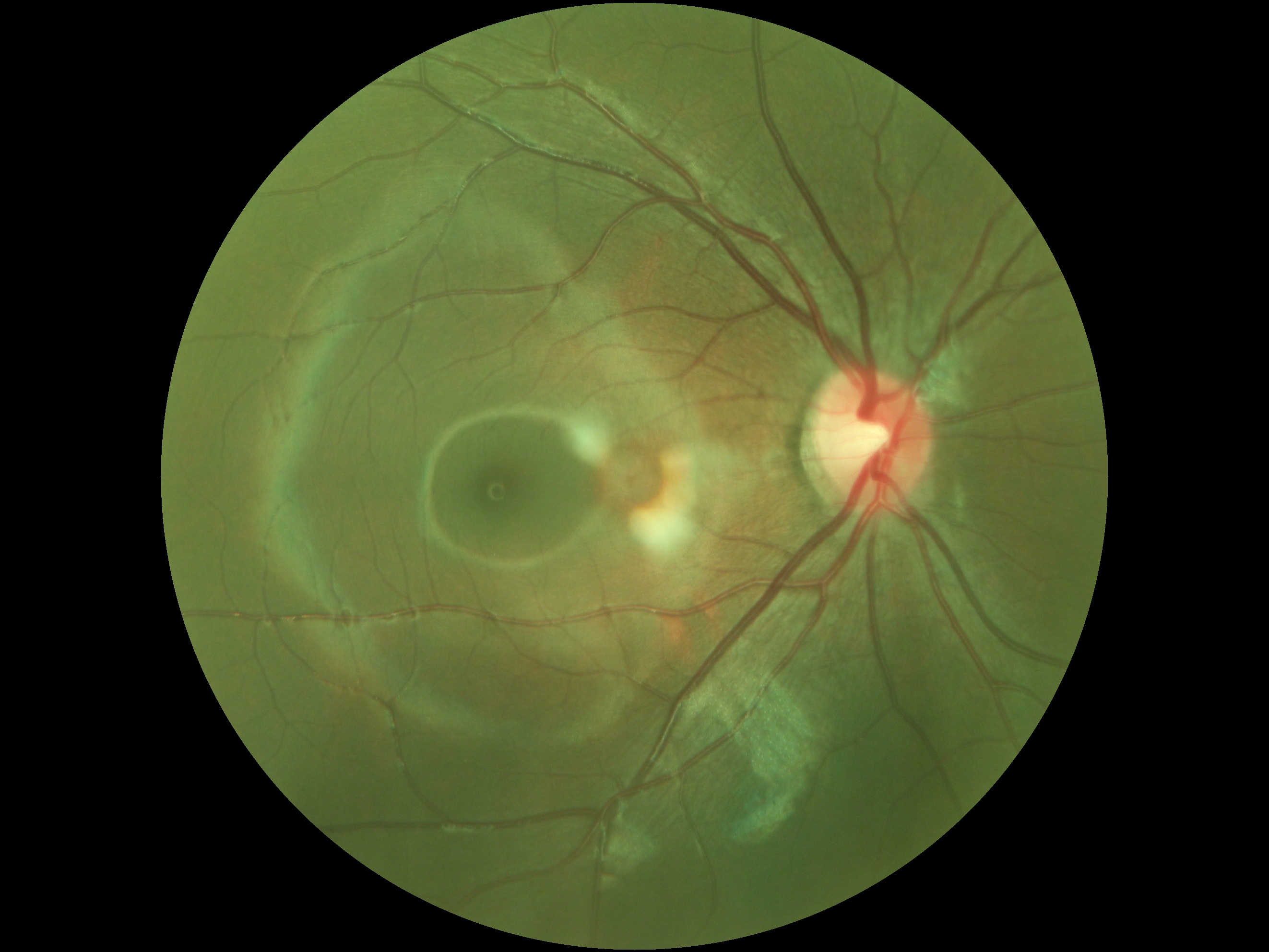

Supplement: S3 File — (ZIP) [file pone.0324352.s003.zip › Original fundus photographs (1)/Subject 44/OD_20230615526078_20230615161104_3.jpg]

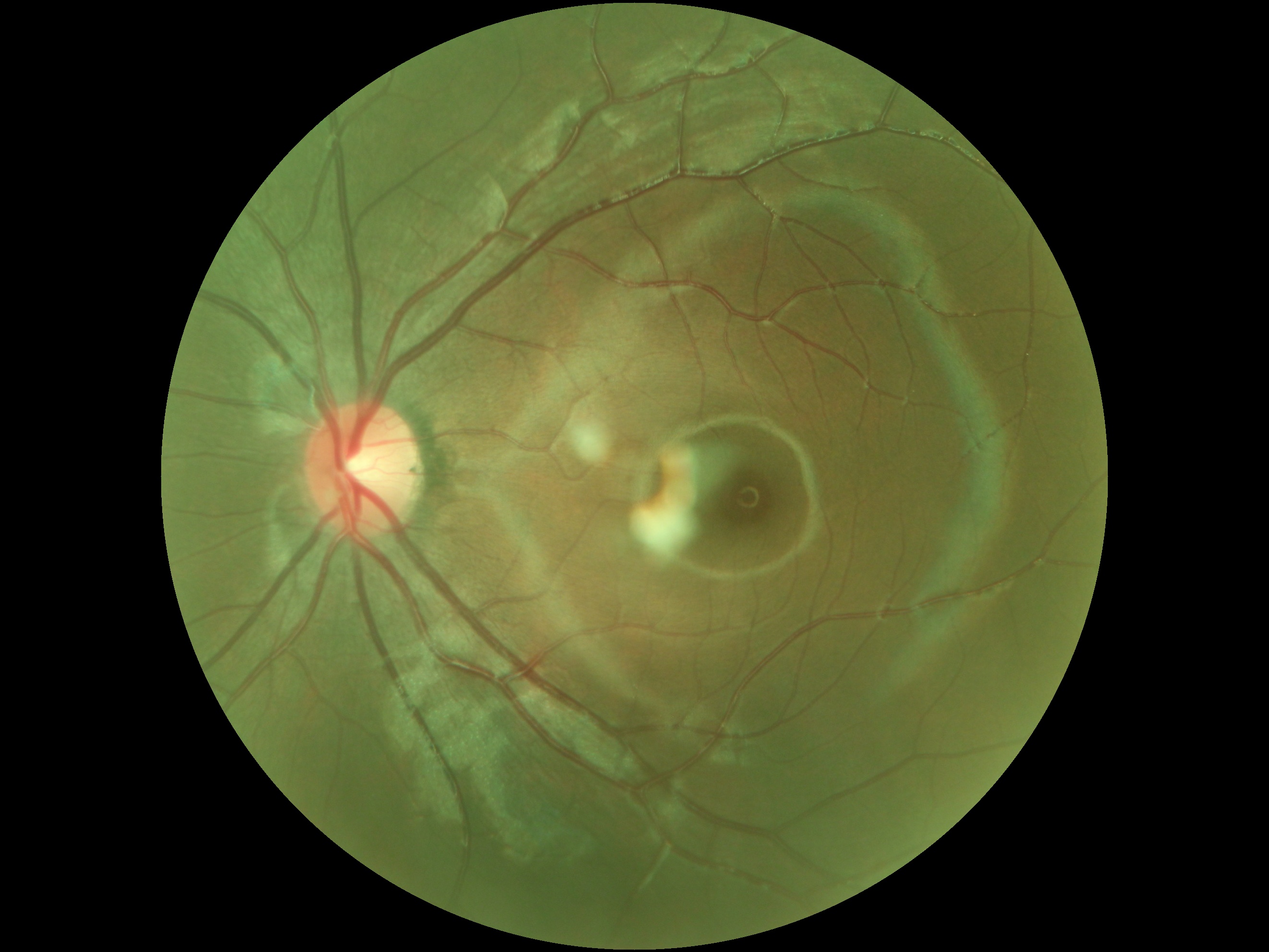

Supplement: S3 File — (ZIP) [file pone.0324352.s003.zip › Original fundus photographs (1)/Subject 44/OS_20230615526078_20230615161029_1.jpg]

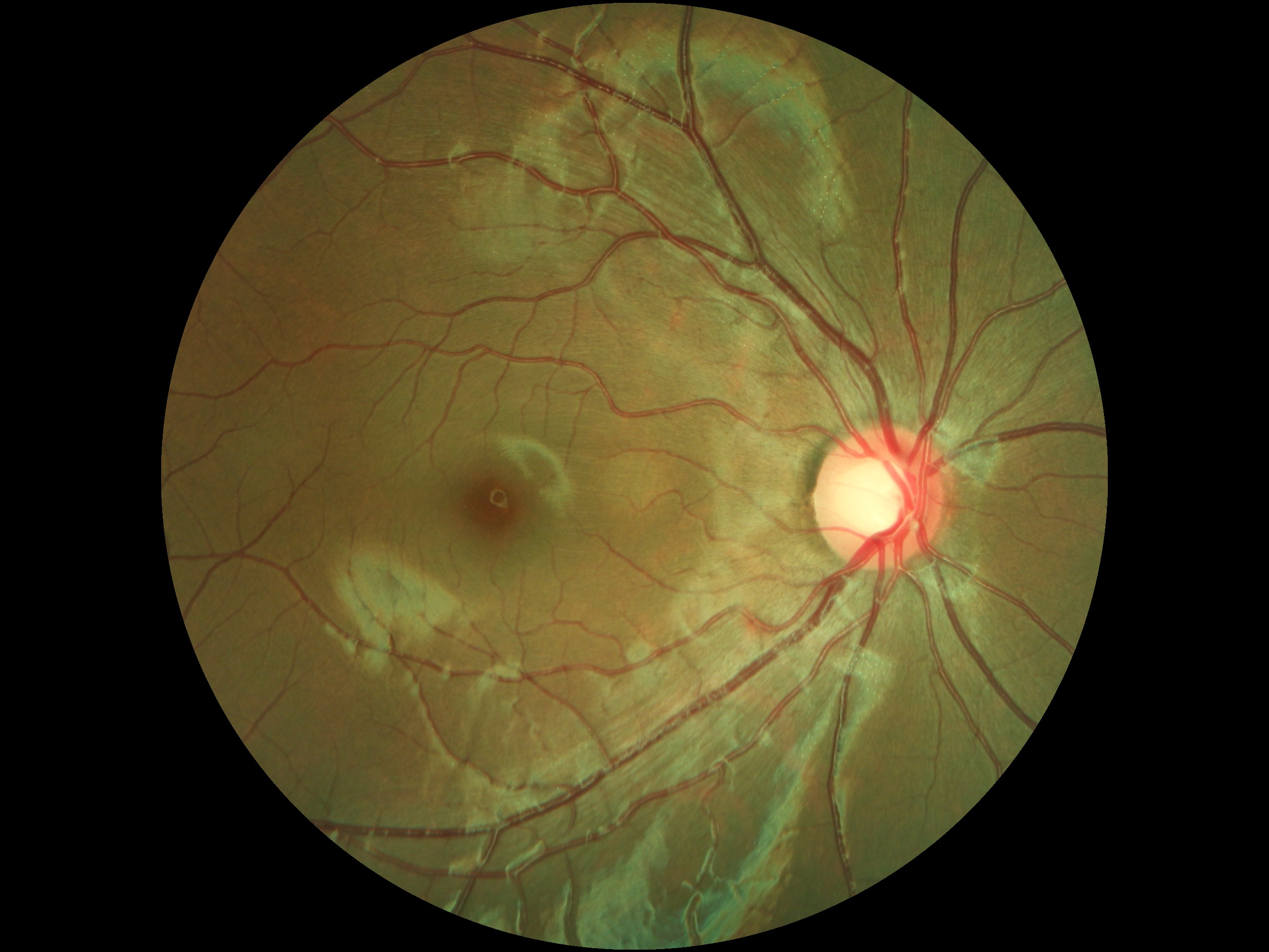

Supplement: S3 File — (ZIP) [file pone.0324352.s003.zip › Original fundus photographs (1)/Subject 45/OD_20230611506005_20230612112304_1.jpg]

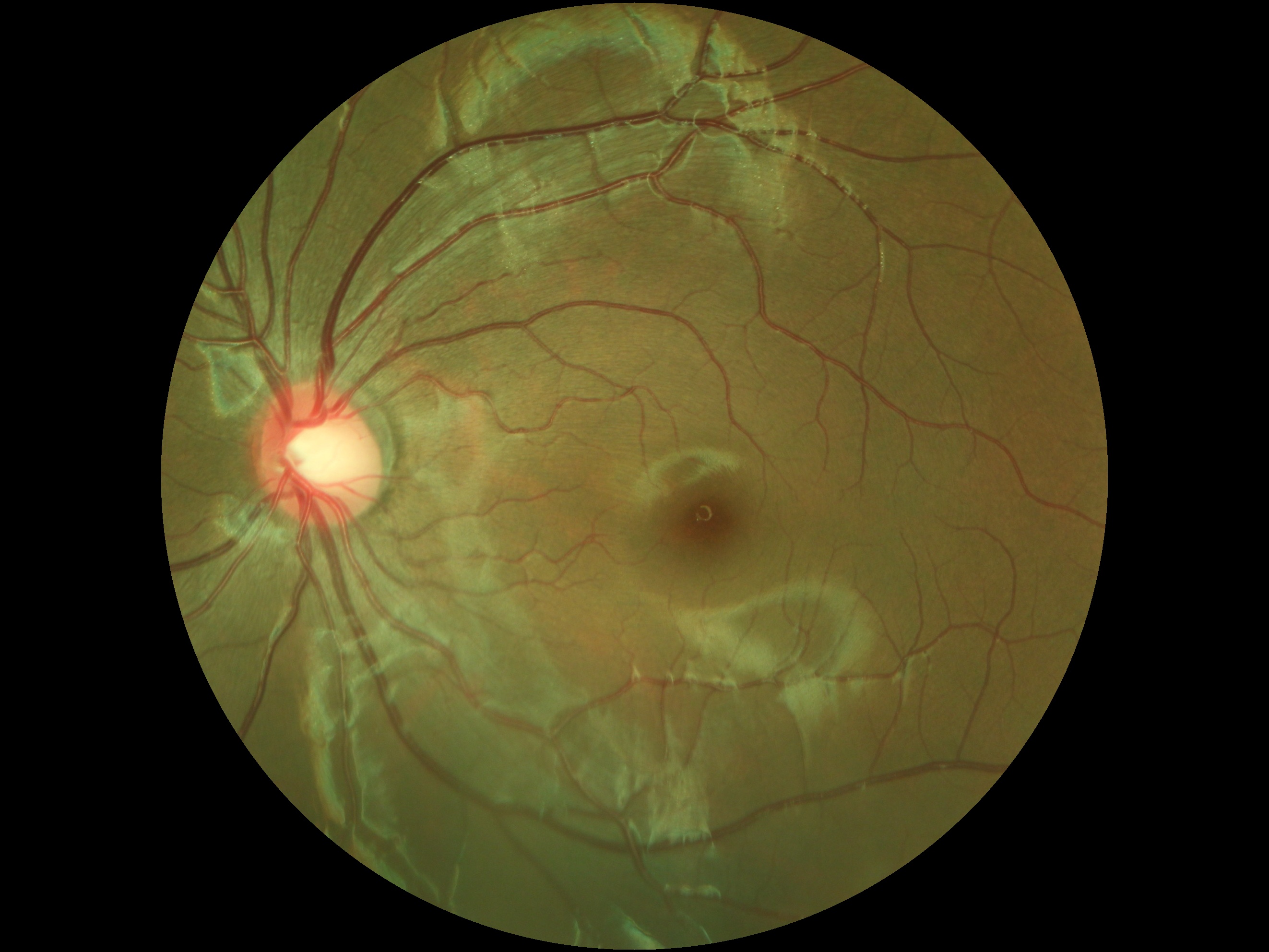

Supplement: S3 File — (ZIP) [file pone.0324352.s003.zip › Original fundus photographs (1)/Subject 45/OS_20230611506005_20230612112342_3.jpg]

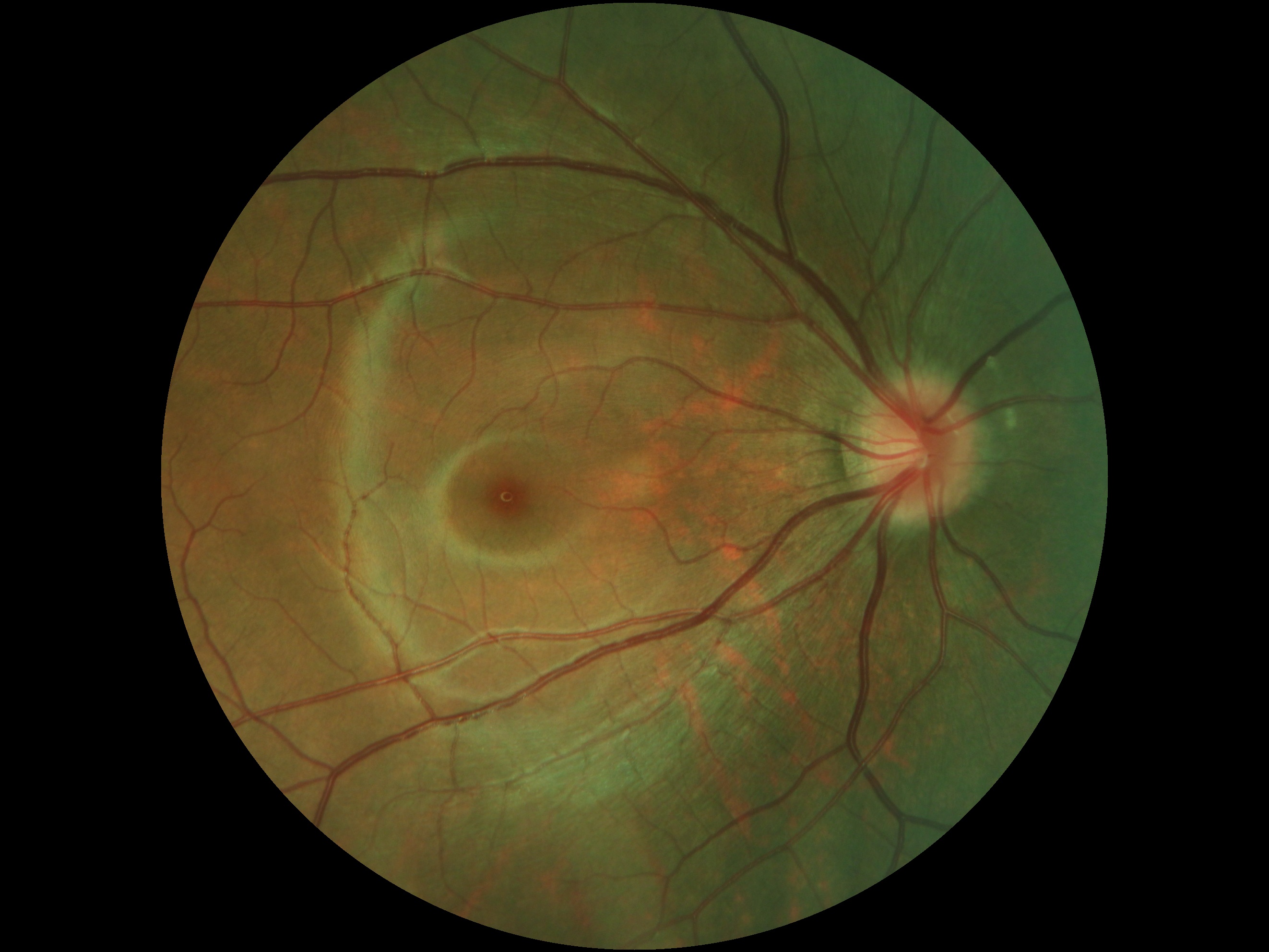

Supplement: S3 File — (ZIP) [file pone.0324352.s003.zip › Original fundus photographs (1)/Subject 46/OD_20230611640087_20230612153357_1.jpg]

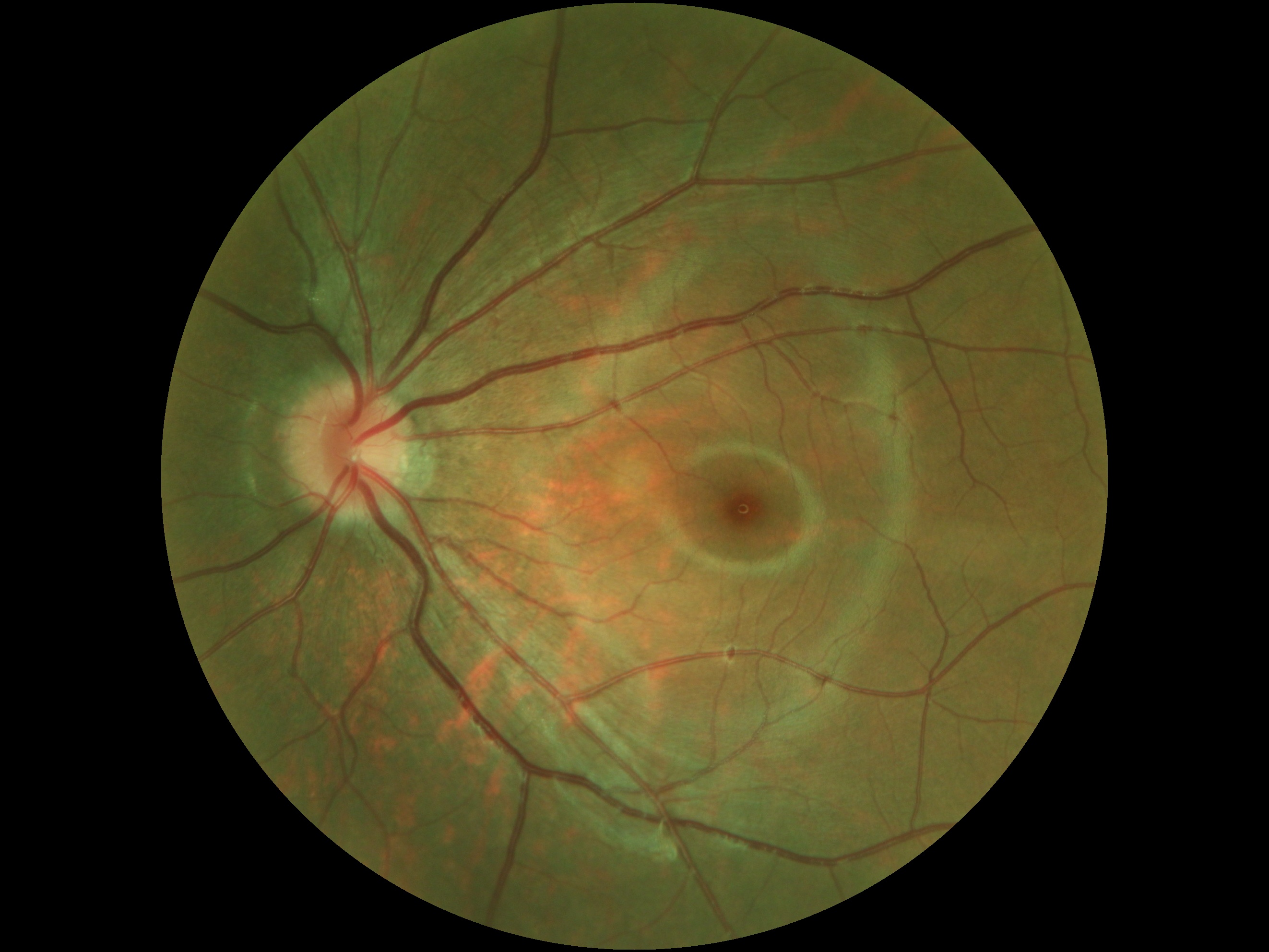

Supplement: S3 File — (ZIP) [file pone.0324352.s003.zip › Original fundus photographs (1)/Subject 46/OS_20230611640087_20230612153422_2.jpg]

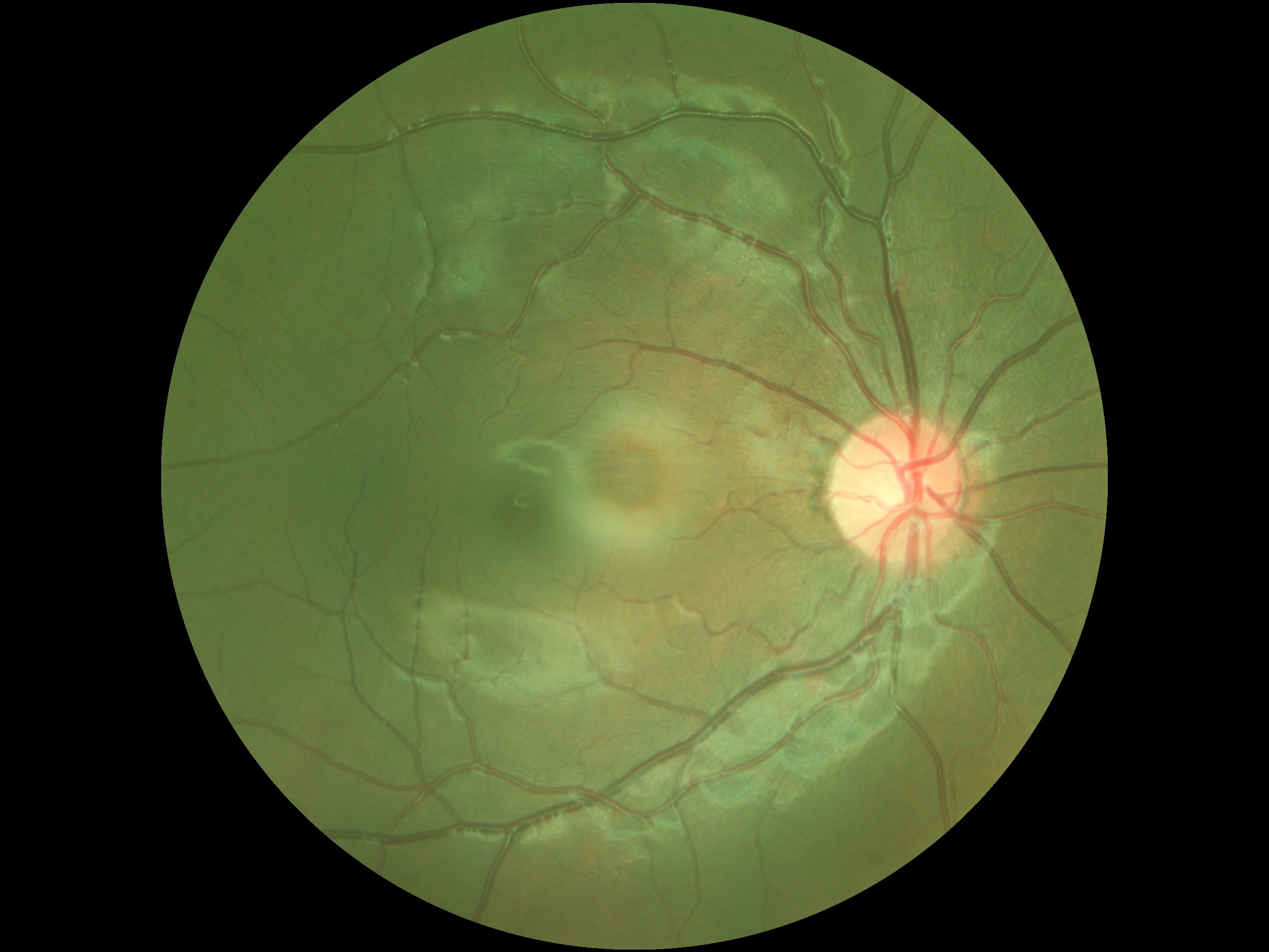

Supplement: S3 File — (ZIP) [file pone.0324352.s003.zip › Original fundus photographs (1)/Subject 47/OD_20230611898265_20230615104315_1.jpg]

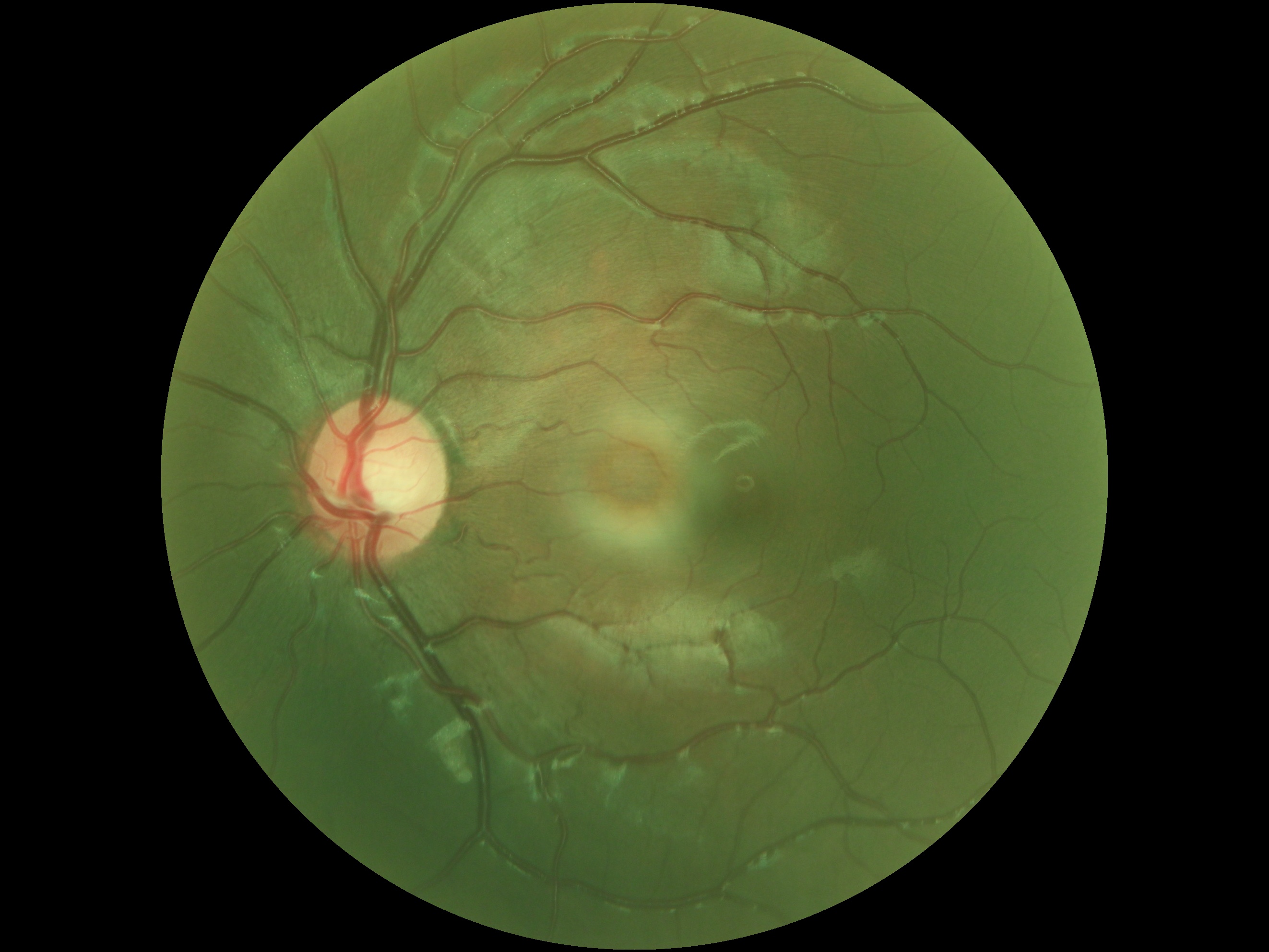

Supplement: S3 File — (ZIP) [file pone.0324352.s003.zip › Original fundus photographs (1)/Subject 47/OS_20230611898265_20230615104337_2.jpg]

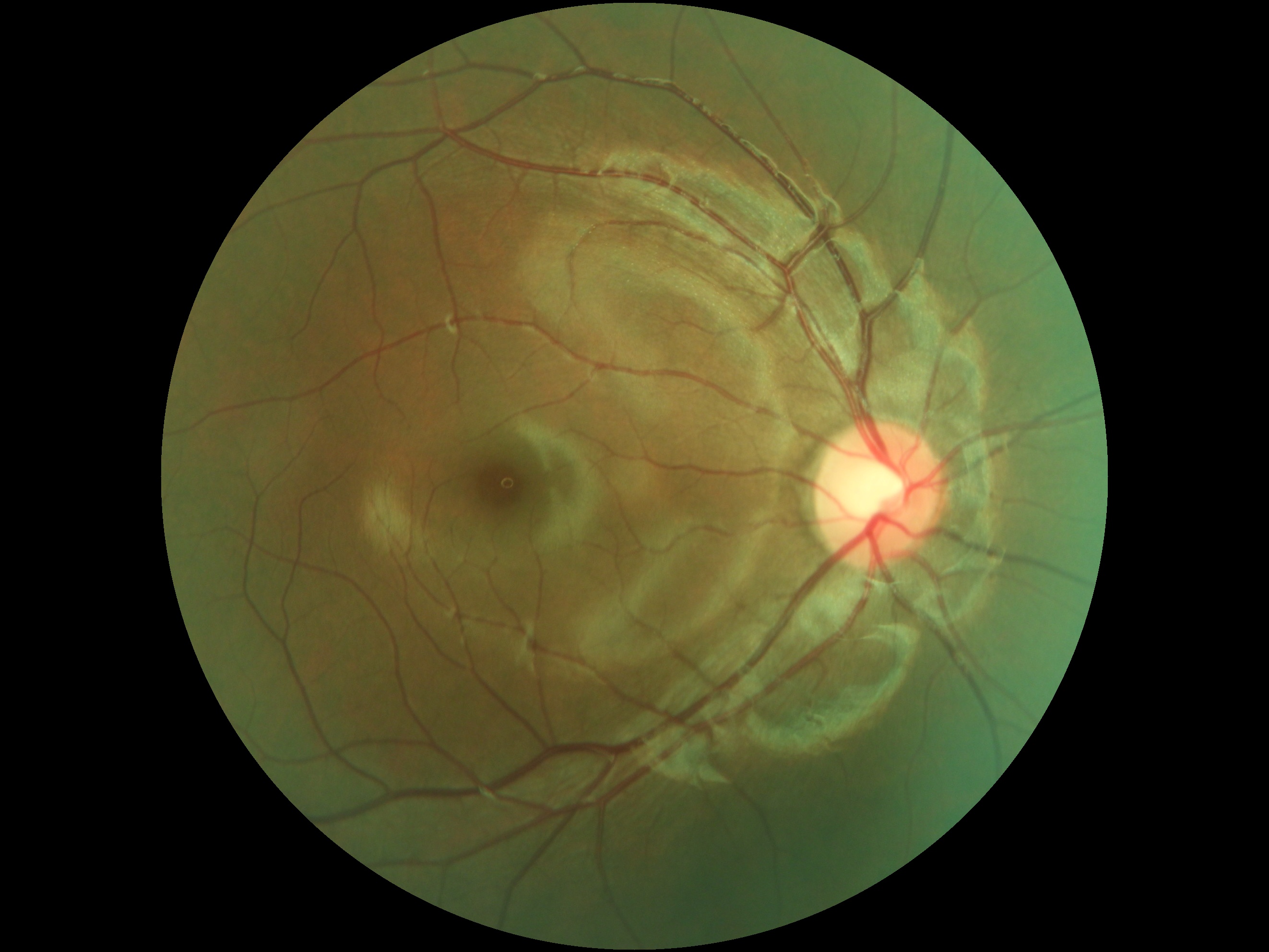

Supplement: S3 File — (ZIP) [file pone.0324352.s003.zip › Original fundus photographs (1)/Subject 48/OD_20230611132089_20230612154006_1.jpg]

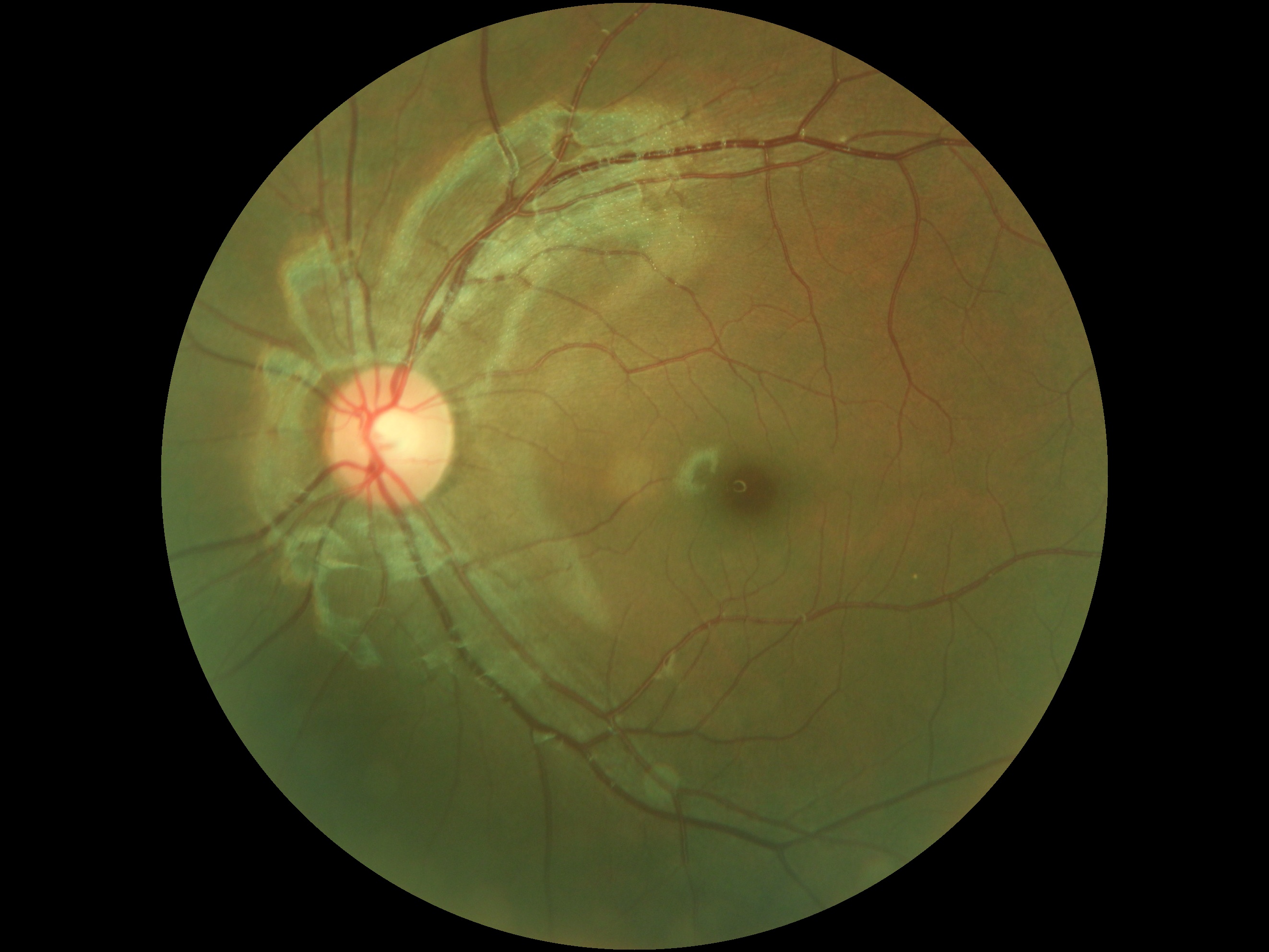

Supplement: S3 File — (ZIP) [file pone.0324352.s003.zip › Original fundus photographs (1)/Subject 48/OS_20230611132089_20230612154040_2.jpg]

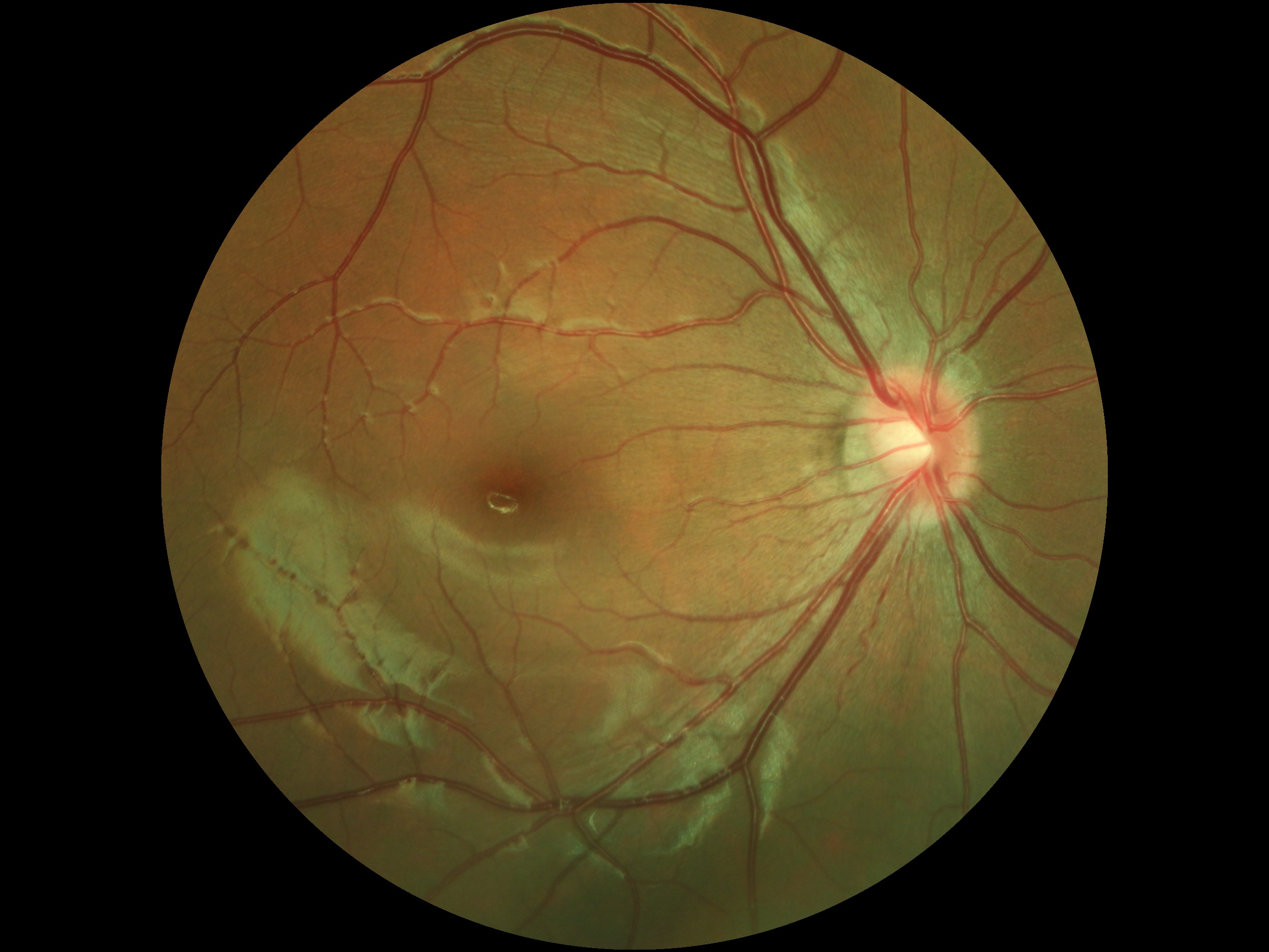

Supplement: S3 File — (ZIP) [file pone.0324352.s003.zip › Original fundus photographs (1)/Subject 49/OD_20230611358004_20230612111809_1.jpg]

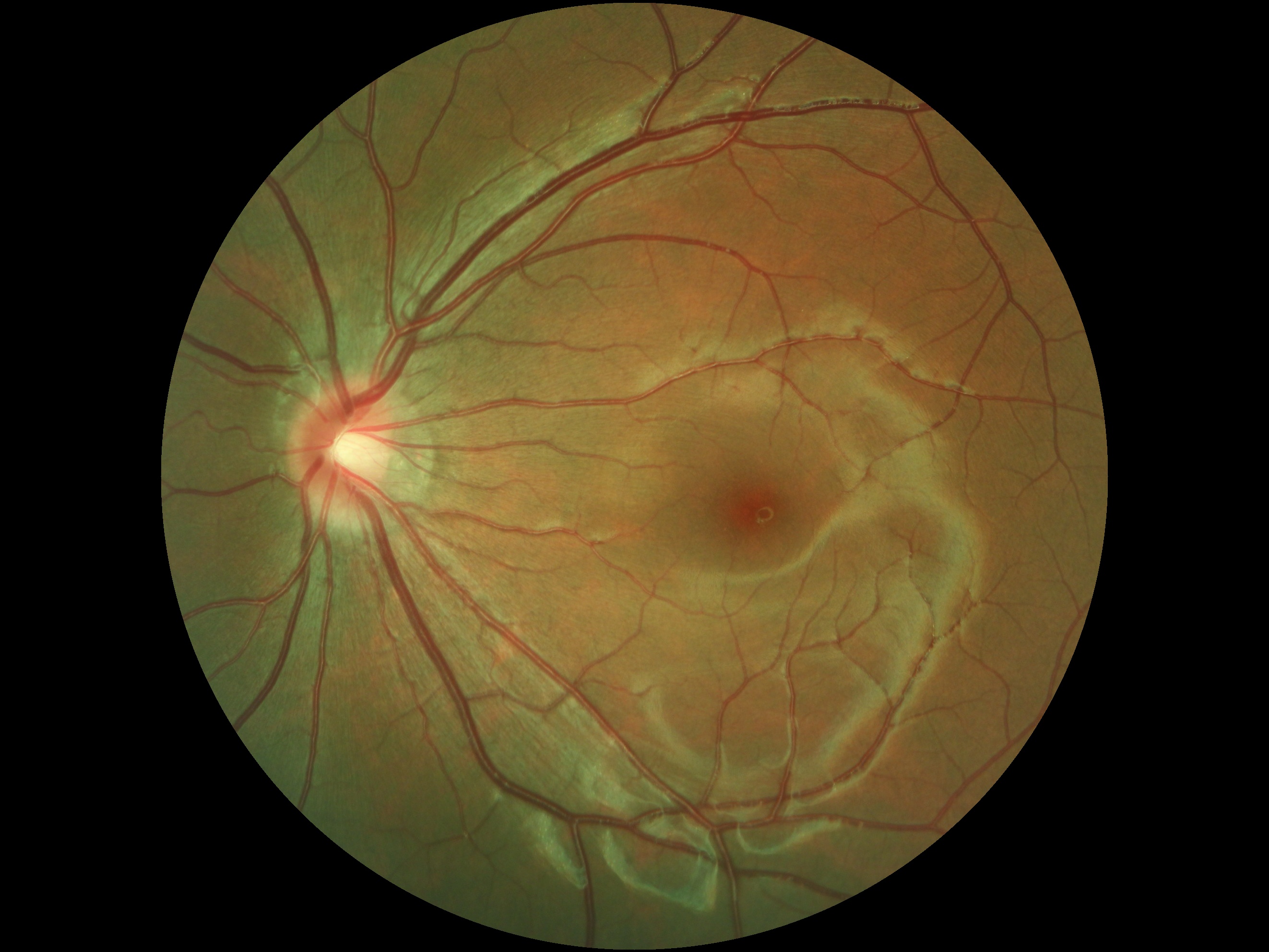

Supplement: S3 File — (ZIP) [file pone.0324352.s003.zip › Original fundus photographs (1)/Subject 49/OS_20230611358004_20230612111905_2.jpg]

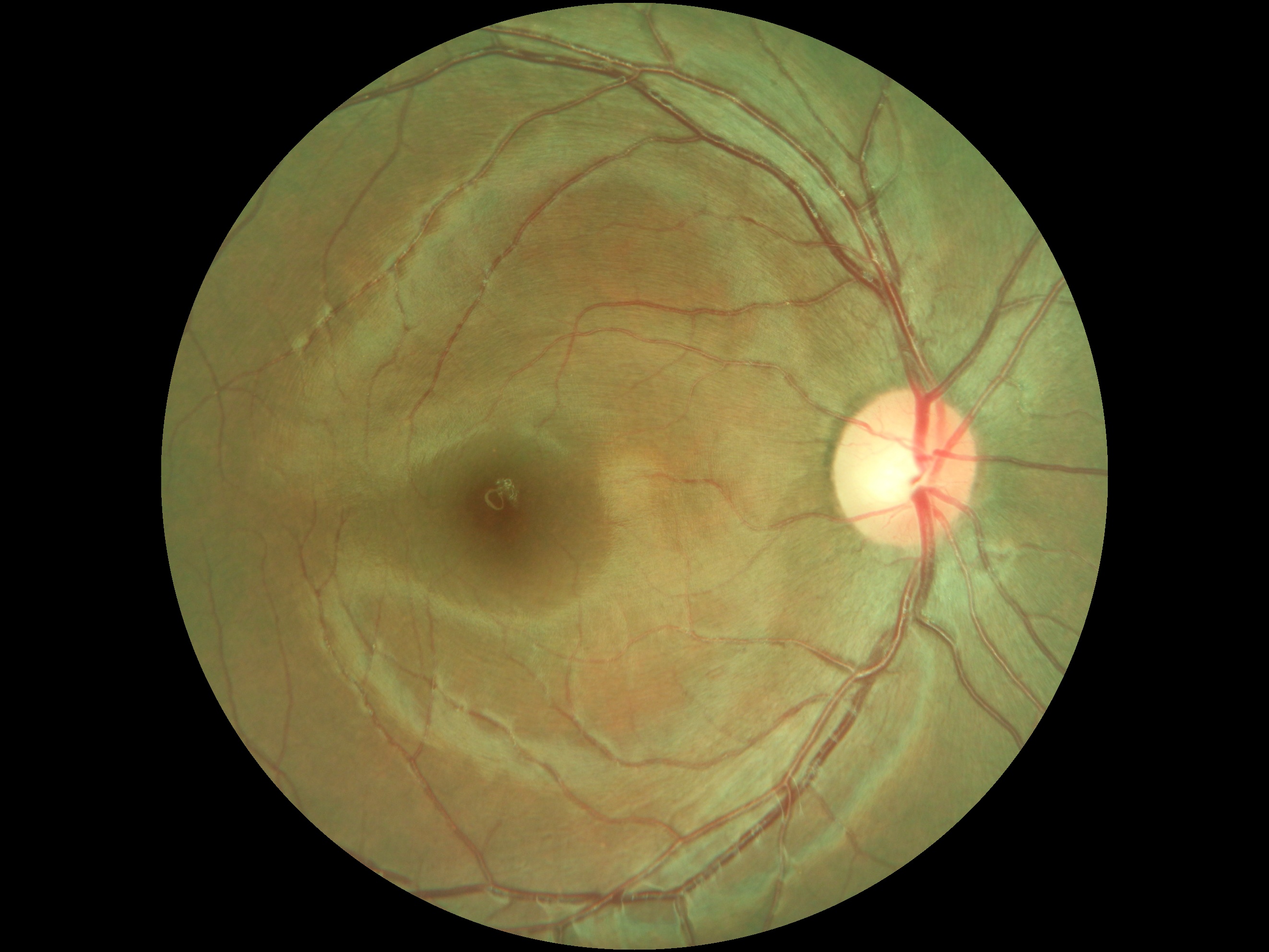

Supplement: S3 File — (ZIP) [file pone.0324352.s003.zip › Original fundus photographs (1)/Subject 5/OD_20230611676003_20230612151942_1.jpg]

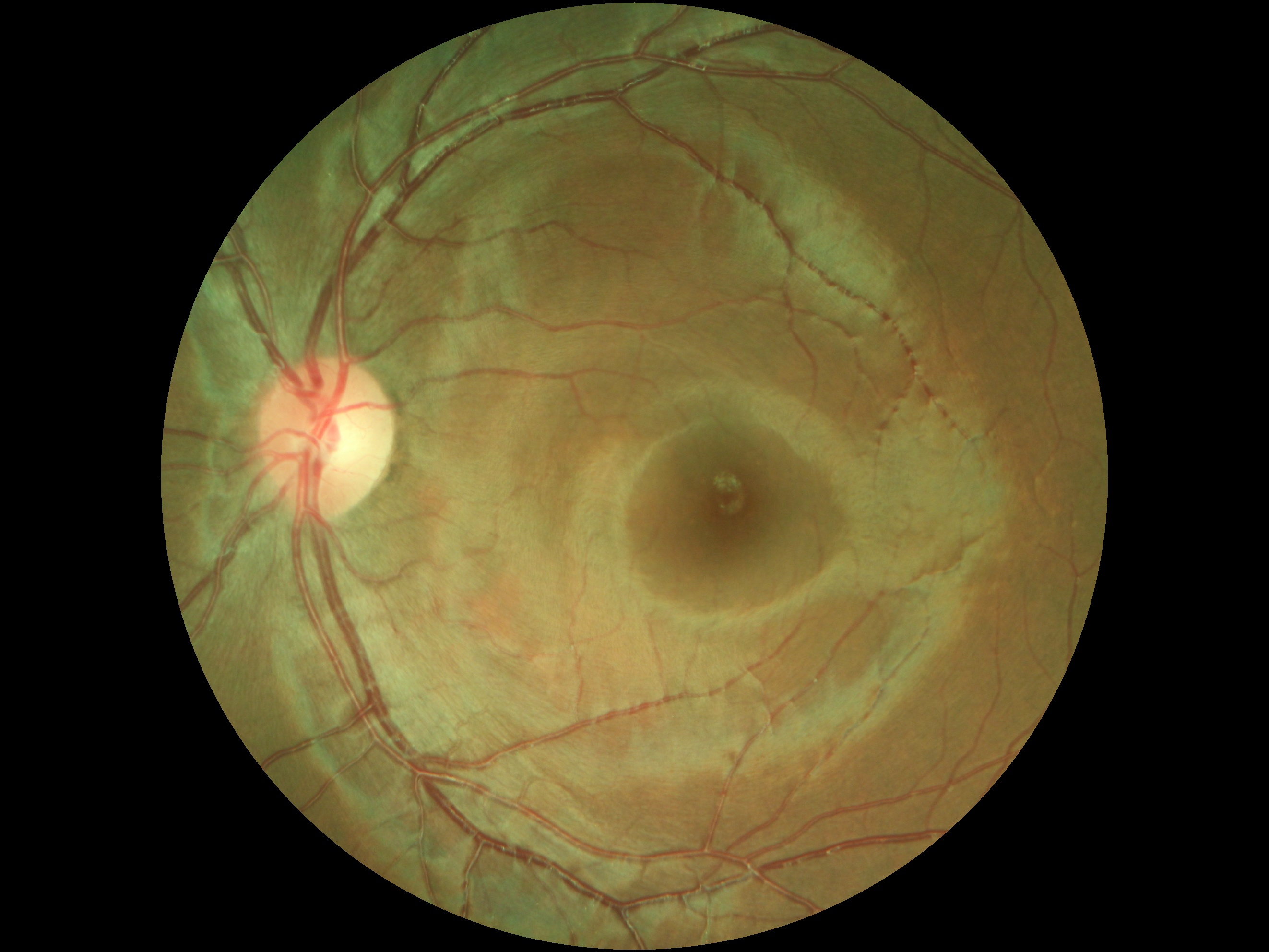

Supplement: S3 File — (ZIP) [file pone.0324352.s003.zip › Original fundus photographs (1)/Subject 5/OS_20230611676003_20230612152209_3.jpg]

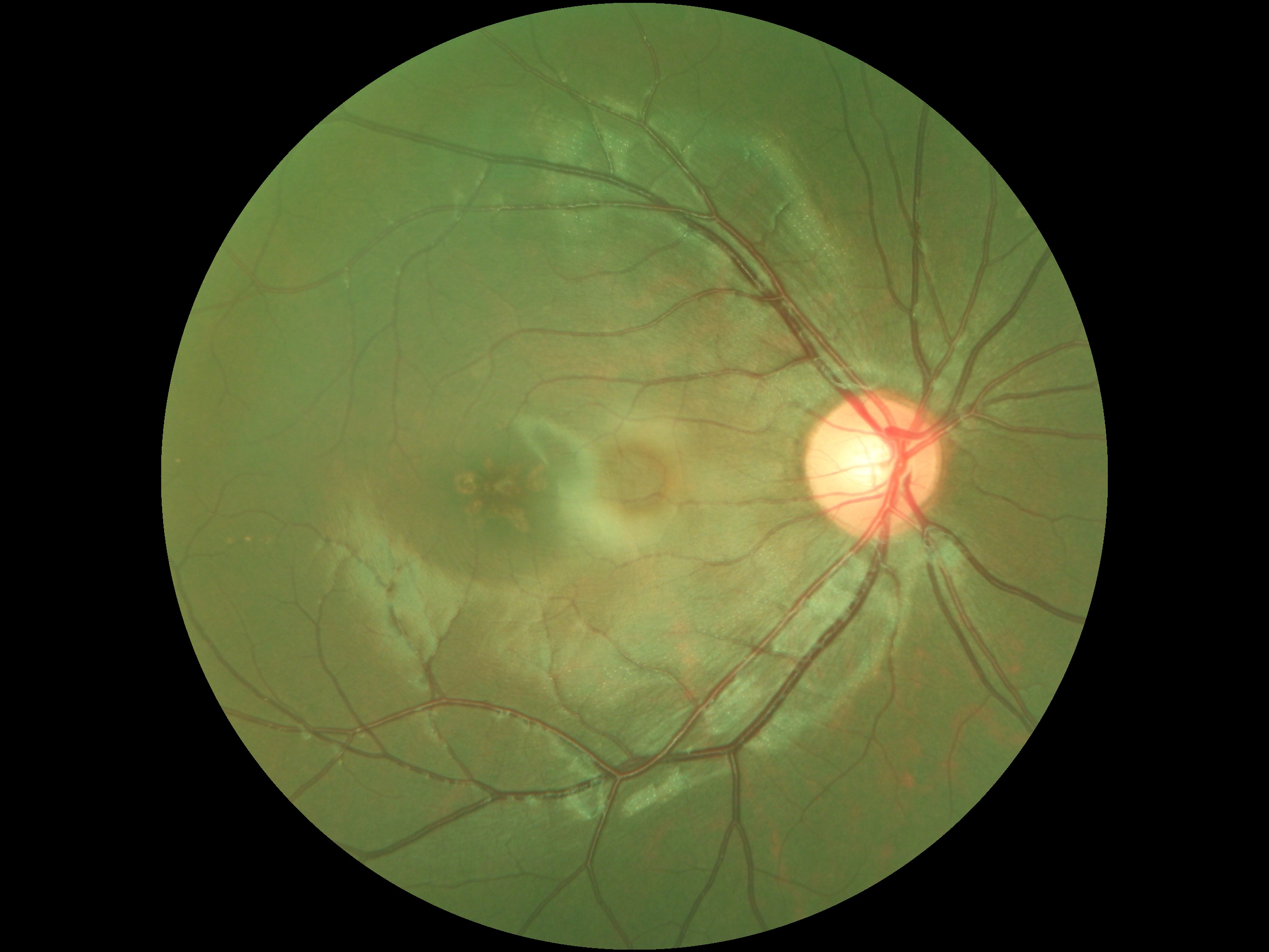

Supplement: S3 File — (ZIP) [file pone.0324352.s003.zip › Original fundus photographs (1)/Subject 50/OD_20230611585200_20230615112751_2.jpg]

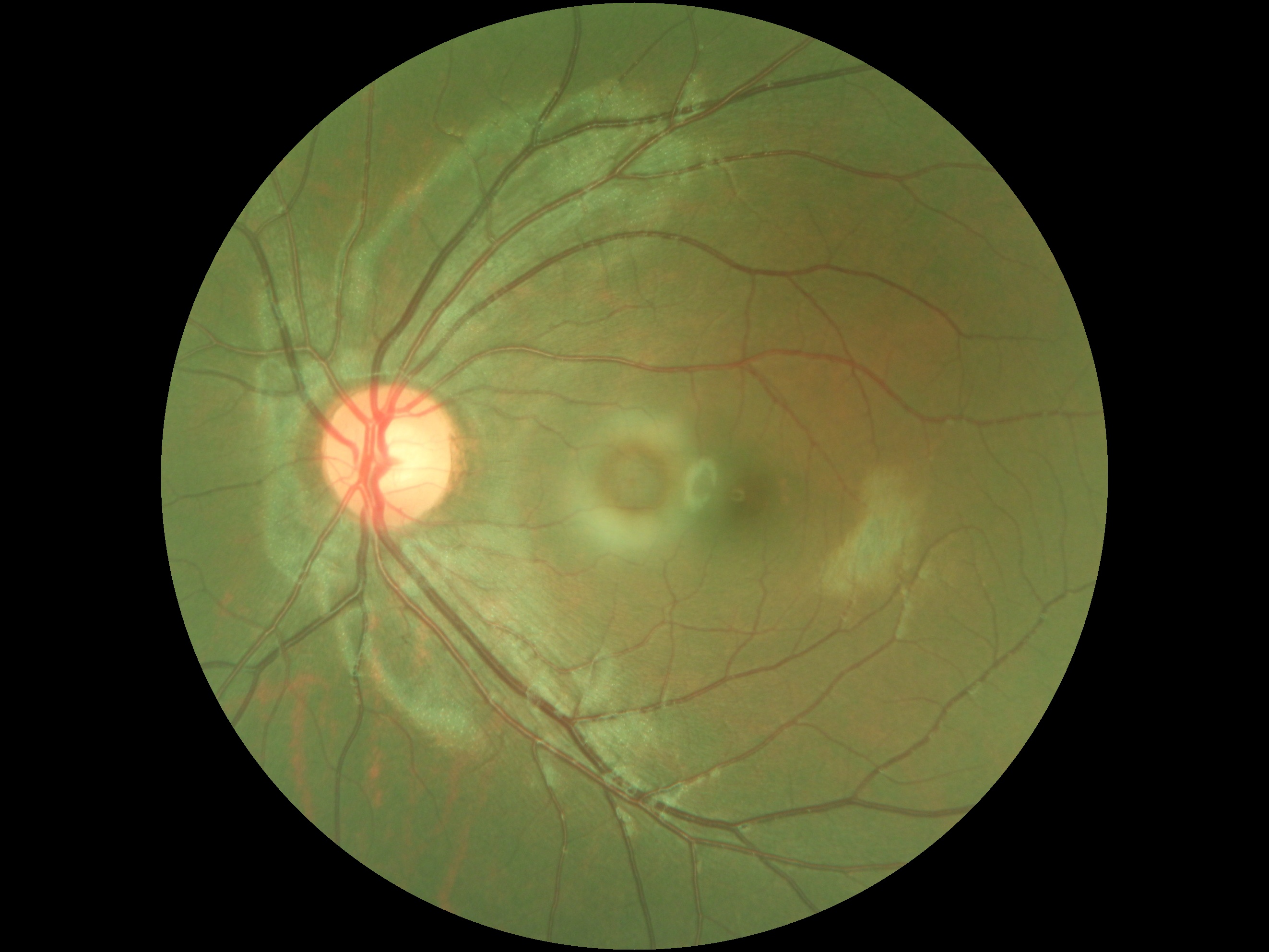

Supplement: S3 File — (ZIP) [file pone.0324352.s003.zip › Original fundus photographs (1)/Subject 50/OS_20230611585200_20230615112735_1.jpg]

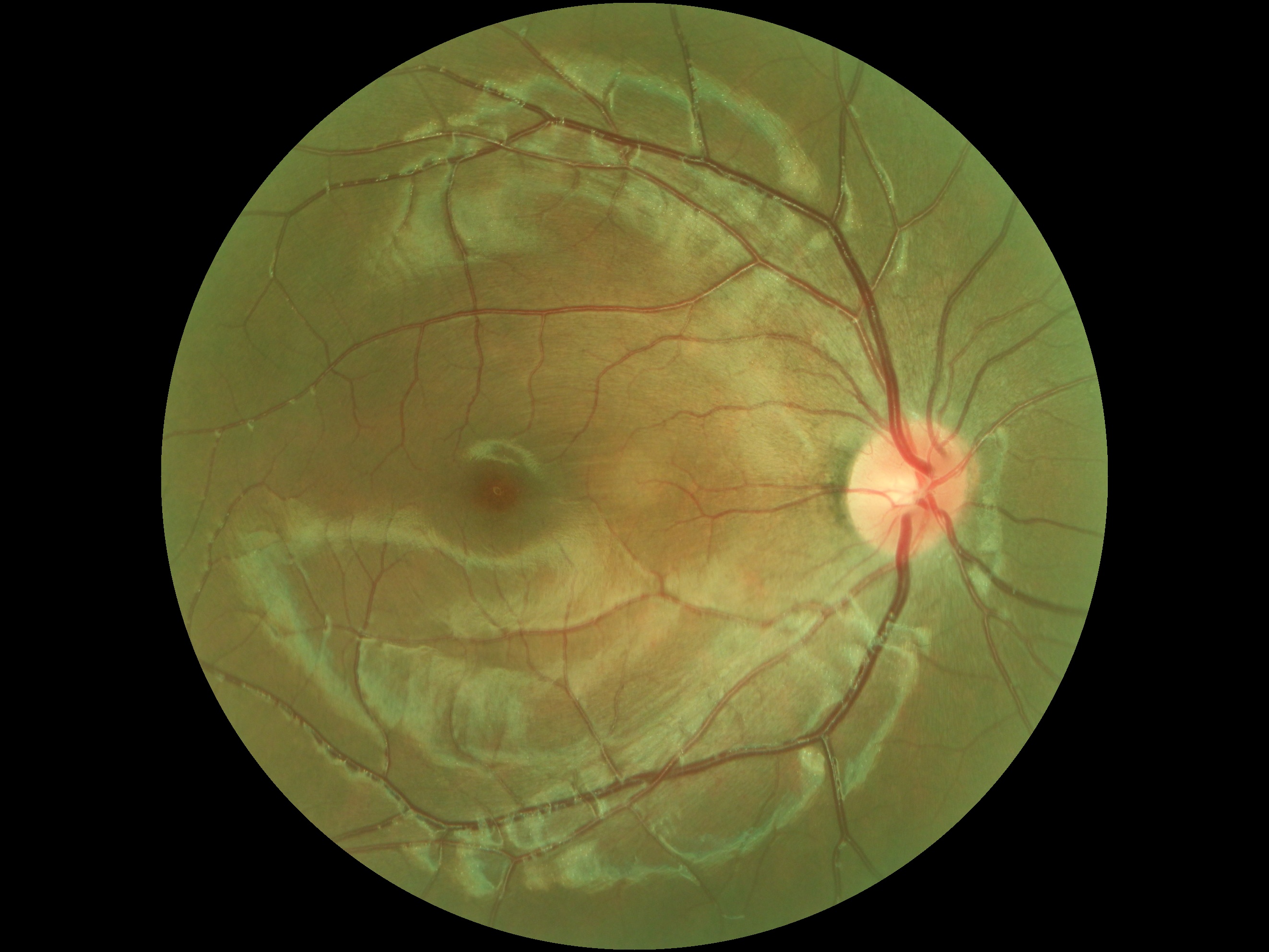

Supplement: S3 File — (ZIP) [file pone.0324352.s003.zip › Original fundus photographs (1)/Subject 51/OD_20230611823073_20230612163013_1.jpg]

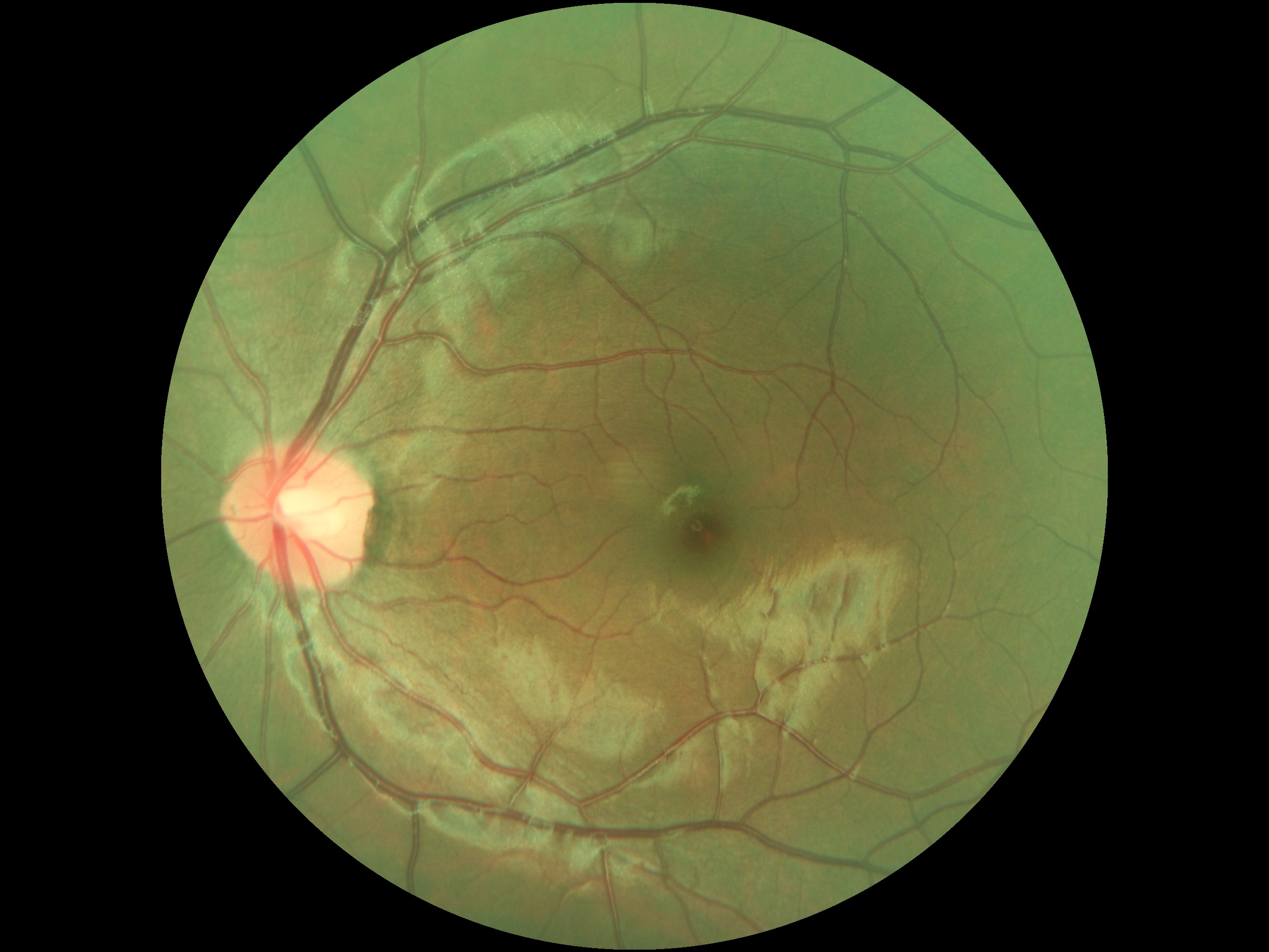

Supplement: S3 File — (ZIP) [file pone.0324352.s003.zip › Original fundus photographs (1)/Subject 51/OS_20230611823073_20230612163036_2.jpg]

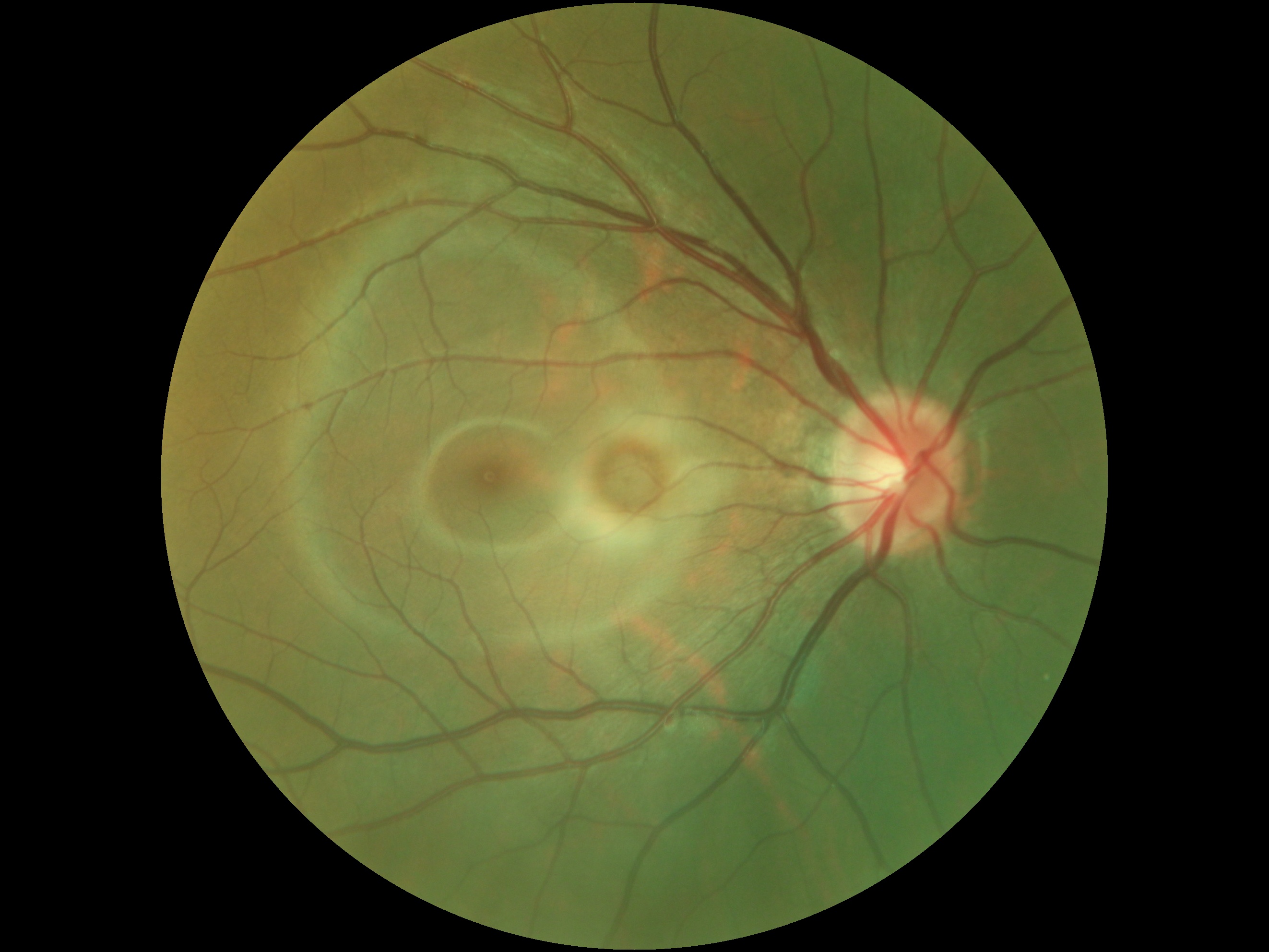

Supplement: S3 File — (ZIP) [file pone.0324352.s003.zip › Original fundus photographs (1)/Subject 52/OD_20230611182134_20230614104142_1.jpg]

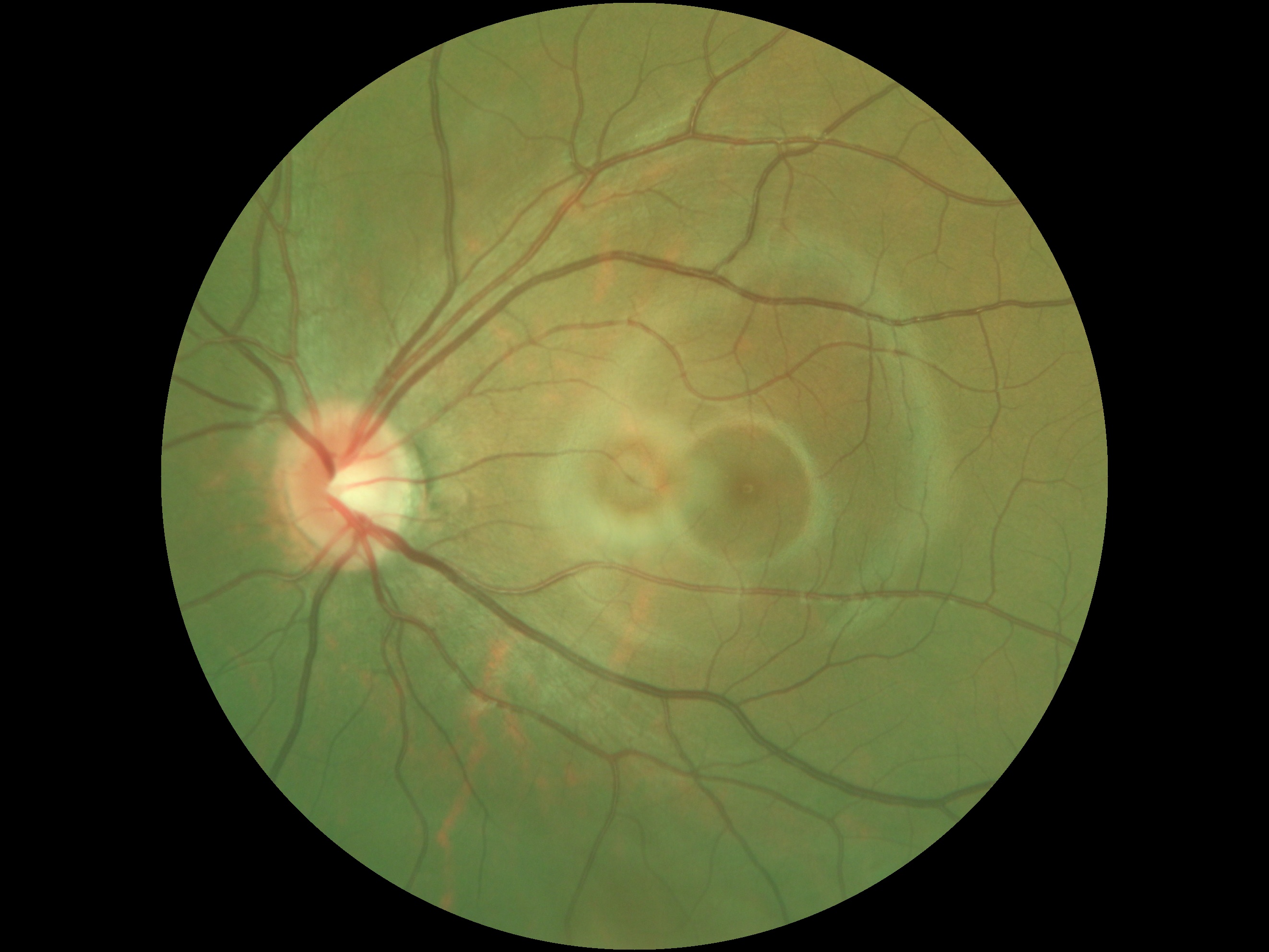

Supplement: S3 File — (ZIP) [file pone.0324352.s003.zip › Original fundus photographs (1)/Subject 52/OS_20230611182134_20230614104206_2.jpg]

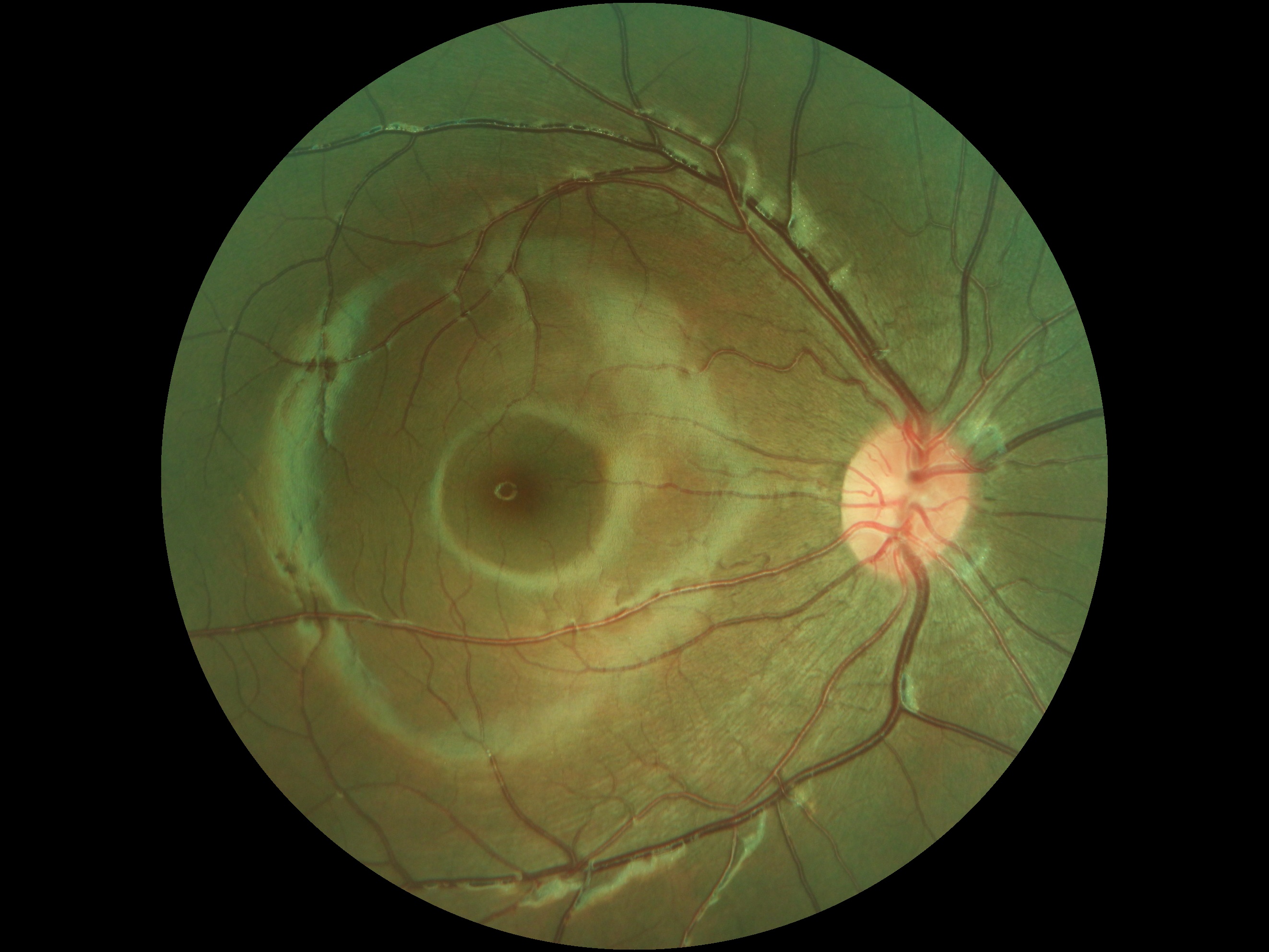

Supplement: S3 File — (ZIP) [file pone.0324352.s003.zip › Original fundus photographs (1)/Subject 53/OD_20230611578074_20230612163224_1.jpg]

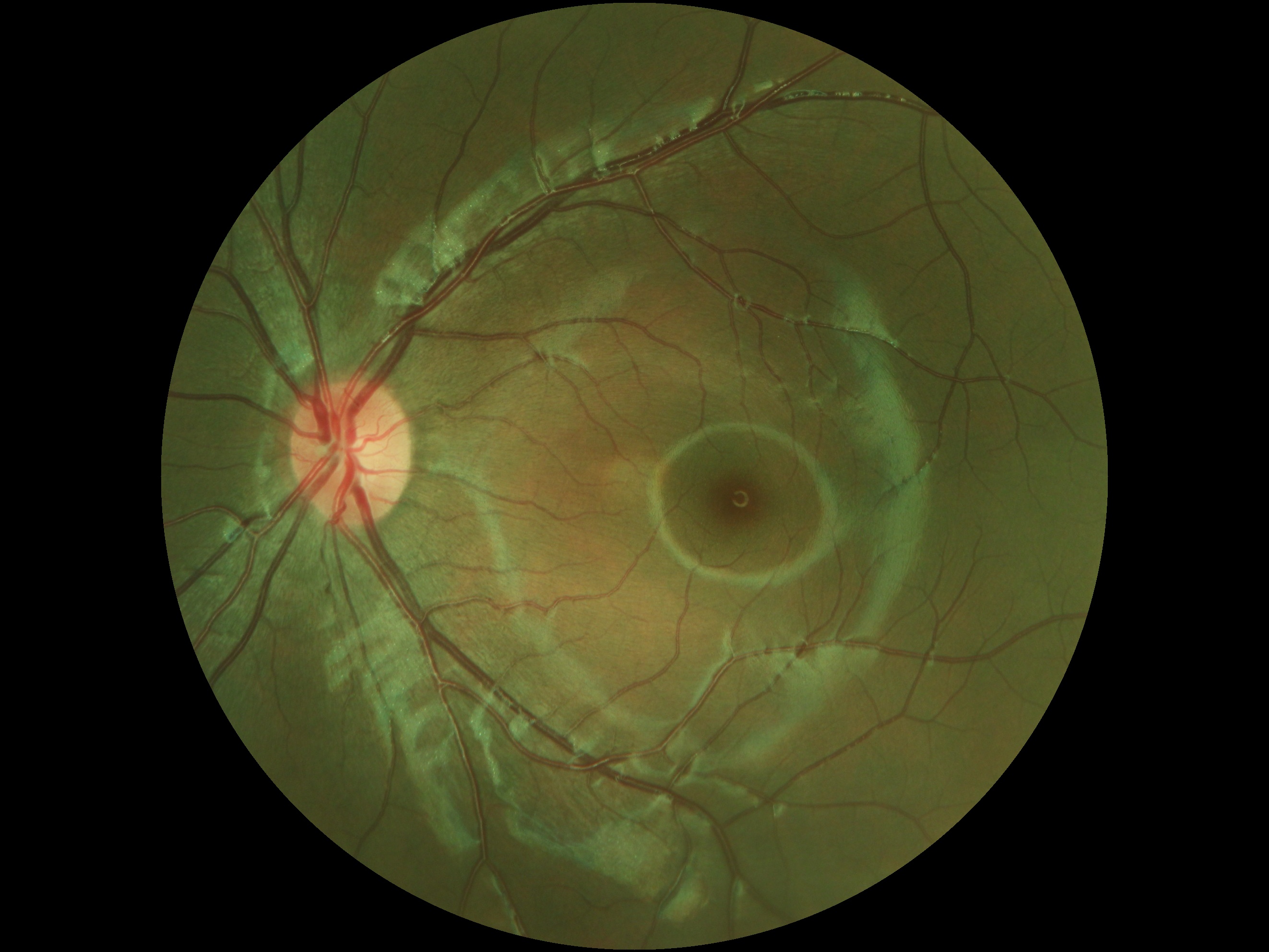

Supplement: S3 File — (ZIP) [file pone.0324352.s003.zip › Original fundus photographs (1)/Subject 53/OS_20230611578074_20230612163304_2.jpg]

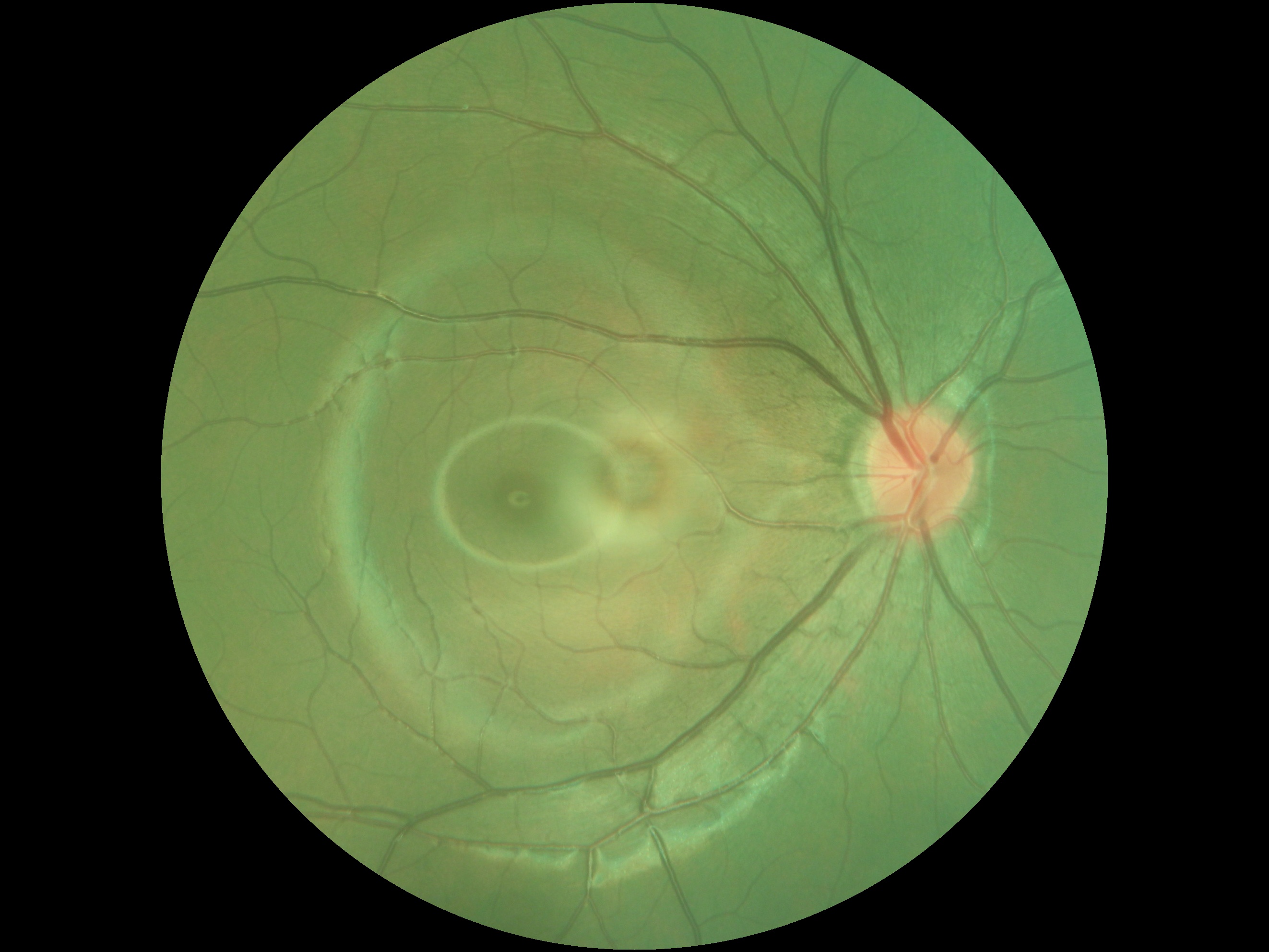

Supplement: S3 File — (ZIP) [file pone.0324352.s003.zip › Original fundus photographs (1)/Subject 54/OD_20230615832081_20230615160454_2.jpg]

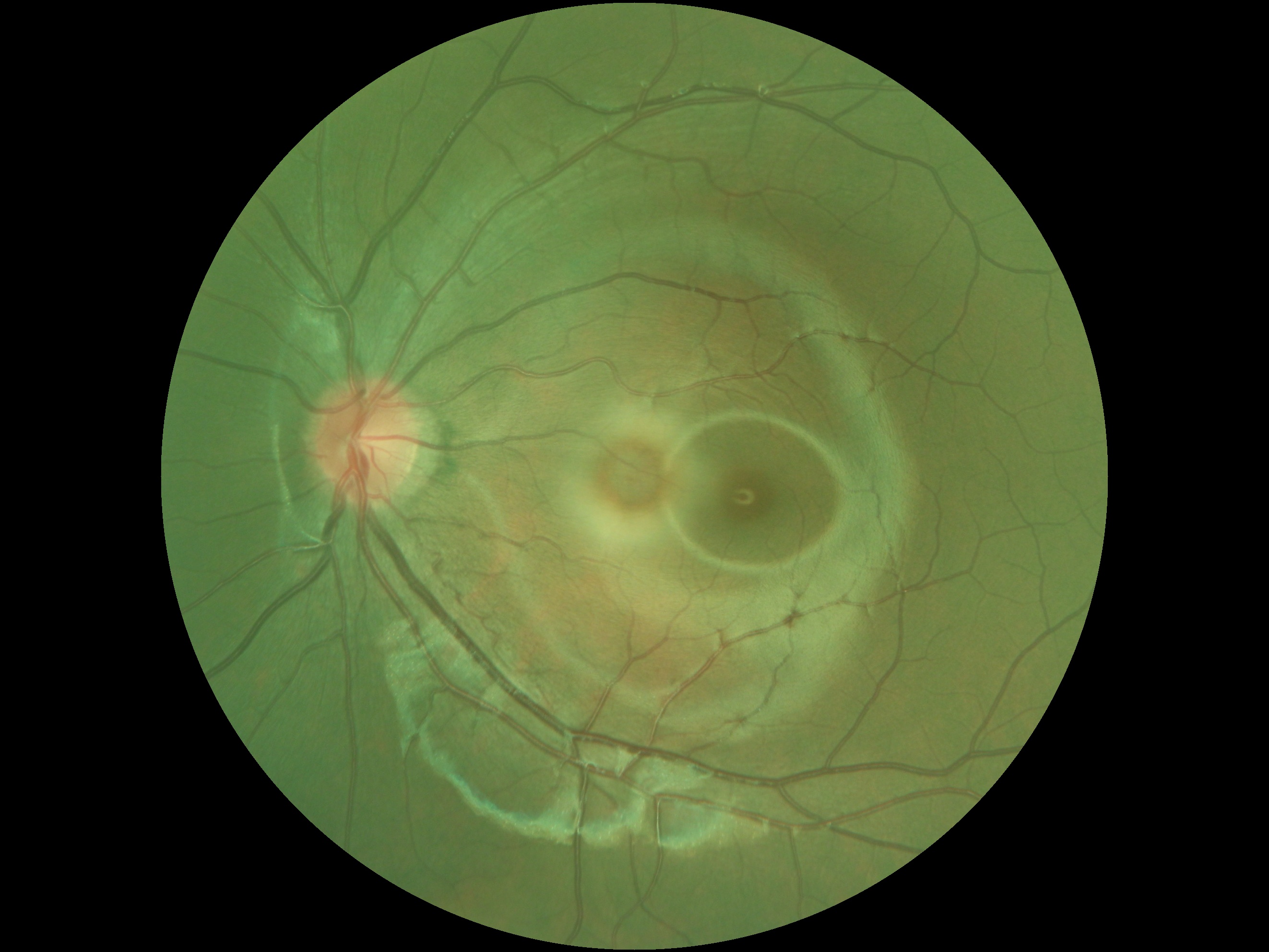

Supplement: S3 File — (ZIP) [file pone.0324352.s003.zip › Original fundus photographs (1)/Subject 54/OS_20230615832081_20230615160429_1.jpg]
