# Supplementary figures and images for: Research on the correlation between retinal vascular parameters and axial length in children using an AI-based fundus image analysis system (part 3 of 3)
Source: PLoS One. 2025 Jun 17;20(6):e0324352. doi: 10.1371/journal.pone.0324352 (PMC12173413; doi:10.1371/journal.pone.0324352)

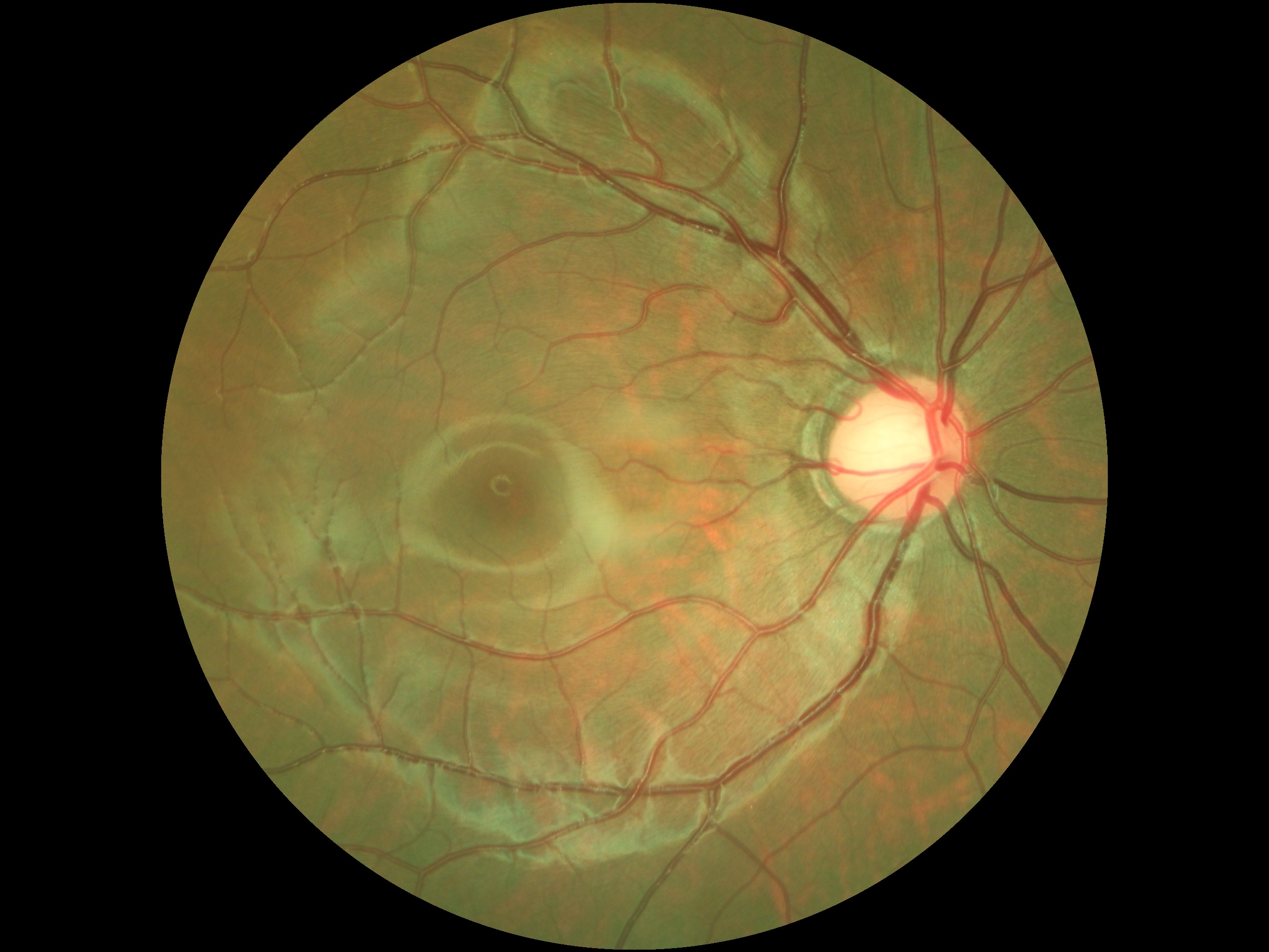

Supplement: S4 File — (ZIP) [file pone.0324352.s004.zip › Original fundus photographs (2)/Subject 76/OD_20230615452066_20230615154304_1.jpg]

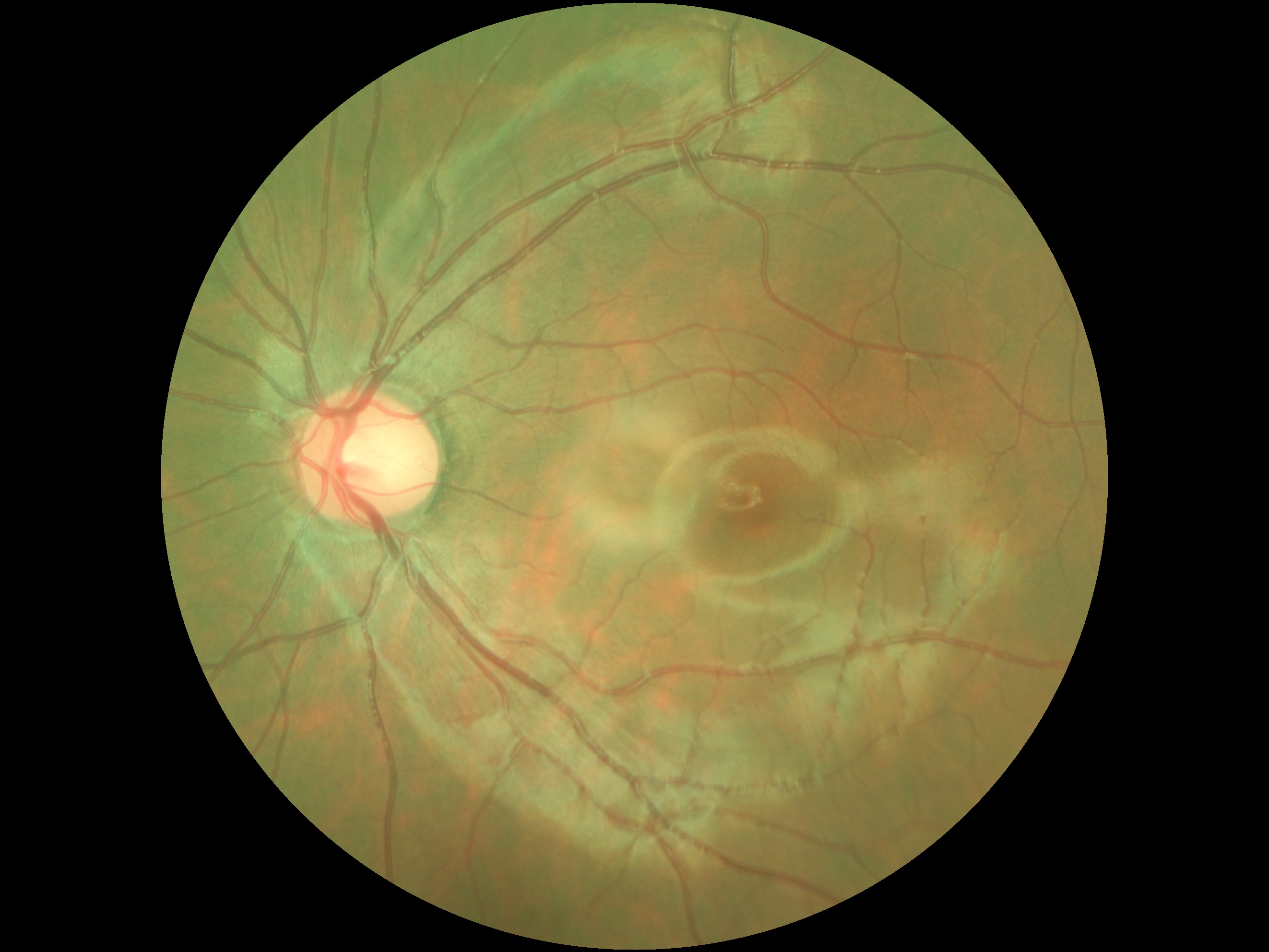

Supplement: S4 File — (ZIP) [file pone.0324352.s004.zip › Original fundus photographs (2)/Subject 76/OS_20230615452066_20230615154412_4.jpg]

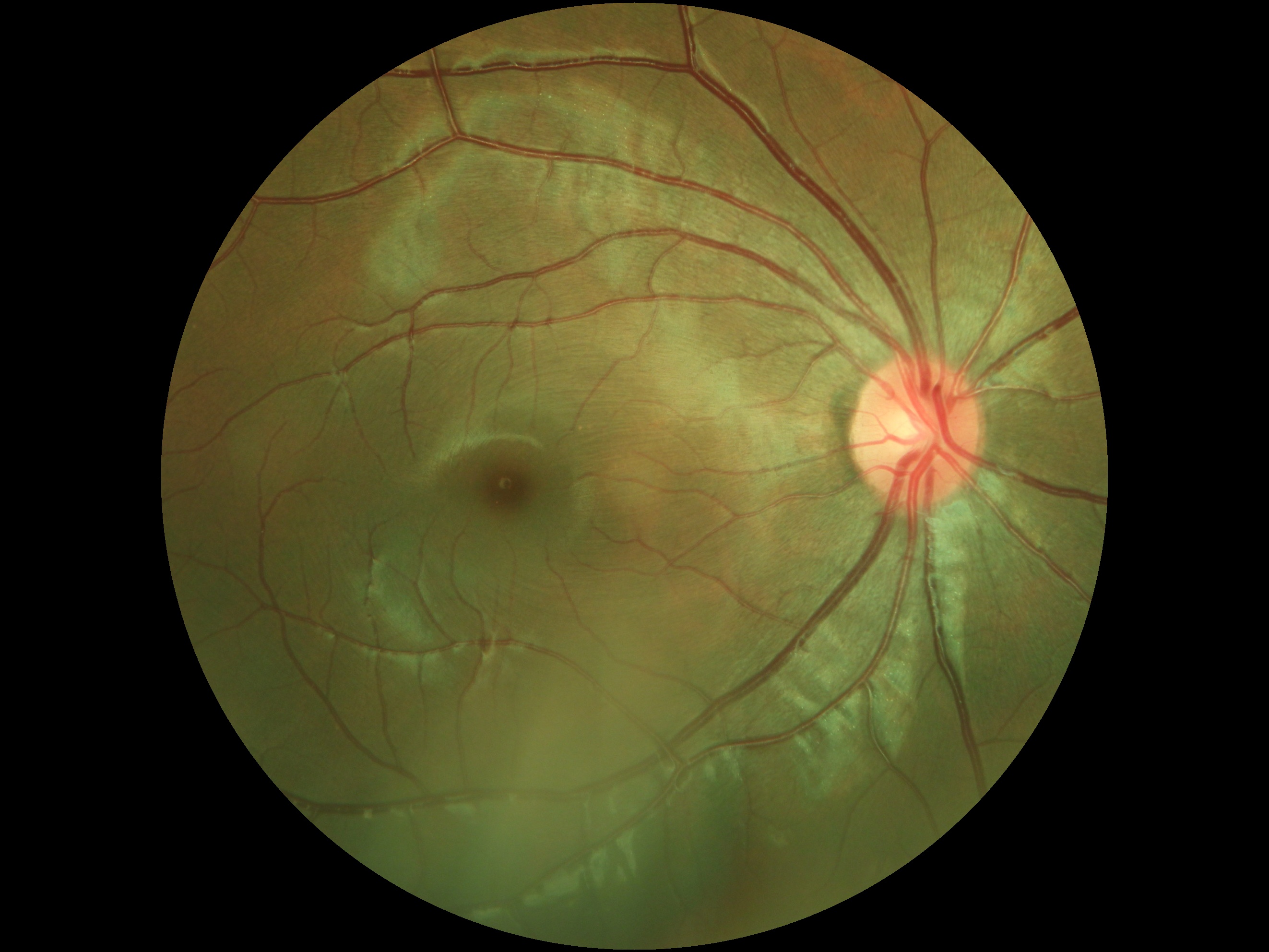

Supplement: S4 File — (ZIP) [file pone.0324352.s004.zip › Original fundus photographs (2)/Subject 77/OD_20230611268024_20230612164109_1.jpg]

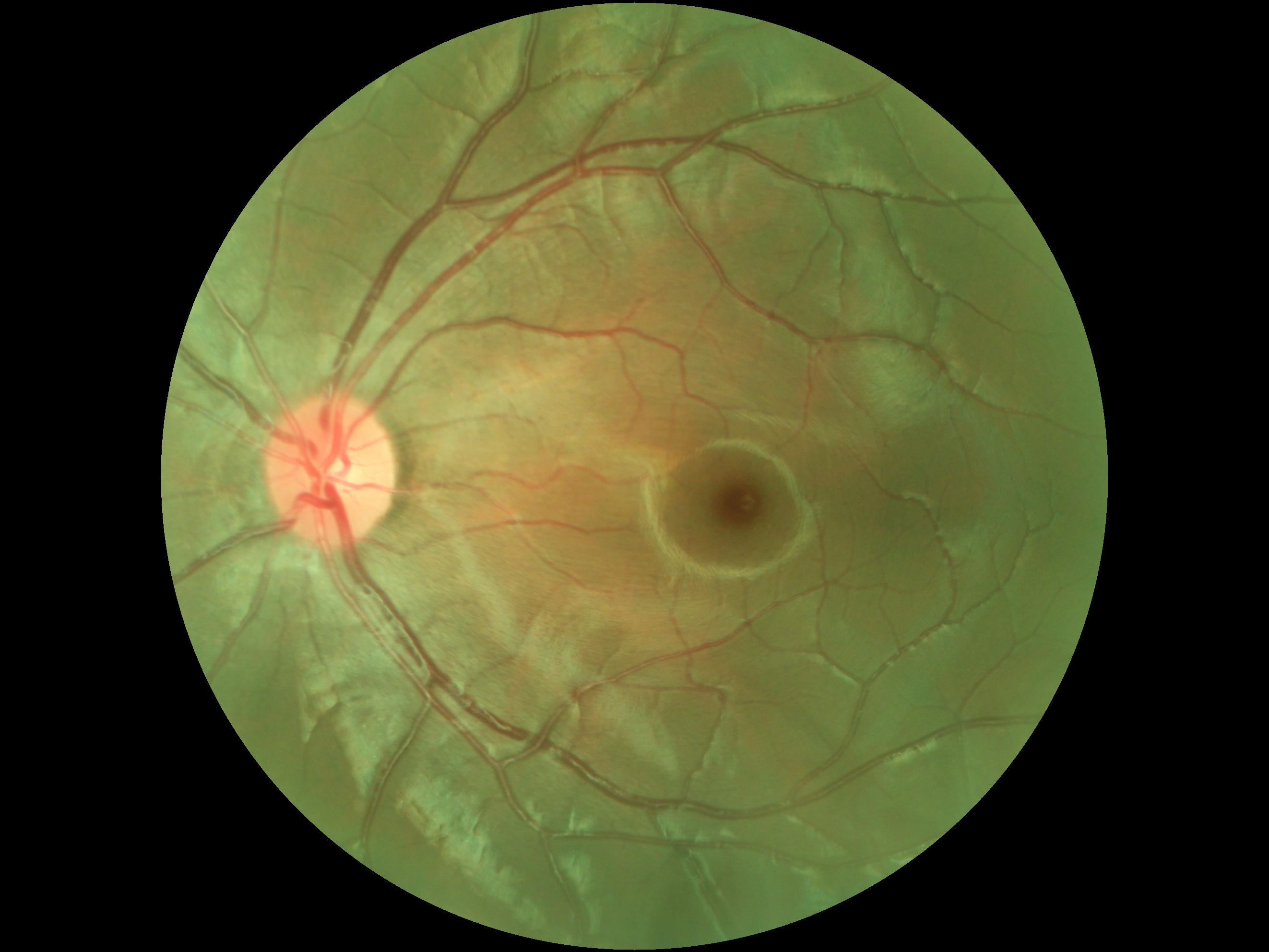

Supplement: S4 File — (ZIP) [file pone.0324352.s004.zip › Original fundus photographs (2)/Subject 77/OS_20230611268024_20230612164205_2.jpg]

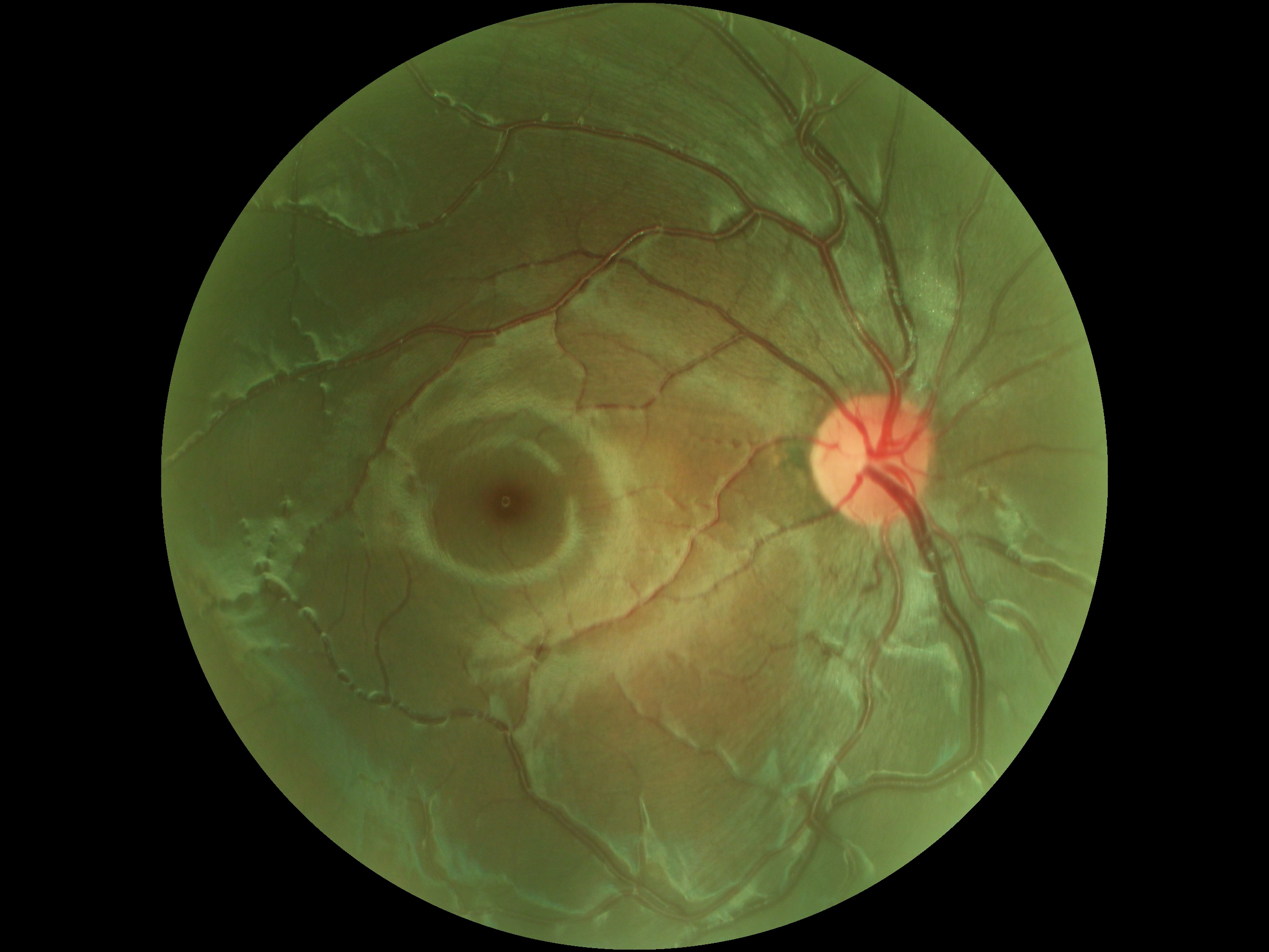

Supplement: S4 File — (ZIP) [file pone.0324352.s004.zip › Original fundus photographs (2)/Subject 78/OD_20230611822049_20230613103908_1.jpg]

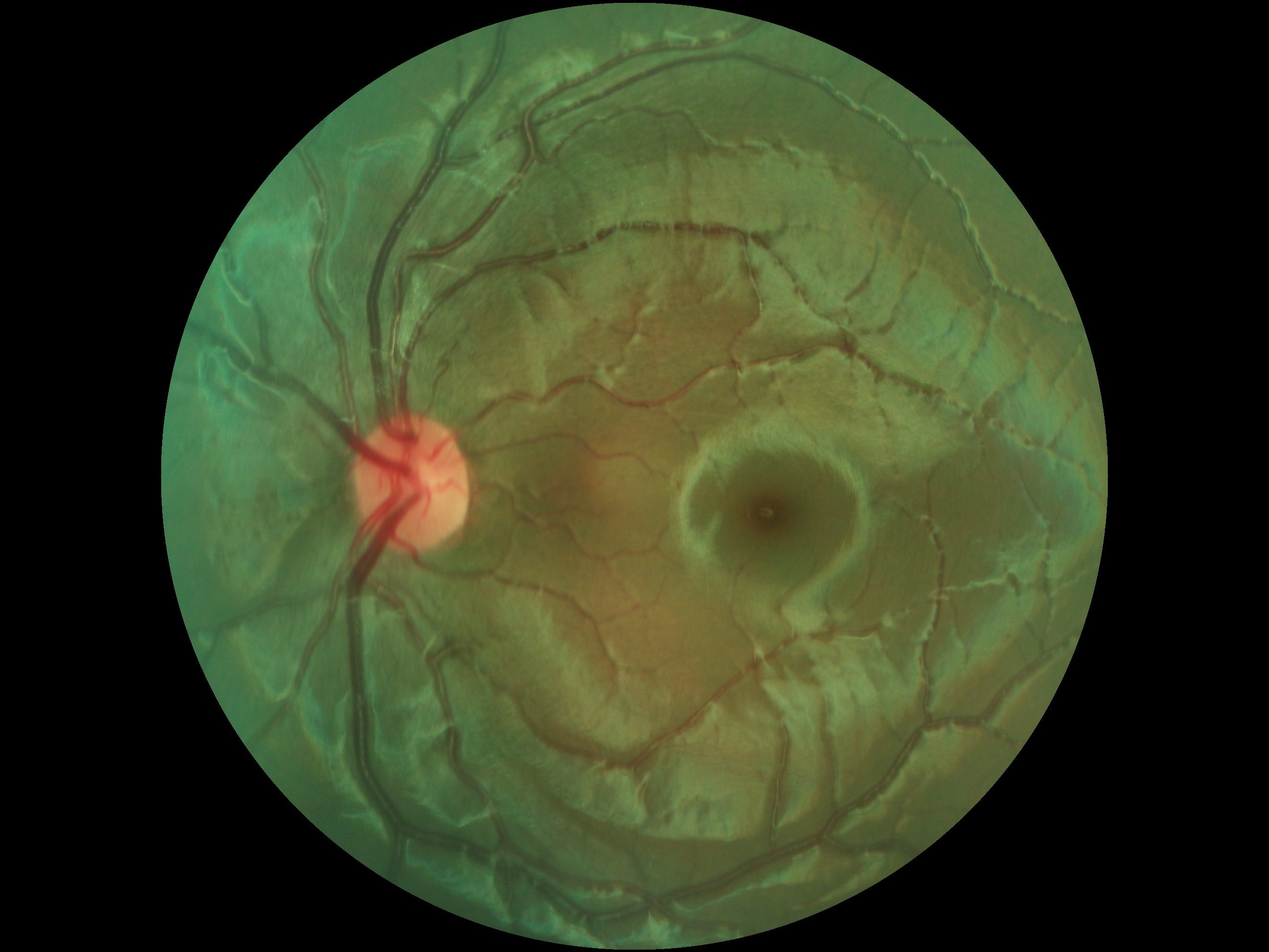

Supplement: S4 File — (ZIP) [file pone.0324352.s004.zip › Original fundus photographs (2)/Subject 78/OS_20230611822049_20230613103930_2.jpg]

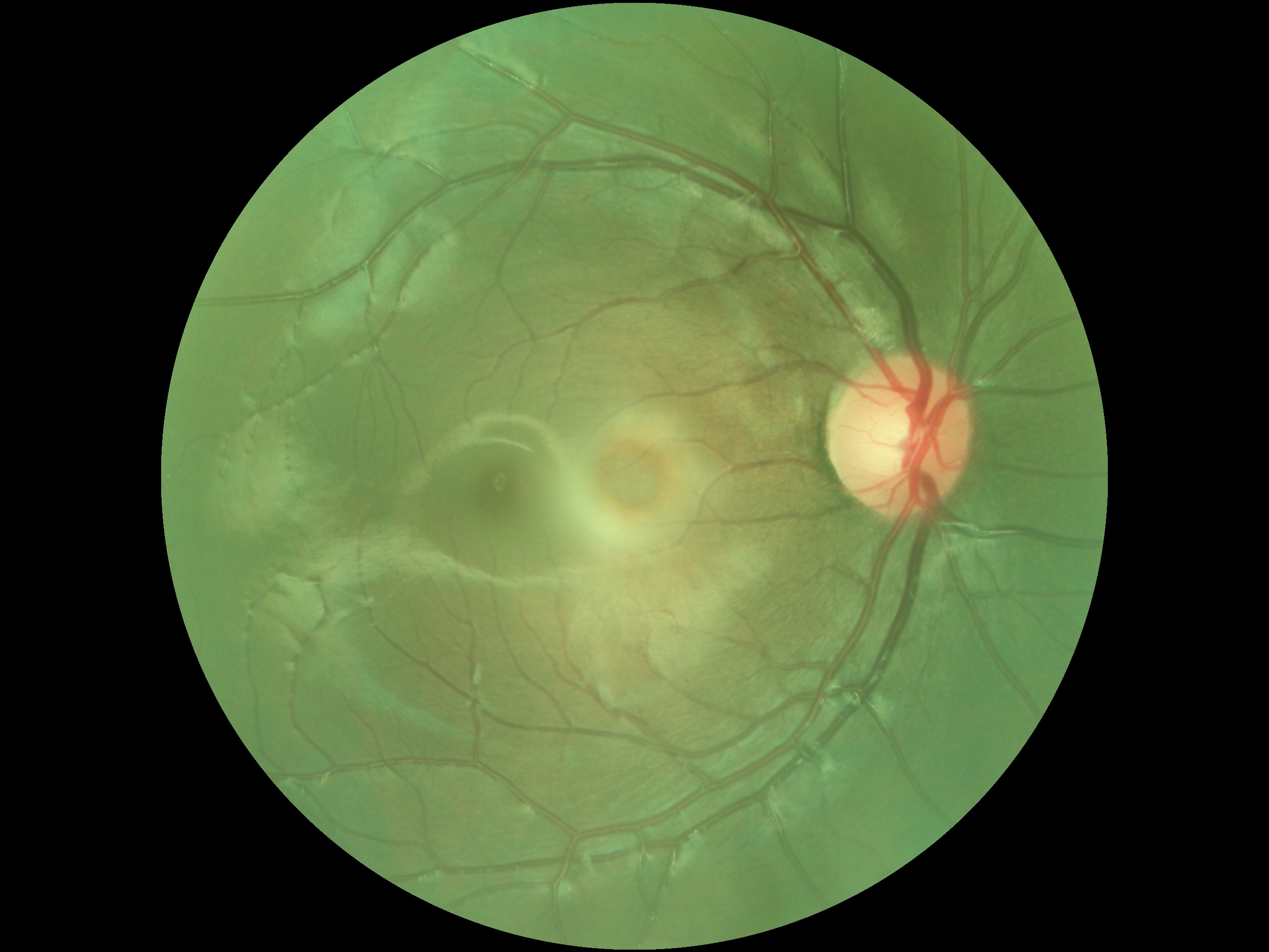

Supplement: S4 File — (ZIP) [file pone.0324352.s004.zip › Original fundus photographs (2)/Subject 79/OD_20230611289232_20230614104425_1.jpg]

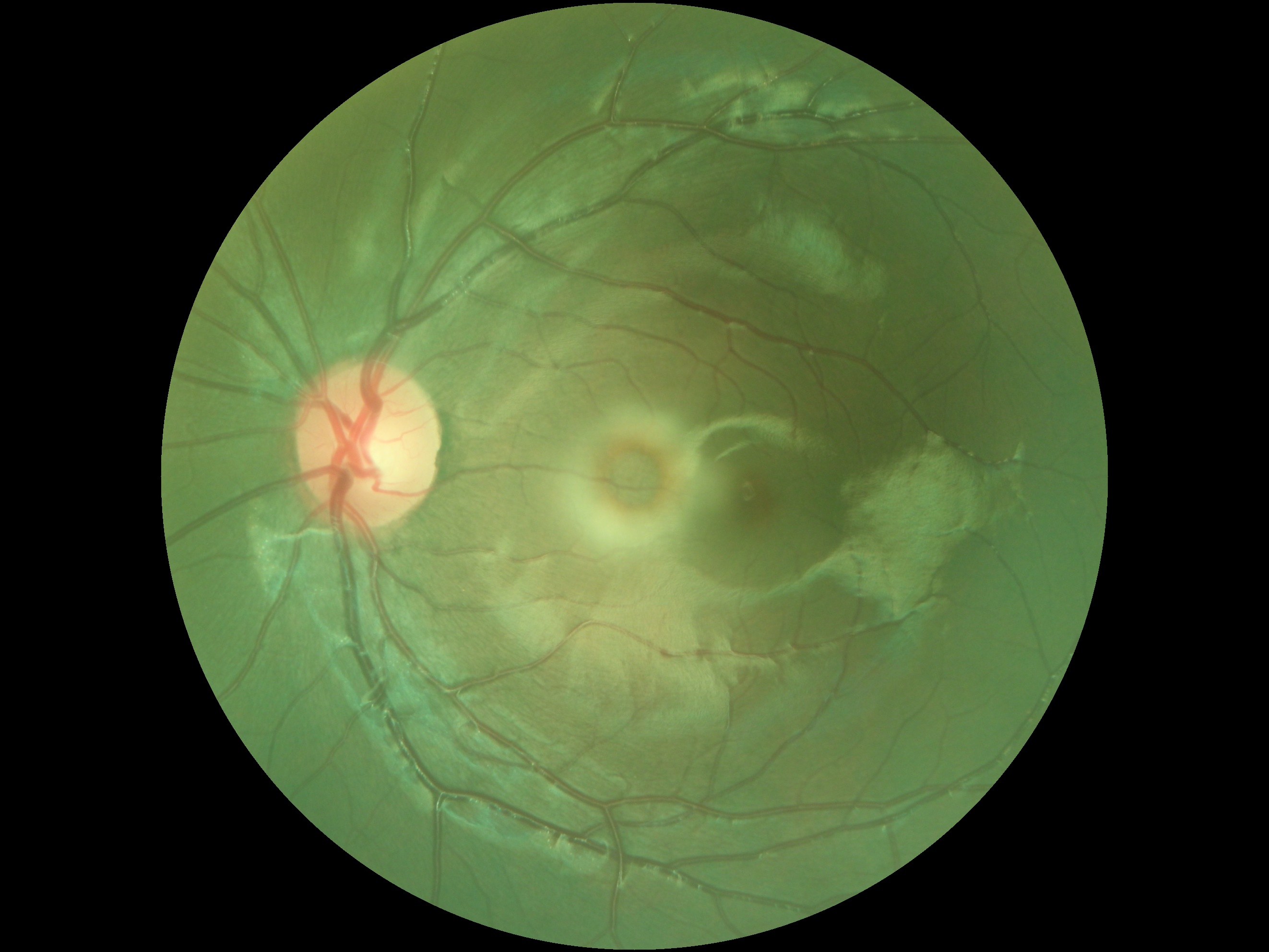

Supplement: S4 File — (ZIP) [file pone.0324352.s004.zip › Original fundus photographs (2)/Subject 79/OS_20230611289232_20230614104443_2.jpg]

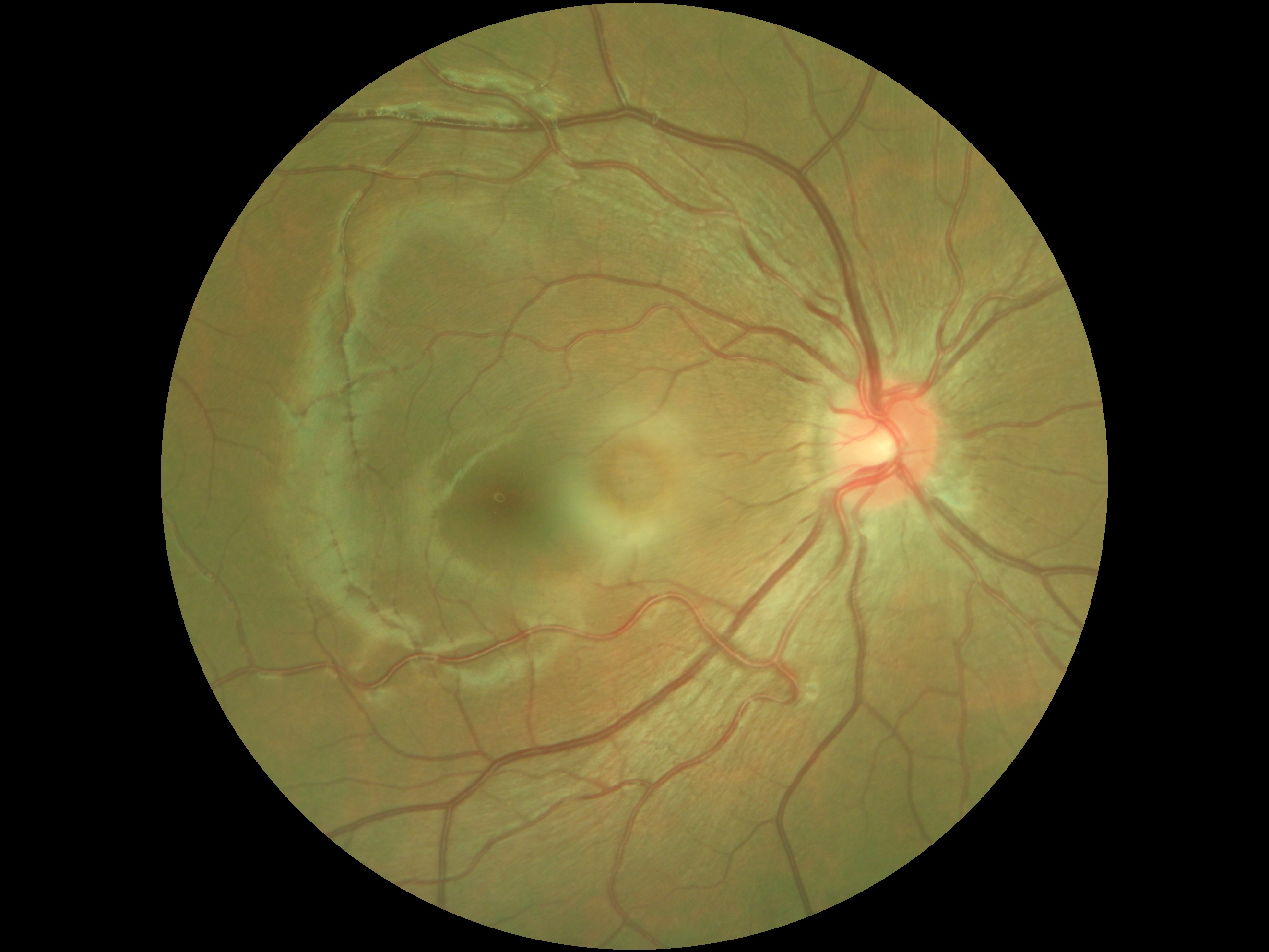

Supplement: S4 File — (ZIP) [file pone.0324352.s004.zip › Original fundus photographs (2)/Subject 80/OD_20230611265193_20230615113933_1.jpg]

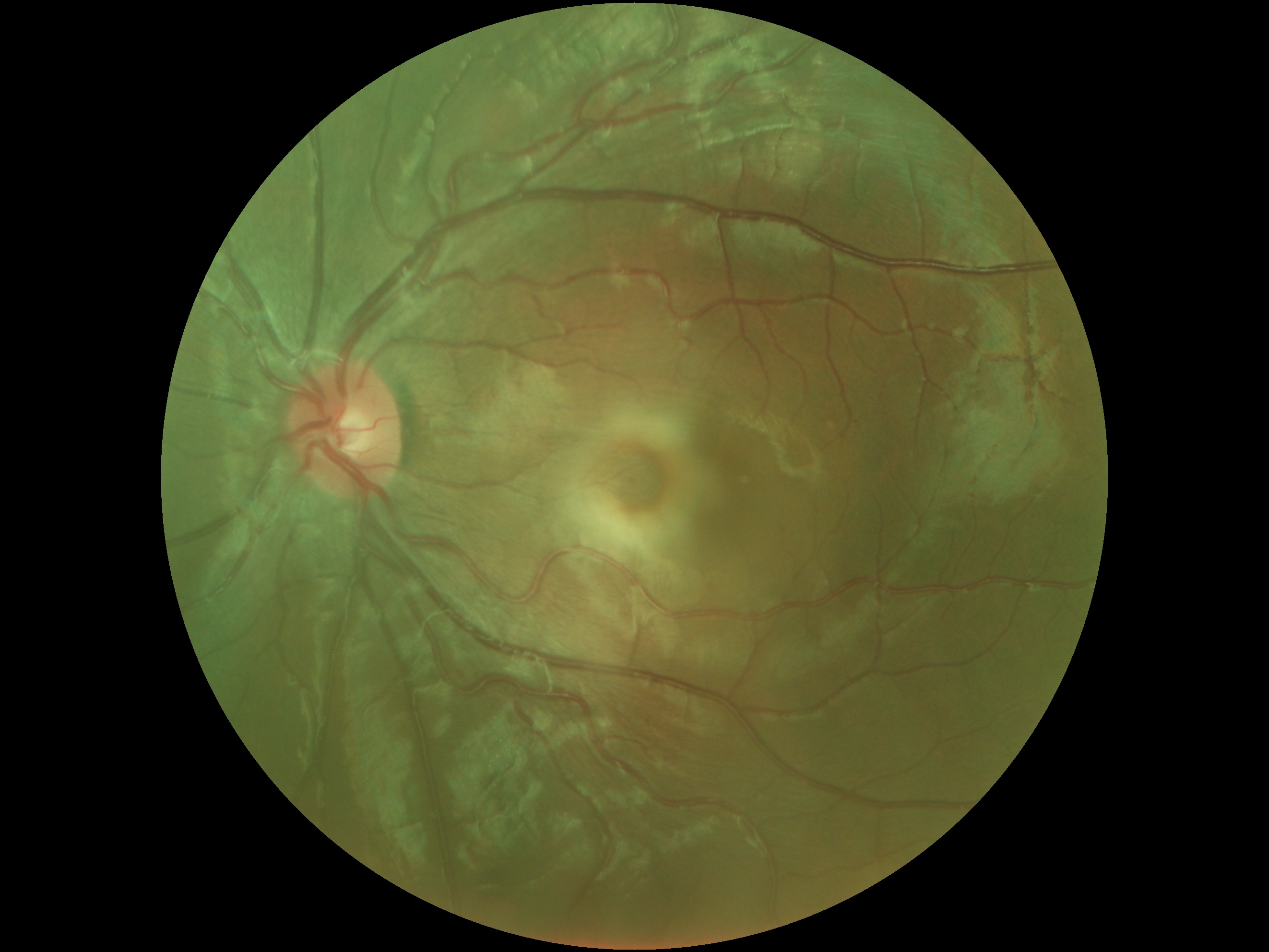

Supplement: S4 File — (ZIP) [file pone.0324352.s004.zip › Original fundus photographs (2)/Subject 80/OS_20230611265193_20230615113945_2.jpg]

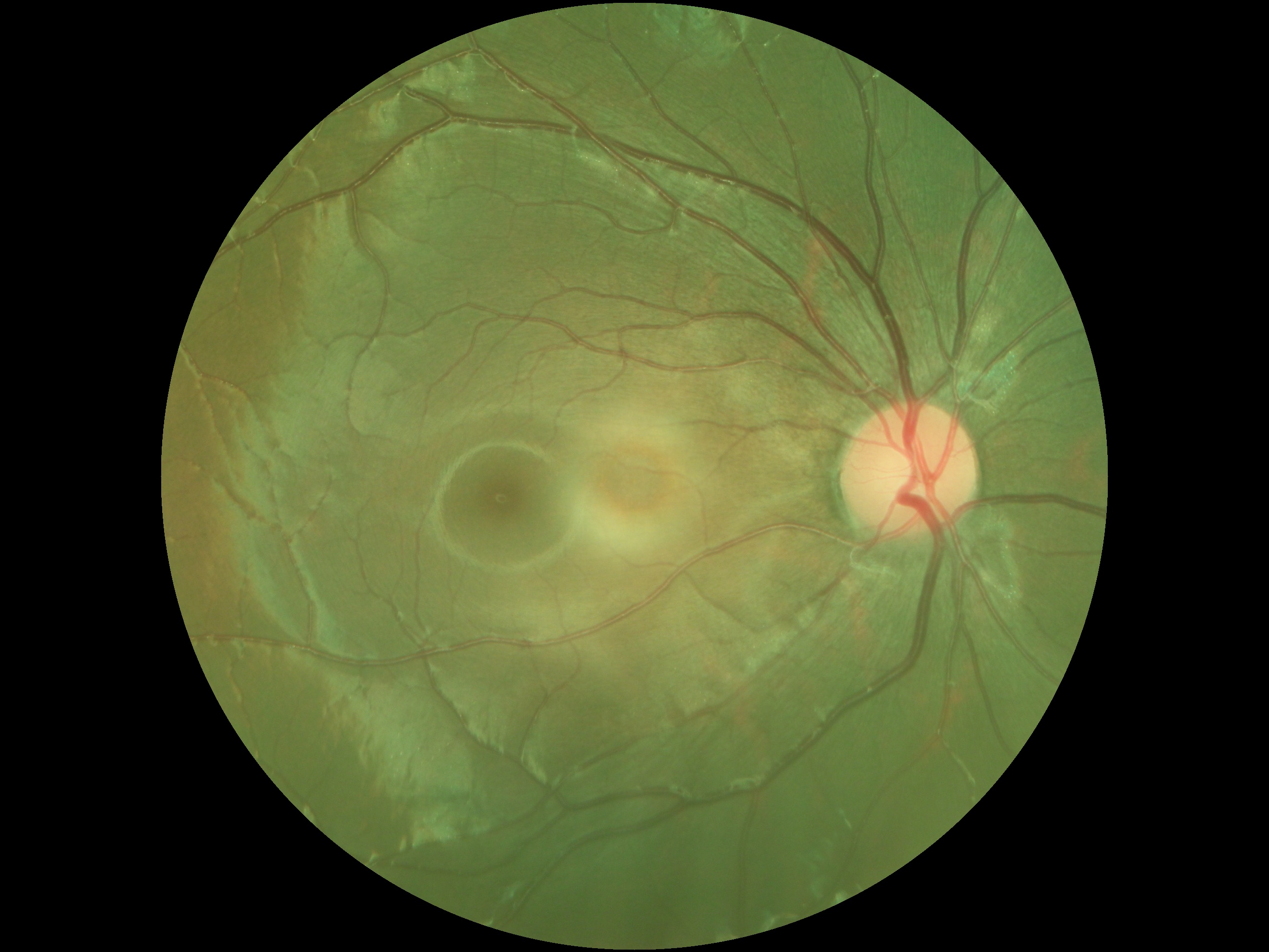

Supplement: S4 File — (ZIP) [file pone.0324352.s004.zip › Original fundus photographs (2)/Subject 81/OD_20230615122030_20230615112113_1.jpg]

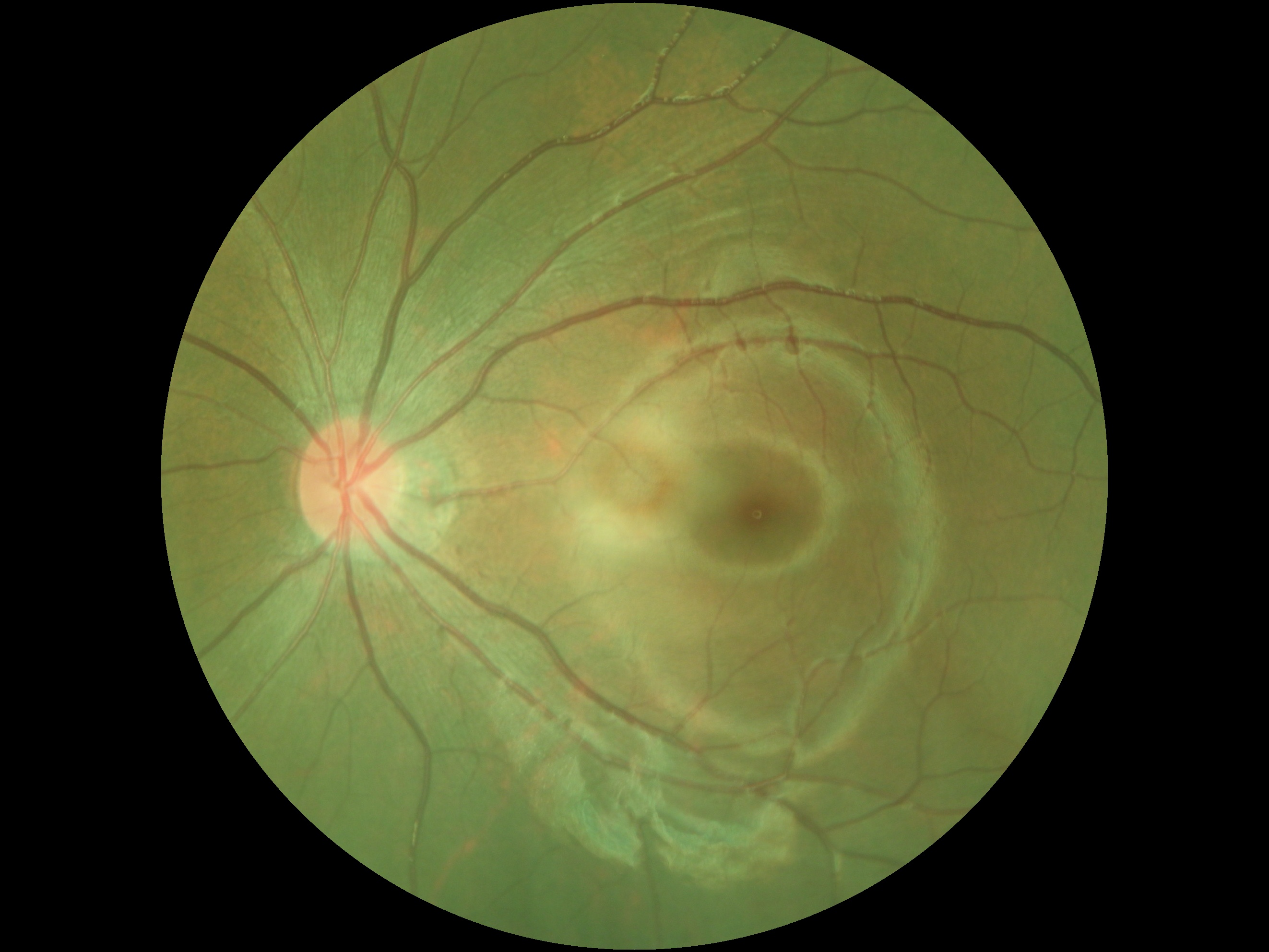

Supplement: S4 File — (ZIP) [file pone.0324352.s004.zip › Original fundus photographs (2)/Subject 81/OS_20230615122030_20230615112129_2.jpg]

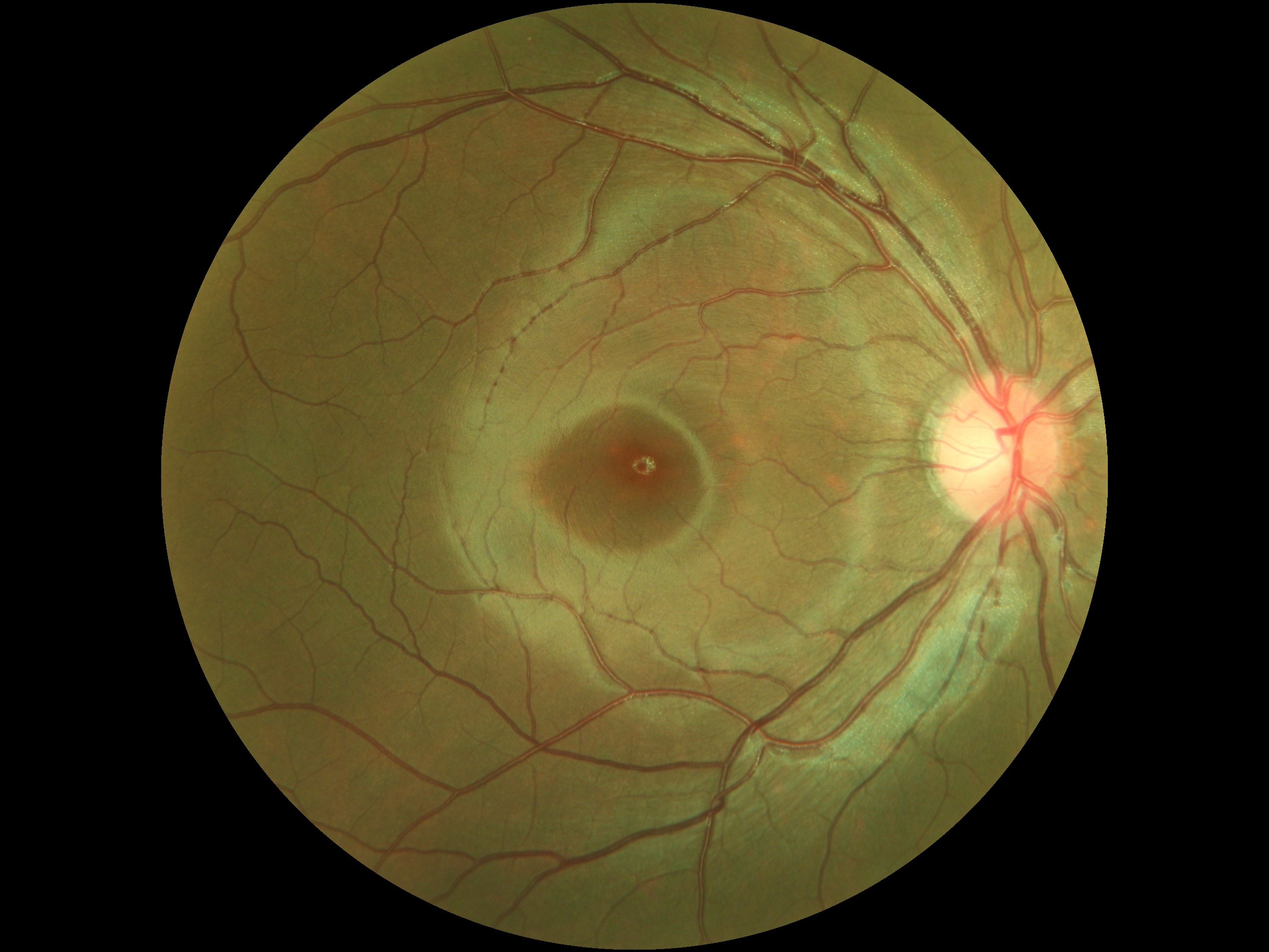

Supplement: S4 File — (ZIP) [file pone.0324352.s004.zip › Original fundus photographs (2)/Subject 82/OD_20230611952047_20230613104917_1.jpg]

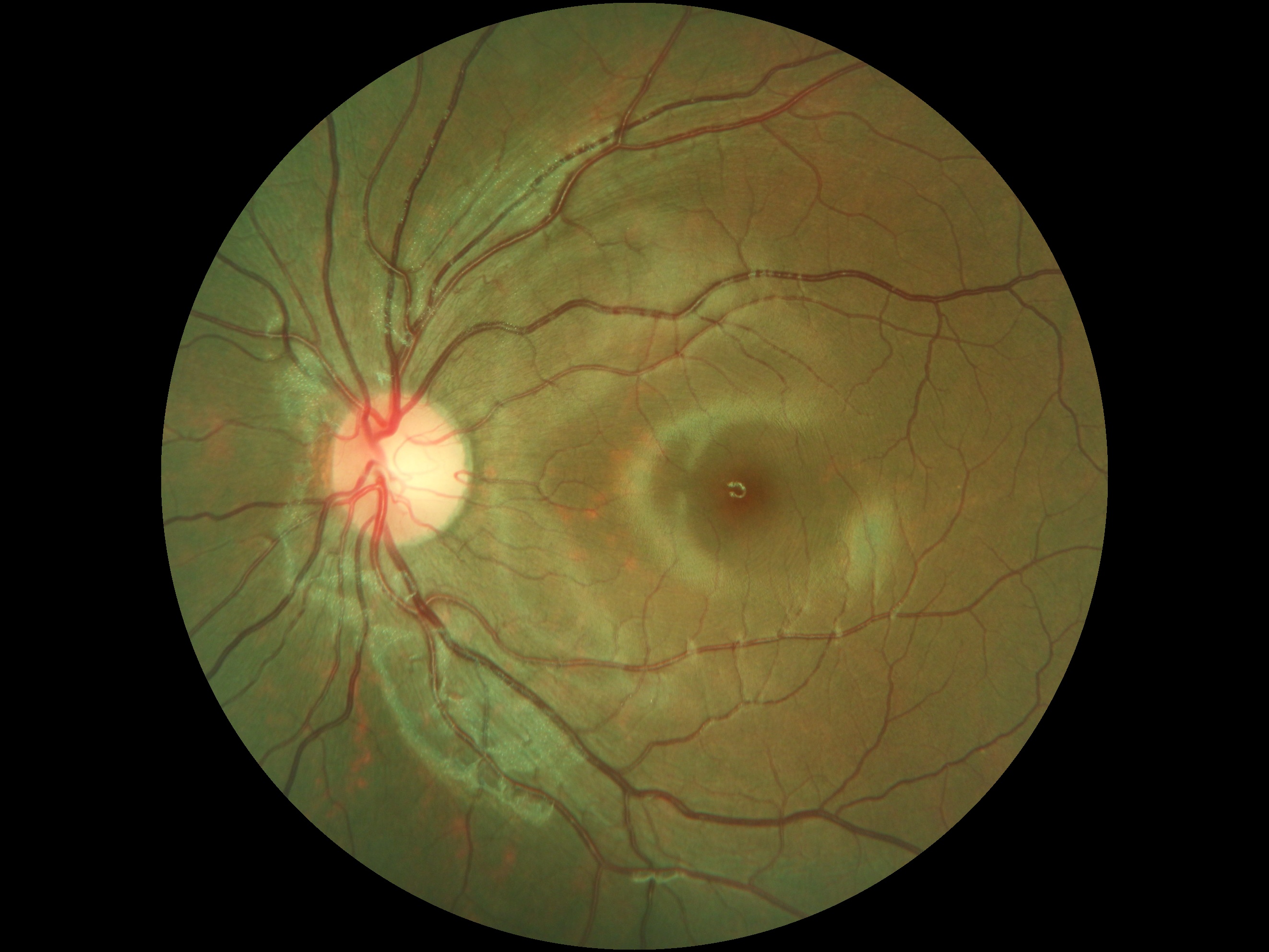

Supplement: S4 File — (ZIP) [file pone.0324352.s004.zip › Original fundus photographs (2)/Subject 82/OS_20230611952047_20230613104938_2.jpg]

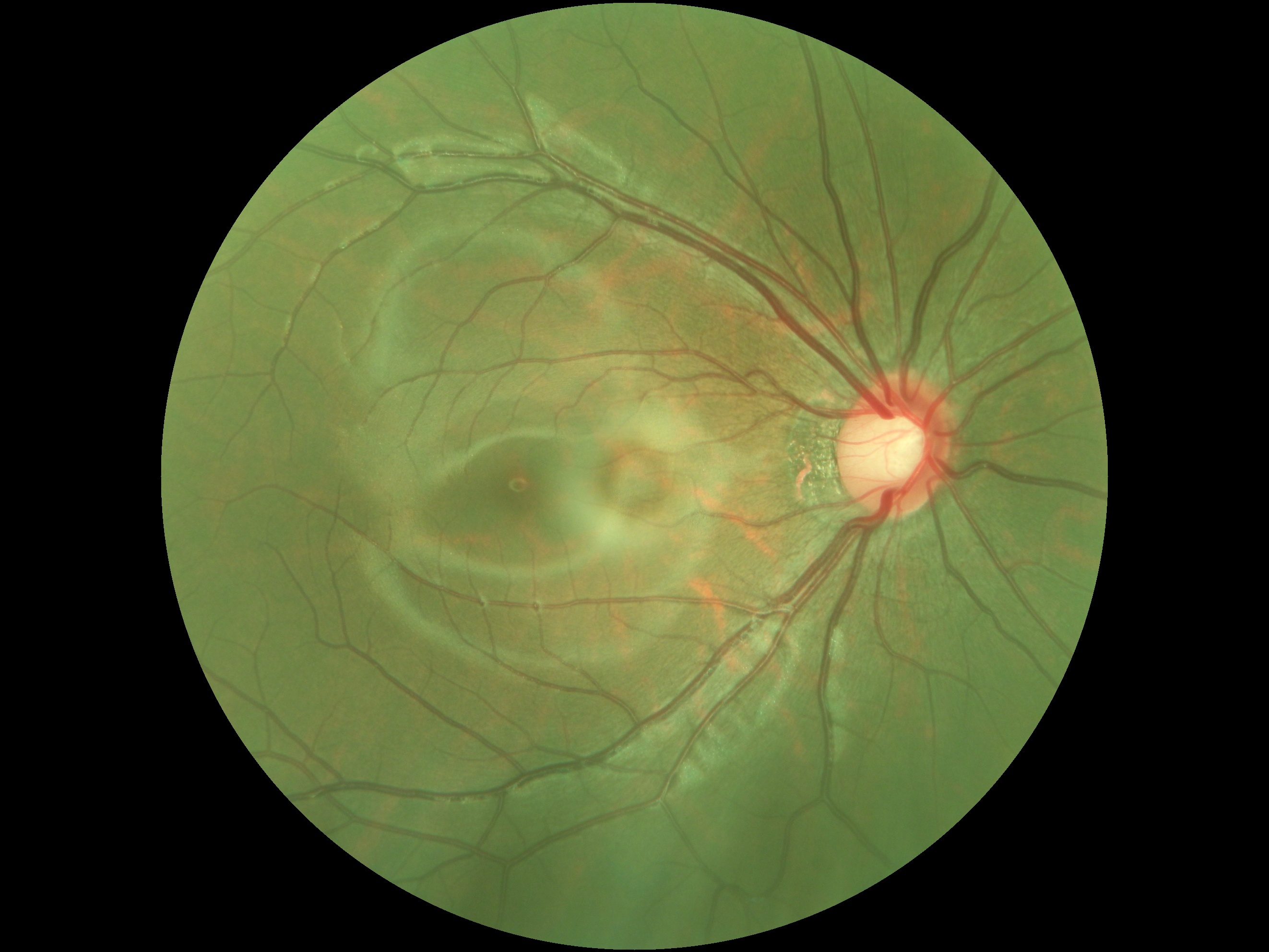

Supplement: S4 File — (ZIP) [file pone.0324352.s004.zip › Original fundus photographs (2)/Subject 83/OD_20230611050086_20230615152401_1.jpg]

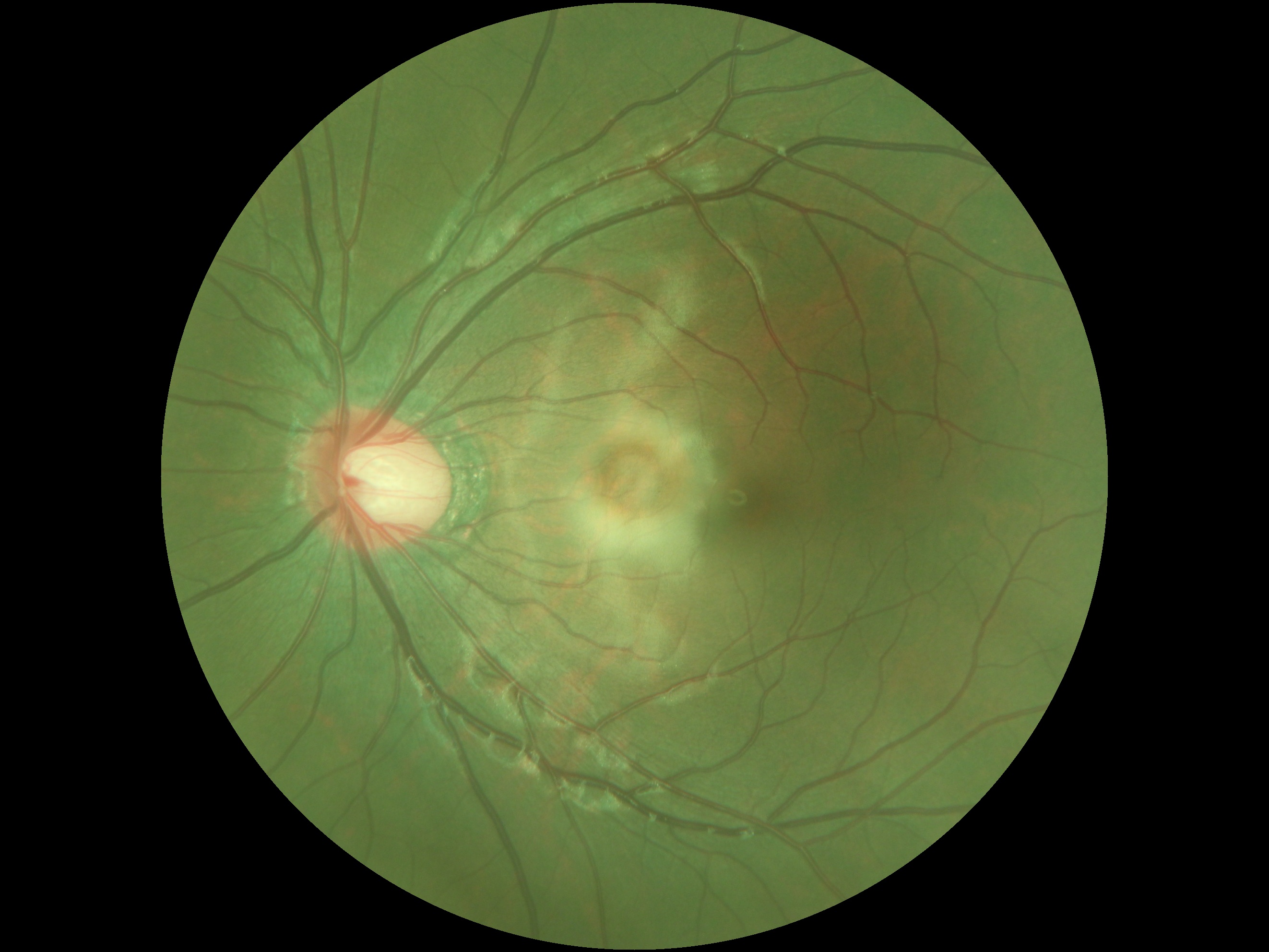

Supplement: S4 File — (ZIP) [file pone.0324352.s004.zip › Original fundus photographs (2)/Subject 83/OS_20230611050086_20230615152413_2.jpg]

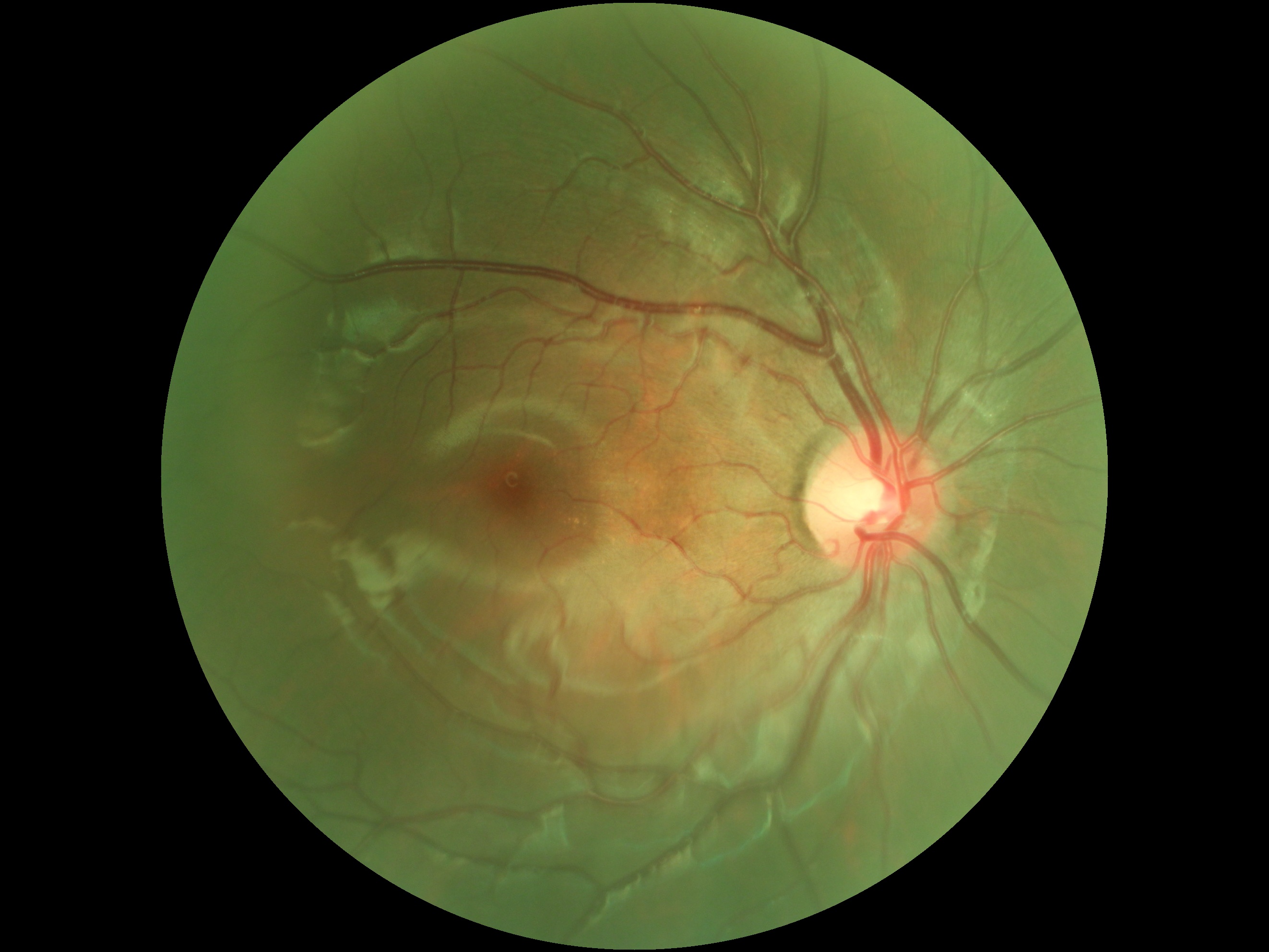

Supplement: S4 File — (ZIP) [file pone.0324352.s004.zip › Original fundus photographs (2)/Subject 84/OD_20230611447096_20230612160927_1.jpg]

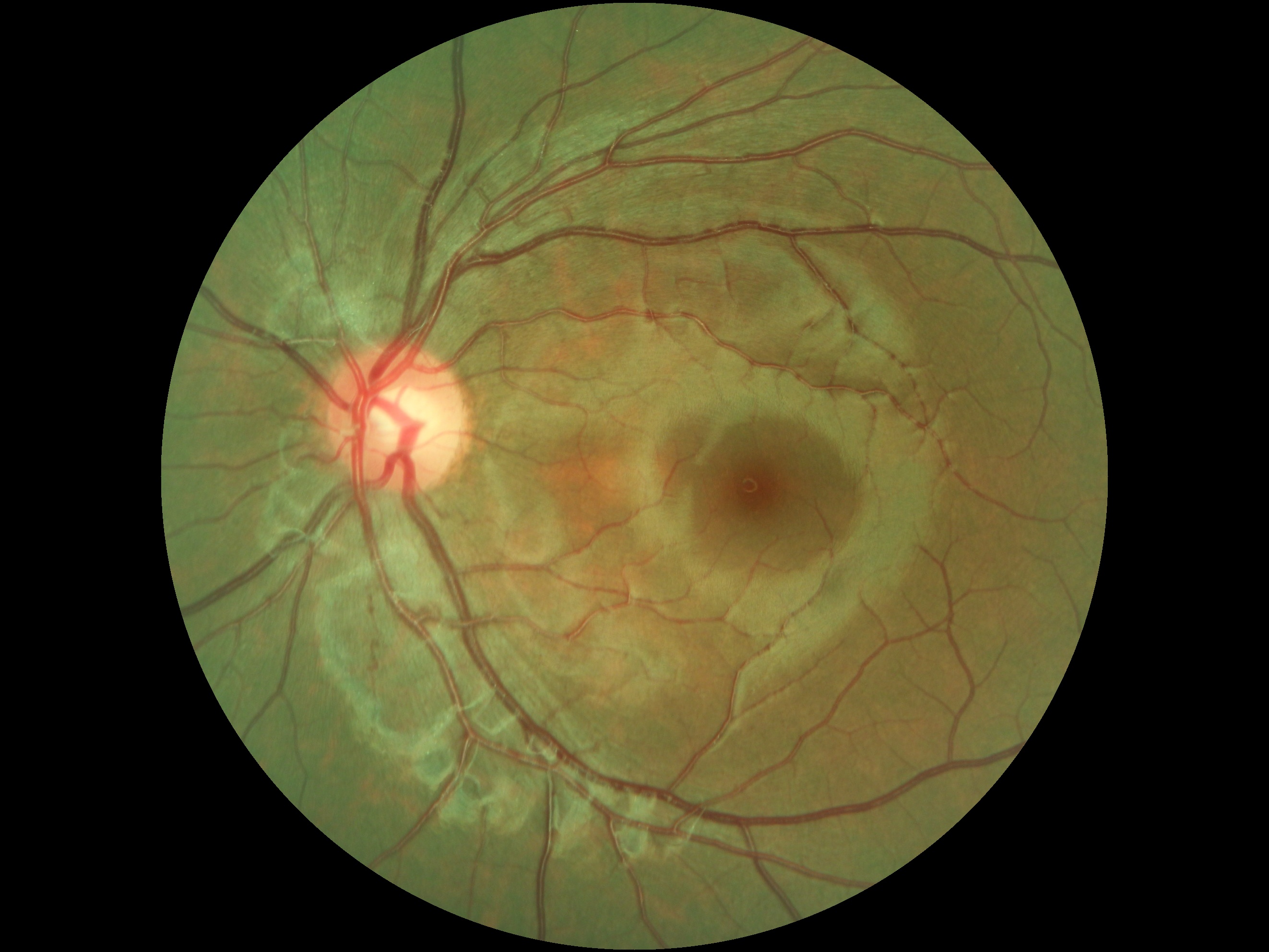

Supplement: S4 File — (ZIP) [file pone.0324352.s004.zip › Original fundus photographs (2)/Subject 84/OS_20230611447096_20230612161006_2.jpg]

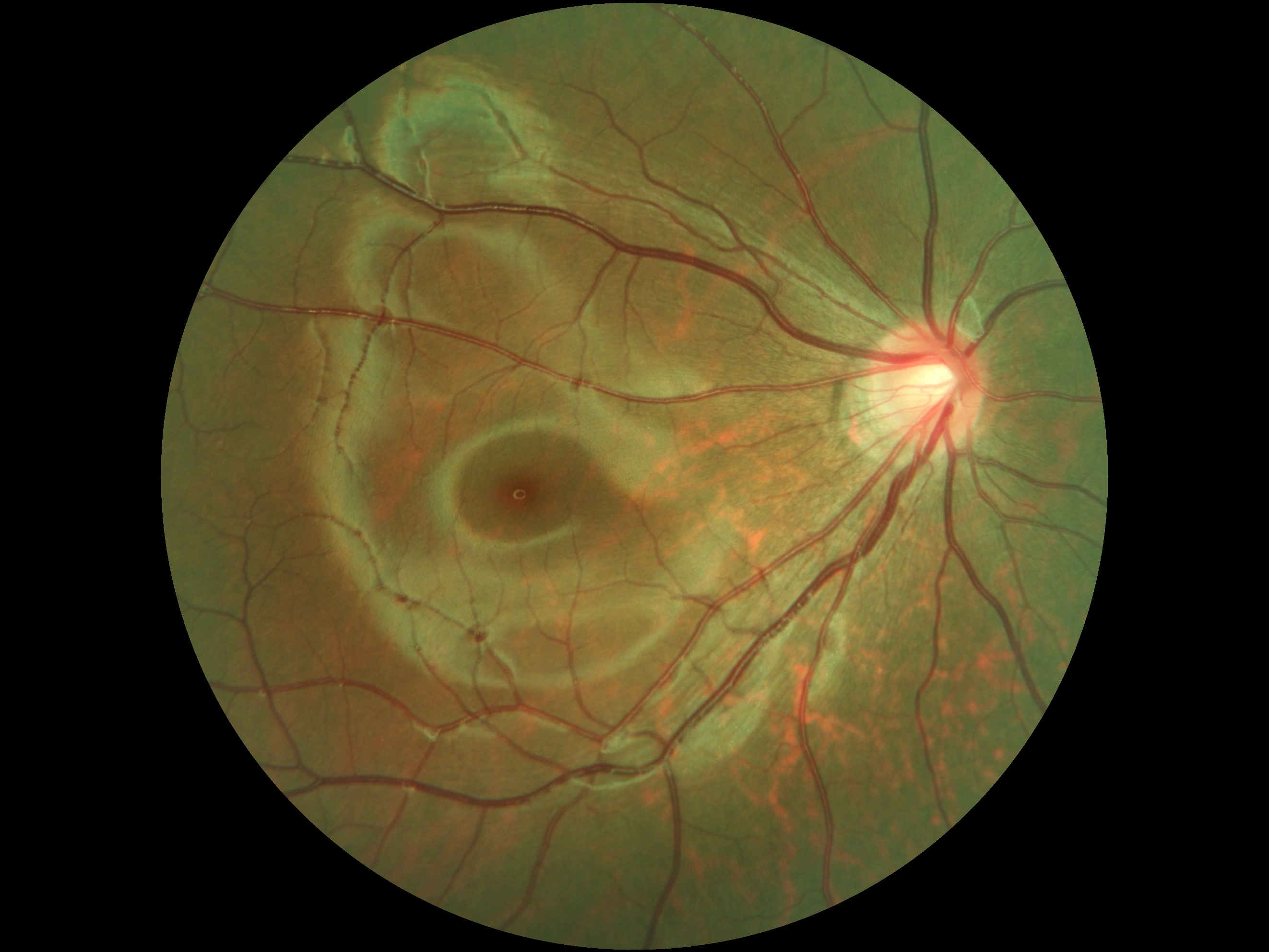

Supplement: S4 File — (ZIP) [file pone.0324352.s004.zip › Original fundus photographs (2)/Subject 85/OD_20230611015097_20230612160623_1.jpg]

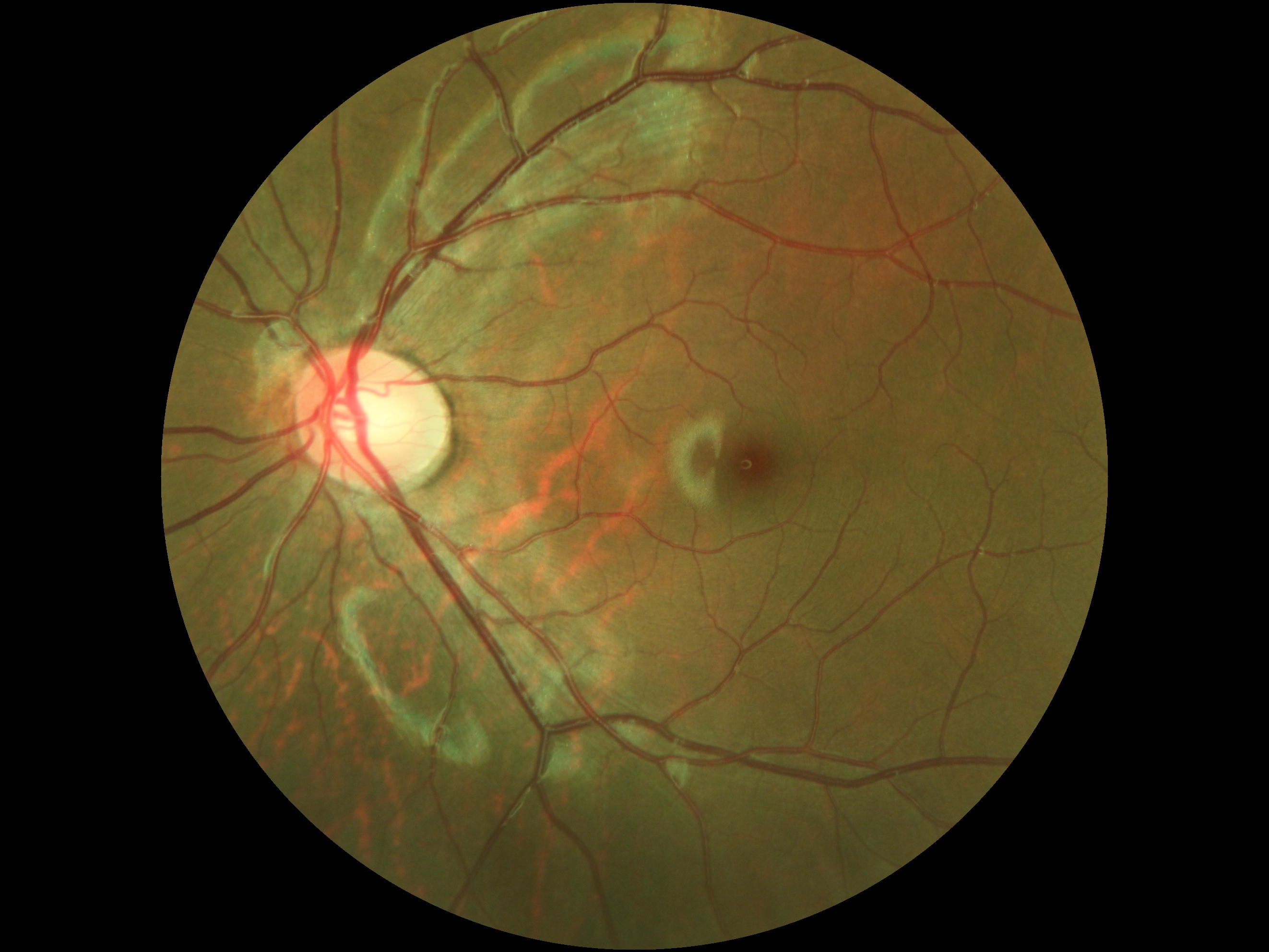

Supplement: S4 File — (ZIP) [file pone.0324352.s004.zip › Original fundus photographs (2)/Subject 85/OS_20230611015097_20230612160703_2.jpg]

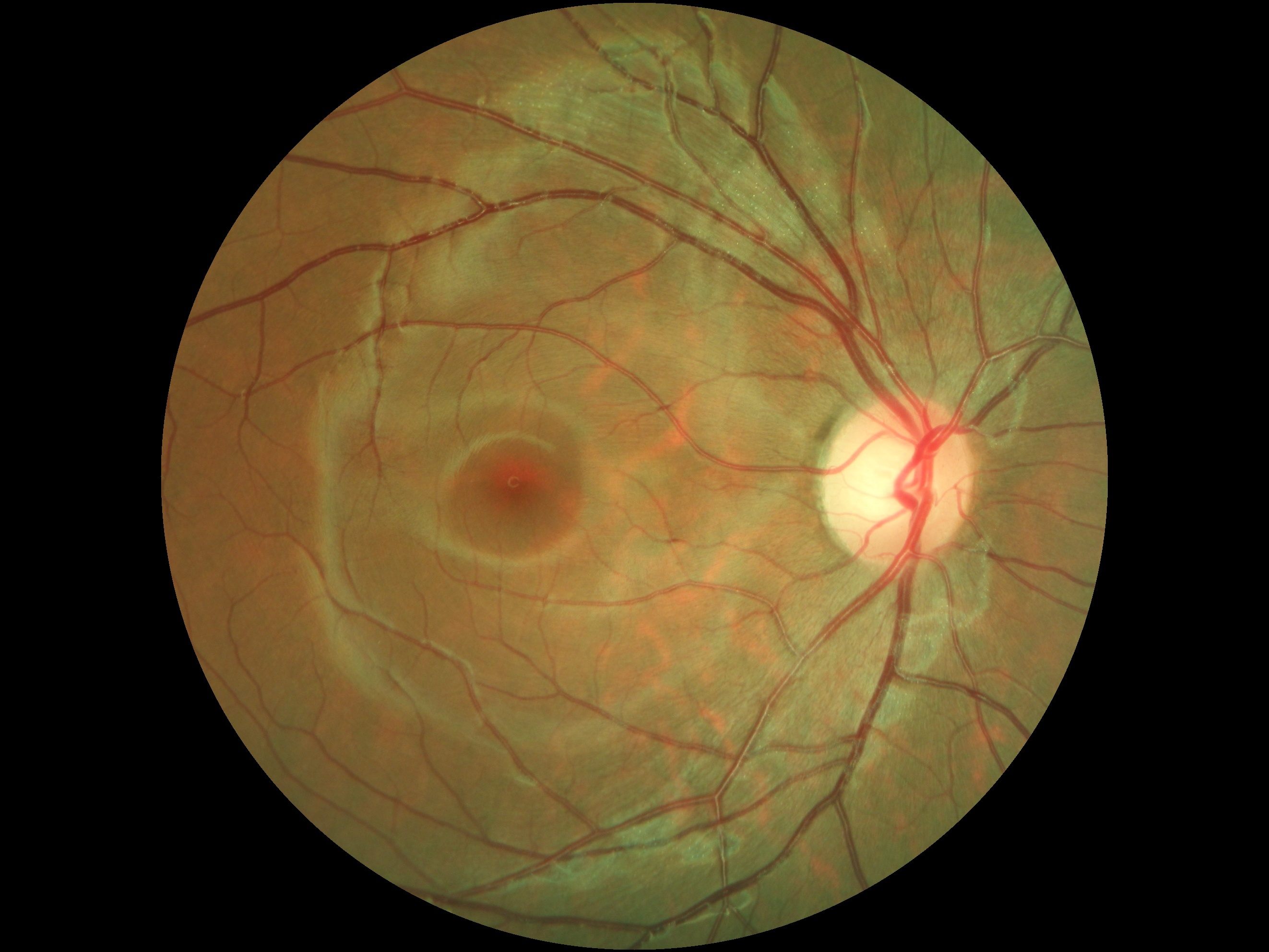

Supplement: S4 File — (ZIP) [file pone.0324352.s004.zip › Original fundus photographs (2)/Subject 86/OD_20230611893056_20230612164330_1.jpg]

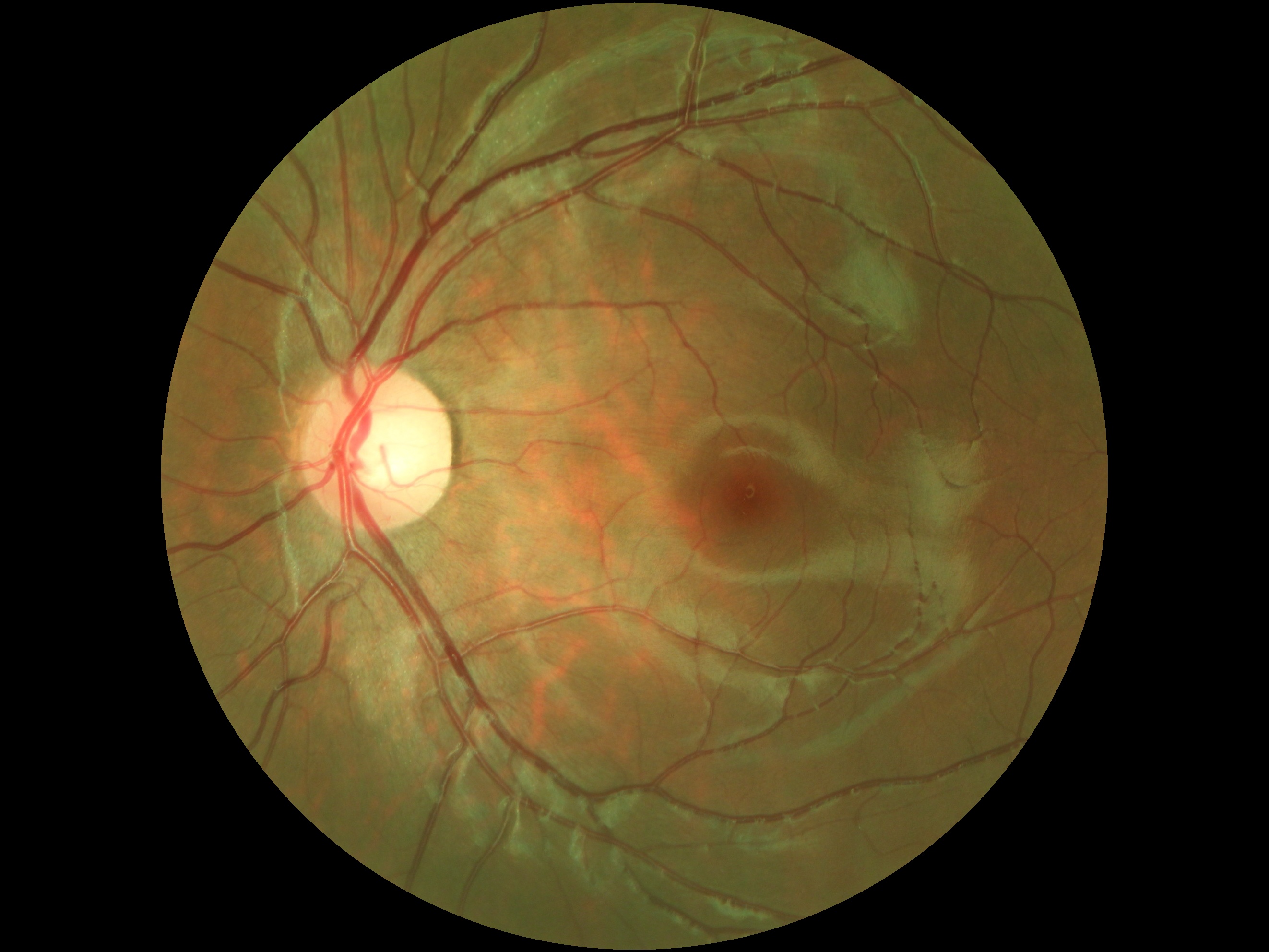

Supplement: S4 File — (ZIP) [file pone.0324352.s004.zip › Original fundus photographs (2)/Subject 86/OS_20230611893056_20230612164351_2.jpg]

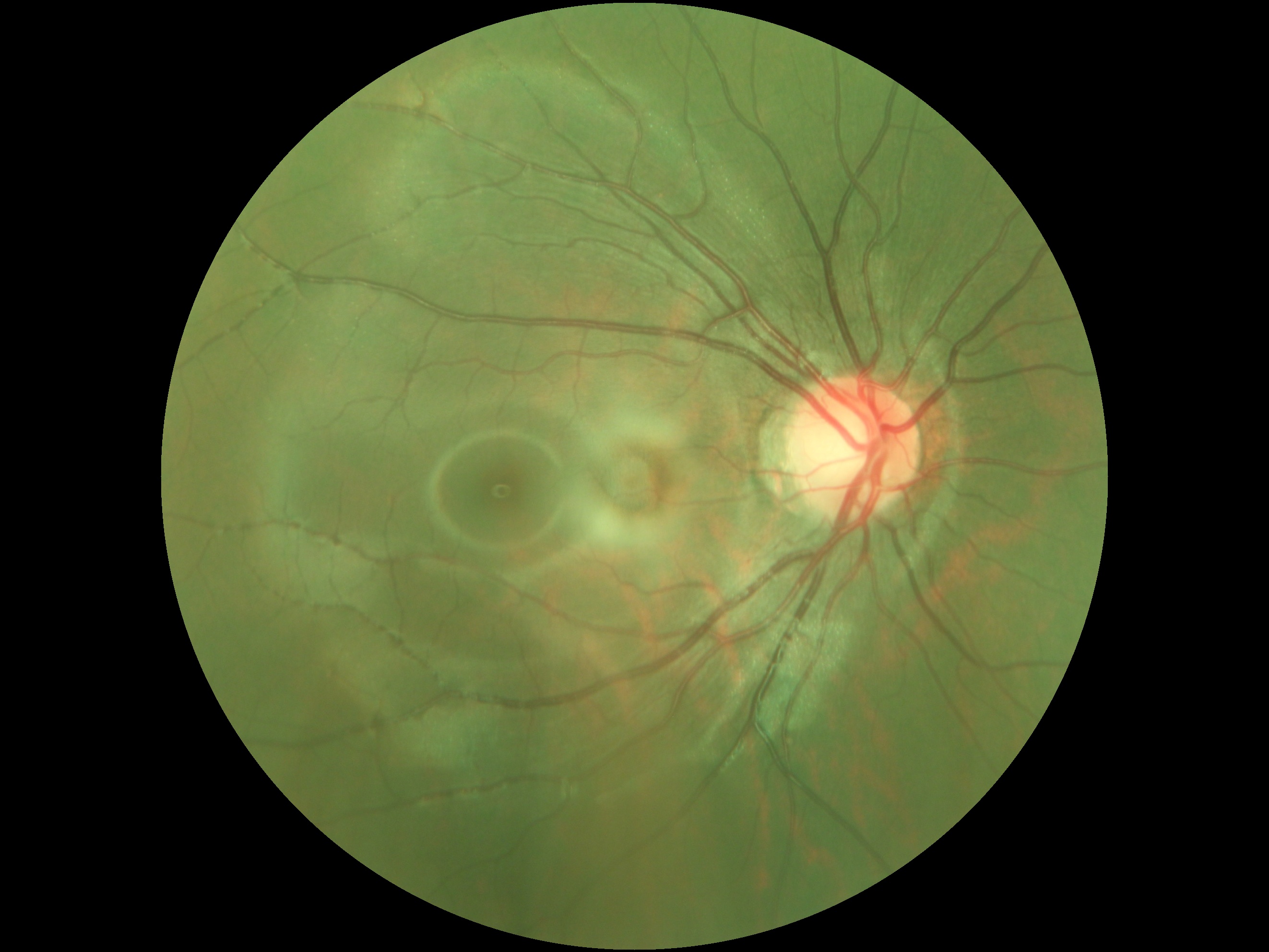

Supplement: S4 File — (ZIP) [file pone.0324352.s004.zip › Original fundus photographs (2)/Subject 87/OD_20230611852201_20230615112459_1.jpg]

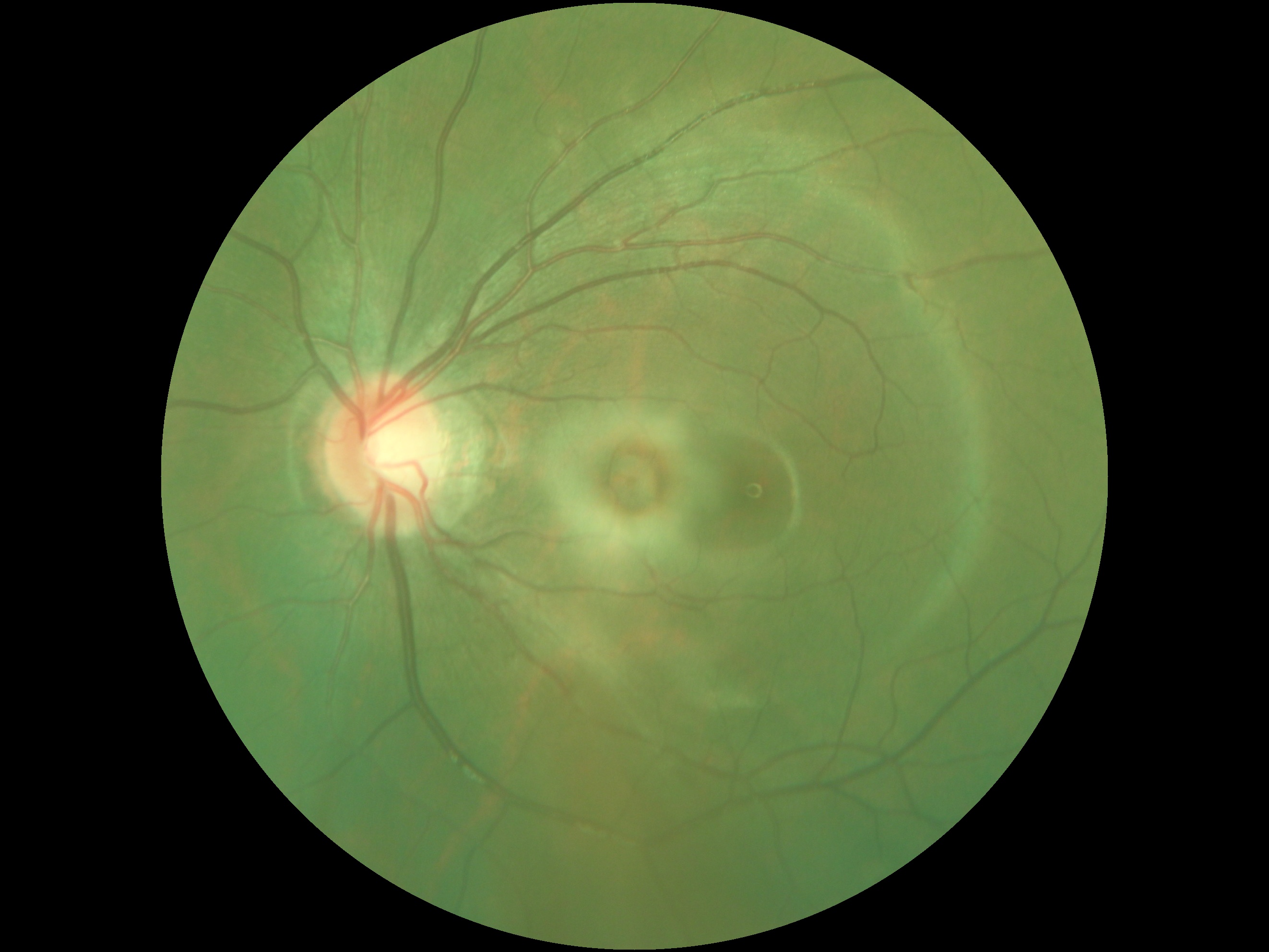

Supplement: S4 File — (ZIP) [file pone.0324352.s004.zip › Original fundus photographs (2)/Subject 87/OS_20230611852201_20230615112655_5.jpg]

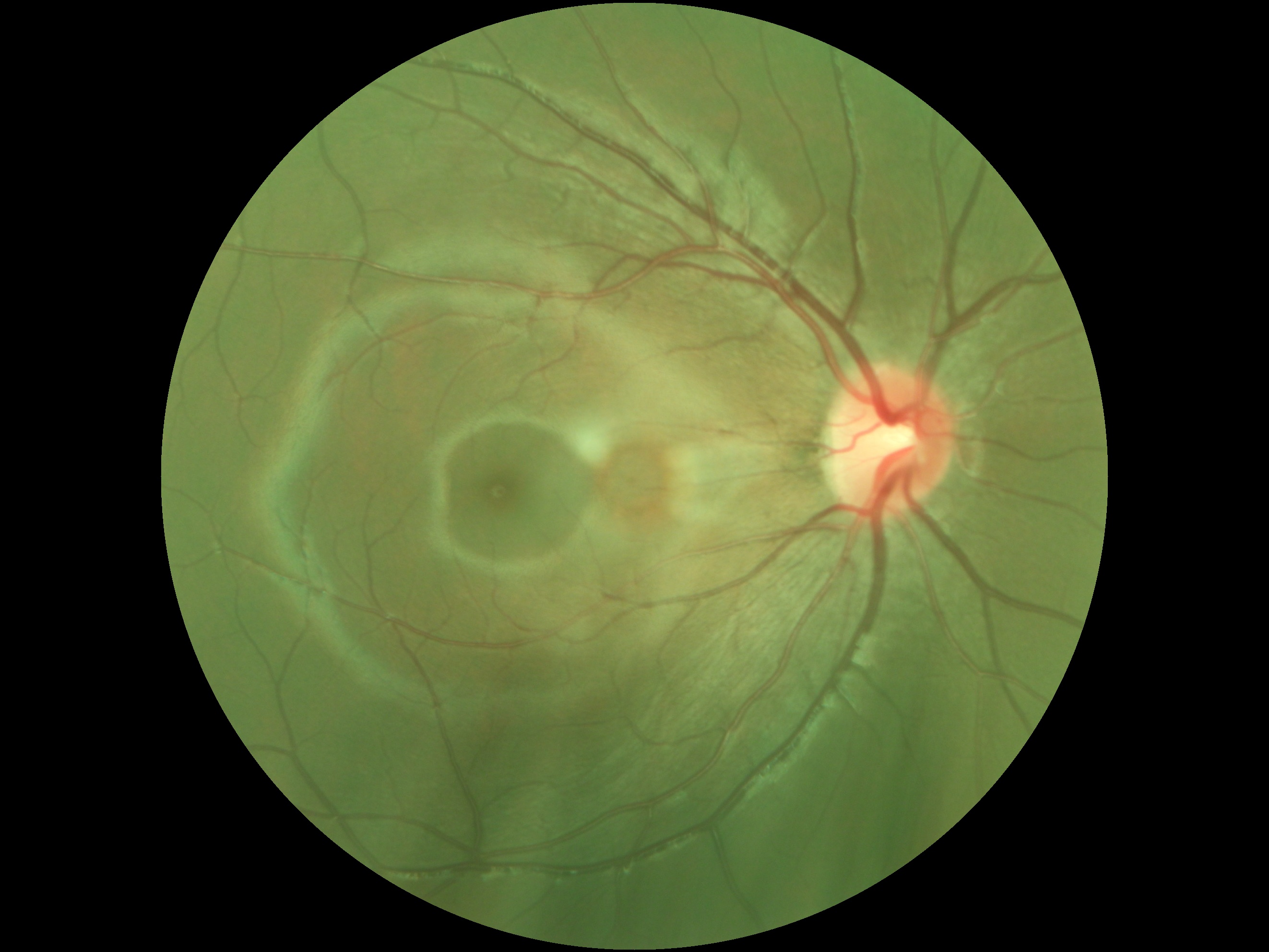

Supplement: S4 File — (ZIP) [file pone.0324352.s004.zip › Original fundus photographs (2)/Subject 88/OD_20230615968098_20230615170425_2.jpg]

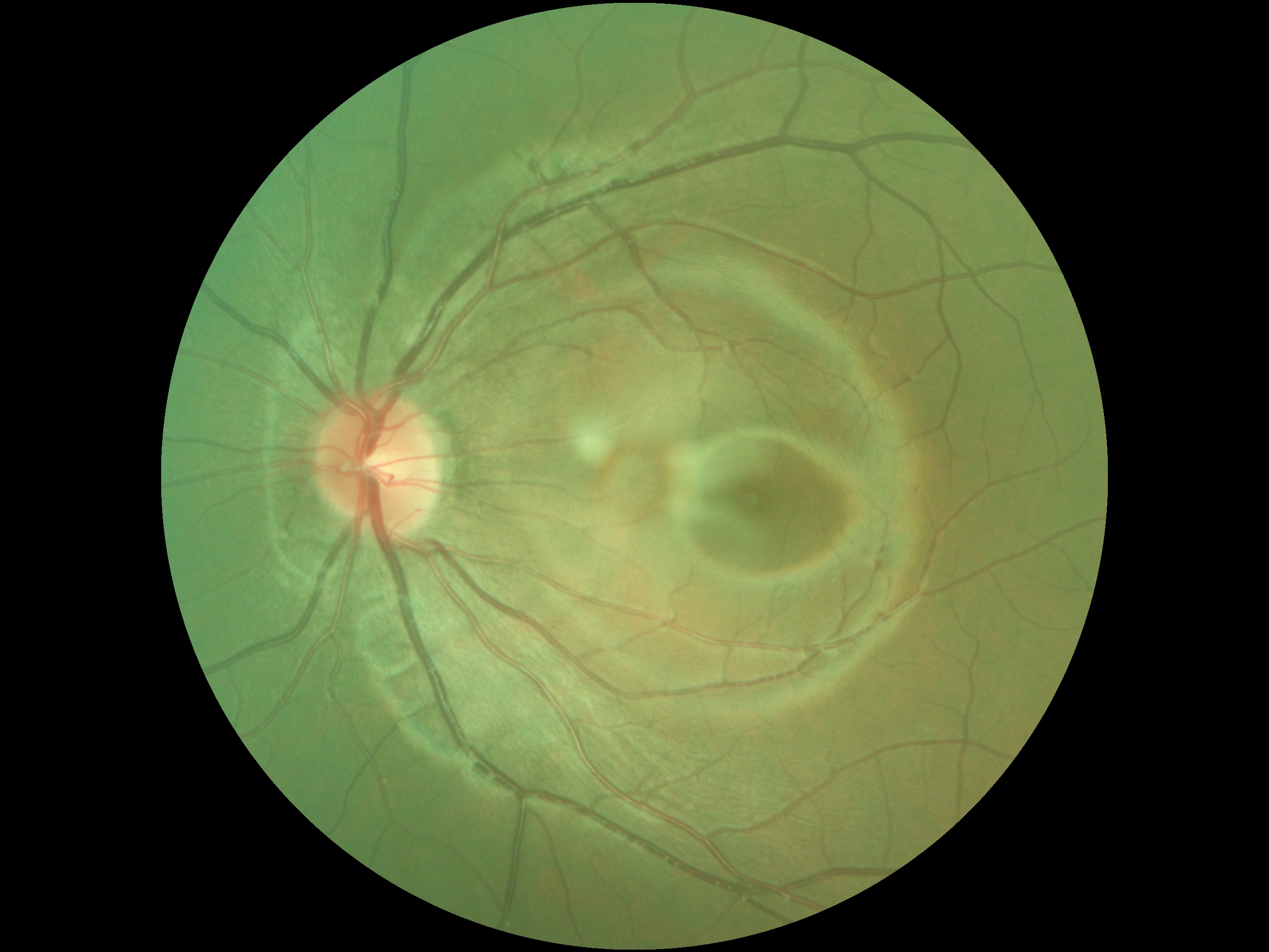

Supplement: S4 File — (ZIP) [file pone.0324352.s004.zip › Original fundus photographs (2)/Subject 88/OS_20230615968098_20230615170348_1.jpg]

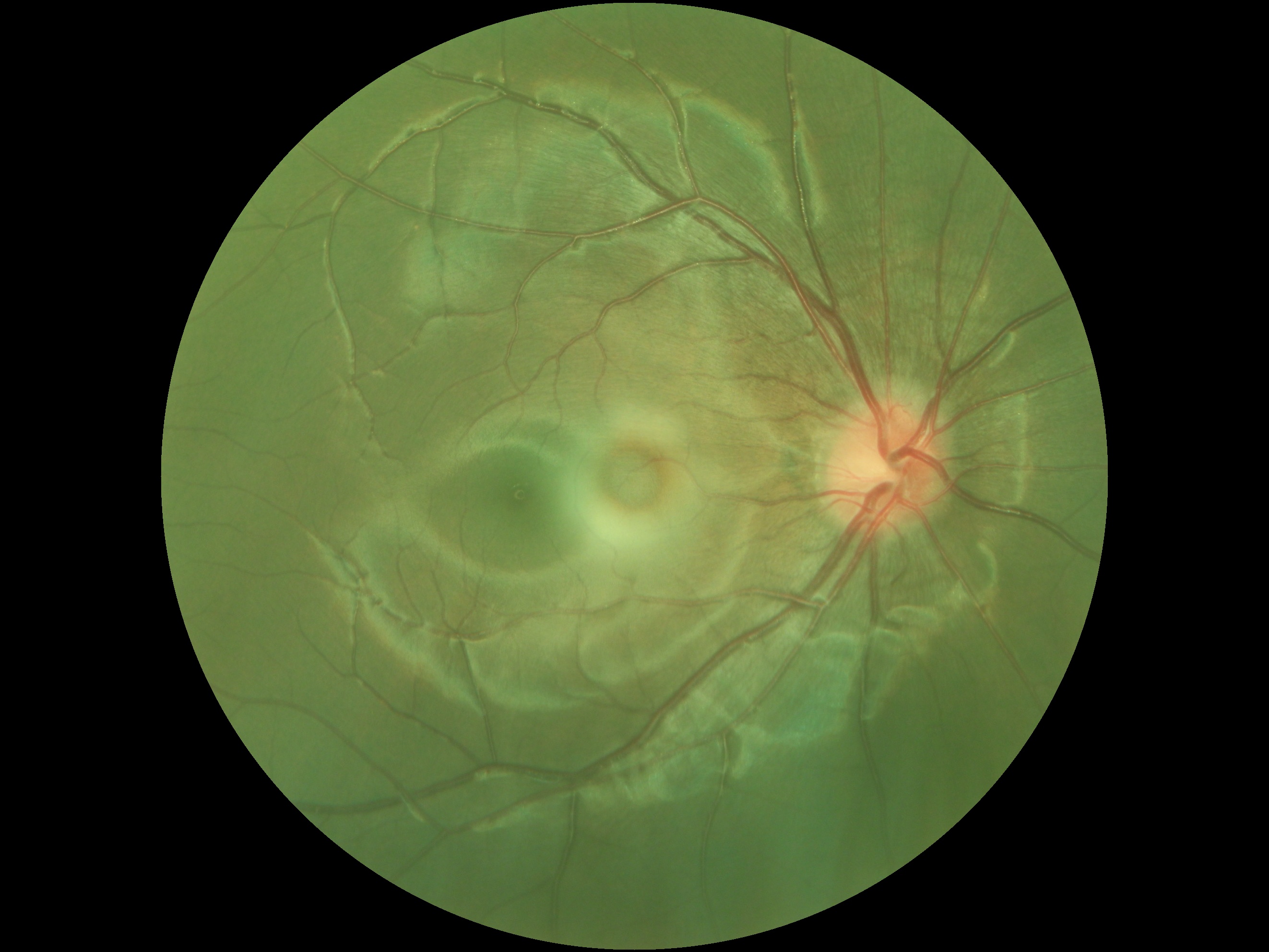

Supplement: S4 File — (ZIP) [file pone.0324352.s004.zip › Original fundus photographs (2)/Subject 89/OD_20230611724124_20230614105802_1.jpg]

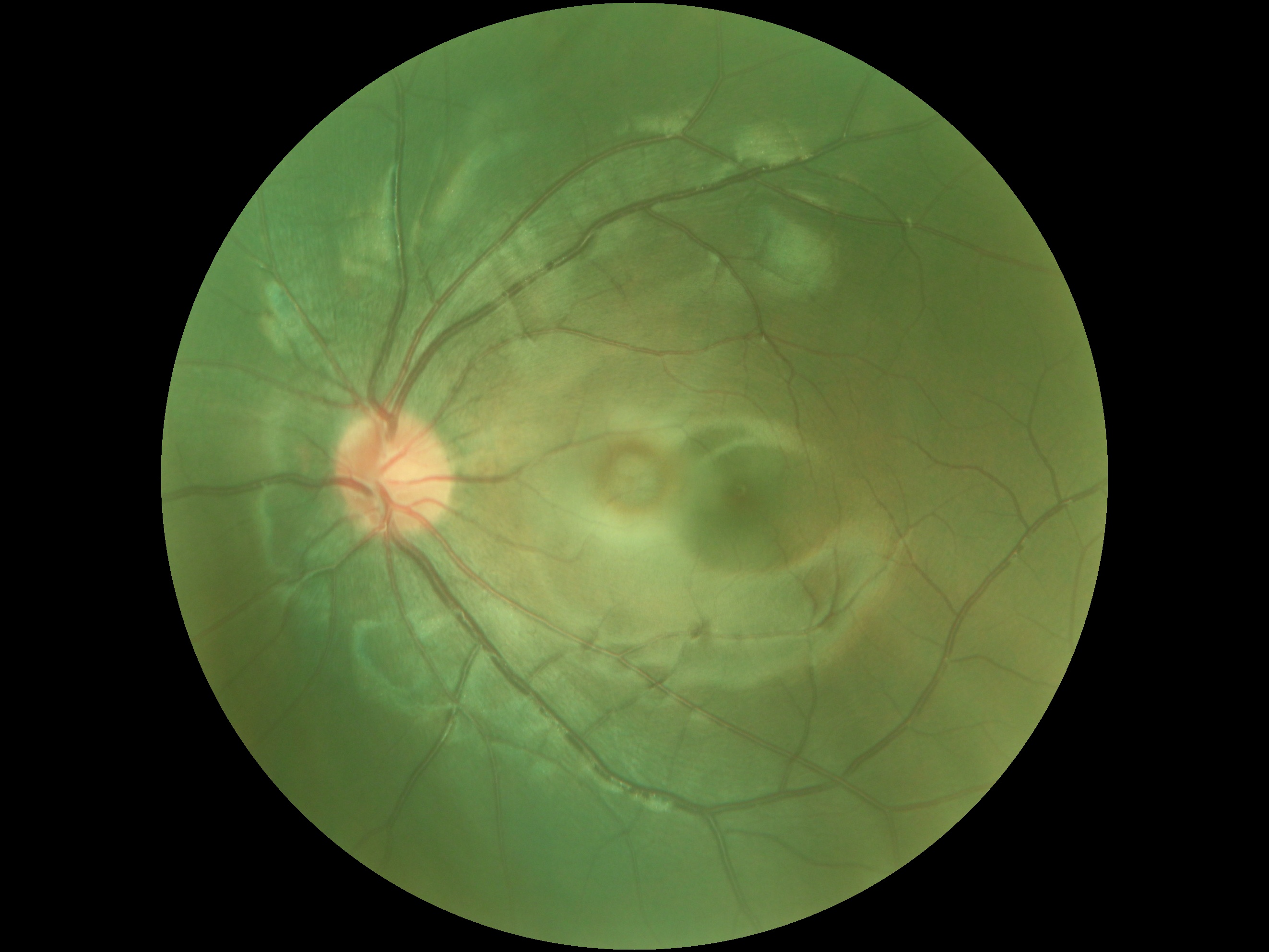

Supplement: S4 File — (ZIP) [file pone.0324352.s004.zip › Original fundus photographs (2)/Subject 89/OS_20230611724124_20230614105937_2.jpg]

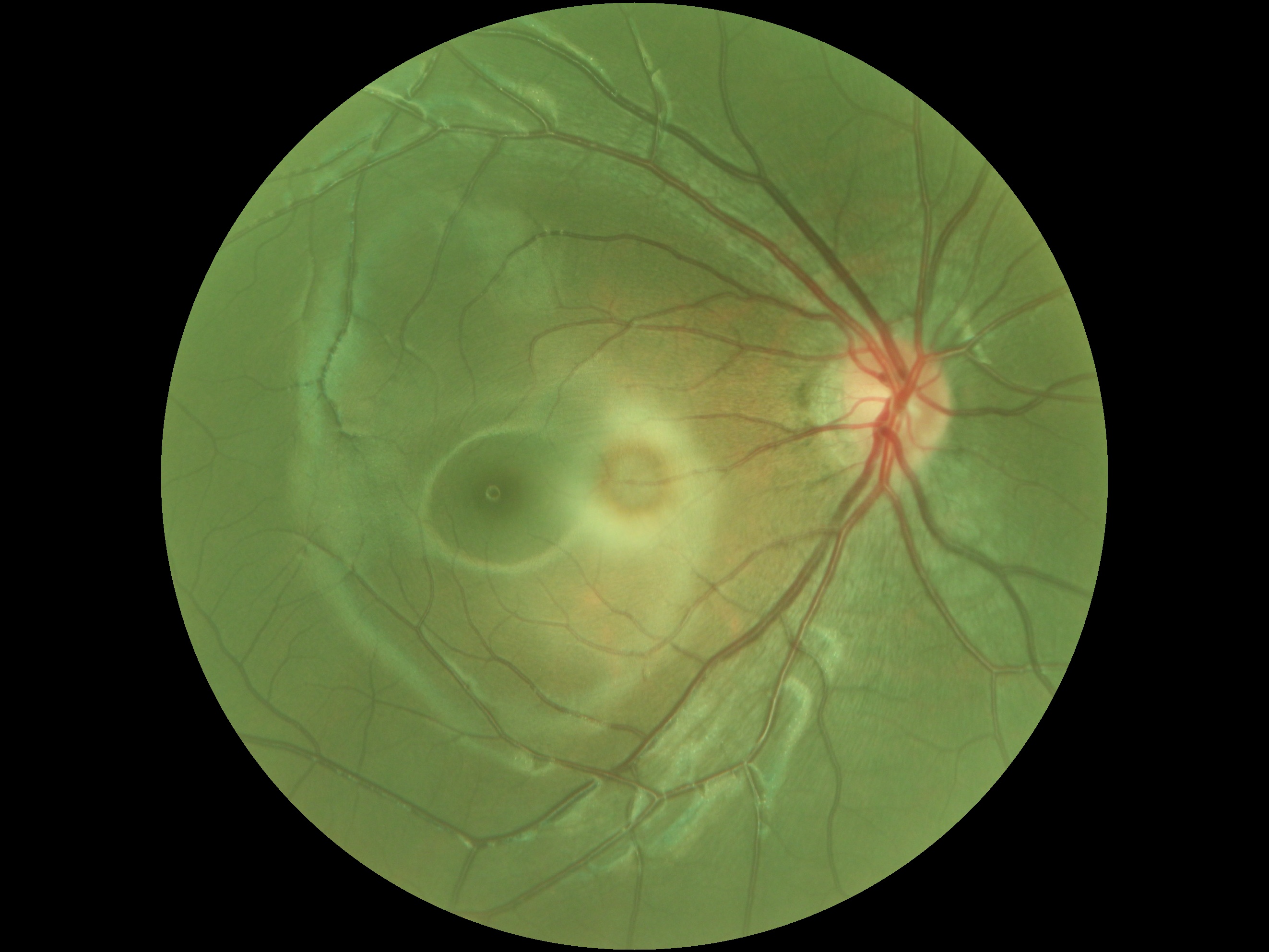

Supplement: S4 File — (ZIP) [file pone.0324352.s004.zip › Original fundus photographs (2)/Subject 90/OD_20230611548085_20230615152522_2.jpg]

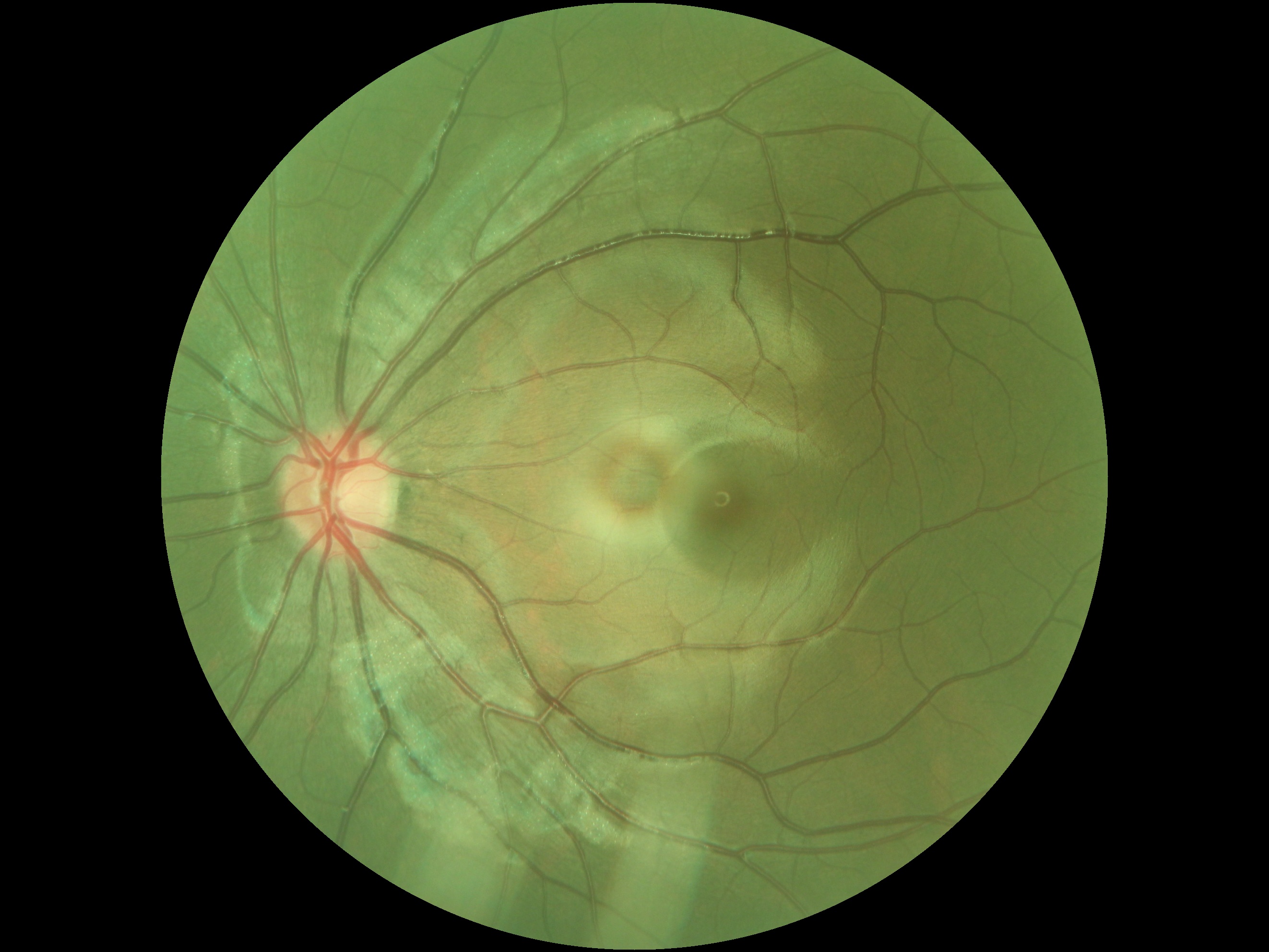

Supplement: S4 File — (ZIP) [file pone.0324352.s004.zip › Original fundus photographs (2)/Subject 90/OS_20230611548085_20230615152459_1.jpg]

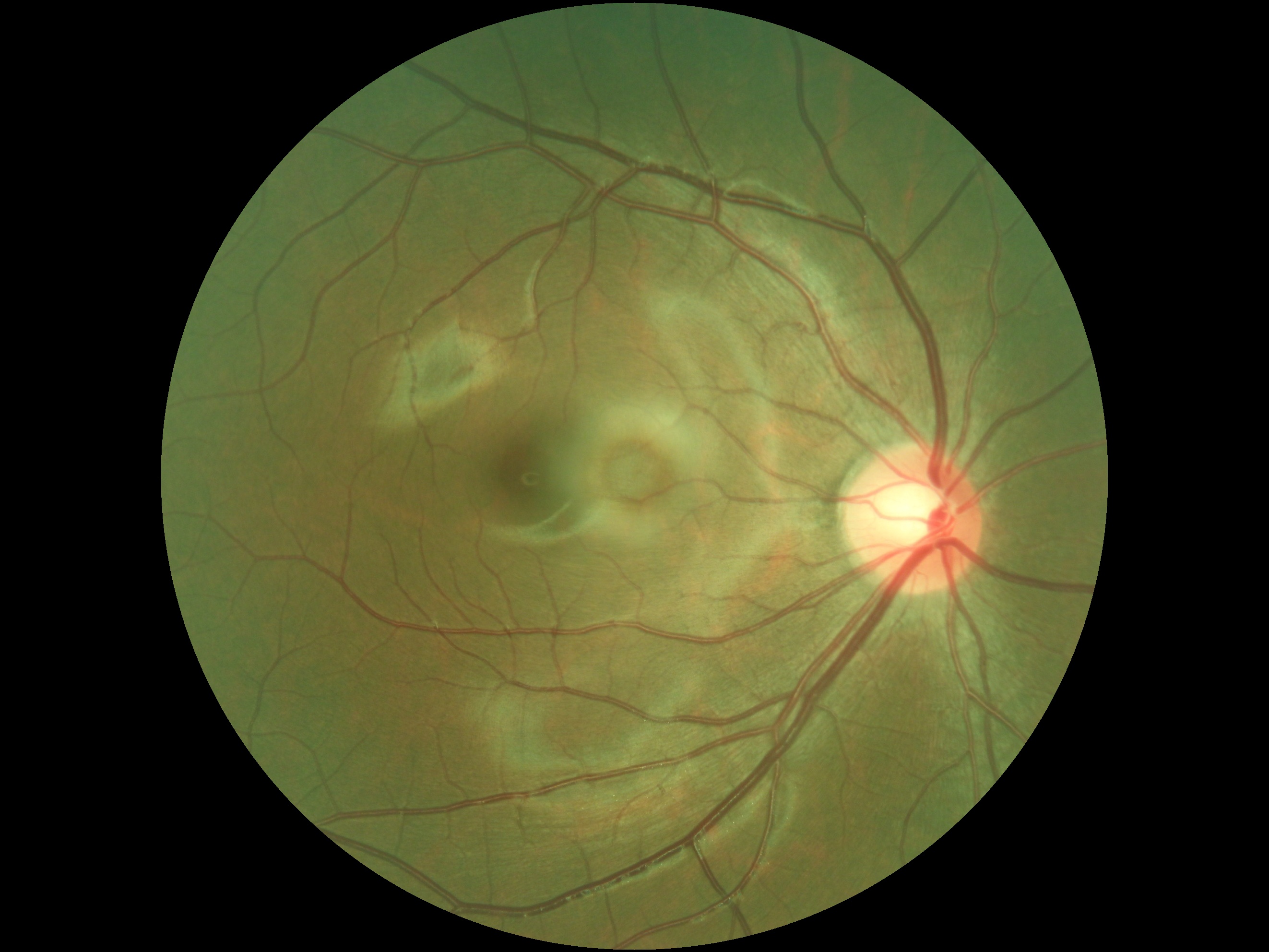

Supplement: S4 File — (ZIP) [file pone.0324352.s004.zip › Original fundus photographs (2)/Subject 91/OD_20230611992269_20230615105735_2.jpg]

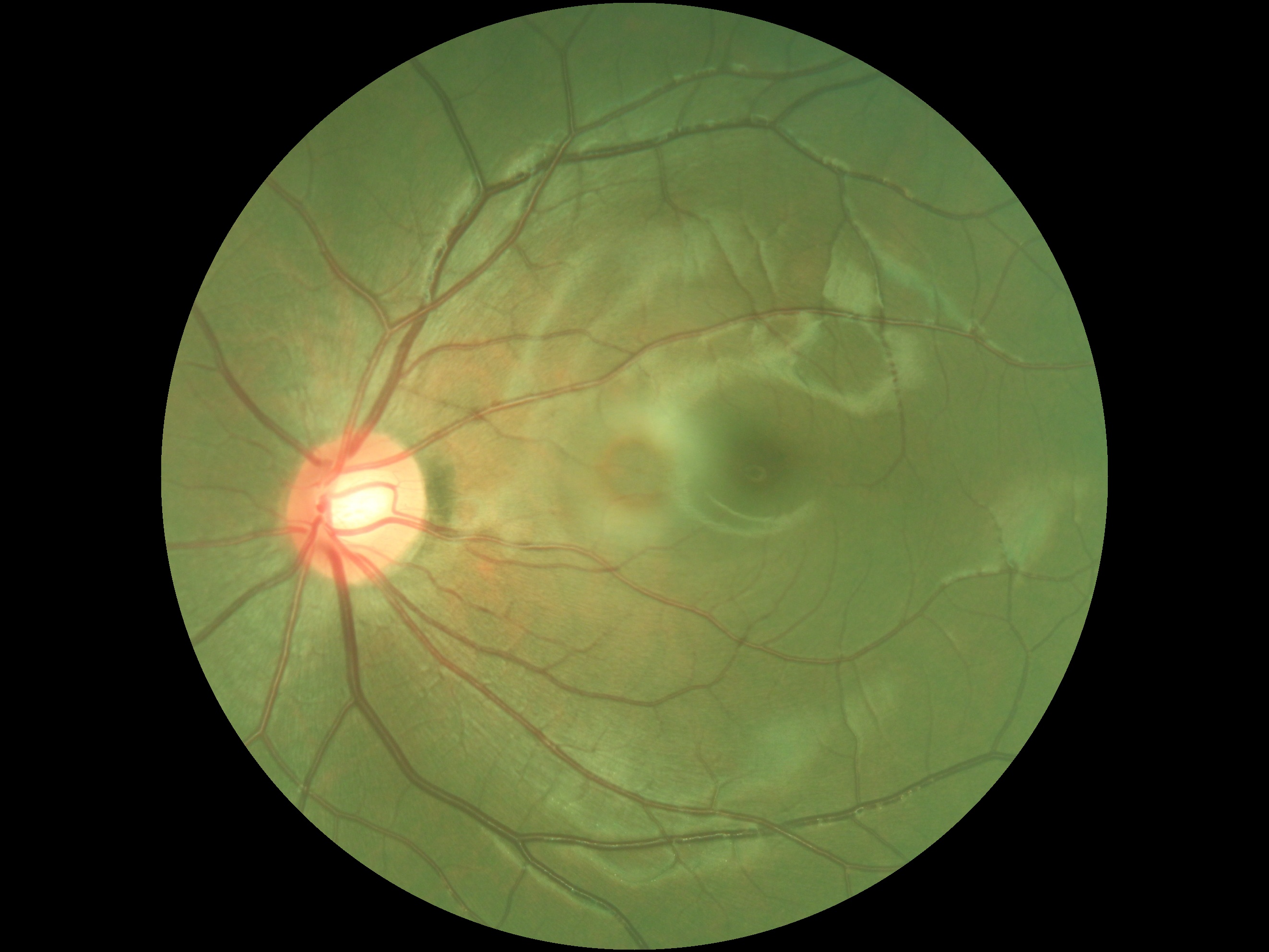

Supplement: S4 File — (ZIP) [file pone.0324352.s004.zip › Original fundus photographs (2)/Subject 91/OS_20230611992269_20230615105631_1.jpg]

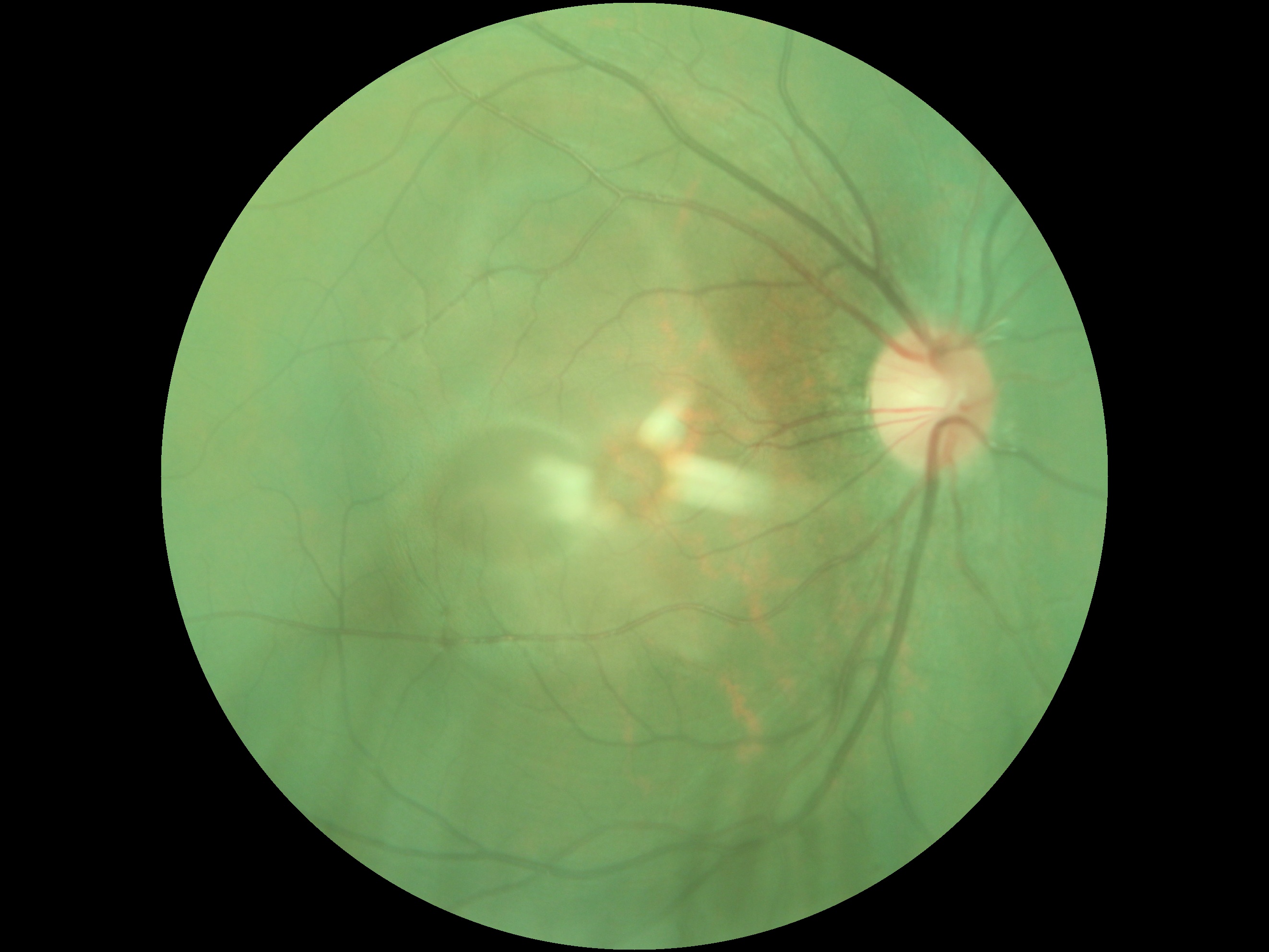

Supplement: S4 File — (ZIP) [file pone.0324352.s004.zip › Original fundus photographs (2)/Subject 92/OD_20230611585060_20230613110149_1.jpg]

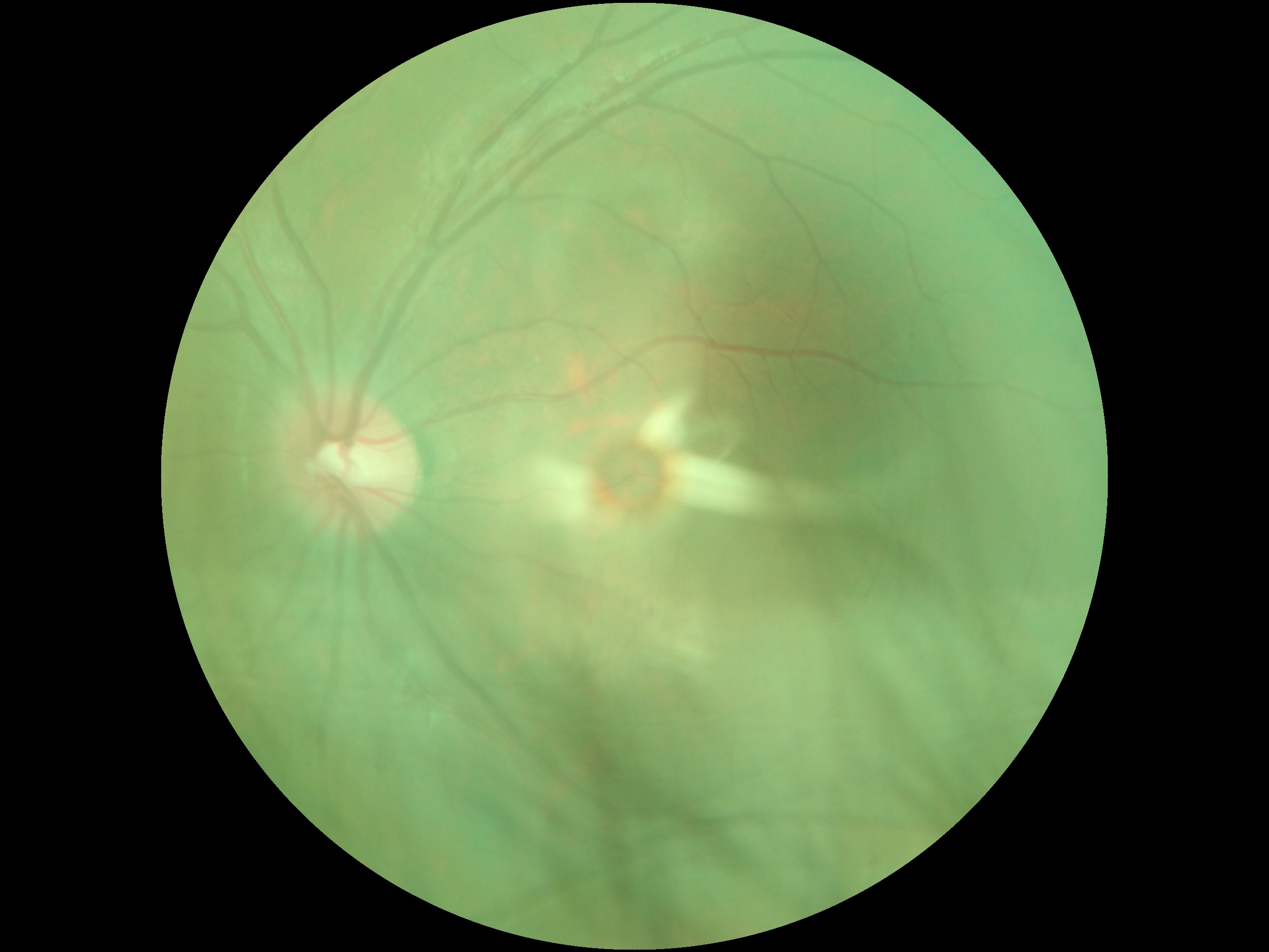

Supplement: S4 File — (ZIP) [file pone.0324352.s004.zip › Original fundus photographs (2)/Subject 92/OS_20230611585060_20230613110300_3.jpg]

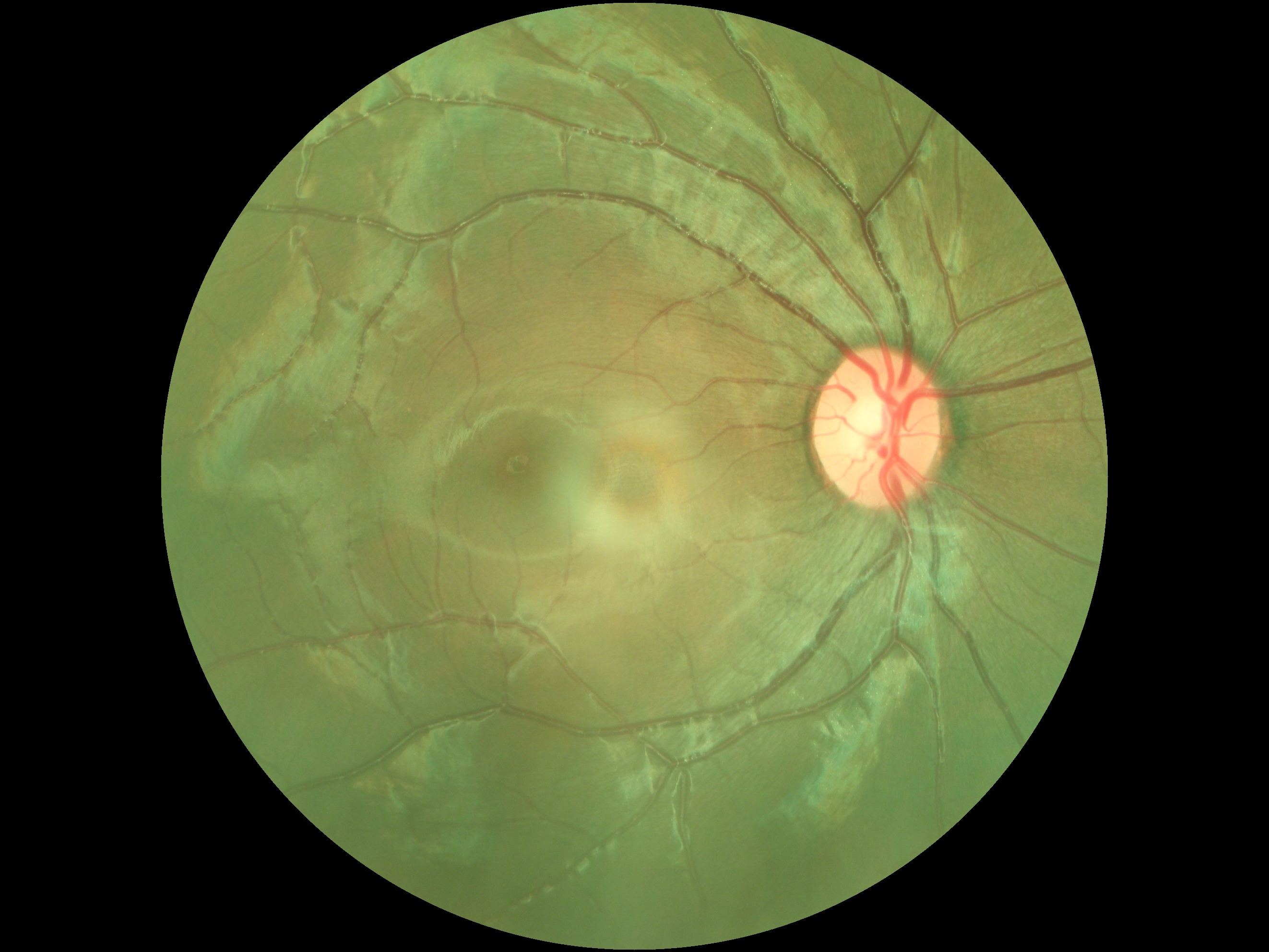

Supplement: S4 File — (ZIP) [file pone.0324352.s004.zip › Original fundus photographs (2)/Subject 93/OD_20230611291130_20230614162051_2.jpg]

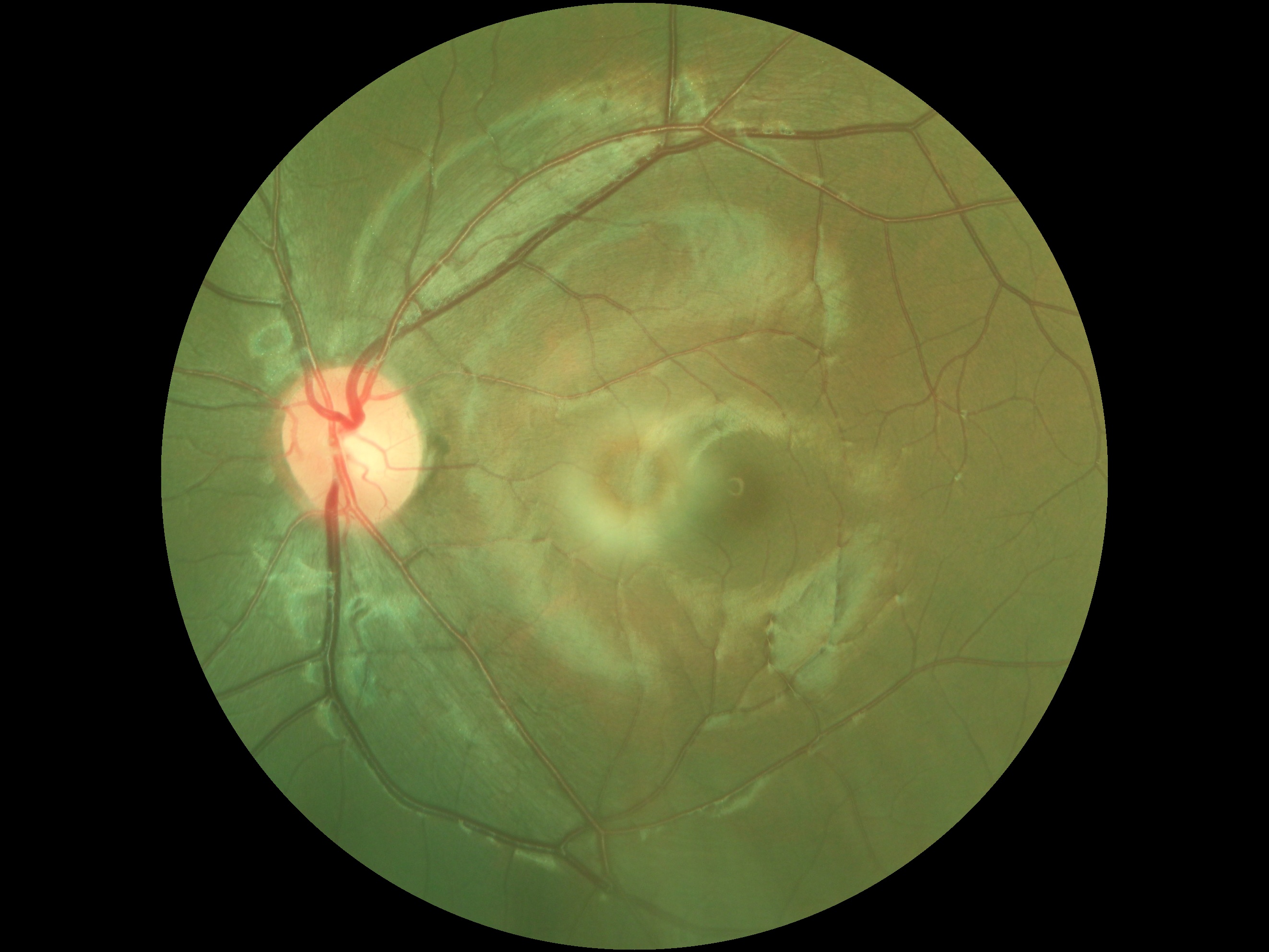

Supplement: S4 File — (ZIP) [file pone.0324352.s004.zip › Original fundus photographs (2)/Subject 93/OS_20230611291130_20230614162222_2.jpg]

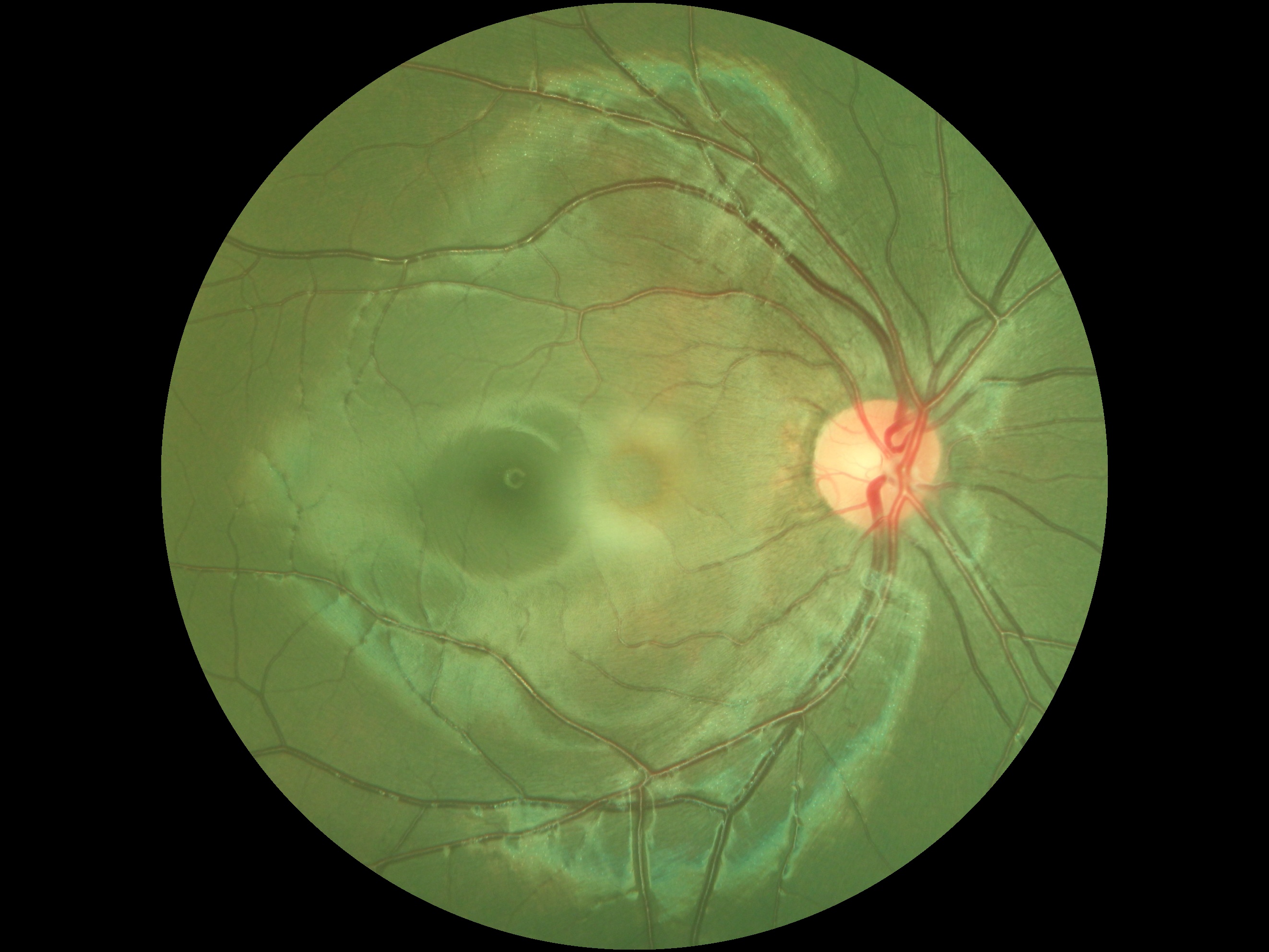

Supplement: S4 File — (ZIP) [file pone.0324352.s004.zip › Original fundus photographs (2)/Subject 94/OD_20230615830061_20230615153410_1.jpg]

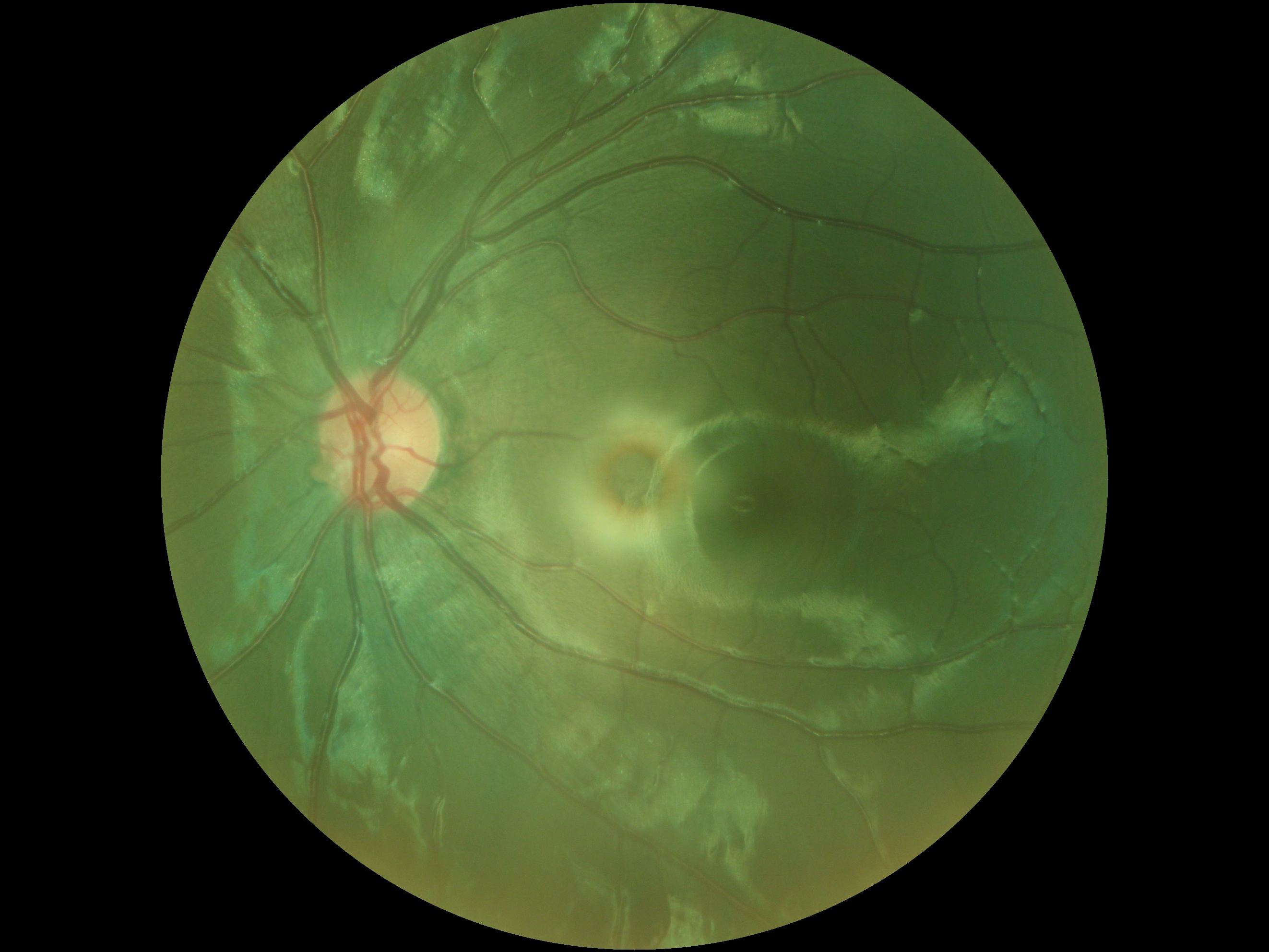

Supplement: S4 File — (ZIP) [file pone.0324352.s004.zip › Original fundus photographs (2)/Subject 94/OS_20230615830061_20230615153422_2.jpg]

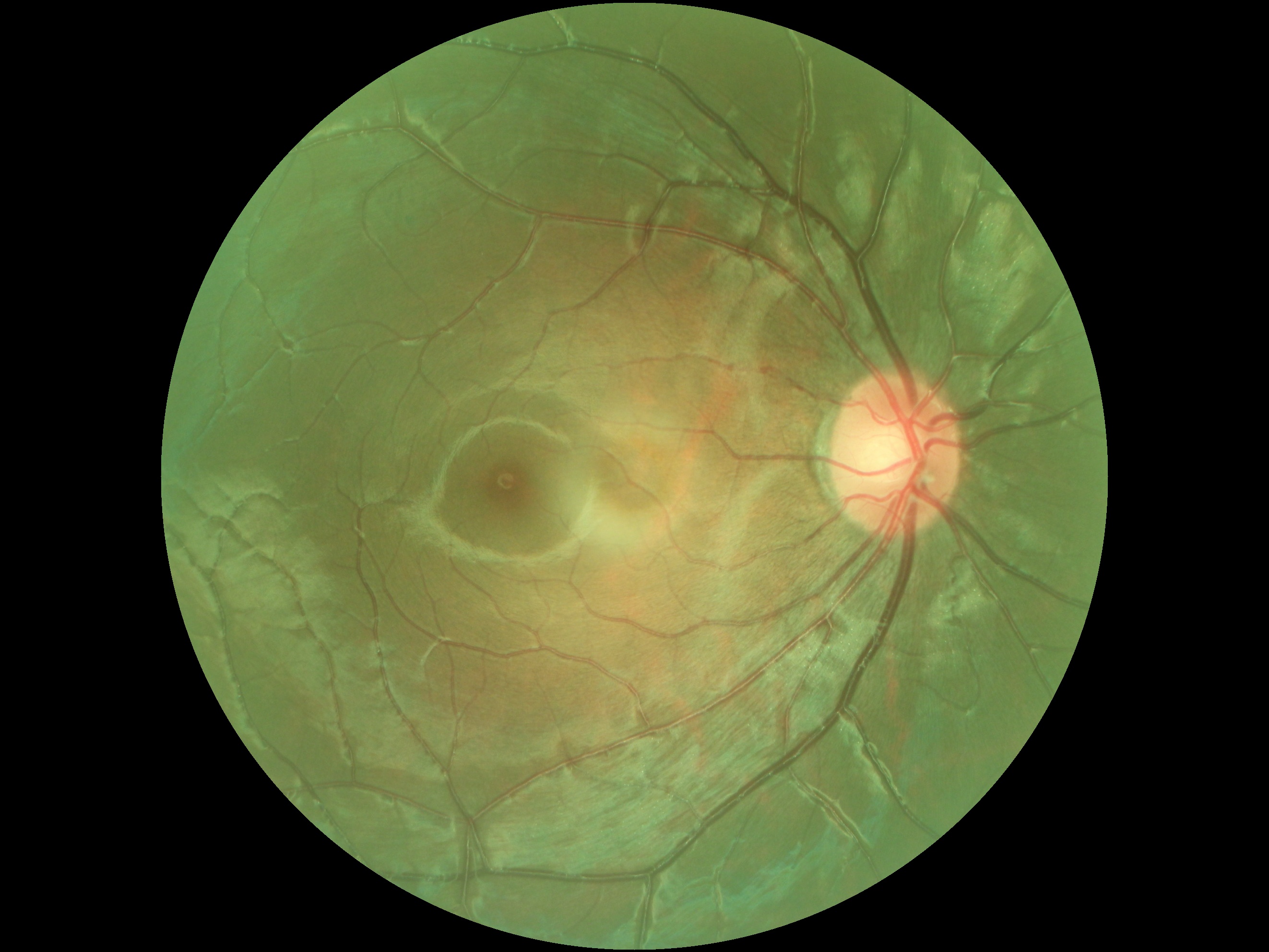

Supplement: S4 File — (ZIP) [file pone.0324352.s004.zip › Original fundus photographs (2)/Subject 95/OD_20230612983003_20230614160747_2.jpg]

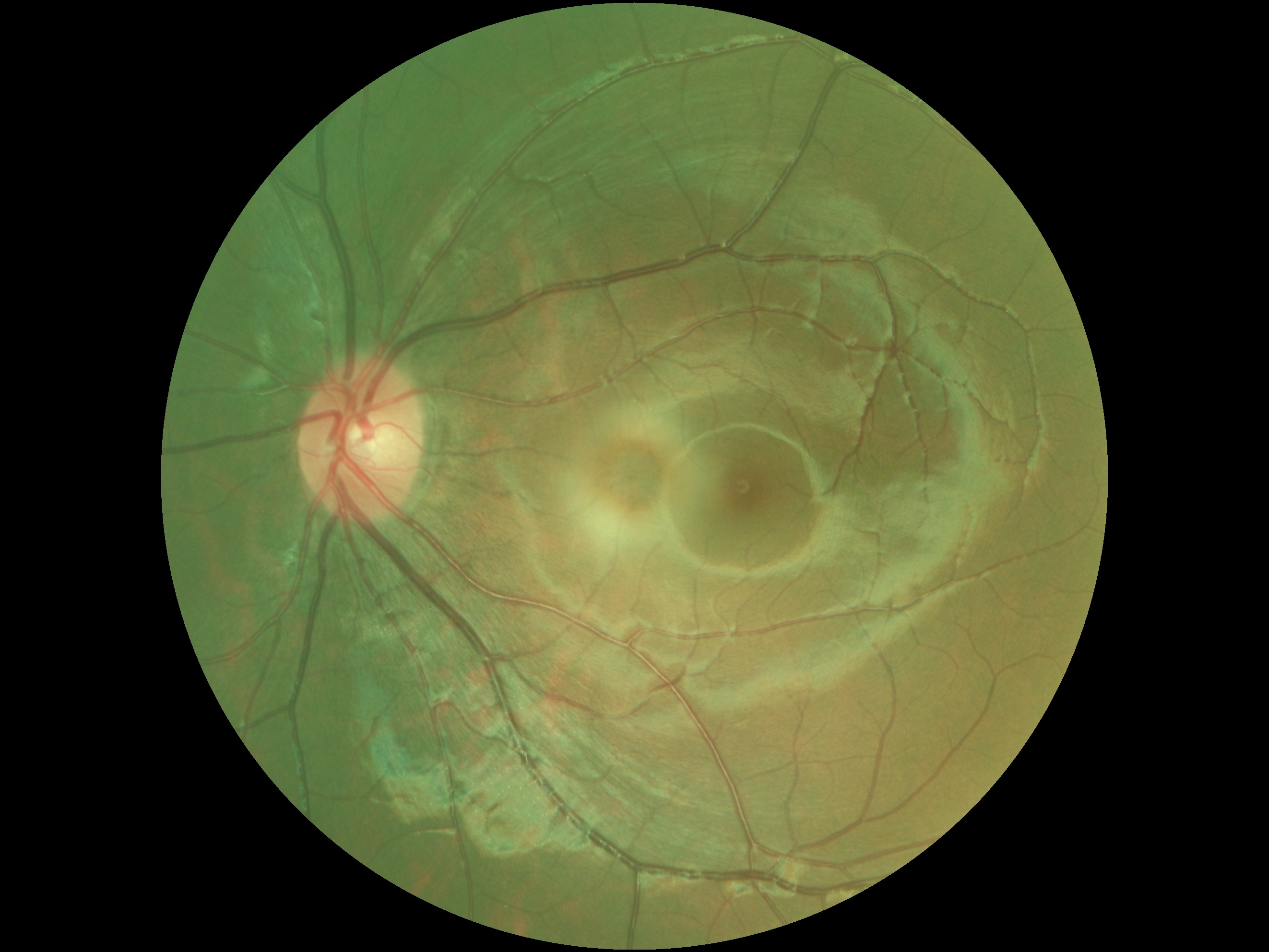

Supplement: S4 File — (ZIP) [file pone.0324352.s004.zip › Original fundus photographs (2)/Subject 95/OS_20230612983003_20230614160659_1.jpg]

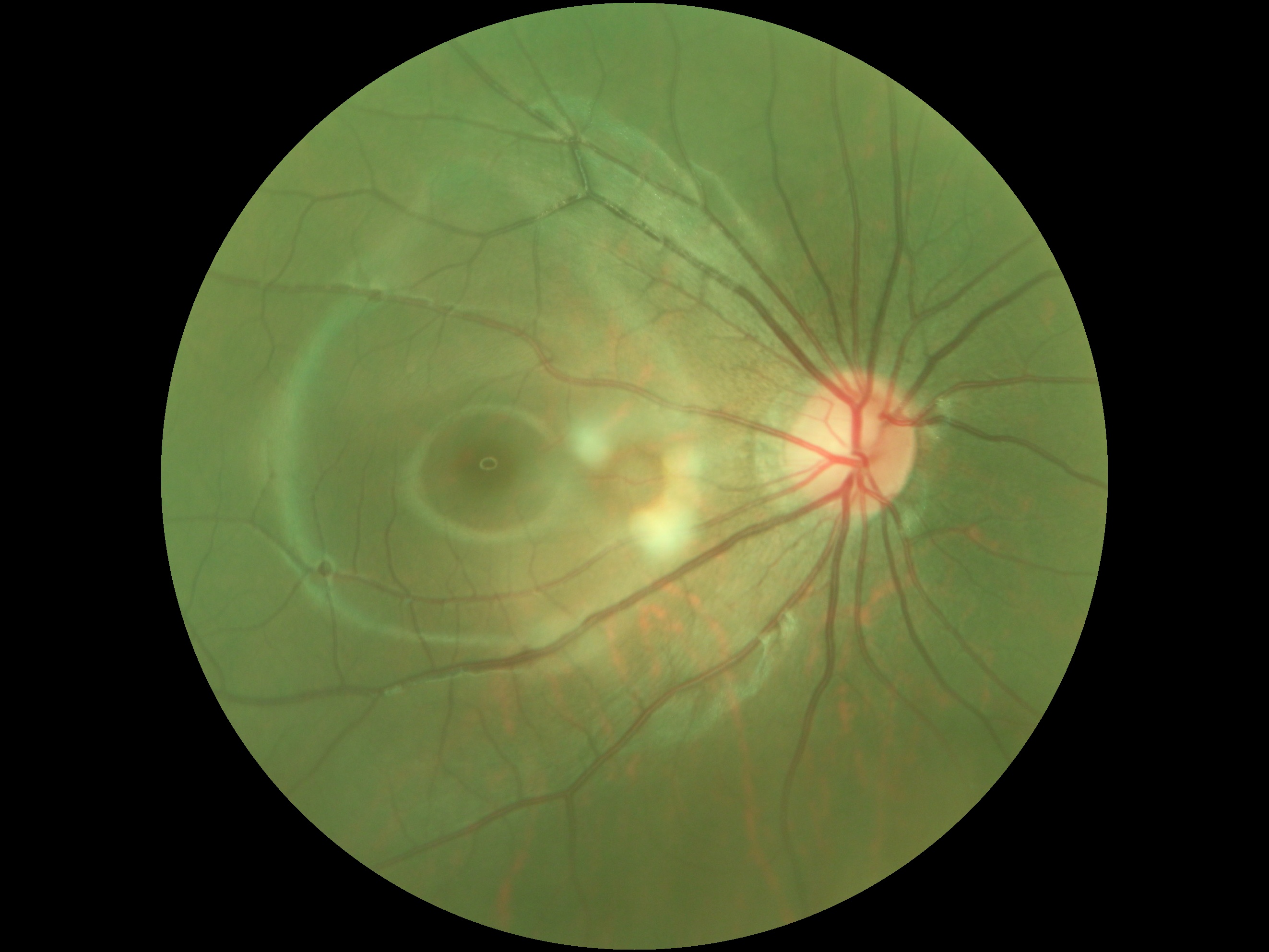

Supplement: S4 File — (ZIP) [file pone.0324352.s004.zip › Original fundus photographs (2)/Subject 96/OD_20230611148057_20230615165421_4.jpg]

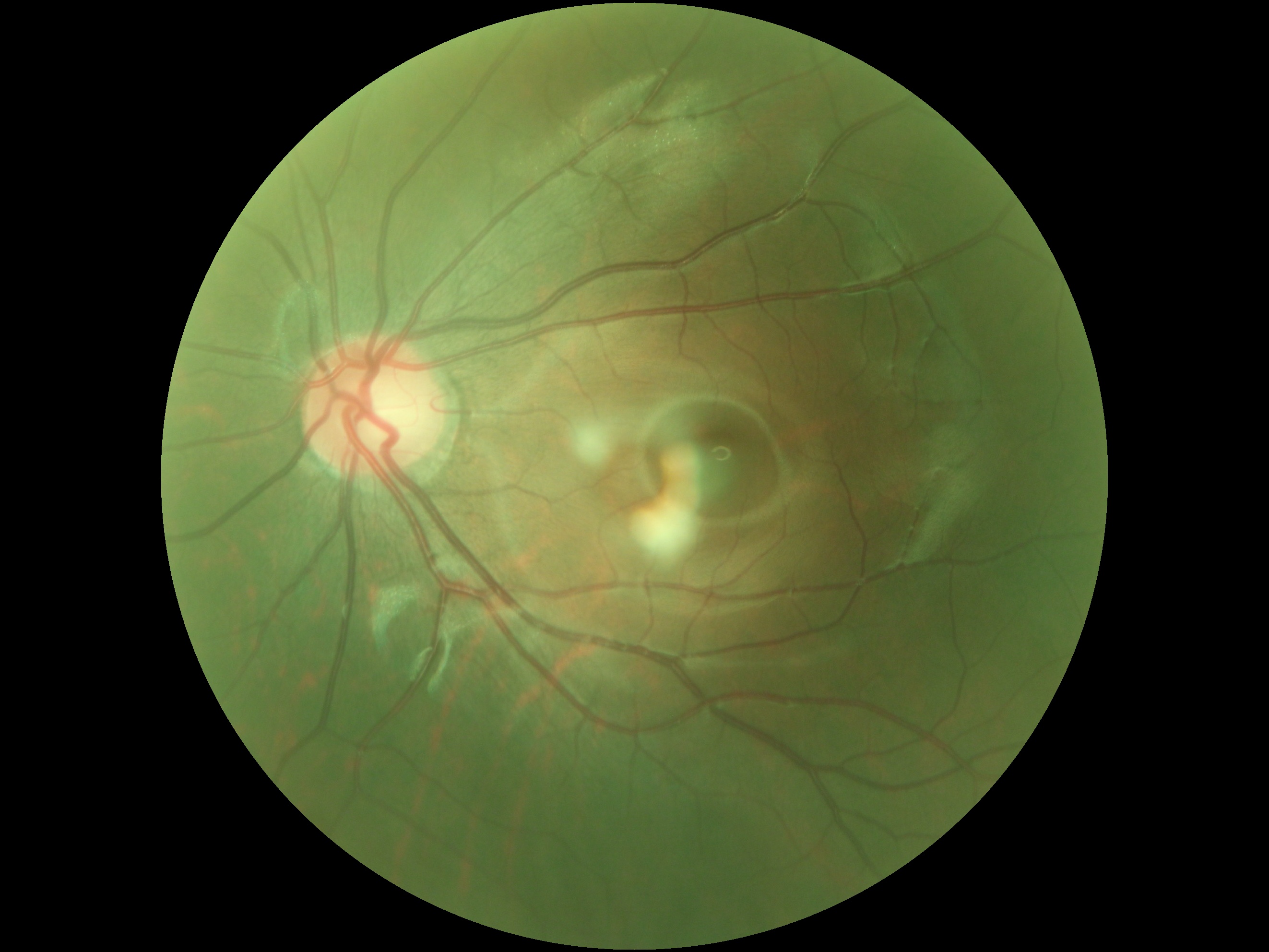

Supplement: S4 File — (ZIP) [file pone.0324352.s004.zip › Original fundus photographs (2)/Subject 96/OS_20230611148057_20230615165403_3.jpg]

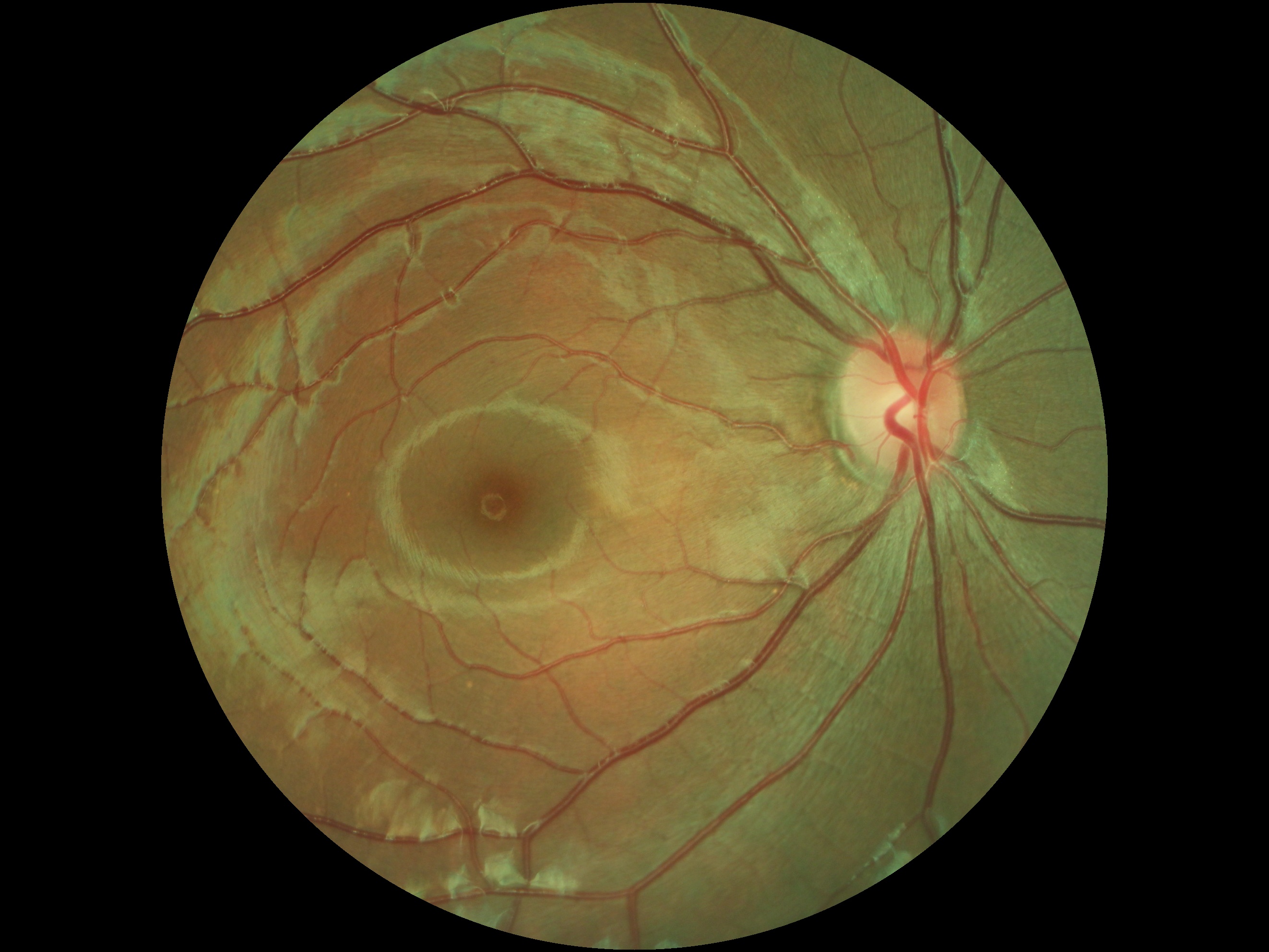

Supplement: S4 File — (ZIP) [file pone.0324352.s004.zip › Original fundus photographs (2)/Subject 97/OD_20230611421046_20230613104011_1.jpg]

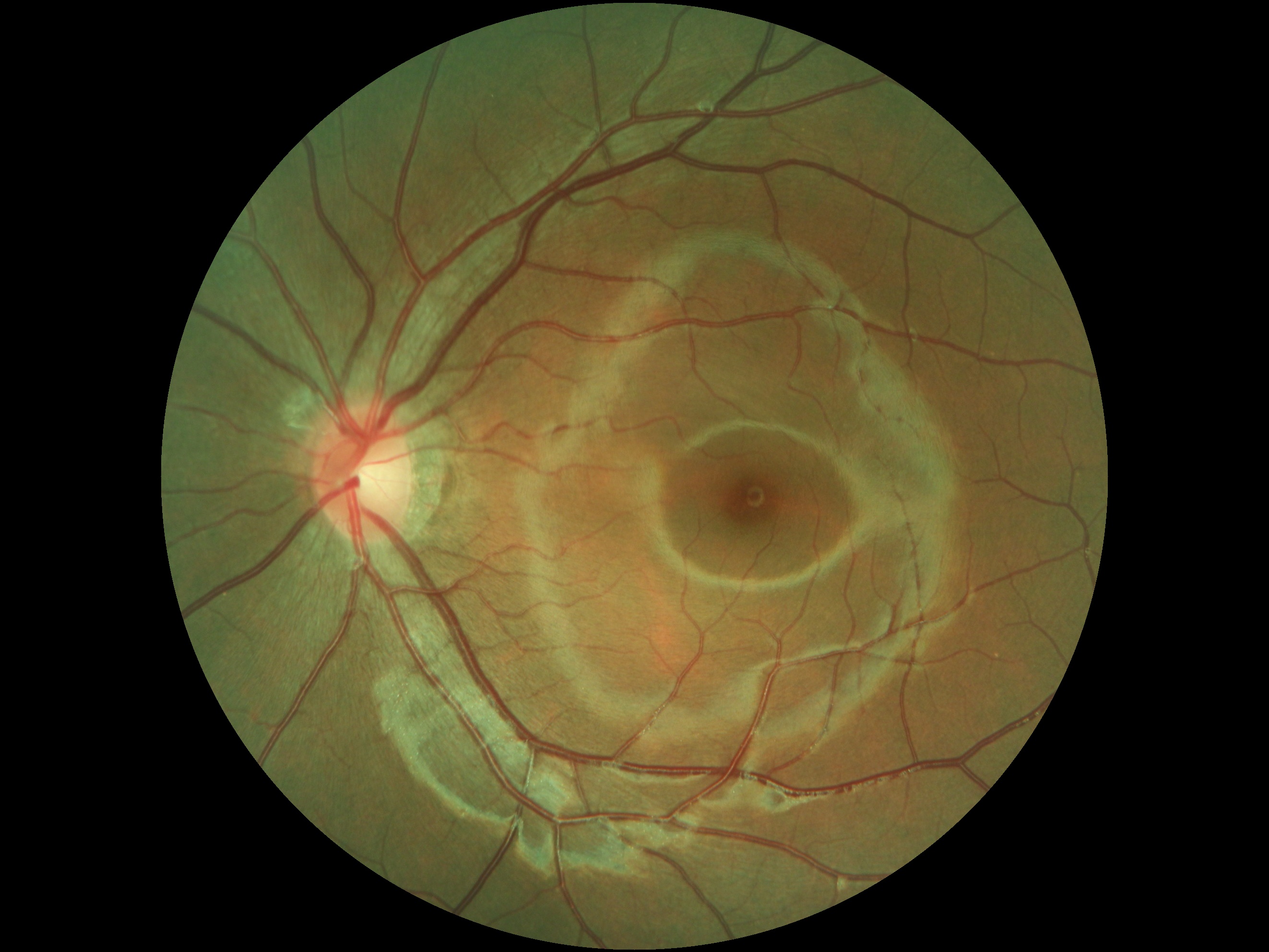

Supplement: S4 File — (ZIP) [file pone.0324352.s004.zip › Original fundus photographs (2)/Subject 97/OS_20230611421046_20230613104110_3.jpg]

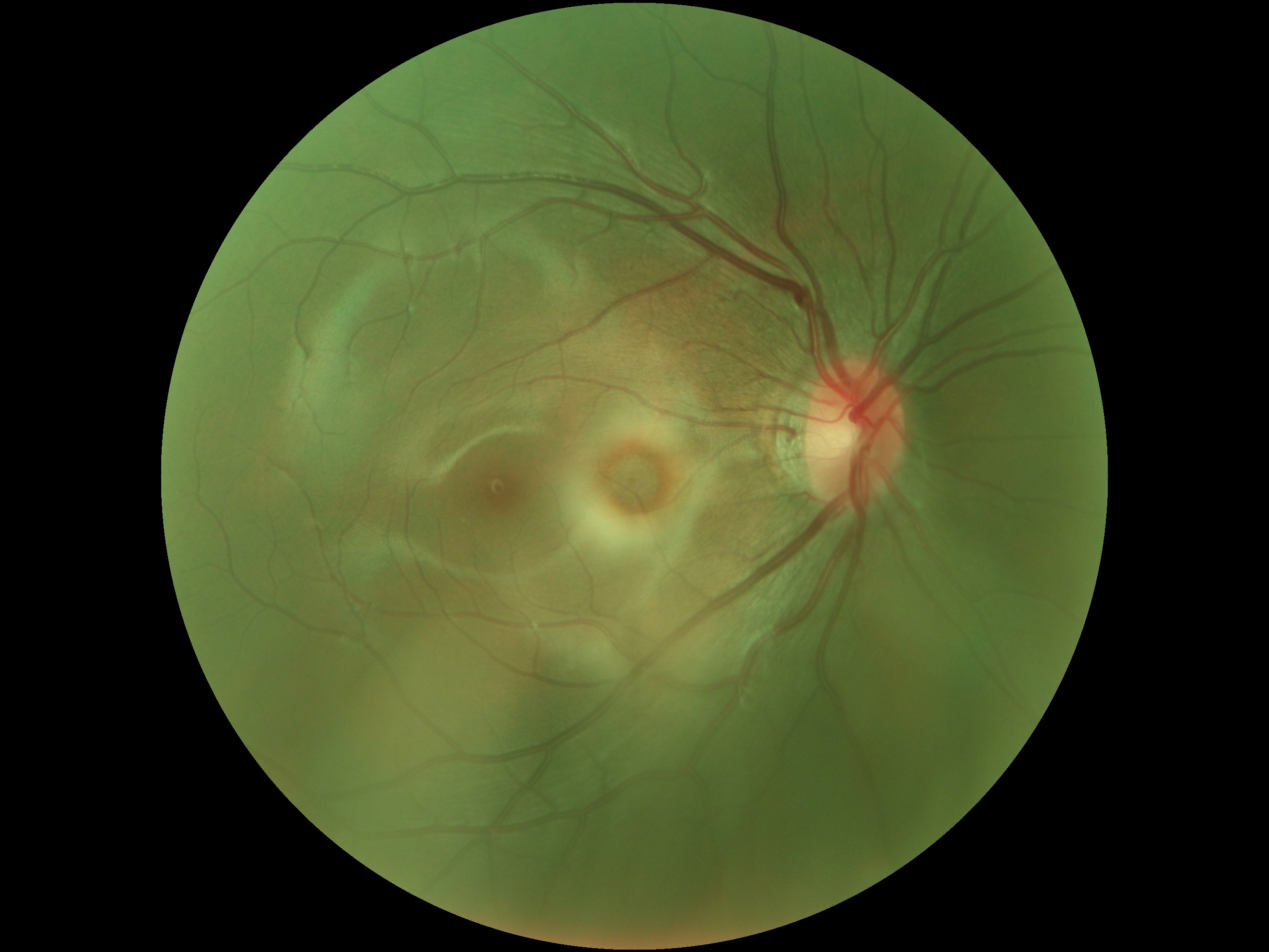

Supplement: S4 File — (ZIP) [file pone.0324352.s004.zip › Original fundus photographs (2)/Subject 98/OD_20230615149048_20230615114428_1.jpg]

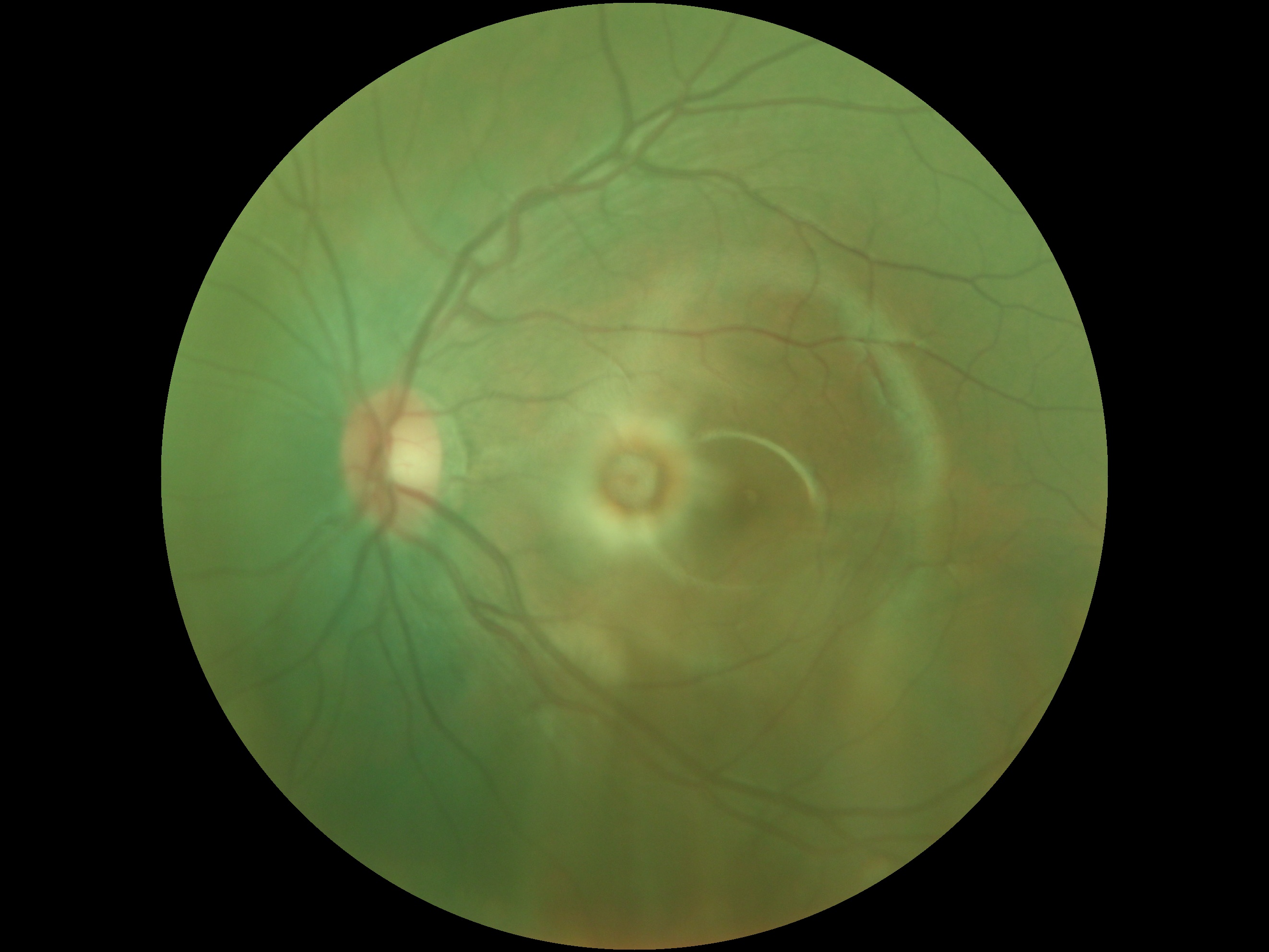

Supplement: S4 File — (ZIP) [file pone.0324352.s004.zip › Original fundus photographs (2)/Subject 98/OS_20230615149048_20230615114455_2.jpg]

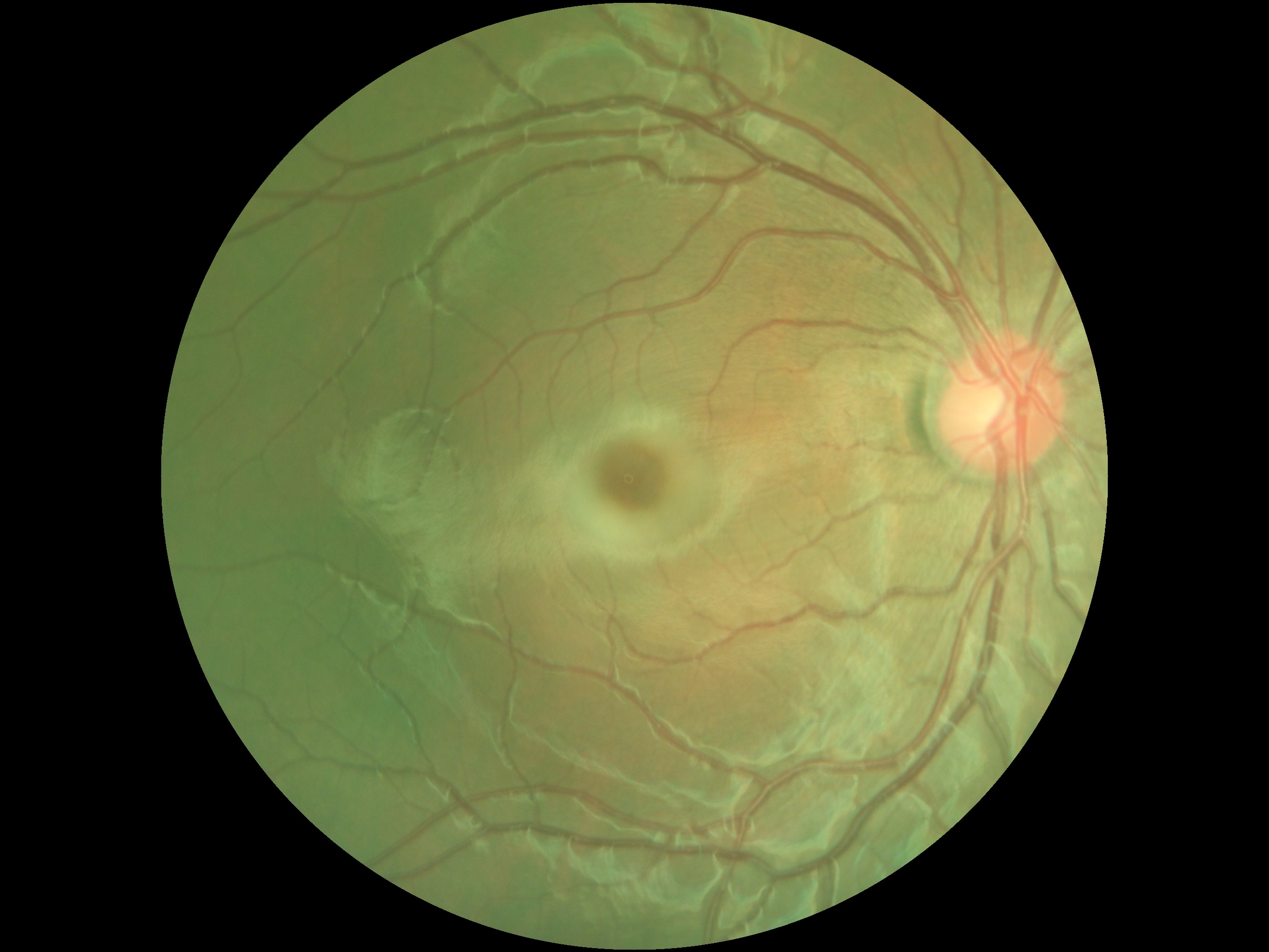

Supplement: S4 File — (ZIP) [file pone.0324352.s004.zip › Original fundus photographs (2)/Subject 99/OD_20230611444173_20230614103240_1.jpg]

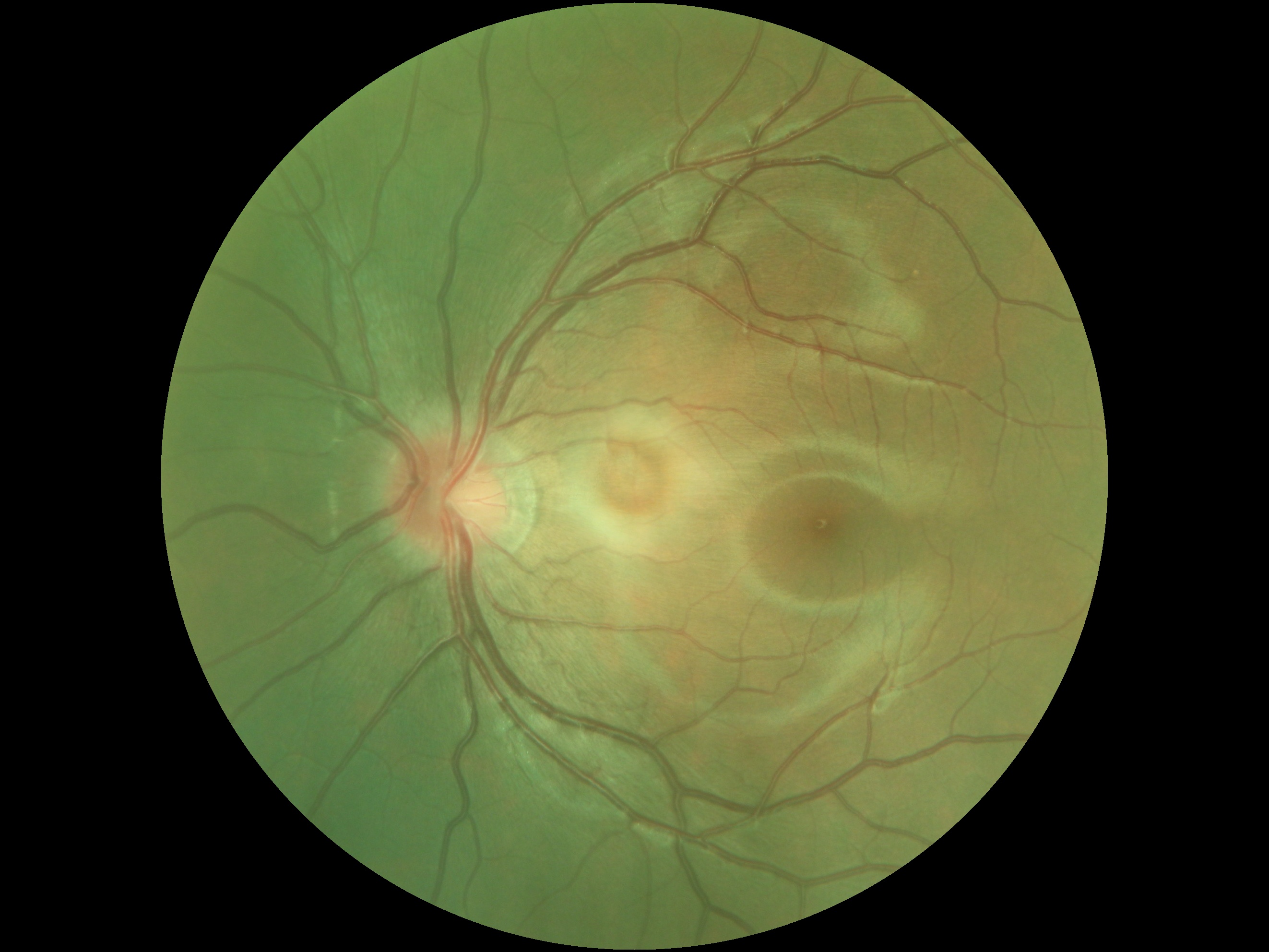

Supplement: S4 File — (ZIP) [file pone.0324352.s004.zip › Original fundus photographs (2)/Subject 99/OS_20230611444173_20230614103318_2.jpg]
