# Supplementary material for: Research on the correlation between retinal vascular parameters and axial length in children using an AI-based fundus image analysis system
Source: PLoS One. 2025 Jun 17;20(6):e0324352. doi: 10.1371/journal.pone.0324352 (PMC12173413; doi:10.1371/journal.pone.0324352)
Supplement: Attachment — Submitted filename: Response to Reviewers.docx [file pone.0324352.s007.docx]

Dear Reviewers,

We deeply appreciate your invaluable feedback on our manuscript, titled "Research on the Correlation between Retinal Vascular Parameters and Axial Length in Children Based on Artificial Intelligence." Recognizing the effort and time you've invested in the review, we sincerely thank you. Your insightful comments have enriched our understanding of the research and provided us with valuable suggestions. We have carefully considered all your recommendations and will address them in the revised manuscript. Here are our point-by-point responses to your main comments:

Reviewer 1:

1.We sincerely appreciate your affirmation of our paper titled "Research on the Correlation between Retinal Vascular Parameters and Axial Length in Children Using an AI-Based Fundus Image Analysis System" [Note: The title has been updated accordingly in this response for consistency]. Your recognition serves as a great encouragement for us.

2.Regarding your concern that the use of the term "Artificial Intelligence" in the title may be misleading, we have carefully considered and revised it. You pointed out that we utilized readily available algorithms within a commercial machine, rather than developing or conducting in-depth research on AI algorithms ourselves. Your observation is highly accurate, and we fully concur with your perspective. Therefore, we have modified the title from "Based on Artificial Intelligence" to "Using an AI-Based Fundus Image Analysis System," to more accurately reflect that we actually employed an AI-based fundus image analysis system in our research, rather than specifically referring to our research or development of AI algorithms.

3.In the Methods section, to directly address your comments, we have provided a more detailed description of the AI system's usage and included a brief explanation of the AI system. We explicitly state that the EVision AI Fundus Image Analysis System is a commercial software that utilizes pre-trained algorithms for automatic extraction of retinal vascular parameters. This description helps eliminate potential misunderstandings and more clearly presents the technical means adopted in our research.

Reviewer 2

1. We have provided a more detailed description of the imaging process and inserted a flowchart to clearly illustrate the various stages of imaging. These modifications will aid readers in better understanding our research methodology and results. Additionally, we have taken note of the reviewer's suggestions regarding the legends of Figures 1-4 and have made corresponding revisions. We have transferred some explanatory details to the main text to make the legends more concise and straightforward. These changes will enhance the readability and clarity of the figures.
2. In response to your comment on page 10, lines 19-21, regarding the lack of sufficient detail and the need for more concrete data in the discussion about mitigating manual measurement errors, we have conducted thorough reflections and made corresponding revisions.To strengthen the argumentation in this section, we have revisited the experimental data and extracted specific statistical indicators related to manual measurement errors. By comparing the results of automatic measurements with those of manual measurements, we found that the artificial intelligence-based system reduced the standard deviation of retinal vessel diameter measurements by 0.12 mm (p < 0.05). This result significantly demonstrates the improvement in accuracy and consistency of automatic measurements. We have included this finding in the discussion section for further elucidation.
3. Regarding the issue you previously pointed out concerning the overlap between the conclusion and results sections, we have conducted thorough reflections and made the necessary revisions. Following your advice, I have endeavored to streamline the restatement of specific research findings, instead emphasizing more on the significance of this study, its contributions to the field, and potential future research directions. Additionally, I have retained the mention of AI technology's potential in the conclusion section and expanded on specific suggestions for areas that future research could explore, aiming to provide readers with a broader perspective and room for contemplation.Furthermore, during the revision process, we paid particular attention to maintaining the paper's logical flow and coherence, ensuring that the conclusion section is both concise and clear while accurately reflecting the research's core value and potential impact. We hope that these adjustments will further enhance the clarity and focus of the paper's conclusion section, making it better serve the understanding and inspiration of readers.

4.Based on your suggestions, we have made the following modifications to the tables in the paper: For data with P-values less than 0.05, we have bolded them (or converted them to superscript format, depending on the format you prefer) to more clearly showcase the significance of these data. Additionally, we have adjusted other table formats to enhance overall readability and data clarity.

Best regards,

Chaoyang Zhao
